# Genome‐wide DNA methylation analysis identifies *MEGF10* as a novel epigenetically repressed candidate tumor suppressor gene in neuroblastoma

**DOI:** 10.1002/mc.22591

**Published:** 2016-11-29

**Authors:** Jessica Charlet, Ayumi Tomari, Anthony R. Dallosso, Marianna Szemes, Martina Kaselova, Thomas J. Curry, Bader Almutairi, Heather C. Etchevers, Carmel McConville, Karim T. A. Malik, Keith W. Brown

**Affiliations:** ^1^ School of Cellular and Molecular MedicineUniversity of BristolBristolUK; ^2^ Faculté de MédecineAix‐Marseille University, GMGF, UMR_S910MarseilleFrance; ^3^ Faculté de MédecineINSERM U910MarseilleFrance; ^4^ Institute of Cancer & Genomic SciencesUniversity of BirminghamUK

**Keywords:** epigenetics, DNA methylation, histone methylation, neuroblastoma, *MEGF10*

## Abstract

Neuroblastoma is a childhood cancer in which many children still have poor outcomes, emphasising the need to better understand its pathogenesis. Despite recent genome‐wide mutation analyses, many primary neuroblastomas do not contain recognizable driver mutations, implicating alternate molecular pathologies such as epigenetic alterations. To discover genes that become epigenetically deregulated during neuroblastoma tumorigenesis, we took the novel approach of comparing neuroblastomas to neural crest precursor cells, using genome‐wide DNA methylation analysis. We identified 93 genes that were significantly differentially methylated of which 26 (28%) were hypermethylated and 67 (72%) were hypomethylated. Concentrating on hypermethylated genes to identify candidate tumor suppressor loci, we found the cell engulfment and adhesion factor gene *MEGF10* to be epigenetically repressed by DNA hypermethylation or by H3K27/K9 methylation in neuroblastoma cell lines. *MEGF10* showed significantly down‐regulated expression in neuroblastoma tumor samples; furthermore patients with the lowest‐expressing tumors had reduced relapse‐free survival. Our functional studies showed that knock‐down of *MEGF10* expression in neuroblastoma cell lines promoted cell growth, consistent with *MEGF10* acting as a clinically relevant, epigenetically deregulated neuroblastoma tumor suppressor gene. © 2016 The Authors. *Molecular Carcinogenesis* Published by Wiley Periodicals, Inc.

**Abbreviations**
hNCC: human neural crest cells
FBS: fetal bovine serum
azadC: 5 aza‐2′‐deoxycytidine
TSA: trichostatin A
DZNep: 3‐deazaneplanocin
MCIP: methyl CpG immunoprecipitation
ChIP: chromatin immunoprecipitation
PRC: polycomb repressive complex
NB: neuroblastoma

## INTRODUCTION

Neuroblastoma, one of the commonest solid tumors of childhood, is an embryonal malignancy that arises via defective differentiation of neural crest cells that give rise to the sympathetic nervous system 1, 2. Neuroblastoma patients have a poor outcome compared to many other childhood cancer sufferers 2, mostly attributable to older children who present with metastatic disease 2, 3.

Compared to other cancers, neuroblastomas demonstrate relatively few mutations 4, 5, 6, although copy‐number changes are common, for example, loss of chromosomes 1p and 11q and gain of 17q 2, 3. Oncogene activation occurs by *MYCN* amplification in high‐risk neuroblastomas 7 and by *ALK* mutation in familial neuroblastoma and in about 10% of sporadic cases 8, 9, 10. Tumor suppressor genes found infrequently mutated in neuroblastoma include *PHOX2B* and *NF1* 11, 12. Genomic sequencing of neuroblastomas has identified additional mutated genes such as the chromatin remodeling genes *ATRX*, *ARID1A*, and *ARID1B*, components of the RAC‐RHO pathway 4, 5, 6 and rearrangements activating the TERT gene 13, 14. Relapsed neuroblastomas demonstrate increased numbers of mutations during disease progression 15, 16.

The lack of identified driver genetic mutations in many cases of neuroblastoma 1 underlines the need to evaluate alternative mechanisms of pathogenesis, including epigenetic aberrations such as DNA methylation 17, which constitute some of the earliest changes in carcinogenesis 18.

Epigenetic deregulation has been shown to play an important role in neuroblastoma pathogenesis, silencing neuroblastoma suppressor genes by aberrant promoter DNA hypermethylation, for example, *RASSF1A*, *CASP8*, and *DCR2* 19, 20, 21, 22, or by aberrant histone methylation, for example, CASZ1, CLU, RUNX3, NGFR 23, and p14^ARF^ 24. DNA hypermethylation of both individual genes and multiple CpG islands has been associated with poor outcome in neuroblastoma 20, 21, 25, 26, 27.

Despite previous gene‐specific analyses of DNA methylation (as discussed above) and more recent genome‐wide analyses 28, 29, 30, 31, 32, 33, 34, it remains unclear which epigenetic alterations are critical for neuroblastoma pathogenesis and what functional roles are played by the affected genes. To address this question, we have taken a novel epigenomic approach, using genome‐wide DNA methylation analysis to compare neuroblastoma cells to their putative normal precursors, human neural crest cells (hNCC). We have identified a series of genes that are differentially methylated in neuroblastoma cells compared to normal human neural crest cells. One of these genes is *MEGF10*, for which we demonstrate growth‐repressive properties predicted for a tumor suppressor gene.

## MATERIALS AND METHODS

### Cell Culture and Neuroblastoma Tumor Samples

Cell lines were obtained from ECACC, apart from BCH‐N‐DW, which is a novel neuroblastoma cell line derived from a bone marrow biopsy (Table S1; C. McConville, unpublished data), SHEP Tet‐21/N which was a kind gift from Prof. M. Schwab, and SK‐N‐AS MYCN‐ER which was a kind gift from Prof. A Sala. Cell lines except SHEP Tet‐21/N and SK‐N‐AS MYCN‐ER were cultured in DMEM/F12‐HAM medium (Sigma, Gillingham, Dorset, UK) supplemented with 10% fetal bovine serum (FBS), 100 U/ml penicillin, 0.1 mg/ml streptomycin, 2 mM L‐glutamine, and 1% non‐essential amino acids (Sigma) at 37°C in a 5% CO_2_ incubator. SH‐EP TET‐21/N cells 35 were cultured in RPMI1640 medium (Invitrogen, Paisley, UK) containing 10% tetracycline‐free FBS (Bioclear, Calne, Wiltshire, UK), with other additives as above and for MYCN silencing 1 μg/ml tetracycline (Sigma) was added. SK‐N‐AS MYCN‐ER cells 36 were cultured in DMEM/F12 containing 10% charcoal‐stripped FBS (Appleton Woods, Birmingham, UK) with other additives as above and for MYCN induction 400 nM 4‐hydroxytamoxifen (Sigma) was added. Neural crest cells were cultured as described previously 37.

Neuroblastoma tumor samples (Table S2) were obtained from Bristol and Birmingham Children's hospitals with appropriate local ethical approval and used as specified in the UK Human Tissue Act.

### 5‐Aza‐2′‐Deoxycytidine, 3‐Deazaneplanocin A, GSK343, and UNC0638 Treatment

Cell lines were incubated in medium containing 2 μM 5‐aza‐2′‐deoxycytidine (azadC; Sigma) plus or minus 0.1 μM trichostatin A (TSA; Sigma), or 0.5 μM—5 μM 3‐deazaneplanocin (DZNep; Cayman, Cambridge Bioscience, Cambridge, UK) plus 0.1 μM TSA, or 10 μM GSK343 (Selleckchem.com; Stratech Scientific, Newmarket, UK) plus 0.1 μM TSA, or 0.1 μM UNC0638 (Sigma) plus 0.1 μM TSA for up to 6 d, with a medium change every 2 d. Control cultures received equivalent volumes of drug solvent (DMSO).

### DNA Extraction and Methyl CpG Immunoprecipitation (MCIP)

An outline of the MCIP workflow is shown in supplementary Figure S1. DNA was extracted with a DNeasy kit (Qiagen, Manchester, UK). MCIP was performed as described in 38 and validated by analysing fractions for their methylated DNA content relative to input by QPCR (QuantiTect SYBR Green; Qiagen), using the following control genes: *WISP3* (hypermethylated), *SNRPN* (imprinted; 50% methylated), and *TBP* (unmethylated). Primer sequences are given in Table S4 and validation results in Figure S2. Methylation‐enriched DNA fractions were co‐hybridized with input DNA on to Human DNA Methylation 385 K Promoter Plus CpG Island Arrays (NimbleGen; Roche, Burgess Hill, Sussex, UK). Statistical analyses employed ChIPMonk software (www.bioinformatics.bbsrc.ac.uk/projects/chipmonk), using windowed T‐tests to identify differentially methylated probes (Figure S1). log_2_ gene methylation levels were derived from the mean probe ratios within 700 bp of the transcriptional start site (Table S3). The data discussed in this publication have been deposited in NCBI's Gene Expression Omnibus 39 and are accessible through GEO Series accession number GSE71958 (http://www.ncbi.nlm.nih.gov/geo/query/acc.cgi?acc = GSE71958).

### Pyrosequencing

DNA was bisulfite converted (EZ DNA Methylation Gold kit; Zymo Research; Cambridge Bioscience, Cambridge, UK), amplified with biotinylated primers (Qiagen) using a Pyromark PCR kit (Qiagen) and pyrosequenced on a PyroMark Q96 instrument (Qiagen). The two assays used for *MEGF10* were Hs‐MEGF10‐01‐PM (sequence analyzed TCGATCGTGAGTCGCCCCTGCCTGAGCGGCTTCCACCGT) and Hs‐MEGF10‐02‐PM (sequence analyzed ACGCGGTTAGCGTYCAAGCAGCGT); both from Qiagen (Figure 3B).

### Chromatin Immunoprecipitation (ChIP)

SHIN cells were fixed in 1% paraformaldehyde for 5 min at room temperature, lyzed, sonicated and the chromatin immunoprecipitated with rabbit anti‐H3K27me3 (Millipore #17‐682, Millipore, Watford, UK), mouse anti‐H3K9me2 (Abcam ab1220, Abcam, Cambridge, UK), or normal rabbit or mouse IgG as a control, using a Magna ChIP G kit (Millipore). Enriched DNA was quantitated by real‐time PCR (QuantiTect SYBR Green; Qiagen) using primers for *GAPDH* and *MEGF10* (Table S4 and Figure S2C).

### MEGF10 Transient Silencing

Cells were transfected with 50 nM of siRNA against *MEGF10* (ON‐TARGET plus SMART pool; Thermo Scientific L‐014897‐01, Thermo Fisher, Hemel Hempstead, UK) or a non‐targeting pool (ON‐TARGET plus Non‐targeting Pool; Thermo Scientific D‐001810‐10), using DUO transfection reagent (Dharmacon, Little Chalfont, Buckinghamshire, UK) and harvested after 72 h.

### RNA Extraction, cDNA Synthesis, and RT‐PCR

Total RNA was extracted with an RNeasy kit (Qiagen), DNase treated with TURBO DNA‐free (Ambion, Thermo Fisher, Hemel Hempstead, UK) and cDNA synthesized using the Thermoscript RT‐PCR system (Invitrogen). Gene‐specific primers (Table S4) were used for end‐point PCR, or QPCR (QuantiTect SYBR Green; Qiagen) on an MX3000P real‐time PCR machine (Stratagene, Cambridge, UK), normalising the amount of target gene to the endogenous level of *TBP*. Human universal RNA (Agilent, Stockport, Cheshire, UK) was used as a reference to standardize results between QPCR batches.

### Protein Extraction and Western Blotting

Cultured cells were washed with ice‐cold PBS and lyzed in cell lysis buffer (Cell Signaling, New England Biolabs, Hitchin, Hertfordshire, UK), with complete mini inhibitors (Roche, Burgess Hill, Sussex, UK) for 10 min on ice, and then sonicated for 5 min at high intermittent pulses (30/30) (Diagenode, Bioruptor, Oxford, UK). Neuroblastoma tumor samples were homogenized in cell lysis buffer, then processed as for cultured cells. Samples were centrifuged for 10 min at 10 000*g* at 4°C to remove any cell debris and typically 25 μg proteins were separated on a SDS–polyacrylamide gel and analyzed by Western blotting. Fetal adrenal protein was from Biochain. Primary antibodies were against MEGF10 (rabbit, Sigma HPA026876) and β‐ACTIN (rabbit, Abcam AB8227), followed by secondary HRP‐labelled anti‐rabbit (Sigma A6154). Chemiluminescence detection was with ECL+ (GE Healthcare, Little Chalfont, Buckinghamshire, UK) and X‐ray films were imaged on a flatbed scanner and analyzed using Image J (http://imagej.nih.gov/ij/).

## RESULTS

### Genome‐Wide DNA Methylation Analysis

To detect genome‐wide DNA methylation alterations in neuroblastoma, we used MCIP and promoter plus CpG island microarrays (Figure S1) 38, 40 to compare four neuroblastoma cell lines (Table S1) with cultured human neural crest cells (hNCC 37).

**Figure 1:**
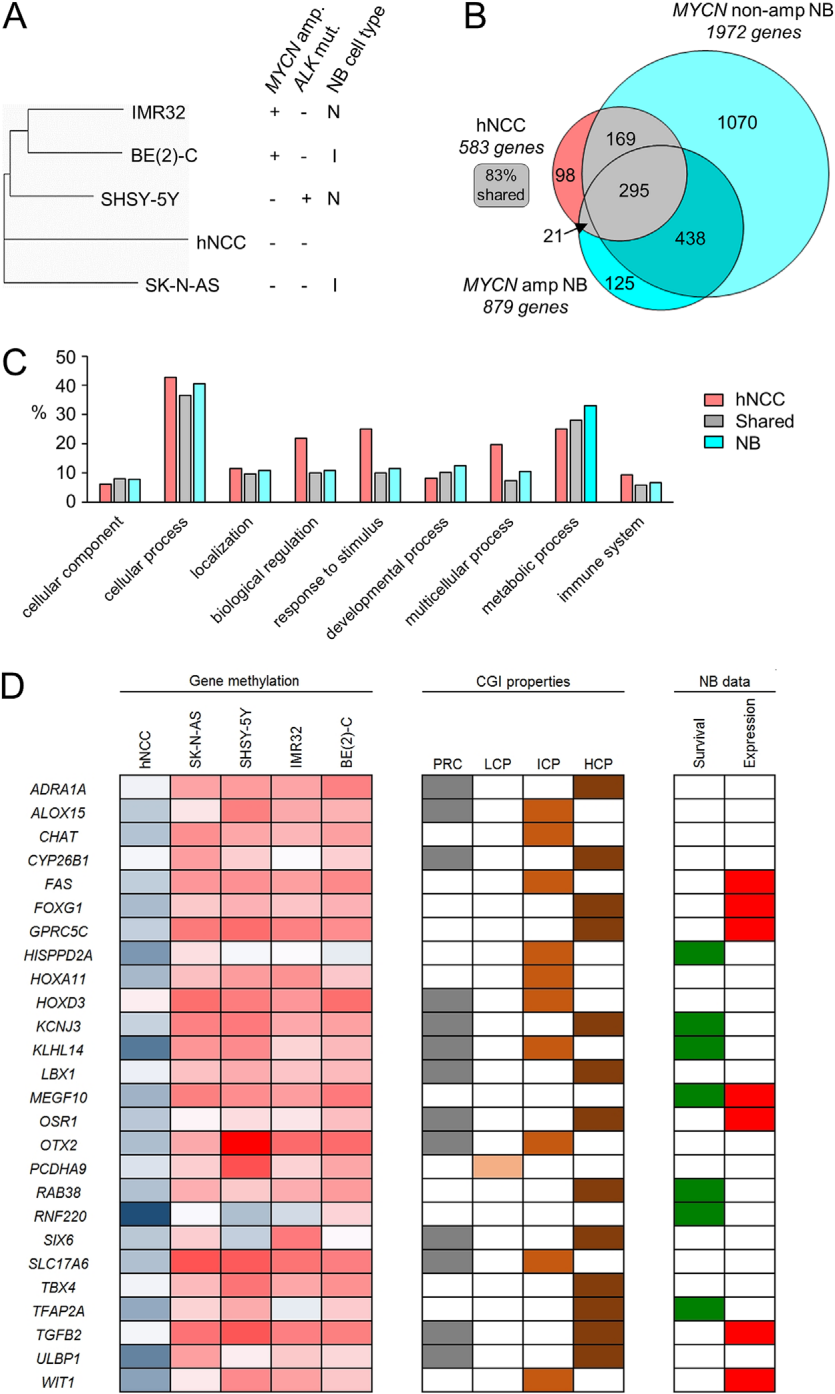
Genome‐wide DNA methylation analysis of neuroblastoma. (A) Dendrogram using Pearson's correlation coefficient, analysing all probe ratios from the MCIP/microarray analysis of human neural crest cells (hNCC) and four neuroblastoma cell lines (SK‐N‐AS, SHSY‐5Y, IMR32, and BE(2)‐C). *MYCN* amplification, *ALK* mutation, and neuroblastoma cell type are indicated next to the cell line names. (B) Venn diagram of overlap between lists of methylated genes having a probe ratio of greater than log_2_ 0.5 (Table S3) found in hNCC, MYCN‐amplified, and non‐amplified cell lines. (C) Gene ontology profiles of the hNCC‐unique methylated genes (hNCC), genes shared between hNCC and neuroblastoma cell lines (shared), and cell line‐unique genes (NB). Full results shown in Table S5. (D) Genes identified by MCIP as hypermethylated in four neuroblastoma cell lines compared to hNCC. The first five columns (“Gene methylation”) are a heatmap of gene methylation values (blue = low, red = high). CGI properties: PRC shows genes that are polycomb marked in ES cells, HCP, ICP, and LCP define which promoters have high, intermediate, or low CpG content. For quantitative DNA methylation results and further explanation of PRC, HCP, ICP, and LCP, see Table S6. NB data: “Survival” shows genes whose decreased expression is significantly associated with reduced relapse‐free survival in neuroblastoma (*P* < 0.05, log rank test); data generated in R2 using GSE16476; and “Expression” shows genes whose RNA expression is decreased in both neuroblastoma cell lines (GSE28019) and neuroblastoma tumors (GSE16476) compared to neural crest cells (GSE14340); comparison was made using the “Megasampler” function in R2 Genomics Analysis and Visualization Platform (http://r2.amc.nl). See Table S8 for full results.

The primary data set of all probe ratios from the microarray (see dendrogram in Figure 1A) suggested that hNCC were distinct from all other cells but most closely related to the I‐type SK‐N‐AS cell line. The three cell lines carrying oncogene mutations clustered together, with the *MYCN* amplified BE(2)‐C and IMR32 being most closely related, while SHSY‐5Y, the only cell line carrying an *ALK* mutation 41, was less related to the other two (Figure 1A).

Analysis of the DNA methylated genes detected by MCIP (Table S3) showed both *MYCN*‐amplified and non‐amplified cell lines had more methylated genes than hNCC (Figure 1B). However, 83% of the genes that were methylated in hNCC were also methylated in the neuroblastoma cell lines and these genes showed a similar gene ontology profile to other genes that were methylated in the neuroblastoma cell lines, but distinct from the pattern in hNCC (Figure 1C, Table S5). This suggests that while part of the neuroblastoma epigenome bears close similarity to hNCC, presumably reflecting common developmental origins, there are also a distinct set of pathogenic epigenetic changes in neuroblastoma.

### Identification of Differentially Methylated Genes

We used windowed T‐tests to identify genes that showed significant hypermethylation or hypomethylation in neuroblastoma cell lines compared to hNCC (Figure S1, Table S6). About 93 genes were significantly differently methylated between all four neuroblastoma cell lines and hNCC, of which 26 (28%) were hypermethylated and 67 (72%) were hypomethylated (Table S6). The hypermethylated genes were enriched in high‐CpG and intermediate‐CpG promoters (Table S6; Figure 1D), whereas the hypomethylated genes were enriched in low‐CpG promoters (LCP; Table S6) 42.

Using publicly available microarray data (GSE19274) we found no direct correlation between gene expression and DNA methylation; however, across the four cell lines, the most highly expressed genes were mostly hypomethylated; 96% of the genes in the top 90% rank of expression were hypomethylated (Figure S3; *P* = 0.002, Fisher exact test).

The chromosomal localization of the differentially methylated genes showed no obvious clusters of hyper‐ or hypomethylated genes (Figure S4).

The hypermethylated genes were significantly enriched in genes involved in transcription and in developmental processes, whereas the hypomethylated genes were enriched for sensory perception, signaling, and multicellular organism processes (Table S7).

In agreement with previous reports 43, 44, 45, we found that the hypermethylated genes were often polycomb repressive complex (PRC) marked 46, when compared to the hypomethylated genes (Figure 1D and Table S6; *P* = 1.37 × 10^−6^; Fisher exact test).

Thus, MCIP identified a set of genes that were differentially methylated between hNCC and neuroblastoma, which are excellent candidates for genes that play a significant role in neuroblastoma pathogenesis via epigenetic deregulation.

### Selection of Candidate Tumor Suppressor Genes

Initially, we concentrated on hypermethylated genes, to identify novel epigenetically repressed tumor suppressor genes. We used publicly available data to screen all 26 hypermethylated genes for their possible involvement in neuroblastoma pathogenesis by examining (i) the association between RNA expression and relapse‐free survival; and (ii) RNA expression in neuroblastoma tumors and cell lines compared to neural crest (Figure 1D, Table S8). Seven genes showed poorer survival in low‐expressing tumors and seven showed decreased RNA expression both in cell lines and tumors compared to neural crest; however, only one gene, *MEFGF10*, had an effect on survival as well as decreased expression in neuroblastoma (Figure 1D). We confirmed the consistent silencing of *MEGF1*0 in all four neuroblastoma cell lines by end‐point PCR (Figure S5). We therefore went on to examine *MEGF10* expression in tumors and its potential biological function in neuroblastoma.

### Expression and Biological Function of MEGF10

**Figure 2:**
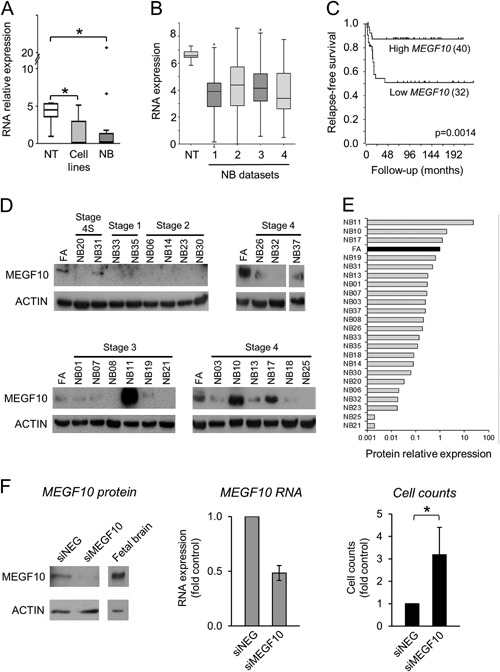
MEGF10 expression and biological function. (A) *MEGF10* RNA expression levels, investigated by QPCR in normal tissues (NT, *n* = 5), neuroblastoma cell lines (*n* = 9), and neuroblastoma tumor tissue (NB, *n* = 25), normalized to the endogenous levels of *TBP* and expressed relative to universal RNA (*P < 0.05, Mann–Whitney test). Full results are shown in Figure S6. (B) *MEGF10* RNA expression in normal adrenal (GSE3526, GSE7307, GSE8514) and four different sets of neuroblastoma tumor tissue (GSE16476, GSE12460, GSE16237, GSE13136), as measured using Affymetrix U133 microarrays (data from R2). Neuroblastomas showed significantly lower expression compared to normal adrenal (one‐way ANOVA *P* = 8.9 × 10^−7^). (C) Relapse‐free survival curve taken from dataset GSE16476 for patients with tumors lacking *MYCN* amplification (data from R2). Low‐expressing tumors showed decreased relapse‐free survival compared to high‐expressing tumors (*P* = 0.0014). (D) MEGF10 protein levels assayed by Western blot in fetal adrenal (FA) and 23 neuroblastomas (NB) with ACTIN as loading control. (E) Bar chart of MEGF10 protein levels relative to ACTIN, expressed as a ratio of the level in fetal adrenal (FA). (F) Growth of GIMEN neuroblastoma cells 72 h after transfection with MEGF10 siRNAs (siMEGF10) or non‐targeting pool (siNEG). Left‐hand panel: Western Blot of MEGF10 protein expression (representative of three experiments). Fetal brain is shown as a control tissue expressing high levels of MEGF10. Middle panel: *MEGF10* RNA expression assayed by QPCR expressed relative to siNEG controls (mean ± SEM of three experiments). Right‐hand panel: Attached cell counts expressed relative to siNEG controls (mean ± SEM of three experiments, *P < 0.05, paired t test).

QPCR analysis of neuroblastoma tumors and cell lines showed significantly lower *MEGF10* RNA expression than in normal tissues (Figures 2A and S6). Our RNA expression results were replicated in publicly available expression microarray datasets (Figure 2B) and importantly, these microarray data demonstrated a significant association between low‐*MEGF10* expression and reduced relapse‐free survival in neuroblastoma patients, especially in *MYCN* non‐amplified tumors (Figure 2C).

Western blotting analysis of MEGF10 protein expression (Figure 2D) showed that 87% (20 out of 23) of neuroblastomas had lower MEGF10 protein levels than that found in fetal adrenal (Figure 2E), in agreement with the RNA expression data (Figure 2A). This suggests that MEGF10 expression is primarily controlled at the transcriptional level.

MEGF10 knockdown effectively reduced RNA and protein expression in the highly expressing neuroblastoma cell line GIMEN and caused a reproducible three‐ to fourfold increase in cell numbers at 72 h (Figure 2F). This result was replicated in a second cell line, BCH‐N‐DW (Figure S7).

These results suggest that *MEGF10* may have an important role in regulating neuroblastoma growth, although further in vitro and in vivo experiments are required to understand the significant of these preliminary functional analyses. The consistent transcriptional down‐regulation of *MEGF10* expression in both neuroblastoma tumors and cell lines led us to carry out a detailed analysis of the epigenetic regulation of *MEGF10* in neuroblastoma.

### DNA Methylation of MEGF10 in Neuroblastoma

**Figure 3:**
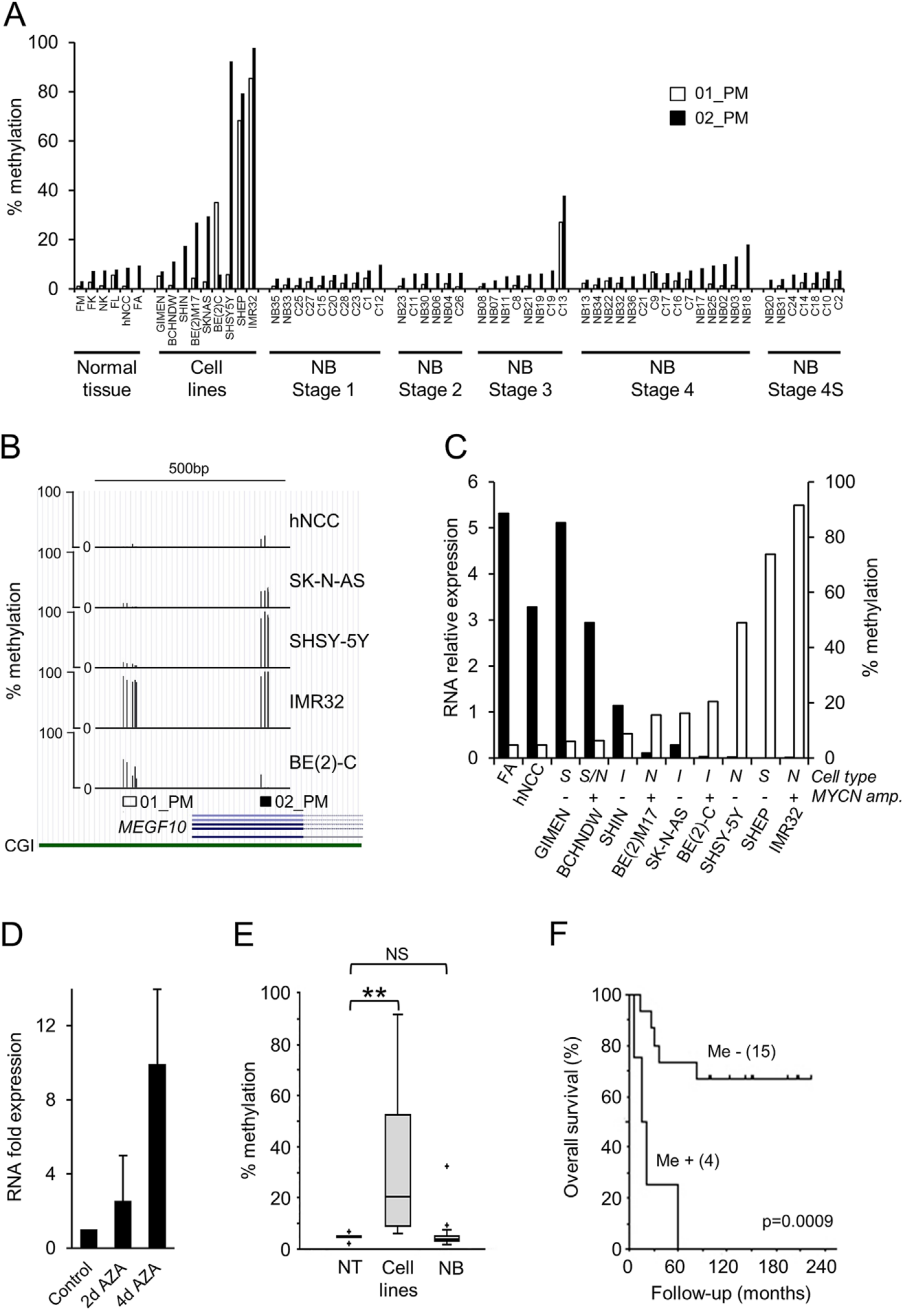
MEGF10 DNA methylation in neuroblastoma. (A) MEGF10 methylation assayed by pyrosequencing assays 01_PM (unfilled bars) and 02_PM (black bars) in normal tissue (fetal muscle [FM], fetal kidney [FK], normal kidney [NK], fetal lung [FL], human neural crest cells [hNCC], fetal adrenal [FA]), neuroblastoma cell lines and neuroblastoma tumors, grouped by stage. (B) *MEGF10* DNA methylation assayed by pyrosequencing assays 01_PM and 02_PM in hNCC and the four neuroblastoma cells lines used for MCIP. Bars show the percentage methylation at each CpG in the two pyrosequencing assays, positioned relative to the *MEGF10* first exon and CpG island (CGI), using the UCSC genome browser (http://genome.ucsc.edu). (C) *MEGF10* RNA expression assayed by QPCR (black bars) and DNA methylation levels detected by pyrosequencing (unfilled bars), in control tissues and neuroblastoma cell lines. RNA levels were normalized to the endogenous levels of *TBP* and expressed relative to universal RNA (full data in Figure S6). DNA methylation was calculated as the average of the 01_PM and 02_PM pyrosequencing assays (A). Neuroblastoma subtypes (I, N, or S) and *MYCN* amplification status are shown above the cell line names. (D) *MEGF10* RNA expression in SHSY‐5Y cells treated with 2 μM azadC (AZA) for 2–4 d. RNA levels were normalized to the endogenous levels of *TBP* and expressed as fold expression relative to levels in controls (solvent‐treated). (E) BoxPlot of *MEGF10* DNA methylation measured by pyrosequencing in normal tissues (NT, *n* = 6), neuroblastoma cell lines (*n* = 9), and neuroblastoma tumor tissue (NB, *n* = 46), using the average of assays 01_PM and 02_PM; full results are shown in (A) (**P < 0.005, NS; not significant, Mann–Whitney test). (F) Kaplan–Meier overall survival curve taken from dataset of NB patients in (A) for whom survival data were available. Me −, tumors with no DNA methylation compared to fetal adrenal and neural crest; Me +, tumors with increased DNA methylation compared to fetal adrenal and neural crest (using the average of assays 01_PM and 02_PM). Me + tumors showed decreased overall survival compared to Me−, tumors (*P* = 0.0009; log rank test).

We examined DNA methylation by pyrosequencing at two sites in the *MEGF10* CpG island in 6 normal tissues, 9 neuroblastoma cell lines, and 46 neuroblastoma tumor samples (Figure 3A and B). The pyrosequencing assays overlapped with the region identified as methylated in the *MEGF10* gene by our MCIP/microarray analysis (Figure S2C).

In the neuroblastoma cell lines there was no apparent relationship between the cell types (I, N, S) and their *MEGF10* expression or methylation, nor between *MYCN* amplification and *MEGF10* DNA methylation or expression (Figure 3C). In two inducible *MYCN* expression systems we found no reproducible change in DNA methylation when *MYCN* expression was altered (Figure S8), in agreement with our previous findings 47.

Of the nine neuroblastoma cell lines, seven expressed reduced levels of *MEGF10* RNA compared to normal tissues (fetal adrenal and hNCC) and most had some degree of DNA hypermethylation, while two cell lines (GIMEN and BCH‐N‐DW), had *MEGF10* RNA expression levels comparable to normal tissues and these two cell lines were unmethylated (Figure 3C). Thus DNA hypermethylation was common in neuroblastoma cell lines (Figure 3E) and there was an inverse relationship between *MEGF10* expression and DNA methylation (Figure 3C; *r* ^2^ = 0.914), suggesting a possible mechanistic role for DNA methylation in regulating *MEGF10* expression. This was confirmed by treating the hypermethylated line SHSY‐5Y with the demethylating agent 5‐aza‐2′‐deoxycytidine (azadC), which increased *MEGF10* RNA expression 10‐fold (Figure 3D).

**Figure 4:**
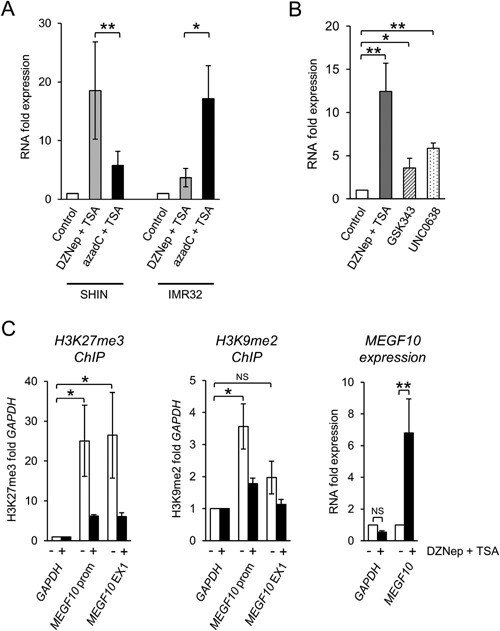
Reactivation of MEGF10 expression and ChIP. (A) QPCR of *MEGF10* expression in SHIN and IMR32 cells treated with 0.5 μM DZNep plus 0.1 μM TSA (gray bars) or 2 μM azadC plus 0.1 μM TSA (black bars) for 6 d. The control treatment was solvent (DMSO) (unfilled bars). RNA expression levels were normalized to the endogenous levels of *TBP* and expressed relative to the untreated controls (mean ± SEM of four experiments, *P < 0.05, **P < 0.005, paired t test). (B) QPCR of *MEGF10* expression in SHIN cells treated with 5 μM DZNep plus 0.1 μM TSA (gray bar), 10 μM GSK343 plus 0.1 μM TSA (hatched bar) or 0.1 μM UNC0638 plus 0.1μM TSA (dotted bar) for 24 h. The control treatment was solvent (DMSO) (unfilled bar). RNA expression levels were normalized to the endogenous levels of *TBP* and expressed relative to the untreated controls (mean ± SEM of four experiments, *P < 0.05, **P < 0.005, paired t test). (C) Left‐hand panel: H3K27me3 at the *MEGF10* promoter (prom) or *MEGF10* exon 1 (EX1), expressed as fold enrichment compared to *GAPDH*, in control and DZNep plus TSA‐treated SHIN cells (mean ± SEM of three experiments, *P < 0.05, paired t test). Middle panel: H3K9me2 at the *MEGF10* promoter (prom) or *MEGF10* exon 1 (EX1), expressed as fold enrichment compared to GAPDH, in control and DZNep plus TSA‐treated SHIN cells (mean ± SEM of three experiments, *P < 0.05, NS, not significant; paired t test). Right‐hand panel: RNA expression of *GAPDH* and *MEGF10* in SHIN cells treated with 5 μM DZNep plus 0.1 μM TSA for 24 h. Results shown relative to untreated controls (mean ± SEM of six experiments, **P < 0.005, NS, not significant; paired t test).

We then investigated DNA methylation of *MEGF10* in neuroblastoma tumor samples and found that only 4 of 46 (9%) had DNA methylation levels higher than normal tissues (Figure 3A and E). Interestingly, one of these four tumors was stage 3 and three were stage 4, suggesting that hypermethylation was associated with more aggressive tumors. All four hypermethylated tumors showed decreased overall survival compared to the hypomethylated tumors (Figure 4F). We found no hypermethylation of *MEGF10* in other childhood cancers, including aggressive cancers such as rhabdoid tumors (Figure S9), suggesting that *MEGF10* hypermethylation is restricted to some poor prognosis neuroblastomas.

We have demonstrated that *MEGF10* expression is consistently down‐regulated in neuroblastoma (Figure 2A and B), and that DNA hypermethylation appears to be prevalent in neuroblastoma cell lines but confined to a small subset of aggressive neuroblastoma primary tumor samples (Figure 3A and E). We therefore investigated other plausible epigenetic modifications that might repress *MEGF10* in neuroblastoma.

### MEGF10 Is Also Silenced by Repressive Histone Modifications

In order to identify other possible epigenetic mechanisms that might explain the transcriptional repression of *MEGF10* in the majority of neuroblastomas, which are not DNA methylated, we examined repressive histone modifications using pharmacological inhibitors and ChIP.

In the cell line that had repressed *MEGF10* expression with the lowest DNA methylation (SHIN; Figure 3C), *MEGF10* expression was poorly stimulated by the DNA methylation inhibitor 5‐aza‐2′‐deoxycytidine plus trichostatin A (azadC + TSA; Figure 4A). However, 3‐deazaneplanocin A, an agent that inhibits repressive histone methylation marks such as H3K27me3 and H3K9me2 48, plus TSA (DZNep + TSA), caused a substantial increase in *MEGF10* expression (Figure 4A). In contrast, in the most highly DNA methylated cell line (IMR32; Figure 3C), *MEGF10* expression was reactivated by azadC + TSA; but not by DZNep + TSA (Figure 4A). To verify the possible involvement of H3K27me3 and/or H3K9me2, we treated SHIN cells with the specific EZH2 inhibitor GSK343 49, or with the specific G9a and GLP inhibitor UNC0638, both of which also caused reactivation of *MEGF10* expression (Figure 4B). These results implicated the repressive histone modifications H3K27me3 and H3K9me2 in the epigenetic silencing of *MEGF10*.

We therefore went on to use chromatin immunoprecipitation (ChIP) to directly test for H3K27me3 and H3K9me2 at *MEGF10* in SHIN cells. At both locations tested, *MEGF10* had higher levels of H3K27me3 than at *GAPDH*, a constitutively expressed housekeeping gene (Figure 4C, left). There was also increased H3K9me2 at the *MEGF10* promoter region (Figure 4C, middle). This pattern of a wide distribution of H3K27 marks but with H3K9 marks concentrated around the promoter region, agrees with the general pattern of histone methylations observed for silenced genes in the human genome 50. Treatment with DZNep + TSA increased expression of *MEGF10* but not *GAPDH* (Figure 4C, right) and at the same time decreased H3K27me3 and H3K9me2 at *MEGF10* (Figure 4C, left and middle).

Thus, even in a cell line where *MEGF10* methylation was low, *MEGF10* expression was epigenetically silenced but by repressive histone methylation, suggesting that in non DNA‐methylated neuroblastomas, *MEGF10* may still be epigenetically repressed.

## DISCUSSION

This epigenomic study detected neuroblastoma‐specific DNA methylation changes by taking the novel approach of comparing malignant neuroblastoma cell lines with cultured normal neural crest cells (hNCC), their putative precursors 1, 2 (Figure S1). Many neuroblastomas are thought to develop from the sympathoadrenal lineage, which contributes to the sympathetic ganglia and medullary region of the adrenal gland. However, neuroblastomas can occur at any position along the sympathetic axis, suggesting that they can also develop from earlier neural crest derivatives 51. Thus, we have used neural crest cells in our experiments, as these probably represent the earliest available common precursor for all neuroblastomas.

### Overview of DNA Methylation Changes

The methylation levels of all probes from the array (Figure 1A), together with the annotated genes identified as methylated (Figure 1B and C), clearly showed that neural crest cells and neuroblastoma cells were related, reflecting their common developmental origins 1. However, there were a large number of neuroblastoma‐specific methylated genes (Figure 1B), demonstrating that deregulated epigenetic modifications play an important role in neuroblastoma pathogenesis.

Of the differentially methylated genes, 72% underwent hypomethylation and 28% showed increased methylation levels compared to hNCC (Table S6). In *MYCN* amplified BE(2)‐C and IMR32 cells, the transcriptional activation power of MYCN, together with induced chromatin changes leading to higher gene activation levels 52, 53, could explain the increased hypomethylation, although another mechanism must be involved in the *MYCN* non‐amplified cell lines. Two recent genome‐wide DNA methylation studies also reported a preponderance of DNA hypomethylation in neuroblastoma 32, 33, suggesting that epigenetic gene activation may be more common than repression in neuroblastoma. In this article, we concentrated on hypermethylated genes, in order to identify epigenetically repressed tumor suppressors.

### Characterization of the Hypermethylated Genes in Neuroblastoma Cell Lines

Our methylation results are not directly comparable with other studies, because we have made the novel comparison of cultured neural crest cells with neuroblastoma cell lines, whereas most other studies have made comparisons between different neuroblastoma subtypes (e.g., low‐ vs. high‐risk). However, some of the hypermethylated genes that we have identified have also been reported in other genome‐wide studies of DNA methylation in neuroblastoma; specifically *ADRA1A* 33, *CHAT* 33, *FAS* 31, *HOXD3* 29, 32, 33, *RAB38* 33, *RNF220* 33, *SLC17A6* 33, and *TBX4* 33, 54. In addition, others have found hypermethylation at the *HOXA* cluster 28 and at the *PCDH* clusters 31, 32, 33, 34, 55, where we found *HOXA11* and *PCDHA9* hypermethylated (Figure 1D).

The shared hypermethylated genes in the four neuroblastoma cell lines were enriched in high‐CpG promoters and PRC‐marked genes (Figure 1D), as expected from previous reports 42, 43, 44, 45. This suggests that many of the hypermethylated genes are normally unmethylated but PRC‐marked in stem cells and undergo “instructive” methylation 56 to irreversibly repress genes that drive differentiation during development.

Gene ontology analysis showed that the hypermethylated hits that we detected were enriched in genes involved in developmental processes (Table S7), which are presumably targets that need to be silenced in neuroblastoma in order to facilitate the developmental arrest that is thought to be the primary cause of childhood cancers 57.

We carried out the genome‐wide DNA methylation analysis using neuroblastoma cell lines, which we compared to cultured neural crest cells, so that we compared cultured cells in all cases, rather than cultured cells versus primary tissue, to reduce differences in DNA methylation caused solely by cell culture 58. In addition, we used publicly available expression data from primary tumors and associated patient survival data to filter the DNA hypermethylated genes, in order to identify candidate genes for further study that had in vivo relevance. *MEGF10* satisfied all our expression and survival criteria (Figure 1D), making it the obvious candidate for detailed analysis.

### Epigenetic Deregulation of MEGF10 in Neuroblastoma

All neuroblastoma cell lines, except the S‐type GIMEN and BCH‐N‐DW, had some degree of DNA hypermethylation of *MEGF10* and very low or absent expression (Figure 3C). There was an inverse relationship between *MEGF10* DNA methylation and expression (Figure 3C) and *MEGF10* could be reactivated by treating hypermethylated cells with the DNA demethylating agent aza‐deoxycytidine (Figures 3D and 4A), suggesting that DNA methylation plays a mechanistic role in the control of *MEGF10* expression in the majority of neuroblastoma cell lines. In the SHIN cell line, where *MEGF10* expression was repressed but there was little DNA methylation (Figure 3C), we demonstrated that *MEGF10* was transcriptionally silenced by repressive histone modifications (Figure 4).

*MEGF10* expression was consistently down‐regulated in neuroblastoma tumor tissue compared to normal tissue, as shown by our own results and by those in publicly available datasets (Figure 2A and B). Thus our genome‐wide epigenetic analysis, based on studying cultured cells, has correctly identified *MEGF10* as a repressed gene with direct relevance to neuroblastoma in vivo. Genome‐wide mutation analyses of neuroblastomas have not identified genetic abnormalities in *MEGF10* 4, 5, 6 and there was only infrequent (4/46; 7%) hypermethylation of *MEGF10* in tumor tissue (Figure 3A and E). It therefore seems likely that *MEGF10* expression is silenced in most neuroblastomas by epigenetic mechanisms other than DNA methylation, such as repressive histone modifications H3K27me3 and/or H3K9me2, as we have shown in the SHIN cell line (Figure 4), and as has been reported for other neuroblastoma tumor suppressor genes 23, 24, 55.

Interestingly, analysis of publicly available DNA methylation data (GSE39626; 32) gave similar results to those reported in this paper, with 2/25 (9%) of neuroblastomas having *MEGF10* DNA hypermethylation. Both of those *MEGF10*‐hypermethylated tumors were high‐risk 32 and in our data all the hypermethylated tumors were stages 3 or 4, and showed decreased overall survival (Figure 3F), suggesting that *MEGF10* DNA hypermethylation occurs predominantly in an aggressive subset of neuroblastomas. Most neuroblastoma cell lines are derived from high‐risk tumors 59 and we found the majority of cell lines to be DNA hypermethylated at *MEGF10* (Fgure 3A–C). It therefore possible that many neuroblastoma cell lines derive from a subpopulation of cells within high‐risk tumors that is hypermethylated at *MEGF10*. Alternatively, DNA hypermethylation of *MEGF10* in neuroblastoma cell lines may represent a long‐term stable epigenetic mark that serves to “lock” the silenced state in a gene that is normally repressed in neuroblastoma tumors by histone modifications 60.

### Functional Consequences of MEGF10 Repression

*MEGF10* encodes a transmembrane protein with multiple epidermal growth factor‐like domains and was initially thought to be mainly involved in the engulfment of apoptotic cells 61, 62. *MEGF10* is strongly expressed in the neural tube during early development, then in the spinal cord, CNS and developing muscle, with inherited mutations causing infantile mypoathies 63. MEGF10 also mediates cell–cell adhesion 64 and regulates retinal neuron patterning via homophilic interactions 65, showing that the gene plays a role in cellular interactions as well as in apoptotic engulfment.

Our siRNA knock‐down experiments (Figures 2F and S7) demonstrated that reduced expression of MEGF10 led to increased proliferation, suggesting that MEGF10 plays a growth regulatory role in neuroblastoma, potentially acting as a tumor suppressor. Publicly available clinical data showed an association between reduced *MEGF10* expression and decreased relapse‐free survival in neuroblastoma (Figure 2C), implying that the in vitro growth regulatory effects may reflect clinically relevant biological behavior. This effect was most pronounced in *MYCN* non‐amplified tumors (Figure 2C), suggesting that in the context of *MYCN* amplification, reduced MEGF10 expression does not have a significant effect on neuroblastoma survival, presumably because the multiple biological pathways related to neuroblastoma aggressiveness that are targeted by MYCN predominate 66. Interestingly, altered expression of *MEGF10* has now been reported in several cancers 67, 68, 69, with prognostic significance in ovarian cancer 68 and glioblastoma 69.

MEGF10 is tyrosine phosphorylated and its phosphorylation can be modulated by FGF 70, suggesting a role in cellular signaling pathways. Functionally, MEGF10 is implicated in Schwann cell plasticity 71 and importantly, in the stemness of neuroblastoma cells 72. Our epigenetic studies have shown that *MEGF10* expression is frequently down‐regulated in neuroblastomas and that it modulates neuroblastoma growth properties. These findings suggest that *MEGF10* plays an important role in neuroblastoma biology and investigation of the mechanisms regulating *MEGF10* and its involvement in cellular signaling pathways may identify new therapeutic targets for neuroblastoma, as well as prognostic markers.

## Supporting information

Additional supporting information may be found in the online version of this article at the publisher's web‐site.

Supplementary figures

supplementary Table S1

supplementary Table S2

supplementary Table S3

supplementary Table S4

supplementary Table S5

supplementary Table S6

supplementary Table S7

supplementary Table S8
