# Supplementary material for: Genome‐wide DNA methylation analysis identifies MEGF10 as a novel epigenetically repressed candidate tumor suppressor gene in neuroblastoma
Source: Mol Carcinog. 2016 Nov 29;56(4):1290–301. doi: 10.1002/mc.22591 (PMC5396313; doi:10.1002/mc.22591)
Supplement: Supplementary file 1 — Supplementary figures [file MC-56-1290-s001.pdf]

Fig. S1

Gene discovery

MCIP

Microarrays	385,000 array probes
	28,266 CpG islands
	24,031 unique genes
Cell lines	Human neural crest cells
	4 neuroblastoma cell lines

Data analysis

Normalisation	Lowess normalisation
Create data groups	Normal tissue and neuroblastoma cell lines
T test for differentially methylated probes	p<0.01, window = 1000bp
Probe grouping	Within 700bp of transcriptional start site
Gene identification	Closest gene within 1400bp probe group

Candidate gene selection

DNA methylation, expression screening and bioinformatic analysis

RT-PCR and COBRA	Fetal adrenal and human neural crest cells
	4 neuroblastoma cell lines
Patient survival and RNA expression	GSE 14340, GSE16476, GSE28019

Validation and functional analysis

DNA methylation and expression analysis

QPCR, COBRA and pyrosequencing	Fetal adrenal and human neural crest cells
	8 neuroblastoma cell lines
	23 neuroblastomas

Functional analysis

siRNA knock-down	Neuroblastoma cell lines

Figure S1: Workflow
Summary of the workflow for gene discovery, candidate gene selection, validation and functional analysis.

Fig. S2

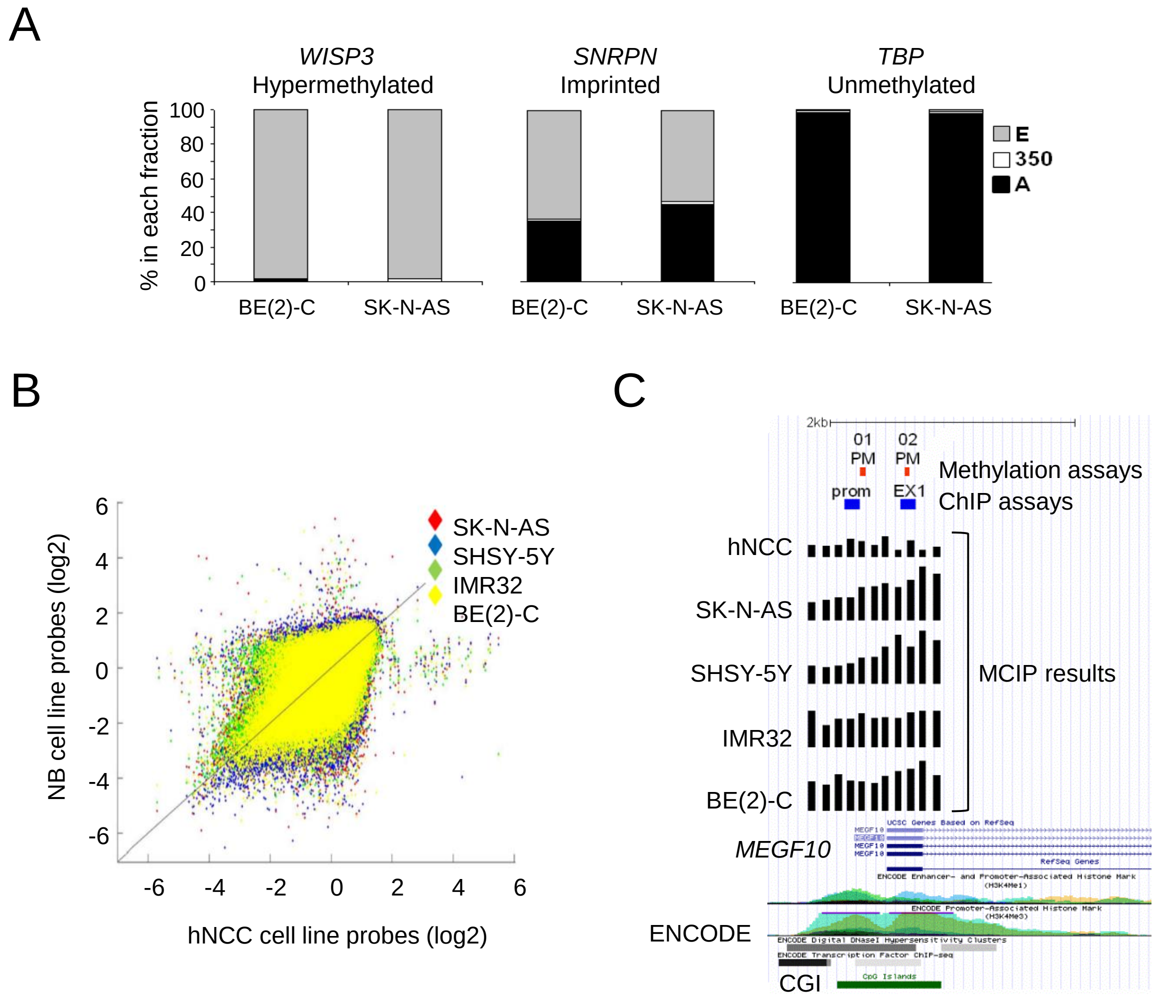

Figure S2: MCIP quality control and results

A: The different MCIP fractions; binding buffer (A), wash buffer (350) and elution buffer (E), were tested for their DNA content of three known marker genes in two of the five cell lines by QPCR. Almost 100% of the hypermethylated gene *WISP3* was recovered in the eluted, methylated-DNA containing fraction (E), while the imprinted *SNRPN* gene was found in both A and E fractions, as predicted for a 50% methylated gene. *TBP* was used as an unmethylated control gene and was only found in the unmethylated DNA fraction (A). Primers sequences are given in table S4.

B: Scatter plot of all probe ratios from the MCIP analysis (log_2) of the neuroblastoma cell lines against hNCC.

C: *MEGF10* methylation detected by MCIP. Black bars show the probe ratios derived from MCIP for hNCC and four neuroblastoma cell lines, positioned on the *MEGF10* promoter region, showing the transcripts (*MEGF10*), H3K4Me1 marks (ENCODE) and CpG island (CGI) (http://genome.ucsc.edu). The positions of the pyrosequencing assays (01_PM and 02_PM) and regions amplified by QPCR for ChIP assays are shown at the top.

Fig. S3

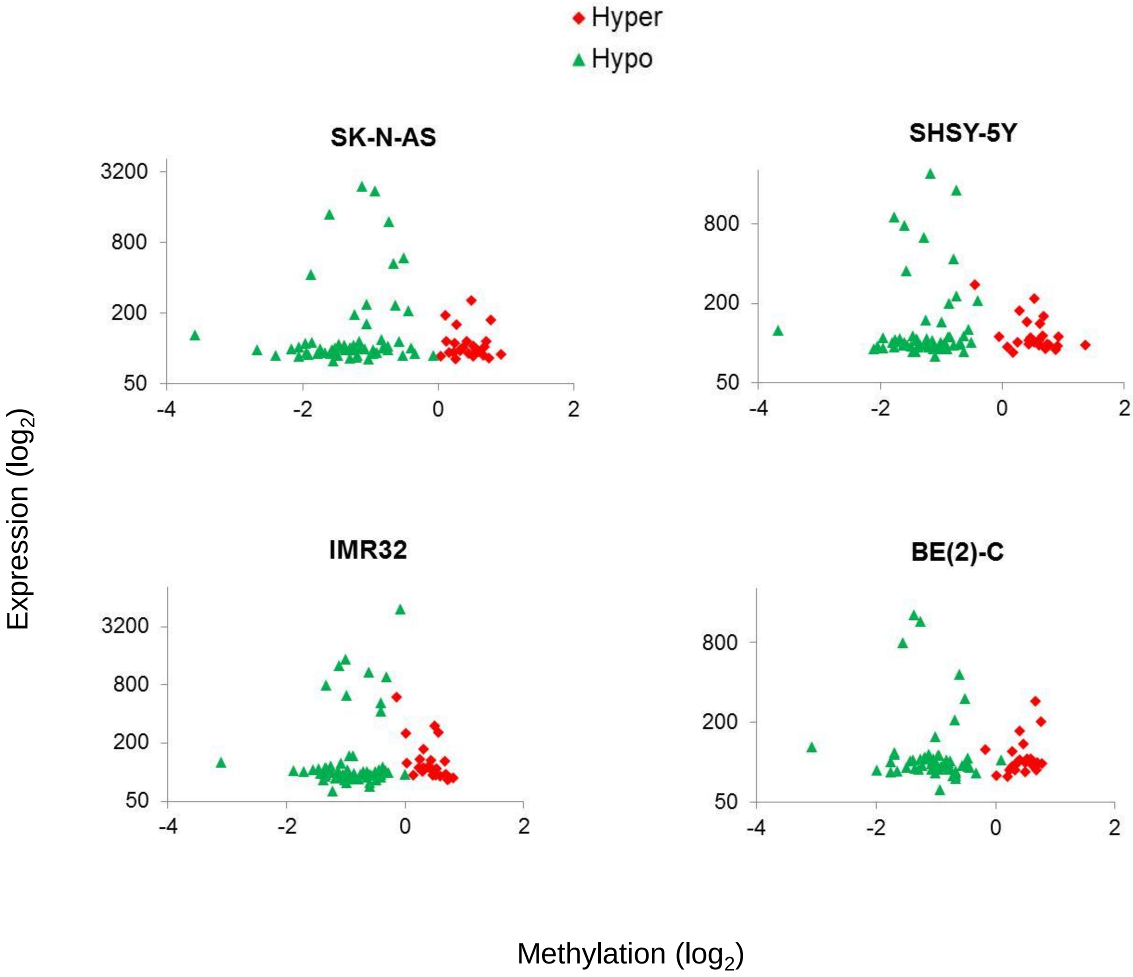

Figure S3: Expression levels of differentially methylated genes in neuroblastoma cell lines

Expression levels of genes that were found by MCIP to be hypermethylated (red diamonds) or hypomethylated (green triangles) in neuroblastoma cell lines compared to neural crest cells. Log_2 expression levels (from published microarray dataset GSE19274) were plotted against log_2 gene methylation levels (table S3). 96% of the genes in the top 90% rank of expression were in the hypomethylated group and only 4% were in the hypermethylated group (p=0.002, Fisher exact test).

Fig. S4

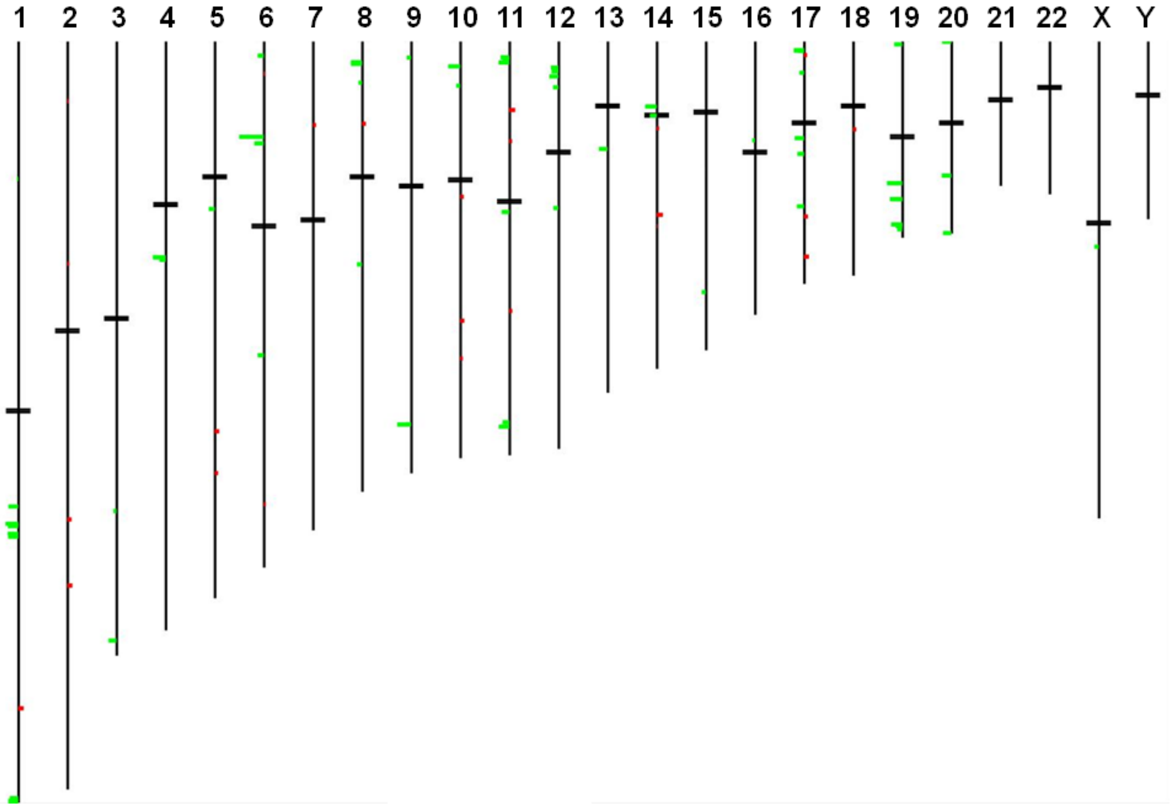

Figure S4: Chromosomal localisation of differentially methylated genes

Chromosomal locations of genes that were differentially methylated between neuroblastoma cell lines and neural crest cells. Green bars indicate hypomethylated genes and red bars hypermethylated genes (table S6). The length of the bar is a function of the log_2 value for gene methylation. Horizontal black bars indicate the centromere of the respective chromosome.

Data produced using “Caryoscope” software:

Awad, I. A., C. A. Rees, et al. (2004). "Caryoscope: an Open Source Java application for viewing microarray data in a genomic context." BMC Bioinformatics 5: 151.

Fig. S5

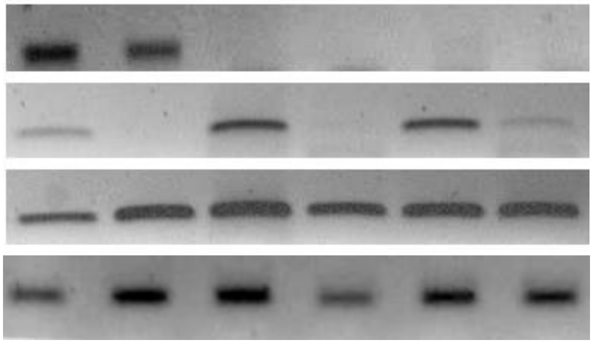

	FA	hNCC	IMR32	BE(2)-C	SK-N-AS	SHSY-5Y
MEGF10	+	+	-	-	-	-
TGFB2	+	+	+	+/-	-	+
KLHL14	+	-	+	-	+	-
ALOX15	+	-	+	-	+	+/-
ULBP1	-	+	+	-	+	-
HOXA11	+	+	+	+	-	-
HOXD3	+	+	+	+	+	+
FAS	+	+	+	+	+	+
TBX4	-	-	+	-	+	+
OTX2	-	-	-	-	-	-
FOXG1	-	-	-	-	-	-

Figure S5: Expression of hypermethylated genes

Selected hypermethylated genes were assayed for their expression by RT-PCR in foetal adrenal (FA), neural crest cells (hNCC) and the four neuroblastoma cell lines used for MCIP. Top panel shows representative gels of RT-PCRs. Table below summarises the results; + indicates strong expression, +/- indicates weak expression and – indicates no expression. *MEGF10* (shaded in grey) showed consistent down-regulated expression in all cell lines compared to normal tissues. Primer sequences are given in table S4.

Fig. S6

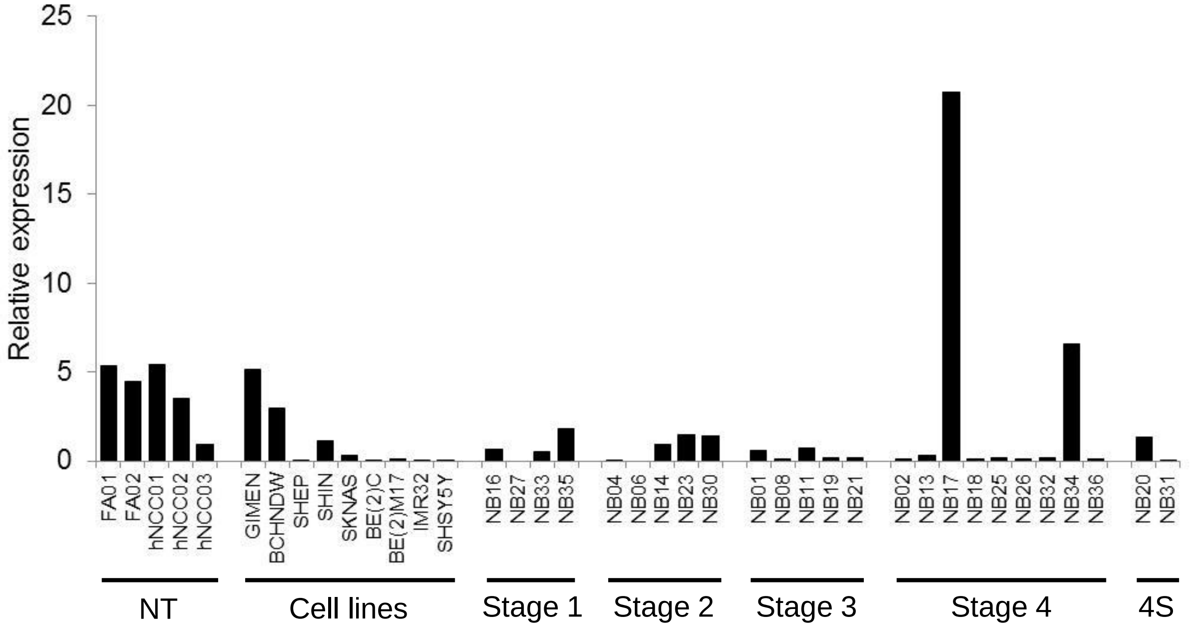

Figure S6: *MEGF10* RNA expression in neuroblastoma

MEGF10 RNA expression was assayed by QPCR using primers MEGF10 F and MEGF10 R (table S4) and cDNA from normal tissue (NT; fetal adrenal (FA) and neural crest cells (hNCC)), neuroblastoma cell lines and neuroblastoma tumours (classified by stage). *MEGF10* expression was normalised to *TBP* and expressed as fold values compared to expression in universal RNA.

Fig. S7

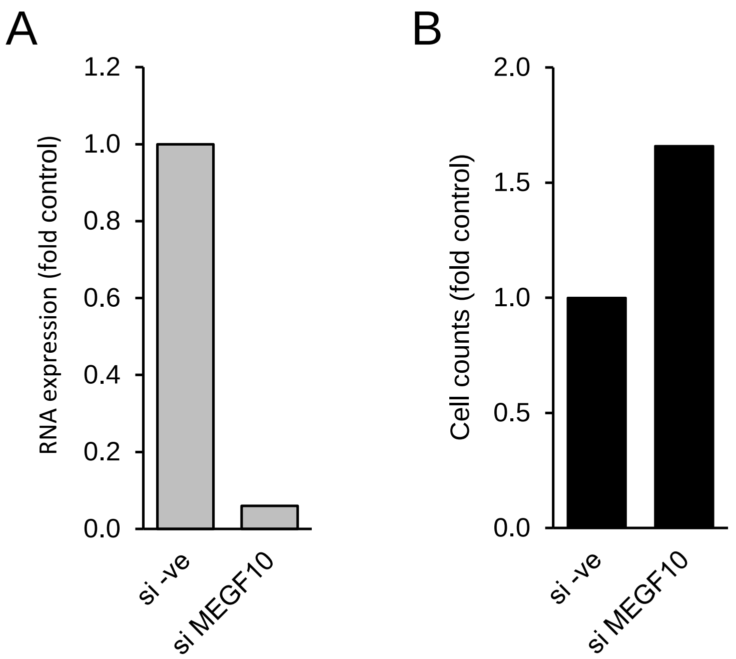

Figure S7: siRNA knockdown of *MEGF10* in BCH-N-DW cells

A: QPCR analysis of *MEGF10* RNA expression.

B: Cell count analysis.

Cells were harvested 72 hours after siRNA-mediated *MEGF10* knockdown.

Results of a single experiment.

Fig. S8

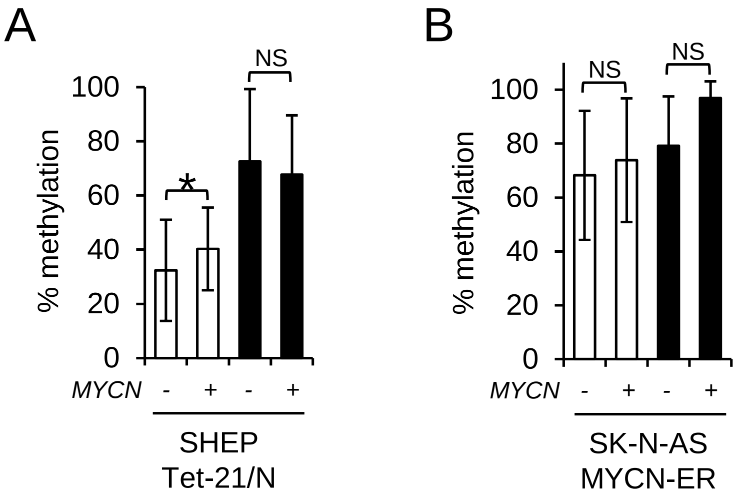

Figure S8: Effect of *MYCN* expression on *MEGF10* DNA methylation

MEGF10 DNA methylation assayed by pyrosequencing assays 01_PM (unfilled bars) and 02_PM (filled bars) in SHEP Tet-21/N cells (A) and SK-N-AS MYCN-ER cells (B) with MYCN expression uninduced (-) or induced (+). Results are mean ± SD of methylation at 5 CpGs (01_PM) or 4 CpGs (02_PM), *; $p<0.05$, NS; not significant, paired t test.

Fig. S9

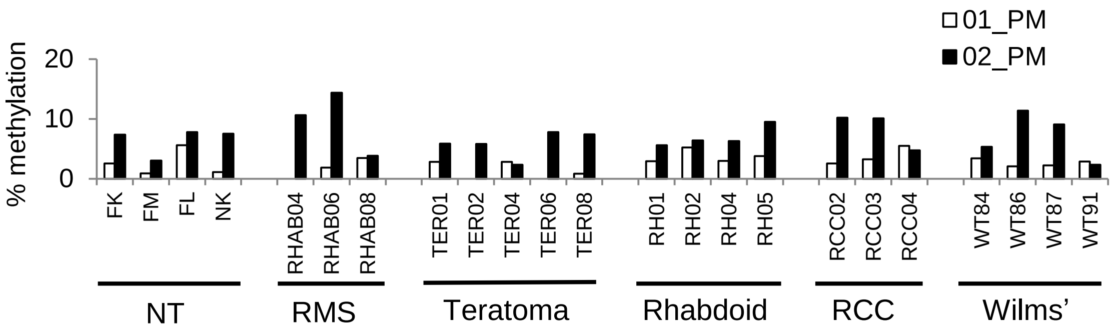

Figure S9: *MEGF10* DNA methylation in other childhood tumours

MEGF10 methylation assayed by pyrosequencing in normal tissue (NT; fetal kidney (FK), muscle (FM), lung (FL), normal kidney (NK)), three rhabdomyosarcomas (RMS), five teratomas, four rhabdoid tumours, three renal cell carcinomas (RCC) and four Wilms' tumours.

All samples were assayed using the pyrosequencing assays 01_PM (unfilled bars) and 02_PM (black bars); for assay details see figure 3B and "Materials and methods".
