# Supplementary material for: Genome‐wide DNA methylation analysis identifies MEGF10 as a novel epigenetically repressed candidate tumor suppressor gene in neuroblastoma
Source: Mol Carcinog. 2016 Nov 29;56(4):1290–301. doi: 10.1002/mc.22591 (PMC5396313; doi:10.1002/mc.22591)
Supplement: Supplementary file 2 — supplementary Table S1 [file MC-56-1290-s002.pdf]

Table S1: Neuroblastoma cell lines used in the study

N = neuronal subtype, S = substrate adherent subtype, I = intermediate subtype.

M = male, F = female, LN = lymph node, BM = bone marrow, ? = not known.

Data taken from Thiele, C. J. (1998) "Neuroblastoma cell lines" Journal of Human Cell Culture 1: 2

Cell line	Cell type	Parental cell line	Patient age (months)	Sex	Primary site	Metastatic site	Cell line origin	Treated
BE(2)-C	I	SK-N-BE(2)	26	M	?	BM	BM	+
BE(2)-M17	N							
BCH-N-DW	N & S	-	17	M	Abdomen	BM	BM	-
GI-ME-N	S	-	24	F	Adrenal	LN, BM	BM	+
IMR32	N	-	13	M	Abdomen	?	Abdomen	-
SH-EP	S	SK-N-SH	48	F	Thorax	BM	BM	+
SH-IN	I							
SH-SY5Y	N							
SK-N-AS	I	-	96	F	Adrenal	BM	BM	+

1-53, except for BCH-N-DW (personal communication, C. McConville).

MYCN amplification	Stage
+	4
+	4
-	4
+	?
-	4
-	4
