# Supplementary material for: Genome‐wide DNA methylation analysis identifies MEGF10 as a novel epigenetically repressed candidate tumor suppressor gene in neuroblastoma
Source: Mol Carcinog. 2016 Nov 29;56(4):1290–301. doi: 10.1002/mc.22591 (PMC5396313; doi:10.1002/mc.22591)
Supplement: Supplementary file 3 — supplementary Table S2 [file MC-56-1290-s003.pdf]

Table S2: Neuroblastoma tumours used in the study

? = not known, ND = not done.

Tumour code	Stage	Alive (A) Dead (D) Relapsed (R)	MYCN amplification
C01	1	A	-
C02	4S	A	-
C07	4	D	-
C08	3	A	ND
C09	4	D	-
C10	4S	A	-
C11	2	A	-
C12	1	A	-
C13	3	A	-
C14	4S	A	-
C15	1	A	-
C16	4	D	+
C17	4	D	-
C18	4S	A	+
C19	3	A	-
C20	1	A	-
C21	4	D	-
C23	1	A	-
C24	4S	A	-
C25	1	A	-
C26	2	A	-
C27	1	A	-
C28	1	A	-
NB01	3	A	-
NB02	4	D	+
NB03	4	D	ND
NB04	2	A	-
NB06	2	R, A	-
NB07	3	D	+
NB08	3	A	-
NB09	4	?	ND
NB10	4	D	+
NB11	3	?	-
NB13	4	A	+
NB14	2	R, A	-
NB15	4	?	-
NB17	4	D	-
NB18	4	D	+
NB19	3	A	-
NB20	4S	R, A	-
NB21	3	A	-
NB22	4	?	-

NB23	2	A	-
NB25	4	D	-
NB26	4	D	-
NB30	2	A	-
NB31	4S	A	-
NB32	4	D	-
NB33	1	R, A	-
NB34	4	A	-
NB35	1	A	ND
NB36	4	D	ND
