# Supplementary material for: Genome‐wide DNA methylation analysis identifies MEGF10 as a novel epigenetically repressed candidate tumor suppressor gene in neuroblastoma
Source: Mol Carcinog. 2016 Nov 29;56(4):1290–301. doi: 10.1002/mc.22591 (PMC5396313; doi:10.1002/mc.22591)
Supplement: Supplementary file 4 — supplementary Table S3 [file MC-56-1290-s004.pdf]

Table S3: Methylation levels of genes identified by MCIP

List of unique genes identified by MCIP using Nimblegen Human DNA Methylation 385K Promoter Plus CpG Island Arrays. Gene methylation levels derived from the mean probe ratios (MCIP/input) within 700bp of the transcriptional start site (log2).

	**Cell line**				
Gene	**hNCC**	**SK-N-AS**	**SHSY-5Y**	**IMR32**	**BE(2)-C**
	0.41312	0.74112	0.792785	0.4968	0.595045
	0.420195	-0.16773	0.685277	-0.53127	-0.03278
	0.42759	0.15915	0.408436	0.272519	0.436259
A26B1	0.572234	0.563665	0.618847	0.42301	0.506278
A26C2	0.672151	0.506679	0.855019	0.62291	0.732548
A26C3	0.416953	0.496753	0.562293	0.299781	0.465388
A2BP1	0.650582	0.222635	0.121292	0.758329	0.031315
A2M	-0.27548	-0.63201	0.258897	-0.53606	-0.44251
A2ML1	0.555416	0.823257	0.906686	0.303972	0.656212
A4GALT	0.006041	0.28154	0.114169	0.268191	-0.00024
A4GNT	-1.19187	-0.88997	-1.10882	-1.03132	-1.23562
AAAS	0.006458	-0.02549	-0.10636	-0.13099	0.795076
AACS	0.097985	-0.01899	0.117254	-0.07648	0.046637
AADAC	0.65494	0.498994	0.238155	0.474352	0.311579
AADACL1	-0.73032	-0.81272	-0.92405	-0.67351	-0.79666
AADACL2	-0.03186	-0.25204	-0.19361	-0.26835	-0.34061
AADACL4	0.295026	0.063706	0.050139	-0.06636	0.041245
AADAT	0.061008	-0.13437	0.179164	-0.03125	0.137163
AAK1 AC092431.7	-0.5147	-0.58561	-0.43197	-0.50107	-0.28678
AAMP PNKD	-0.4367	-0.52593	-0.72286	-0.38155	-0.68406
AANAT	0.37048	0.235709	0.426562	-0.14536	0.564627
AARS	0.159521	0.106217	0.042891	0.087721	0.124475
AARSD1 RUNDC1	-0.02105	-0.05994	-0.11008	-0.13849	-0.11652
AASDH	-0.12473	-0.52954	-0.55731	-0.23487	-0.78423
AASS	0.398289	0.140709	-0.48009	-0.50721	-0.14821
AATF	-0.45088	0.194905	-0.03591	-0.34445	0.050155
AATK	0.093116	0.242285	-0.01898	0.09698	0.302521
ABAT	-0.19959	-0.20579	-0.34886	-0.07467	-0.2969
ABCA1	-0.21021	0.299344	0.631354	0.444663	0.566161
ABCA10	-0.117	-0.31989	-0.2793	-0.30947	-0.32713
ABCA13	-0.14225	-1.29413	-1.55774	-0.66396	-0.99935
ABCA2 C9orf139	0.156464	0.073316	-0.00461	0.038339	0.015467
ABCA3	0.132436	0.28728	0.257754	0.242943	0.292246
ABCA4	0.471091	0.682164	0.817618	0.49828	0.62475
ABCA6	-0.47839	-0.49298	-0.97354	-0.98436	-1.00514
ABCA7	0.271938	-0.54393	0.005378	-0.32355	-0.23072
ABCA8	-0.2026	-0.16579	-0.74039	-0.68248	-0.64187
ABCA9	-0.17053	-0.37084	-0.79729	-0.58158	-0.55519
ABCB1	-0.04989	0.080945	0.051576	0.149636	0.101519
ABCB1 RUNDC3B	-0.21599	-0.25525	-0.25517	-0.32456	-0.35209
ABCB10	0.054642	0.103186	0.248087	0.054052	0.230824

ABCB11	-0.27502	-0.49354	-0.78786	-0.7058	-0.78968
ABCB5	-0.17053	-0.4157	-0.48758	-0.31907	-0.3953
ABCB6 ATG9A	0.012627	0.262333	-0.0028	-0.03469	0.151768
ABCB7	0.284833	0.103869	0.248299	-0.37799	-0.58758
ABCB8	-0.05108	-0.013	-0.15718	0.156211	-0.11532
ABCB9	0.132947	-0.05466	0.146678	-0.00043	0.003868
ABCB9 OGFOD2	-0.30742	-0.41919	-0.48089	-0.35921	-0.37154
ABCC1	0.289557	0.349508	0.409979	0.166862	0.246006
ABCC10	0.561428	0.6947	0.702658	0.434868	0.214493
ABCC12	0.14345	0.036192	0.20396	0.070386	-0.73315
ABCC2	0.410376	0.565939	0.55849	0.159809	0.320846
ABCC3	0.189095	0.385755	0.364857	0.341126	0.432248
ABCC4	-0.12828	0.010028	-0.30954	-0.28762	-0.19135
ABCC5	0.190332	0.099324	0.022869	0.253922	0.057691
ABCC6	0.411418	0.688163	0.466354	0.226992	0.405493
ABCC8	-0.00275	0.18509	0.071407	0.102727	0.11856
ABCC9	0.03604	0.460272	0.544045	-0.0407	0.211166
ABCD2	-0.58047	-1.06114	-1.34109	-0.95535	-1.39078
ABCD3	-0.12188	-0.20072	-0.21649	0.170583	0.165057
ABCD4	0.144161	0.161574	0.261913	0.170952	0.036675
ABCF1	-0.75629	-0.5926	-0.90988	-0.50048	-0.761
ABCF2	-0.36225	-0.26044	-0.62138	-0.31133	-0.37304
ABCF3	-0.11237	-0.03406	-0.30022	-0.15342	-0.41582
ABCG2	0.149507	0.22944	0.269893	0.069639	-0.09448
ABCG4	-0.25344	0.171257	-0.09714	-0.21534	0.136672
ABCG5 ABCG8	0.314957	0.392267	0.423698	0.198147	0.407566
ABHD1	0.312209	0.458981	0.361523	0.429287	0.423071
ABHD10	0.376513	0.657919	-0.03696	0.350138	0.43128
ABHD11	-0.06529	0.112268	-0.17366	-0.04664	-0.19695
ABHD12	0.057664	0.070385	0.072896	0.039475	0.046849
ABHD14B ABHD14A	0.305444	-0.05007	-0.27548	0.255055	0.032644
ABHD2	-0.40864	-0.3731	-0.41934	-0.03672	-0.13178
ABHD3	0.275347	0.372213	0.347112	0.260878	0.096273
ABHD4	-0.20378	-0.13926	-0.28011	-0.2519	-0.25231
ABHD5	0.15201	0.219542	0.260376	0.107693	0.204436
ABHD6	0.076205	0.180223	-0.03344	0.165401	0.123201
ABHD7	0.289102	0.41125	0.423338	0.320293	0.471754
ABHD8 MRPL34	0.034602	0.073135	0.038747	0.177999	0.228607
ABHD9	0.33974	0.404132	0.603137	0.334712	0.265625
ABI2	-0.23704	-0.2036	-0.30058	-0.25857	-0.22079
ABI3BP	0.114604	-0.08353	-0.27252	-0.22031	-0.09078
ABLIM2	0.049121	0.280307	0.193536	0.14293	0.298498
ABLIM3	0.347784	0.400337	0.566079	0.30409	0.371876
ABO	0.200535	0.112462	0.31969	0.21004	0.298383
ABP1	0.662701	0.781855	0.982228	0.391574	0.650384
ABRA	0.510846	0.787353	0.293541	0.51297	0.506647
ABT1	-0.18908	-0.11513	-0.37966	-0.26003	-0.31712
ABTB2	-0.15281	0.093634	-0.02734	0.108937	0.04175
AC001226.1 CLN5	0.321062	0.539129	0.50114	0.210532	0.371055
AC002055.2	-0.00766	-0.05334	0.082387	0.023299	-0.03613

AC002064.2	-0.13914	-0.21176	-0.65896	-0.02016	-0.5054
AC002347.3	0.520947	0.529373	0.777153	0.382244	0.355367
AC002472.8	-0.23823	-0.25504	-0.41513	-0.24377	-0.41918
AC002472.8 P2RX6	-0.31442	-0.31192	-0.52489	-0.30313	-0.42344
AC002550.1	0.389047	0.395864	0.382429	0.150689	0.374029
AC003042.2	0.056272	0.041272	-0.02633	0.120175	-0.10627
AC003662.3	0.405464	0.471309	0.617297	0.382894	0.534632
AC004014.2	0.002209	-0.06193	-0.05841	-0.29218	-0.19824
AC004053.1 SCYE1	-0.72518	-0.86683	-0.71342	-0.71174	-0.79091
AC004166.12 AC004166.12	-0.56183	-0.64166	-0.97801	-0.65188	-0.69831
AC004224.1	0.023618	0.10244	0.090084	0.17905	0.212209
AC004241.2	-0.11144	0.046542	-0.28361	-0.21474	-0.25385
AC004449.1 RNF126	0.319669	0.378336	0.446882	0.37961	0.374014
AC004466.1	0.068638	0.258859	0.34212	0.331682	0.295603
AC004691.1	0.226924	0.323364	0.41551	0.271035	0.436855
AC004754.1	-0.04532	-0.00783	-0.23068	-0.21456	-0.24315
AC004771.1 KIF1C	-0.0521	0.069746	0.009959	0.078895	0.075968
AC004797.1	0.411041	0.122891	-0.35368	-0.39261	0.443837
AC004836.2	0.281255	0.438788	0.524358	0.16342	0.409666
AC004877.1 AC093458.6	0.160646	0.179498	0.058179	0.085151	0.195601
AC004889.1	0.206739	0.494617	0.43402	0.250679	0.297204
AC004917.2	-0.11243	-0.19848	-0.25346	-0.02661	-0.35668
AC004951.5	0.513502	0.590715	0.47257	0.439635	0.369523
AC004953.2	0.509438	0.403759	0.457002	0.2684	0.298952
AC004982.3	0.144256	0.335966	0.416046	0.179382	0.127308
AC004985.2	0.038683	-0.03103	-0.21372	0.014	0.142671
AC004985.2 UBE2D4	0.27987	0.228855	0.525343	0.051707	0.252722
AC004997.2	-0.11983	0.200866	-0.22955	-0.10835	0.090319
AC005013.3	0.156685	-0.15853	0.055489	0.217273	0.073728
AC005020.5	-0.0307	-0.36811	-0.44211	-0.23848	-0.4009
AC005041.2	0.102302	0.152116	0.027784	-0.13317	0.091484
AC005077.5	0.471237	0.716593	0.707801	0.462666	0.546453
AC005082.3	0.044237	0.014332	-0.18597	0.039558	-0.09623
AC005253.1	0.131962	0.12965	0.162076	0.214633	-0.07103
AC005261.2	0.208039	0.104814	0.002571	-0.1662	0.03923
AC005277.1	-0.20109	-0.05974	0.039021	-0.14564	0.144383
AC005361.5	0.150264	-0.07817	0.357387	0.158809	-0.0122
AC005392.2	0.057822	-0.24769	-0.19856	0.092492	0.144207
AC005599.5	0.508139	-0.52527	1.28082	0.224834	-0.41937
AC005691.1 TMEM132E	-0.1672	-0.14668	-0.07551	0.125244	-0.195
AC005692.1	0.055029	-1.13261	-0.50998	-0.10263	0.634866
AC005726.6 KIAA0100	0.374766	0.377757	0.451413	0.252147	0.316657
AC005726.8	0.343479	0.366053	0.399722	-0.20532	0.441341
AC005921.3	0.158218	-0.00536	0.12984	-0.00756	0.000721
AC006014.3 TRIM73	0.697283	0.934056	0.788835	0.635367	0.714406
AC006017.2	0.046146	0.080501	-0.00801	0.168462	0.166217
AC006156.5	0.365579	0.077848	0.119089	0.311699	0.225865
AC006273.1	0.010983	0.012864	0.08004	-0.04819	-0.02335
AC006435.7	0.155489	0.380146	0.209872	0.164684	0.38444
AC006960.1	0.354781	0.339449	0.251565	0.280026	0.179948

AC006960.1 ANLN	0.166603	0.016097	-0.26976	0.078156	0.019785
AC007000.2 CCDC146	-0.67185	-0.66932	-1.07972	-0.58452	-0.94537
AC007001.2	0.185841	0.038708	0.041601	-0.41332	-0.101
AC007068.17	0.216908	0.258916	0.478164	0.099661	0.136861
AC007364.3	0.023794	0.352734	0.465732	-0.08059	0.026028
AC007389.7	-0.56009	-0.52865	-0.6299	-0.36894	-0.41497
AC007455.7	0.132917	-0.14441	-0.58331	-0.55359	-0.82106
AC007546.6 AC007546.6	0.147381	0.384189	0.096863	0.300692	0.361029
AC007562.6	0.156961	-0.03773	0.065274	-0.02562	0.061115
AC007610.10	0.03277	0.063918	0.1887	-0.04242	0.062758
AC007679.4	-0.39977	-0.25523	-0.29222	-0.27041	-0.38011
AC007920.18	0.509073	-0.38568	-0.13939	0.474486	0.073555
AC007964.3	0.473865	0.740942	0.779453	0.606746	0.617035
AC008012.8 RAD51AP1	-1.02089	-1.01796	-1.17317	-0.68714	-1.00749
AC008073.5	-0.78345	-0.7295	-1.38895	0.005964	-0.54915
AC008074.1	-0.02984	0.051235	-0.04549	-0.1291	0.01805
AC008114.25	0.397958	0.497016	0.69944	0.399021	0.532596
AC008132.13	0.049885	0.117062	0.340681	-0.02262	0.456135
AC008132.35	0.285916	0.225757	0.626483	0.404255	0.445175
AC008174.2	-0.75986	-0.53964	-0.84159	-0.85383	-0.64081
AC008175.3	0.181715	0.214343	0.22567	0.036017	0.337143
AC008393.7	0.052038	-0.01298	-0.04679	-0.12509	-0.05804
AC008413.7	-0.56915	-2.31031	-1.9706	-1.88523	-2.1136
AC008429.6 RPL26L1	-0.0759	-0.23805	-0.27277	-0.24658	-0.18323
AC008543.9	0.121977	0.233536	0.075369	0.081262	-0.15381
AC008560.6	0.255831	0.576856	0.67429	-0.02421	0.482399
AC008623.4	-0.54095	-0.41301	-0.60214	-0.30311	-0.56356
AC008686.8	0.347502	0.250042	0.227434	0.365628	0.163884
AC008686.8 C19orf53	0.147314	0.057193	-0.11289	-0.03196	-0.16491
AC008735.9	-0.04583	-0.24414	-0.05534	-0.27547	-0.52812
AC008738.7	-0.07076	0.130667	0.262163	0.080134	0.196975
AC008743.9	0.288933	0.402339	0.418883	0.183496	0.359498
AC009019.10	-0.27963	-0.20699	-0.4427	-0.0679	-0.19848
AC009027.10 CIRH1A	-0.69375	-0.99444	-0.86382	-0.81686	-0.87787
AC009053.7	0.342378	0.205102	0.371558	0.369277	0.511649
AC009065.8 TRAF7	-0.4654	-0.54716	-0.50154	-0.31826	-0.41251
AC009084.9	0.444686	0.406117	0.636128	0.172047	0.451816
AC009090.12 RSPRY1	-0.36023	-0.42343	-0.48317	-0.3437	-0.41099
AC009113.8	-0.18725	-0.01729	-0.13228	0.020615	-0.16124
AC009163.6	0.690574	0.986061	0.832725	0.669204	0.707673
AC009171.3	-0.1951	-0.14243	-0.11618	-0.15252	-0.19017
AC009271.7	0.73501	0.885138	1.026708	0.49279	0.662055
AC009365.9	0.274165	0.463774	0.319289	0.119739	0.408169
AC009412.5	0.2126	-0.01499	0.197843	0.279717	0.247545
AC009451.21	0.353351	-0.18469	0.480517	0.196722	0.413238
AC009477.4	0.057488	0.226586	0.212047	0.039359	0.322635
AC009501.4 MDH1	-0.75185	-0.78382	-0.81083	-0.66715	-0.49771
AC009509.7	0.666273	0.090032	0.223194	-0.26211	0.350502
AC009532.9	0.30879	0.300932	0.516227	0.170624	0.2946
AC009656.11	-0.10764	0.306388	0.264517	0.046616	0.201477

AC009758.8	0.159471	-1.09173	-0.96339	-0.82535	-0.47304
AC009779.18	-0.25528	-0.22121	-0.15024	-0.2235	-0.27139
AC009976.6	0.189166	0.08379	0.231868	-0.01687	0.007998
AC009996.7 EIF3S1	-0.04217	-0.05608	0.055194	-0.03055	0.02009
AC010088.4	0.374355	0.407074	0.611562	0.331728	0.235181
AC010170.3	0.181954	0.314646	0.300108	0.214921	0.372244
AC010178.40	0.075586	0.056351	0.0734	0.062222	0.288829
AC010409.6	0.54769	0.85234	0.77057	0.356844	0.51566
AC010441.6	0.085128	-0.02834	0.419192	0.197332	0.261379
AC010442.7 SLC9A3	0.163674	0.106715	0.233832	0.106745	0.263832
AC010598.6	0.337729	0.122325	0.340323	0.142118	0.069288
AC010619.7	0.271074	-0.93978	0.388245	-0.06277	0.018778
AC010624.8	0.184359	0.273401	0.025801	0.245835	-0.0576
AC010650.9	-0.33087	-0.04593	-0.22622	0.053916	-0.0754
AC010655.7	0.16173	0.021853	-0.1028	0.157362	-0.02234
AC010679.6	0.25888	0.205085	0.125556	0.126307	0.112252
AC010969.11	0.194948	0.568552	0.313328	0.103446	0.449862
AC010974.9	0.224568	0.690943	0.761922	0.030509	0.376841
AC010987.1	-0.11319	-0.088	-0.23318	-0.24284	-0.29474
AC011352.6	0.293585	0.275742	0.483244	0.13544	-0.03358
AC011379.7	0.216058	0.348216	0.405849	-0.04407	0.267018
AC011427.4	-0.04313	0.017282	0.189283	0.115452	0.199545
AC011448.5	0.189876	0.278374	0.23638	0.232127	0.337993
AC011450.4	-0.0857	-0.5972	-0.72803	-0.18996	-0.43388
AC011455.6 SIRT2	0.2664	0.358823	0.250805	0.160345	0.278581
AC011484.7	0.427586	0.720483	0.730018	0.34846	0.496702
AC011495.8	0.33404	0.574287	0.763915	0.532697	0.597406
AC011498.7	-0.39705	-0.83655	-1.0868	-0.59203	-0.80695
AC011499.5 HSD11B1L	-0.00529	-0.16359	-0.23512	-0.30092	-0.04417
AC011500.7	0.042721	-0.03654	0.087371	0.108743	0.015805
AC011740.7	0.228477	0.031393	0.119299	0.126956	0.232915
AC011816.17 MLH1	-0.71241	-0.46893	-0.94427	-0.65607	-0.37824
AC011895.4	-0.05026	-0.12099	-0.36367	-0.07569	-0.08647
AC012073.4 CENPO	-0.44884	-0.4081	-0.55159	-0.49529	-0.46414
AC012100.1	0.049085	0.280475	0.000532	0.099167	0.08291
AC012158.32	-0.13002	0.145854	-0.00439	-0.0395	0.003566
AC012307.8	0.501119	0.668872	0.718386	0.431751	0.661501
AC012413.10	0.403266	0.417732	0.514957	0.348922	0.411097
AC012467.9	-0.22879	-0.1104	-0.54026	-0.41897	-0.30177
AC012487.12 AC012487.12	-0.02958	-0.01027	0.012197	-0.02934	0.239259
AC012568.7 IQCH	0.098204	-0.17277	-0.26019	-0.30686	-0.30547
AC012615.4	0.17574	0.303151	0.142889	0.204413	0.188041
AC012622.6	-0.06366	-0.17251	-0.26734	-0.13424	-0.39719
AC013272.5	0.441038	0.772741	0.613532	0.657604	0.616431
AC013283.23 APAF1	-0.00731	-0.08353	-0.13523	-0.03375	-0.09656
AC013414.7	0.357966	0.661084	0.856263	0.465482	0.387074
AC013461.1	0.111461	0.242528	-0.07252	-0.08561	0.315964
AC013468.12	-0.61574	-0.42981	-0.62303	-0.47701	-0.8618
AC013472.5	0.094265	-0.01798	0.068584	-0.06683	-0.05165
AC013480.12	0.050265	0.082276	-0.13271	-0.0137	0.021252

AC013553.14	0.200479	-0.06707	-0.04806	-0.18226	0.070347
AC013726.7	0.293152	0.431514	0.586034	0.522355	0.521683
AC015723.8	0.554757	0.68354	0.692844	0.377922	0.416531
AC015804.14	0.151036	0.276394	-0.01928	-0.09204	-0.11524
AC015976.3	0.121991	0.215213	0.216911	0.146494	0.319364
AC015980.4 EHD3	-0.12225	-0.15658	-0.12001	0.009273	-0.00879
AC016136.21	0.469436	0.805469	0.745064	0.294954	0.044929
AC016450.10	0.16391	0.609932	0.530009	0.157043	-0.03673
AC016692.1	0.474754	0.573868	0.566359	0.549938	0.603758
AC016736.6 DBI	-0.51924	-0.32462	-0.51828	-0.32426	-0.15293
AC016753.9	0.158929	0.108517	0.104349	0.204184	0.337114
AC016772.9 PHOSPHO2	-0.41564	-0.18953	-0.49183	-0.42915	-0.34056
AC016889.28 G6PC	0.186997	-0.10217	0.331386	-0.43358	-0.09373
AC018452.11	-0.45908	0.20829	0.131469	0.325492	0.030818
AC018470.2	-0.21023	-0.06878	0.106437	-0.07543	0.177015
AC018506.5 SETD5	-0.25532	-0.04386	-0.03781	-0.16235	0.037477
AC018512.6 PDIA3	0.092876	0.269309	0.146059	0.07485	0.219108
AC018865.4	-0.13009	-0.20088	0.037541	-0.17709	0.06437
AC019206.4	-0.45193	-0.21212	-0.55171	-0.48092	-0.26586
AC020593.1	0.304869	0.234919	0.625034	0.450617	0.508936
AC020663.7	0.017796	-0.03011	0.083598	0.061664	0.056833
AC020763.6	0.044362	-0.05647	0.065621	0.163201	0.059557
AC020908.7	-0.38872	-0.36803	-0.20626	-0.3845	-0.47739
AC020916.8	0.098093	0.007546	0.04737	-0.04628	-0.0723
AC021089.4	-0.39079	-0.47397	-0.68169	-0.48813	-0.61489
AC021097.5	-0.3545	-0.34841	-0.64256	-0.26452	-0.34501
AC021180.6	0.068737	0.161825	0.437007	0.296437	0.272592
AC021317.19	0.062904	0.314131	-0.02269	0.023221	0.146371
AC021534.1	0.369952	0.427948	0.345361	0.400423	0.621276
AC021860.6 AC021860.6 KLF3	-0.22252	-0.32489	-0.36615	-0.22133	-0.32578
AC022007.5	0.001719	0.152867	0.130598	0.08221	0.059343
AC022007.5 FANCD2	-0.49193	-0.32122	-0.75546	-0.42804	-0.44484
AC022137.6	0.675736	0.600844	0.14677	0.012206	0.119271
AC022143.7	0.403477	-0.00487	0.46757	0.023945	0.235071
AC022150.6	0.749577	0.937623	0.834404	0.427628	0.496339
AC022188.13	-0.02674	-0.09261	0.177392	-0.0191	0.16658
AC022692.11	0.336189	0.448169	0.266805	0.258283	0.422179
AC022762.8	-0.34103	-1.17485	-0.94319	-0.94275	-0.6099
AC022893.6	0.230727	-0.1344	0.107197	0.353472	0.513662
AC023024.6	0.097072	0.418683	0.377927	0.384349	0.439002
AC023050.36	0.548883	0.556602	0.63402	0.367631	0.407401
AC023156.5	0.075834	0.44195	0.544972	0.090727	0.156943
AC023157.38	0.256722	0.091471	0.155936	-0.14953	0.06145
AC023886.7	0.502476	0.328158	0.249006	0.110276	0.341255
AC024032.5	0.467026	0.612972	0.682205	0.479583	0.458891
AC024085.5 CPA5	0.392185	0.563348	0.540009	0.268323	-0.07526
AC024132.7	0.18484	-0.41166	-0.11043	0.078892	-0.4348
AC024270.6	0.026331	0.179166	0.145958	0.109208	0.027435
AC024361.21	-0.0592	-0.2819	-0.53149	-0.13259	-0.11745
AC024558.21 TXNRD3	0.094474	0.185451	0.164516	0.002311	0.092863

AC024575.6 AC024575.6	0.188486	-0.09299	-0.11051	-0.01897	-0.04744
AC025165.27	0.000792	-0.24943	-0.16587	-0.07226	-0.63677
AC025260.29	-0.13589	0.138885	0.130234	-0.07071	-0.08554
AC025370.12 FZD6	0.144999	0.148607	0.036877	0.379907	0.120588
AC025449.6	0.382671	0.627981	0.42274	-0.07344	0.178267
AC025573.13	0.013777	0.091851	0.269661	0.160957	0.20921
AC025594.5	-0.02306	0.163704	-0.17614	-0.02081	0.034036
AC025917.8	0.472591	0.263979	0.403234	0.186072	0.362247
AC026120.33	0.198245	0.398833	0.379169	0.273009	0.360035
AC026250.16	0.0439	0.154963	0.345074	0.115617	0.188198
AC026458.8	0.321635	-0.04859	0.325854	-0.14122	0.006525
AC026461.11	-0.26651	0.025497	0.40573	0.080315	0.191103
AC026468.4	0.421424	0.67191	0.561119	0.323363	0.483901
AC026495.13	0.437443	0.369073	0.322671	0.274036	0.366402
AC026698.6	-0.21458	-0.21179	-0.38317	-0.33665	-0.40184
AC026713.5	-0.15909	-0.139	-0.05748	-0.03827	-0.01869
AC026790.5 BASP1	-0.83378	-0.54647	-0.5868	-0.73757	-0.72939
AC026801.7	0.158736	0.353743	0.266285	0.147059	0.427216
AC026954.14	-0.15891	0.05314	-0.15886	-0.0852	0.179074
AC027228.16	-0.2314	-0.1726	-0.26251	-0.12849	-0.23583
AC027307.5	0.168048	-0.19287	-0.04605	0.070661	0.079534
AC027674.10	0.739848	0.714435	0.882384	0.705583	0.660174
AC027763.2	0.39171	0.307592	0.541511	0.365685	0.516151
AC027763.2 RNASEK C17orf49	0.081531	-0.20409	-0.09782	0.093477	-0.16477
AC034139.7	0.333726	0.392667	0.557776	0.004182	0.212086
AC034193.5	-0.06671	0.110288	-0.20204	0.228814	0.048721
AC034193.5 VHL	0.396146	0.275458	0.51076	0.294405	0.386659
AC034222.5	-0.05557	-0.03011	-0.04777	-0.10404	-0.16748
AC034229.4 CCT5	-0.12426	-0.17121	-0.19445	-0.21396	-0.15267
AC034236.1	0.140266	-0.28981	0.372724	0.319163	0.44648
AC037487.12	0.070387	-0.02606	-0.16964	-0.14968	-0.05698
AC044802.6	0.266996	0.56864	0.612994	0.386671	0.583665
AC044802.7	0.138996	-0.08255	0.005563	-0.07447	-0.02662
AC046185.14	0.039399	-0.33683	-0.62949	-0.04206	-0.40014
AC048382.7	-0.10108	-0.37622	-0.33768	-0.33898	-0.3064
AC051635.7	0.046361	-0.03948	0.501248	0.298288	0.316899
AC053545.4 BBS12	-0.70244	-0.70949	-0.685	-0.48031	-0.57743
AC055811.2	-0.02932	-0.08974	-0.05689	-0.13493	0.200411
AC055811.3	0.285007	0.307948	0.268886	0.000121	0.1922
AC055866.19 RPL27	-0.27635	-0.4885	-0.56726	-0.77414	-0.58312
AC062028.6 C2orf50	0.303759	0.53722	0.463324	0.234407	0.416768
AC063977.6	-0.47308	-0.86892	-0.8691	-0.67843	-0.56004
AC064836.1	-0.62897	-0.70905	-1.09785	-0.63198	-0.58795
AC064853.6	0.522373	0.638276	0.603709	0.415919	0.328948
AC067942.6	0.528046	0.564155	0.691225	0.280501	0.256229
AC068473.19 CTDP1	0.078306	0.038024	0.237622	0.161313	0.296794
AC068499.1	-0.08109	-0.09075	-0.31409	-0.04888	-0.26552
AC068533.6	-0.0551	-0.13767	-0.20992	-0.03002	-0.04725
AC068657.6 TTC1	0.228889	0.370046	0.296517	0.049859	0.083636
AC068792.31	-0.10061	-0.05368	-0.06971	-0.12248	0.02987

AC069154.5	-0.23668	0.132006	-0.14797	0.053319	-0.03457
AC069234.35	0.105319	0.050221	0.132425	0.128287	0.187672
AC069236.27	-0.34082	-0.44087	-0.39253	-0.21041	-0.39699
AC069257.28	0.16035	0.286666	0.489745	0.152765	0.24449
AC069282.6	0.472846	0.515771	0.612936	0.358422	0.013299
AC069513.28	0.256075	0.21264	0.431234	0.309939	0.3226
AC069525.16	0.392416	0.575164	0.580996	0.426663	0.602844
AC073072.11	0.399744	-0.87728	-0.89712	-0.34018	-0.38314
AC073082.6	0.392196	0.383081	0.430477	0.021992	0.291521
AC073135.3	0.003448	0.00442	-0.03873	0.114094	0.037449
AC073183.7	-0.17357	-0.11577	-0.60809	-0.42525	-0.47222
AC073264.5	0.349173	0.458772	0.173745	0.372348	0.344638
AC073347.3	-0.02679	0.050558	-0.00016	0.073721	0.124502
AC073476.6	0.226049	0.266001	0.180309	0.238035	0.302451
AC073487.34 ORMDL2	-0.59316	-0.29084	-0.41942	-0.45136	-0.42754
AC073517.6	-0.13077	-0.03115	-0.57487	0.045958	-0.41192
AC073585.8	0.04128	0.250049	0.018038	-0.02237	0.249054
AC073624.1	-0.05183	0.106618	-0.03552	0.008419	0.136851
AC073624.28	0.374232	0.362044	0.366141	0.241451	0.290376
AC073635.3	0.35238	0.474491	0.527231	0.162251	-0.13977
AC073834.6	-0.11472	-0.6868	-0.58643	0.395068	-0.29187
AC074029.15	-0.34845	-0.27714	-0.50149	-0.26615	-0.44995
AC074130.3	-0.42914	0.705596	0.016617	0.440437	0.641865
AC074389.6	0.013864	0.204267	0.369839	-0.04712	0.070372
AC078802.14	0.058626	-0.03091	-0.23592	0.097403	-0.00974
AC079061.8	0.524383	0.777438	0.666519	0.411075	0.53657
AC079135.6	0.651998	0.744098	0.600284	0.502027	0.269492
AC079140.6	0.216667	0.411827	0.136648	0.38009	0.387141
AC079305.6 AGPS	0.241629	0.179859	0.880549	-0.03833	0.08875
AC079315.30	0.608586	0.694306	0.724412	0.458512	0.385321
AC079325.7	0.048127	0.19596	0.280902	-0.0333	-0.07439
AC079610.4 SPAG16	-0.3882	-0.54337	-0.64122	-0.42658	-0.4612
AC079612.1	0.307493	0.557508	0.674805	0.376235	0.500885
AC079776.5	0.17109	-0.20239	-0.45291	-0.13494	-0.00932
AC079780.2	-0.20798	0.014788	-0.34922	-0.16681	-0.12114
AC079789.7	0.593258	0.924894	1.046747	0.14142	0.388114
AC080080.5	0.509742	0.581557	0.502647	0.340566	0.300818
AC080112.15	-0.38681	-0.22613	-0.5109	-0.34724	-0.41317
AC084125.9 AC084125.9	0.017536	0.123941	-0.08562	0.072574	0.30265
AC084199.28 HSP90B1	-0.59781	-0.43274	-0.59841	-0.57709	-0.47069
AC087071.2	-0.25119	-0.45334	-0.68569	-0.49204	-0.64045
AC087269.5	-0.01994	0.092722	0.166946	0.356907	0.157714
AC087294.18	0.097548	0.126211	-0.1593	0.023	0.103473
AC087312.8	0.606194	0.87622	0.969363	0.739073	0.741245
AC087350.3	0.210373	0.106427	-0.05152	0.293282	0.146955
AC087623.21	-0.01922	-0.36098	-0.36751	-0.5381	-0.29874
AC090453.4	-0.15982	-0.59457	-0.43218	-0.57069	-0.80072
AC090520.1 TSGA13	-0.21706	-0.17634	-0.30139	-0.17362	-0.03349
AC090587.1	0.05577	0.044959	-0.4229	-0.09138	0.063756
AC090616.2	0.213908	0.025327	-0.4657	-0.22315	-0.02446

AC090625.5	0.150265	0.305605	0.464591	0.07655	0.310288
AC090670.11	-0.02868	-0.34531	-0.61556	-0.48694	-0.71327
AC091060.3	-0.3757	-0.11676	-0.22535	-0.22423	-0.07225
AC091073.3	0.248719	0.301848	0.042679	-0.07743	-0.02567
AC091152.18	0.067188	-0.08285	-0.16602	-0.42833	-0.27893
AC091304.15	0.436223	-0.38896	0.09896	0.02082	0.20507
AC091544.11	0.185923	0.478955	0.313793	0.097823	0.515907
AC091654.4	0.118339	0.154169	-0.00143	0.101401	0.150763
AC091736.2	0.612749	0.737883	1.029299	0.571398	0.727583
AC091817.6	-0.71994	-0.2201	-0.76741	-0.66648	-0.71314
AC091842.3	-0.08238	-0.06316	-0.16795	-0.3306	-0.33516
AC092031.4	0.15695	0.502066	0.445169	0.2584	0.450045
AC092117.4	-0.03964	-0.17213	-0.04095	-0.01122	-0.06956
AC092142.5	0.330289	0.202457	0.30239	0.26691	0.401621
AC092373.2	-0.07718	-0.04679	-0.25014	-0.44649	-0.57561
AC092436.5	-0.25505	0.088448	-0.05845	-0.1497	0.043051
AC092610.10	0.525786	0.687767	0.824953	0.522841	0.609082
AC092636.3	0.537841	0.621331	0.66913	0.388043	0.710917
AC092653.3	0.491467	0.640734	0.373463	0.426867	0.269148
AC092656.1	0.198807	0.14157	0.009634	-0.10848	-0.12926
AC092839.4	-0.1562	-0.18775	-0.32044	-0.13859	0.111474
AC092849.4	0.345735	0.564406	0.691781	0.439573	0.206515
AC092892.5	-0.18198	-0.20294	-0.24207	-0.19369	-0.10224
AC092981.3	-0.4377	-0.22799	-0.49983	-0.36824	-0.48612
AC093230.2	0.324143	0.03288	0.519715	0.269122	0.241954
AC093251.2 IQGAP2	-0.13687	0.03808	-0.04504	0.022372	0.16094
AC093310.1	-0.27024	-0.28772	-0.31325	-0.19098	-0.3073
AC093323.3	0.141172	0.303911	0.108172	0.131368	0.144431
AC093331.9	0.582478	-1.43126	-0.16155	0.395905	0.712796
AC093376.5	-0.32035	-0.4352	-0.42658	-0.21784	-0.37604
AC093388.4	0.207338	0.182535	-0.18538	-0.28118	-0.3112
AC093510.2 POLR3G	0.160077	0.005988	-0.06481	0.034769	0.178864
AC093512.2	0.316397	0.304084	0.351628	0.145461	0.251168
AC093520.4	-0.12242	-0.7568	-0.6582	-0.24029	-0.32071
AC093638.4	-0.59942	-1.07906	-1.36334	-0.35434	-0.99197
AC093642.5	0.381493	0.364555	0.397626	0.681869	0.708045
AC093700.4	-0.08351	0.090819	-0.1026	-0.17667	-0.03109
AC093726.4 HTR5A	0.21711	0.195492	0.650138	0.418915	0.394581
AC093729.2	0.6029	0.18028	0.245714	0.44324	0.256671
AC093788.3	-0.25159	-0.23463	-0.32198	-0.28892	-0.1639
AC093835.3	0.07419	-0.17348	-0.03864	-0.02652	-0.19919
AC093838.3	0.194515	0.329691	0.362868	0.111209	0.182625
AC093852.3	-0.38791	-0.62693	-0.40853	-0.30815	-0.43526
AC096587.1	-0.79026	-0.72132	-0.82769	-0.69003	-0.51035
AC096768.1 MRPS18C	-0.55109	-0.55448	-0.76003	-0.5028	-0.7348
AC097359.2 GOLGA4	0.163727	0.017955	-0.13212	0.090857	0.309453
AC097458.2	0.398924	0.589679	0.470771	0.396853	0.538803
AC097534.3	0.271506	0.411826	0.383916	-0.10075	-0.04207
AC097658.2 GAB1	-0.31785	-0.52036	-0.31375	-0.17901	-0.22101
AC097717.3	0.309481	0.263622	0.042911	-0.03793	-0.02627

AC097720.1 WDR43	-0.02569	-0.06194	-0.07073	-0.0208	-0.03775
AC099489.2	0.22212	0.287746	0.460689	0.235175	0.368776
AC099513.2	0.483222	0.170503	-0.0491	0.116939	0.336746
AC099518.3	0.196576	0.213365	0.208192	0.166437	0.303228
AC099522.3	-0.17519	-0.1107	-0.25653	-0.28055	-0.09547
AC099784.2	0.410085	0.561399	0.442847	0.388388	0.30782
AC099849.4	0.393646	0.424769	0.202834	0.140603	0.334401
AC100756.4	0.383035	0.096256	0.12823	0.064674	0.318808
AC100793.8 RAMP2	0.152979	0.145821	0.326342	-0.08414	0.284439
AC103564.5	0.083431	0.072431	0.158907	0.244515	0.295273
AC103849.3	0.688621	-0.77527	-0.25723	0.175949	0.235326
AC104066.3	0.272913	0.446365	0.500475	0.316107	0.332446
AC104073.3	0.218426	0.126536	0.636512	0.415472	0.480638
AC104333.2	0.278942	0.392906	0.339666	-0.01828	0.164629
AC104333.2 FLVCR1	0.021827	-0.00497	-0.08869	-0.16911	0.004275
AC104389.8 HBG1	0.32016	-1.5611	-1.37927	-1.05413	-0.83231
AC104564.11 TP53I13	-0.03812	0.04029	-0.02583	0.055092	0.121381
AC104698.2	-0.36683	-0.14303	-0.22298	-0.17674	-0.17705
AC104819.4	0.069929	0.051758	-0.01091	0.037181	0.012806
AC105001.3	-0.149	-0.17009	-0.27069	-0.27496	-0.36355
AC105046.10	0.224515	0.328076	0.484104	0.036876	0.320173
AC105129.4	0.048289	0.12186	0.351462	0.080286	0.240794
AC105219.6	0.529305	0.888849	0.73523	0.674112	0.789962
AC105339.9	-0.58636	-0.7151	-0.97407	-0.81755	-0.78882
AC105345.3	0.023472	0.246798	0.240719	0.140111	0.371126
AC105391.1	0.177129	-0.03832	0.572891	0.240169	0.195295
AC105398.2	0.709431	0.665749	0.824729	0.58213	0.377598
AC105398.3	0.111912	0.031622	0.348195	-0.11523	0.152208
AC105915.5	0.559478	0.640003	0.920196	0.598723	0.745554
AC106736.3	0.122223	0.113097	0.20598	0.075872	0.177458
AC106819.3	0.429644	0.682434	0.749403	0.413729	0.52027
AC106819.5	0.072598	0.055332	0.071665	0.002321	-0.05214
AC106886.3 RNF40	-0.06816	-0.13722	0.074582	0.076862	-0.07635
AC106900.1 SCRN3	-0.0997	-0.14117	-0.3463	-0.25118	-0.02497
AC107071.3	-0.67678	-0.45457	-0.52538	-0.46611	-0.67045
AC107081.5	-0.33866	-0.43386	-0.56625	-0.5306	-0.26806
AC108142.1	-0.32355	-0.25078	-0.16159	-0.13975	-0.09929
AC109319.17 FKBP10	-0.2671	-0.22467	-0.11175	-0.08005	-0.16257
AC109326.11	-0.71519	-0.84138	-1.01539	-0.58056	-0.73134
AC109326.11 TMEM106A	0.297438	0.301823	0.655368	0.357368	-0.54852
AC109583.2	0.426373	0.576774	0.598315	0.412536	0.318298
AC110079.3	0.011883	-0.43192	-0.42585	-0.33126	-0.43307
AC110491.5	0.653682	0.760238	0.719271	0.253496	0.644505
AC110760.3	0.238572	0.142608	0.43646	0.245956	0.342863
AC110814.5	-0.05549	-0.04628	-0.00703	-0.00438	-0.06402
AC110998.4	0.095728	-0.07005	-0.22337	-0.52306	-0.4576
AC111200.7	0.534313	0.402297	0.431131	0.151871	0.221411
AC112128.4	-0.34926	-0.30741	-0.17349	-0.48005	-0.54169
AC112491.4	-0.18971	-0.28394	-0.31759	-0.24118	-0.3657
AC113191.13	0.235935	-0.16334	-0.21178	-0.14359	-0.30499

AC114772.5	0.553084	0.65898	0.780247	0.328856	0.608157
AC114803.5	0.04523	0.803655	0.863935	0.513722	0.597344
AC114947.1	0.038354	-0.18433	0.094055	0.037386	-0.09635
AC114967.2	-0.61279	-0.44868	-0.69869	-0.62931	-0.63725
AC115090.8	-0.25792	-0.34089	-0.31364	-0.32265	-0.36454
AC116334.1	-0.95957	-0.72571	-0.79394	-0.74552	-1.17965
AC116342.2	-0.06778	-0.05901	-0.10756	-0.05587	-0.02674
AC116366.4	0.064863	0.068028	0.21486	0.300717	0.197842
AC116655.7	0.665986	0.358937	0.606799	0.514272	0.325282
AC116904.7	-0.26823	-0.13271	-0.17903	-0.10508	0.141067
AC117398.3	0.196399	0.47409	0.56724	0.434415	0.430323
AC118758.3	0.523952	0.926952	0.288452	0.161248	0.400852
AC120024.22	0.076131	0.03679	-0.00734	0.122964	0.147217
AC120498.2	0.360184	-0.15853	0.082347	0.319981	0.297477
AC120498.2 UBE2I	-0.18936	-0.21673	-0.12121	-0.13622	-0.08626
AC121493.1	-0.23063	-0.37098	-0.39304	-0.21034	-0.46092
AC122684.3	0.193779	-0.38472	-0.80593	-0.2631	0.014256
AC124798.5	0.1683	0.986727	0.859605	0.36753	0.459103
AC125611.4	-0.07071	-0.33489	-0.39007	0.082085	-0.21852
AC126182.2	0.140843	0.335078	0.202323	-0.22418	-0.09007
AC126281.3	-0.25844	-0.80512	-0.16699	-0.28728	-0.56179
AC126323.6	0.414321	0.228164	0.319392	0.051218	0.226997
AC126365.6	0.608251	0.593362	0.747437	0.340944	0.432568
AC127496.5	-0.19766	-0.50383	-0.36931	-0.3356	-0.27016
AC127496.5 BAIAP2	-0.16976	-0.19607	-0.17356	-0.25537	-0.154
AC128677.4	-1.929	-2.02485	-2.31075	-2.21013	-2.47588
AC129507.9	0.166699	0.254359	0.148895	0.130977	0.117562
AC130366.5	0.444207	-0.08988	0.137975	-0.148	-0.17412
AC130416.4	0.487777	0.699862	0.896493	0.426342	0.492262
AC130449.1	0.209155	0.336808	0.318368	0.281503	0.313186
AC130454.2	0.194702	0.515247	0.509196	0.374312	0.497877
AC131097.1	0.376712	0.768301	0.480387	0.231761	0.546049
AC131281.7 ZNF596	-0.42884	-0.2477	-0.64663	-0.42051	-0.40857
AC131952.1	0.04691	-0.09358	0.253725	0.284931	0.164814
AC132938.1	0.443289	0.463316	0.665685	0.400259	0.40654
AC132938.9 HEXDC	0.227242	0.039719	-0.01899	-0.07461	0.055778
AC133485.1	0.707837	-0.3381	-0.62521	-0.01207	-0.49664
AC133555.3	-0.12953	-0.14018	-0.22107	-0.05733	-0.21396
AC133644.1	-0.52668	-0.50052	-0.3061	0.334086	-0.37866
AC134504.2	0.253639	0.651189	0.518264	0.200793	0.368474
AC134775.8	0.095802	0.017227	0.202779	0.075583	0.049743
AC135048.2 FBXL19	-0.06917	-0.05086	-0.08042	-0.01365	-0.06671
AC135050.5	0.417093	0.408289	0.486452	0.391835	0.574011
AC135052.2	0.179441	0.021605	0.408665	0.014269	0.002082
AC135457.2	0.340508	0.396215	0.169879	0.126679	0.224782
AC135592.2	0.325849	0.274624	0.717273	0.368031	0.464182
AC135891.3	-0.51757	-0.35611	-0.03618	0.170276	-0.00891
AC135995.7	-0.35472	-0.0981	-0.30858	-0.1997	-0.04934
AC136604.2	0.184827	0.289103	0.15431	0.177439	0.154372
AC136698.6	-0.22732	0.120408	-0.18473	-0.39276	-0.25782

AC136704.9	0.130198	0.33934	0.315193	0.16031	0.329673
AC136932.4	0.029081	-0.17911	0.159953	0.128185	0.113311
AC138028.1	0.279647	0.115481	0.433499	0.242169	0.141735
AC138356.3	-0.08087	-0.18107	-0.10142	-0.08933	-0.18107
AC138811.3	-0.23858	-0.23036	-0.249	-0.0771	-0.36492
AC138932.4	0.778821	0.225122	0.328416	0.026274	-0.01835
AC138956.3 HIGD2A	-0.29609	-0.28827	-0.49716	-0.40657	-0.44642
AC139026.2	0.15362	0.26992	0.049099	-0.09523	-0.04817
AC139277.2	-0.51763	-0.4918	-1.08114	-0.51665	-0.61274
AC139451.7	-0.10381	-0.06976	-0.31859	-0.44559	-0.14132
AC139713.1	0.43902	-0.25411	0.495679	0.399874	-0.14717
AC139768.14	-0.19294	-0.03787	-0.02335	0.019263	0.088775
AC139887.3	-0.14439	-0.12562	-0.24886	-0.07883	-0.21826
AC140077.17	0.263372	0.036013	0.372298	0.26447	0.21416
AC140481.3	0.218152	0.46725	0.648805	0.631395	0.549452
AC145098.2	0.079805	0.056488	0.10562	-0.01432	0.05463
AC145124.2	-1.31971	-1.15034	-1.49402	-0.94609	-1.19033
AC145132.2 RAD17	-0.24919	-0.3431	-0.45384	-0.38681	-0.52296
AC145146.2	-0.51597	-0.86686	-1.45435	-0.80771	-1.02973
AC145423.8	0.098814	-0.29327	-0.07059	-0.13156	-0.02696
AC147362.2	0.329849	0.658129	0.707513	0.445258	0.590349
ACAA1 MYD88	-0.05517	-0.08022	0.020311	-0.23456	0.066646
ACAA2 AC090227.10	-0.25076	-0.20013	-0.4063	-0.28446	-0.1743
ACACA TADA2L	-0.25432	-0.18058	-0.34077	-0.30817	-0.20125
ACACB	0.407577	0.560786	0.587172	0.33628	0.527013
ACAD9	-0.36938	-0.47656	-0.61782	-0.3995	-0.61357
ACADL	0.515707	0.658365	0.500074	0.466269	0.290257
ACADM	-0.27074	-0.31757	-0.48377	-0.50104	-0.24993
ACADS	0.285902	0.399607	0.407117	0.281161	0.350635
ACAN	0.171419	0.069219	0.060606	0.162997	0.170749
ACAT1	0.054933	0.180937	-0.13697	-0.07486	-0.16115
ACAT2	0.16601	-0.10835	-0.37842	-0.16439	-0.33114
ACBD3	0.157453	0.005263	0.012163	-0.01649	0.087988
ACBD4	0.308588	0.221961	-0.00209	0.027605	0.245372
ACBD5	-0.28109	-0.72844	-0.78511	-0.0245	-0.05258
ACBD6	0.014177	-0.28947	-0.18755	-0.12505	-0.01517
ACBD7	0.199701	0.279404	0.474041	0.193006	0.253581
ACCN1	0.26283	0.261683	0.333246	0.379519	0.319813
ACCN2	-0.03518	-0.0306	-0.17594	-0.14338	-0.25004
ACCN3	0.424888	0.668995	0.594257	0.359336	0.538448
ACCN4	-0.16298	-0.50032	-0.6171	-0.51751	-0.2659
ACCN5	0.135557	-0.14028	0.237575	-0.07677	-0.74223
ACCS	0.336803	0.366427	0.253319	0.443326	0.53242
ACD PARD6A	0.18709	0.177181	0.149797	0.168978	0.092953
ACE2	0.206207	0.688368	0.103813	0.055499	0.109322
ACHE	-0.28331	-0.34156	-0.29703	0.048067	-0.19651
ACIN1 C14orf119	-0.58042	-0.69567	-0.96975	-0.49425	-0.70204
ACLY	-0.1018	-0.08653	0.029291	-0.34393	-0.13264
ACMSD	-0.29152	-0.41012	-0.44494	-0.36123	-0.27415
ACN9	0.129284	0.325167	0.250487	0.133491	0.248984

ACO1	-0.42569	-0.38868	-0.52198	-0.25531	-0.49007
ACOT12	0.322847	0.564685	0.632188	0.078916	0.504913
ACOT2	0.319028	0.302302	0.080134	0.118477	0.214969
ACOT4	0.324953	0.668585	0.713597	0.447287	0.512782
ACOT6	0.339706	0.565924	0.415478	0.197861	0.194956
ACOT8 ZSWIM3	0.070318	-0.19865	-0.02919	0.032242	-0.04524
ACOT9	0.173777	0.01639	0.262267	0.052629	-0.06848
ACOX1 AC040980.1	-0.13356	-0.34221	-0.63442	-0.39235	-0.41525
ACOX2	0.074466	0.029703	0.274823	0.035747	0.264045
ACOX3 C4orf23	-0.0888	-0.35599	-0.21401	-0.28305	-0.21509
ACOXL	0.72294	0.380623	1.112336	0.674596	0.590975
ACP2 NR1H3	-0.49677	-0.33768	-0.6921	-0.40962	-0.28765
ACP5	0.174987	0.174544	0.177892	0.168861	0.151611
ACP6	0.170847	0.148671	0.009133	-0.01244	0.051392
ACPL2	-0.0534	0.004486	-0.04461	0.078399	-0.1223
ACPP	0.287307	0.163605	0.229381	0.137393	0.133591
ACPT	2.590097	0.33543	0.292724	-0.05763	0.001148
ACR	0.12311	-0.05699	-0.07328	-0.06435	-0.1956
ACRBP	0.26309	0.57275	0.488432	0.411496	0.493695
ACRC	0.691506	0.735963	0.935114	0.588817	0.81287
ACRV1	-0.13532	-0.03384	-0.60426	-0.39039	-0.6148
ACSBG1	0.171409	0.37231	0.38011	0.216026	0.433489
ACSBG2	0.228385	-0.08555	0.556449	-0.05638	0.484911
ACSF3	0.055739	0.323296	0.301457	0.235416	0.195237
ACSL1	0.129012	0.239767	0.459166	0.232694	0.286847
ACSL3	-0.10139	0.073693	0.019885	-0.06026	-0.06723
ACSL4	0.348061	0.212028	0.370306	-0.02479	0.035
ACSL5	0.261834	0.516387	0.415456	0.190907	0.340901
ACSL6	0.060969	0.211114	0.149304	0.135112	0.095703
ACSM1	-0.04589	-0.97626	-0.94761	-1.16025	-0.86719
ACSM2	-2.09628	-2.1262	-1.82425	-1.70185	-2.17764
ACSM2A	-1.77102	-2.13776	-1.97016	-1.76601	-2.02305
ACSM3	0.094069	0.255647	-0.87402	-0.80899	-0.75404
ACSM5	0.203569	0.26412	0.341768	0.300033	0.262205
ACSS1	0.284152	-0.20465	0.332612	0.373041	0.25229
ACSS3	0.448907	0.346978	-0.01777	0.52893	0.582181
ACTA1	0.054329	0.247796	0.79839	0.485949	0.475176
ACTB	0.056742	0.109858	0.051574	0.080269	0.167589
ACTBL2	0.272017	0.331969	0.477185	0.271389	0.167285
ACTC1	0.134562	-0.05339	0.13851	0.026778	0.220723
ACTG1	-0.33547	-0.37018	0.035836	-0.22561	0.086335
ACTG2	0.655259	0.679415	0.77851	0.662453	0.684314
ACTL6A	-0.01583	-0.12796	-0.04537	0.01258	-0.05914
ACTL6B	0.235627	0.088091	0.054946	0.110509	0.087528
ACTL7A	0.528082	0.52686	0.431949	0.230898	0.442607
ACTL7B	0.187276	0.111378	0.223704	0.270737	0.179495
ACTL8	0.212749	0.500618	0.509532	0.3421	0.458024
ACTN1	-0.38217	-0.16806	-0.28038	-0.19645	-0.08694
ACTN2	0.034336	-0.05732	0.058474	-0.08848	0.133526
ACTN4	-0.07204	0.11374	0.036972	0.079893	-0.02001

ACTR10	-0.29176	-0.04424	-0.21621	-0.22074	-0.3227
ACTR1A SUFU	-0.81744	-0.73723	-0.83961	-0.74225	-0.75382
ACTR1B AC017099.11	-0.05187	-0.05158	-0.14651	0.04692	0.102314
ACTR2	-0.00508	0.074458	-0.09823	-0.08462	0.149495
ACTR3	-0.69955	-0.52448	-0.53331	-0.50425	-0.31175
ACTR3B	0.036566	0.02901	-0.0803	-0.02163	-0.21118
ACTR5	0.099991	-0.08415	-0.0023	-0.04575	-0.07281
ACTR6	-0.51864	-0.58166	-0.83431	-0.49461	-0.63702
ACTR8	-0.1222	0.15902	-0.20593	-0.16487	-0.14443
ACTRT1	-0.14592	0.055961	-0.14508	-0.01699	-0.10669
ACVR1	0.170471	-0.87361	-1.20021	-0.68861	-0.64024
ACVR1B	0.022697	-0.14204	0.018725	-0.09472	-0.12194
ACVR1C	-0.10888	0.128488	-0.16678	-0.17384	-0.05659
ACVR2A	-0.24758	-0.1048	-0.13601	-0.209	-0.06177
ACVR2B	-0.17581	-0.04455	-0.11756	-0.05891	-0.08632
ACVRL1	0.118206	0.270943	0.36101	0.100252	0.278576
ACY1	0.230223	0.124153	0.017655	0.127547	0.215178
ACY3	0.526088	0.249402	0.602789	0.271564	0.569739
ACYP1.	-0.11398	-0.21217	-0.22564	-0.22449	-0.27243
AD000671.1	0.065382	-0.12325	-0.04039	-0.06308	0.159995
ADA	0.137835	0.133675	0.247329	0.268492	0.240921
ADAD1	0.378033	0.554628	0.812769	0.38762	0.639126
ADAM10	-0.09309	0.153131	-0.08952	-0.28155	-0.25661
ADAM11	0.146933	0.086384	0.22927	0.234904	0.256006
ADAM12	0.234064	0.279678	0.513648	0.330034	0.403872
ADAM17	-0.55646	-0.34253	-0.46212	-0.48136	-0.49748
ADAM18	0.654321	1.034123	1.24531	0.541216	0.782586
ADAM19	-0.18173	0.09047	0.074332	-0.02148	0.050521
ADAM2	0.586926	0.43681	0.701146	0.371376	0.475115
ADAM20	-0.66624	-0.54247	-0.78581	-0.78695	-0.72034
ADAM21P.	-0.308	-0.84437	-0.16266	-0.46519	-0.87919
ADAM22	-0.33914	-0.36129	-0.73063	-0.39799	-0.15183
ADAM23	-0.17841	0.08763	-0.136	-0.18735	0.052444
ADAM28	-0.12857	0.174319	-0.42054	0.01015	-0.24905
ADAM29	0.257273	-0.36333	-0.11251	-0.50868	-0.37942
ADAM30	0.395064	-0.34067	0.587473	0.270037	0.481088
ADAM32	0.422845	0.69739	0.872592	0.424288	0.447288
ADAM33	0.201006	0.171857	0.42954	0.244424	0.232592
ADAM7	0.023451	-0.32769	-0.45963	-0.14599	-0.26913
ADAM8	0.239154	0.153041	0.233174	0.066743	0.12918
ADAMDEC1	0.240693	-0.2714	-0.56816	-0.24904	-0.24839
ADAMTS1	-0.43064	-0.18635	-0.05992	-0.25427	-0.32192
ADAMTS10	-0.87364	-0.74273	-0.78482	-0.19863	-0.33596
ADAMTS12	-0.61172	-0.70689	-0.20946	-0.20072	-0.07427
ADAMTS13	0.535241	0.604805	0.59309	0.330046	0.308112
ADAMTS13 C9orf7	-0.15508	-0.0605	-0.41089	-0.16808	-0.25399
ADAMTS14	0.026359	0.035598	0.081217	-0.002	-0.00358
ADAMTS15	-0.22044	0.259597	0.28465	0.111262	-0.04772
ADAMTS16	0.073872	0.014302	0.214857	0.174363	0.222972
ADAMTS17	0.243551	-0.01455	-0.12804	-0.08729	0.085582

ADAMTS18	0.066957	-0.04228	0.259457	0.211732	0.292423
ADAMTS19	-0.58381	0.091067	-0.68093	0.072649	-0.48768
ADAMTS2	0.007155	0.028178	0.023977	0.008905	0.12503
ADAMTS20	0.037226	0.252857	0.283576	-0.13143	0.333128
ADAMTS3	-0.65961	-0.48116	-0.51902	-0.15978	-0.58199
ADAMTS4 NDUFS2	0.279301	-0.40887	-0.50221	0.05602	0.051607
ADAMTS5	-0.28819	-0.01091	-0.15257	-0.12498	-0.3009
ADAMTS6	-1.50144	-1.31841	-1.3324	-1.13412	-1.31401
ADAMTS7	0.090682	0.098202	0.211713	0.107992	0.021947
ADAMTS8	-0.01476	0.277213	0.00944	0.201071	-0.06178
ADAMTS9	-0.48901	-0.55116	-0.11212	-0.39892	-0.45979
ADAMTSL2	0.151877	0.079019	-0.06868	-0.0688	-0.16419
ADAMTSL3	-0.01643	0.223534	0.348755	0.194652	0.248853
ADAMTSL4	-0.05074	0.052616	0.162572	-0.02552	0.01309
ADAMTSL5	0.015888	-0.09737	-0.03426	0.231086	0.071159
ADARB1	-0.01882	-0.13926	-0.06563	-0.01003	0.006275
ADARB2	-0.23556	-0.15863	-0.26089	-0.25331	-0.17796
ADAT1	0.004063	0.116743	0.006411	0.078253	-0.15435
ADAT2	0.012256	0.040006	0.044347	-0.24013	-0.15013
ADAT2 PEX3	-0.67109	-0.3334	-0.7669	-0.64353	-0.70298
ADC	0.208268	0.087477	-0.46355	-0.23638	-0.13893
ADCK1	0.133591	0.317945	0.181345	0.178135	0.097973
ADCK2	0.026573	-0.02421	0.130568	-0.10895	0.024287
ADCK4 ITPKC	-0.2755	-0.3056	-0.22649	-0.08031	-0.29165
ADCK5	0.079035	0.119232	-0.07576	0.014428	0.110635
ADCY1	0.18156	0.294085	0.27657	0.228724	0.337268
ADCY10	0.342008	0.683324	0.399125	0.272369	0.376218
ADCY2	0.066605	0.024924	0.288687	-0.00898	0.043507
ADCY3	0.259611	0.317622	0.27872	0.140697	0.366772
ADCY4	-0.03387	-0.08253	0.114497	0.35639	0.019212
ADCY5	0.121388	0.076177	0.363146	0.200981	0.334364
ADCY7	0.184122	0.130801	0.405317	0.063864	0.308168
ADCY8	0.120196	0.390076	0.367283	-0.03795	0.173455
ADCY9	0.121095	0.125724	0.16798	0.218703	0.173463
ADCYAP1	-0.27901	0.251229	0.463199	-0.22433	-0.07656
ADCYAP1R1	-0.06632	0.055237	0.085258	-0.03941	0.1885
ADD1	0.018058	-0.08001	-0.08997	0.102539	0.017506
ADD2 FIGLA	-0.02611	-0.08736	-0.02661	0.002945	0.162861
ADFP	0.005369	-0.22259	-0.15521	0.085659	0.288679
ADH1B	-0.6418	-0.32296	-0.30433	-0.45175	-0.79816
ADH1C	-0.47358	-0.32836	-0.34183	-0.40277	-0.5434
ADH4	-0.72419	-0.55588	-0.73459	-0.61142	-0.79663
ADH5	-0.48591	-0.83148	-0.99314	-0.6152	-0.73079
ADH6	-0.27198	-0.13991	-0.37821	-0.47611	-0.48889
ADH7	-0.33242	-0.28603	-0.37064	-0.5035	-0.49012
ADHFE1	-0.0027	0.390293	0.564992	0.157053	0.230708
ADI1	0.108332	0.157736	0.177445	-0.07285	0.256353
ADIG	0.401809	0.19845	0.473051	0.284692	0.353062
ADIPOQ	0.339183	-0.38758	0.580696	0.073259	0.347926
ADIPOR1	-0.26638	-0.34079	-0.22535	-0.39942	-0.31148

ADIPOR2	-0.04183	-0.10197	0.030641	-0.13412	0.025018
ADK	-0.42332	-0.21424	-0.60415	-0.5306	-0.60886
ADM	-0.25736	-0.39881	-0.15812	-0.12935	-0.05664
ADM2	0.186254	0.125242	0.103681	0.086407	0.133359
ADM2 MIOX	0.234404	0.468465	0.829544	0.508502	0.559172
ADNP	-0.27455	-0.36671	-0.29027	-0.38423	-0.35244
ADNP2	0.134471	0.180278	0.314183	0.098403	-0.09824
ADORA2B	0.217534	0.195637	0.406538	0.240491	0.188015
ADORA3	0.460391	0.394361	0.356337	0.218321	0.131006
ADPGK	0.04782	-0.04837	-0.12364	0.022231	0.050886
ADPRH	0.411193	0.766553	0.847194	0.516614	0.487403
ADRA1A	0.039976	0.59698	0.545376	0.481747	0.720248
ADRA1B	0.198271	0.316617	0.060685	0.232176	0.105369
ADRA1D	0.013272	-0.14624	0.115529	0.065247	0.150886
ADRA2A	-0.07359	-0.25743	-0.26737	-0.10162	-0.16116
ADRA2C	0.220102	0.216623	0.312944	0.310777	0.378424
ADRB1	-0.04402	-0.02839	0.011698	0.003751	0.214987
ADRB2	0.034266	-0.02695	-0.10297	0.332239	0.117101
ADRB3	0.110378	0.265042	0.514094	0.344585	0.316127
ADRBK1	0.172775	0.101307	0.403363	0.31262	0.279144
ADRBK2	-0.16432	-0.32749	-0.33186	-0.18688	-0.2741
ADRM1	-0.07377	-0.15592	-0.0004	-0.01926	-0.13778
ADSL	-0.01806	-0.2516	-0.26895	-0.26937	-0.16972
ADSS	0.098755	-0.0781	0.024548	-0.26816	-0.20228
AEBP1	0.086497	0.003279	-0.10307	-0.08291	0.169801
AEBP2	-0.03154	0.092783	0.140853	-0.31908	0.036248
AES	0.222077	0.383243	0.250465	0.262025	0.284728
AF003627.1	0.23345	0.20701	0.562533	0.112854	0.267747
AF196972.1	0.123213	-0.12678	-0.04376	-0.23163	-0.21911
AF196972.1 RBM3	0.20915	-0.1325	0.280125	0.044812	-0.39947
AF233439.5	-0.14826	-1.60747	-1.92639	-1.41466	-1.12799
AF235103.4 ZNF7	0.042665	-0.03539	0.052986	-0.13157	0.097156
AFAP1L1	0.115363	0.572114	0.490104	0.226969	0.179397
AFAP1L2	0.142089	0.524653	0.755231	0.462338	0.410338
AFDC1	-0.30124	-0.24644	-0.43619	-0.24108	-0.47129
AFF1	-0.62619	-0.31287	-0.37532	-0.26709	-0.51364
AFF2	0.172817	0.381114	0.280949	0.172995	0.286211
AFF4	-0.03892	0.008825	0.101598	-0.02563	0.019245
AFG3L1.	0.001621	-0.15417	-0.05471	0.213783	-0.13515
AFG3L2	-0.35287	-0.28865	-0.29803	-0.32744	-0.12022
AFM	-0.03616	-0.41457	-0.27864	-0.27196	-0.39907
AFP	-0.35605	-0.4105	-0.37649	-0.42003	-0.51292
AFTPH	-0.15401	-0.30459	-0.44103	-0.2002	0.074129
AGA	0.22877	-0.00999	-0.0965	0.191896	-0.03543
AGBL1	0.070454	0.318991	0.194901	-0.10725	-0.03844
AGBL2	0.656208	0.53432	0.711829	0.77234	0.770728
AGBL3	-0.01077	0.011094	-0.31051	0.040971	0.025548
AGBL5	-0.34476	-0.27096	-0.34966	-0.17224	-0.22633
AGER PBX2	0.284899	0.318479	0.232694	0.047483	0.231647
AGGF1	-0.21566	0.05833	-0.44496	-0.2675	-0.33036

AGMAT	0.24365	0.430964	0.464276	0.356571	0.355717
AGPAT1 RNF5	-1.18345	-1.00544	-1.08356	-1.05801	-1.26331
AGPAT2	0.009545	-0.10131	0.050849	0.013147	-0.05452
AGPAT4	-0.54661	0.375695	0.354677	-0.52607	-0.10557
AGPAT5	-0.24863	-0.38906	-0.24378	-0.26645	-0.25007
AGPAT6	0.12172	0.055128	-0.07825	-0.00265	0.151872
AGPAT7	0.046547	0.059143	0.119131	0.108923	0.088873
AGPAT9	-0.15842	-0.21688	-0.17575	0.17627	0.085061
AGR2	0.649611	0.73785	0.813654	0.461703	0.358706
AGR3	0.345331	-0.04022	0.11487	-0.08575	-0.24639
AGRN	-0.12926	-0.03712	-0.06491	-0.06443	-0.12709
AGT	0.376115	0.536013	0.440555	0.235877	0.41584
AGTPBP1	-0.30976	-0.23411	-0.2335	-0.20625	-0.20081
AGTR1	0.151017	0.423809	0.417568	0.051396	-0.00284
AGTR2	-0.19346	0.015859	-0.05848	0.075895	-0.05542
AGTRAP	-0.10317	-0.09309	-0.27259	0.050621	-0.07149
AGXT	0.305772	0.303557	0.56799	0.244487	0.238405
AGXT2	0.561935	-1.18241	0.639493	0.326948	-0.40339
AGXT2L1	0.306717	-0.01648	-0.00938	0.448148	0.257074
AGXT2L2	0.244302	0.294276	0.38426	0.123107	0.30093
AHCTF1P.	0.605005	0.794948	0.720402	0.452253	0.533704
AHCY	0.271414	0.488649	0.456745	0.308335	0.473512
AHCYL1	-0.12983	-0.21663	-0.4005	-0.36569	-0.12666
AHCYL2	-0.30898	-0.36479	-0.65321	-0.33896	-0.27469
AHDC1	-0.27109	-0.27598	-0.16288	-0.25853	-0.09341
AHI1	0.020689	-0.10927	-0.66346	-0.18255	-0.49236
AHNAK	-0.12431	0.225505	0.038792	0.254503	0.442735
AHR	0.077863	-0.08025	0.444138	0.168911	0.160508
AHSA2	0.164278	0.180869	0.263863	0.245508	0.193606
AHSG	0.076337	-0.56952	0.076882	-0.48979	-0.41802
AICDA	-0.15651	-0.86307	-0.64454	-0.71588	-0.61802
AIF1	0.122682	0.268439	0.3516	-0.06158	0.228596
AIFM1	0.12359	-0.23514	-0.57832	-0.25798	-0.25132
AIFM2	0.261576	0.449177	0.561388	0.289575	0.394282
AIFM3	0.007232	-0.08664	-0.37469	-0.18526	-0.1585
AIG1	-0.35454	-0.00059	-0.24134	-0.22973	-0.05041
AIM1	-0.53888	-0.45429	-0.43275	-0.10693	-0.10833
AIM1L AL451139.40	0.361154	0.152377	0.393204	0.225635	0.225208
AIM2	0.375206	-1.01332	-0.79414	-0.15004	0.193584
AIP	0.237467	0.098548	0.25953	-0.02826	0.261438
AIPL1	0.182554	0.259103	0.444324	0.269715	0.291854
AJAP1	0.011422	0.068157	0.049363	0.179734	0.117332
AK1	0.187769	0.429588	-0.43272	0.164315	-0.10567
AK2	0.338461	0.498011	0.497557	0.270327	0.301399
AK3	-0.06372	0.394637	-0.13303	0.123894	0.431617
AK3L2	0.014123	0.246568	0.294472	0.228806	0.325338
AK5	0.056354	0.145274	0.135008	-0.00076	0.193527
AK7	-0.0992	0.010287	-0.16575	0.010662	0.084393
AKAP1	-0.08713	-0.09975	-0.19312	-0.21359	-0.20993
AKAP10	0.335005	0.404498	0.296284	0.229104	0.332751

AKAP11	-0.22239	0.089994	-0.13058	-0.36799	-0.19948
AKAP14	-0.23921	-0.18464	-0.2961	-0.32618	-0.39722
AKAP3	0.092172	-0.38763	-0.39708	-0.52125	-0.37327
AKAP4	-0.2879	-0.22907	-0.56331	-0.36829	-0.23609
AKAP6	-0.21515	0.413532	-0.47612	-0.01953	-0.21356
AKAP8 AKAP8L	0.022062	-0.06387	-0.0461	0.08994	0.219108
AKAP8L	-0.27244	-0.17265	-0.18251	-0.242	-0.3329
AKAP9	-0.30721	-0.1709	-0.48426	-0.22794	-0.12425
AKNA	0.408143	0.17153	0.485339	0.358601	0.4309
AKR1A1	0.049419	0.06771	-0.16294	-0.15531	0.088314
AKR1B1	-0.10333	-0.23506	-0.44607	-0.26644	-0.16531
AKR1C2	-0.37766	-0.21581	-0.55898	-0.51705	-0.65281
AKR1C3	0.171333	0.361653	-0.29434	0.032355	-0.73127
AKR1C4	-0.01159	0.073379	-0.6165	-0.45834	-0.89505
AKR1CL1	-0.0116	0.351299	0.169568	0.030917	-0.36786
AKR1CL2	0.218869	0.160897	0.144426	0.178879	0.052731
AKR1D1	0.100141	0.056104	-0.3355	-0.48401	-0.11089
AKR7A2 PQLC2	0.017398	-0.04104	0.092467	0.100875	0.214623
AKR7A3	0.435529	0.589595	0.78671	0.402207	0.294834
AKT1	0.185566	0.189456	0.318007	0.096024	0.231208
AKT1S1	-0.4597	-0.40469	-0.5904	-0.38878	-0.59238
AKT2	-0.0841	-0.04752	-0.00376	-0.05282	-0.13165
AKT3	0.245264	0.664331	0.78537	0.17586	0.409993
AL020998.1	-0.08744	-0.03075	0.596029	0.037658	0.028776
AL021368.1	-0.13725	-0.4449	-0.72835	-0.19407	-0.51094
AL021453.1	-0.34018	-0.18767	-0.24684	-0.2808	-0.33482
AL021808.2	-0.41001	-0.78983	-0.51417	-0.5875	-0.79785
AL022320.23 CBY1	-0.17008	-0.1402	-0.09932	-0.26103	-0.33975
AL022328.21	0.043889	0.133912	0.151809	0.163025	0.159985
AL022341.6 C16orf14	-0.12542	-0.20801	-0.05727	-0.08495	-0.07405
AL023882.4	0.391992	0.223092	0.680114	0.364903	0.185981
AL031258.12	0.242935	-0.0111	0.271592	0.215475	0.233171
AL031282.1	-0.06485	-0.08137	-0.0901	0.060228	-0.10154
AL031289.1 NFYC	-0.09427	-0.12935	-0.37154	-0.40485	-0.27102
AL031705.25	0.45271	0.54729	0.756677	0.46883	0.582292
AL032819.32	0.284495	0.626314	0.600121	0.305408	0.528123
AL033383.26	0.05111	0.071818	-0.26607	0.120627	0.106188
AL034376.10	0.422205	-0.28092	0.318603	-0.10239	0.291854
AL035413.19	0.294086	0.595301	0.715853	0.302112	0.420233
AL035696.14	0.004566	-0.84349	-0.82205	-0.51486	-0.8491
AL049742.1	-0.16208	0.078877	-0.02294	-0.31122	-0.10147
AL049775.2 FLRT2	-1.13293	-1.30836	0.051046	-1.02682	0.004952
AL050312.8	0.105004	0.155686	0.031768	0.053684	0.139199
AL109758.3	0.218493	0.108841	0.183136	-0.00285	0.052118
AL109923.29 DYNLRB1	0.017879	-0.33864	-0.51423	0.179893	0.049115
AL109947.19	-0.08788	0.106161	0.04133	-0.20267	-0.18269
AL109948.9	-0.35815	-0.72183	-1.49757	-0.10916	-0.81863
AL117692.5	0.33903	0.11111	0.059026	0.292101	0.250339
AL121673.41	0.56519	0.390147	0.923073	0.137613	0.580748
AL121796.17	0.341285	0.051285	0.261391	0.263571	0.446103

AL121901.20	-0.5499	-0.89195	-0.87325	-0.72034	-0.64462
AL121918.26	-0.10669	-0.53379	-0.40912	-0.18408	-0.56937
AL121935.17	0.757301	0.481886	0.730121	0.478788	0.530328
AL132661.32	-0.63275	-0.36323	-0.54021	-0.13605	-0.53375
AL133216.10	-0.21949	-0.47419	-0.58736	-0.39326	-0.23124
AL133351.34	-0.1479	-0.09943	-0.11817	-0.12406	-0.22598
AL133375.25	0.111606	0.338286	0.353661	0.222242	0.291287
AL133476.17	0.675312	0.911262	0.945021	0.635648	0.64366
AL135841.11	0.42861	0.643541	0.781951	0.41902	0.582431
AL135932.7	-0.63988	-0.54347	-0.56142	-0.42442	-0.37918
AL135932.7 ZCSL3	-0.90995	-0.94211	-0.70361	-0.86352	-0.50029
AL135934.19	-0.21432	0.059362	-0.09374	-0.15994	0.054928
AL136170.14 ZBTB37	-0.6616	-0.98434	-1.05091	-0.84978	-0.66955
AL136218.26	-0.47057	-0.49657	-0.70836	-0.52143	-0.55022
AL136295.17	0.068304	0.162397	0.200764	0.072402	0.24879
AL137058.8	-0.37524	-0.8863	-1.0531	-0.38845	-0.90067
AL137229.4 KIAA0329	-0.41057	-0.42933	-0.55172	-0.3918	-0.35592
AL139021.4	0.232666	0.497223	0.26504	0.289122	0.430077
AL139099.3	-0.31097	-0.26202	-0.00072	-0.20381	-0.30225
AL139136.17 DENND1B	0.166847	0.306961	0.145644	-0.01734	0.09751
AL139188.14	0.286096	0.399317	0.65413	0.094802	0.387323
AL139275.30	-0.62871	-0.69956	-0.6612	-0.55565	-0.61229
AL157819.15	0.591889	0.377844	-1.5435	-1.16014	-1.16521
AL158055.12	0.502113	0.544593	0.69169	0.211156	0.529313
AL158205.12	0.261527	0.539391	0.637026	0.392079	0.242719
AL159167.23 AL159167.23	-0.06484	-0.13403	-0.33164	-0.71485	-0.33129
AL159169.14	0.000355	0.056042	0.174915	0.009408	0.061718
AL160279.21	0.267229	0.349721	0.344728	0.154061	0.193646
AL161668.6	-0.22437	-0.27093	-0.30274	-0.18418	-0.27589
AL162293.22	0.121196	0.450573	0.822015	0.470406	0.67817
AL162458.10 C20orf67	-0.14387	-0.25816	-0.41544	-0.2312	-0.24868
AL162595.17	0.181989	0.364576	0.243373	-0.06732	0.236534
AL353597.20	0.474189	0.547504	0.30133	0.340965	0.440211
AL353652.17	0.631274	0.828378	0.799103	0.831384	0.642641
AL353715.21	-0.1406	-0.01526	-0.11145	-0.15194	-0.16168
AL353732.14	-0.70395	-0.51613	-0.64128	-0.66488	-0.95649
AL353997.3	-1.62854	-0.97546	-1.55862	-1.34215	-0.55583
AL353997.8	0.412411	0.127095	0.301168	-0.10456	-0.22819
AL354776.15 RAB22A	-0.0652	0.24283	0.075687	0.075524	0.021719
AL354836.13	0.448695	0.359519	0.682928	0.341732	0.519534
\L354861.11 C9orf130. C9orf102	-0.22068	-0.28627	-0.17506	-0.23029	-0.14475
AL354889.14	0.357004	0.176184	0.560223	0.305839	0.533398
AL354951.7 ADD3	-0.18354	-0.11124	-0.25491	-0.03464	-0.36249
AL355075.6 PARP2	-0.49268	-0.40241	-0.33348	-0.22632	-0.04255
AL355149.13	0.168165	-0.04539	-0.30674	-0.57191	-0.62924
AL355336.15 RREB1	-0.39829	-0.35338	0.115092	-0.23265	-0.04038
AL355985.18	0.406706	0.406133	0.440001	0.193103	0.222716
AL356123.21	0.242517	0.746898	0.939363	0.579381	0.676072
AL356390.24	0.033941	-0.10868	-0.04974	0.072951	0.027473
AL356983.33	0.055984	0.159514	0.207595	0.013198	0.288348

AL357673.12 MRPL37	-0.23872	-0.21292	-0.203	-0.36538	-0.13817
AL357874.13 CCDC107	-0.09136	-0.05416	-0.01452	0.115959	-0.13682
AL358777.12 PAK1IP1	-1.01414	-0.78502	-1.18398	-0.63717	-1.08459
AL359712.12	0.101356	0.40542	0.091721	0.186058	0.024551
AL359736.19	-0.11947	-0.01931	-0.22541	0.023697	0.028335
AL359878.13 GTPBP4	-0.25717	-0.27612	-0.07772	-0.05416	-0.08956
AL360004.22	0.701554	0.752452	0.451651	0.874024	0.76136
AL365360.9 MLANA	0.516343	0.572376	0.760809	0.460652	0.204376
AL390294.19 GATA3	-0.23242	-0.43021	-0.50317	-0.52249	-0.49869
AL390719.47 TTLL10	0.196041	-0.09594	0.389251	0.164792	0.38557
AL391005.9 FOXI2	0.111059	0.283792	0.633988	0.435117	0.614546
AL391099.12	-0.06798	0.281252	0.141816	0.008667	0.173725
AL391137.11	0.46669	0.610634	0.406082	0.337971	0.431994
AL392172.9	0.053891	0.337532	0.427211	0.092802	0.315647
AL450334.15	-0.54352	-0.55353	-0.89943	-0.45899	-0.66204
AL450472.14	-0.07342	0.096295	0.234751	0.182493	0.140052
AL450998.19 SPEN	0.133834	-0.00155	-0.02884	0.106396	0.119902
AL451139.40	0.312851	0.302688	0.339572	0.064001	0.29258
AL512506.8	0.284177	0.441619	0.262513	0.360267	0.583716
AL512662.8	-0.63365	-0.70731	-0.56368	-0.65426	-0.64075
AL512791.3	0.001491	-0.22354	-0.17731	-0.10062	-0.06437
AL589743.4 AL589743.4	0.206454	0.330081	0.485623	0.26163	0.364353
AL589931.14	0.318742	0.316689	0.445499	0.25515	0.240739
AL590131.8	0.298232	0.50765	0.481401	0.383193	0.52738
AL590303.9 NUS1	0.112182	0.211962	0.021161	0.066047	-0.05389
AL590399.4	0.04164	-0.64701	-0.78554	-0.32464	-0.63742
AL591845.27	0.172042	0.017285	-0.02545	0.157208	0.267423
AL592464.24	0.257966	-0.04854	-0.22035	0.096439	0.1552
AL603840.9	0.313846	0.518258	0.609135	0.220611	0.464802
AL606514.3 COG3	-0.08295	0.023964	-0.05973	-0.08688	-0.10015
AL611925.27	0.092465	0.170266	0.079653	0.133646	0.170623
AL627230.15	0.700432	0.736955	0.733673	0.537126	0.81346
AL645568.9	0.336013	0.345741	-0.00111	-0.18251	-0.14395
AL645922.11	0.555402	-0.23323	0.824282	-0.05571	0.447418
AL645933.8	-0.21576	0.062799	0.177104	0.38552	0.355544
AL645939.6	-0.34863	-0.0151	-0.02195	0.388444	0.474768
AL645949.16	0.480823	0.509377	0.723119	0.431592	0.491057
AL662782.3	0.094975	0.054097	0.052556	0.182824	0.129582
AL662844.12	-0.13382	-0.45217	-0.42672	0.297472	-0.15416
AL671277.5 ZNRD1	-0.82944	-0.86598	-1.0423	-0.70581	-1.01152
AL671762.10	0.536933	0.592314	0.81036	0.55184	0.636523
AL671967.1	0.393004	0.526768	0.558109	0.283057	0.470567
AL713922.12	0.393375	0.357308	0.422021	0.306531	0.565755
AL928654.1	0.350313	-0.26301	0.496864	0.260221	-0.08299
AL928769.1	0.362984	0.489305	0.45148	0.259242	0.414268
AL953889.9	0.495385	0.500867	0.667631	0.719146	0.83449
ALAD	0.24036	0.301176	0.318911	0.192634	0.40454
ALAS1	0.323711	0.472179	0.3184	0.265603	0.310115
ALAS2	-0.49699	-0.06985	-0.46186	-0.54645	-0.33445
ALB	0.297121	-0.60834	-0.34217	0.107165	0.010313

ALCAM	-0.59151	-0.55737	-0.57756	-0.13239	-0.56909
ALDH18A1	-0.32068	-0.27339	-0.24757	-0.30245	-0.33483
ALDH1A1	0.2287	0.22596	-0.96775	0.059436	-1.32909
ALDH1A3	0.036484	0.077214	0.215503	0.159024	0.122674
ALDH1B1	0.002906	0.209578	-0.0141	-0.08694	0.034444
ALDH1L1	0.087265	0.373998	0.259502	0.107097	0.215545
ALDH1L2	0.179634	0.176622	0.275156	0.182884	0.177169
ALDH2	0.134925	0.236811	0.162103	0.069709	0.283256
ALDH3A1	0.289595	0.232999	0.403432	0.071546	0.272532
ALDH3A2	-0.14994	0.015903	-0.1371	-0.23632	0.054565
ALDH3B1	-0.17372	0.001365	0.028308	0.12292	-0.27319
ALDH3B2	0.364437	-0.02916	0.620114	-0.15976	0.435938
ALDH4A1	-0.23179	-0.21762	-0.24096	-0.11601	-0.04055
ALDH5A1	0.268341	0.602187	0.632542	0.331564	0.354369
ALDH6A1 LIN52	-0.78615	-0.72966	-1.18586	-0.88647	-0.95053
ALDH7A1	0.276696	-0.03139	0.518893	-0.13602	0.059711
ALDH8A1	0.319417	0.11578	0.2349	-0.16686	0.100553
ALDH9A1 AL451074.13	0.099355	0.034974	-0.0476	-0.03059	0.086252
ALDOB	-0.20268	0.052182	0.125498	-0.19534	-0.65041
ALDOC	0.143158	0.003586	-0.14915	0.150514	-0.13709
ALG1	0.229045	0.183708	0.070801	0.081404	-0.09562
ALG10	-1.37066	-1.85802	-1.99687	-1.88427	-2.02331
ALG10B	-1.09822	-1.48433	-1.66631	-1.22961	-1.35005
ALG12 CRELD2	0.119383	0.052495	0.179032	0.124662	0.144131
ALG14	-0.49708	-0.45443	-0.7872	-0.7891	-0.47936
ALG2 SEC61B	-0.24294	0.000156	-0.05202	-0.17753	-0.17202
ALG3 ECE2	-0.48387	-0.41033	-0.46958	-0.35898	-0.5763
ALG5 EXOSC8	-0.36107	-0.33963	-0.27188	-0.36616	-0.30711
ALG6	0.296417	0.300089	0.280228	0.233014	0.322723
ALG8	-0.05418	0.192271	-0.19926	-0.25479	0.03538
ALG9	0.027385	-0.11043	-0.39813	-0.25312	-0.04256
ALKBH1 C14orf156	-0.53397	-0.78481	-0.92189	-0.45621	-0.96351
ALKBH2	0.281188	-0.06245	-0.14945	-0.29626	-0.02609
ALKBH3	0.304608	0.01472	0.14957	-0.0002	0.171565
ALKBH4 LRWD1	-0.07399	0.088756	-0.25837	0.059325	-0.05529
ALKBH5	-0.40164	-0.02672	-0.23597	-0.25655	-0.08504
ALKBH6	0.150199	0.457849	0.286557	0.23673	0.359583
ALKBH6 CLIP3	-0.55982	-0.63054	-1.18383	-0.72582	-0.9229
ALKBH7	0.348008	0.369996	0.542088	0.349414	0.297262
ALKBH8	-0.2165	-0.01799	-0.30362	-0.26014	-0.5472
ALMS1	-0.14792	-0.1077	-0.14286	-0.04019	0.171566
ALOX12B	0.280946	0.028517	0.401946	0.085807	0.207898
ALOX15	-0.18281	-0.17439	0.630887	0.405876	0.371254
ALOX15B	0.120983	0.455133	0.606652	0.121646	0.291183
ALOX5	0.087946	0.164943	0.260732	0.273369	0.379917
ALOX5AP	0.098741	0.153516	-0.02746	-0.13577	-0.07954
ALOXE3	-0.59145	-0.26396	-0.57828	-0.38963	-0.17107
ALPI	0.210003	0.206099	0.467297	0.055353	0.086443
ALPK1	0.567166	0.948774	0.110421	0.682466	-0.09011
ALPK2	0.052251	0.423919	0.351481	0.065864	0.236583

ALPK3	0.401716	0.394671	0.591548	0.523365	0.538768
ALPL	-0.01978	0.245832	0.252883	0.10901	0.208886
ALPP	0.248488	0.293702	0.338353	0.038823	0.31178
ALPPL2	0.251233	0.376861	0.381326	0.12792	0.252937
ALS2	-0.41783	-0.61101	-1.23072	-0.81436	-1.06113
ALS2CR11	0.637338	0.455251	0.849755	0.376458	0.519715
ALS2CR12	0.485874	0.193466	0.604785	0.199178	0.492305
ALS2CR13	-0.06958	0.246515	0.17165	0.078452	0.492101
ALS2CR4	-0.43556	-0.2591	-0.43955	-0.48033	-0.42493
ALS2CR7	-0.0003	0.119122	0.203316	-0.33001	-0.44623
ALX1	-0.63652	-0.67142	0.172233	0.091436	-0.1302
ALX3	0.012137	0.077615	0.107482	0.126073	0.153976
ALX4	-0.23701	-0.05158	-0.09031	-0.08971	-0.05073
AMAC1	0.119067	0.320235	0.246011	0.247862	0.13023
AMAC1L2	0.670371	0.326401	0.559226	0.559331	0.661886
AMBN	0.403093	-0.92565	-0.77615	-0.57794	-0.38972
AMBP	0.295377	0.495367	0.575682	0.159284	0.358294
AMBRA1	0.711224	0.346399	0.832471	0.313384	0.378783
AMD1	-0.49887	-0.13833	-0.44826	-0.37389	-0.37896
AMELY	-0.08923	0.117051	0.131746	-0.15486	0.181746
AMFR	0.15183	0.205448	0.30012	0.095689	0.286307
AMHR2	-0.0101	-0.06882	0.177483	-0.08627	-0.05908
AMICA1	-0.5067	-0.52634	-0.95527	-0.65431	-0.86589
AMIGO1	-0.04979	-0.13641	-0.22282	0.019094	0.026143
AMIGO2 FAM113B	-0.33114	0.106364	0.39485	0.123043	0.529204
AMIGO3 GMPPB IHPK1	0.018639	-0.00651	-0.12388	0.091293	0.174627
AMMECR1L	-0.74221	-0.68358	-0.64533	-0.69314	-0.85483
AMN	0.129815	0.303178	0.690966	0.352732	0.403463
AMN1	-0.18737	0.127658	-0.11431	0.043658	-0.14113
AMOT	-0.14582	-0.51937	-0.03559	-0.41244	-0.70337
AMOTL1	-0.11604	-0.14243	-0.34065	-0.26307	-0.39027
AMOTL2	-0.38843	-0.34785	-0.33174	-0.0322	-0.35946
AMPD1	0.504127	-0.36524	0.682025	0.21629	0.109211
AMPH	-0.00785	0.135273	0.146111	-0.04419	0.164324
AMT NICN1	0.222737	0.180809	0.106855	0.224485	0.286862
AMTN	-0.49007	-0.73222	-0.7641	-0.58501	-0.61239
AMY2A	0.563416	0.531759	0.926138	0.493127	0.445686
AMY2B	-0.08309	-0.22356	-0.47867	-0.63194	-0.51109
AMZ1	0.504059	0.493484	0.392734	0.492614	0.446634
AMZ2	0.046348	0.107656	-0.01651	0.054474	0.109179
ANAPC1	-0.04503	0.066564	-0.13883	-0.2367	0.150041
ANAPC10P ABCE1	-0.34634	-0.64776	-0.66017	-0.46958	-0.72547
ANAPC13 CEP63	-0.20722	-0.26886	-0.32532	-0.27513	-0.26017
ANAPC2 SSNA1	-0.03511	-0.12874	0.03228	0.053913	0.008645
ANAPC4	0.108064	0.149494	0.312672	0.078238	0.083514
ANAPC5	-0.11468	-0.16879	-0.17192	-0.14838	-0.3175
ANAPC7	-0.23461	-0.37446	-0.60651	-0.46341	-0.57424
ANGEL1	0.035506	0.025532	0.194261	0.034549	-0.12038
ANGEL2	-0.13401	-0.11005	-0.00801	-0.09796	0.052557
ANGPT1	-0.54469	0.252328	-0.88022	0.362369	0.313398

ANGPT4	0.089915	-0.07256	0.537382	0.25303	0.015007
ANGPTL5 KIAA1377	-0.61264	-0.25235	-0.51112	-0.40752	-0.63482
ANGPTL6	0.247004	0.525135	0.208805	0.08908	0.144472
ANK2	0.066156	-0.08519	-1.3362	-0.94943	-1.28902
ANKAR	0.37453	0.628206	0.535776	0.231283	0.479152
ANKFN1	-0.1531	-0.76183	-1.83466	0.339524	-0.6816
ANKH	-0.13093	-0.25373	-0.29853	-0.23218	-0.22916
ANKK1	0.242824	0.34218	0.46457	0.318128	0.365408
ANKLE1	0.224012	-0.06324	-0.0333	0.282228	0.39642
ANKMY1	0.238956	0.288159	0.299614	0.03956	0.225228
ANKMY2 BZW2	-0.40335	-0.48795	-0.66209	-0.28534	-0.45112
ANKRA2 UTP15	-0.70648	-0.90056	-0.81082	-0.67986	-0.80011
ANKRD1	-0.53066	0.012957	-0.18598	-0.1129	0.029264
ANKRD10	-0.18163	-0.01099	-0.13051	-0.09849	-0.175
ANKRD11	-0.04347	0.11243	0.005853	0.068527	0.001921
ANKRD12	-0.19154	-0.29461	-0.17874	-0.11351	-0.02009
ANKRD13A	-0.15656	0.207381	0.146949	0.126913	0.208385
ANKRD13B	0.057261	0.145342	0.108004	0.042156	0.050523
ANKRD13C HHLA3	0.134925	0.002227	-0.0576	-0.08046	0.237904
ANKRD13D	0.027966	0.091769	-0.0247	-0.08498	-0.02383
ANKRD16 FBXO18	-0.15937	-0.11571	-0.02535	-0.08132	0.034593
ANKRD17	-0.32749	-0.42398	-0.36623	-0.34913	-0.38156
ANKRD18A	-1.13825	-1.15837	-1.43217	-1.26543	-1.37835
ANKRD18B.	-0.03021	-0.14392	-0.08609	0.147469	0.209972
ANKRD19	-0.19904	-0.11108	-0.00654	0.14425	0.193771
ANKRD2	0.239907	0.218417	0.285037	0.162327	0.391389
ANKRD2 C10orf65	0.129614	0.345484	0.194775	0.183105	0.261266
ANKRD20A1	0.239475	0.186647	0.35507	0.374956	0.494746
ANKRD20A2	0.250651	0.11756	0.273342	0.320637	0.409738
ANKRD20A3	0.227743	0.109108	0.324509	0.333916	0.31201
ANKRD21	0.537869	0.5651	0.637066	0.623792	0.714065
ANKRD23	0.263115	0.464576	0.415824	0.292998	0.318118
ANKRD26 AL162272.10	0.372474	0.129048	0.049128	-0.01883	-0.135
ANKRD27 RGS9BP	-0.12521	-0.1442	-0.1266	-0.07963	-0.03363
ANKRD28	-0.06295	0.206492	0.024588	-0.10633	0.085112
ANKRD29	0.219379	0.267853	0.19563	-0.02186	-0.11795
ANKRD30A	0.45044	0.660042	0.623342	0.414271	0.665335
ANKRD30B	0.386426	0.300515	0.334318	0.461936	0.461452
ANKRD33	0.195827	0.294218	0.210471	0.249436	0.241725
ANKRD34B	0.106689	0.289624	0.060949	0.184144	0.197955
ANKRD35	0.28712	-0.20451	-0.38827	-0.10299	-0.10537
ANKRD36	-0.04405	-0.12002	0.104639	-0.04204	0.117725
ANKRD36B	-0.30745	0.225565	0.058979	-0.25661	-0.31944
ANKRD37	0.110616	-0.00915	-0.15798	0.29183	-0.02138
ANKRD39	-0.06065	0.055387	0.019496	-0.01378	0.016887
ANKRD40	-0.42719	-0.56008	-0.81324	-0.68985	-0.56946
ANKRD42	-0.56194	-0.28091	-0.48111	-0.34649	-0.41311
ANKRD43	0.137489	0.450411	0.27062	0.132421	0.233946
ANKRD44	0.657114	0.63829	0.914075	0.375408	0.61657
ANKRD45	0.149035	-0.12192	-0.3689	-0.1577	-0.42927

ANKRD46	0.166903	0.0958	0.218457	0.128867	0.072988
ANKRD5	0.108642	-0.11311	0.04523	-0.06328	0.028144
ANKRD50	-0.16993	-0.6647	-0.3155	-0.34427	-0.03045
ANKRD53	0.261385	0.019196	0.670732	0.322271	0.387671
ANKRD54 hsa-mir-658	-0.05731	-0.37805	-0.60579	-0.21807	-0.33587
ANKRD55	0.391141	0.445485	0.451934	0.080425	0.316128
ANKRD56	0.105999	0.322333	0.265655	0.285988	0.329562
ANKRD6	0.112468	0.084224	-0.01739	0.013755	0.038974
ANKRD60	-0.04753	0.029433	-0.08043	0.241581	0.141435
ANKRD7	0.427346	0.584754	0.608617	0.404906	0.142105
ANKRD9	0.002163	0.031697	-0.03988	0.070378	0.221903
ANKS3 C16orf71	-0.18306	-0.15319	-0.29088	-0.13507	0.009998
ANKS4B	0.463545	0.609304	0.808569	-0.36233	-0.64744
ANKS6	0.40865	0.330177	0.465221	0.232413	0.341019
ANP32A	-0.33267	-0.43648	-0.60872	-0.3382	-0.34086
ANP32B	-0.02482	0.018421	-0.08801	0.018286	0.044616
ANP32C	0.52722	0.173057	0.608284	0.439448	0.453383
ANP32D	0.448106	0.711165	-0.89329	0.35846	0.161814
ANP32E	-0.53656	-0.4956	-1.0929	-0.82324	-0.92963
ANPEP	0.323057	0.47518	0.414514	0.387556	0.395905
ANTXR1	-0.18039	-0.22055	-0.26758	-1.02901	-0.11173
ANTXR2	-0.17941	-0.25477	0.068526	-0.23026	0.022271
ANUBL1	0.074593	0.252988	0.314953	0.168174	0.092734
ANXA1	-0.06916	-0.16997	-0.20691	-0.30653	-0.55856
ANXA10	-0.11365	-0.01101	0.0135	-0.16548	-0.11508
ANXA11	0.060281	-0.0947	-0.08747	0.061845	0.03978
ANXA13	0.542857	0.59218	0.653474	0.318408	0.447043
ANXA2	0.02782	0.044913	0.042629	0.062701	-0.37552
ANXA5	-0.20369	-0.07335	0.029318	-0.04646	0.02972
ANXA6	-0.16693	0.165808	-0.11932	-0.22173	-0.15198
ANXA7	-0.1685	-0.39185	-0.56265	-0.38148	-0.44781
ANXA8L2	0.22841	0.330278	0.208028	0.183096	0.355851
ANXA9	0.080132	0.215931	0.239514	-0.06019	0.086602
AOAH	0.300923	0.573914	0.665032	0.116686	0.199444
AOC2	0.35866	0.352365	0.177849	-0.0396	0.231763
AOC2 AOC3	0.491378	-0.16169	0.32005	-0.08337	-0.14491
AOF2	-0.04653	0.123601	-0.0416	0.169782	-0.20535
AOX1	0.286934	0.219387	0.476254	0.262909	0.160577
AP000346.1	0.222362	0.25986	0.333454	-0.07739	0.385453
AP000550.1	0.144557	0.087063	-0.00844	0.194856	-0.1523
AP000569.1	-0.08702	0.230077	0.319955	0.148361	0.23805
AP000744.4	0.180245	0.331447	0.437589	0.207684	0.271161
AP000751.4	-0.55398	-0.30813	-0.81541	-0.4352	-0.72955
AP000752.4	0.035466	0.194202	0.006711	-0.13386	0.112761
AP000753.4	0.131903	0.1217	0.264455	0.149127	0.272758
AP000775.4	0.400751	0.441465	0.408674	0.366179	0.089717
AP000807.4 SAPS3	-0.07311	-0.19733	-0.1848	-0.06062	-0.31994
AP000859.4	-0.02987	0.431543	-0.06596	0.102342	0.113435
AP000879.4 TMEM16A	-0.1196	0.181641	0.156055	0.09111	0.076727
AP000889.6	-0.33176	-0.33077	-0.48534	-0.2525	-0.25306

AP000920.4	0.433625	0.391987	0.748881	0.417415	0.675264
AP000926.6	0.21258	0.225571	0.110998	0.292854	0.349796
AP001004.5	0.158561	-0.01351	0.070538	0.244042	0.216352
AP001042.1	0.262891	0.58823	0.387858	0.039027	0.086522
AP001160.4 SLC3A2	-0.86316	-0.81221	-0.91028	-0.69732	-0.46576
AP001187.5	0.112499	-0.22068	-0.54828	-0.38259	-0.37088
AP001264.4	0.277394	0.35793	0.210886	0.04718	0.321379
AP001372.4 AP001372.4	0.267061	0.432775	0.50385	0.18255	0.226128
AP001458.6	0.087941	0.100853	-0.01362	0.091874	0.050032
AP001781.5 C11orf1	-0.13688	-0.27742	-1.01078	-0.34015	-0.32179
AP001888.4	0.442111	0.646137	0.558456	0.474245	0.475875
AP002407.3	-0.32645	-1.14801	-1.42478	-1.15278	-1.70442
AP002448.3 AP002448.3	0.220335	0.32549	0.388236	0.287197	0.367417
AP002490.4 RNF121	-0.87961	-0.48526	-0.77232	-0.72957	-0.79654
AP002796.3	0.265514	0.540539	0.337786	0.381341	0.391845
AP002965.3	0.047857	-0.52819	-0.78186	-0.5723	-0.718
AP003068.3	0.305721	0.353485	0.285721	0.269386	0.272156
AP003087.2	-0.03458	-1.20428	-1.80115	-1.03167	-1.74992
AP003108.3 C11orf79	-0.18162	-0.40847	-0.39066	-0.31912	-0.04844
AP003117.2	0.293611	0.248632	0.594944	0.257782	0.387975
AP003122.2	-0.26739	-0.67705	-0.79699	-0.59614	-0.77993
AP003181.2	0.074673	-0.0767	0.024508	-0.22232	-0.14942
AP003356.2	-0.07962	0.015121	0.02651	-0.04392	0.028773
AP003781.3 JRKL	-0.17277	-0.19754	-0.55844	-0.15846	-0.43375
AP005329.1	-0.10373	-0.53327	0.035551	0.137434	-0.00686
AP005329.2	0.02471	0.142572	0.138442	-0.0293	0.132652
AP006216.1	0.166108	0.202406	0.359654	0.160685	0.243289
AP006284.2	-0.37375	-0.38723	-0.48336	-0.25528	-0.16337
AP006284.2 DRD4	0.301961	0.353587	0.342033	0.388284	0.39746
AP006285.2	0.111568	-0.47146	0.236376	-0.08443	0.235288
AP006289.2	-0.0909	0.033729	-0.23393	-0.24082	0.024277
AP1B1	0.094993	0.147489	0.187147	0.098678	-0.13336
AP1G1	-0.21842	-0.06273	0.063148	0.02226	-0.15363
AP1G2 JPH4	-0.21773	-0.66761	-1.15903	-0.85851	-0.94161
AP1GBP1	-0.12662	-0.12085	-0.34322	-0.46963	-0.35202
AP1M1	-0.11804	-0.22346	-0.19797	-0.0224	-0.09722
AP1M2	-0.11303	0.01532	0.441916	0.208328	-0.06633
AP1S1	-0.10162	0.002689	-0.2456	-0.12822	-0.35217
AP1S2	-0.27325	-0.24879	-0.31696	-0.08822	-0.10848
AP1S3	0.19889	0.317472	0.40249	0.310793	0.223759
AP2A1	-1.05804	-0.78593	-0.79646	-0.64018	-0.93473
AP2A2	0.210946	0.100931	0.174729	0.138254	0.14901
AP2B1	0.111976	-0.60859	-0.8192	-0.58642	-0.78598
AP2M1	-0.1347	-0.05581	-0.1447	0.002636	-0.0784
AP2S1	-0.21018	-0.06946	0.0006	-0.1633	-0.11344
AP3B1	-0.83297	-0.79962	-0.95093	-0.63203	-0.93541
AP3B2	0.061825	0.334303	0.224538	0.149132	0.19454
AP3D1	0.016171	0.139629	0.081789	0.040994	0.125861
AP3M1 ADK	-0.51746	-0.59097	-0.45359	-0.46062	-0.7314
AP3M2	-0.125	-0.05976	0.110042	0.056651	0.112166

AP3S2	0.067034	-0.32834	-0.4769	-0.0908	-0.41291
AP4E1	-0.02393	-0.00636	0.089455	0.03924	0.074904
APBA1	0.095005	0.184078	0.105801	0.110198	0.178975
APBA2	0.605768	0.748735	0.754858	0.401565	0.473048
APBB1IP	0.323867	0.657274	0.24917	0.303695	0.286947
APBB2	0.026054	-0.15053	0.086494	-0.01034	-0.20685
APBB3 SLC35A4	-0.69395	-0.56631	-0.90592	-0.68733	-0.6483
APC	-0.38365	-0.16713	-0.38666	-0.42262	-0.46766
APC2	0.210917	0.124967	0.103952	0.104604	0.07597
APCDD1	-0.00739	0.060066	0.158192	-0.06033	0.13505
APCDD1L	-0.02173	0.405676	0.448196	0.372493	0.432564
APCS	0.115007	-0.97947	-0.75155	-0.81978	-0.46591
APEH	-0.11784	-0.00977	-0.1833	-0.18376	0.011024
APEX2	-0.03561	-0.28441	0.373156	-0.15933	-0.20533
APH1A C1orf54	-0.24763	0.166679	0.390401	-0.02539	0.134073
APH1B	0.132167	0.357822	0.084426	0.028368	0.315542
API5	0.012593	-0.44202	-0.49382	-0.26898	-0.28633
APIP PDHX	-0.4464	-0.35253	-0.41338	-0.51372	-0.27213
APLN	0.135317	0.079742	0.121115	0.115406	0.132456
APLNR	0.073362	0.455054	0.44382	0.09925	0.006357
APLP1	-0.48896	-0.4863	-0.48242	-0.40413	-0.44471
APLP2	-0.03209	0.066885	-0.11919	-0.01121	-0.10661
APOA1	0.158716	0.097318	0.178142	0.046187	0.15345
APOA1BP	0.245993	0.23765	0.301268	-0.02896	0.061728
APOA2 TOMM40L	0.096796	0.059835	-0.19977	-0.14779	-0.00526
APOA4	0.41329	0.351194	0.515494	0.028386	0.387134
APOA5	0.160048	0.275041	0.376255	0.114293	0.452915
APOB	0.008678	0.11738	0.159819	0.075292	0.139726
APOBEC1	0.159034	-0.55816	-0.32197	-0.40663	-0.26919
APOBEC2	-0.19138	-0.28491	-0.42501	-0.50997	-0.39123
APOBEC3A	0.420599	0.47347	0.508115	0.231881	0.433663
APOBEC3B	0.290402	-0.05761	0.110991	0.025868	0.148734
APOBEC3H	0.291204	0.176646	0.299757	0.083364	0.22932
APOC1	0.352914	0.239909	0.338083	0.386417	-0.01241
APOC3	0.281188	0.167462	0.225628	0.035818	0.283998
APOD	0.453417	0.65397	0.752225	0.421074	0.618664
APOE	-0.01362	-0.09642	0.106637	0.021726	-0.01015
APOF	0.56225	0.294774	0.505859	-0.7262	0.09085
APOH	0.456956	0.608192	0.607976	0.185954	0.197852
APOL1	0.182173	0.236805	0.340683	0.101167	0.349578
APOL2	0.121953	0.130468	0.410938	0.209841	0.414942
APOL5	0.215765	0.14631	0.047287	0.035298	-0.00855
APOL6	0.526473	0.418546	0.918524	0.426583	0.432906
APOLD1	0.330931	0.491927	0.523652	0.415679	0.582728
APOM	0.434219	0.349172	0.405572	-0.10192	0.270451
APOO CXorf58	0.111527	-0.07047	0.127834	-0.09625	-0.10112
APOOL	0.112791	-0.19925	-0.22225	-0.41766	-0.45779
APP	-0.16464	0.030962	-0.08844	-0.00612	-0.14684
APPBP1	0.00246	0.02198	-0.01165	-0.14999	-0.0612
APPBP2	-0.64074	-0.5954	-0.7657	-0.74176	-0.43529

APPL	-0.1417	-0.23287	-0.49206	-0.40216	-0.20612
APPL2	0.18192	0.267683	-0.02207	0.057019	-0.06324
APRT	-0.02867	0.150078	0.153872	0.073875	0.269995
AQP1	0.258612	0.227859	0.364323	0.153819	0.257334
AQP10	0.247684	0.315539	0.223853	0.078906	0.046449
AQP10 ATP8B2	-0.44075	-0.76003	-0.75437	-0.63162	-0.60858
AQP11	0.282348	0.217604	0.179948	0.173681	0.24104
AQP12A	0.170749	0.146604	0.146449	0.120117	0.004769
AQP2	0.404411	0.454257	0.397769	0.299684	0.320206
AQP3	-0.03647	-0.01788	-0.02231	0.05433	0.19622
AQP5	0.181998	0.188762	0.456928	0.201982	0.296657
AQP6	0.408448	0.600744	0.352341	0.448825	0.567997
AQP7	0.500832	-0.23346	0.191841	-0.06659	-0.04326
AQP8	0.427105	0.389263	0.480234	0.368402	0.359759
AQP9	0.214171	0.373738	0.535222	-0.14445	0.399708
AQR	-0.26148	-0.33262	-0.64633	-0.2567	-0.68664
ARAF	0.220051	0.258173	0.357058	0.24109	0.240098
ARC	0.260966	0.033028	0.194289	0.214288	0.207319
ARCN1	0.04118	0.339849	-0.16709	0.012575	-0.18667
ARD1A RENBP	-0.01741	0.014453	0.079372	0.187132	0.068818
ARF1	-0.11643	-0.31539	-0.15354	-0.2422	-0.14103
ARF3	-0.35607	-0.20967	-0.33303	-0.57109	-0.57914
ARF4	-0.54503	-0.0048	-0.44721	-0.22582	0.076832
ARF5	0.108873	0.300086	0.422559	0.338783	0.276227
ARF6	-0.15898	-0.11529	-0.17382	-0.12398	-0.04137
ARFGAP2	-0.35815	-0.47516	-0.4699	-0.30323	-0.36729
ARFGAP3	0.010856	0.26135	0.413709	0.190303	0.249726
ARFGEF1	-0.42271	-0.33766	-0.40736	-0.40275	-0.41662
ARFGEF2	-0.23137	-0.35209	-0.43155	-0.44838	-0.1836
ARFIP2 FXC1	-0.23881	-0.4791	-0.61655	-0.23642	-0.08306
ARFRP1 ZGPAT	0.176071	0.211811	0.2108	0.197506	-0.01476
ARG1	0.188743	0.237924	0.383928	0.049101	0.292883
ARG2	0.189954	-0.07	-0.10979	-0.16759	-0.25191
ARGLU1	0.003969	0.091694	-0.01592	-0.13449	0.048822
ARHGAP1 ZNF408	-0.10861	-0.20646	-0.56041	-0.20647	-0.26365
ARHGAP10	-0.06204	0.120559	0.11368	-0.01646	0.138062
ARHGAP11A	-1.31038	-1.39224	-1.61779	-1.04044	-1.2246
ARHGAP11B	-1.71349	-1.6881	-1.95243	-1.14653	-1.85664
ARHGAP12	-0.42722	-0.36256	-0.35802	-0.30766	-0.2036
ARHGAP15	0.327983	0.599664	0.484563	0.226981	0.075403
ARHGAP17	0.101261	-0.05784	0.08216	0.000916	0.101192
ARHGAP18	0.638536	0.462092	-0.0926	-0.22414	0.558948
ARHGAP19	-0.40822	-0.23325	-0.59563	-0.56918	-0.46747
ARHGAP20	0.044438	0.249073	0.109846	-0.01064	0.090859
ARHGAP21	-0.08072	-0.05921	0.086349	-0.08199	-0.2048
ARHGAP22	-0.13871	-0.18354	-0.0377	0.331206	0.159823
ARHGAP26	-0.11044	-0.143	-0.2698	-0.21068	-0.06899
ARHGAP27	0.049385	0.270955	0.187276	0.237739	0.124991
ARHGAP28	-0.25633	-0.08419	-0.43972	-0.50295	-0.36525
ARHGAP29	0.012865	0.045755	0.163316	-0.02995	0.2057

ARHGAP30	0.190796	0.212756	0.308593	0.015317	0.188589
ARHGAP4	-0.0041	-0.00568	0.27333	0.104618	0.050301
ARHGAP5	-0.67886	-0.64827	-0.64215	-0.53408	-0.52876
ARHGAP9 MARS	0.083694	-0.112	-0.14258	-0.05728	-0.16231
ARHGDIA	0.04021	-0.0605	0.0501	-0.00043	0.250807
ARHGDIB	0.717257	0.568781	1.028201	0.661928	0.859151
ARHGDIG	0.140591	0.306006	0.163159	0.193916	0.176592
ARHGEF1	-0.23665	-0.15693	-0.18488	0.002665	-0.31724
ARHGEF10	-0.08771	0.000852	-0.0168	0.031923	-0.02953
ARHGEF11	-0.27713	-0.35989	-0.34399	-0.36468	-0.29634
ARHGEF12	-0.48585	-0.3614	-0.3282	-0.11202	-0.39974
ARHGEF15	0.128772	-0.25949	-0.06464	-0.04934	-0.34949
ARHGEF16	0.220409	0.066274	0.381986	0.121347	0.203646
ARHGEF17	0.202009	0.036434	0.364526	0.207521	0.199444
ARHGEF18	0.30145	-0.1186	0.210765	0.011949	0.140756
ARHGEF19	-0.08097	-0.05822	-0.08522	-0.00192	-0.16812
ARHGEF2	-0.6802	-0.74436	-0.85271	-0.6359	-0.47958
ARHGEF3	0.251244	0.26512	-0.11211	-0.24836	-0.19778
ARHGEF4	0.240914	0.538505	0.527646	0.361353	0.443367
ARHGEF6	0.015708	-0.01727	-0.15216	0.156049	-0.12856
ARHGEF9	-0.23852	-0.70968	-0.03389	-0.57468	-0.33485
ARID1A	-0.01542	-0.03721	0.18629	5.57E-05	0.059129
ARID1B	0.097943	0.143851	0.089258	0.081063	0.169305
ARID2	-0.59682	-0.62751	-0.46958	-0.48594	-0.4623
ARID3A	0.203488	0.493273	0.467004	0.332203	0.383047
ARID3B	-0.54807	-0.36399	-0.41861	-0.38084	-0.54634
ARID3C	-0.20227	-0.04548	0.16546	-0.0406	-0.10983
ARID4A	-0.81948	-0.75717	-0.72853	-0.65072	-0.6021
ARID4B GGPS1	-0.88418	-0.96143	-1.04778	-0.9869	-0.83408
ARID5A	0.011843	0.311382	0.286337	0.087352	0.154756
ARID5B	-1.10549	-1.33713	-1.17166	-0.9942	-1.32164
ARIH1	-0.24905	-0.07566	-0.345	-0.30999	-0.27022
ARL1	-0.17939	-0.14714	-0.4786	-0.26733	-0.45629
ARL10	0.181851	0.339009	0.044551	0.202887	0.193379
ARL11	0.444406	0.602319	0.871461	0.328033	0.473579
ARL13A	-0.16642	0.136122	-0.09981	-0.08077	-0.01757
ARL14	0.419882	0.547696	0.477279	0.101471	0.27382
ARL15.	-0.83243	-0.8484	-0.7136	-0.6256	-0.73076
ARL16 HGS	0.028946	-0.16478	-0.07454	0.014374	-0.05925
ARL2	-0.05317	0.125522	0.004975	-0.01614	0.149074
ARL2BP	0.095184	0.172935	0.264274	0.069448	0.19843
ARL3 SFXN2	-0.41004	-0.35934	-0.24803	-0.24246	-0.26049
ARL4A	0.214835	0.012988	0.290441	0.330307	0.062617
ARL4C	0.149754	0.144456	0.072584	0.246083	0.173234
ARL4D	-0.12004	-0.13286	-0.15218	-0.19447	-0.30795
ARL5A	-0.03289	-0.01492	-0.07997	-0.22121	0.017401
ARL5B	-0.37267	-0.51914	-0.30344	-0.14881	-0.231
ARL6	-0.50695	-0.04475	-0.31657	0.012357	-0.29471
ARL6IP1	-0.09341	0.002567	-0.10548	-0.06218	-0.16429
ARL6IP2	-0.2358	-0.29922	-0.35031	-0.27618	-0.26127

ARL6IP5	-0.78778	-0.90147	-0.84334	-0.65061	-0.78252
ARL6IP6	-0.93835	-0.38562	-0.91455	-0.79931	-0.32746
ARL8A	-0.03698	-0.12629	-0.25874	-0.17226	0.061734
ARL8B	-0.12097	0.13275	0.094565	-0.19897	-0.03027
ARL9	0.39456	0.714669	0.815953	0.385003	0.413041
ARMC1	-0.25144	-0.1373	-0.49192	-0.39011	-0.60654
ARMC2	0.177348	0.400152	-0.32321	0.183648	0.348389
ARMC3	0.705141	0.689845	0.795771	0.585276	0.31503
ARMC4	0.218757	0.275751	0.204187	0.350058	0.182814
ARMC5	-0.05401	-0.11569	-0.06782	0.245951	-0.02212
ARMC7	0.215393	0.206062	0.131383	0.352563	0.044864
ARMC8	-0.43153	-0.50863	-0.68401	-0.53172	-0.6094
ARMC9	-0.01951	-0.07842	-0.06681	-0.05225	-0.04761
ARMCX1	0.170201	-0.13506	-0.29344	-0.29418	-0.31374
ARMCX2	0.237272	0.319358	-0.80038	-0.07915	0.484251
ARMCX3	-0.16206	-0.33716	-0.53826	-0.34969	-0.36777
ARMCX4.	0.209594	-0.1313	-0.15124	-0.17787	-0.18453
ARMCX5	0.185827	-0.09597	0.039662	-0.09963	-0.08537
ARMCX6	-0.03581	-0.16019	-0.20154	-0.15715	-0.09764
ARMET	-0.60715	-0.38064	-0.72497	-0.41148	-0.34014
ARMETL1 HSPA14	0.032298	0.04369	-0.05812	0.036166	0.035126
ARNT	-0.00303	-0.1776	-0.34465	0.003518	-0.16028
ARNT2	0.017843	0.216455	0.084727	0.069106	0.136957
ARNTL	-0.47029	-0.34918	-0.47135	-0.19674	-0.12662
ARNTL2	-0.0382	0.322192	0.30433	0.307402	0.253913
ARPC1A	-0.14785	-0.21325	-0.23557	-0.07281	-0.10424
ARPC1B	0.083105	0.178739	0.02947	0.192365	0.211663
ARPC2	-0.11334	0.228939	0.094328	0.089799	0.017812
ARPC3	0.119025	0.277755	0.080866	0.025156	-0.00494
ARPC5 RGL1	-0.60637	-0.93087	-1.04691	-0.74822	-0.69254
ARPC5L	-0.10016	-0.12856	-0.21923	-0.03965	-0.04321
ARR3	-1.08738	-0.39256	-1.36951	-0.67176	-1.19486
ARR3 RAB41	0.37382	0.50368	0.461948	0.301203	0.409226
ARRB1	0.078972	-0.01683	-0.2041	0.318395	-0.2256
ARRB2	-0.40525	-0.19811	-0.80429	-0.38256	-0.2979
ARRDC1	-0.14355	-0.05376	0.034112	0.029459	-0.06498
ARRDC3	-0.47972	-0.36338	-0.44025	-0.33597	-0.3344
ARRDC4	0.187147	0.054122	-0.08541	0.004457	0.169571
ARSA	0.042447	0.125955	0.119832	0.067811	0.045207
ARSB	-0.01874	0.256409	-0.20804	-0.02545	0.410788
ARSD	0.099002	0.184565	0.134336	0.244314	0.016234
ARSE	0.068553	-0.14444	0.217232	0.460265	0.332069
ARSF	0.441766	0.008651	-0.19045	-0.05864	-0.1425
ARSG	0.591218	0.650271	0.677468	0.389226	0.650901
ARSH	0.220554	0.561363	0.105278	0.287448	-0.03912
ARSI	0.091361	0.387867	0.476708	0.289176	0.37809
ARSJ	-0.56804	-1.08345	-0.37039	-0.20008	0.044676
ART1	0.313833	0.240545	0.414729	0.221812	0.275015
ART3	0.21215	0.392799	0.451453	-0.19901	0.197393
ART4	0.3925	0.364718	0.368674	0.303295	0.262741

ART5	-0.11953	0.068624	0.00184	-0.00563	0.222861
ARTN	0.158418	0.30905	0.196267	0.125698	0.166799
ARTS-1	-0.03716	-0.11344	-0.07712	-0.13626	0.032238
ARVCF C22orf25	0.04449	0.040579	0.141228	0.175708	0.0303
ARX	0.361736	0.123444	0.477037	0.488506	0.732676
ASAH1	-0.1604	-0.2713	-0.58161	-0.36671	-0.26502
ASAH2	-0.35476	-0.61706	-0.33984	-1.55685	-1.76443
ASAH3	0.163986	-0.17557	0.143522	0.18328	0.373968
ASAH3L	0.214306	0.203557	0.192483	0.288474	0.187535
ASB1	-0.11993	0.014432	-0.09782	-0.19356	0.022197
ASB10	0.179611	0.445743	0.326899	0.212589	0.478435
ASB11	0.10663	0.193763	-0.11291	-0.07879	0.047149
ASB12	0.09837	-0.02133	-0.39854	-0.0624	-0.28032
ASB13	0.243416	0.43852	0.275962	0.126911	0.263536
ASB14	0.358366	0.446105	0.599251	0.194542	0.264145
ASB15	0.063871	0.139618	0.179636	-0.22215	-0.22246
ASB16	0.181112	0.116467	0.26464	0.145386	0.090462
ASB17	0.35718	0.433925	0.390107	-0.10412	0.237339
ASB2	0.049651	0.177036	0.177722	0.028131	0.265417
ASB4	0.465794	0.732017	0.766012	-0.28275	0.493139
ASB5	0.537186	0.649972	0.774613	0.31748	0.519134
ASB6	0.100334	0.120798	0.017995	0.010105	-0.03784
ASB8	-0.54926	-0.50573	-0.61704	-0.64645	-0.6682
ASB9	0.308161	0.199097	0.096988	0.226626	0.314522
ASCC1 C10orf104	0.371075	0.064376	0.285534	0.158054	0.13826
ASCC2	-0.06114	-0.15053	-0.2857	-0.20667	-0.36812
ASCC3	0.03255	-0.46616	-0.33701	-0.43898	-0.45681
ASCC3L1	-0.22269	-0.20446	-0.3542	-0.16771	-0.28742
ASCL1	-0.63038	-0.69591	-0.15854	-0.1192	-0.46357
ASCL2	-0.0404	0.364472	0.444368	0.194267	0.386456
ASCL3	0.447744	0.444868	0.619414	0.125695	0.398213
ASCL4	0.702481	-0.39997	0.685919	0.186794	-0.21187
ASF1B	-0.05549	-0.18935	-0.3172	-0.17997	-0.13067
ASGR1	0.192577	0.175676	0.310199	0.229933	0.113764
ASGR2	0.327711	-0.04261	-0.04855	0.167515	0.369673
ASH1L	-0.38194	-0.55893	-0.65897	-0.42613	-0.46327
ASH2L	-0.00426	-0.05025	-0.05964	-0.11789	-0.10525
ASIP	-0.02247	-0.08583	-0.21466	-0.36213	-0.19994
ASL	0.104143	0.015113	-0.0886	0.047141	0.084026
ASMTL	-0.56171	-0.4339	-0.40913	-0.25264	-0.43897
ASNA1	-0.51656	-0.50947	-0.782	-0.3496	-0.60394
ASNS	0.163212	0.122279	-0.25953	-0.00862	-0.11978
ASNSD1	-0.77591	-0.63996	-1.08846	-0.86945	-1.48925
ASPA	-0.00021	-0.41215	-0.73949	-0.65038	-0.19648
ASPHD2	-0.00367	0.254987	-0.00435	0.238781	-0.0844
ASPM	-0.06534	-0.27615	-0.49389	-0.34195	-0.23944
ASPRV1	0.360103	0.273064	0.297449	0.205014	0.27854
ASPSCR1	0.05316	0.070037	-0.01882	0.058512	0.117877
ASRGL1	0.174999	0.125147	-0.00724	0.167088	0.125962
ASS1	-0.0318	0.058761	0.530645	0.010457	0.02159

ASTE1	0.520437	0.133531	0.044792	0.31563	0.30266
ASTL	-0.4819	0.033842	0.306367	0.20864	0.322955
ASTN1	-0.36867	-0.77015	-0.64859	-0.60328	-0.55697
ASXL1	-0.47891	-0.58867	-0.57164	-0.42804	-0.54291
ASXL2	-0.48519	-0.21833	-0.4444	-0.18494	-0.17792
ASZ1	0.560906	0.512944	0.378657	0.310042	0.61256
ATAD2	-0.22261	-0.00796	0.015184	-0.13074	-0.08016
ATAD3A	-0.08665	0.05092	-0.02484	0.155619	0.094136
ATAD3B	0.00011	0.00489	-0.03956	0.101314	-0.07698
ATAD3C	0.625984	0.386349	0.68449	0.329576	0.493006
ATAD4	-0.03984	0.344279	0.401054	0.195473	0.312878
ATAD5	0.067594	0.296722	0.1392	0.188302	0.253479
ATBF1	0.034385	0.173162	-0.0494	0.182919	-0.14369
ATCAY	-0.16035	-0.1044	-0.20625	-0.1453	-0.09265
ATE1	0.048931	-0.08921	-0.18557	-0.05185	0.088203
ATF1	-0.27869	-0.10657	-0.12184	-0.09663	0.069695
ATF2	-1.05912	-0.98666	-1.20175	-0.93521	-0.62556
ATF4	-0.35395	-0.18791	-0.11091	-0.32722	-0.28471
ATF6	-0.03742	-0.79979	-0.94594	-0.57162	-0.49378
ATF7	-0.07576	-0.32547	-0.61782	-0.42997	-0.53519
ATF7IP	0.126255	0.104345	0.044125	0.547606	-0.14522
ATF7IP2	0.044854	-0.63542	-0.01606	-0.43511	-0.19324
ATG10	-0.42572	-0.11565	-0.40893	-0.18846	-0.4139
ATG12 AP3S1	-0.40221	-0.19222	-0.30477	-0.2682	-0.44678
ATG16L1	0.165352	0.20775	0.177004	0.068742	0.038478
ATG16L2	0.035758	0.037151	-0.08613	-0.03525	0.065919
ATG2A	-0.36956	-0.18789	-0.40656	-0.24524	-0.11719
ATG3 SLC35A5	-0.61635	-0.48815	-0.49588	-0.42282	-0.53678
ATG4C	-0.27961	-0.42537	-0.54477	-0.24612	-0.36805
ATG4D	0.141777	0.021495	0.04465	-0.0707	-0.14475
ATG5	0.059782	0.336383	0.314856	0.090823	-0.02372
ATG9A ANKZF1	-0.14699	-0.05306	-0.19046	-0.14342	-0.09826
ATG9B	0.280104	0.153957	0.301219	0.026219	0.053327
ATHL1	0.318804	-0.02109	0.567417	0.334107	0.41662
ATIC	0.134071	-0.01262	0.103265	0.138654	0.000951
ATM	0.511597	0.54525	0.646765	0.179852	0.39051
ATMIN	0.446668	0.259149	0.521793	0.293195	0.443972
ATOH1	-0.6734	-0.56354	-0.50054	-0.3551	-0.38984
ATOH7	0.454888	0.794183	0.817751	0.362727	0.60718
ATOH8	0.017163	0.084342	0.308308	-0.06927	0.331664
ATOX1	0.002494	0.359942	0.208673	0.124992	0.038181
ATP10A	0.343886	0.421645	0.792375	0.517754	0.6136
ATP10D	-0.12346	-0.01748	-0.32822	-0.1776	-0.18418
ATP11A	-0.18675	-0.07262	0.078192	0.101963	0.063204
ATP11B	-0.10548	-0.1483	-0.1507	-0.19721	-0.18536
ATP11C	-0.51095	-0.14772	-0.62958	-0.15175	-0.39054
ATP12A	0.117026	0.408699	0.2277	0.293331	0.255257
ATP13A1	0.299615	-0.25488	0.294927	0.387531	0.404583
ATP13A2	-0.05337	-0.01642	-0.03854	0.13545	0.084972
ATP13A4	0.355038	0.267806	0.56632	0.226007	-0.00438

ATP13A5	0.221334	-1.36126	-1.21043	-0.41597	-1.3441
ATP1A1	-0.00553	0.273704	0.311798	0.031892	0.133391
ATP1A2	0.136763	0.193337	0.447329	-0.1334	-0.32837
ATP1A3	0.05919	0.056458	-0.20725	-0.13984	0.027613
ATP1B1	-0.10094	-0.01955	-0.0613	-0.13451	-0.05652
ATP1B2	0.034311	-0.03618	-0.06077	-0.11057	-0.0329
ATP1B3	-0.12703	-0.00555	-0.10926	0.041063	-0.13255
ATP1B4	-0.00609	0.203248	-0.39394	0.188645	0.127718
ATP2A1	0.380148	0.155948	0.320205	0.315037	0.436336
ATP2A2	-0.17382	-0.184	-0.10358	-0.14413	-0.12175
ATP2A3	0.000552	0.180006	0.374659	0.136739	0.061225
ATP2B1	-0.0126	0.240154	0.137837	-0.23315	-0.16604
ATP2B2	0.20981	0.089425	0.277175	0.261353	0.209812
ATP2B3	0.196152	0.086956	-0.21642	0.194506	0.233405
ATP2B4	-0.5234	-0.81477	-0.62855	-0.53781	-0.83664
ATP2C1	-0.2238	-0.29679	-0.26162	-0.39386	-0.23151
ATP2C2	0.33533	0.17761	-0.13258	-0.02344	-0.33335
ATP4A	0.167506	-0.6372	-0.36138	0.108704	0.1346
ATP4B	0.162257	0.024149	0.547333	0.084141	0.280792
ATP5A1	0.015406	0.123592	0.285002	-0.06517	0.16464
ATP5A1 CCDC5	-0.01862	0.05545	-0.24022	-0.01913	0.054051
ATP5B	-0.17612	0.085573	0.021868	-0.06707	-0.24467
ATP5D	0.105763	0.214257	0.053914	0.062686	0.233002
ATP5E	-0.07418	-0.11122	-0.0349	-0.06039	0.057411
ATP5G1	-0.53057	-1.07828	-1.34665	-0.48454	-0.58925
ATP5G2	0.129682	0.445597	0.383508	0.206763	0.320096
ATP5G3	-0.12105	-0.31699	-0.53818	-0.29447	-0.08433
ATP5H KCTD2	-0.18757	-0.29874	-0.03494	-0.07147	-0.05327
ATP5I	0.041345	-0.10073	0.003621	0.027517	0.11469
ATP5J GABPA	-0.38782	-0.42222	-0.47377	-0.35635	-0.38782
ATP5J2	0.239068	0.237489	0.06506	-0.02206	-0.07633
ATP5L	-0.67164	-0.47955	-1.13241	-0.68737	-0.91886
ATP5O	-0.04293	-0.27338	-0.2198	-0.10094	-0.08062
ATP5SL	0.07172	-0.13811	-0.06985	-0.13464	-0.05602
ATP6AP1 GDI1	0.125055	-0.01503	0.288701	0.087509	0.274629
ATP6AP2	0.20936	0.166169	-0.06424	-0.02036	0.063823
ATP6V0A1	-0.13845	-0.37472	-0.38284	-0.38522	-0.43802
ATP6V0A2	0.014254	0.02736	0.133265	0.053117	0.159969
ATP6V0A4	0.283797	0.471316	0.617223	0.246039	0.47246
ATP6V0A4 AC018663.3	0.101625	0.274	0.440944	0.232355	0.349768
ATP6V0B	-0.03557	-0.06639	0.11767	0.049204	0.195672
ATP6V0C	0.08077	0.192609	0.082466	0.079464	0.202668
ATP6V0C AMDHD2	0.359047	0.294954	0.284435	0.344814	0.183118
ATP6V0D1 AGRP	0.009528	-0.00666	0.050634	0.081726	0.067033
ATP6V0D2	0.452791	-0.08546	0.195313	-0.13912	0.423275
ATP6V0E	-0.0914	-0.05095	-0.23906	-0.10856	-0.14907
ATP6V1B1	-0.27557	-0.17352	-0.01172	-0.48601	0.024304
ATP6V1B2	0.042027	0.138916	0.217039	-0.15342	0.039142
ATP6V1C1	0.175341	-0.10211	-0.63769	-0.57494	-0.63147
ATP6V1C2	-0.08127	0.073565	0.042246	0.198504	0.182812

ATP6V1D EIF2S1	-0.67638	-0.42802	-0.61846	-0.46999	-0.47921
ATP6V1E1 BCL2L13	-0.36371	-0.2146	-0.51132	-0.41312	-0.58978
ATP6V1E2	0.323012	0.683055	0.504131	0.296612	0.261159
ATP6V1E2 RHOQ	-0.20911	-0.0791	-0.31375	-0.18221	-0.12615
ATP6V1G1	-0.20277	-0.10776	-0.36376	-0.14324	-0.49284
ATP6V1G2 NFKBIL1	-0.53767	-0.81838	-0.83777	-0.60192	-0.85389
ATP6V1G3	0.062684	0.053885	-0.11204	-0.33446	0.016187
ATP6V1H	0.063003	0.231106	0.089574	-0.02397	0.014111
ATP7B UTP14C	-0.30294	-0.19921	-0.23107	-0.23667	-0.27252
ATP8A1	-0.33174	-0.37041	-0.48665	-0.25501	-0.40057
ATP8A2	0.081341	0.478038	0.505658	0.281214	0.470468
ATP8B1	0.553495	0.535997	0.789078	0.474489	0.365662
ATP8B3	-0.37407	-0.37681	-0.75219	-0.21352	-0.45374
ATP8B4	-0.89317	-0.65794	-1.25497	-1.05832	-1.5096
ATP9A	0.207387	0.142995	0.252619	0.069583	0.075907
ATP9B	0.231721	0.230726	0.406428	0.285726	0.190405
ATPAF1	0.112585	-0.14809	-0.2376	0.309989	0.236188
ATPAF2 C17orf39	-0.36733	-0.15259	-0.41017	-0.39609	-0.10688
ATPBD1B GPATCH3	0.314423	0.06282	-0.15517	0.22311	0.135104
ATPBD3	0.292453	0.510772	0.489074	0.292533	0.545784
ATPBD4	0.331439	0.091338	0.165757	0.257035	0.102761
ATPIF1	-0.1635	-0.05603	-0.46572	-0.11006	-0.19944
ATR	-0.08902	-0.14346	-0.46359	-0.32632	-0.26462
ATRN	0.203327	0.103689	0.15226	0.073313	0.174764
ATRNL1	-0.3406	-0.25195	-0.30085	-0.23228	-0.18923
ATRX	0.334654	-0.31746	-0.99976	-0.34272	-0.53482
ATXN1	-0.6705	-0.64537	-0.66551	-0.60115	-0.71085
ATXN10	-0.11092	-0.02505	-0.28987	-0.18826	-0.25419
ATXN2	-0.20179	-0.37455	-0.25052	-0.10656	-0.27908
ATXN2L	0.076623	-0.11542	-0.12219	-0.04	-0.06843
ATXN3	-0.05602	0.053081	-0.22612	-0.20244	-0.09858
ATXN7L1	-0.17327	-0.23953	-0.38329	-0.26298	-0.49219
ATXN7L2	-0.32958	-0.28693	-0.32268	-0.54038	-0.3447
AUH	0.401687	0.364253	0.534448	0.38553	0.30997
AUP1 HTRA2	-0.04034	-0.2683	-0.18028	-0.10761	-0.01554
AURKA CSTF1	-0.66489	-0.75013	-1.12339	-0.7459	-0.96186
AURKAIP1	-0.07043	0.06029	0.052724	0.083361	0.041057
AURKB	0.141224	0.145967	-0.30294	-0.06012	-0.11539
AURKC	0.310556	0.571226	0.548233	0.340664	0.619014
AUTS2	-0.80559	-0.68173	-0.85447	-0.63537	-0.57591
AVEN CHRM5	-0.07515	0.085592	-0.03712	-0.06548	0.026118
AVIL	0.162528	-0.25534	-0.4416	-0.50459	-0.78889
AVP	0.315213	0.207062	0.517457	0.23385	0.358166
AVPI1	0.28246	0.250953	0.357114	0.160513	0.285117
AVPR1A	-0.58426	-0.65991	-0.56837	-0.47519	-0.62244
AVPR1B	0.137737	0.219348	0.321144	0.189788	0.228877
AVPR2	0.1269	0.12821	0.092434	0.007346	0.199017
AXIN1	0.03767	-0.02137	0.033549	0.047149	-0.12371
AXIN2	-0.01886	0.004591	-0.26864	-0.27307	0.027996
AXUD1	0.147754	0.075808	0.264506	0.041941	0.147055

AYTL2	0.014849	-0.09959	-0.02339	-0.02935	-0.02141
AZGP1	0.095254	-0.43905	-0.31972	-0.33186	-0.07853
AZI1	-0.07694	-0.00389	-0.15859	-0.21117	-0.02535
AZI2 ZCWPW2	-0.06266	-0.3483	-0.64321	-0.52005	-0.03839
AZIN1	-0.21107	-0.28249	-0.27131	-0.3037	-0.24209
AZU1	0.347585	0.458563	0.586546	0.31404	0.445107
B2M	-0.34364	-0.45078	-0.5333	-0.50664	-0.56254
B3GALNT1	0.286183	0.308628	0.538692	-0.00607	0.255556
B3GALNT2	-0.381	-0.4742	-0.55432	-0.3586	-0.35511
B3GALT1	0.22365	0.722757	0.591388	0.237986	-0.12186
B3GALTL	0.201628	0.151415	0.307754	0.191992	0.269178
B3GAT2	0.04184	0.067831	-0.06776	0.054508	0.052022
B3GAT3	0.128942	0.088022	0.033721	-0.01216	0.102543
B3GNT1	-0.03581	0.01348	0.118969	-0.0433	0.059386
B3GNT3	0.133788	0.432542	0.517558	0.454145	0.467357
B3GNT7	0.065221	0.310916	0.311072	0.244315	0.309833
B3GNTL1	0.091305	0.085412	0.186566	0.123853	0.056924
B4GALNT1	-0.16415	-0.18064	-0.18385	-0.42278	-0.3413
B4GALNT2	0.289912	-0.05802	0.444979	0.532373	0.313693
B4GALNT3	0.079315	0.075124	0.170022	0.178137	0.264796
B4GALNT4	-0.12668	-0.1282	-0.07801	0.032547	-0.04548
B4GALT1	0.189393	0.162647	0.052668	0.065286	0.266264
B4GALT2	0.059955	0.050756	-0.03119	-0.05953	-0.1299
B4GALT2 CCDC24	-0.07695	-0.08678	0.002402	0.166939	0.051747
B4GALT3	0.348637	0.526433	0.162947	-0.02533	0.05599
B4GALT4	-0.11286	-0.02004	-0.16434	-0.13187	-0.24942
B4GALT5	-0.16687	-0.37594	-0.35524	-0.37272	-0.31852
B4GALT6	-0.11505	-0.14887	-0.10586	-0.06429	0.135875
B4GALT7	-0.02239	-0.0897	-0.00457	-0.04608	-0.05483
B9D2	0.056074	-0.01035	-0.1871	-0.01025	0.014105
BAALC	0.143487	0.288162	0.265404	0.14141	0.10944
BACE1.	0.107341	0.155487	0.115948	0.100332	0.095755
BACH2	-0.11825	0.000643	-0.1511	-0.03398	0.091218
BAD GPR137	-0.08423	-0.18497	-0.0717	-0.00639	-0.03633
BAG1 CHMP5	-0.62313	-0.5242	-0.66935	-0.60927	-0.79467
BAG2	0.184478	0.07637	0.02935	0.008282	0.152608
BAG3	-0.31391	-0.16915	0.199255	-0.11299	-0.15124
BAG5 C14orf153	-0.11824	-0.08192	0.070977	-0.03787	-0.32576
BAHD1	-0.26429	-0.2051	-0.2292	-0.23671	-0.26296
BAI1	0.300211	0.430627	0.434969	0.289389	0.31932
BAI2	-0.55978	-0.6018	-0.72818	-0.50821	-0.45794
BAI3	-0.33218	-0.44941	-0.48933	-0.22998	-0.59338
BAIAP2L2	0.088073	0.337127	0.398357	0.239944	0.419037
BAIAP3	0.107977	0.009378	0.150808	0.138781	0.093727
BAK1	-0.1013	0.27177	-0.02025	-0.05993	-0.00321
BAMBI	-0.0069	-0.08663	0.066729	0.154078	0.016867
BANK1	0.218624	0.378579	0.604443	0.286252	0.43938
BANP	-0.11406	0.077868	0.162197	0.061822	0.071543
BAP1 PHF7	-0.70005	-0.4556	-0.7247	-0.66718	-0.49356
BAPX1	-0.05196	0.040598	0.134	-0.03062	0.108867

BARD1	-0.07108	-0.12452	0.071715	0.056142	-0.05934
BARHL1	-0.22725	0.05995	0.032961	0.279937	0.120033
BARHL2	-0.33143	0.134642	0.538121	0.281922	0.357015
BARX1	-0.22854	-0.0787	-0.04833	-0.02611	0.296701
BARX2	0.18586	-0.03171	0.541076	0.344735	0.268532
BAT1	-0.63915	-0.74925	-1.06585	-0.47457	-0.72342
BAT2	-0.66029	-0.54624	-0.78063	-0.59399	-0.64196
BAT2D1	-0.091	-0.42657	-0.5264	-0.53743	-0.43487
BAT3 APOM	-0.5865	-0.58213	-1.09022	-0.58431	-0.67163
BAT4	-0.59978	-0.55056	-0.83043	-0.56809	-0.54798
BAT5	-0.05494	0.048712	-0.22395	-0.14107	0.082356
BATF	0.330007	0.574328	0.756583	0.438458	0.558264
BATF2	-0.46077	-0.00395	-0.29922	-0.46355	-0.37732
BAX	0.030823	0.080606	0.190764	-0.03444	0.00379
BAZ1A	0.198351	-0.18091	0.082517	-0.09006	0.038001
BAZ1B	0.166248	0.250957	0.216549	0.175067	0.295318
BAZ2A	-1.48021	-1.34707	-1.56626	-1.52997	-1.55767
BBC3	-0.25947	-0.31711	-0.18203	-0.25716	-0.10726
BBOX1	1.947581	-0.34835	-0.21669	-0.48363	-0.49187
BBS10	-0.30266	-0.08757	-0.16218	-0.27841	-0.20304
BBS2	0.05498	0.090019	0.068817	0.061497	-0.05992
BBS5	0.278346	0.356857	0.372601	0.203089	0.409649
BBS7	-0.02527	-0.04042	-0.1844	-0.23929	-0.13653
BBX	-0.73392	-0.65099	-0.84581	-0.55304	-0.58046
BCAM	-0.28524	-0.576	-0.58255	0.014211	-0.07559
BCAN	-0.33016	-0.03298	-0.20905	-0.32609	-0.03663
BCAP29	0.025981	-0.15126	-0.09852	-0.11016	0.077936
BCAP31 ABCD1	0.21865	0.150156	0.308489	0.098584	0.192964
BCAR1	0.115132	0.271687	0.151531	0.055179	0.198095
BCAR3	0.21055	0.28416	0.337298	0.252016	0.20437
BCAS1	0.18594	-0.40249	0.002343	-0.09057	-0.40261
BCAS2	-0.21256	-0.11295	-0.37637	-0.3813	-0.0369
BCAS3	-0.25224	-0.26726	-0.73779	-0.48207	-0.48694
BCAS4	0.243134	0.308208	0.441151	0.300544	0.284968
BCAT1	-0.02756	0.011547	-0.32226	-0.32875	-0.29163
BCAT2	-0.03934	0.297268	0.071682	-0.02787	0.030921
BCDIN3D	-0.04701	0.029402	-0.32893	-0.22619	-0.17401
BCDO2	0.019308	-0.30771	-0.82953	-0.5525	-0.57655
BCHE	-0.42728	-0.36776	-0.32197	-0.312	-0.53072
BCKDHB	-0.76045	-0.71049	-0.70639	-0.5933	-0.69691
BCKDK	0.268769	0.278226	0.20494	0.125697	0.383247
BCL10	0.48961	0.495781	0.521521	0.221458	0.221022
BCL11A	-0.30057	-0.50287	-0.29057	-0.23215	-0.10804
BCL11B	-0.1918	0.027948	0.230371	-0.04564	-0.00722
BCL2	-0.66164	-0.82276	-0.70494	-0.49181	-0.47113
BCL2A1	0.332364	0.386125	0.418616	0.046248	0.171809
BCL2L1	-0.52232	-0.22306	-0.50328	-0.27899	-0.4487
BCL2L10	0.444624	0.676123	0.759638	0.497337	0.598748
BCL2L11	0.00204	0.034897	0.181152	0.03445	0.18816
BCL2L13	-0.47425	-0.34449	-0.71371	-0.41787	-0.56358

BCL2L2	-0.26781	-0.50873	-0.44958	-0.39756	-0.3343
BCL3	-0.23346	-0.32949	-0.3523	-0.14136	-0.44812
BCL6B	0.295631	-0.05307	0.617419	0.473386	0.465089
BCL7A	0.166015	0.480038	0.409012	0.235535	0.322806
BCL7B	0.151225	0.177707	0.105796	0.128728	0.216706
BCL7C CTF1	0.152607	0.132322	0.182186	0.182136	0.336297
BCL9	-1.20514	-1.41965	-1.05254	-1.10877	-1.07144
BCL9L	-0.25439	-0.14016	0.395468	0.170207	0.377825
BCLAF1	-0.77141	-0.2869	-0.84831	-0.57069	-0.73847
BCMO1	0.3001	0.558002	0.835031	0.064159	0.756375
BCORL1	-0.12406	-0.6575	-0.61733	-0.49783	-0.6401
BCORL2.	0.161378	0.334988	0.323404	0.494685	0.33185
BCR	0.196666	0.205299	0.23063	0.215217	0.46966
BCS1L	-0.12697	-0.3804	-0.29427	-0.36919	-0.33282
BDH2	-0.56043	-0.67737	-0.52744	-0.54591	-0.87732
BDKRB1	0.516266	0.401529	0.471764	0.421099	0.630758
BDKRB2	0.508761	0.571028	0.652692	0.436353	0.613073
BDP1	0.12785	0.098985	0.041222	0.073485	0.133765
BECN1	0.123868	0.272413	0.075397	0.042659	0.402712
BEGAIN	-0.05232	0.084233	-0.17489	0.139697	0.240777
BEST1	0.28155	0.132808	-0.07795	-0.02007	0.069761
BEST2	0.479783	-0.94457	-0.07024	-0.03594	-0.94945
BEST4	0.299346	0.769466	0.687709	0.393099	0.892579
BET1	-0.50425	-0.13638	-0.23086	-0.62016	-0.57752
BET1L RIC8A	-0.46617	-0.36277	-0.47569	-0.38237	-0.06715
BEX1	0.031	0.047907	-0.18182	-0.10887	-0.12332
BEX2	0.072891	0.10667	0.064722	-0.08989	-0.00187
BEX5	0.209896	-0.07748	-0.19699	0.155784	0.184288
BFSP1	0.190465	0.214344	0.322717	0.136286	0.261212
BFSP2	-0.23838	-0.20427	-0.40333	-0.1678	-0.33286
BHLHB2	-0.59931	-0.33514	-0.69639	-0.27879	-0.24804
BHLHB3	-1.59738	-1.52956	-1.60178	-1.18047	-0.46783
BHLHB4	0.045554	0.197852	0.462915	0.233174	0.196508
BHLHB5	-0.3386	-0.45134	-0.14243	-0.18955	-0.13844
BHLHB8	0.191763	0.545198	0.407109	0.421913	0.52538
BHLHB9	-0.03116	-0.26598	-0.21901	-0.26425	-0.50942
BHMT	0.395138	0.727761	0.87201	0.413127	0.598484
BICD1	-0.19435	-0.14759	-0.18696	-0.2112	-0.2672
BICD2	-0.05808	0.007007	-0.0508	-0.01199	-0.07773
BID	0.064184	0.127994	0.077911	0.126113	0.187892
BIK	-0.08175	0.200361	0.226248	0.130967	0.021673
BIN1	0.010838	0.062694	-0.11993	0.078758	-0.04508
BIN2	0.284397	0.39343	0.563726	0.304778	0.445529
BIN3	0.02357	-0.0814	-0.17622	-0.08932	-0.11321
BIRC2	-0.25789	0.021883	-0.09286	-0.02456	-0.26936
BIRC3	0.066513	-0.28184	-0.85518	0.032333	-0.58133
BIRC5	0.183875	0.385678	0.436853	0.165394	0.180448
BIRC6	-0.09372	-0.14247	-0.00733	-0.14263	-0.07889
BLK	0.39729	0.808107	0.473733	0.169401	0.565741
BLM	-0.4337	-0.51542	-0.62003	-0.41764	-0.57958

BLMH	-0.00642	0.244407	0.165418	-0.10412	0.144805
BLNK	0.434986	-0.54414	0.608221	0.334153	-1.22645
BLOC1S1	-0.1613	0.126866	0.186958	-0.03209	-0.17387
BLOC1S2	-0.43315	-0.31811	-0.58475	-0.21395	-0.5717
BLVRA	-0.06384	0.149134	0.086586	0.156898	0.060468
BLVRB SPTBN4	0.085411	0.003034	-0.25065	0.061222	-0.17679
BMI1	-0.39566	-0.29277	-0.32423	-0.18707	-0.15828
BMP1	-0.15258	-0.17908	0.045468	-0.13569	-0.03185
BMP10	0.310114	0.35421	0.143372	-0.08287	-0.48094
BMP15	0.15376	-0.02371	-0.21852	-0.09672	0.247054
BMP2	-0.14412	0.036706	0.056052	0.179328	0.20009
BMP2K	-0.17978	-0.17906	-0.12727	-0.04376	-0.01816
BMP3	-0.09136	0.36896	0.368316	0.141416	0.111451
BMP5	-0.27821	-0.55245	-0.40033	-0.37803	-0.43393
BMP6	-0.06076	0.064597	0.068222	0.225337	0.089085
BMP7	-0.05757	0.105811	0.130417	0.176666	0.265713
BMP8A	0.08099	0.180668	0.251269	0.052622	0.253903
BMPER	-0.15286	-0.24644	-0.19344	-0.0802	0.005884
BMPR1A	-0.2144	-0.12909	-0.21795	-0.07225	-0.06799
BMPR1B	-0.08507	-0.39248	-0.30051	-0.29525	-0.28884
BMPR2	-0.79549	-0.84057	-0.88734	-0.57911	-0.55703
BMS1	-0.29053	-0.08935	-0.62603	-0.27565	-0.42656
BNC1	-0.00033	0.261083	0.264809	0.133193	0.316858
BNIP1	-0.00871	0.161816	0.078482	-0.01318	0.044752
BNIP2	-0.32344	-0.24654	0.207153	-0.28806	-0.11283
BNIP3	-0.03928	-0.00428	0.028494	-0.00867	0.159354
BNIP3L	0.276255	0.15211	-0.06578	-0.50317	-0.72174
BOC	-0.14186	-0.12407	0.089738	-0.00851	0.058969
BOK	0.184544	0.365067	0.366751	0.188999	0.304367
BOLA1	0.346667	0.082756	0.255561	0.191815	0.249403
BOLA3	0.186613	0.110084	-0.1097	-0.32917	-0.74026
BOLL	0.332909	0.388347	0.574022	0.305668	0.483365
BOP1 HSF1	-0.08022	-0.01506	-0.06411	-0.11995	0.003955
BPGM	-0.57472	-0.54029	-1.07155	-0.6599	-1.10339
BPHL	0.00736	-0.03281	0.110967	0.002798	-0.19392
BPI	0.484509	0.660405	0.832345	0.291498	0.305622
BPIL1	0.309619	-0.59123	-0.37684	0.064045	0.258658
BPIL2	-0.10487	-0.04953	-0.20437	-0.32706	-0.35417
BPIL3	0.182506	-0.72629	-0.48087	-0.11831	0.147281
BPNT1	-0.04351	-0.27375	-0.52414	-0.30127	-0.47856
BPY2	-0.26366	-0.49317	-0.28498	-0.34314	-0.36911
BPY2B	-0.17126	-0.31416	-0.36332	-0.55156	-0.39745
BPY2C	-0.06732	-0.34495	-0.17186	-0.56034	-0.11269
BRAF	-0.02895	0.025377	-0.37159	-0.08433	0.075309
BRAP ACAD10	-0.05983	-0.29831	-0.25705	-0.02388	-0.21258
BRCA1 NBR2	-0.10697	-0.62601	-0.78907	-0.69632	-0.76506
BRCA2	0.226412	0.298901	0.004753	0.117854	0.024276
BRCTD1.	0.153973	0.33977	0.23659	-0.00739	0.223524
BRD1	0.269733	0.410025	0.546334	0.131034	0.421824
BRD2	-0.53689	-0.36652	-0.42048	-0.35366	-0.4793

BRD3	-0.00607	-0.15866	-0.02762	0.079928	0.08843
BRD4	0.205538	-0.55575	-0.99994	-0.82784	-1.0364
BRD7	0.369901	0.387033	0.344837	0.142133	0.042164
BRD8 KIF20A	-0.85422	-0.65904	-0.85758	-0.63348	-0.89229
BRD9 TRIP13	0.141048	-0.1042	-0.27864	0.010009	-0.0331
BRDG1	0.36139	0.268939	0.269803	-0.17076	-0.37714
BRF2	-0.1611	-0.13873	-0.35876	-0.27977	-0.20112
BRI3BP	-0.0305	-0.1642	-0.04836	-0.11838	-0.02582
BRI3P1	-0.06295	0.010729	0.002283	-0.03317	0.088662
BRIP1	-0.70609	-0.8347	-1.11182	-1.09699	-0.76097
BRMS1 AP001107.5	0.022622	0.066709	-0.02107	0.004244	0.069872
BRMS1L	-0.01682	0.06039	0.098643	-0.02533	-0.14228
BRP44 IQWD1	-0.44309	-0.5964	-0.59028	-0.42957	-0.35404
BRP44L	-0.00641	0.275543	0.150889	0.064304	0.081125
BRPF1	-0.55037	-0.34087	-0.49581	-0.4123	-0.33154
BRPF3	-0.15998	-0.14112	-0.28267	-0.0493	0.000168
BRS3	-0.01218	-0.1631	-0.06614	0.080144	0.140776
BRSK1	-0.26157	-0.24042	-0.45583	-0.45985	-0.45908
BRSK2	0.12362	0.165797	0.228189	0.224043	0.344449
BRUNOL4	-0.49582	-0.58076	-0.60436	-0.4385	-0.41479
BRUNOL5	-0.02696	-0.12007	-0.02846	-0.11013	-0.23814
BRUNOL6	0.05588	0.22036	-0.02943	0.064462	0.160269
BRWD1	-0.17341	-0.23558	-0.11742	-0.11873	-0.19139
BRWD3	-0.07929	-0.69944	-0.17133	-0.55545	-0.66017
BSCL2	0.037971	0.452622	0.198177	0.241997	-0.33527
BSDC1	-0.7658	-0.61245	-1.10801	-0.87725	-0.79076
BSG	-0.00491	-0.04012	0.125843	0.070017	0.141922
BSN	-0.07306	0.037423	-0.1577	-0.17594	-0.05182
BSND	0.350257	0.367574	0.535642	0.247128	0.282342
BSPRY	0.519695	0.809277	0.754595	0.19785	0.047002
BST1	0.327056	0.620207	0.403497	0.415083	0.288328
BST2	0.498867	0.465238	0.65069	0.521159	0.600068
BTAF1	0.08019	-0.03644	0.265858	0.095047	0.055848
BTBD1	0.212856	0.141927	0.372022	0.207816	0.246988
BTBD10	0.085529	-0.1575	-0.16271	-0.12192	-0.02734
BTBD12	-0.0496	0.020877	-0.24127	-0.00293	-0.00892
BTBD14A	0.097563	0.211396	0.215713	0.216661	0.145782
BTBD16	0.312295	0.33786	0.368103	0.136133	0.453944
BTBD2	-0.00142	-0.4121	-0.50748	-0.3637	-0.79808
BTBD7 KIAA1409	-0.19893	-0.17754	-0.19271	-0.29315	-0.34758
BTBD8	0.118092	0.1825	0.068221	0.104236	0.052227
BTBD9	0.414363	0.427748	0.493252	0.111109	0.302072
BTC	0.301566	-0.27744	-0.27061	0.214009	0.028598
BTF3	-0.03503	-0.33802	-0.62325	-0.47005	-0.23888
BTG1	-0.49889	-0.25423	-0.43361	-0.42323	-0.26263
BTG2	0.082245	0.070418	0.067521	-0.07452	-0.00616
BTG3	0.27509	0.162312	0.311582	0.264419	0.255563
BTG4 hsa-mir-34b AP002008.5	0.269048	0.486658	0.369889	0.301594	0.268153
BTK	-0.08258	0.530497	-0.05482	0.159704	0.116817
BTLA	0.097144	0.32868	0.255883	-0.05918	0.175985

BTN1A1	0.592036	0.620093	0.503398	0.403754	0.211361
BTN2A1	-0.37497	-0.38302	-0.57654	-0.50524	-0.56867
BTN2A2	-0.03482	0.013689	-0.4308	-0.1937	-0.31244
BTN2A3	-0.63161	-0.53885	-1.05603	-0.82245	-0.69242
BTN3A1	-0.20813	-0.58045	-0.69857	-0.42637	-0.87009
BTN3A2	-0.88493	-1.24933	-1.60438	-1.02989	-1.51502
BTN3A3	0.194566	-0.74506	-0.92366	-0.05684	-0.66841
BTNL2	0.088896	-0.79145	-0.84488	-0.51379	-0.57689
BTNL3	-0.13442	-1.12409	-0.60057	-0.5231	-1.01172
BTNL8	0.545893	-0.1356	0.533357	0.411856	0.419955
BTNL9	0.256087	-0.74608	-0.72174	-0.36027	-0.07736
BTRC	-0.0667	0.073747	-0.13851	0.060245	-0.40157
BUB1	-0.4123	-0.53682	-0.58592	-0.3319	-0.22988
BUB1B	-0.31455	-0.11197	-0.75176	-0.48142	-0.71616
BUB3	-0.31319	-0.14293	-0.2153	-0.28915	-0.28819
BUD13	-0.62744	-0.46644	-0.80245	-0.3392	-0.7559
BVES	0.064937	0.011978	0.118359	0.093775	-0.05226
BX284668.4	0.392967	0.568797	0.809488	0.499725	0.595797
BX322799.22 NOXA1	-0.03464	0.022427	0.03068	-0.02382	0.056209
BX323046.3	-0.32886	-0.30956	-0.50782	-0.43137	-0.09345
BX664615.10	0.084885	-0.36926	-0.22291	-0.12944	-0.49754
BX664615.10 CR769767.5	-0.1002	-0.91876	-0.9282	-0.24929	-0.92422
BX936347.3 ATP6AP1	0.28125	0.221382	0.449444	0.132342	0.326439
BXDC1	0.185137	0.167582	-0.32789	-0.34106	-0.20206
BXDC2	0.23923	0.116114	0.316918	-0.07009	0.075965
BXDC5	0.102099	-0.31016	-0.08371	-0.11235	-0.08673
BZRAP1	0.253761	0.413819	0.443238	0.102025	0.150906
BZRPL1	0.249868	0.401802	0.509376	0.163879	0.519653
BZW1	-0.16647	-0.1498	-0.35326	-0.09988	-0.14842
C10orf107	0.199674	0.108435	0.35657	0.453527	0.442505
C10orf11	0.582499	0.544637	0.750985	0.459632	0.630516
C10orf114	-0.05601	-0.11498	0.009333	-0.06945	-0.0246
C10orf116	0.362721	0.499617	0.483597	0.29578	0.482757
C10orf118	0.067288	-0.13584	-0.37812	-0.09664	-0.23766
C10orf119	0.024738	-0.02394	-0.00675	0.090793	-0.02805
C10orf12	0.730501	0.96815	1.011441	0.510507	0.74148
C10orf120	-0.11018	-0.02607	0.037755	0.007824	-0.38396
C10orf125	0.103464	-0.08283	0.053226	0.05572	0.217079
C10orf129	0.22519	-0.62627	0.066262	-0.08285	-0.96486
C10orf132	0.127077	0.083802	0.211287	0.019776	0.317158
C10orf137	0.001615	-0.00032	-0.17366	-0.17084	0.061785
C10orf140.	-0.92004	-0.79691	-0.71342	-0.75411	-0.68414
C10orf22	0.067427	0.098131	0.088032	0.009507	0.04822
C10orf25 ZNF22	0.342913	0.399805	0.46421	0.089121	0.282321
C10orf26	-0.04328	0.075906	0.174993	0.146614	0.350266
C10orf27	0.296175	0.24967	0.418795	0.252663	0.338983
C10orf28	0.291806	0.161579	0.275702	0.109517	0.100251
C10orf30	0.2875	0.348654	0.521978	0.160033	0.236433
C10orf33	0.105273	-0.02384	0.242058	0.057581	0.099684
C10orf35	0.132308	0.107301	0.162169	0.244316	0.110569

C10orf38	-0.07337	-0.10975	-0.04623	-0.0134	0.036678
C10orf4	-0.84215	-0.95292	-1.16732	-0.93895	-1.06497
C10orf46	0.217817	0.363093	0.362515	0.20509	0.26461
C10orf47	-0.07779	0.041282	0.070634	0.058087	0.126177
C10orf49	0.351184	0.629512	0.614264	0.410813	0.619431
C10orf53	0.088605	0.152007	0.75813	0.285105	-0.01987
C10orf56	-0.28576	-0.32961	-0.15097	-0.21454	-0.29457
C10orf57	0.011505	0.156125	0.081688	0.066914	0.37752
C10orf59	0.497944	0.614195	0.879395	0.536171	0.533109
C10orf6	-0.15486	-0.28031	-0.1945	-0.33919	-0.14578
C10orf63 THNSL1	-0.7947	-0.78397	-0.66285	-0.63375	-0.88553
C10orf64	0.160595	0.186761	0.355714	0.039252	0.025236
C10orf65	0.040895	-0.11458	0.053655	-0.11893	-0.11574
C10orf67	0.076776	0.411202	2.034225	0.14494	0.34201
C10orf72	0.271918	0.315542	0.233496	0.214647	0.354716
C10orf78	-0.65408	-0.55402	-0.79625	-0.45637	-0.48568
C10orf79	-0.22962	-0.22962	-0.25281	-0.29142	-0.14284
C10orf81	-0.3704	-0.18855	-0.41107	-0.34623	-0.46782
C10orf82	0.19799	0.506337	0.472167	0.361199	0.301793
C10orf83	0.222309	0.310413	0.144139	0.236791	0.11161
C10orf84	-0.111	-0.12242	0.110665	-0.12875	-0.13373
C10orf88	0.123394	0.303365	0.265639	0.003827	0.138885
C10orf90	0.273399	0.479898	0.758827	0.209728	0.323342
C10orf92	0.473641	0.620854	0.842264	0.460369	0.532046
C10orf93	0.138526	0.297407	0.286786	0.266587	0.365551
C10orf95	0.479208	0.448266	0.560715	0.412497	0.557801
C10orf96	0.543192	0.794957	0.770121	0.578269	0.678331
C10orf97	0.227045	0.269593	0.179642	0.103386	0.159885
C10orf99	-0.24012	0.096189	-0.04618	-0.0834	-0.0171
C11orf10 FEN1	-0.44922	-0.42452	-0.5674	-0.38793	-0.32016
C11orf16	0.144343	0.324179	0.481317	0.204942	0.286187
C11orf17	0.003727	-0.09334	-0.29377	-0.04752	-0.20077
C11orf2	0.306241	0.418891	0.226768	0.235412	0.419386
C11orf2 TM7SF2	0.068803	0.131837	0.02461	0.126506	0.046117
C11orf21 TSPAN32	0.233971	0.342368	0.30992	0.192824	0.283124
C11orf24	-0.04941	0.006131	-0.14623	-0.0142	-0.0188
C11orf30	-0.37803	-0.23961	-0.43802	-0.33412	-0.40458
C11orf35 RASSF7	0.064235	0.008233	0.118608	0.204483	0.151674
C11orf38	0.090239	-0.66853	-0.62117	-0.61761	-0.76602
C11orf40	-0.09466	-0.27705	-0.10339	-0.17898	-0.04223
C11orf41	0.127288	-0.31523	0.170377	-0.23724	-0.21918
C11orf42	-0.11945	-0.33602	-0.4099	-0.50165	-0.45436
C11orf44	0.204362	0.248427	0.291004	0.257291	0.223849
C11orf46	-0.06091	-0.10497	-0.49531	-0.40094	-0.1717
C11orf47	0.408996	0.768081	0.54485	0.412921	0.663373
C11orf48 AP001458.6	0.089651	-0.06776	-0.1226	0.099521	0.075258
C11orf49	0.332187	0.176	-0.0571	0.173903	-0.09071
C11orf51	-0.71632	-0.21939	-0.44152	-0.55185	-0.45901
C11orf52	0.584394	0.67584	0.930465	0.379638	0.504143
C11orf53	-0.16903	0.034371	-0.22564	-0.36098	-1.26149

C11orf55. COMMD9	0.647602	0.639829	0.852162	0.429592	0.574039
C11orf56 CNGA4	-0.47116	-0.47693	-0.56769	-0.33753	-0.47704
C11orf58	-1.0404	-0.87603	-1.27507	-0.87497	-0.70427
C11orf60	0.378843	0.19852	0.266615	0.343677	0.302547
C11orf61	-0.22905	-0.07849	-0.11463	-0.15185	-0.21673
C11orf63	0.435875	0.503151	0.53671	0.433842	0.60037
C11orf64	0.507743	0.528443	0.144262	0.390614	0.294681
C11orf65	0.263945	0.294338	-0.24212	0.055939	-0.33558
C11orf66	0.286235	0.597043	0.895104	0.530191	0.52885
C11orf68 DRAP1	-0.17926	-0.03904	-0.07275	-0.05309	-0.07088
C11orf70	-0.1084	0.065053	-0.10153	-0.16453	0.053016
C11orf71 RBM7	-0.62715	-0.22709	-0.83177	-0.44206	-0.8507
C11orf72. NDUFV1	0.091662	0.160508	-0.0809	0.199119	0.058574
C11orf73	-0.63943	-0.23237	-0.70292	-0.61456	-0.76476
C11orf77 HARBI1	-0.53063	-0.45605	-0.66514	-0.33363	-0.13984
C11orf80	0.027086	0.056363	-0.06607	0.09534	0.154738
C11orf80 RCE1	0.06158	-0.01877	0.057593	0.058021	0.061879
C11orf9	0.312868	0.414771	0.494072	0.351853	0.423914
C12orf11 FGFR1OP2	-0.49789	-0.41672	-0.33726	-0.33726	-0.33029
C12orf23	-0.09164	-0.14818	-0.02172	-0.13157	-0.05023
C12orf25	0.106316	0.33283	0.260579	0.119961	0.003877
C12orf29	0.455197	0.488714	0.483451	0.23474	0.298176
C12orf30	-0.14543	-0.32704	-0.03223	-0.17195	-0.27642
C12orf31	-0.24507	-0.30271	-0.33336	-0.26676	-0.21095
C12orf34	0.549279	0.149934	0.471428	0.180545	0.05771
C12orf35	0.011918	0.249161	0.201016	0.01469	0.156396
C12orf39	-0.06303	0.224679	-0.00121	-0.10419	-0.2174
C12orf4	0.573621	0.976151	1.063078	0.753049	0.854681
C12orf41	0.077303	-0.39905	-0.08536	-0.16728	-0.37991
C12orf42	-0.55987	-0.20048	-0.4387	-0.42059	-0.34532
C12orf43	-0.54771	-0.59876	-0.63462	-0.60298	-0.76253
C12orf44	-0.06154	-0.17558	-0.23609	-0.05913	-0.21677
C12orf45	-0.03661	-0.33867	-0.21717	-0.13762	-0.16859
C12orf48	-0.04556	0.010608	-0.08088	-0.19527	-0.14283
C12orf49 RNFT2	-0.1553	-0.58227	-0.75852	-0.30941	-0.43576
C12orf5	-0.06756	-0.04017	-0.0356	0.382789	-0.07571
C12orf50	-0.21948	-0.13753	-0.18765	-0.29939	-0.26891
C12orf51	-0.38021	-0.52285	-0.89561	-0.7452	-0.83615
C12orf54	-0.43806	-0.25958	-1.15928	-0.90591	-1.43424
C12orf59	0.524335	-0.32858	0.053762	-0.04268	0.041816
C12orf60	0.203907	0.069409	0.255371	-0.11315	-0.10932
C12orf62	0.077108	0.058885	0.017273	0.089376	-0.02057
C12orf63	0.189963	0.312776	0.328708	0.119852	0.227532
C12orf64.	-0.15643	0.054088	-0.29438	-0.14122	-0.44692
C12orf65	-0.4539	-0.50177	-0.42618	-0.34673	-0.28267
C13orf1	-0.12443	0.031681	-0.12869	-0.06967	-0.13277
C13orf15	0.187256	0.279638	0.179354	0.32707	0.373291
C13orf16	0.386938	0.381867	0.481394	0.269668	0.349187
C13orf18	0.189913	0.257582	0.257483	0.303532	0.462054
C13orf23 NHLRC3	-0.25166	-0.29679	-0.27544	-0.2325	-0.2897

C13orf26	0.333137	0.688834	0.768362	0.328649	0.376671
C13orf27	0.344787	0.47393	0.530063	0.273803	0.361113
C13orf28	-0.2015	-0.68333	-0.64125	-0.559	-0.62711
C13orf3 MRP63	-0.02349	0.268604	0.085	0.050415	0.072251
C13orf30	-0.46367	-0.46708	-0.81559	-0.68585	-1.03615
C13orf33	0.322243	0.363724	0.396646	0.300355	0.270748
C13orf35	0.586098	0.559008	0.621157	0.374566	0.450899
C13orf7	0.170898	-0.48794	-0.49857	-0.40009	-0.32817
C13orf8	0.228117	0.233086	0.092156	0.010706	-0.00064
C14orf1 TTLL5	-0.40859	-0.15256	-0.42379	-0.28454	-0.38561
C14orf10 KIAA0391	-0.30839	-0.46506	-0.41235	-0.25173	-0.42022
C14orf101	0.072888	0.075865	-0.10387	0.017309	-0.06251
C14orf102	0.074601	-0.44767	-0.88934	-0.57702	-0.78467
C14orf103	-0.04562	0.146599	0.08971	-0.13269	0.155936
C14orf104	0.050317	0.187617	0.097661	0.058964	0.14689
C14orf105	0.261593	0.258226	0.454868	0.279504	0.209162
C14orf106	0.363455	0.279569	0.336251	0.24939	0.571695
C14orf112	0.009052	0.411657	0.123402	0.204878	-0.06884
C14orf115	-0.11272	-0.02458	0.140996	-0.12815	0.025694
C14orf118	-0.28342	-0.28936	-0.279	-0.42946	-0.48845
C14orf122	-0.21953	-0.19397	-0.34369	-0.3102	-0.38477
C14orf124	-0.58121	-0.32864	-0.62925	-0.4446	-0.63619
C14orf126	-0.07999	0.140531	0.179031	-0.03019	0.132849
C14orf129	0.122764	0.363432	0.511049	-0.02671	0.127277
C14orf131	0.626301	0.825497	0.935245	0.474387	0.796117
C14orf133 AHSA1	-0.64873	-0.62886	-0.60831	-0.5536	-0.71693
C14orf138 SOS2	-0.40565	-0.42446	-0.54694	-0.44752	-0.70189
C14orf140	0.089985	0.116719	0.086532	0.363549	-0.20048
C14orf142 C14orf130	-0.09123	-0.43731	-0.18816	-0.16521	-0.21458
C14orf143 TDP1	-0.36954	-0.68744	-0.68882	-0.571	-0.68878
C14orf145	0.115622	0.129861	-0.28216	-0.44905	-0.4739
C14orf147	-0.29159	-0.23225	-0.18725	-0.27169	-0.30928
C14orf148	0.462752	0.500222	0.492408	0.314102	0.437662
C14orf149 C14orf100	-0.24784	-0.17288	-0.12296	-0.17161	-0.12982
C14orf151.	0.06689	-0.04973	0.242702	0.11582	0.116902
C14orf152	0.326313	0.389636	0.578573	0.293616	0.263664
C14orf155	-0.8318	-0.99225	-0.23811	-0.70882	-0.79753
C14orf159	-0.04793	-0.34736	-0.02284	0.03669	0.143449
C14orf165.	0.069641	-0.17444	0.034142	-0.31352	-0.2513
C14orf166	-0.04203	-0.04142	0.003433	0.204444	-0.17725
C14orf166B	0.192712	0.442438	0.136934	-0.05207	0.197508
C14orf167. DHRS4	0.194503	-0.09596	-0.14853	-0.08884	0.021039
C14orf172	-0.06314	-0.06376	-0.20783	-0.10482	-0.11311
C14orf177	0.464088	0.289964	0.011249	0.322839	0.189653
C14orf179	-0.33276	-0.24815	-0.48862	-0.3882	0.011158
C14orf180	0.314596	0.079972	0.233835	0.242466	0.296875
C14orf2	0.146082	0.135297	0.222341	0.035302	0.192653
C14orf21	0.270543	0.081824	0.006445	-0.23831	0.00183
C14orf24	0.005493	0.035879	-0.42776	0.025462	-0.22475
C14orf28	0.047116	0.163187	0.23932	0.071126	0.154044

C14orf29	0.222546	0.390247	0.474097	0.227877	0.464315
C14orf33. KTN1	-0.13475	-0.14	-0.13942	-0.04222	-0.05992
C14orf37	-0.0139	0.010597	0.097036	0.111183	-0.01267
C14orf39	0.228648	-0.06358	0.746317	0.010213	-0.22401
C14orf43	-0.10094	-0.05922	0.137451	-0.01891	0.049605
C14orf44 COQ6	0.012594	-0.1638	-0.22123	-0.13858	-0.13116
C14orf48	0.023267	0.110776	0.003056	-0.01159	0.02682
C14orf49	0.49466	0.738776	0.703895	0.528236	0.695498
C14orf50	0.477677	0.787838	0.887783	-0.11042	0.371779
C14orf53	-0.68256	-0.60153	-0.94588	-0.75224	-1.00021
C14orf68	0.170571	0.365698	0.375476	0.1621	0.28626
C14orf70.	0.326173	0.751221	0.626807	0.487014	0.714984
C14orf80	-0.12106	-0.14424	-0.15497	-0.19588	-0.19592
C14orf83	0.295337	0.340166	0.413372	0.139663	0.279723
C14orf93	0.081433	-0.11967	-0.37378	0.016143	-0.00376
C14orf94	0.221568	0.25093	0.225881	0.13591	0.267055
C15orf15	-0.41411	-0.61191	-0.43839	-0.40745	-0.50192
C15orf17	0.195251	-0.04907	0.053124	0.16317	0.028625
C15orf2	0.227961	0.323845	0.54018	0.388246	0.563352
C15orf20	-0.44015	-0.34857	-0.8945	-0.43486	-0.53951
C15orf23	-0.12481	0.053375	-0.05055	0.053555	-0.14888
C15orf24 PGBD4	-0.44116	-0.32965	-0.57031	-0.12651	-0.63908
C15orf27	-0.07176	-0.20098	-0.22804	-0.2862	-0.10623
C15orf29	0.113851	-0.06455	-0.00278	-0.1044	-0.21439
C15orf32	-0.01906	-0.13526	-0.06776	-0.19464	0.018585
C15orf33	-0.02434	-0.20502	-0.32462	-0.29148	-0.27929
C15orf33 DTWD1	-0.44802	-0.66345	-0.63544	-0.53884	-0.60836
C15orf38	0.035372	0.189938	0.297212	0.178046	0.265046
C15orf39	-0.02327	0.18999	-0.03978	-0.06589	-0.03721
C15orf40	0.20569	0.269027	0.348711	0.282731	-0.10841
C15orf42	0.468905	0.561635	0.483013	0.061454	0.377378
C15orf43	0.35753	0.468762	0.642617	0.24038	0.306239
C15orf44	-0.04664	-0.0355	-0.10165	0.019333	-0.15199
C15orf51.	0.040078	0.097823	-0.07897	0.016399	0.057898
C15orf52	-0.24637	0.264191	0.45632	0.340764	0.393658
C15orf53	0.029352	0.039548	0.082522	0.222261	0.120846
C15orf54	-0.35062	-0.47291	-0.74337	-0.53012	-1.04153
C16orf13	0.073945	-0.03772	0.028513	0.093527	0.072865
C16orf24	0.035695	0.047363	-0.00047	0.123345	-0.02287
C16orf35	-0.37147	-0.57954	-0.65377	-0.54294	-0.6371
C16orf42 GNPTG	0.264713	0.159054	0.198433	0.15723	0.020554
C16orf44	0.104843	0.304861	0.192735	0.215349	0.186422
C16orf45	0.202625	0.115795	-0.14271	0.280313	-0.04871
C16orf46	0.268961	0.257814	0.454036	0.213737	0.145584
C16orf47	0.510528	1.059479	1.172129	0.659886	0.833061
C16orf48 C16orf86	0.164879	-0.04193	0.030904	0.221342	-0.07087
C16orf5	0.19152	0.320463	0.183706	0.127526	0.013792
C16orf51 PMM2	-0.43145	-0.54173	-0.86426	-0.47932	-0.62735
C16orf53	-0.08581	0.082017	-0.22453	-0.07828	-0.28388
C16orf54	0.144276	0.252652	-0.05025	0.068354	0.020296

C16orf58	0.011468	0.034838	-0.07205	0.042096	0.00649
C16orf59	-0.05794	-0.0134	-0.01392	0.182331	0.143497
C16orf61 CENPN	0.063139	-0.19342	0.021267	0.03169	-0.3447
C16orf62	0.272837	0.297542	-0.04634	0.201944	0.337664
C16orf63	-0.12219	0.18042	-0.06198	-0.03459	-0.15639
C16orf65	0.151754	0.21267	0.231764	-0.07642	0.01763
C16orf67.	-0.10847	0.205851	-0.10981	0.037664	0.075422
C16orf68	-0.10029	-0.02516	-0.10935	-0.05836	-0.01175
C16orf7 ZNF276	0.053567	-0.05695	0.016304	0.072639	0.203573
C16orf70	0.135192	0.052592	-0.11467	0.076875	0.044138
C16orf72	-0.05027	-0.12134	0.064741	-0.03516	-0.09698
C16orf73	-0.27982	-0.50664	-0.25569	-0.57553	-0.64601
C16orf74	0.019434	0.089137	0.157789	0.134926	0.18989
C16orf75	-0.20045	-0.13909	-0.17934	-0.20227	-0.23999
C16orf76	0.094537	0.069233	0.267058	0.020553	-0.06688
C16orf77	0.522379	0.740034	0.759101	0.338006	0.338363
C16orf78	-0.55116	-0.7872	-1.01881	-0.54134	-0.98939
C16orf79	0.269715	0.471367	0.418599	0.186719	0.16106
C16orf80	0.067077	0.057688	-0.09006	-0.14613	-0.12694
C16orf81	0.251832	0.176623	0.503679	0.223079	0.290383
C16orf85 ZC3H18	0.028401	0.129671	-0.0441	0.099355	-0.1288
C17orf28	0.047349	0.20245	-0.09246	0.073714	0.081635
C17orf37	-0.02568	0.19725	0.01875	0.002827	0.10102
C17orf42	-0.18883	0.006471	-0.72567	-0.16837	-0.1523
C17orf45	-0.28905	-0.21088	-0.58919	-0.38279	-0.15471
C17orf46	0.159016	0.111299	0.047894	0.192979	0.154252
C17orf47	0.575072	0.625571	0.91492	0.467456	0.62955
C17orf50	-0.2436	-0.33111	-0.38005	-0.40175	-0.30097
C17orf53	0.178826	0.054804	0.211145	-0.05655	0.069555
C17orf54.	0.453682	0.800536	0.782456	0.269396	0.539733
C17orf55	0.150732	0.177299	0.150473	0.179384	-0.10599
C17orf56	0.046952	-0.12982	-0.16123	-0.00645	0.214392
C17orf57	0.202746	-0.1432	0.420606	0.369886	0.197193
C17orf58	-0.01824	-0.12466	-0.19425	-0.2504	-0.13622
C17orf59	0.02246	-0.01394	0.015206	-0.04162	0.091782
C17orf61	-1.59727	-0.94816	-1.49979	-1.21971	-1.08271
C17orf62	0.070131	-0.02692	-0.30731	-0.00043	-0.10576
C17orf63	-0.14618	0.180355	0.132263	-0.04768	-0.02336
C17orf64	0.354111	0.740845	0.648576	0.460254	0.488518
C17orf65 TMUB2	-0.43255	-0.93409	-1.07045	-1.04016	-0.88104
C17orf66	0.130075	0.048517	0.205729	-0.0637	0.059844
C17orf67 DGKE	0.064285	0.019615	0.003346	-0.16467	0.248117
C17orf68 PFAS	-0.46266	-0.2356	-0.58458	-0.51766	-0.33918
C17orf70	-0.05426	-0.06226	0.039966	-0.01294	-0.00072
C17orf71	0.22501	-0.12012	-0.43181	0.089228	0.035811
C17orf74	0.420446	0.408342	0.51459	0.387927	0.398022
C17orf75	0.158169	0.206713	0.141779	0.166948	0.282313
C17orf76	-0.05828	0.011902	-0.10311	-0.05549	0.079712
C17orf79	0.008408	0.170212	0.000509	0.003136	0.248438
C17orf82	0.115896	-0.12803	0.302665	-0.10657	0.131731

C17orf85	0.458455	0.348525	0.492955	0.01362	0.286752
C17orf87	0.507144	0.335556	0.558769	0.422048	0.544006
C17orf90 CCDC137	0.314749	0.069547	0.242587	0.155272	0.276861
C17orf91 WDR81	-0.33137	-0.2722	-0.59785	-0.29099	-0.16819
C17orf92	-0.23688	-0.4507	-0.3493	-0.19434	-0.11951
C18orf10 KIAA1328	-0.02115	-0.02129	-0.26381	-0.27784	-0.0009
C18orf17	0.257029	0.216463	-0.28684	0.022999	0.301968
C18orf19 RNMT	-0.30399	-0.27527	-0.21892	-0.15657	-0.13001
C18orf20.	0.297762	0.334238	0.526266	0.120333	0.238583
C18orf21	-0.16478	0.00315	-0.18582	-0.20433	0.022404
C18orf22	-0.43335	-0.19169	-0.82078	-0.48402	-0.42314
C18orf24	-0.02383	-0.03805	-0.36453	-0.06736	-0.05936
C18orf25	-0.15799	0.009284	-0.10248	-0.10901	0.127503
C18orf26	-0.01584	-0.51835	0.157255	-0.43841	-0.75635
C18orf32	0.469403	0.301836	0.263638	0.346684	0.103535
C18orf34	-0.12555	0.361491	0.101416	-0.08511	0.092441
C18orf37	0.178193	0.243507	0.008797	0.089216	0.333736
C18orf4	-0.60306	-0.62213	-0.55278	-0.51934	-0.24908
C18orf43	0.069984	0.269282	0.191674	0.110476	0.075682
C18orf45	0.02942	-0.41239	-0.69977	-0.4103	-0.38772
C18orf54	-1.34498	-0.73215	-1.50452	-1.22956	-0.57768
C18orf56 TYMS	-0.01172	0.093321	0.023227	-0.08719	0.006388
C18orf62	0.625015	0.62266	0.678132	0.527939	0.431456
C18orf8	0.012736	0.070332	0.106433	-0.02079	0.098246
C19orf10	0.20312	0.184928	0.240681	0.057323	0.247083
C19orf12	0.144668	0.136292	0.102271	0.054088	-0.00095
C19orf15	-0.42539	-0.10572	-0.42302	-0.43335	-0.56634
C19orf18	0.494844	0.626571	0.722794	0.437087	0.465755
C19orf19	0.555564	0.480316	0.647139	0.390947	0.415893
C19orf2	0.003626	-0.12065	-0.00053	0.083977	0.085367
C19orf20	-0.08465	0.089367	-0.02996	0.139622	0.047928
C19orf21	0.161076	0.091636	0.338022	0.210327	0.137821
C19orf22	-0.07795	-0.14135	-0.23553	-0.28062	-0.10372
C19orf24	0.182013	0.247516	0.481257	0.301295	0.169003
C19orf26	-0.04541	0.056527	0.070144	-0.01402	0.033809
C19orf30	-0.41554	-0.44276	-0.713	-0.47118	-0.58529
C19orf33 YIF1B	0.187372	0.418772	0.41577	0.250182	0.458297
C19orf35	0.292744	0.416858	0.532743	0.261496	0.254439
C19orf39	-0.02842	-0.59788	-0.73201	-0.41233	-0.73102
C19orf41	0.405569	0.121543	0.462914	0.380564	0.355135
C19orf42	0.524542	0.765649	0.434159	0.303583	0.414365
C19orf43	0.016921	0.025911	-0.0572	-0.06999	0.063503
C19orf45	0.599631	0.480059	0.640206	0.302332	0.385316
C19orf47 PLD3	0.024932	-0.33064	-0.66103	-0.28904	-0.47997
C19orf48	-0.46156	-0.15542	-0.39719	-0.39323	-0.30633
C19orf50	0.224291	0.01152	0.323855	0.215788	0.156546
C19orf51 AC010327.8	-0.1786	-0.1425	-0.38297	0.293492	-0.3558
C19orf54 SNRPA	-0.35964	-0.22821	-0.39979	-0.26181	-0.27817
C19orf56 AC010422.7	-0.30621	-0.25293	-0.38161	-0.11191	-0.45838
C19orf57 CC2D1A	0.19673	0.001665	0.085407	-0.13917	0.24909

C19orf59	0.068474	-1.13272	-1.05609	0.017986	-0.64469
C19orf6 AC004528.1	-0.00567	-0.20717	-0.40153	-0.22378	-0.16838
C19orf60	0.051048	-0.05775	0.190251	-0.09266	-0.1443
C19orf61	0.368814	0.602615	0.423284	0.205307	0.107514
C19orf62	0.080238	0.283216	0.164911	-0.00031	-0.06908
C1GALT1	0.29491	0.61693	0.253037	-0.13132	-0.0653
C1GALT1C1	0.047547	0.085042	0.176737	-0.22904	-0.22958
C1orf100	0.435668	0.680899	0.320742	0.294592	0.516452
C1orf101	0.202221	0.456055	0.258863	0.225841	0.337855
C1orf102	0.080015	0.232149	-0.27318	0.006337	0.120976
C1orf103	0.029637	-0.2679	-0.62227	-0.38147	0.165993
C1orf106	-0.28647	0.433747	0.185604	0.087609	0.151077
C1orf107	0.466709	0.38334	0.483363	0.329829	0.213902
C1orf108	-0.21085	-0.19537	-0.19355	-0.21768	-0.0863
C1orf109 CDCA8	-0.0968	-0.49635	-0.39651	-0.29117	-0.43889
C1orf110	-0.08864	0.226837	0.013937	-0.2572	-0.12591
C1orf111	0.688754	0.58984	0.852871	0.342782	0.556387
C1orf113	0.253654	0.395962	0.277125	0.363719	0.3249
C1orf114	-0.27411	-0.49739	-0.6854	-0.75809	-0.85907
C1orf115	0.4048	0.625012	0.674059	0.335462	0.288891
C1orf116	-0.16087	-0.76755	-0.59817	-0.50969	-0.58847
C1orf119	0.023333	-0.22619	-0.0199	-0.01815	-0.0379
C1orf120.	-0.81712	-1.72951	-0.76314	-1.33341	-1.38335
C1orf123	-0.08584	-0.1163	-0.25641	-0.33159	0.043678
C1orf125	0.22166	0.008165	-0.06997	-0.20429	-0.29675
C1orf126. TMEM51	-0.15478	-0.18981	-0.14063	-0.0089	-0.00719
C1orf127	0.328243	0.586583	0.786606	0.62573	0.782341
C1orf128	0.223492	0.114394	0.109205	0.218428	0.241003
C1orf129	-0.14111	-0.11205	-0.2085	-0.18799	-0.09486
C1orf130	0.093715	0.485716	0.375209	-0.07619	0.209522
C1orf131 GNPAT	-0.60801	-0.64324	-0.80304	-0.74833	-0.50096
C1orf132.	0.556226	0.998244	0.868863	0.582639	0.617521
C1orf133	-0.19734	-0.52206	-0.56856	-0.37265	-0.25964
C1orf135	-0.01805	-0.03964	-0.04706	-0.09421	0.026149
C1orf137	0.360746	0.515604	0.701495	0.308472	0.427196
C1orf138	-0.6663	-0.6865	-0.7883	-0.43104	-0.46154
C1orf14	0.404238	0.368941	0.498315	0.314499	0.408068
C1orf142	-0.29405	-0.35822	-0.42187	-0.24262	-0.36879
C1orf144	0.186757	0.087404	0.215754	0.081934	0.239432
C1orf145	0.057267	0.180565	0.30736	0.00615	0.048732
C1orf145 OBSCN	-0.22639	0.040726	-0.12887	0.002337	0.094937
C1orf146	0.448817	0.671681	0.841863	0.506119	0.563763
C1orf147	0.273379	-0.0991	-0.00905	-0.3595	-0.07743
C1orf148 C1orf69	-0.046	-0.06403	-0.0954	-0.09661	0.048369
C1orf149	0.042271	0.138731	0.047318	0.024816	0.050941
C1orf151	-0.15852	-0.17103	-0.33152	-0.11325	-0.17654
C1orf156 C1orf112	-0.438	-0.72362	-0.64769	-0.57752	-0.50404
C1orf157.	0.227016	0.365667	0.537633	0.059221	-0.26613
C1orf158	-0.94397	-1.09398	-1.27291	-0.58717	-0.12585
C1orf159	0.102069	0.128079	0.281647	0.15893	0.180699

C1orf160	0.100665	0.287906	0.334818	0.123147	0.210604
C1orf162	0.621606	0.863884	0.905973	0.633265	0.486858
C1orf163	0.12936	0.026627	0.036074	-0.40438	0.081173
C1orf164	-0.87674	0.065668	-0.23005	-0.42541	0.267625
C1orf166	-0.09514	-0.05075	-0.05975	-0.05863	0.05729
C1orf168	0.450845	0.370734	0.350119	0.405477	0.30807
C1orf172	0.077853	0.151758	0.248456	0.194899	0.130449
C1orf174	-0.3445	-0.03435	-0.41254	-0.10434	-0.04536
C1orf176	0.090292	0.598275	-0.0056	-0.08516	0.194911
C1orf177	0.41878	0.471493	0.449479	0.327098	0.451936
C1orf180.	-0.00048	0.088831	-0.09755	-0.32438	-0.01721
C1orf181	0.341074	0.334328	0.431643	0.138132	0.169214
C1orf183	-0.29966	-0.24034	-0.31578	-0.41546	-0.30625
C1orf189 C1orf43	0.018037	-0.00769	-0.28462	-0.1489	-0.11536
C1orf19	-0.60967	-1.02646	-0.9965	-0.74721	-0.94574
C1orf190	-0.79458	-0.80317	-1.47316	-0.82069	-0.94613
C1orf192	0.150471	-0.13496	-0.15886	-0.12215	-0.53887
C1orf194 KIAA1324	0.46501	0.325838	0.407054	0.509908	0.16559
C1orf198	-0.10651	-0.08289	-0.0818	-0.00357	-0.09306
C1orf2 SCAMP3	0.034794	-0.24882	-0.32814	-0.18012	-0.08456
C1orf201	0.477974	0.627787	0.577829	-0.50795	0.495758
C1orf204	-0.04978	0.019969	0.019389	-0.10491	-0.04968
C1orf21	-0.04382	0.033381	-0.00978	-0.04281	-0.08179
C1orf210	0.391823	0.71347	0.760965	0.437877	0.530258
C1orf212	0.195829	0.074445	0.064361	-0.07393	-0.02519
C1orf216	0.520239	0.382005	0.584759	0.174478	0.400526
C1orf222	0.26505	0.257713	0.503759	0.357435	0.463027
C1orf25 C1orf26	-0.77739	-1.01546	-0.96075	-0.67368	-0.59965
C1orf27	0.587295	0.417283	0.784473	0.170087	0.717407
C1orf31	-0.38737	-0.59095	-0.99925	-0.58309	-0.60238
C1orf32	-0.07912	-0.10589	-0.1378	-0.0512	-0.23167
C1orf35	0.198275	0.110391	0.067249	0.111018	0.242838
C1orf38	0.284718	0.368323	0.113377	0.132578	0.438795
C1orf41 LRRC42	-0.60093	-0.42375	-0.5692	-0.54231	-0.16857
C1orf43 UBAP2L	-0.40014	-0.67131	-0.51003	-0.54737	-0.34269
C1orf49	0.370026	0.498419	0.596707	0.337346	0.338425
C1orf52	-0.00744	0.022549	-0.01921	-0.15376	-0.20168
C1orf53	0.204283	0.217913	-0.05286	-0.01053	0.152833
C1orf54	-0.24218	0.143094	0.230859	-0.23924	-0.10542
C1orf55	0.001973	0.163416	-0.42359	-0.14312	-0.44059
C1orf56	-0.5614	-0.83591	-0.83477	-0.70087	-0.62822
C1orf57	-0.32637	-0.51354	-0.63318	-0.54266	-0.51545
C1orf59	0.502764	0.550473	0.837685	1.00974	0.667212
C1orf61	-0.96951	0.164952	0.310926	0.067779	0.313563
C1orf62 AL449266.17	-0.23572	-0.04029	-0.44841	-1.47282	-0.50137
C1orf63	-0.17558	-0.02617	-0.10209	-0.08713	0.14174
C1orf64	0.458766	0.755776	0.676855	0.415522	0.612374
C1orf65	0.346564	0.083468	0.207817	0.328554	0.102053
C1orf74	-0.03263	-0.33201	-0.44726	-0.13671	0.021678
C1orf76	0.125327	0.216476	-0.16793	0.070519	0.229715

C1orf80 C1orf58	-0.45204	-0.40635	-0.72477	-0.55227	-0.42354
C1orf85	0.568139	0.40489	-0.11487	0.517888	-0.11073
C1orf86	-0.10642	0.103535	-0.01334	-0.01411	-0.02997
C1orf87	0.557482	0.800214	0.791612	0.643048	0.734287
C1orf88	-0.18518	-0.41256	-0.02562	-0.38082	-0.44263
C1orf89	-0.26973	-0.42157	-0.67733	-0.29958	-0.36835
C1orf9	-0.48306	-0.62465	-0.69383	-0.63539	-0.44815
C1orf90	-0.14031	0.01033	0.14315	-0.18283	-0.1251
C1orf91 EIF3I	-0.48324	-0.36897	-0.96068	-0.41147	-0.30654
C1orf92	0.202704	0.692029	0.820474	0.462661	0.428879
C1orf93	0.089302	0.172452	0.053708	0.165547	0.32623
C1orf94	-0.01532	0.006459	0.144508	-0.02767	-0.0547
C1orf95	-0.19876	-0.38701	-0.04257	-0.27207	-0.06527
C1orf96	0.055344	0.371948	-0.12179	0.042546	0.134701
C1orf97	0.20538	-0.39815	-0.58481	-0.43716	-0.33762
C1QBP	0.095852	0.053053	0.087028	-0.06587	0.084801
C1QC	0.259233	0.202336	0.224884	0.040614	0.184602
C1QL1	-0.04732	0.167177	0.233361	-0.17459	0.212692
C1QL2	0.117902	0.328167	0.531593	0.245068	0.376281
C1QL3	-0.14378	0.038791	0.03997	-0.10922	0.194749
C1QL4	0.217994	0.503335	0.461961	0.272521	0.224482
C1QR	0.569936	-0.12455	-0.15755	0.612787	0.217954
C1QTNF1	0.449249	0.602358	0.571871	0.329115	0.470918
C1QTNF2	0.348	0.588984	0.679544	0.235522	0.255691
C1QTNF6	-0.24297	-0.15677	-0.18619	-0.11341	-0.23754
C1QTNF7	0.285164	0.381705	0.476027	0.043976	0.048105
C1QTNF8	0.213164	0.195713	0.462608	0.163245	0.235314
C1QTNF9	0.320275	0.484366	0.359119	0.046105	0.037647
C1RL AC018653.29	0.139587	0.202005	0.198592	0.167152	0.327917
C1S	0.19197	0.044202	0.25815	-0.77221	-0.3392
C2	0.236639	0.563876	0.70618	-0.16848	0.417134
C2 CFB	0.25884	0.470629	0.594645	0.066763	0.520853
C20orf10	0.061644	0.08027	-0.23729	0.137653	0.074956
C20orf100	0.434956	0.132331	0.140618	0.357138	0.692903
C20orf102	0.408738	0.179412	0.417024	0.274812	0.140255
C20orf103	-0.57152	-0.45681	-0.9555	-0.59384	-1.11637
C20orf107	0.207278	0.432323	0.417583	0.29856	0.471235
C20orf108	0.195057	0.118254	0.382707	0.138981	0.015697
C20orf111	-0.34572	-0.28067	-0.29884	-0.23464	-0.38783
C20orf112	-0.41733	-0.40394	-0.36344	-0.24	-0.38757
C20orf114	0.360136	0.230818	0.553853	0.277327	0.358845
C20orf116	0.213718	0.203733	0.316779	0.122832	0.32298
C20orf118	0.357001	0.3167	0.460487	0.224682	0.531515
C20orf12 POLR3F	-0.44485	-0.28802	-0.38021	-0.08253	-0.29499
C20orf121	-0.0873	-0.10283	-0.30698	-0.31465	-0.29719
C20orf132. RPN2	-0.24221	-0.39396	-0.31382	-0.33296	-0.41102
C20orf141	0.036497	0.00097	-0.0639	-0.13845	-0.05876
C20orf149	-0.155	-0.15576	-0.10345	-0.11945	-0.05621
C20orf151	0.329658	0.106176	0.444051	0.230155	0.31474
C20orf152	-0.20755	-0.22735	-0.58475	-0.38898	-0.55618

C20orf160	0.057997	-0.49437	0.163027	0.18387	0.07464
C20orf165	0.735782	0.662332	0.841717	0.553669	0.801155
C20orf169	0.279735	0.051153	0.144239	0.150338	0.071643
C20orf172	-0.06941	-0.10941	-0.46177	-0.13275	-0.21527
C20orf173	-0.30124	-0.02483	0.07777	-0.11885	-0.24774
C20orf175	0.182399	0.491796	0.419912	0.3112	0.412927
C20orf177	-0.17365	-0.15274	-0.22589	0.044974	-0.07391
C20orf179	0.192887	-0.04074	0.189555	0.004615	0.082075
C20orf185	0.192246	-0.9646	-0.81469	0.054557	0.161413
C20orf186	0.196827	-0.48551	-0.46916	0.071673	-0.04046
C20orf19	-0.24121	-0.37785	-0.6213	-0.54454	-0.43713
C20orf191.	-0.23319	-0.97415	-1.02192	-0.93993	-1.08995
C20orf195	-0.13074	-0.08668	0.237468	0.028913	-0.08305
C20orf196	-0.10305	0.271538	-0.12029	0.000266	0.022859
C20orf197	0.198285	0.191975	0.090103	0.162579	0.278772
C20orf198.	0.185136	0.091314	0.049417	0.16716	0.146173
C20orf20	-0.05295	-0.01635	-0.24144	-0.02316	-0.093
C20orf200 C20orf166	0.051453	0.016282	0.18336	0.154765	0.163469
C20orf23	0.311855	0.418099	0.49397	0.317104	0.381278
C20orf27	-0.20688	-0.11364	-0.12884	-0.09482	-0.22562
C20orf28	0.396916	0.516803	0.213872	0.19833	0.356855
C20orf29	0.031967	0.223519	0.360979	0.223428	0.077248
C20orf3	-0.09253	-0.26686	-0.14221	-0.13165	-0.2757
C20orf30	-0.29819	-0.41118	-0.52772	0.057695	-0.30344
C20orf32	0.578088	0.995634	0.913752	0.653982	0.673604
C20orf39	0.057536	0.377872	0.466572	0.212489	0.276107
C20orf4	0.171974	0.271226	0.152781	0.26945	-0.36985
C20orf42	0.013778	0.37625	0.55563	0.152102	0.308415
C20orf43	-0.00382	-0.11547	0.01041	-0.22599	-0.35088
C20orf44	0.099685	0.098494	-0.26721	-0.11746	-0.23734
C20orf45	0.436955	0.432643	0.425579	0.402903	0.39755
C20orf46	0.213872	-0.57172	0.524976	0.289539	0.129923
C20orf54	0.341721	0.591955	0.41546	0.116621	0.196563
C20orf58 ARF1GAP	0.014571	-0.00386	-0.06275	-0.0104	-0.10809
C20orf59	0.167565	0.255974	0.353747	0.258571	0.204704
C20orf6 C20orf7	-0.50528	-0.27391	-0.62726	-0.34654	-0.52328
C20orf70	0.12128	-0.98281	-1.06235	-0.10552	-0.00122
C20orf71	-0.1334	-0.42014	-0.30861	-0.19608	-0.23801
C20orf74	0.092557	0.130898	0.283461	0.148339	0.198036
C20orf75	0.11059	-1.11437	-0.08904	-0.24583	-0.3305
C20orf78	-0.0934	-0.50832	-0.23474	-0.22975	-0.29292
C20orf85	0.458825	0.739685	0.812273	0.63885	0.444361
C20orf91	-0.33309	-0.815	-1.31497	-1.08623	-1.9426
C20orf96	-0.25357	0.144753	0.291919	-0.00308	0.301198
C21orf125	0.293675	0.543565	0.59223	0.503955	0.784679
C21orf13 SH3BGR	0.067937	0.2479	-0.18314	-0.23592	-0.2791
C21orf2	-0.02246	-0.14433	-0.08776	-0.05546	-0.1302
C21orf25	0.537439	0.554753	0.44421	0.308857	0.341333
C21orf33	0.073978	-0.22794	-0.09837	0.055296	0.028012
C21orf45	-0.03061	-0.23555	-0.23118	0.140656	0.030899

C21orf51	0.389133	0.411928	0.310074	0.249109	0.387589
C21orf55	-0.20182	-0.1087	-0.35428	-0.27406	-0.39918
C21orf56	-0.54844	-0.62007	0.368783	-0.81571	-0.70542
C21orf58 PCNT	-0.32035	-0.0307	-0.19195	0.148076	-0.12136
C21orf59	-0.0772	-0.16133	-0.2959	0.002002	-0.1218
C21orf6	0.235274	0.113921	0.248432	0.089821	0.271984
C21orf62	0.034002	0.346169	0.577705	-0.17515	0.138655
C21orf63	0.093953	0.153223	0.228823	0.343489	0.264143
C21orf66 C21orf49	-0.01091	0.04965	0.037149	0.022562	0.036569
C21orf67.	0.241176	0.093047	0.278731	0.167106	0.195055
C21orf67. C21orf70	0.015457	0.060818	0.02795	0.052298	-0.02624
C21orf7	0.334358	0.310853	0.428241	0.193552	-0.23522
C21orf81	0.403025	0.404029	0.508124	0.467963	0.482702
C21orf84	0.168439	0.513496	0.472779	0.341097	0.32937
C21orf91	-0.47198	-0.55296	-0.48397	-0.40417	-0.5575
C21orf93	0.478288	0.724059	0.614972	0.501566	0.65169
C21orf99.	-0.45845	-1.83023	-1.4992	-1.29394	-1.23604
C22orf13 SNRPD3	-0.6937	-0.65368	-0.8294	-0.55978	-0.59905
C22orf15	0.206364	0.270102	0.366427	0.062007	0.325888
C22orf16	-0.11324	0.041458	0.06676	0.050153	0.115068
C22orf23 POLR2F	0.025847	0.151316	-0.16022	-0.18037	-0.21988
C22orf24	-0.1126	-0.01567	-0.11718	-0.03658	-0.02601
C22orf25	0.126894	-0.2485	-0.21752	-0.13012	-0.00315
C22orf26 RP4-695O20__B.10	0.03156	-0.10213	-0.10224	-0.00679	-0.15431
C22orf28	0.255401	0.209141	0.263592	-0.01864	-0.02135
C22orf30	0.44413	0.650746	0.749787	0.491027	0.75643
C22orf31	0.282342	0.22133	0.119988	0.200708	0.114122
C22orf33	-0.47396	-0.00085	-0.06889	-0.15259	-0.30871
C22orf39	0.016536	0.089819	0.032299	-0.32694	-0.29261
C2CD2L	-0.16227	0.054364	-0.17592	-0.09209	-0.09538
C2CD3 PPME1	-0.84364	-0.25776	-0.91176	-0.63148	-0.66578
C2orf10	-0.36783	-0.44308	-0.26083	-0.3681	-0.34882
C2orf18	0.203991	0.256642	0.427201	0.143443	0.14954
C2orf21	-0.04076	-0.21365	-0.07407	-0.15338	-0.26836
C2orf24 C2orf17	-0.19698	-0.33009	-0.46228	-0.2264	-0.39884
C2orf25	0.054939	-0.82657	-1.08422	-0.94058	-0.78058
C2orf27	0.488203	-0.0806	0.09879	-0.11823	-0.22532
C2orf28 CAD	0.418815	0.321061	0.41269	0.201895	0.115757
C2orf29	-0.065	0.01343	0.053731	-0.02236	0.068326
C2orf3	0.565265	0.444985	0.654479	0.210605	0.338557
C2orf32	-0.40469	-0.36948	-0.51398	-0.38385	-0.19767
C2orf33	0.64894	0.903465	1.030847	0.538948	0.688495
C2orf34	-0.37809	-0.40357	-0.43352	-0.49881	-0.56147
C2orf39	0.362019	0.570879	0.62213	0.396873	0.315652
C2orf40	0.239837	0.423964	0.448084	0.449687	0.323261
C2orf42	0.349188	0.498585	0.373576	0.19879	0.387096
C2orf43	0.10572	0.034207	0.071594	0.019793	-0.05785
C2orf44 FKBP1B	0.060583	0.050932	-0.25319	0.103059	-0.16809
C2orf48	0.347855	0.698256	0.734964	0.467429	0.510717
C2orf49	-0.54994	-0.36836	-0.59194	-0.47938	-0.64252

C2orf51	0.31414	0.365445	0.526205	0.177211	0.141742
C2orf52	0.094747	0.085309	-0.25567	0.063172	0.306876
C2orf53	0.124457	0.186444	0.004364	-0.25421	-0.2086
C2orf54	0.314541	0.339985	0.395158	0.164602	0.35102
C2orf55	0.093666	0.202951	0.251923	0.185475	0.265425
C2orf57	0.241913	0.521062	0.137888	0.137901	0.259595
C2orf60 C2orf47	-0.24755	-0.24136	-0.1856	-0.13012	-0.19055
C2orf61	0.410296	0.362159	0.645508	0.438379	0.489152
C2orf62	-0.04746	0.075327	-0.2443	-0.23912	-0.09555
C2orf63 RPS27A	-0.48386	-0.63643	-0.91458	-0.54116	-0.43212
C2orf64 UNC50	-0.32587	-0.26052	-0.29352	-0.28915	-0.02907
C2orf65	0.509008	0.549143	0.713041	0.4623	0.486398
C2orf66	0.453356	0.512118	0.590796	0.05604	0.027095
C2orf67	-0.37618	-0.41915	-0.33588	-0.35318	-0.30494
C2orf7 CCT7	-0.35525	-0.35253	-0.32701	-0.28945	-0.13897
C3	-0.18615	-0.57222	-0.47943	-0.4342	-0.34984
C3AR1	-0.53703	-0.41308	-0.68245	-0.56722	-0.6576
C3orf1	-0.25787	-0.26812	-0.72684	-0.18255	-0.33493
C3orf14	0.441176	0.259585	0.403619	0.204523	0.326221
C3orf15	-0.4733	0.461713	-1.2066	0.262227	0.276744
C3orf17	0.095448	0.346946	0.264047	0.005274	0.008263
C3orf18 HEMK1	0.200194	0.239159	0.211508	0.062001	0.313637
C3orf19	0.095982	0.105976	-0.27561	-0.00638	0.086071
C3orf20	0.580417	0.760268	0.914767	0.343841	0.584942
C3orf21	-0.14269	0.069471	-0.07043	-0.04492	0.006204
C3orf22	0.357011	0.19235	0.137546	0.078116	0.016438
C3orf23	0.142102	0.078689	-0.0088	-0.15081	0.082018
C3orf24	0.486597	0.462475	0.562578	0.29323	0.340184
C3orf25	-0.16759	-0.35279	-0.6545	-0.41393	-0.44569
C3orf27	0.490216	0.531166	0.515309	0.288972	0.495425
C3orf31	0.315899	0.413105	0.315669	0.141929	0.359322
C3orf32	0.123434	-0.01241	-0.16028	0.059045	-0.9653
C3orf33	0.28993	0.159276	-0.07866	0.052316	0.094972
C3orf34 PIGX	0.198575	0.19899	0.101727	0.02215	0.050333
C3orf35	0.349136	0.368026	0.342962	0.135017	0.298278
C3orf37	-0.01761	0.044416	0.017977	-0.02467	-0.0917
C3orf38	-0.31238	-0.37275	-0.3182	-0.25735	-0.13765
C3orf39	0.00927	0.034843	-0.27634	-0.08471	-0.09434
C3orf44	0.385334	0.195332	-0.19991	-0.03258	-0.17
C3orf45	-0.21061	-0.02888	-0.34565	-0.12558	-0.05234
C3orf48.	0.551995	0.609993	0.368946	0.215688	0.312579
C3orf52	0.261425	0.43967	0.159814	0.142606	0.385262
C3orf54	0.054151	0.128163	-0.03821	-0.11534	0.084912
C3orf56	0.488033	0.650034	0.701985	0.60251	0.74163
C3orf58	-0.27336	-0.01549	-0.14427	-0.18832	-0.26889
C3orf59	-0.71257	-0.47406	-0.53695	-0.46035	-0.50432
C3orf62 USP4	0.553375	0.364887	0.82345	0.67905	-0.04794
C3orf63	-0.46656	-0.07987	-0.78574	-0.61027	-0.40594
C3orf64	0.058464	0.127	-0.0731	-0.01857	-0.05753
C3orf67	0.4036	0.211218	0.13559	0.31631	0.113607

C3orf68	0.143248	0.321737	0.123691	0.141062	0.30717
C4A	0.349518	-0.00025	0.249663	-0.34339	0.295252
C4BPA	0.559153	-0.40337	0.329809	0.166426	0.333048
C4BPB	0.069254	-0.61748	-0.49394	-0.50069	-0.25495
C4orf14 POLR2B	0.009054	-0.26714	-0.49881	-0.00319	-0.33204
C4orf15	0.113327	0.214966	-0.01585	0.080236	0.105971
C4orf16	-0.40757	-0.39348	-0.26524	-0.32604	-0.25794
C4orf17	-0.3218	-0.02196	-0.18244	-0.35247	-0.15226
C4orf18	-0.06561	-0.51456	-0.3841	-0.38084	-0.72064
C4orf19	0.466582	0.729623	0.753892	0.434076	0.679943
C4orf21 LARP7	-0.93388	-1.11982	-1.17544	-0.80603	-1.11611
C4orf22	-0.28781	-0.32623	-0.36366	-0.33413	-0.41098
C4orf23	0.465336	0.549282	0.439538	0.342172	0.567765
C4orf26	-0.13962	0.250036	0.236715	0.166563	-0.2033
C4orf27	0.216446	0.171903	0.115441	-0.01414	-0.07293
C4orf28	-0.48853	-0.6371	-0.73399	-0.60107	-0.74982
C4orf30 HCAP-G	-0.73936	-0.62082	-0.64712	-0.42739	-0.52899
C4orf31	-0.45247	0.164107	-0.4436	-0.44527	-0.1427
C4orf32	0.061185	0.128484	0.057976	0.094208	0.113359
C4orf34	0.0073	-0.41853	-0.32281	0.04381	0.044495
C4orf35	0.067483	-0.78851	-0.5168	-0.51648	-0.23476
C4orf36	0.275521	0.299022	0.163426	0.11374	0.089192
C4orf37	0.211106	0.061209	-0.24834	-0.17349	0.062669
C4orf40	-0.04256	-0.59333	-0.30614	-0.27236	-0.20157
C4orf6	-0.29311	-0.42162	-0.23472	-0.13982	-0.37626
C4orf7	-0.07871	-0.64371	0.081247	-0.39832	0.149207
C4orf8	0.575503	0.744441	0.788935	0.636245	0.623002
C5	0.250836	0.172253	-0.20666	-0.28903	-0.17384
C5AR1	0.297105	-0.71148	0.144747	0.448882	-0.06576
C5orf14	0.145976	0.38325	0.219475	0.047194	0.050643
C5orf15	0.344848	0.000292	0.161656	0.187562	0.125826
C5orf20	0.015498	0.256901	0.20895	0.217868	-0.03082
C5orf22	-0.55005	-0.80761	-1.13271	-0.68543	-0.74257
C5orf23	-0.50972	-0.6644	-0.74672	-0.32445	-0.75254
C5orf24	0.11537	0.128989	0.23602	0.300838	-0.10183
C5orf25	0.280228	0.091153	0.210733	0.222343	0.314617
C5orf28	-0.24189	-0.40491	-0.50627	-0.31279	-0.44026
C5orf3 MFAP3	-0.6725	-0.76469	-0.93446	-0.8438	-0.97622
C5orf30	0.11761	0.042724	0.09929	-0.06169	0.115371
C5orf32	-0.08254	-0.00666	0.027683	-0.07973	0.036636
C5orf33	0.126779	0.177629	0.114357	-0.06302	0.088994
C5orf34	-0.70959	-0.69177	-0.77047	-0.68034	-0.7813
C5orf35	0.230284	0.15618	0.218088	0.05419	0.174812
C5orf36 BRCTD1.	-0.06104	-0.10623	-0.21138	-0.07587	-0.20787
C5orf39	-0.73223	-0.60291	-0.3947	-0.23673	-0.38974
C5orf41	-0.47057	-0.57599	-0.72519	-0.45692	-0.70453
C5orf5	-0.37029	-0.03348	-0.55894	-0.12329	-0.14632
C6	-0.42722	-0.31873	-0.49863	-0.31473	-0.46871
C6orf1	-0.0731	-0.07603	-0.28029	-0.09277	-0.17692
C6orf10	0.117131	-0.53278	-0.69716	-0.50944	-0.61752

C6orf105	0.531546	0.497519	0.626138	0.432287	0.259558
C6orf106	0.226551	-0.11436	0.118219	-0.13705	-0.2907
C6orf108	0.205078	0.306551	0.375277	0.299356	0.364625
C6orf117	-0.03764	-0.25574	-0.27463	-0.29546	-0.40562
C6orf118	0.09789	0.054755	-0.12199	-0.02562	-0.04609
C6orf123	0.283126	0.446658	0.35005	0.284005	0.442037
C6orf124 MLLT4	-0.11851	-0.01739	0.040452	0.063878	0.02917
C6orf125	0.245781	0.191874	0.335868	0.171725	0.096965
C6orf126	0.200143	0.267488	0.236761	0.128107	0.216843
C6orf128 TBC1D22B	-0.16672	0.215356	0.061377	-0.13082	-0.43053
C6orf129	-0.17287	0.008936	-0.41871	-0.2007	-0.20134
C6orf130 NFYA	-0.38867	-0.32831	-0.33387	-0.29454	-0.30064
C6orf134 C6orf136	0.098908	0.142849	0.160539	0.122988	0.033786
C6orf141.	-0.00943	0.501108	0.666758	0.501112	0.365695
C6orf142	0.194022	-0.03272	-0.44956	-0.24177	-0.55458
C6orf145	-0.07102	0.151148	-0.02447	0.003733	0.072239
C6orf146 C6orf201	0.162022	0.279321	0.359052	0.209203	0.15632
C6orf148	0.264251	0.114653	0.09962	0.269455	0.26755
C6orf15 PSORS1C1	-0.08721	-0.50514	-0.3114	-0.55399	-0.35802
C6orf150	0.267569	0.541037	0.563894	0.388228	0.263911
C6orf151	0.057422	0.033455	-0.15409	-0.16816	-0.12168
C6orf154	0.191961	0.319642	0.455137	0.192897	0.240087
C6orf162	0.280991	0.153848	0.346863	0.128216	0.37307
C6orf163	0.709912	0.799928	0.750935	0.283996	0.432639
C6orf165	-0.05835	-0.14314	-0.22723	-0.29171	-0.32704
C6orf166	-0.28461	-0.16297	-0.30792	-0.21141	-0.37305
C6orf167	-0.07336	-0.26651	-0.36607	-0.27965	-0.30619
C6orf168	0.103839	0.046238	0.057036	0.031244	0.070363
C6orf170	0.193542	0.205519	-0.10656	-0.13247	-0.28857
C6orf173	0.191027	0.083309	-0.34961	-0.44772	-0.74747
C6orf174	-0.69215	-0.42635	-0.55555	-0.56502	-0.44757
C6orf182	0.689519	0.599913	0.538642	0.355726	0.257037
C6orf190	-0.51472	-0.24067	-1.01041	-0.76577	-0.76288
C6orf191	0.547943	-0.31247	-0.10417	-0.21529	0.165653
C6orf192	-0.00166	-0.23106	-0.37119	0.006366	-0.50518
C6orf195	0.009244	0.135221	-0.04531	0.009373	-0.10159
C6orf199 FIG4	-0.27891	-0.11734	-0.34872	-0.26478	-0.4472
C6orf203	0.063452	-0.01361	0.135976	0.044949	0.018291
C6orf205	0.234642	-0.82524	-1.0509	-0.53305	-0.44316
C6orf206	0.267382	0.405975	0.17492	0.396479	0.133374
C6orf218	0.325195	-0.74674	-0.21753	0.29505	-0.3623
C6orf221	0.419904	0.54915	0.476032	0.352329	0.510132
C6orf222	0.346301	0.143137	0.341502	0.07542	0.171881
C6orf223	0.393608	0.34521	0.453258	0.306609	0.281563
C6orf224	0.120021	-0.0824	-0.32383	-0.37258	-0.83657
C6orf25	0.461904	0.654597	0.679749	0.426783	0.423563
C6orf27	-0.10111	-0.15362	-0.13087	0.031391	-0.15073
C6orf32	0.22889	0.398502	0.259568	0.00956	-0.00601
C6orf47 BAT4	0.0444	-0.02943	-0.08833	-0.03043	-0.20536
C6orf48	-0.12944	-0.17665	-0.407	-0.25738	-0.27017

C6orf51	0.282675	0.230767	0.254285	0.136928	0.130639
C6orf57	0.292006	0.163206	0.233267	0.021778	-0.0279
C6orf58	-0.12921	-0.27746	-0.44178	-0.35785	-0.57249
C6orf59.	0.034589	-0.14263	-0.12284	-0.16382	-0.33957
C6orf60	0.036386	0.130895	-0.11851	-0.20703	0.139506
C6orf62	-0.4402	-0.82137	-0.81281	-0.56352	-0.82042
C6orf64	0.242473	0.355428	0.316778	0.21582	0.384449
C6orf65	-0.00453	-0.01941	0.049814	-0.02737	-0.08086
C6orf66	-0.55172	-0.3427	-0.36768	-0.36088	-0.35715
C6orf70	-0.1564	0.036583	-0.20255	-0.14436	-0.07721
C6orf72	-0.26332	0.041607	-0.21265	-0.10263	-0.11177
C6orf81	0.194939	0.597517	0.246683	0.218933	0.281526
C6orf89	0.023177	0.193659	-0.10171	-0.04494	-0.21807
C6orf97	0.487053	0.255697	0.191642	0.104004	0.295365
C7	0.098348	-0.07149	0.092586	-0.08542	-0.05829
C7orf11 C7orf10.	0.151175	-0.01782	0.024907	0.135429	0.183696
C7orf16	0.373191	-0.29028	-0.87042	-0.19212	-0.27789
C7orf20	0.129992	0.353437	0.421499	0.156473	0.254013
C7orf23	-0.31596	0.001509	-0.32536	-0.17195	0.027493
C7orf24	0.193295	-0.38131	-0.59057	0.030835	-0.02121
C7orf25	-0.13386	-0.07393	-0.12195	-0.24222	-0.17733
C7orf26	0.148606	0.304123	0.452342	0.232246	0.267946
C7orf27	0.151473	-0.04208	0.04024	-0.0185	0.085614
C7orf28A	0.109084	-0.38522	-0.3827	-0.10459	-0.05857
C7orf28B	0.013683	0.062885	-0.21449	0.062572	0.214543
C7orf30	-0.01593	-0.52749	0.071007	0.050243	0.036467
C7orf31	0.181747	0.080147	-0.00191	-0.06798	0.031798
C7orf33	0.264853	0.337637	0.562899	-0.00973	-0.05593
C7orf34	0.337123	0.418947	-0.20248	0.375355	0.119335
C7orf36	-0.69347	-0.87249	-1.16506	-0.78537	-0.49635
C7orf41	0.720877	0.626757	0.713425	0.280571	0.529172
C7orf42	-0.10324	-0.11029	-0.07142	0.029797	-0.27784
C7orf43 GAL3ST4	0.183163	0.115196	0.036797	0.160742	0.202974
C7orf44	-0.40546	-0.55164	-0.89265	-0.47499	-0.82587
C7orf46	0.436776	0.227632	-0.13397	-0.19354	0.557974
C7orf47	0.070078	0.106449	0.139227	0.016123	0.014809
C7orf49	0.193182	0.285229	0.275053	0.180969	0.121508
C7orf51	-0.06774	0.010479	-0.17242	0.092173	0.096347
C7orf52	0.006573	-0.23072	0.1292	0.009409	0.164458
C7orf53	0.365884	-0.32197	0.027737	0.204009	-0.47781
C7orf55	-0.85965	-0.79357	-1.52404	-0.68617	-0.92004
C7orf58	-0.23273	-0.49698	-0.65571	-0.27713	-0.54798
C7orf9	0.081088	-0.00392	-0.08248	-0.36315	-0.3734
C8A	-0.30924	-0.68618	-0.53422	-0.69782	-0.29343
C8B	-0.29559	-0.25337	-0.43121	-0.4822	-0.03263
C8orf13	0.143524	-0.03666	0.052872	0.079001	0.107128
C8orf22	0.6042	0.273666	0.313121	0.508938	0.269023
C8orf30A	0.165989	-0.1449	0.07777	0.03187	0.131549
C8orf31	0.306312	-0.53616	-0.5193	-0.30051	-0.31673
C8orf32	0.064437	0.11499	-0.06597	0.076041	0.214175

C8orf33	-0.17755	-0.3106	-0.17737	0.081421	0.084279
C8orf34	-0.16448	-0.34205	-0.1791	-0.39423	-0.40105
C8orf37	-0.56044	-0.71747	-0.87875	-0.538	-0.53389
C8orf4	0.767114	1.013841	1.290084	0.664718	0.71444
C8orf41	-0.62834	-0.46749	-0.52841	-0.68023	-0.941
C8orf42	-0.31454	-0.2857	-0.33755	-0.20594	-0.09991
C8orf45	0.546246	0.459999	0.605082	-0.07884	0.323514
C8orf46	0.323532	0.716812	0.863341	0.524152	0.625817
C8orf47	0.201876	0.319418	0.52545	0.38563	0.160848
C8orf53	0.052862	-0.09263	-0.12456	-0.22037	-0.45629
C8orf55	0.155092	0.024827	0.129148	0.162461	0.345177
C8orf58	-0.23263	-0.42094	-0.24737	-0.21096	-0.30203
C8orf58 KIAA1967	-0.07152	-0.22205	0.050809	-0.03431	-0.26566
C8orf70	0.132541	-0.04741	-0.06997	-0.05466	-0.19055
C8orf76	0.213489	0.024527	0.071755	0.106696	0.047971
C9	0.348104	-0.09447	-0.11971	-0.2474	-0.21296
C9orf100	0.155145	-0.10071	-0.10022	-0.02751	-0.0622
C9orf103	0.352317	0.462594	0.482021	0.206802	0.285629
C9orf106	0.189083	0.176515	0.124685	0.163295	0.066584
C9orf11	0.565142	0.589299	0.370836	0.254319	0.487923
C9orf114	0.362349	0.32397	0.265587	0.18888	0.345399
C9orf116 MRPS2	0.06683	0.23358	0.203487	0.14842	0.234293
C9orf123	0.126523	0.312768	0.103969	0.087647	0.056427
C9orf125	0.365218	0.396105	0.197586	0.020929	0.134362
C9orf125 RNF20	-0.40267	-0.33459	-0.57471	-0.14045	-0.40056
C9orf126	-0.35211	-0.39156	-0.7899	-0.37537	-0.57654
C9orf127	-0.39877	-0.38889	-0.40028	0.332763	-0.49546
C9orf131	0.014479	0.261945	0.002386	-0.18141	-0.11631
C9orf135	0.568091	0.615804	0.80836	0.547264	0.641046
C9orf139	0.251021	0.044218	0.533806	0.15475	0.584067
C9orf140	0.110715	0.174545	0.102333	0.122525	0.052248
C9orf142	0.04572	0.243236	0.289592	0.203625	0.115651
C9orf150	-0.58927	-0.25224	-0.55776	-0.43311	-0.48101
C9orf152	-0.10804	-0.199	0.162145	-0.08755	-0.73297
C9orf153	0.161501	0.274505	0.197717	0.038946	0.401509
C9orf156	0.189763	-0.27296	-0.04471	-0.07028	-0.32346
C9orf16	0.017039	-0.09781	-0.23447	-0.13981	-0.12526
C9orf163	-0.34843	-0.33811	-0.33345	-0.30846	-0.29706
C9orf165	0.10766	0.348264	0.211067	0.196396	0.235493
C9orf167	-0.13134	-0.07432	0.232494	0.181556	0.118062
C9orf19	-0.04014	0.089285	0.067263	0.041726	0.117605
C9orf21	-0.09846	-0.05108	0.067499	0.108324	0.088799
C9orf23	-0.38638	-0.41504	-0.41685	-0.09212	-0.42213
C9orf24	0.390384	0.622488	0.625062	0.41978	0.544088
C9orf24 C9orf25	-0.11091	-0.20151	-0.33173	-0.3875	-0.14319
C9orf25 DNAI1	-0.20045	-0.10314	-0.10936	-0.03396	-0.09602
C9orf27.	0.586402	0.467032	0.462517	0.329179	-1.92507
C9orf29	-0.17003	-0.47444	-0.6322	-0.5564	-1.28155
C9orf29 GNG10	-0.20957	-0.16367	-0.33537	-0.33035	-0.34738
C9orf3	0.163742	0.228526	0.259029	0.204223	0.096651

C9orf37	-0.18225	-0.29903	-0.17139	-0.09322	-0.14325
C9orf4	0.22896	0.356203	0.383484	0.106339	0.329429
C9orf40	0.267875	0.165195	0.041701	-0.06501	0.174154
C9orf41	0.204431	0.358369	0.176064	0.085232	0.265547
C9orf46	0.030856	0.118481	-0.04379	0.025929	0.114236
C9orf47	0.019349	0.441513	0.121255	0.301492	0.342757
C9orf48 NUDT2	-0.95224	-0.65292	-0.96027	-0.61069	-0.69074
C9orf5	0.113964	0.099068	0.215156	0.151388	0.308313
C9orf52	0.067759	-0.04212	-0.07472	-0.04259	-0.23691
C9orf61	0.203664	-0.47508	0.057726	-0.39645	-0.59204
C9orf64	0.620974	-0.21348	-0.14983	-0.19598	0.016069
C9orf66	0.27515	0.008054	0.085109	0.113977	0.024661
C9orf71	-0.27963	-1.40386	-1.25479	-0.18182	-0.84694
C9orf72	-0.34101	-0.67811	-1.04474	-0.58706	-0.56836
C9orf75	0.028289	0.186768	0.327668	0.113807	0.158005
C9orf78 USP20	0.016897	-0.22246	-0.26374	-0.14613	-0.07641
C9orf79	0.246475	0.523251	0.428132	0.194632	0.186981
C9orf80	-0.0114	-0.05532	-0.60247	-0.18945	-0.16097
C9orf82	-0.03847	-0.38577	-0.51867	-0.42296	-0.34132
C9orf84	0.655475	0.86725	0.848097	0.437114	0.480229
C9orf89	-0.09864	-0.28952	-0.20495	-0.21605	-0.07508
C9orf90 SLC25A25	0.035322	0.079127	0.143789	-0.10061	-0.1246
C9orf91	0.256758	0.491439	0.682399	0.177056	0.331223
C9orf93	-0.36282	-0.12972	-0.50379	-0.35139	-0.5601
C9orf95 OSTF1	-0.17922	-0.01356	-0.1039	0.009053	-0.08636
C9orf97 NCBP1	-0.30149	-0.41314	-0.40404	-0.35323	-0.4887
C9orf98 C9orf9	0.182982	0.343247	0.304918	0.213927	0.11658
CA10	-0.22451	-0.04602	-0.16252	-0.2636	-0.22257
CA11	-0.51037	-0.74668	-0.72804	-0.58122	-0.66367
CA12	0.312065	0.150736	0.152266	0.121338	0.177528
CA13	-0.14036	-0.66641	-0.04587	-0.08205	-0.17205
CA2	-0.10164	-0.11452	0.082639	0.123302	0.004965
CA3	0.294512	-0.56854	-0.51704	0.028914	-0.36318
CA4	0.028558	0.298385	0.299347	0.36124	0.393546
CA5A	0.62263	0.793768	0.977592	0.572622	0.975091
CA5B	0.098022	0.177271	-0.82072	0.444721	-0.41794
CA6	0.491316	0.631236	0.556376	0.259011	0.423154
CA7	-0.17024	0.300874	0.270262	0.167727	0.087561
CA8	0.131383	0.334832	0.389179	0.185411	0.199196
CA9	0.139983	0.087589	0.139964	-0.0376	0.153993
CAB39	0.019138	0.026314	0.078476	0.0253	0.053797
CABC1	-0.04285	-0.11241	-0.0617	-0.07323	-0.01042
CABLES1	0.246004	0.312506	0.175446	0.184546	-0.05111
CABLES2	0.180871	0.281994	0.303971	0.186623	0.298596
CABP2	0.069876	0.129815	0.211879	0.054225	0.083944
CABP5	0.07963	-0.49444	-0.69072	-0.31183	-0.22278
CABP7	0.144545	0.301107	0.304548	0.270938	0.169603
CABYR	0.29424	0.413047	0.338535	0.356475	0.230217
CACHD1	0.116228	0.098784	-0.50596	-0.6405	-0.34627
CACNA1A	-0.27348	-0.57681	-0.24905	0.325096	0.063424

CACNA1C	-0.13909	-0.13571	-0.13433	-0.20289	-0.12332
CACNA1D	0.022432	0.093355	0.297358	0.054073	0.097323
CACNA1E	-0.14333	-0.15166	0.237671	0.398945	0.426336
CACNA1F AF235097.12 CCDC22	0.304054	0.347518	0.158055	0.110727	0.347517
CACNA1G	-0.16905	-0.1679	-0.20127	-0.06689	-0.00748
CACNA1H	-0.005	0.161534	0.090811	0.09529	0.130624
CACNA1I	0.05411	0.015141	0.027339	-0.12127	0.065344
CACNA1S	-0.07411	-0.20329	-0.24729	-0.56921	-0.26956
CACNA2D1	-0.24679	-0.30476	-0.57597	-0.23892	-0.17776
CACNA2D2	0.072043	0.149789	0.219274	0.122287	0.243039
CACNB1	-0.37528	-0.34164	-0.29005	-0.34564	-0.35389
CACNB3	-0.22833	-0.3236	-0.3819	-0.25759	-0.129
CACNG1	0.514636	0.552528	0.652523	0.505225	0.639319
CACNG2	-0.07506	-0.21783	-0.41021	-0.25176	-0.44461
CACNG3	-0.03508	-0.02676	-0.24122	0.151275	0.122461
CACNG4	-0.19141	-0.07968	0.005067	-0.19323	-0.10669
CACNG5	0.197843	0.35454	0.424332	0.100432	0.102758
CACNG6	0.132299	-0.09069	0.399952	0.21233	0.376043
CACNG7	0.155783	-0.00426	0.43626	0.311451	0.228753
CACNG8	-0.0109	-0.7741	0.340728	0.234139	0.00483
CACYBP	-0.32501	-0.59922	0.257532	0.043772	0.230643
CADM1	-0.37986	-0.12985	-0.42616	-0.28484	-0.24246
CADM2	-0.4024	-0.36885	-0.34087	-0.29488	-0.41738
CADM3	-0.09542	0.368141	-0.50002	-0.37826	-0.36845
CADM4	-0.15904	-0.35472	-0.66354	-0.192	0.13136
CADPS	-0.42824	0.12434	-0.08318	-0.35607	-0.16458
CAGE1 RIOK1	-0.60207	-0.47925	-0.55274	-0.41772	-0.39698
CALB1	-0.37835	-0.24105	-0.52771	-0.58994	-0.67036
CALB2	0.133479	-0.11654	-0.01231	-0.06521	-0.13528
CALCOCO1	-1.03212	-0.61302	-1.06254	-0.67236	-0.97558
CALCOCO2	0.023362	0.024998	-0.02443	0.215458	-0.0905
CALCR	-0.09738	0.089908	0.471366	0.135813	-0.07245
CALCRL	-0.13081	-0.30708	-0.47465	-0.41185	-0.13754
CALM2	-0.28586	-0.31945	-0.47464	-0.1799	-0.41814
CALM3	-0.16828	-0.05542	-0.31117	-0.14372	-0.34525
CALML3	0.160039	0.376887	0.445871	0.289253	0.33805
CALML4	0.291146	0.467746	0.593367	0.254854	0.511123
CALML5	0.357859	0.458379	0.459691	0.302352	0.18869
CALML6	0.02507	0.340952	0.356821	0.224085	0.360678
CALR	-0.2216	-0.0638	-0.11514	-0.14701	-0.12432
CALR3 C19orf44	0.226812	0.231589	-0.01631	0.296897	-0.14389
CALU	-0.30254	-0.61054	-0.97382	-0.55823	-0.80354
CAMK1	0.035624	-0.1342	-0.07481	0.043667	-0.13743
CAMK1D	-0.18526	-0.02924	-0.20743	-0.07809	-0.04145
CAMK1G	-0.48274	-0.14888	-0.2646	-0.26509	-0.4639
CAMK2A	0.074285	0.076746	0.436333	0.074062	0.156345
CAMK2B	0.008284	-0.18196	-0.00845	-0.06401	0.044871
CAMK2D	-0.20647	-0.38362	-0.23659	-0.07587	-0.07023
CAMK2G	-0.12053	-0.07972	-0.17392	-0.03711	0.021177
CAMK2N1	-0.37659	-0.27276	-0.287	-0.14134	-0.09876

CAMK4	0.064522	0.050189	0.20159	0.097054	-0.07565
CAMKK1	0.092479	0.15249	-0.02762	-0.18306	-0.18199
CAMKK2	0.115402	0.05816	0.042183	0.007128	0.017415
CAMKV	-0.4846	-0.02852	-0.31391	-0.37911	-0.36728
CAMLG	-0.25544	0.031583	-0.13976	-0.14716	-0.27461
CAMP	0.380145	0.259876	0.463889	0.356283	0.157505
CAMSAP1	0.227072	0.566724	0.454626	0.404995	0.668819
CAMSAP1L1	-0.39297	-0.37458	-0.51058	-0.33539	-0.32534
CAMTA1	-0.12436	-0.03511	-0.22709	-0.1012	-0.00266
CAMTA2 AC004771.1	0.02989	0.1392	-0.10161	-0.10959	0.215882
CAND1	-0.55554	-0.59948	-0.56612	-0.45214	-0.56397
CANT1	0.321769	0.054206	0.131406	0.054115	0.322437
CANX	-0.06779	-0.01488	-0.07198	0.007988	0.008784
CAP1	-0.3087	-0.37558	-0.41035	-0.28456	-0.10179
CAP2	-0.36905	-0.14286	-0.49768	-0.46862	-0.2677
CAPG	0.389125	0.441686	0.80586	0.436202	0.516989
CAPN1	-0.29852	-0.39285	-0.33488	-0.2155	-0.19964
CAPN10	-0.08649	-0.05563	-0.17091	-0.12479	-0.21856
CAPN11	0.053022	-0.01562	-0.19348	-0.09088	-0.09206
CAPN12	0.317688	0.10055	0.350564	0.096342	0.361451
CAPN13	-0.10494	-0.60198	0.198205	0.043609	0.011192
CAPN2	0.291058	0.298762	0.379248	0.260661	0.256767
CAPN6	-0.21836	0.615701	0.07807	0.350512	0.364952
CAPN7	0.073453	0.1779	0.022397	-0.10988	0.182063
CAPN9	0.446717	0.398769	1.149804	0.389775	0.582468
CAPNS1	-0.04644	0.02024	0.103803	0.08309	0.176795
CAPRIN1	-0.02099	0.017163	-0.11548	0.020315	-0.01033
CAPRIN2	-0.24449	-0.28518	-0.51142	-0.22029	-0.25608
CAPS	0.076209	0.100806	0.169968	0.077452	0.101632
CAPS2	0.257227	-0.2194	-0.20435	0.095014	-0.49682
CAPS2 GLIPR1L2	0.262119	-0.57561	-0.587	-0.01642	-0.35423
CAPSL	-0.447	-0.92004	-0.60923	-0.56347	-0.79109
CAPZA2	0.019336	0.249001	0.289557	0.149903	0.057053
CAPZB	0.244246	0.475672	0.689405	0.274841	0.551704
CARD10	0.118967	0.210742	0.401433	0.286096	0.26508
CARD11	0.166101	0.377073	0.368304	0.142707	0.115869
CARD15	0.281769	0.434811	0.678278	0.406976	0.31627
CARD4	0.070048	-0.16527	-0.23627	-0.04689	0.05384
CARD6	0.271484	0.444459	0.68051	-0.00438	-0.03614
CARD8	0.277707	0.747894	0.594044	0.355848	0.414328
CARD9	0.355262	0.446005	0.444437	0.351039	0.516645
CARKL	0.330961	0.276613	0.580518	0.372022	0.365585
CARKL CTNS	0.109863	0.085774	-0.1365	0.03737	0.101466
CARM1	-0.20121	-0.36461	-0.37157	-0.29308	-0.18658
CARS	-0.08138	-0.2278	-0.34635	-0.19033	-0.00588
CARS2	-0.05538	-0.13698	-0.21159	-0.19301	-0.27043
CARTPT	0.045724	-0.04728	0.231274	0.475798	0.213951
CAS1	0.002452	0.240169	0.159852	0.213981	0.124884
CASC1 LYRM5	-0.45543	-0.49167	-1.01348	-0.6812	-0.91409
CASC3	0.172366	-0.18071	-0.34706	-0.1865	-0.24101

CASC4	-0.28669	-0.14986	-0.25925	-0.23665	-0.36797
CASC5	-0.78723	-0.85446	-1.24757	-0.4955	-1.19144
CASKIN1	-0.01806	-0.09501	0.018765	0.024699	0.111908
CASKIN2 TSEN54	-0.12157	-0.25502	-0.05601	-0.23449	-0.12086
CASP10	0.5227	1.002625	1.000698	0.647949	0.669913
CASP14	-0.17047	-1.12063	-1.20271	-0.80602	-1.07394
CASP3 CCDC111	-0.122	-0.19084	-0.19008	-0.15293	0.062512
CASP4	0.288663	-0.09023	0.307879	0.275066	0.17313
CASP5	0.097785	0.309611	0.329404	-0.01179	0.204877
CASP6	-0.01929	-0.29944	-0.04833	-0.03136	-0.0142
CASP7	0.013515	-0.06812	0.072718	0.038519	-0.15399
CASP8AP2	-0.49736	-0.65396	-1.21946	-0.7449	-0.82511
CASP9 DNAJC16	-0.49654	-0.43656	-0.53091	-0.17142	-0.21814
CASQ1	0.231535	0.170778	0.416548	0.104026	0.273471
CASQ2	0.563305	0.693496	0.824107	0.621245	0.597164
CAT	-0.34462	-0.37106	-0.32572	-0.29168	-0.07582
CATSPER1	0.640948	0.516959	0.764586	0.315517	0.509964
CATSPER3	0.436163	0.45668	0.591774	0.308295	0.504725
CATSPER4	0.124836	0.184172	0.244543	0.199653	0.160352
CATSPERB	-0.19485	-0.41262	-0.38553	-0.39714	-0.39624
CAV1	-0.55947	-0.36973	-0.18662	-0.03777	-0.36845
CAV2	0.140723	0.191405	0.277002	0.212513	0.220638
CAV3	0.361371	0.440684	0.391973	0.305085	0.430469
CBARA1	-0.58407	-0.56447	-0.84008	-0.61083	-0.8395
CBFB	-0.0082	0.089365	0.129757	-0.03154	0.141055
CBL	-0.21557	-0.01714	-0.34673	-0.12472	-0.34851
CBLB	-0.28005	-0.44598	-0.37456	-0.21359	-0.29735
CBLC	0.199461	-0.01213	-0.03735	0.044251	0.183165
CBLL1	-0.10911	-0.20015	-0.58662	-0.264	-0.39884
CBLN2	-0.04287	-0.05111	-0.06508	-0.10974	0.061954
CBLN3 KIAA0323	0.136036	-0.05581	-0.20708	0.089363	0.080992
CBLN4	-0.08322	-0.02199	-0.24192	0.423106	0.225498
CBR3	-0.09054	0.304542	0.383309	0.193627	0.213346
CBR4	-0.49084	-0.71249	-0.70201	-0.44993	-0.63961
CBS	0.068194	-0.12475	-0.25032	-0.17427	-0.19627
CBWD1	-0.31319	-0.77533	-0.83761	-0.67823	-0.98364
CBWD2	-0.59182	-0.81635	-0.96787	-0.81178	-1.28899
CBWD3	-0.50494	-1.11737	-1.00587	-0.8181	-1.28886
CBWD5	-0.37794	-0.54709	-0.7809	-0.62023	-0.84154
CBX1	-0.09334	-0.26269	-0.29248	-0.27335	-0.15649
CBX2	-0.06058	-0.2473	-0.03397	-0.18983	0.047513
CBX4	-0.1855	-0.30096	-0.44123	-0.19406	-0.22108
CBX5 HNRNPA1	-2.04203	-1.98577	-2.28965	-1.76063	-1.94367
CBX7	0.187285	0.300178	0.188238	0.281368	0.170448
CBX8	-0.43963	-0.6038	-0.69806	-0.53135	-0.36564
CC2D1B	0.163032	0.220723	0.200114	0.152806	0.108494
CCAR1	0.029011	0.030439	0.012832	-0.06056	-0.09504
CCBE1	-0.00493	0.287971	0.613806	0.252503	0.289962
CCBL1 LRRC8A	-0.29336	-0.39203	-0.51277	-0.46669	-0.42462
CCBL2	-0.37885	-0.60675	-0.72951	-0.89361	-0.57558

CCDC101	0.23217	0.154036	0.079828	0.061271	0.149456
CCDC102A	0.218496	0.39642	0.490745	0.193548	0.436299
CCDC102B	0.649873	0.855605	0.826651	0.50375	0.535639
CCDC104	-0.49974	-0.36006	-0.81376	-0.35817	-0.40716
CCDC105	0.19988	-0.01064	0.335231	0.302468	0.277634
CCDC106	-0.11673	-0.09013	-0.31438	0.047041	-0.18096
CCDC108	0.925597	0.276726	0.294861	0.101356	-1.18338
CCDC109A	0.387373	0.338623	0.370608	0.182534	0.135518
CCDC109B	-0.15397	-0.08512	-0.29253	0.077698	0.047986
CCDC11	0.284751	0.179347	0.062667	0.13941	0.233123
CCDC110	0.225196	0.308988	0.135962	0.025934	0.084148
CCDC112	-0.17525	-0.37692	-0.45936	-0.44052	-0.38703
CCDC113	0.183977	0.270653	0.228627	0.098089	-0.1877
CCDC114	0.481911	0.014131	0.732879	0.499351	0.469293
CCDC115 IMP4	-0.42076	-0.30407	-0.50088	-0.30299	-0.10854
CCDC116	0.438827	0.549004	0.688956	0.493263	0.418746
CCDC117	0.283972	0.2075	0.294796	0.231261	0.124623
CCDC12	-0.09193	0.021695	-0.53644	-0.08019	-0.13393
CCDC120	-0.13902	0.207367	0.017127	0.003965	-0.01246
CCDC121 XAB1	-0.35893	-0.35498	-0.7479	-0.55921	-0.65958
CCDC123 C19orf40	0.197094	-0.28448	-0.47142	-0.24055	-0.48004
CCDC125	0.332961	0.210704	0.302942	0.13338	-0.03327
CCDC126	0.469366	0.451837	0.474749	0.227537	0.281435
CCDC127 SDHA	-0.0725	0.188618	0.026132	0.06015	0.096681
CCDC128	-0.58074	-0.56414	-0.50232	-0.44349	-0.26464
CCDC129	0.461611	0.679732	0.222425	0.513112	0.497966
CCDC130	-0.06856	0.04546	0.01937	0.016165	0.017542
CCDC131 THAP2	-1.11838	-0.91733	-1.04809	-0.93703	-0.93794
CCDC132	-0.88619	-0.92994	-1.05957	-1.00903	-0.80961
CCDC134	0.057288	0.207113	0.316986	0.251702	0.105481
CCDC135	0.407651	0.443072	0.383324	0.061815	0.352971
CCDC136	-0.35947	-0.30458	-0.61226	-0.38002	-0.50615
CCDC138	-0.20027	-0.16714	-0.33001	-0.08909	-0.00761
CCDC14	-0.08484	-0.05233	-0.22759	-0.15177	-0.28397
CCDC141	0.263243	-0.43276	0.186504	-0.58772	-0.28186
CCDC142 TTC31	-0.28146	-0.36211	-0.51143	-0.30276	-0.2249
CCDC144B	0.522276	0.494024	0.695377	0.199827	0.269842
CCDC146	0.453645	0.795407	1.002145	0.195818	-0.10324
CCDC147	0.37904	0.643079	0.076342	0.28672	-0.658
CCDC148 PKP4	-0.1374	-0.30914	-0.2112	-0.24777	-0.04383
CCDC149	0.282946	0.40302	-0.13382	0.245976	0.297217
CCDC15	0.080856	-0.03056	-0.45413	-0.11385	0.112537
CCDC151 PRKCSH	-0.154	-0.28776	-0.31101	-0.12597	-0.27974
CCDC17	0.541985	0.329762	0.653295	0.423689	0.562788
CCDC19	0.138214	0.525456	0.570493	0.236998	-0.11341
CCDC21	-0.02155	0.037435	0.104934	-0.08856	-0.12733
CCDC23 ERMAP	-0.15523	-0.30474	-0.41698	-0.23631	-0.06284
CCDC25 ESCO2	-0.14063	-0.25276	-0.19727	-0.32304	-0.31303
CCDC27	0.272219	0.300251	0.667235	0.234431	0.445076
CCDC28A	-0.39268	-0.40359	-0.7929	-0.66818	-0.94701

CCDC28B	-0.57872	-0.52361	-0.7504	-0.58056	-0.34341
CCDC28B IQCC	0.084747	-0.00831	-0.09785	-0.01038	0.087391
CCDC3	0.114592	0.0129	0.209299	0.003613	0.14779
CCDC3 OPTN	-0.2387	-0.08448	-0.15748	0.004256	-0.05808
CCDC32	0.496126	0.187814	0.07523	0.177589	0.219979
CCDC33	-0.19979	-0.16176	-0.01378	-0.2582	-0.50682
CCDC34	0.280421	0.482398	0.570763	0.286951	0.332075
CCDC37	0.304796	0.612388	0.760395	0.474391	0.580899
CCDC38 AMDHD1	-0.05356	-0.12715	-0.3109	-0.06495	-0.26546
CCDC4	0.106759	0.355674	0.419301	0.31464	0.47798
CCDC41	-0.46216	-0.23346	-0.43929	-0.33066	-0.39922
CCDC42	0.499102	0.538095	0.737457	0.373619	0.408543
CCDC43	-0.06457	-0.203	-0.09189	-0.11593	-0.33388
CCDC46	-0.3206	-0.49343	-0.22125	-0.47352	-0.36239
CCDC47 DDX42	-0.49416	-0.57947	-0.62631	-0.68027	-0.39573
CCDC48	0.321175	0.47681	0.177704	-0.2361	-0.09153
CCDC49	-0.02216	0.180817	0.017634	-0.06303	-0.01814
CCDC51	-0.07183	-0.05716	-0.17025	-0.18532	0.02843
CCDC52	-0.49155	-0.72894	-1.04401	-0.71188	-0.91665
CCDC53	0.330627	0.258092	0.159433	0.069065	-0.13461
CCDC54	-0.23581	-0.39439	-0.52505	-0.57085	-0.69326
CCDC55	-0.081	0.07254	-0.30324	0.09944	0.074254
CCDC56 CNTD1	0.527464	0.529441	0.619662	0.340857	0.570309
CCDC57	0.06239	-0.01439	0.035016	-0.03602	-0.01193
CCDC58 C3orf28	-0.83755	-0.62097	-0.68251	-0.66115	-0.79129
CCDC59 C12orf26	-0.60339	-0.77981	-0.69116	-0.58543	-0.8473
CCDC6	-0.14066	-0.00774	0.036389	-0.10433	-0.11699
CCDC60	0.139016	0.508367	0.68866	0.65734	0.388347
CCDC62	0.277365	0.617614	0.249773	0.240198	-0.01474
CCDC63	0.108516	0.582353	0.387706	0.285881	0.502443
CCDC64	-0.11865	0.196698	0.086735	0.054521	0.19325
CCDC66	-0.23847	-0.24398	-0.48187	-0.43276	-0.36877
CCDC68	0.430268	0.709756	0.828114	0.402702	0.528064
CCDC69	-0.02809	-0.04722	-0.04629	-0.20975	-0.02278
CCDC70	0.153835	0.06459	0.320317	-0.12699	-0.24727
CCDC74A	-0.06098	0.374738	0.236573	0.209399	0.337975
CCDC74B	0.079147	0.264351	0.172327	0.274002	0.335091
CCDC77	0.531677	0.657618	0.53446	0.240586	0.251929
CCDC78 HAGHL	0.132061	0.194534	0.246208	0.150024	0.258745
CCDC80	-0.39095	-0.23657	-0.12398	-0.20886	-0.18112
CCDC81	-0.04006	0.503267	0.672149	0.49339	0.105312
CCDC83	0.266399	0.411616	0.326124	0.298938	0.376719
CCDC84	-0.03444	0.089776	-0.23995	0.196512	0.044689
CCDC86	0.330334	0.213777	0.33938	0.117524	0.305118
CCDC87 CCS	0.348308	0.48198	0.466749	0.235826	0.447081
CCDC88A	-0.34072	-0.31446	-0.38568	-0.35638	-0.15136
CCDC88B	0.282399	0.212585	0.295226	0.17778	0.285939
CCDC89	0.169151	-0.19874	-0.18292	-0.3628	-0.98618
CCDC9	-0.28116	-0.41056	-0.47514	-0.25905	-0.3931
CCDC90A	0.273932	0.069704	0.043451	-0.05774	0.048747

CCDC90B	0.283038	0.445554	0.495516	0.142836	0.222953
CCDC91	-0.54439	-0.57142	-0.71443	-0.5369	-0.44278
CCDC92 ZNF664	0.035331	0.007172	0.081193	0.069004	0.113086
CCDC96 AC097382.3	-0.02924	0.104496	0.042828	0.065858	-0.05859
CCDC97	-0.23069	-0.29631	-0.91855	0.042006	-0.80345
CCDC98	0.023599	-0.02156	-0.02253	-0.09033	-0.24906
CCDC99	-0.20822	-0.96115	-1.15053	-0.88934	-0.54658
CCHCR1 TCF19	-1.15329	-0.85741	-1.5952	-0.66806	-1.03697
CCIN	0.16305	0.217365	0.083328	0.224848	0.352867
CCK	0.062836	0.177357	0.205917	0.204299	0.478566
CCKAR	0.492317	0.646248	0.393638	0.013649	0.263797
CCKBR	-0.43428	-0.62726	-0.47133	-0.10269	-0.35172
CCL1	-0.47871	-0.88051	-0.73798	-0.69084	-0.77501
CCL11	-0.17928	-0.57808	-0.99487	-0.80863	-1.02428
CCL13	0.042456	-0.24809	-0.3459	-0.59957	-0.4027
CCL16	0.298985	-0.09606	-0.55607	-0.32384	-0.28183
CCL17	0.321001	0.2443	0.268598	0.204308	0.231033
CCL18	-0.55531	-0.31514	-0.43992	-0.39006	-0.47445
CCL19	0.102913	0.197149	-0.02812	-0.16361	-0.00819
CCL2	-0.51363	-0.49182	0.253852	-0.53734	-0.87796
CCL20	0.580707	0.533011	0.034648	0.29824	-0.07881
CCL21	0.139471	-0.08472	0.147652	0.045259	-0.01862
CCL22	0.290931	0.527256	0.590796	0.185406	0.569372
CCL23	0.229984	-0.44332	-0.72089	-0.46031	-0.29162
CCL24	0.345372	-0.27061	0.352262	-0.08802	0.228181
CCL26	0.386631	0.56553	0.160375	0.011312	0.144888
CCL27	-0.27476	-0.05015	-0.57979	-0.5078	-0.37426
CCL28	-0.25298	-0.16299	-0.47191	-0.49328	-0.56163
CCL3	0.237033	-1.14278	-1.7702	-1.18415	-1.00947
CCL3L1	0.153045	-0.78168	-1.74875	-0.69554	-1.11855
CCL3L3	0.037178	-0.89845	-1.45924	-0.60022	-0.92682
CCL4	-0.2367	-1.41494	-1.74632	-1.01337	-1.41052
CCL4L1	-0.31044	-1.57719	-1.94381	-1.08682	-1.62193
CCL4L2	-0.24721	-1.56432	-1.912	-1.2718	-1.44025
CCL5	0.355448	0.30498	0.537099	0.221042	0.338598
CCL7	-0.6461	-0.50563	-0.62184	-0.72023	-0.65127
CCL8	-0.01637	-0.35837	-0.76609	-0.88337	-1.10961
CCNA1	-0.01777	0.15689	-0.16607	-0.06532	0.131086
CCNA2	-0.69267	-0.91306	-0.68813	-0.45178	-0.54139
CCNB1	0.157188	0.065036	0.074448	-0.16328	-0.2088
CCNB2	-0.12955	-0.09374	-0.27319	-0.32619	-0.52323
CCNB3	-0.29253	0.048002	-0.29834	-0.5991	-0.10959
CCNC	-0.21144	-0.14972	-0.3653	-0.2498	-0.26955
CCND1	-0.76558	-0.70435	-0.78583	-0.65184	-0.66142
CCND2	-0.09886	-0.0606	0.029046	0.255031	0.120864
CCND3	0.01448	0.055345	0.05032	0.063521	0.059378
CCNE1	0.023286	0.01455	-0.02442	0.051852	0.084445
CCNE2 C8orf38	-0.06749	-0.03746	-0.03287	-0.10288	-0.11984
CCNF	0.03066	-0.1005	-0.1034	0.033787	-0.02923
CCNG1	-0.4705	-0.25611	-0.43614	-0.41713	-0.50378

CCNG2	-0.28901	-0.30351	-0.37731	-0.15386	-0.27244
CCNH	-0.6915	-0.57229	-0.9586	-0.76073	-0.81343
CCNI	-0.37571	-0.39173	-0.5944	-0.36154	-0.39579
CCNI2	0.117752	0.130083	0.457936	0.264101	0.169264
CCNJ	0.256754	0.446096	0.289576	-0.04809	0.397081
CCNJL	-0.04985	-0.12125	-0.24322	-0.13257	-0.16638
CCNL1	0.027784	0.05661	-0.05763	-0.06155	-0.04928
CCNO	0.013593	0.078488	0.146966	-0.00173	0.09377
CCNT1	-0.32104	0.109942	-0.71501	-0.09259	-0.42588
CCNT2	-0.68945	-0.73443	-1.07741	-0.79518	-0.4254
CCNYL1	-0.07348	-0.00987	-0.13183	-0.14227	-0.12566
CCPG1	0.163375	0.178411	0.071431	-0.02606	0.046457
CCR1	-0.1624	-0.04827	-0.9053	-0.37908	-0.30993
CCR10 CNTNAP1	-0.30485	0.201236	-0.0018	-0.15007	0.070311
CCR2.	-0.1758	-0.71043	-1.21071	-0.4214	-1.3543
CCR3	0.381415	0.078073	-1.04367	0.004012	-0.5148
CCR4	0.356004	0.489223	0.690712	0.332447	0.593415
CCR5	0.3967	0.172092	-0.66515	0.226425	-0.35296
CCR6	0.437085	0.712823	0.931033	0.574884	0.737134
CCR7	0.187671	0.307134	0.454608	0.096881	0.241289
CCR8	-0.389	-0.23145	-0.36836	-0.20671	-0.20511
CCR9	0.343465	0.156357	0.306903	0.161402	0.260896
CCRK	0.173061	0.143766	-0.13971	0.07646	0.028815
CCRL2	0.365914	0.652674	0.597585	0.51996	0.395543
CCRN4L	0.011097	-0.21042	0.022619	-0.06744	-0.22866
CCT2	0.009144	0.000907	-0.03451	0.063792	-0.08705
CCT3	0.313766	0.356192	0.351216	-0.05257	-0.04057
CCT4	-0.30935	-0.36192	-0.30596	-0.05176	-0.124
CCT6A SUMF2	0.309367	0.442333	0.742776	0.16091	0.172892
CCT6B ZNF830	-0.7246	-0.5969	-0.80228	-0.17019	-0.8808
CCT8	-0.2238	-0.11529	-0.04026	-0.15278	-0.17599
CD109	0.196841	0.047095	0.231833	0.218953	0.216263
CD14 AC116353.2	0.184142	0.553024	0.713592	0.366848	0.244996
CD15 TSPAN4	-0.15529	-0.19426	-0.19272	0.023593	0.079033
CD151	-0.04407	0.152934	0.105418	-0.00786	0.14098
CD160	0.517714	0.113518	0.621414	-0.02721	0.466178
CD163	-0.34096	-0.4297	-0.60995	-0.64701	-0.88064
CD163L1	-0.09735	-0.16905	-0.44098	-0.53024	-0.50951
CD164	-0.50553	-0.1305	-0.31096	-0.41899	-0.40181
CD164L2	0.250222	0.361128	0.44737	0.325691	0.34525
CD180	-0.17107	0.100556	-0.32132	-0.24504	-0.02709
CD19	0.594622	0.679646	0.8395	0.510305	0.726081
CD1A	-0.31891	-0.80539	-0.86793	-0.80936	-0.53283
CD1B	-0.84539	-1.64137	-1.40636	-1.49428	-1.2952
CD1C	-0.66595	-2.47265	-2.283	-2.07777	-2.10173
CD1D	0.20694	-1.54012	-1.54416	-0.24871	-0.98624
CD1E	-1.04404	-1.67928	-1.43131	-1.28305	-1.28288
CD2	0.148248	0.098263	0.195454	-0.26395	-0.01128
CD200	0.041997	0.314671	0.110666	0.010348	-0.01721
CD200R1	0.431884	0.683843	0.408443	0.047488	-0.04688

CD207	0.330749	-0.39397	0.240887	-0.08784	0.13245
CD209	-0.47659	-1.21629	-0.97009	-0.99982	-0.98032
CD22	0.601848	-0.20238	0.604166	0.094653	0.396965
CD226	0.671381	0.939381	1.150286	0.697484	0.838073
CD244	0.114439	-0.88324	-1.10161	-0.85094	-0.36231
CD247	0.297997	0.536484	0.579757	0.326393	0.255901
CD248	0.258361	-0.2946	0.086815	-0.16448	-0.1564
CD27	0.423107	0.727686	0.402948	0.345084	0.585869
CD27 TAPBPL	0.138852	-0.46565	0.412022	-0.49162	-0.63023
CD274	0.178444	-0.01549	0.26491	0.2435	0.399077
CD276	-0.23682	-0.39162	-0.36043	-0.31268	-0.2204
CD28	0.486757	0.852056	0.872419	0.431803	0.459003
CD2AP	0.012072	-0.07544	0.141742	0.043721	0.113154
CD300A	0.302461	0.56731	0.545812	0.284567	0.484347
CD300C	0.058428	0.120498	0.301084	0.128437	0.362473
CD300E	-0.06345	-0.67923	0.428386	0.135162	-0.02083
CD300LB	0.209537	0.284592	0.556126	0.311369	0.532413
CD300LG	0.333276	0.382985	0.430859	0.343134	0.428165
CD302	0.545973	0.352466	0.073902	0.393745	0.235408
CD33	0.146991	-0.66939	-0.54426	-0.49772	-0.40791
CD34	0.353901	0.482386	0.617362	0.33604	0.288847
CD38	0.212136	0.397028	0.392845	0.272832	0.184459
CD3D CD3G	0.383638	0.286233	0.367408	0.134507	0.181092
CD3E	0.179691	0.100528	-0.11964	-0.26193	-0.20325
CD4	0.458958	0.653198	0.689031	0.232249	0.651619
CD40	0.279001	0.336913	0.599946	0.303539	0.551907
CD40LG	0.357604	0.412879	0.316272	0.187387	0.465869
CD44	-0.24854	0.069588	-0.01387	0.105683	0.000534
CD46	-0.08015	0.046087	-0.20645	0.013794	0.085192
CD47	0.254195	0.34744	0.431819	0.177859	0.316767
CD48	-0.04482	-1.47805	-1.47703	-1.29323	-0.96022
CD5	0.201779	0.08138	0.321506	0.151816	0.15215
CD53	-0.44513	-1.70078	-0.8841	-1.21506	-1.494
CD55	-0.22309	-0.46832	0.076606	0.246728	-0.26665
CD58	0.01401	0.017449	-0.01756	0.108522	0.204754
CD59	-0.14499	-0.0787	-0.33743	0.111168	0.114895
CD5L	-0.48893	-1.60908	-1.39328	-1.2761	-1.1634
CD6	0.44025	0.593603	0.683931	0.444696	0.329822
CD69	0.349269	-1.25892	-0.93068	-0.77017	-0.82483
CD7	0.180628	0.243921	0.426443	0.271765	0.385303
CD70	-0.20766	-0.26715	0.223642	0.346971	0.428535
CD72	0.33659	0.41047	0.427966	0.313227	0.519499
CD74	0.409351	0.712577	0.593917	0.514663	0.567214
CD79A.	0.182983	-0.11599	-0.04438	-0.2976	-0.18547
CD79B	0.356522	0.242891	0.458104	0.322691	0.348655
CD80	-0.00439	0.127468	0.433675	0.222014	0.33238
CD81	0.044934	0.054266	0.09971	-0.02746	0.177051
CD82	-0.20113	0.02605	-0.01897	0.148479	0.127425
CD83	0.049642	0.039009	-0.0568	-0.07117	-0.13281
CD84	-0.44826	-0.952	-0.98999	-0.66041	-0.63717

CD8A	-0.16005	0.066763	0.506883	0.453743	0.065709
CD8B	0.288562	0.246408	0.630155	0.623291	0.476377
CD9	0.138862	0.310623	0.138525	-0.34356	0.175007
CD97	0.355729	0.213157	0.595856	0.268353	0.093301
CD99	0.179297	0.320588	0.39341	0.269855	0.318984
CD99L2	0.035008	0.059132	0.227784	0.058675	0.043212
CDADC1	-1.05497	-0.93047	-1.32008	-0.89752	-1.18398
CDAN1	0.159752	0.275193	0.19911	0.124279	0.156992
CDC14A	-0.80307	-0.67085	-0.76709	-0.8065	-0.58577
CDC14B	0.108212	0.250524	0.576672	0.342014	0.38091
CDC16	0.109647	0.112167	0.136957	-0.02634	0.057084
CDC2	-0.23024	-0.41571	-0.25414	-0.31537	-0.72829
CDC20	-0.29329	-0.18354	-0.43585	-0.24869	-0.08182
CDC23	-0.41285	-0.14374	-0.46635	-0.30337	-0.41789
CDC25A	-0.17115	0.028562	-0.05724	-0.02186	-0.01742
CDC25B	0.265017	0.200451	-0.08491	0.097204	0.133424
CDC25C	-0.08444	-0.55256	-0.87479	-0.35483	-0.68871
CDC26 PRPF4	-0.29895	-0.43577	-0.47642	-0.33997	-0.53449
CDC27	-0.38747	-0.49101	-0.26537	-0.49954	-0.34406
CDC2L5	-0.10529	-0.39965	-0.27854	-0.12015	-0.12122
CDC2L6	-0.46246	-0.1482	-0.42817	-0.23698	-0.44079
CDC34	0.079606	0.037391	0.212866	0.04351	0.176681
CDC37	-0.48052	-0.41085	-0.48984	-0.31385	-0.35887
CDC42	0.098432	0.059727	0.261571	0.13275	0.173016
CDC42BPA	-0.16891	-0.19051	-0.09318	-0.20585	-0.15887
CDC42BPB	0.007298	0.076189	0.065357	0.0067	0.085356
CDC42BPG	0.182372	0.46851	0.463633	0.303131	0.449186
CDC42EP1	-0.05913	0.000606	0.15269	0.043011	0.028126
CDC42EP2	-0.06416	-0.05755	0.039454	-0.02806	-0.01018
CDC42EP3	-0.04797	0.019471	0.081487	-0.07461	-0.06106
CDC42EP4	-0.00732	-0.14608	-0.03642	-0.16961	0.01402
CDC42EP5	-0.06384	-0.93253	-0.57506	-0.56075	-0.92759
CDC42SE2	0.099806	-0.15919	-0.08382	0.045103	0.04535
CDC5L	-0.29154	-0.63863	-1.01656	-0.62718	-0.67829
CDC6	-0.62882	-0.48931	-0.77281	-0.59321	-0.56011
CDC7	-0.741	-0.29569	-0.59135	-0.70761	-0.22064
CDC91L1	-0.06491	0.078883	-0.01582	-0.07499	0.050965
CDCA3 USP5	-0.66467	-0.69582	-0.7494	-0.25072	-0.696
CDCA4	-0.05306	0.09437	0.03553	0.028198	0.060055
CDCA5 ZFPL1	-0.28205	-0.12144	-0.38587	-0.18223	-0.11867
CDCA7	-0.34684	0.101584	-0.15031	-0.05418	0.073674
CDCA7L	-0.24032	-0.2451	-0.40604	-0.09827	-0.14016
CDCP1	0.35523	0.445287	0.794002	0.415448	0.489592
CDCP2	0.307583	0.594189	0.777888	0.544789	0.48647
CDH1	0.211425	0.464051	0.572171	0.46679	0.496399
CDH10	-0.30958	0.073076	-0.84633	-0.74599	-0.74263
CDH11	-0.37577	-0.34459	-0.3032	-0.19364	-0.23192
CDH12	-0.01399	-0.44961	-0.52973	-0.4122	-0.3947
CDH15	0.131519	-0.07748	0.194068	0.266525	-0.07108
CDH16	0.076592	0.101644	0.023505	-0.05568	-0.01445

CDH17	0.112518	0.497381	0.194611	0.109322	0.259131
CDH18	-0.2365	0.061942	-0.4796	-0.28592	-0.43685
CDH19	0.40414	0.662459	0.358602	0.220377	0.307618
CDH2	-0.22561	-0.18118	-0.159	-0.14827	-0.20796
CDH20	0.418054	0.617905	0.534994	0.249925	0.125665
CDH22	0.257582	0.214643	0.409378	0.224943	0.368754
CDH24	0.030202	-0.08455	-0.22899	-0.18924	-0.21082
CDH3	0.082358	0.174311	0.597874	0.354696	0.25541
CDH4	0.035552	-0.09238	0.03776	-0.00364	-0.0056
CDH5	0.001811	0.074913	0.075693	0.032583	0.151287
CDH6	0.018675	0.290215	-0.0564	0.2619	0.244914
CDH7	-0.15783	0.393427	0.469258	-0.07769	0.180457
CDH8	-0.56858	-0.2973	-0.33076	0.037256	0.329969
CDH9	0.143381	0.869327	-0.64391	0.466204	0.44413
CDIPT AC120114.2	0.104956	0.129465	0.014575	0.036886	-0.04131
CDK10	0.123321	0.051456	0.003228	0.075645	0.099532
CDK2AP1	0.139237	0.344011	0.506194	-0.05867	0.431473
CDK2AP2	-0.02192	0.087073	0.045091	0.039788	0.001587
CDK3	0.012612	0.138293	0.156612	0.134369	0.250407
CDK4	-0.43226	-0.47202	-0.68403	-0.49592	-0.60891
CDK5	-0.30833	-0.48897	-0.86748	-0.35732	-0.33313
CDK5R1	0.033662	0.007308	0.060509	0.085953	0.202765
CDK5R2	-0.0986	-0.06257	-0.30585	-0.1927	-0.2339
CDK5RAP1	-0.1954	-0.09965	-0.3687	-0.418	-0.46178
CDK5RAP2	0.090102	0.156039	-0.02923	-0.01203	0.023187
CDK5RAP3	-0.06304	-0.06421	-0.17681	0.052532	-0.11813
CDK6	-0.34879	-0.48024	-0.60603	-0.26531	-0.35254
CDK8	-0.1546	-0.22412	-0.19132	-0.16802	-0.01237
CDK9	-0.33734	-0.1508	-0.28283	-0.30455	-0.28084
CDKAL1	-0.10165	0.113984	-0.51475	-0.52867	-0.38511
CDKL1	0.521904	0.659215	0.686375	0.21523	0.294884
CDKL2	0.115577	0.225239	-0.04362	0.144203	0.239186
CDKL3	-0.11758	-0.29074	-0.33087	-0.28102	-0.31008
CDKL4	-0.16618	0.015613	-0.20434	-0.17123	-0.26489
CDKN1A	-0.42768	-0.49706	-0.61771	-0.39992	-0.40893
CDKN1B	-0.30913	0.566827	-0.2644	-0.31304	-0.35053
CDKN1C	-0.12808	-0.02546	-0.00119	-0.00927	0.127459
CDKN2AIP	-0.06505	-0.09079	-0.07574	-0.01755	0.098568
CDKN2AIPNL	0.108192	0.181862	0.068221	0.234978	0.053615
CDKN2C	-0.48258	-0.59159	-0.52847	-0.58372	-0.11429
CDKN2D	0.001898	0.04133	0.118742	0.104641	0.112573
CDKN3	0.239464	0.174675	0.110798	0.088295	0.073169
CDO1	-0.08564	0.374507	-0.08519	-0.16933	0.328189
CDON	0.18904	0.168916	0.016235	0.120873	-0.02209
CDR1	0.438932	-0.53601	0.081187	0.36921	0.519109
CDR2	-0.22356	0.024166	-0.18316	-0.03349	-0.20742
CDR2L	0.000893	-0.0012	0.193683	-0.02791	0.1538
CDRT15	0.31515	0.419877	0.627092	0.325426	0.231071
CDS1	0.041577	0.325687	0.175203	0.220781	0.381594
CDT1	-0.01584	0.069696	-0.18775	0.055029	-0.07903

CDV3	-0.43614	-0.30087	-0.55983	-0.52715	-0.44386
CDX1	0.141902	0.542026	0.485562	0.278373	0.293404
CDX2	-0.30504	-0.29826	0.306431	0.281402	0.330775
CDX4	0.286512	0.315153	0.378319	0.410253	0.541277
CDY1	0.223685	0.242682	0.367669	-0.05727	0.26665
CDY2A	0.240096	0.18816	0.165888	-0.11035	0.128128
CDYL2	-0.10822	-0.00306	-0.0359	-0.01146	0.025344
CEACAM1	0.030583	-0.17768	0.038637	-0.10802	-0.10973
CEACAM19	0.194361	0.063167	-0.10972	0.012782	0.078342
CEACAM21	-0.11325	-0.79189	-0.46428	-0.28697	-0.22328
CEACAM3	-0.2455	-0.74955	-0.54893	-0.37107	-0.34377
CEACAM4	-0.29889	-0.31477	-0.55682	-0.36817	-0.54661
CEACAM5	-0.3365	-0.58104	-0.5364	-0.46607	-0.55162
CEACAM6	-0.30616	-0.87721	-0.70476	-0.68232	-0.75569
CEACAM7	-0.46396	-0.97251	-0.60444	-0.6739	-0.50202
CEACAM8	0.02177	-0.34409	-0.35122	-0.22755	-0.17989
CEBPB	0.070065	0.008178	0.1769	0.015621	0.07929
CEBPE	0.474121	0.379343	0.42575	-0.00242	0.157489
CEBPG	0.086397	0.13431	0.197189	0.177391	0.199337
CECR6	-0.20863	-0.38933	-0.36246	-0.12431	-0.26828
CEL	0.291892	0.551593	0.528269	0.243957	0.46133
CELSR1	0.029178	0.295569	0.106102	0.186755	0.150127
CELSR2	-0.23708	-0.31651	-0.28472	-0.18332	-0.15779
CEND1 SLC25A22	0.214928	0.025653	-0.00429	0.266566	0.241743
CENPA	-0.30902	0.319512	0.041804	0.169174	-0.17792
CENPB	0.061125	-0.01835	-0.13558	-0.00117	0.080254
CENPC1	-0.27111	-0.19831	-0.40544	-0.40007	-0.27747
CENPE	0.179598	-0.10826	-0.15964	0.068327	-0.22067
CENPF	0.38311	0.315544	0.31508	-0.28751	0.227044
CENPH	-0.36706	-0.28835	-0.45707	-0.31603	-0.32659
CENPI	0.112386	-0.04346	0.055962	-0.34382	-0.34966
CENPJ	0.046438	-0.20669	-0.21878	-0.06648	0.022497
CENPK PPWD1	-0.63618	-1.33616	-1.42799	-0.80842	-1.21585
CENPL DARS2	-0.61578	-0.99361	-1.16856	-0.77427	-0.71814
CENPM	-0.01547	0.167888	0.099642	-0.10072	-0.0827
CENPT	0.222651	0.274509	0.178857	0.057846	0.110559
CENTA1	0.023087	0.098269	0.271164	0.017423	0.128109
CENTA2	0.196377	0.420079	0.324691	0.316598	0.38697
CENTB1	0.258408	0.022254	0.340403	0.133039	0.196895
CENTB2	0.01891	0.089451	-0.05872	0.008854	-0.01257
CENTB5	0.29111	0.362913	0.260866	0.229977	0.282954
CENTD3	0.059678	0.178521	-0.03938	-0.01363	0.130872
CENTG1	0.557356	0.718719	0.509707	-0.02682	0.018906
CENTG2	0.031934	0.092477	0.073822	0.086936	0.0899
CEP110	0.071747	0.175753	0.13934	-0.03585	-0.1267
CEP135	-0.23275	-0.08282	-0.20554	0.002607	0.033878
CEP152	-0.16353	-0.14689	-0.48125	-0.2175	-0.54838
CEP164	0.271589	0.395716	0.400977	0.122648	0.056886
CEP192	0.114547	0.098754	0.000328	-0.19159	0.088057
CEP290 TMTC3	-0.67565	-0.89609	-0.86622	-0.8646	-0.92487

CEP350	-0.196	-0.52668	-0.37535	-0.4267	-0.14067
CEP55	-0.12891	-0.09451	-0.08537	-0.1516	-0.0434
CEP68	-0.10897	-0.21356	-0.13448	-0.08322	0.020229
CEP70	-0.52743	-0.28492	-0.56374	-0.33093	-0.23896
CEP72	-0.25659	-0.40834	-0.33183	-0.18826	-0.19381
CEP76 TNFSF5IP1	0.011396	-0.12757	0.02616	-0.01196	0.064611
CEP97	0.130455	-0.62335	0.020438	-0.1117	-0.37257
CER1	-0.34216	0.335385	0.518304	0.084545	0.469053
CERCAM	0.13433	0.134801	0.182935	0.170749	0.18942
CERKL	0.251205	0.471892	0.362812	0.447454	0.223962
CES1	0.369272	0.741213	0.558237	0.419687	0.547341
CES3	0.107023	0.339973	0.199496	0.06428	0.210509
CES7	-0.28598	-0.62672	-0.50351	-0.52005	-0.55021
CESK1	0.284336	-0.18138	-0.11914	-0.22622	-0.20958
CETN1	0.456034	0.669369	0.701404	0.474905	0.678697
CETN2 NSDHL	-0.0052	-0.28066	-0.67233	-0.22567	-0.26628
CETN3 AC093510.2	-1.03815	-0.95407	-1.39395	-0.99879	-1.07103
CETP	0.515711	0.700052	0.709398	0.432406	0.625377
CFD	0.087474	-0.13558	0.000697	-0.14435	0.015988
CFDP1	0.044473	-0.15019	0.463451	0.268068	-0.39348
CFH	-0.58995	-0.26233	0.071169	-0.35257	-0.08277
CFHR1	0.321976	-0.39392	0.285794	0.092708	0.161291
CFHR2	0.29832	-0.42574	0.276757	0.069234	0.102467
CFHR3	0.1867	0.021637	-0.28522	-0.25865	-0.16292
CFHR5	0.359357	-0.49532	0.300591	0.147723	0.162662
CFI	-0.46812	-0.5672	-0.312	-0.34779	-0.5036
CFL1 MUS81	-0.46261	-0.43854	-0.40541	-0.51352	-0.39307
CFL2	0.017353	-0.05452	-0.04769	-0.21123	-0.28188
CFLAR	0.265379	0.147956	0.346965	0.335266	0.046581
CFP	0.180703	0.285169	0.232393	0.275212	0.144734
CFTR	0.327465	0.531146	0.081196	0.506261	0.486985
CGA	0.7485	0.664211	0.704522	-0.05108	0.129872
CGB	0.584016	-0.43149	0.806246	0.365283	0.628524
CGB1	0.595983	0.178033	0.962573	0.71867	0.410729
CGB2	0.354351	-0.26752	0.557756	0.445769	0.424684
CGB5	0.660119	-0.58186	0.809998	0.474231	0.588617
CGB7	0.337569	-0.19479	0.601098	0.388067	0.420528
CGB8	0.69301	-0.51645	0.736407	0.454969	0.639902
CGGBP1	-0.09394	-0.0925	-0.0504	-0.15016	-0.04709
CGN	0.052326	0.156646	-0.01183	0.070308	0.076456
CGNL1	0.377338	0.415055	0.632666	0.378499	0.347951
CGREF1	0.251762	0.368458	0.143064	0.210072	0.340589
CGRRF1	-0.31739	-0.4656	-0.60688	-0.44958	-0.63813
CH25H	-0.33444	0.393703	-0.25068	-0.13374	-0.11216
CHAC1	0.134118	0.137533	0.127573	0.19519	0.293771
CHAF1B	0.230591	0.425465	0.273535	0.275108	0.410197
CHCHD1	-0.28507	-0.65473	-0.88761	-0.40248	-0.63108
CHCHD2	0.334249	0.296535	0.354814	0.063093	0.132332
CHCHD3	0.049199	0.081568	-0.2252	-0.09497	-0.10227
CHCHD4 TMEM43	-0.05848	-0.07475	-0.17442	-0.081	0.082693

CHCHD5	-0.15955	-0.65235	-0.87924	-0.49607	-0.30348
CHCHD6	0.049273	0.130555	-0.14268	-0.15715	0.082974
CHCHD8 PAAF1	-0.46751	-0.2035	-0.79146	-0.55991	-0.72613
CHD1	0.199229	-0.25443	0.08091	-0.14781	-0.28596
CHD1L	0.227034	-0.06411	-0.21978	-0.43318	-0.39086
CHD2	-0.23828	-0.20058	-0.49878	-0.44491	-0.29525
CHD4	-0.64758	-0.61786	-0.84963	-0.68759	-0.74295
CHD5	0.0109	0.104839	0.111399	0.013299	0.044958
CHD6	-0.1446	-0.01252	-0.12696	-0.06934	-0.09843
CHD7	0.084255	0.175902	0.156606	-0.01611	0.163379
CHDH IL17RB	0.240967	0.41941	0.314046	0.219923	0.441868
CHEK1	-1.12407	-0.9712	-1.72222	-0.94359	-1.21993
CHEK2 HSCB	-0.36087	-0.29829	-0.61804	-0.29581	-0.29002
CHERP	-0.31743	-0.28448	-0.36566	-0.191	-0.32358
CHFR	0.13519	0.119879	0.09528	0.385838	0.18395
CHGA	0.272732	-0.0378	-0.03419	-0.07543	-0.20449
CHGB	-0.14374	-0.50754	-0.35566	-0.32596	-0.4427
CHI3L1	0.54709	0.640045	0.722235	0.473409	0.555743
CHI3L2	0.215378	-0.14724	0.062453	-0.26778	-0.25122
CHIA	0.423156	-0.0553	0.199511	-0.13685	-0.32108
CHIC1	0.085024	-0.42022	-0.39077	-0.41727	-0.407
CHIC2	-0.13386	0.029977	-0.27088	-0.33449	-0.18153
CHID1	0.049158	0.077525	-0.0779	0.057909	0.106256
CHIT1	0.13378	0.019023	0.086739	0.024342	0.087757
CHKA	0.149744	0.150331	0.155944	0.115846	0.148333
CHL1	-0.23535	0.155931	0.116239	0.017453	-0.04687
CHM	0.513643	0.447673	0.458984	0.458677	-0.00878
CHMP1A C16orf55	0.02178	-0.15957	-0.25957	0.020686	0.023974
CHMP2A UBE2M	0.061973	-0.05476	-0.04308	0.11238	-0.04913
CHMP2B	-0.51602	-0.38997	-0.43465	-0.46089	-0.51261
CHMP4A	0.097162	0.246348	0.060324	-0.05668	0.060676
CHMP4B	-0.50513	-0.6072	-0.7654	-0.44395	-0.62367
CHMP4C	0.350784	0.571746	0.838944	0.416581	0.506499
CHMP6	0.263698	0.263251	0.318704	0.140441	0.307376
CHMP7	0.274623	0.79995	0.658479	0.14689	0.025975
CHN1	0.076315	0.03202	-0.11144	-0.05106	0.055678
CHODL	0.000133	0.381345	0.308919	0.034717	0.126865
CHORDC1	-0.24383	-0.12241	-0.3665	-0.23226	-0.44587
CHPF TMEM198	-0.09589	-0.18881	-0.03468	-0.19656	-0.05325
CHPT1	0.213299	0.333103	0.162356	-0.02811	0.220927
CHRAC1	-0.08762	-0.15236	-0.25022	-0.16106	-0.02694
CHRDL1	0.078288	0.174473	0.160196	-0.04974	0.130174
CHRDL2	-0.14538	-0.20677	0.201631	-0.18626	0.104335
CHRFAM7A	0.050881	-0.29665	-0.42474	-0.03808	-0.13966
CHRM1	0.390785	-0.21221	0.236788	-0.34374	-0.11611
CHRM2	-0.5314	-0.73432	-0.52163	-0.71956	-0.61891
CHRNA1	0.238343	0.596392	0.480431	-0.0961	0.300311
CHRNA10	0.15616	0.410979	-0.01091	-0.07443	0.112397
CHRNA2	-0.04462	0.045031	0.228148	0.048019	0.071544
CHRNA3	-0.0395	-0.23963	-0.34402	-0.24659	-0.30216

CHRNA4 AL121827.33	-0.12542	-0.02359	0.312907	0.136115	0.190894
CHRNA5	-0.00406	-0.09023	0.120287	0.011159	0.07602
CHRNA6	-0.27361	-0.15261	-0.3666	-0.36069	-0.70899
CHRNA7	-0.06993	-0.04354	0.030521	0.068573	0.030203
CHRNA9	0.131097	0.321149	0.091668	-0.09116	0.331246
CHRNB2	-0.21356	-0.28884	-0.38375	-0.35435	-0.2382
CHRNB3	0.512107	0.607023	0.910613	0.424331	0.642404
CHRNB4	0.013253	-0.22019	-0.10902	-0.03469	-0.04717
CHRND	0.137192	0.237224	0.02103	-0.02397	0.073336
CHRNE	0.212029	0.2592	0.157975	0.223438	0.015332
CHRNG	0.164665	-0.00169	0.184569	0.08403	0.059935
CHST1	0.13103	0.043675	0.157094	0.028607	0.105813
CHST10	0.157088	0.120311	0.220822	0.130416	0.394973
CHST13	0.153509	0.228776	0.268556	0.132523	0.184468
CHST2	-0.16406	0.007334	0.122962	0.051351	0.077281
CHST3	-0.02399	0.16902	0.120903	0.049209	0.191592
CHST4	0.109524	-1.37022	0.436697	-0.30751	-0.86623
CHST5	0.298509	0.658859	0.701715	0.27529	0.544813
CHST6	0.20592	0.330493	0.456892	0.219179	0.353388
CHST7	0.023716	0.031622	0.019252	0.010645	-0.07471
CHST8	0.205801	0.482779	0.564271	0.063119	0.51976
CHST9	0.262685	0.428841	0.314723	0.320934	0.044716
CHSY1	0.052732	0.064963	0.0055	0.053083	0.150584
CHSY3	0.042536	-0.02594	0.139281	0.121123	0.091442
CHUK	0.176107	0.287651	0.005768	0.009409	-0.03409
CHURC1	0.146458	-0.02463	-0.3065	-0.28748	-0.35934
CIAPIN1 COQ9	-0.05464	-0.07927	-0.21984	-0.20146	-0.20351
CIB1	0.272878	0.031121	-0.06114	0.118973	-0.05285
CIB2	0.219913	0.37161	0.251008	0.168573	0.318148
CIB3	0.073325	-0.89383	-0.56897	-0.64368	-0.78966
CIB4	0.093132	0.229301	0.019927	-0.21393	0.200563
CIC	-0.13755	-0.26964	-0.28524	-0.31046	-0.20688
CIDEA	0.128757	0.259653	0.298977	0.138865	0.282127
CIDEB LTB4R2 LTB4R	0.056814	0.293399	0.30719	0.035794	0.247372
CIDEC	0.18159	0.339704	0.59122	0.297885	0.510282
CIITA	0.132953	0.063289	-0.30737	0.208031	-0.54593
CILP	-0.08981	-0.17314	-0.30224	-0.36511	-0.18432
CILP2	0.442029	0.439549	0.341418	0.431679	0.324396
CIRBP	0.108611	0.185478	0.063888	0.119151	0.160799
CISH	0.004886	-0.14971	-0.39303	-0.17862	-0.04044
CIT	0.098458	0.166137	0.038807	-0.02043	-0.12361
CITED1	0.088895	-0.00747	0.154763	0.100104	0.133965
CITED2	-0.62252	-0.33383	-0.26745	-0.42464	-0.45278
CITED4	0.209107	-0.02302	0.2173	0.047208	0.118124
CIZ1 DNM1	-0.11816	-0.1178	-0.18206	-0.06366	-0.1751
CKAP2	-0.25828	-0.15989	-0.27152	0.057193	-0.02049
CKAP2L	-0.05311	0.110318	-0.16256	-0.04392	0.084582
CKAP4	-0.114	0.067736	0.000227	0.003156	0.094889
CKAP5	-0.14186	-0.37198	-0.41581	-0.29811	-0.213
CKB	-0.02437	0.118706	0.221103	0.179765	0.168163

CKM	0.117165	-0.17527	0.284782	-0.06351	0.292685
CKMT1A	-1.03781	0.201531	0.039781	-0.18251	-0.05312
CKMT2	0.421914	0.571742	0.723477	0.351436	0.587733
CKS2	-0.50608	-0.27697	-0.49373	-0.3316	-0.42289
CLASP1	-0.61332	-0.53431	-0.47043	-0.43939	-0.3279
CLASP2	-0.10723	-0.01161	-0.28973	-0.30491	0.006802
CLC	-0.51798	-0.84864	-1.13709	-0.88204	-0.96209
CLCA1	-0.08965	0.1222	-0.21919	-0.37092	-0.30889
CLCA2	-0.38168	-0.34146	-0.51764	-0.43994	-0.5579
CLCA3	-0.00239	0.166474	-0.60585	-0.17549	-0.63794
CLCA4	-0.17445	0.101865	-0.69004	-0.49082	-0.45508
CLCC1	0.028696	0.035575	0.251722	-0.07519	-0.05571
CLCF1	-0.31072	-0.19278	-0.17258	-0.00877	-0.211
CLCN1	0.285389	0.307965	0.354569	0.24489	0.3579
CLCN2 POLR2H	-0.199	-0.34113	-0.53846	-0.26006	-0.26675
CLCN3	-0.28774	-0.37927	-0.46561	-0.39613	-0.49865
CLCN4	-0.13131	-0.2032	-0.46655	-0.28403	-0.01561
CLCN5	-0.62629	-0.11804	-0.86887	-0.45293	-0.396
CLCN7	0.192631	0.256525	0.279678	0.146655	0.164143
CLCNKA	-0.01349	0.216733	-0.51692	-0.25466	0.023493
CLCNKB	0.265405	0.362355	0.353848	0.17246	0.135804
CLDN1	0.141105	-0.12247	-0.21783	0.224607	0.042336
CLDN11	0.407176	0.520904	0.619768	0.441123	0.467805
CLDN12	-0.66531	-0.47746	-0.75999	-0.45381	-0.68051
CLDN15	0.27823	0.486611	0.595785	0.379958	0.24488
CLDN16	0.571471	-1.13923	-0.97524	0.322581	-0.32252
CLDN17	-0.00989	-0.73771	-0.59041	-0.37385	-0.40043
CLDN19	0.14438	0.413781	0.41349	0.226446	0.367832
CLDN2	-0.0142	0.32453	-0.10967	-0.03643	-0.10048
CLDN3	0.176713	0.259096	0.337165	0.174628	0.126152
CLDN4	0.355359	0.545651	0.567198	0.391675	0.546146
CLDN5	0.143268	0.224807	0.160388	0.221739	0.312481
CLDN6 TNFRSF12A	0.152076	0.14822	0.238341	0.154114	0.264547
CLDN7	-0.10704	-0.29036	0.607792	0.385277	0.359847
CLDN8	0.54087	-1.38649	-1.21038	0.056956	0.021502
CLDND1	-0.48236	-0.42035	-0.32833	-0.2418	-0.44563
CLDND2	0.212032	0.345505	0.250863	0.259601	0.390088
CLEC10A	0.428701	-0.43198	-0.31806	-0.36593	0.416961
CLEC11A	0.062909	0.454997	0.596316	0.353211	0.528896
CLEC14A	0.468566	0.262289	0.589831	0.317364	0.495547
CLEC1A	-0.08823	-0.57288	-0.56451	-0.51758	-0.33025
CLEC2B	0.206155	-0.25761	-0.34064	-0.33278	0.015269
CLEC3A	0.244161	-0.24254	0.298957	0.273276	-0.58506
CLEC3B	0.271191	0.263396	0.245048	0.234117	-0.02046
CLEC4A	0.585871	-0.14832	0.488099	-0.17152	0.186665
CLEC4D	0.277046	-1.29299	-1.21709	-0.86161	-0.77404
CLEC4E	0.162036	-0.78164	-0.46729	-0.25189	-0.23856
CLEC4F	0.205007	0.036805	0.61527	0.071035	0.155949
CLEC4G	0.159853	-0.40308	-0.15847	-0.09974	-0.04679
CLEC4M	0.097661	-1.43058	-1.08863	-0.93675	-0.92069

CLEC5A	-0.43167	-0.69091	-0.96441	-0.38959	-0.59899
CLEC6A	-0.46566	-0.47492	-0.57562	-0.50334	-0.94049
CLEC7A	0.046377	-0.70848	-0.53208	-0.52379	-0.62752
CLEC9A	0.49635	0.295603	0.166065	0.168946	0.196036
CLECL1	0.391523	-0.4417	0.413642	-0.02374	-0.08815
CLGN	0.321048	0.161033	0.390974	0.048922	-0.00179
CLIC1	-0.33916	-0.16062	-0.2806	-0.20083	-0.05946
CLIC1 MSH5	0.089839	-0.39804	-0.50543	-0.02111	0.329662
CLIC2	-0.13522	0.079509	-0.09411	0.124488	-0.14672
CLIC3	0.143003	0.169525	0.366328	0.14677	0.192167
CLIC4	0.055175	0.077111	0.289269	0.12482	0.115935
CLIC5	0.031301	0.024836	0.004737	0.248199	0.285095
CLIC6	-0.02108	-0.17324	0.320125	0.218824	0.431898
CLINT1	0.228394	0.309253	0.334214	0.004519	0.099193
CLIP1	-0.15017	-0.26174	-0.09199	-0.12764	-0.17432
CLIP3	-0.32886	-0.30702	-0.49285	-0.3713	-0.39533
CLIP4	0.240913	0.061295	-0.23768	0.160191	-0.12238
CLK1	0.326185	0.411778	0.331215	0.211925	0.454815
CLK2	0.107998	0.26129	0.178338	0.06211	0.136035
CLK3	-0.20818	-0.1647	-0.42844	-0.42289	-0.37836
CLK4	0.19469	0.210206	0.281118	0.139191	0.211748
CLLU1OS	-0.48674	-0.21182	-0.30887	-0.54077	-0.55212
CLLU1OS CLLU1	0.32696	0.371697	0.221714	0.132569	0.193928
CLMN	0.131857	0.252075	0.213767	0.108702	0.309864
CLN3	-0.53999	-0.49376	-0.65707	-0.31332	-0.37151
CLN6	-0.06468	-0.08197	-0.05257	-0.08091	-0.20776
CLNS1A	-0.39782	0.049891	-0.31056	-0.39445	-0.49019
CLP1	0.18691	0.321079	0.393516	0.172874	0.291092
CLPB	0.356348	0.393485	0.527659	0.398396	0.380026
CLPP	0.021335	0.102498	-0.01357	-0.06188	-0.06098
CLPS	0.205033	0.2157	0.182799	0.087908	0.149894
CLPTM1	-0.46564	-0.52944	-0.78482	-0.49489	-0.65644
CLPTM1L	0.044459	-0.05559	-0.08218	-0.03096	-0.00073
CLPX	-0.00682	-0.08406	-0.09139	-0.07079	-0.16663
CLRN3	0.088627	0.329585	0.211842	-0.07035	-0.04356
CLSPN	-0.18383	-0.05915	-0.18537	-0.20715	-0.20726
CLSTN1	0.010067	0.365507	0.486155	0.271252	0.3501
CLSTN2	0.069395	0.124073	-0.08987	0.060389	0.038921
CLTA	-0.42015	-0.5023	-0.51661	-0.4903	-0.77278
CLTB	0.15216	0.287438	0.338223	0.141279	0.144344
CLTC	-0.65677	-0.80753	-0.83548	-0.76084	-0.33414
CLTCL1	0.118531	0.096811	0.062904	0.129867	0.168178
CLYBL	-0.05285	-0.08849	-0.39078	-0.27589	-0.14689
CMA1	-0.75396	-0.83838	-0.84306	-0.68439	-0.87288
CMAS	0.067119	0.247554	0.192681	0.163238	-0.02799
CMBL	-0.05139	0.456686	-0.57568	-0.35749	0.200163
CMKLR1	0.164412	0.508779	0.420621	0.219395	0.400628
CMPK1	-0.10663	0.021985	0.133524	0.145348	0.205943
CMPK2	0.193647	0.009033	0.059916	0.049618	0.144091
CMTM1 CMTM2	0.29756	0.442072	0.545507	0.434457	0.028721

CMTM3	-0.02776	-0.04166	-0.13336	-0.13885	-0.08859
CMTM4	-0.00755	0.235542	0.042805	0.089231	0.16599
CMTM6	0.196526	0.404634	0.211398	0.072616	0.026768
CMTM7	0.125505	0.304207	0.271593	0.018415	0.134352
CMTM8	-0.06394	0.125115	-0.03826	0.017878	0.132257
CMYA1	0.455256	0.33865	0.378603	0.312208	0.188001
CMYA5	0.32267	0.488572	0.674074	0.407267	0.543648
CNBD1	-0.13777	-0.1165	-0.17361	-0.43695	-1.08733
CNBP	-0.03556	-0.12029	-0.13954	-0.06209	-0.08471
CNDP1	0.555453	0.687114	0.832212	0.407581	0.492914
CNDP2	-0.12498	-0.30183	-0.18404	-0.20001	-0.19086
CNFN	0.073403	0.041176	0.481784	-0.06648	-0.12099
CNGA1	-0.12535	0.193204	0.099431	-0.28812	-0.33745
CNGA2	-1.11736	-0.75386	-1.19764	-0.37729	-0.97866
CNGA3	0.383956	0.341183	0.612559	0.296434	0.101136
CNGA4	-0.63181	-0.65933	-0.95481	-0.67661	-0.56414
CNGB1	0.114673	0.22333	0.157012	0.004814	0.167108
CNGB3	0.120002	0.273024	-0.06627	-0.28319	-0.26272
CNIH	0.145748	-0.17018	-0.26076	-0.07381	-0.21398
CNIH2	0.038424	0.07143	0.174782	0.113221	0.191187
CNIH3	-0.22875	0.154401	0.280284	0.30639	0.388065
CNIH4	-0.04376	-0.12672	-0.25711	-0.16843	-0.10498
CNKSR1	0.454353	0.711615	0.726002	0.450821	0.419341
CNKSR2	0.127805	-0.12253	0.346816	-0.12453	0.040996
CNN1	0.252569	0.309627	0.422361	-0.02378	0.024581
CNN2	-0.05125	0.029302	0.108975	0.009205	0.01051
CNN3	0.064354	-0.0062	0.076432	0.114487	0.127078
CNNM1	0.283176	0.444356	0.577062	0.336219	0.408953
CNNM2	-0.16216	-0.09227	-0.0019	0.008711	-0.22599
CNNM3	-0.11197	-0.24024	-0.33254	-0.08795	-0.09696
CNNM4	0.111068	0.178927	0.299922	0.22058	0.434207
CNO	0.259418	0.146976	0.195146	0.056549	0.081882
CNOT1	-0.70981	-0.50346	-0.73883	-0.64501	-0.68345
CNOT10	-0.10929	-0.09583	-0.38067	-0.48693	-0.19842
CNOT2	-0.27576	-0.28683	-0.26058	-0.25391	-0.13901
CNOT3	-0.42457	-0.55302	-0.61504	-0.49514	-0.4896
CNOT4	-0.62051	-0.72332	-1.18495	-0.57493	-0.72459
CNOT6	0.144453	0.153146	-0.12374	0.113457	0.038058
CNOT6L	-0.25567	-0.28566	-0.28294	-0.11821	-0.21791
CNOT7 VPS37A	-0.46944	-0.37551	-0.46206	-0.40697	-0.57462
CNOT8	-0.5128	-0.47275	-0.5152	-0.30148	-0.31884
CNP	0.089286	0.14701	0.188997	-0.01022	0.158968
CNPY2	-0.11773	-0.17463	-0.24502	-0.08214	-0.01329
CNR1	0.449101	0.204553	0.686506	0.412842	0.233035
CNR2	0.2783	0.300398	0.457538	0.026854	0.276139
CNTD2	0.355721	0.356398	0.771041	0.478335	0.703922
CNTFR	-0.09213	0.12903	0.073218	0.098344	-0.07717
CNTN2	0.19124	-0.39499	-0.49198	-0.31015	-0.25675
CNTN3	0.208413	0.207484	0.399324	0.143653	-0.34219
CNTN6	0.020009	-0.58153	-0.97077	-0.82435	-0.66581

CNTNAP2	0.061278	-0.0698	-0.02517	0.1553	0.157069
CNTNAP3	-0.08636	0.170463	-0.13556	0.052429	0.237181
CNTNAP5	-0.04771	-0.15095	-0.13706	0.017406	-0.26057
COASY	-5.2E-05	0.108219	-0.15977	-0.05182	0.09637
COBL	-0.07238	0.090615	0.080592	0.041	0.202363
COBLL1	-0.09858	0.081932	-0.11314	0.010966	-0.03688
COBRA1	-0.10393	-0.16176	-0.23653	-0.01424	0.003265
COCH	0.324091	0.412975	0.584969	0.259622	0.47627
COG1	-0.19737	-0.11289	-0.32607	-0.41313	-0.15522
COG2	0.023293	0.104237	0.170406	0.147117	0.041263
COG4 SF3B3	-0.90433	-0.79722	-0.96488	-0.34708	-1.09174
COG5 DUS4L	-0.40462	-0.20106	-0.52465	-0.26466	-0.05404
COG6	-0.46143	-0.39507	-0.5456	-0.38226	-0.43468
COG7	0.04082	-0.01508	0.043626	0.142557	0.180419
COG8	0.392563	0.25585	0.145253	0.145178	0.259283
COG8 NIP7	-0.18937	-0.08283	-0.23687	-0.13882	-0.14686
COIL	0.190855	0.191617	0.058776	-0.10101	0.125037
COL11A1	-0.59457	0.589094	0.574447	0.047048	0.535598
COL11A2	0.305788	-0.05187	-0.09899	0.187201	-0.11
COL12A1	-0.7822	-0.01805	0.335632	-0.06974	0.091857
COL13A1	-0.51581	-0.29175	0.120723	-0.25563	-0.37296
COL14A1	0.182142	-0.13208	0.391884	0.070075	0.020785
COL15A1	0.209347	0.237866	0.321746	0.355681	0.345865
COL16A1	-0.10738	0.127256	-0.07812	0.190149	0.192317
COL17A1	0.215808	0.209115	0.066738	-0.21914	-0.46902
COL19A1	0.054626	-1.2615	-0.97428	0.237484	-0.36638
COL1A1	-0.75188	-0.20358	0.213662	0.101831	-0.14599
COL1A2	0.089761	0.581953	0.549102	0.35697	0.742913
COL20A1	0.179642	0.069853	0.30445	0.079325	0.142813
COL21A1	0.154177	-0.2416	0.138119	0.271675	0.386006
COL22A1	-0.18776	-0.04915	0.491612	0.114636	-0.11204
COL23A1	0.138089	-0.02452	-0.20115	0.089283	-0.22718
COL24A1	0.190054	0.516042	0.034902	0.458659	0.280737
COL25A1	-0.29049	-0.46998	0.228666	-0.30885	-0.11864
COL27A1	-0.43498	-0.36941	-0.36797	-0.25452	-0.42347
COL28A1	-0.20249	-0.22347	-0.31279	-0.27532	-0.56812
COL2A1	-0.14782	0.066614	0.224206	-0.055	-0.03037
COL3A1	0.265003	-0.54752	0.120919	0.199128	0.504303
COL4A1 COL4A2	-0.02045	0.245575	0.056358	0.09908	0.290601
COL4A3BP POLK	-0.48058	-0.67638	-0.7458	-0.54209	-0.73294
COL4A4 COL4A3	-0.01747	0.047052	0.09035	-0.12964	0.171396
COL4A6 COL4A5	0.020352	0.201125	-0.90342	-0.06339	-0.37152
COL5A1	-0.02585	0.013283	0.075095	0.042821	0.002556
COL5A2	-0.51099	0.398679	0.289365	-0.01378	0.159433
COL5A3	-0.14833	-0.67784	-0.23799	-0.01093	-0.43028
COL6A1	-0.34541	-0.34525	-0.49694	-0.36921	-0.38998
COL6A2	-0.08561	0.109704	0.00715	-0.0118	0.011041
COL6A3	0.527143	0.594641	0.823814	0.400804	0.505788
COL7A1	0.27988	0.337272	0.403072	0.402221	0.273816
COL8A1	-0.80057	0.766479	-0.74721	-0.12651	-0.23874

COL8A2	0.598297	0.663254	0.672988	0.398248	0.482727
COL9A2	0.203824	0.313479	0.312678	0.201223	0.179081
COL9A3	0.24229	0.177047	0.557792	0.278059	0.432802
COLEC10	0.251206	-0.31894	-1.30935	-0.55908	-0.82712
COLEC11	0.466366	-0.14783	0.113972	0.524033	-0.09484
COLEC12	-0.19592	-0.31044	0.058501	0.082424	0.158374
COMMD1	-0.33848	-0.26912	-0.6135	-0.34518	-0.1036
COMMD10	0.107568	-0.00369	0.22259	0.080362	0.067305
COMMD2	0.079601	0.18945	-0.00861	-0.06902	0.017935
COMMD3	-0.25562	-0.06222	-0.05832	-0.08758	-0.14035
COMMD4	-0.00626	-0.20952	-0.08684	-0.27104	-0.49435
COMMD5	0.085284	-0.03407	0.197253	0.00511	0.096436
COMMD6	-0.2663	-0.39179	-0.4587	-0.47385	-0.45036
COMMD7	0.044534	0.328601	0.072995	0.268115	0.245039
COMMD8	-0.03672	-0.00929	-0.03289	-0.16578	-0.11827
COMMD9	-0.28781	-0.58753	-0.61	-0.36281	-0.62423
COMP	0.043428	0.072961	0.43751	0.223136	0.181417
COMT	0.246018	0.316158	0.428022	0.282292	0.376557
COMTD1	0.051541	0.138703	-0.0675	0.013947	0.009103
COPA NCSTN	-0.46799	-0.89501	-1.13145	-0.99162	-0.89825
COPB1	-0.47087	-0.51101	-0.62821	0.104045	-0.34078
COPB2	0.025802	-0.14323	-0.17674	-0.09227	0.061893
COPE DDX49	-0.17796	-0.21176	-0.57709	-0.14502	-0.35186
COPEB	-0.15963	-0.08453	-0.0619	-0.13599	-0.03616
COPG	-0.19789	-0.07568	-0.28895	-0.14264	-0.27596
COPS2 GALK2	-0.44997	-0.44427	-0.41217	-0.26637	-0.3383
COPS3	0.037113	0.118189	-0.43885	-0.22281	-0.25535
COPS4	-0.19472	-0.29972	-0.68056	-0.38439	-0.46911
COPS5	-0.1646	-0.33424	-0.15628	-0.20145	-1.14638
COPS6	-0.12124	-0.20907	-0.25408	-0.25732	-0.16849
COPS7A	-0.48946	-0.55357	-0.87592	-0.6031	-0.45987
COPS7B	0.246308	0.170425	0.046446	0.159149	0.127547
COPS8	-0.05382	0.12065	0.016546	0.013233	0.011366
COPZ1	0.18305	0.277975	0.236702	0.098254	-0.17111
COPZ2	0.163788	0.156354	0.079874	0.088798	-0.00183
COQ10A	0.048657	-0.01684	-0.25284	-0.03033	-0.0078
COQ10B	-0.51272	-0.28533	-0.5133	-0.43796	-0.62705
COQ2	0.111396	0.028239	0.135502	-0.04893	-0.07191
COQ3	0.406003	0.088984	0.179118	0.282482	0.369654
COQ5	-0.07193	0.14401	-0.20976	-0.53418	-0.1189
COQ7	0.127288	-0.03445	0.105196	0.028156	0.013668
CORIN	0.255388	-0.37536	0.483984	0.411564	-0.28578
CORO1B	0.148167	0.140301	-0.0831	0.120515	0.373296
CORO1C	-0.33471	-0.31845	-0.61354	-0.51751	-0.59922
CORO6	0.33136	0.266766	0.443437	0.349493	0.276329
COTL1	-0.29762	0.025281	-0.10407	-0.0711	-0.27618
COX10	0.142599	-0.00929	0.209739	-0.07324	-0.04362
COX11 STXBP4	-1.04076	-1.12492	-1.5849	-1.41439	-1.12568
COX15 CUTC	-0.32028	-0.34454	-0.54181	-0.4219	-0.54333
COX17	0.168248	0.84669	0.712653	0.309171	0.571509

COX18	0.225365	-0.02446	-0.10248	-0.17399	-0.18894
COX19	0.156235	0.075959	-0.17072	0.174841	0.097343
COX4I2	0.331518	0.471137	0.404645	0.315841	-0.01738
COX4NB COX4I1	0.058396	0.108582	0.167593	0.075458	0.052637
COX5A	-0.00313	-0.11955	-0.08466	-0.05735	0.105102
COX5B	-0.03997	0.046788	-0.35487	0.025089	0.005485
COX6A1	-0.21085	-0.41339	0.286013	-0.24347	-0.52342
COX6A2	0.234771	0.374223	0.571591	0.143527	0.448997
COX6B1	0.18796	-0.05264	-0.21095	-0.10287	-0.06937
COX6B2 AC020922.9	-0.13639	-0.28387	-0.39506	-0.19652	-0.19312
COX6C	-0.26208	-0.60514	-0.97479	-0.53076	-0.78066
COX7	0.429085	0.467576	0.408765	0.188381	0.08687
COX7A1	0.421212	0.510569	0.554738	0.260881	0.484162
COX7A2	-0.10128	-0.36674	-0.63888	0.186357	-0.79485
COX7A2L	-1.21113	-1.18874	-1.50464	-0.86354	-1.384
COX7B	0.410344	0.314591	0.403228	0.052109	0.178305
COX8A	0.05151	0.296636	0.129924	0.171058	0.193946
CP	0.082693	0.409123	0.250415	-0.01737	0.275332
CPA1	0.060325	0.137548	-0.03643	0.15266	0.040423
CPA2	-0.20002	-0.05616	-0.42176	-0.27185	-0.58133
CPA3	0.212339	0.328411	0.256362	0.227015	0.14928
CPA4	-0.01288	0.146545	0.09034	-0.12932	-0.13392
CPA6	0.479775	0.005313	-0.99801	0.531716	-0.14965
CPAMD8	-0.15352	-0.0804	0.064244	-0.07154	0.074735
CPB1	0.230072	0.455437	0.406318	0.058131	0.234415
CPB2	-0.10785	-0.5379	-0.48249	-0.92346	-1.2222
CPD	0.221584	0.20165	0.179764	0.190265	0.227468
CPE	0.059004	-0.00825	-0.01733	-0.07154	0.00957
CPEB1	0.062004	0.071862	0.333462	0.019997	-0.11234
CPEB2	-0.21891	-0.12003	-0.16454	-0.14803	-0.02322
CPEB3 MARCH5	-0.2964	-0.14096	-0.19713	-0.18515	-0.12211
CPEB4	-0.18923	-0.76256	-0.50765	-0.33413	-0.70122
CPLX3	0.167651	0.189284	0.479724	0.314407	0.466221
CPLX4	0.273307	0.106059	0.003158	-0.44557	-0.04085
CPN1	0.656343	0.858867	0.817107	0.421061	0.443574
CPNE2	-0.07747	0.00906	0.003035	0.358865	0.09335
CPNE3	0.10374	0.523171	0.411429	0.213516	0.345452
CPNE4	-0.53775	-0.52152	-0.64634	-0.16236	-0.69068
CPNE5	-0.15989	0.112244	0.33978	-0.01666	0.14971
CPNE6	0.375322	0.44292	0.712374	0.418534	0.563247
CPNE7	-0.02318	-0.08266	0.243889	0.116398	0.121072
CPNE8	-0.29779	-0.39386	0.626319	0.479054	-0.35602
CPNE9	0.159083	0.103302	0.034309	0.019286	0.207247
CPO	-0.1241	0.095709	-0.21681	-0.41405	-0.1664
CPOX	0.068932	-0.03503	0.079308	0.016627	0.089168
CPS1	-0.35737	-0.35031	-0.39338	-0.53022	-0.38458
CPSF1	0.03968	-0.00381	-0.06897	-0.07231	0.161776
CPSF3L GLTPD1	-0.11962	-0.39789	-0.21338	-0.1079	-0.31232
CPSF6	-0.03939	0.077965	-0.09747	-0.02873	-0.21803
CPT1A	0.096436	0.213985	0.283748	0.272617	0.303906

CPT1B	0.14417	0.261319	0.2429	0.156094	0.339712
CPT1B U62317.2	0.016425	-0.09904	-0.17376	-0.08311	-0.13116
CPT1C	-0.26009	-0.44941	-0.75036	0.062301	-0.3451
CPT2	0.247199	0.514568	0.518669	0.205846	0.393272
CPVL	0.009043	0.233222	0.249807	0.220578	-0.18391
CPXCR1	0.31221	0.261417	-0.01707	0.149055	0.285298
CPXM1	-0.1475	0.175699	0.138742	0.035559	0.183177
CPXM2	0.153823	0.426475	0.421716	0.314308	0.34415
CPZ	0.021412	0.182199	0.289332	0.124347	0.261684
CR1	0.316306	0.138941	0.111482	0.491093	0.615853
CR2	-0.03798	-0.14069	0.177267	0.31873	0.271404
CRABP1	-0.12797	0.357886	0.375956	0.26084	0.374261
CRABP2	-0.06403	-0.58125	0.452661	-0.26572	-0.31764
CRADD	0.19059	-0.10731	-0.27462	-0.08078	-0.50576
CRAMP1L	-0.12186	0.015474	0.041083	-0.19412	-0.02748
CRAMP1L C16orf34	-0.14607	-0.06762	0.083735	-0.04474	-0.0309
CRAT PPP2R4	0.08056	0.033181	-0.01152	0.027971	0.032388
CRB1	-0.47437	-0.42002	-0.87765	-0.8922	-0.66444
CRB2	-0.64667	0.214041	0.186412	0.231325	0.354045
CRB3	0.107948	0.15477	0.36373	0.214001	0.126583
CRBN	0.051565	0.031103	0.12436	-0.03876	-0.04959
CRCT1	-0.27597	-1.22308	-1.32216	-1.1242	-0.3742
CREB1	-0.00678	0.141294	-0.04468	0.071622	-0.02071
CREB3L1	-0.0623	0.471109	0.511294	0.310599	0.243809
CREB3L2	-0.05527	-0.01895	-0.10948	-0.17063	-0.27146
CREB3L3	0.296643	-0.1814	0.309093	0.207521	0.286045
CREBL1 FKBPL	-0.12621	-0.26458	-0.51027	-0.11514	-0.36311
CREBL2	0.046219	0.002202	-0.10805	-0.19818	-0.1071
CREBZF	-0.14486	0.271694	-0.0108	-0.15915	-0.01158
CREG1	-0.08383	-0.12552	-0.32606	-0.36088	-0.18272
CREG2	0.083456	-0.04918	0.004945	0.018112	-0.00797
CRELD1	0.065086	0.025239	0.171187	0.196798	0.116475
CRH	0.493569	-0.64175	0.491182	0.219351	-0.54253
CRHBP	0.224822	0.388935	0.495069	0.166128	0.069117
CRHR1	-0.22393	-0.23488	-0.32631	-0.29418	-0.14185
CRHR2	-0.00358	0.303709	0.28719	0.063116	0.374522
CRIM1	-0.04801	0.027481	0.262507	0.189313	0.219825
CRIP2	0.095013	-0.01795	0.118983	0.146835	0.231364
CRIP3	0.54108	0.680908	0.850675	0.537836	0.22162
CRISP1	-0.65562	-0.60276	-0.51776	-0.33958	-0.63178
CRISP2	0.74348	0.306971	0.228401	0.791364	0.343166
CRISPLD1	-0.18957	-0.19917	-0.08304	-0.26638	-0.11972
CRISPLD2	0.395595	0.413412	0.622629	0.326948	0.413497
CRK	0.070247	-0.02069	-0.00794	-0.03083	-0.03905
CRKL	0.048312	0.06613	0.036568	-0.05427	0.116201
CRKRS	-0.23435	-0.00394	-0.3527	-0.31767	-0.40713
CRLF1	-0.04891	-0.05063	0.026574	0.021536	-0.06702
CRLF3	-0.032	-0.00727	-0.10855	-0.16311	-0.01747
CRLS1	0.060042	-0.05821	0.155371	0.036752	-0.03349
CRNKL1	-0.61022	-0.53517	-0.57303	-0.47744	-0.42741

CRNKL1 C20orf26	0.519988	0.868969	0.943038	0.45561	0.450392
CRNN	-0.04032	-1.21882	-0.82056	-0.63321	-0.50655
CROCC	0.046981	0.147055	0.326122	-0.08338	0.03915
CRP	0.226703	-1.08131	-1.18098	-0.83239	-0.52688
CRSP3	-0.56838	-0.13996	-0.34358	-0.42471	-0.55609
CRSP9	-0.59571	-0.07621	-0.71994	-0.56465	-0.52875
CRTAC1	0.046419	0.117396	0.106096	-0.01326	0.154171
CRTAM	-0.11729	-0.0608	-0.26947	-0.45187	-0.45094
CRTAP	0.24338	0.296622	0.393766	0.244182	0.419033
CRTC1	0.04702	0.077612	0.161857	0.012542	0.010743
CRTC2 SLC39A1	-0.82015	-0.88894	-1.26942	-0.88568	-0.91836
CRTC3	0.12159	0.038424	0.028001	0.191679	0.156569
CRX	0.542829	-0.66881	-0.3907	-0.19384	0.370649
CRY1	-0.27209	-0.28264	-0.17315	-0.15962	-0.19691
CRY2	0.321993	0.274529	0.373265	0.082653	0.311456
CRYAA	0.513327	0.629399	0.908518	0.454243	0.534427
CRYAB AP000907.6	0.297952	0.321429	0.510859	0.298508	0.455257
CRYBA1	-0.25205	-0.0199	-0.02287	-0.07662	-0.1316
CRYBA2	0.043469	0.381837	0.587413	0.311321	0.552022
CRYBA4	-0.01576	0.223861	0.157775	0.048749	0.053017
CRYBB1	0.104323	0.213728	0.144353	0.116365	0.38081
CRYBB3	0.479428	0.591643	0.680904	0.533502	0.597755
CRYGA	0.115651	0.298077	0.216884	-0.03708	0.197558
CRYGB	0.597535	0.9185	0.95807	0.529215	0.792532
CRYGC	0.477211	0.513046	0.589457	0.568948	0.38169
CRYGD	0.456658	0.985038	0.870554	0.461604	0.318954
CRYGN	0.002498	0.395631	0.389595	0.232591	0.172288
CRYGS	-0.05966	-0.16857	-0.46018	-0.55678	-0.42436
CRYL1	0.056643	-0.03236	-0.00707	0.045499	-0.10895
CRYM	-0.23104	-0.37224	-0.5657	0.075086	-0.36056
CRYM AC008740.7	0.23791	0.230528	0.293405	-0.30446	-0.63503
CRYZ TYW3	-0.96476	-1.38302	-1.30087	-1.02314	-0.54628
CRYZL1 ITSN1	-0.43128	-0.48117	-0.40677	-0.26318	-0.36548
CS	0.294509	0.027103	0.059188	0.149031	0.063104
CSAD	-0.44009	-0.41946	-0.42829	-0.28328	-0.34216
CSAG4. CSAG1	0.327228	0.304571	-0.37329	0.255936	0.360267
CSDA	-0.14968	-0.03085	0.126654	0.102239	0.277105
CSDC2	0.014005	-0.01645	-0.26914	-0.49986	-0.29366
CSDE1	-0.46813	-0.30479	-0.19628	-0.47062	-0.44649
CSE1L	0.275551	-0.08166	0.18029	-0.01198	0.248058
CSF1	-0.2611	-0.27246	-0.16615	-0.24301	-0.13986
CSF1R PDGFRB	0.205857	0.51809	0.592056	0.19007	0.410244
CSF2	0.109201	0.31346	0.310698	0.102755	0.254865
CSF2RB	0.176163	0.431061	0.396409	0.222144	0.381201
CSF3	0.188407	0.329457	0.483875	0.215241	0.284572
CSF3R	0.078642	0.228324	0.258955	0.04135	-0.01671
CSGALNACT1	0.652976	0.266808	-1.1216	0.537586	0.070315
CSGALNACT2	0.0252	0.012881	0.029387	0.014495	-0.08348
CSH2 CSH1	0.351126	0.388729	0.552693	0.416632	0.492082
CSHL1	0.257024	0.406679	0.361852	0.454877	0.360864

CSK	-0.02301	-0.20498	-0.27678	-0.23306	-0.31587
CSN1S1	-0.14835	-0.49453	-0.23389	-0.42975	-0.02846
CSN2	-0.32712	-0.58502	-0.26772	-0.46272	-0.45506
CSN3	0.111105	-0.40861	-0.06918	-0.48525	-0.20071
CSNK1A1	-0.37813	-0.29841	-0.58612	-0.32966	-0.37425
CSNK1A1L	0.763683	0.754712	0.922723	0.403004	0.599899
CSNK1D	0.063278	0.008995	0.067021	0.029711	0.059386
CSNK1G1	-0.27491	-0.329	-0.32759	-0.31938	-0.44148
CSNK1G3	-0.25735	0.016992	0.084884	-0.14613	0.013312
CSNK2A1	0.055039	0.186616	-0.03622	-0.24926	0.02016
CSNK2A2	-0.23255	-0.3805	-0.47956	-0.23424	-0.26795
CSPG2	-0.85705	-0.5851	-0.61812	-0.52536	-0.40855
CSPG4	0.095856	0.174041	0.318555	0.294429	0.410544
CSPG5	-0.06368	-0.22113	-0.36165	-0.19438	-0.09564
CSRP1	0.240833	0.689489	0.517061	0.465623	0.739781
CSRP2	-0.02987	0.053359	0.002305	0.002151	0.123551
CSRP3	-0.11729	-0.28315	-0.01576	-0.1392	-0.30522
CST1	0.033486	-0.79168	-1.0461	-0.28736	-0.41006
CST11	0.379157	0.318934	0.279371	0.163404	0.276959
CST2	0.001086	-0.53289	-0.71329	-0.13408	-0.13949
CST3	-0.26872	-0.13447	-0.24532	-0.14917	-0.09422
CST4	0.121488	-0.61342	-1.17686	-0.19388	-0.48369
CST5	0.101541	-0.6758	-0.61039	-0.09197	-0.53022
CST6	0.370662	0.300601	0.338824	0.209226	0.317804
CST7	0.33711	0.476125	0.001935	0.296194	0.341684
CST8	0.010294	-0.11073	-0.11261	-0.04054	0.0069
CST9	0.218407	0.259399	0.361265	0.056448	0.134541
CST9L	-0.02967	-0.58052	-0.53389	-0.63935	-0.48334
CSTA	0.150406	0.521783	0.557569	0.018087	-0.4283
CSTB	0.11014	0.149677	-0.0084	0.133516	0.079034
CSTF2	0.393839	0.129471	0.2108	0.051425	0.012532
CSTF3	-0.09301	-0.03684	-0.1098	-0.37439	0.275494
CSTL1	0.454176	0.518522	0.606239	0.042593	0.292602
CTA-126B4.5	0.121699	0.117423	0.106809	0.118149	0.018471
CTAG1B	0.436979	0.243796	0.612908	0.289634	0.46433
CTAG2	0.455181	0.406225	0.603958	0.396409	0.379955
CTAGE	0.026523	-0.15606	-0.0279	-0.18767	0.074841
CTBP1 C4orf42	0.146253	0.243331	0.163519	0.199033	0.19732
CTBP2	-0.21597	-0.41158	-0.34646	-0.18528	-0.25079
CTBS	-0.22018	-0.07002	-0.60678	-0.36593	-0.16462
CTCF	0.055795	0.120398	0.093137	-0.02709	0.082646
CTCFL	-0.23952	-0.13242	-0.09513	-0.24666	-0.09501
CTDSP1	-0.07552	-0.33562	0.057002	0.133099	0.210213
CTDSP2	-0.06308	0.046393	-0.02094	-0.03623	0.141485
CTDSPL	-0.10471	-0.04856	-0.19883	-0.0848	-0.00146
CTDSPL2	-0.75141	-0.59223	-1.18274	-0.66517	-0.92817
CTGF	-0.56933	-0.32453	0.256121	0.01914	0.159513
CTH	-0.69221	-0.38136	-0.8057	-0.62639	-0.29442
CTHRC1	0.239561	0.160423	0.103093	0.090134	0.14579
CTLA4	0.239505	0.456315	0.466195	0.194117	-0.09926

CTNNA2	-0.09429	-0.05968	-0.15796	-0.01983	0.137577
CTNNAL1	-0.17926	-0.16933	-0.18701	-0.23086	-0.15839
CTNNB1	-0.14093	-0.0853	-0.17045	-0.16612	0.009474
CTNNBIP1	-0.48251	-0.36953	-0.66968	-0.21341	-0.252
CTNNBL1	-0.6463	-0.69524	-0.90627	-0.65707	-0.95981
CTNND1	0.301988	0.001253	0.162598	0.0419	0.066118
CTNND2	-0.04	0.057729	0.115488	-0.15395	0.159749
CTPS	0.121019	0.200231	0.206647	0.151333	0.185269
CTR9	-0.10042	-0.17189	-0.09006	-0.08543	-0.01701
CTRB1	0.110573	0.269169	0.339401	0.292235	0.376588
CTRB2	0.267318	0.292379	0.568074	0.235432	0.403072
CTRC	0.303475	0.475948	0.690895	0.338089	0.672947
CTRL	0.174208	0.478604	0.417556	0.282327	0.448529
CTSB	0.059397	0.252637	0.048643	0.136381	0.094874
CTSC	-0.35483	-0.23698	-0.49561	-0.19744	-0.53948
CTSD	0.174943	0.227484	0.129473	0.156009	0.194736
CTSE	0.284882	0.617814	0.554513	0.365918	0.212646
CTSF	0.323168	0.242342	0.18036	-0.00129	0.282134
CTSG	0.287583	-0.96122	-1.00548	-0.99654	-0.81096
CTSH	0.147978	-0.07455	0.06719	-0.3009	0.095803
CTSK	0.369608	0.569653	0.710891	0.370067	0.235811
CTSL1	0.027651	-0.01094	-0.11878	-0.1282	-0.02095
CTSL2	0.195966	0.217364	0.117805	0.294134	0.044018
CTSO	0.217425	0.030836	-0.03518	0.098673	0.237007
CTSS	0.273372	-0.12482	0.133659	-0.32355	-0.19215
CTSW	0.286128	0.332163	0.476706	0.194865	0.1527
CTSZ	0.168597	0.324288	0.379373	0.02908	0.407273
CTTN	-0.04515	-0.19116	-0.22292	-0.204	-0.22052
CTTNBP2	0.115075	0.245469	-0.06544	0.084688	-0.18493
CTTNBP2NL	-0.63102	-0.60078	-0.60273	-0.62755	-0.55507
CUBN	-0.43963	-0.45877	-0.85894	-0.68523	-0.56805
CUEDC1	-0.12222	-0.27952	-0.26148	-0.29711	-0.3469
CUEDC2	-0.42309	-0.43908	-0.7638	-0.34806	-0.30352
CUL1	0.096966	-0.13243	-0.08704	0.106739	0.014935
CUL2	-0.44535	-0.41663	-0.51996	-0.47636	-0.61018
CUL3	-0.49817	-0.50939	-0.53086	-0.50347	-0.44441
CUL4B	0.449476	0.859617	0.470346	0.345536	0.366148
CUL5	0.449839	0.512239	0.409974	0.235007	0.305265
CUL7 MRPL2	0.339729	0.161609	-0.17827	0.069292	0.272893
CUTA	-0.16129	-0.21176	-0.31288	-0.31256	-0.10156
CUX1	-0.07062	-0.08019	-0.09886	-0.14341	0.012136
CUX2	-0.10171	-0.13712	-0.10733	-0.06007	-0.11608
CWC15 JMJD2D	-0.41985	-0.39917	-0.81984	-0.47803	-0.65747
CWF19L1	-0.05447	0.007791	0.150897	0.080416	-0.10797
CWF19L2	-0.25591	-0.00866	-0.11405	-0.16262	-0.32937
CX3CL1	0.069905	0.088226	0.138895	0.06855	0.115008
CX3CR1	0.122342	0.168721	0.240119	-0.14556	-0.0569
CXADR	-0.05001	0.02197	-0.08457	0.009101	-0.01309
CXCL1	0.128391	-0.31961	0.166665	0.158488	0.1554
CXCL10	-0.16688	0.127679	-0.12126	-0.22343	-0.46832

CXCL11	0.334827	0.21157	0.081777	-0.31871	-0.21998
CXCL12	0.22999	0.376801	0.492018	0.261449	0.301135
CXCL13	-0.22974	-0.82372	-1.01814	-0.81528	-1.06236
CXCL14	-0.0728	0.021726	0.019578	0.143801	0.079601
CXCL16 ZMYND15	0.248057	0.291022	0.576508	0.364838	0.339043
CXCL17	0.304081	0.061035	0.303302	0.095591	-0.25302
CXCL2	0.145215	-0.14396	0.016457	0.289651	0.119454
CXCL3	-0.20163	-0.27647	-0.37881	0.007947	-0.11218
CXCL5	0.145815	-0.29326	-0.12915	0.043485	0.193896
CXCL6	-0.09489	-0.1651	-0.25513	0.091277	0.0928
CXCL9	-0.93387	-0.64034	-0.84274	-0.77831	-0.88888
CXCR3	0.04018	0.240364	-0.13859	0.127163	0.411014
CXCR7	-0.22897	-0.68548	-0.57484	-0.29567	-0.52294
CXorf15	-0.39299	-0.20671	-0.70356	-0.41174	-0.50911
CXorf20	0.554801	0.706424	0.86351	0.382784	0.565838
CXorf21	0.766383	0.735576	0.523045	0.49571	0.594011
CXorf22	0.283248	0.237264	-0.63814	0.22485	-0.17537
CXorf23	0.286997	0.547004	0.008972	0.115874	0.198689
CXorf26	-0.19682	-0.60592	-0.10625	-0.33742	-0.4744
CXorf34	0.274912	0.178223	0.187488	0.157069	0.339202
CXorf36	-0.08639	0.184917	-0.21864	0.107187	-0.50937
CXorf38	-0.20616	-0.21895	-0.46488	-0.16402	-0.31409
CXorf39	0.028004	0.104913	0.194052	0.09362	-0.00313
CXorf40A	-0.29757	-0.72654	-0.32609	-0.45345	-0.76847
CXorf48	0.105778	0.36581	-0.20375	0.199566	0.123256
CXorf56	0.250886	0.00945	-0.31986	0.082809	0.05619
CXorf57	-0.13072	-0.46401	0.140305	-0.13064	-0.21473
CXorf59	-0.4291	0.083782	-0.06918	-0.0449	-0.11926
CXorf61	0.408167	0.659786	0.328828	0.483679	0.454544
CXXC1	-0.59492	-0.40729	-0.61783	-0.50807	-0.44236
CXXC4	-0.71908	-0.79832	-0.78183	-0.50845	-0.58038
CXXC5	-0.02437	0.145081	-0.2943	0.103199	0.088708
CYB561D1	0.048411	-0.10852	-0.19657	-0.23097	-0.14053
CYB5A	0.194194	-0.00277	-0.11118	0.160184	0.092974
CYB5B	-0.29647	-0.11869	-0.57071	-0.12969	-0.36047
CYB5R1	-0.18498	-0.25427	-0.15067	-0.01881	0.064638
CYB5R2	0.416887	0.407193	0.668637	0.478439	0.36902
CYB5R4	-0.55261	-0.24657	-0.42712	-0.36798	-0.36239
CYBA	0.181457	0.259196	0.080494	0.176954	-0.00214
CYBASC3 TMEM138	0.079487	-0.46678	-0.62892	-0.2706	-0.02583
CYBB	-0.15237	-0.15351	-0.17218	-0.14623	-0.12681
CYBRD1	-0.19595	-0.383	-0.59926	-0.41571	0.195747
CYC1	0.148823	0.31192	0.410706	0.254388	0.319977
CYCS	-0.3022	-0.66873	-0.6994	-0.50454	-0.60117
CYGB PRCD	-0.01413	-0.43485	-0.13202	0.134825	-0.13904
CYLC2	-0.5752	-0.34932	-0.35671	-0.21856	-0.38494
CYLD	-0.03464	0.017003	-0.22333	-0.06308	-0.17268
CYLN2	0.048434	0.143127	0.017633	0.082518	0.101835
CYorf15A.	0.056257	0.400307	0.386481	0.0975	0.218581
CYorf15B	-0.12032	0.189308	0.255102	-0.15924	0.129218

CYP11A1	0.286158	0.270683	0.202433	0.042928	0.234803
CYP11B1	0.187045	-0.61631	-0.14404	-0.16261	-0.37778
CYP11B2	0.356905	-0.71569	0.147525	0.181134	0.066484
CYP17A1 RP11-753C18.4	0.294901	0.338113	0.203867	0.13086	0.243832
CYP19A1	0.166091	0.150706	0.025244	-0.13137	-0.06621
CYP1A1	-0.12067	-0.1777	-0.08212	-0.13396	-0.18604
CYP1A2	-0.277	-0.03519	-0.33829	-0.61776	-0.39413
CYP1B1	-0.10801	-0.08642	0.660416	0.371234	0.40002
CYP20A1	0.457447	0.119853	0.377772	0.225555	0.33131
CYP24A1	0.042794	0.166187	0.024042	0.256512	0.292083
CYP26A1	-0.19243	0.006343	-0.01398	0.148635	0.176288
CYP26B1	-0.26468	0.053954	0.118012	-0.13001	0.049366
CYP26C1	0.085884	0.488031	0.499464	0.361791	0.547821
CYP27A1	0.341845	0.376683	0.72661	0.419952	0.521551
CYP27B1	-0.42865	-0.40886	-0.44156	0.059895	-0.49427
CYP27C1	0.351284	0.441358	0.568538	-0.14699	0.11216
CYP2A13	-0.60273	-1.85291	-1.77886	-1.59698	-1.59897
CYP2A6	-0.42865	-1.33654	-1.31682	-1.13789	-1.03712
CYP2A7	-0.69013	-2.39214	-2.10498	-1.88893	-1.98946
CYP2B6	0.196486	-0.88866	-0.94898	-0.93557	-0.95525
CYP2C18	0.129828	-0.04598	-0.10156	0.084289	-0.28412
CYP2C19	-0.49981	-0.962	-0.77458	-0.68706	-1.30824
CYP2C8	-0.04654	-0.53259	-0.23769	-0.32519	-0.51004
CYP2C9	-0.73343	-0.92971	-0.71114	-0.68651	-1.06992
CYP2D6	0.333001	0.282223	0.35272	0.166794	0.234457
CYP2F1	0.043526	-0.96929	-0.81271	-0.85649	-0.63828
CYP2J2	0.088019	-0.27161	-0.44862	-0.24726	-0.32604
CYP2R1	-0.05229	0.046884	-0.00089	-0.0181	0.121105
CYP2S1	0.191566	-0.38779	0.181329	0.299006	0.331742
CYP2U1	0.083831	0.163632	-0.18415	0.101724	-0.08039
CYP2W1	0.069972	0.119981	0.079853	-0.03161	0.072479
CYP39A1 SLC25A27	-0.36679	-0.51836	-0.20992	-0.01453	-0.81957
CYP3A4	-0.54503	-1.19339	-1.55168	-1.23101	-1.33656
CYP3A43	-0.4569	-0.79406	-1.14808	-0.89624	-0.9085
CYP3A5	-0.03309	-0.84138	-2.02078	-1.38806	-1.62342
CYP3A7	-0.4033	-1.16478	-1.43905	-1.10289	-1.4209
CYP46A1	-0.24598	-0.11284	-0.21866	-0.14008	-0.1658
CYP4A22	-0.31615	-0.63745	-0.90226	-0.38485	-0.50256
CYP4B1	-0.23295	-0.06347	-0.47741	0.002841	-0.02116
CYP4F11	-0.27837	-0.582	-0.75367	-0.30744	-0.50157
CYP4F12	0.448562	-0.36102	-0.15547	-0.17708	-0.19776
CYP4F2	-0.14596	-0.24282	-0.49802	-0.22849	-0.26956
CYP4F3	0.003391	-0.51452	-0.48233	-0.53646	-0.48706
CYP4F8	0.072308	-0.763	-0.71262	-0.2167	-0.02116
CYP4V2	-0.04597	-0.17659	-0.13323	-0.00338	-0.17862
CYP4X1	0.497253	0.739844	0.696052	0.189766	0.582768
CYP4Z1	-0.78896	-0.7138	-0.74308	-0.3707	-0.40781
CYP51A1	0.081578	0.005655	-0.27864	-0.14667	-0.00226
CYP7A1	-0.55027	-0.42324	-0.54264	-0.53171	-0.54151
CYP7B1	0.325128	0.472646	0.645648	0.431285	0.561084

CYP8B1	-0.28199	0.092241	-0.36385	-0.36299	-0.15163
CYR61	-0.37094	0.177647	0.132925	0.103323	0.314751
CYS1	-0.04947	0.020648	0.426065	0.114942	0.134226
CYSLTR1	-0.33404	-0.3551	-0.56685	-0.39151	-0.41248
CYSLTR2	0.28903	0.41649	0.437839	0.0555	0.234796
CYTL1	0.0798	0.041764	-0.20378	0.057524	0.145547
CYYR1	-0.07502	0.680187	0.833626	0.115263	0.576241
D2HGDH	0.013831	0.111814	0.14997	0.098794	0.053816
D4ST1	-0.02104	-0.06848	-0.0617	-0.03231	-0.0058
DAAM1	-0.49629	-0.6217	-0.72391	-0.39438	-0.54976
DAAM2	0.263589	0.235766	0.220142	-0.06637	0.137292
DAB1	-0.08714	0.393968	0.005853	-0.02107	0.262664
DAB2	-0.14979	-0.66877	-0.04298	0.187986	-0.23142
DACH1	-2.1818	-2.14713	-2.12866	-1.72398	-1.55163
DACH2	0.052427	0.094457	0.378404	0.227812	0.319055
DACT1	-0.0953	0.06822	0.17109	-0.04913	0.222176
DACT2	0.363048	0.388959	0.706148	0.330821	0.461009
DACT3	0.589866	0.698441	0.76996	0.489029	0.765524
DAD1	-0.53225	-0.41914	-1.10262	-0.65771	-0.74539
DAG1	-0.10086	-0.14091	-0.17159	-0.09619	-0.10007
DAGLA	0.10121	0.028555	0.011418	0.000643	0.230787
DAGLB	-0.01795	0.103331	-0.11065	0.052683	0.023036
DALRD3 C3orf60	0.191298	0.212064	0.186694	0.129501	0.24855
DAND5	0.178987	0.222999	0.097376	0.177577	0.239755
DAO	0.148154	0.368301	0.392754	0.096748	0.300622
DAOA	-0.58772	-0.48422	-0.60684	-0.55609	-0.60028
DAP	-0.02673	0.079651	0.110473	0.05995	0.054586
DAPK1	0.226556	0.523047	0.109178	0.166032	0.149456
DAPK2	0.285764	0.303494	0.465813	0.276495	0.358106
DAPK3	-0.17158	-0.08293	0.201291	0.145121	-0.21709
DAPL1	0.150182	0.148441	-0.01212	-0.10043	0.015931
DAPP1	0.220761	0.20041	-0.03408	-0.15618	-0.19094
DARC	0.147657	-2.0539	-1.46722	-1.32768	-0.79306
DARS	0.2077	0.15192	0.240208	-0.02448	0.224028
DAXX	-1.25119	-0.97156	-1.47221	-0.89816	-1.17275
DAZ1	0.4459	0.239113	0.45981	0.227845	0.112417
DAZ2	0.462934	0.228088	0.483164	0.386052	0.231017
DAZ3	0.348099	0.317923	0.572507	0.385253	0.308338
DAZAP1	0.046406	0.112174	0.33032	0.147934	0.26596
DAZAP2	0.078941	-0.1692	-0.4	-0.01539	-0.12751
DAZL	0.25447	0.306336	0.483924	0.156528	0.242824
DBC1	-0.27748	-0.04706	-0.42292	-0.25102	-0.02746
DBF4B	-0.7411	-0.85588	-1.29396	-1.12008	-1.46817
DBH	0.536736	0.507757	-0.11081	0.01119	0.082942
DBN1	-0.14299	0.030803	0.073943	-0.08527	-0.0263
DBNDD1	0.427168	0.434585	0.743607	0.24982	0.340065
DBNDD2	-0.04404	0.184651	0.052266	0.174787	0.040097
DBNL	-0.19537	-0.12038	-0.23317	-0.09281	-0.00229
DBP	-0.55992	-0.59527	-0.74041	-0.50298	-0.53486
DBR1	0.212819	0.260407	0.064938	0.175128	0.028103

DBT	-0.77401	-0.56093	-0.61365	-0.72026	-0.62261
DBX1	-0.18059	-0.11423	0.430728	0.519337	0.009578
DBX2	0.054762	0.137916	-0.07089	-0.02546	0.211797
DCAKD NMT1	-0.27787	-0.45557	-0.52449	-0.30477	-0.32574
DCAMKL1	0.257245	0.074719	0.410932	0.097477	0.39486
DCBLD1	0.091246	0.165728	0.005348	0.191752	0.136251
DCBLD2	-0.46123	-0.14317	-0.35583	-0.3121	-0.23689
DCD	-0.40224	-0.66652	-0.88489	-0.67102	-0.46474
DCDC2 KAAG1	0.084847	0.438646	0.475259	0.155053	0.209497
DCHS1	-0.50404	-0.14073	-0.02679	-0.39006	-0.05365
DCI	0.253376	0.053183	0.087933	0.220005	-0.06658
DCK	0.058577	-0.02181	-0.27066	-0.00354	-0.18985
DCLK2	-0.11735	-0.02238	-0.01355	0.009505	-0.03449
DCLRE1A NHLRC2	0.071822	-0.50112	-0.52954	-0.01608	-0.40845
DCLRE1C	-0.09309	-0.07206	-0.07711	-0.05033	-0.16585
DCP1A	0.019907	0.054912	-0.00974	-0.07934	-0.02187
DCP1B	-0.09352	0.110219	-0.21383	-0.39326	-0.00785
DCP2	-0.18322	-0.58269	-0.55438	-0.83433	-0.43298
DCPS	0.301864	0.123818	-0.00247	-0.03586	0.204098
DCST1 ADAM15	0.034427	0.043638	0.061501	0.005032	0.143217
DCST2 DCST1	0.160438	0.422721	0.257724	0.327999	0.268286
DCT	-0.11663	0.232052	0.31173	-0.06147	0.051779
DCTD	0.068126	-0.08165	0.017401	-0.00756	0.09708
DCTN2	-0.87449	-0.61849	-1.10481	-0.75795	-0.85385
DCTN3	-0.46408	-0.61998	-0.67268	-0.58541	-1.0138
DCTN4	-0.00706	0.125321	0.227198	0.121666	0.047589
DCTN6	-0.13525	-0.32383	-0.56714	-0.28116	-0.44533
DCUN1D1	0.120359	0.292906	0.126002	0.102683	0.308488
DCUN1D2 TMCO3	-0.07768	-0.14375	-0.13403	-0.01776	0.093178
DCUN1D4	-0.27004	0.130201	-0.08223	0.014087	-0.10813
DCUN1D5	-0.15805	-0.23229	-0.27988	-0.31066	-0.36491
DCX	-0.38699	0.135745	-0.84413	-0.58496	-0.38785
DCXR	0.191023	0.247158	0.204375	0.129719	0.193316
DDA1	-0.22771	-0.11003	-0.48252	-0.25489	-0.34825
DDAH1	-0.01439	0.094558	0.047427	-0.06769	0.078335
DDAH2 CLIC1	-0.04068	0.034177	0.033173	-0.116	-0.01759
DDB1 DAK	-0.22444	-0.09565	-0.26751	-0.36889	-0.16883
DDB2	-0.01286	-0.60165	-0.57647	-0.20322	-0.10447
DDC	-0.01708	-0.11544	-0.22488	-0.10097	-0.21743
DDEF1	0.495862	0.658573	0.812004	0.401915	0.657254
DDEF2	-0.04155	0.184783	0.052962	0.004614	0.259032
DDEFL1	-0.0175	0.002228	-0.22246	-0.10947	0.022678
DDHD1	0.235884	0.398717	0.270772	0.165011	0.176568
DDHD2	-0.13423	-0.02043	0.047685	-0.07346	0.856873
DDIT3 MBD6	-0.26298	-0.26893	-0.32392	-0.10562	-0.31853
DDIT4	-0.21411	-0.30647	-0.3081	-0.32779	-0.15417
DDIT4L	-0.10997	0.523554	0.397052	0.56373	0.415199
DDN	-0.24066	-0.67558	-0.4787	-0.59767	-0.66297
DDO	0.195305	0.018341	0.294722	0.286103	0.082278
DDOST	-0.31855	-0.36243	-0.45662	-0.11044	-0.12856

DDR2	0.287752	-1.59138	-1.60433	-1.0085	-1.2823
DDT GSTT2	0.267018	-0.1873	-0.20197	0.118169	-0.06591
DDX1	0.226924	0.20433	0.261533	-0.19966	0.033493
DDX10	0.117124	0.187942	0.21616	0.159885	-0.24981
DDX11	-0.68166	-0.538	-0.92071	-0.7115	-0.74975
DDX18	-0.5009	-0.51745	-0.45337	-0.42227	-0.27936
DDX19A	0.214971	0.321935	0.123783	0.202359	0.07382
DDX19B	0.203015	0.21318	0.102198	0.133714	0.176542
DDX20	-0.34173	-0.39711	-0.24491	-0.39019	-0.226
DDX21	-0.44017	-0.4102	-0.50875	-0.29985	-0.52486
DDX23	-0.35298	-0.45398	-0.72998	-0.33119	-0.79591
DDX24 FAM14B	-0.17582	0.043419	0.054706	-0.09176	-0.07474
DDX26B	0.451359	0.268608	0.269163	0.035045	0.108319
DDX27	-0.06643	-0.16834	-0.04193	-0.09106	-0.10235
DDX28 DUS2L	0.213094	0.228619	0.380865	0.181719	0.050367
DDX31 GTF3C4	-0.03943	-0.02826	-0.00717	-0.04196	-0.05643
DDX39	-0.05245	-0.03979	-0.15812	-0.09751	-0.20157
DDX3X	-0.27007	-0.27239	-0.17865	-0.11869	-0.17871
DDX3Y	0.124739	0.256536	0.364908	0.165375	0.260451
DDX4	0.332951	0.33322	0.64871	0.270682	0.278506
DDX41	-0.13431	-0.2786	-0.30816	-0.07214	-0.2871
DDX46	0.007972	0.180247	0.089516	-0.05059	0.038286
DDX47	0.245941	0.074288	0.109854	0.048971	0.081041
DDX5 CCDC45	-1.11342	-1.33191	-1.30619	-1.11856	-0.88502
DDX50	0.048632	-0.05369	-0.07005	-0.03397	0.050156
DDX51 NOC4L	0.089293	0.053616	0.125224	0.147002	0.151085
DDX52	-0.1292	-0.54882	-0.57866	-0.58899	-0.52925
DDX53	0.436628	0.64701	0.543317	0.279546	0.335194
DDX54 C12orf52	-0.11049	-0.07396	-0.15224	-0.01568	-0.15348
DDX55	0.148571	0.060264	-0.00718	0.117826	0.230094
DDX56	0.183541	0.06462	0.296775	0.074294	0.099533
DDX58	-0.23253	0.075335	0.09511	0.175272	0.014847
DDX59	-0.52478	-0.53423	-0.53035	-0.4341	-0.43516
DDX6	-0.40258	-0.36078	-0.50811	-0.44376	-0.59615
DDX60	-0.35593	-0.55537	-0.73283	0.39361	-0.52991
DEAF1 TMEM80	-0.0542	0.074211	0.00872	-0.07372	-0.00578
DECR1	-0.4109	-0.46962	0.266618	-0.51102	-0.34057
DECR2	-0.01534	-0.06229	0.240782	0.069608	0.085937
DEDD2 ZNF526	0.11285	0.319963	0.250517	0.121586	0.201656
DEF6	0.285178	0.369385	0.484659	0.166886	0.427386
DEF8	0.105165	0.180721	0.107654	0.132273	0.191613
DEFA3	-0.2172	-1.50741	-1.68477	-1.23212	-0.76123
DEFA4	0.139951	-0.45512	-0.58691	-0.00076	0.24415
DEFA5	-0.49278	-1.33641	-1.34843	-1.04038	-1.15921
DEFA6	-0.12018	-0.63076	-0.4722	-0.46072	-0.66748
DEFB1	0.073226	-0.38376	-0.372	-0.05306	-0.52488
DEFB103A	-0.07215	-0.9118	-1.30192	-0.70129	-0.60509
DEFB104B	0.270742	-1.82062	-1.79192	-0.86828	-1.49142
DEFB106B DEFB105B	-0.42857	-1.54495	-1.37322	-1.02233	-0.86409
DEFB107B	-0.22562	-0.59321	-0.66144	-0.66452	-0.36234

DEFB108B	-0.07776	-1.02136	-0.88851	-1.35393	-1.13064
DEFB110	-0.2742	-0.36445	-0.2602	-0.32061	-0.43224
DEFB112	-0.33012	-0.38923	-0.29749	-0.36129	-0.56889
DEFB113	-0.95005	-1.14962	-0.88987	-0.83636	-0.97666
DEFB114	-0.41965	-0.46481	-0.37515	-0.42668	-0.40936
DEFB115	-1.47142	-1.42929	-1.49329	-1.04948	-1.19928
DEFB116	-0.42274	-1.01622	-0.75408	-0.77666	-0.82885
DEFB118	-0.64019	-2.01529	-2.00108	-1.6449	-1.87926
DEFB119	-0.54011	-0.7253	-0.56453	-0.54934	-0.54368
DEFB121	-0.68941	-1.01859	-0.94914	-0.69428	-1.10599
DEFB123	0.448198	-0.44011	-0.22683	-0.05922	0.177811
DEFB124 REM1	-0.13828	-0.61209	-0.32352	-0.07639	-0.5538
DEFB125	-0.10291	-0.98914	-0.76808	-0.51391	-0.07203
DEFB126	-0.92702	-1.04886	-0.90493	-0.65263	-0.51589
DEFB127	0.134194	-0.65415	-1.19308	-0.49358	-0.0947
DEFB128	0.069869	-1.45884	-1.23128	-1.26937	-1.11886
DEFB129	-0.12155	-0.1425	-0.02963	-0.14981	0.17844
DEFB132	0.011174	-0.60849	-0.54793	-0.16848	-0.49968
DEFB4	-0.08357	-1.11514	-0.97214	-0.82436	-0.70733
DEGS1	0.34053	0.469351	0.315418	0.140633	0.356777
DEGS2	0.092909	0.221367	0.249375	0.320694	0.266006
DEK	-0.18484	-0.30639	-0.37451	-0.32746	-0.19152
DENND1A	-0.00745	0.199339	0.257121	0.189301	0.288281
DENND1C	0.459371	-0.09424	0.628735	0.431432	0.644704
DENND2A	0.105592	0.235463	0.298444	0.079517	0.189062
DENND2C	-0.13119	-0.18544	0.346099	-0.14033	0.115971
DENND2D	0.141759	-0.00901	0.074276	-0.24347	-0.03385
DENND3	0.109439	0.082831	0.020667	0.088595	0.207635
DENND4A	-0.18945	-0.09621	-0.19532	-0.12014	-0.04698
DEPDC1	-0.43229	-0.31134	-0.31241	-0.49204	-0.13606
DEPDC1B	0.010523	-0.17826	-0.17284	-0.09884	-0.17049
DEPDC2	-0.37411	-0.07955	0.157235	-0.08763	-0.19844
DEPDC4 SCYL2	-0.33379	-0.30198	-0.32887	-0.40505	-0.35209
DEPDC5	-0.11318	-0.23287	-0.45852	-0.01904	-0.4545
DEPDC6	0.318753	0.081623	0.175734	-0.07905	0.151288
DEPDC7	0.204538	0.366383	0.150763	0.195619	0.295632
DERL1	0.04745	-0.16186	-0.20562	-0.26872	-0.10444
DERL2 MIS12	-0.29964	-0.09329	-0.44822	-0.19982	-0.13717
DERL3	-0.05349	0.610884	0.456531	0.43644	0.501039
DES	0.240176	0.349084	0.355431	0.241868	0.490349
DET1	-0.10537	0.110771	0.014834	-0.17449	-0.10773
DEXI CLEC16A	0.131317	0.238018	0.281952	0.018069	0.11207
DFFA PEX14	0.121497	0.197761	0.203486	0.171895	0.265366
DFNA5	0.243706	0.374244	0.143734	0.316316	0.300646
DFNB31	0.121126	0.109034	0.356061	0.174268	0.147752
DGAT1	0.116323	0.126231	0.060745	0.153152	0.071065
DGAT2	0.112848	0.211722	0.190226	0.046013	0.023632
DGAT2L3	-0.34212	0.063012	-0.41144	-0.19151	-0.18185
DGAT2L4	-0.55163	-0.27355	-1.45123	-0.55661	-0.74851
DGAT2L6	-0.25801	0.300812	-0.31392	-0.11821	0.054396

DGCR2	-0.05025	0.085787	-0.15455	-0.06415	-0.07281
DGCR6	-0.00893	-0.20472	-0.23722	-0.19374	-0.26848
DGCR6L	-0.02484	-0.00449	0.089196	0.057813	0.088664
DGCR8	0.357343	0.092564	0.137406	0.188838	0.226496
DGKA	0.073707	-0.1982	-0.38899	-0.30368	-0.1414
DGKB	-0.06772	-0.17366	-0.47185	-0.3259	-0.15191
DGKG	0.183672	0.088857	0.25827	0.177112	0.194565
DGKH	-0.32565	-0.26795	-0.21748	-0.24072	-0.2694
DGKI	0.083021	0.022105	-0.01998	0.029925	0.211876
DGKK	0.061851	-0.24181	-0.06964	0.249685	-0.09091
DGKQ	-0.04432	0.135046	0.174982	0.061053	0.15984
DGKZ MDK	-0.0272	-0.21805	-0.16954	-0.12279	-0.03459
DGUOK	0.226658	0.36561	0.091374	0.077482	0.173103
DHCR24	0.047288	0.163496	-0.06853	0.063512	0.238539
DHCR7	-0.18022	0.010125	-0.05484	-0.11918	-0.07074
DHDDS	-0.18584	0.280096	0.298277	-0.09273	0.062689
DHFRL1 NSUN3	-0.34281	-0.43203	-0.72832	-0.48646	-0.72495
DHH	-0.11851	-0.3865	-0.36936	0.198369	-0.31765
DHODH	-0.05795	0.45346	0.272968	0.187993	0.162429
DHPS	-0.20615	-0.0141	-0.67718	-0.23412	-0.24377
DHRS1 C14orf21	0.017888	-0.21363	-0.11059	-0.02057	-0.067
DHRS12	0.404745	-0.03355	-0.09262	-0.06249	0.418243
DHRS13	0.097302	0.371847	0.095872	0.126328	0.166979
DHRS2	0.131792	0.771008	0.126086	0.13992	-0.03198
DHRS3	-0.49509	0.379077	0.521418	0.276271	0.416123
DHRS4L2	0.116738	0.353913	0.246385	0.157324	0.255507
DHRS7B	0.030742	0.03805	-0.10486	-0.11519	-0.1627
DHRS8	-0.06767	-0.51797	-0.80563	-0.22045	-0.6325
DHRSX	-0.20817	-0.28531	-0.27248	-0.23326	-0.17194
DHTKD1	0.096368	0.150545	0.331157	0.228225	0.114163
DHX15	0.070281	0.100994	0.074952	0.075111	0.238092
DHX16	0.29634	0.088783	0.167422	0.116475	0.045
DHX29 SKIV2L2	-0.58913	-0.70242	-0.72665	-0.65424	-0.69647
DHX32	0.013951	0.753715	0.3572	0.197325	0.251594
DHX33	0.100264	0.156313	0.119084	0.101836	0.186173
DHX34	-0.11445	-0.02041	-0.55721	-0.40153	-0.29081
DHX35	-0.25867	-0.27512	0.215338	-0.32644	-0.17937
DHX36	0.044496	-0.30525	-0.53244	-0.11531	-0.34955
DHX37	0.173544	0.223173	0.304771	0.11138	0.224142
DHX40	0.275531	0.429567	-0.15694	-0.47581	0.023568
DHX57 MORN2	0.167854	-0.2328	-0.51367	-0.34275	-0.47918
DHX58 GCN5L2	0.090234	0.129021	0.361702	0.165329	0.175905
DHX8	-0.02902	-0.11334	-0.2121	-0.40007	-0.21606
DHX9	0.136407	0.183198	0.124725	0.002557	0.076919
DIABLO	0.054034	-0.22837	-0.12773	-0.066	0.070536
DIAPH1	-0.28025	-0.14894	-0.25784	-0.25006	-0.3721
DIAPH3	0.595052	0.669619	0.822963	0.332772	0.511667
DIDO1	-0.07202	-0.04087	-0.04177	0.156644	-0.06189
DIDO1 C20orf11	-0.09379	-0.14961	-0.21743	-0.07423	-0.07706
DIMT1L	-0.05337	-0.11141	-0.43706	-0.44002	-0.3364

DIO1	0.314242	0.404264	0.628137	0.329915	0.346502
DIO2.	0.041318	0.145916	-0.02744	-0.08858	-0.53895
DIO3	0.250398	0.390313	0.593703	0.276209	0.391824
DIP2A	-0.11745	-0.03837	-0.03922	-0.02296	-0.01328
DIP2B	-0.08444	0.020655	-0.02699	0.006384	-0.13627
DIRAS1	-0.26666	-0.32578	-0.06372	-0.21613	-0.20205
DIRAS2	-0.00026	0.209373	0.082229	-0.03816	-0.28938
DIRAS3	0.497224	0.59822	0.59836	0.649173	-0.37735
DIRC1	-0.53519	-0.21525	-0.41149	-0.33646	-0.22881
DIS3 PIBF1	-0.65587	-0.86886	-0.73577	-0.54848	-0.63694
DIS3L	0.332067	0.388957	0.285942	0.326929	0.235605
DIS3L2	0.351365	0.30965	0.367842	0.253101	0.281604
DISC1	-0.18602	-0.20315	-0.34513	-0.15067	-0.07674
DISP1	-0.17203	-0.19844	-0.42256	-0.3883	-0.25442
DISP2	0.106223	0.120743	0.052682	0.053442	0.061363
DKC1	0.341612	-0.06232	0.296899	-0.01126	0.07602
DKK1	-0.44613	-0.599	-0.63771	-0.23841	-0.14334
DKK2	-0.61018	-1.05702	-0.81049	-0.55015	-0.68694
DKK3	-0.05974	0.222428	0.011646	0.068962	0.085995
DKK4	0.325155	0.019513	0.412787	0.294915	-0.47823
DLAT	-0.03539	-0.09549	-0.59132	-0.14888	-0.56938
DLD	-0.39209	-0.49785	-1.06675	-0.48839	-0.70736
DLEC1	0.271552	0.599976	0.602735	-0.10071	0.088254
DLEU7	0.370346	0.534094	0.568985	0.315136	0.468833
DLG1	-0.08593	0.219306	-0.28033	0.005326	-0.1771
DLG2	0.286295	0.446892	-0.93694	0.265197	-0.86639
DLG3	-0.11707	-0.24675	-0.30589	-0.09161	-0.19991
DLG4 ACADVL	-0.08727	-0.07653	-0.28695	-0.12098	-0.0879
DLG7	0.14984	-0.18029	-0.24216	-0.25019	-0.63505
DLGAP2	0.411177	0.813464	0.853358	0.476173	0.337283
DLGAP4	-0.04214	-0.02277	-0.03729	0.032216	0.120026
DLK1	0.534057	0.504855	0.575483	0.51214	0.648972
DLL1	-0.30143	-0.09821	-0.01256	-0.0101	-0.04909
DLL3	-0.1578	-0.46749	-0.80316	-0.62174	-0.56189
DLL4	-0.35825	-0.27768	-0.34158	-0.08898	-0.26639
DLSTP.	-0.17061	0.155512	-0.22143	-0.10156	-0.25667
DLX1	-0.2123	-0.02879	-0.03555	0.059721	0.120532
DLX2	-0.50293	-0.66491	-0.73564	0.103757	-0.27232
DLX3	-0.30172	-0.50514	0.183222	-0.4217	0.048501
DLX5	-0.68725	0.127035	0.403966	-0.19523	0.24826
DLX6	-0.30036	-0.23645	-0.41759	-0.24701	-0.19854
DMAP1	-0.08751	-0.20044	-0.51658	-0.13186	-0.11437
DMBT1	-0.0164	-1.32106	-0.83773	-0.45773	-1.36433
DMBX1	0.255052	0.41993	0.604752	0.288789	0.246539
DMC1	-0.23401	0.03163	-0.10916	-0.01571	0.095324
DMGDH BHMT2	0.392348	0.234943	0.664023	0.281281	0.500929
DMN	0.064196	0.11563	0.009627	0.122795	0.084946
DMP1	0.491689	0.370591	0.620393	0.099799	0.133754
DMPK DMWD	0.063733	-0.09442	-0.0753	0.224143	-0.15132
DMRT1	-0.00167	-0.0718	0.127462	0.245392	0.151304

DMRT2	-0.03802	0.166054	0.188146	0.324762	0.411795
DMRT3	-0.05116	0.098258	0.344951	0.121621	0.258857
DMRTA1	0.070695	-0.07667	-0.11963	-0.18947	0.015909
DMRTB1	0.241192	0.448953	0.580736	0.308052	0.416994
DMRTC1B	0.616248	0.487545	0.191351	0.356427	0.645915
DMTF1	-0.39145	-0.43006	-0.57044	-0.39545	-0.19435
DMWD	-0.12976	-0.30078	-0.14344	-0.54967	-0.58117
DMXL1	-0.01006	0.038889	0.039888	-0.03773	-0.0372
DMXL2	0.040607	-0.00544	0.078295	-0.00183	-0.04351
DNAH1	0.106963	0.071716	-0.06749	-0.06188	-0.07447
DNAH11	0.357162	-0.00026	0.530606	0.449372	0.538728
DNAH12L	-0.07572	0.154478	-0.02459	-0.10474	-0.15643
DNAH2	0.481673	0.418258	0.715752	0.542301	0.499125
DNAH3 TMEM159	-0.08576	0.18981	0.238265	0.057322	0.195061
DNAH5	0.393824	0.0383	0.072042	0.198101	0.057404
DNAH7	0.309063	-0.02233	-0.36119	0.232372	0.134823
DNAH8	0.603661	0.867729	0.909771	0.454199	0.636139
DNAJA1	0.121965	-0.13658	0.154288	0.047184	0.07016
DNAJA2	-0.0168	-0.0152	-0.27461	-0.16656	-0.14751
DNAJA3	-0.2436	-0.3452	-0.57995	-0.44106	-0.44907
DNAJA4	0.284669	0.3665	0.470267	0.197123	0.422444
DNAJA5	-0.20254	-0.10297	-0.21773	-0.30852	-0.31958
DNAJB1	0.083654	0.2666	0.247274	0.162624	0.223124
DNAJB11	-0.44864	-0.3636	-0.43551	-0.38286	-0.50626
DNAJB12	0.137172	0.076623	0.196014	-0.04888	-0.10778
DNAJB2	-0.10644	-0.0055	-0.10891	-0.21203	0.087843
DNAJB4	-0.6789	-0.65647	-0.92271	-1.02245	-0.29282
DNAJB5	-0.29143	-0.34069	-0.27546	-0.28076	-0.33578
DNAJB6	-0.01156	0.044172	0.308218	0.233823	0.114996
DNAJB8	0.150974	0.201179	0.533919	0.195666	0.284695
DNAJC1	-0.05412	0.101583	0.070021	-0.07352	0.049468
DNAJC10	-0.11174	-0.17958	-0.05347	-0.25117	-0.18159
DNAJC11	0.007846	0.123085	0.217277	0.086707	0.229548
DNAJC12	0.498566	0.299136	0.414031	0.122113	0.026797
DNAJC13	-0.25442	-0.29546	-0.33702	-0.21678	-0.31403
DNAJC14	-0.3121	-0.36272	-0.29156	-0.35058	-0.27833
DNAJC15	0.604435	0.664387	0.664645	0.724742	0.642334
DNAJC17 ZFYVE19	0.263208	0.054955	-0.07962	0.169402	0.172087
DNAJC18	-0.11617	0.129275	-0.12853	-0.17363	-0.30421
DNAJC19	-0.55855	-0.60303	-0.79409	-0.60445	-0.8544
DNAJC3	-0.45763	-0.26725	-0.28409	-0.26085	-0.28694
DNAJC4 VEGFB	-0.03971	0.008282	-0.00374	-0.02977	0.01598
DNAJC5	-0.17624	0.246353	-0.14513	0.273808	-0.05481
DNAJC5B	0.131064	-0.25495	-0.12836	-0.34567	0.071472
DNAJC5G	0.601449	0.760285	0.836955	0.43749	0.609525
DNAJC7 NKIRAS2	-0.08866	-0.23393	-0.33399	-0.22083	-0.31673
DNAJC8	0.136727	0.373696	0.232516	0.091464	0.308573
DNAJC9	-0.18685	-0.24662	-0.32675	-0.23393	-0.19802
DNAL1	-0.25881	-0.60484	-0.82993	-0.48568	-0.84791
DNASE1	0.542107	0.599394	0.620281	0.402207	0.459518

DNASE1L1 TAZ	0.254264	0.201359	0.19897	0.175965	0.274365
DNASE1L2	0.163299	0.382858	0.148804	0.302262	0.410563
DNASE1L3	0.200115	0.068386	0.167871	-0.31702	-0.19485
DNASE2	0.366029	0.260696	0.151239	0.204477	0.305649
DND1 HARS	0.467706	0.636195	0.587671	0.485031	0.572578
DNER	-0.02779	0.082233	0.060935	0.061405	0.157021
DNHD1	-0.21314	-0.02728	-0.79975	-0.62005	-0.34375
DNHL1	0.44366	0.067191	-0.02369	-0.01214	0.004021
DNM1L	0.080456	0.129457	0.139252	0.086701	0.117007
DNM2	-0.0036	0.108093	0.103911	0.142363	0.118206
DNM3	-0.00029	0.135928	0.021695	-0.04372	-0.05893
DNMBP	0.276597	0.333871	0.397626	-0.05279	0.421977
DNMT1	-0.38568	-0.24759	-0.29823	-0.32971	-0.2345
DNMT3L	-0.58577	-3.60306	-0.4579	-0.35518	-0.48781
DNPEP	-0.34719	-0.27298	-0.39274	-0.2176	-0.19618
DNTT	0.205203	0.029717	0.015303	-0.1922	-0.23356
DNTTIP2	0.056398	0.242991	0.004158	-0.03339	0.055995
DOC2A	-0.16283	0.011659	0.032	-0.04455	-0.01483
DOCK1	-0.13001	0.137546	0.133426	0.020324	0.231974
DOCK10	0.190711	0.219486	0.002512	-0.08631	-0.70644
DOCK11	-0.01534	0.057818	0.045912	-0.06543	-0.03725
DOCK2	0.421651	0.676761	0.606892	0.330684	0.5014
DOCK3	0.05613	0.066793	-0.0279	0.179929	0.111465
DOCK8	0.700982	0.708678	0.67991	0.501781	0.563533
DOCK9	0.015683	0.154562	0.195192	0.12385	0.239333
DOHH RAX2	0.105296	0.298105	0.078715	0.14414	0.162703
DOK2	0.536946	0.523552	0.85026	0.186936	0.339775
DOK3	0.224362	0.30464	0.521819	0.347688	0.341318
DOK4	-0.16996	-0.47798	-0.61051	-0.34008	-0.76177
DOK5	-0.36519	-0.14534	0.152688	0.170359	0.287269
DOK6	-0.06513	0.034161	0.050754	-0.04616	0.089478
DOK7	0.361408	0.152877	0.288287	0.17218	0.237145
DOLK NUP188	-0.07269	-0.03002	-0.1254	0.076271	-0.17043
DOLPP1	-0.20077	-0.19192	-0.14124	-0.28967	-0.29996
DOM3Z STK19	0.007413	-0.19738	-0.24514	-0.02308	-0.26108
DONSON CRYZL1	0.04104	0.001471	-0.02108	0.064818	0.065202
DOPEY2	0.298133	0.344012	0.285341	0.067987	-0.16657
DOT1L	0.074855	0.084727	0.150473	0.007644	0.024083
DPAGT1	-0.44635	-0.33144	-0.64939	-0.37554	-0.57159
DPCR1	-0.65749	-3.58404	-3.67462	-3.11303	-3.07947
DPEP1	0.430595	0.270445	0.766731	0.222193	0.59585
DPEP3	0.273466	0.329986	0.58541	0.368048	0.362856
DPF1	0.170837	-0.02018	-0.13324	0.091997	0.03273
DPF2	-0.1691	-0.23657	-0.32987	-0.11603	0.011912
DPF3	-0.9311	-0.80194	-0.9968	-0.60387	-0.93596
DPH2	0.017761	0.162006	-0.08533	0.118634	0.026006
DPH5	-0.52237	-0.39433	-0.52624	-0.53835	-0.50611
DPM1 MOCS3	-0.15024	-0.1665	0.072158	-0.02008	-0.03394
DPM2	0.12665	0.271851	0.198156	0.25235	0.232863
DPM3	-0.00396	-0.06477	-0.43551	-0.32995	-0.11801

DPP10	-0.24974	-0.03791	-0.18471	0.351414	0.031544
DPP4	-0.12556	0.207343	0.086797	0.152573	0.228407
DPP7	0.146741	0.009055	0.072399	-0.00571	0.141825
DPP8	-0.03519	-0.00085	-0.01803	-0.0344	-0.06987
DPP9	-0.36214	-0.54266	-0.42028	-0.34183	-0.19175
DPPA2	0.288486	0.307821	0.55375	0.236864	0.288283
DPPA3	0.688996	-0.42936	-0.36502	-0.16116	0.121164
DPPA4	0.816019	1.032951	1.162133	0.696423	0.412946
DPPA5	0.425503	0.650389	0.67899	0.4706	0.708575
DPRX	0.619584	-0.43878	0.590974	0.383503	0.500983
DPT	0.344694	0.462249	0.154135	0.294002	0.301569
DPY19L2	-0.25375	0.391258	0.382789	0.214739	0.267122
DPY19L2P2.	-0.24901	0.185391	0.303826	0.264624	0.151401
DPY19L3	0.137284	0.091391	0.174364	0.17677	0.082695
DPY19L4	-0.29706	-0.19975	-0.34483	-0.24596	-0.33448
DPY30	-0.30522	-0.16469	-0.47304	-0.32979	-0.39306
DPYD	-0.27365	-0.42739	-0.30258	-0.38238	-0.34266
DPYS	0.27254	0.438967	0.555804	0.199681	0.211871
DPYSL2	-0.30805	-0.22573	-0.13919	-0.24257	-0.27528
DPYSL3	-0.3992	-0.3733	-0.41748	-0.07766	-0.08524
DPYSL4	-0.0751	-0.12035	-0.01014	0.015811	0.08973
DPYSL5	0.016599	0.054644	0.005479	0.001058	-0.14009
DQX1 AUP1	0.314977	0.82014	0.607409	0.499663	0.501883
DR1	-0.40801	-0.4343	-0.57091	-0.43983	-0.17692
DRD1	-0.11556	-0.01462	0.512813	0.174516	0.400843
DRD1IP	0.045153	0.060098	0.008549	-0.11797	-0.10547
DRD2	-0.00729	0.122827	0.130216	0.112683	0.195111
DRD3	-0.81245	0.209689	0.124996	-0.19376	-0.47802
DRD5	0.244394	0.481346	0.454902	0.454135	0.556992
DRG1	0.192944	0.321156	0.219992	0.031214	0.113362
DRG2	0.079998	0.25437	-0.00151	0.069346	0.17722
DRP2	-0.21501	0.418946	-0.43456	-0.13074	0.278933
DSC1	0.141097	-0.9415	-0.78011	-0.66572	-0.49764
DSC2	0.338091	-0.2038	0.41648	0.19858	0.239326
DSC3	0.008357	0.181035	0.532024	0.381933	0.375251
DSCAM	0.268549	0.642669	0.664125	0.244537	0.316006
DSCAML1	0.327332	0.451365	0.41332	0.250656	0.257099
DSCC1	0.099458	0.129189	0.312181	0.193727	0.331384
DSCR1L1	0.421225	-0.10259	-1.08134	-0.73338	-0.32662
DSCR2	-0.33695	-0.17918	-0.3419	-0.32836	-0.26977
DSCR3	-0.13779	0.074739	-0.14607	-0.0408	-0.12969
DSCR4 DSCR8	0.610777	0.62782	0.599685	0.383163	0.065953
DSCR6	-0.02187	0.340778	0.258048	0.279255	0.273714
DSE	-0.05819	0.403455	-0.07561	-0.0242	0.003149
DSG1	-0.06787	-0.94435	-0.83441	-0.59667	-0.27758
DSG2	0.156278	0.061487	0.2801	0.190752	0.193267
DSG3	0.677314	-0.3043	-0.52709	-0.48288	-0.11605
DSG4	0.261286	-1.31282	-0.91599	-0.87224	-0.38103
DSP	0.027735	0.413968	0.604995	0.436781	0.469433
DSPP	-0.08057	0.056289	0.046547	-0.11363	-0.23917

DSTN	-0.15658	-0.17571	-0.24668	-0.15398	-0.12206
DTNB	0.10186	0.143773	0.30131	0.132148	0.277224
DTNBP1	0.16394	0.337395	0.417143	0.047331	0.219753
DTWD2	0.450863	0.091919	-0.05093	0.22551	-0.13479
DTX1	0.201465	0.48115	0.452692	0.299538	0.37457
DTX2	-0.15758	-0.24304	-0.23573	-0.12585	-0.2094
DTX3	-0.5429	-0.35251	-1.06763	-0.51663	-0.82711
DTYMK	0.164737	-0.02295	0.08126	0.041521	0.049404
DULLARD C17orf81	-0.29653	-0.10291	-0.40512	-0.1758	-0.19649
DUOX2 DUOXA2	-0.36733	0.091317	0.634061	0.390794	0.294644
DUOXA1 DUOX1	-0.05659	0.368682	0.437462	0.338708	0.404145
DUPD1	0.402442	0.485125	0.409741	0.363961	0.545464
DUS1L	0.148996	0.148373	0.353287	0.264116	0.229513
DUSP	-0.25091	-0.24557	-0.1813	-0.1069	-0.09202
DUSP1	0.051815	0.024074	0.03189	-0.03266	0.007976
DUSP11	-0.30842	-0.29038	-0.55006	-0.4329	-0.42445
DUSP12	-0.13932	-0.09381	-0.22311	-0.34992	-0.14085
DUSP14	-0.03931	-0.13015	-0.06116	0.030417	0.016195
DUSP15	-0.11104	-0.32188	-0.48787	-0.32351	-0.34445
DUSP16	-0.3125	-0.29322	-0.41168	-0.29123	-0.34206
DUSP18	0.030393	0.258556	-0.13609	0.136767	-0.10568
DUSP19	-0.36454	-0.79557	-1.40955	-0.90785	-0.74678
DUSP2	0.077376	0.166187	0.190695	0.158799	0.188222
DUSP21	0.498814	0.68253	0.768595	0.432525	0.654879
DUSP22	0.177085	-0.09383	0.041557	0.080434	0.140695
DUSP23	-0.0776	0.50654	-0.38562	0.359014	0.508551
DUSP26	0.133818	0.173185	-0.09736	-0.08317	-0.02219
DUSP3	0.00485	0.098064	-0.05584	-0.10406	-0.00427
DUSP4	-0.34809	-0.41372	-0.47435	-0.27952	-0.49576
DUSP6	-1.1502	-1.21568	-0.8791	-0.68772	-0.91333
DUSP7	-0.00329	0.052509	0.113224	0.015701	0.153031
DUSP8	-0.13354	-0.05061	-0.04053	0.086228	-0.02245
DUSP9	0.019462	-0.16615	0.171372	0.07185	0.197719
DUT	-0.33133	-0.36731	-0.18761	-0.22915	-0.24595
DUXA	0.293842	-0.17361	-0.27029	-0.08194	-0.06383
DVL1	0.044899	0.053964	0.143168	0.184782	0.245223
DVL2 PHF23	-0.16271	-0.28231	-0.35737	0.010616	-0.15521
DVL3	-0.0411	0.065685	0.027161	-0.07134	0.071264
DYDC1 DYDC2	-0.08309	0.201497	-0.02754	-0.35339	-0.40241
DYM	0.096693	0.249854	0.249039	0.23174	0.294558
DYNC1H1	0.306625	0.183705	0.40407	0.293623	0.118217
DYNC1I1	-0.11287	0.235005	-0.01145	0.025906	0.007014
DYNC1I2	-0.27235	-0.22446	-0.454	-0.42525	-0.16541
DYNC1LI1	-0.29488	0.297806	-0.67347	-0.16815	0.063193
DYNC1LI2	0.194578	0.226481	0.343383	0.050788	0.164142
DYNC2LI1	-0.02933	0.120583	0.150588	-0.08395	-0.07474
DYNLL1	-0.30172	-0.24724	-0.42968	-0.12894	-0.0903
DYNLL2	-0.11954	-0.16931	-0.02401	-0.12268	-0.11689
DYNLRB2	0.144423	-0.0879	0.034655	0.073961	-0.15401
DYNLT1	0.243521	0.156443	0.177154	0.038499	0.16547

DYNLT3	0.220305	0.062338	0.04665	-0.0385	-0.01328
DYRK1B FBL	0.288561	0.345802	0.308729	0.27076	0.265005
DYRK2	-0.29051	-0.21336	-0.23779	-0.23678	-0.26395
DYRK3	0.216788	0.449736	0.489114	0.290275	0.284058
DYRK3 MAPKAPK2	-0.1079	-0.4705	-0.27312	-0.4275	-0.12497
DYSF	-0.19439	0.186352	0.291617	-0.0311	0.210576
DYSFIP1	0.54382	0.743188	0.566769	0.706695	0.649239
DYX1C1	0.242929	0.55794	0.400206	0.100721	0.071769
DZIP1	0.197528	0.210613	0.41095	0.073551	-0.04395
DZIP1L	-0.20153	-0.12661	-0.37226	-0.32507	-0.19506
E2F1	0.150288	0.215458	0.228265	0.143263	-0.01947
E2F2	0.061345	-0.02967	-0.10045	0.056657	0.112609
E2F3	-0.67663	-0.66815	-0.73237	-0.45106	-0.64612
E2F4 ELMO3	0.279271	0.499689	0.384606	0.321959	0.533235
E2F5	0.036017	0.280295	0.093733	0.108901	0.157443
E2F6	0.340798	0.077953	0.149181	0.054394	0.06085
E2F7	-0.26672	-0.27741	-0.24032	-0.26378	-0.3321
E2F8	-0.23381	-0.35922	-0.48534	-0.2627	-0.1403
E4F1	0.078635	0.047856	-0.01116	0.017236	-0.07062
EAPP	0.362452	0.263564	0.061179	0.104995	0.16525
EARS2 UBFD1	0.312079	0.098357	0.334548	0.116766	0.33216
EBAG9	-0.39978	-0.44951	-0.4846	-0.68389	-0.53802
EBF1	-0.32202	-0.35123	-0.33594	-0.39586	-0.3154
EBF3	-0.08461	0.203311	0.208942	0.136667	0.081788
EBI3	0.029335	-0.47455	-0.1173	-0.02808	-0.10991
EBNA1BP2 WDR65	-0.30058	-0.52058	-0.79107	-0.52745	-0.35081
EBP	-0.06439	-0.47419	-0.14064	-0.58813	-0.76198
EBPL	0.111767	0.142642	0.122229	0.098874	0.026307
ECD FAM149B1	-0.04904	0.014434	-0.01789	-0.01008	0.004027
ECE1	-0.03385	0.127261	0.085297	0.081496	0.008311
ECEL1	-0.1813	0.17178	0.35394	0.226783	0.264927
ECGF1 U62317.2	0.154596	0.131035	0.389008	0.240238	0.22317
ECH1	0.281076	0.253132	0.201156	0.024416	-0.04296
ECHDC1	-0.42247	-0.26897	-0.45197	-0.51407	-0.71156
ECHDC2	0.290438	0.575451	0.786977	0.463866	0.442647
ECHDC3	0.310237	0.301083	0.306561	0.309881	0.451306
ECHS1	0.152062	0.229634	0.268176	0.1774	0.310286
ECM1	-0.01508	0.082841	-0.00241	-0.0804	-0.06452
ECSIT	0.13248	-0.27979	-0.14239	-0.16534	-0.26235
ECT2	-0.1214	0.134461	-0.08752	-0.0303	-0.07877
EDA	0.087511	0.014039	-0.05666	-0.08748	-0.17219
EDA2R	-0.23946	-0.24942	-0.21781	-0.02532	-0.17848
EDAR	0.189948	-0.29014	0.505787	0.109219	0.107558
EDARADD	0.411233	0.451053	0.563552	0.254329	0.044251
EDC4	-0.04337	-0.00021	-0.17331	0.170152	-0.08312
EDC4 NRN1L	0.366681	0.373987	0.420914	0.327985	0.273481
EDEM1	-0.08047	0.11018	0.236345	-0.00829	-0.08595
EDEM2	-0.20478	-0.10597	-0.29928	-0.19003	-0.34832
EDEM3	-0.15553	-0.30607	-0.25169	-0.19833	-0.15332
EDF1	0.225592	0.130594	0.039887	0.093963	0.103434

EDG1	-0.79434	-0.41038	-0.35443	-0.41462	-0.52375
EDG2	-0.15248	0.168002	0.227092	-0.02235	0.136317
EDG7	0.447385	0.460322	0.474766	0.255198	0.591061
EDIL3	-0.36414	-0.25522	-0.28845	0.012385	-0.31342
EDN1	-0.9003	-0.76869	-0.91263	-0.38355	-0.85205
EDN2	0.339991	0.350031	0.47659	0.247517	0.214289
EDN3	0.100968	0.138201	0.401881	0.359467	0.335877
EDNRA	0.200601	-0.04347	0.462096	0.382876	0.474647
EDNRB	-0.23314	-0.02522	-0.08736	0.160443	0.00913
EEA1	0.039029	-0.0212	0.058694	-0.05079	-0.03846
EED	0.036384	-0.2347	-0.49957	-0.4918	-0.46305
EEF1A1	-0.67311	-0.58555	-0.49901	-0.54447	-0.56453
EEF1A2	-0.03914	0.178283	0.031847	0.019561	0.146724
EEF1D TIGD5	0.122901	0.112059	0.166087	0.093997	0.083592
EEF1E1	-0.18016	-0.33911	-0.4038	-0.36589	-0.60189
EEF1G	-0.50565	-0.56438	-0.52062	-0.35648	-0.34181
EEF2	-0.39645	-0.57385	-0.709	-0.6437	-0.63121
EEF2K	-0.02091	-0.11636	0.018542	0.035011	0.035353
EEFSEC	0.195783	0.140197	0.120136	-4.2E-05	0.073365
EEPD1	0.07475	0.138245	-0.24863	-0.08204	-0.08439
EFCAB1	0.522758	0.753708	0.82314	0.677649	0.635404
EFCAB2	-0.37013	-0.21517	-0.36455	-0.36936	-0.2001
EFCAB3	0.329374	0.479073	0.481372	0.141739	0.015417
EFCAB4A	0.105007	0.168415	0.327517	0.183006	0.077894
EFCAB4B	0.244389	0.384044	0.360696	0.185437	0.164009
EFCAB5	0.148522	0.133295	0.151089	0.011953	0.131648
EFCAB6	-0.21439	-0.13749	-0.05585	-0.14798	-0.23009
EFCBP2	-0.06412	-0.10127	0.085388	-0.18304	-0.13341
EFEMP1	-0.31346	0.371606	0.167578	0.182582	-0.01847
EFEMP2	-0.15507	-0.29337	-0.43765	-0.14103	-0.13593
EFHA1	0.035152	0.144808	0.117163	0.054108	-0.10695
EFHA2	0.102293	0.122129	-0.04054	0.087794	0.057777
EFHB	0.460371	0.801545	0.740837	0.350255	0.402671
EFHB RAB5A	-0.19433	0.18993	0.076714	0.07646	0.154927
EFHC1	-0.64166	-0.21521	-0.37264	-0.30023	-0.32451
EFHC2	0.30698	0.450095	0.144696	0.240755	0.330624
EFHD1	-0.11811	-0.05056	0.088263	0.05817	-0.08922
EFHD2	-0.05078	-0.14081	-0.25088	-0.01242	-0.00896
EFNA1	-0.44879	-0.43506	-0.44001	-0.4246	-0.16097
EFNA2	0.112653	0.047973	0.089593	0.045248	0.139284
EFNA3	-0.17005	-0.2499	-0.45333	-0.33805	-0.26746
EFNA4	-0.14988	-0.58425	0.101249	-0.07023	-0.66051
EFNA5	-0.35221	-0.42944	-0.32307	-0.27932	-0.32376
EFNB1	0.035462	0.015713	0.121327	0.040635	0.095324
EFNB2	0.547471	-0.04961	0.010425	0.108596	-0.01761
EFNB3	-0.12934	-0.40159	-0.89266	-0.24741	-0.43788
EFR3A	0.359153	0.219457	0.39294	0.029542	0.194863
EFS	0.073816	-0.02264	0.001066	0.159532	0.143192
EFTUD1 FAM154B	-0.42213	-0.15093	-0.65905	-0.28494	-0.33155
EFTUD2 CCDC103	-0.61809	-0.84855	-1.00675	-0.87353	-0.93027

EGF	0.185179	-0.27936	-0.94768	-0.38765	-0.77237
EGFL6	0.267129	0.455366	0.21306	0.347943	0.219572
EGFL7	0.238959	0.103013	0.323936	0.239558	0.312693
EGFL8	0.329845	0.113798	0.14971	0.337903	0.459899
EGFL9	0.126319	-0.0947	-0.18383	-0.20157	-0.31874
EGFR	-0.05399	0.092364	-0.03879	0.090132	-0.03597
EGLN1	0.047859	-0.08519	-0.10443	-0.36484	-0.15255
EGLN3	-0.36042	-0.33464	-0.37645	-0.17839	-0.25002
EGR1	-0.13526	-0.10879	-0.25778	-0.1333	-0.12822
EGR2	-0.69282	-0.2191	-0.51604	0.007661	-0.32877
EGR3	-0.64971	-0.97855	-1.29009	-0.56503	-0.97266
EGR4	-0.10677	-0.08416	0.006316	0.083989	0.032834
EHBP1	-0.96212	-0.51648	-0.91966	-0.87562	-0.70006
EHD1	-0.21983	-0.14348	-0.31319	-0.1945	-0.09408
EHD2	-0.02906	0.146584	0.38223	0.149987	-0.02064
EHD4	0.010413	0.094626	0.156938	0.051849	0.079592
EHF	-0.44782	-0.48721	-0.83847	-0.77051	-0.66129
EHHADH	0.770143	0.248877	0.09541	0.388167	0.041884
EHMT2	-0.36754	-0.33158	-0.44865	-0.41179	-0.55974
EI24	-0.0383	0.187442	0.047323	0.058757	0.053758
EID2	0.155054	-0.00688	0.140071	0.022262	-0.10328
EID2B	0.067441	0.005622	-0.10373	-0.15942	-0.10231
EIF1	-0.21734	-0.26151	-0.37402	-0.29609	-0.26596
EIF1AD BANF1	-0.39303	-0.62821	-0.8159	-0.49898	-0.83769
EIF1AX	-0.03701	0.184066	0.081138	0.112404	0.160193
EIF1AY	0.094641	0.224917	0.458154	-0.1501	0.353543
EIF1B	-0.11727	0.079665	0.213782	0.059246	-0.0564
EIF2AK1	0.160492	0.180313	0.187802	0.01582	0.022317
EIF2AK3	-0.09446	-0.13918	-0.12685	-0.03821	0.214315
EIF2AK4	0.280279	0.491003	0.365946	0.369956	0.442084
EIF2B1 GTF2H3	0.163962	-0.60401	-0.67842	-0.27362	-0.47096
EIF2B2	-0.31334	-0.26808	-0.46305	-0.373	-0.34627
EIF2B3	-0.12648	-0.17291	-0.33075	-0.08528	-0.17006
EIF2B4 SNX17	-0.00014	-0.14355	-0.0354	0.026802	-0.04618
EIF2B5	-0.93574	-0.93491	-1.18102	-0.91447	-0.91941
EIF2C1	0.086058	0.325404	0.279544	0.292505	0.232959
EIF2C2	-0.11502	-0.16212	-0.13187	-0.09029	-0.10079
EIF2C3	0.107639	0.216769	0.083569	0.054559	0.04615
EIF2C4	-0.02027	0.123369	0.062922	0.096629	0.18606
EIF2S2	-0.03471	-0.01391	-0.07559	-0.11289	-0.33301
EIF2S3	-0.34366	0.035993	-0.44671	-0.01326	-0.177
EIF3CL	0.221004	-0.00116	-0.18301	-0.04824	-0.13803
EIF3D	0.080688	0.230406	-0.05726	0.003688	-0.22764
EIF3E	-0.55973	-0.56351	-0.63918	-0.88953	-0.86327
EIF3EIP	0.087179	0.384481	0.170793	0.260222	0.275435
EIF3F	-0.60059	-0.77868	-0.73387	-0.65876	-0.52179
EIF3G	0.326295	0.018367	0.154582	-0.00029	0.076941
EIF3H	-0.72615	-0.77551	-1.14532	-1.14081	-1.15899
EIF3M	0.114635	0.201792	-0.16163	0.15464	0.21111
EIF3S10	-0.07779	-0.21271	-0.19302	-0.08263	-0.21519

EIF3S8	0.103354	-0.22286	-0.21598	-0.03458	-0.14876
EIF4A2	-0.79952	-0.822	-0.94284	-0.79907	-0.91323
EIF4A3	-0.20584	-0.47961	-0.43928	-0.39977	-0.21753
EIF4B	-0.98635	-0.73349	-1.16334	-0.80107	-0.95535
EIF4E3 GPR27	0.095272	0.164605	0.265735	0.14302	0.233518
EIF4EBP1	-0.14745	-0.42032	-0.28027	-0.0684	-0.09027
EIF4EBP2	-0.40922	-0.30492	-0.55757	-0.34589	-0.50459
EIF4ENIF1	0.118892	0.042258	0.148613	-0.06954	0.047589
EIF4ENIF1 SFI1	0.089618	0.044206	0.021305	0.084411	0.296238
EIF4G2	-0.33956	-0.28784	-0.28271	-0.32588	-0.16371
EIF4G3	0.274916	0.503283	0.229449	0.044163	0.178359
EIF5	0.004651	0.093497	0.059454	0.104039	-0.05538
EIF5A	-0.5385	-0.20972	-0.63635	-0.43589	-0.31132
EIF5A2	0.105105	0.231461	0.037446	0.089181	0.074554
ELA1	0.390395	0.548198	0.454171	0.322053	0.298614
ELA2	0.246011	0.220366	0.242479	0.273121	0.39041
ELA3A	0.335468	0.354519	0.32571	0.243483	0.470747
ELA3B	0.35496	0.480329	0.442001	0.272293	0.442398
ELAC1	0.336517	0.556243	0.493367	0.424708	0.54917
ELAC2	-0.14323	0.006961	-0.14254	-0.10821	-0.06368
ELAVL1 HNRNPM	0.043723	-0.02531	0.065405	0.117772	0.095222
ELAVL2	-1.13664	-0.953	-0.99469	-1.03523	-0.96327
ELAVL3	0.268093	0.198417	0.185614	0.108211	0.260238
ELAVL4	-0.14658	-1.14063	-0.95987	-0.68408	-0.9025
ELF1	0.502574	0.62417	0.666657	0.465106	0.456107
ELF3	0.212028	-0.19199	0.128958	0.054819	-0.1511
ELF4	-0.12733	-0.07442	0.054244	-0.01958	0.068146
ELF5	0.458974	0.649554	0.734637	0.515447	0.492867
ELFN2	0.367297	0.433853	0.48472	0.356851	0.324237
ELK1	-0.05793	-0.18181	0.109321	-0.05114	-0.3097
ELK3	0.063983	0.27516	0.181346	0.046534	0.161897
ELK4	-0.64848	-0.27256	-0.4382	-0.60326	-0.52151
ELL2	0.256981	0.377394	0.569223	0.253701	0.07275
ELL3 HYPK	0.265818	0.186542	0.315934	0.484467	0.423575
ELMO2	0.062589	-0.29655	-0.40423	-0.23248	-0.20569
ELMOD2	0.144593	0.051077	0.208919	0.237893	-0.13186
ELN	0.04633	-0.11054	-0.33119	0.053438	0.23012
ELOVL1	-0.05461	-0.14608	-0.11387	-0.03511	-0.00702
ELOVL2	0.032329	0.274836	0.352781	0.296921	0.329976
ELOVL3	0.09176	0.397724	-0.18792	0.079924	0.177881
ELOVL4	-0.20518	-0.25407	-0.26308	-0.37965	-0.40692
ELOVL5	-0.10728	-0.10037	-0.20712	-0.31945	-0.2972
ELOVL6	-0.93658	-0.83625	-0.90296	-0.76159	-0.93762
ELP3	-0.02477	-0.29322	-0.81914	-0.37656	-0.56582
ELSPBP1	-0.1287	-0.97156	-0.65651	-0.45288	-0.70307
EMB	0.333795	-0.28921	0.178408	0.141604	0.270275
EMCN	0.286585	0.459342	0.370571	0.096586	0.076398
EMD	0.132572	-0.00919	0.391448	0.177555	0.160973
EMID1	0.183118	0.367158	0.310582	0.310089	0.373326
EMID2	-0.20697	-0.00097	-0.01178	0.323086	0.215969

EMILIN1	0.037069	-0.25243	-0.3964	-0.32868	-0.44807
EMILIN2	-0.16608	-0.21043	-0.02175	-0.02597	0.112376
EMILIN3	0.258529	0.279348	0.435131	0.22921	0.291428
EML1	0.301709	0.432213	0.318414	0.283556	0.440387
EML2	0.259989	0.36184	0.508021	0.333945	0.394372
EML3 ROM1	-0.07889	-0.09057	-0.26999	-0.13245	-0.16081
EML4	0.294905	0.365662	0.470307	0.176248	0.265092
EML5	0.069936	0.115599	-0.00435	0.002344	0.138429
EMP1	-0.70993	-0.3252	-0.1131	-0.17926	-0.28202
EMP2	0.083286	0.10524	0.143111	0.141955	0.264612
EMP3	-0.07871	0.319269	0.621953	0.261759	0.085918
EMR1	-0.38122	-1.69614	-1.40646	-1.23327	-1.29034
EMR2	0.216093	0.055522	-0.0487	0.118778	0.279291
EMR3	0.28428	-1.39576	-1.2902	-0.845	-1.18986
EMX1	0.128137	-0.00469	0.122322	0.068443	0.105338
EMX2	-0.73844	-0.20634	-0.09214	-0.31903	-0.41278
EN1	-0.32888	0.036327	0.284683	0.443881	0.137679
ENAH	-0.06919	-0.10221	-0.15904	-0.10152	-0.04616
ENAM	-0.81987	-0.80027	-0.72601	-0.75375	-0.81913
ENC1	-0.2398	-0.17388	-0.26135	-0.15299	-0.18955
ENDOG	0.129385	0.26137	0.277157	0.131866	0.162261
ENDOGL1	0.08457	0.073681	-0.11024	0.140342	-0.02976
ENG	0.109583	0.109237	0.20378	0.281904	0.546697
ENO1	-0.06066	-0.02887	-0.16928	0.034466	0.12076
ENOSF1	0.188651	0.399018	0.554495	0.474934	0.437229
ENOX1	0.355191	0.500541	0.526124	0.161494	0.273655
ENOX2	-0.17715	-0.30043	-0.78999	-0.38862	-0.45198
ENPEP	0.528965	0.337718	0.125823	0.412832	-0.0547
ENPP1	-0.09245	-0.18761	-0.28388	-0.00707	-0.23957
ENPP2	-0.54302	0.783328	1.058142	-0.25964	-0.87545
ENPP4	-0.09318	-0.3645	0.407005	0.364523	-0.4214
ENPP5	0.124313	-0.55761	-0.14082	0.020041	-0.22241
ENPP6	0.254446	0.547949	0.665231	0.284372	0.417107
ENPP7	0.227718	0.481584	0.376564	0.470514	0.39688
ENSA	-0.46745	-0.98383	-1.79158	-0.73504	-1.22093
ENTHD1	0.333757	0.23722	0.461943	0.170994	0.358528
ENTPD1	0.226675	0.034183	0.45773	0.26076	-0.46952
ENTPD2	0.131429	0.390571	0.361455	0.230845	0.37646
ENTPD3	0.010447	0.423734	0.58956	0.282685	0.322889
ENTPD6	0.027602	0.149626	0.140963	0.056631	0.058618
ENTPD7	-0.07872	0.038258	-0.26055	-0.02148	-0.29241
ENTPD8	-0.0912	-0.33671	-0.32107	-0.06933	-0.25373
EOMES	-0.046	0.083097	0.208662	0.114686	0.054314
EP300	-0.01583	0.172941	0.128341	-0.07546	0.054485
EP400	0.036087	-0.01211	-0.01198	0.157247	0.212247
EP400NL.	0.430734	0.437998	0.515708	0.156347	0.312836
EPAS1	-0.08344	0.032333	-0.01797	0.077796	0.018271
EPB41	0.309228	0.429604	0.407195	0.279209	0.443726
EPB41L3	0.223711	0.056496	-0.02749	0.124959	0.216471
EPB41L4B	0.166675	0.137456	0.126127	0.153059	0.336251

EPB41L5	0.300569	0.323582	0.399376	0.247993	0.365514
EPB42	-0.01599	0.131954	-0.0113	-0.15105	-0.11026
EPB49	-0.16984	0.066157	-0.1795	-0.13719	-0.1018
EPC1	-0.05288	-0.05615	-0.03299	-0.05984	-0.06004
EPC2	-0.08878	-0.17234	-0.06095	-0.03761	0.077731
EPDR1	0.21833	0.146362	0.087561	-0.07458	0.099588
EPGN	0.444054	-0.43332	-0.15525	-0.51073	-0.79076
EPHA1	0.230219	0.608694	0.589769	0.352466	0.235049
EPHA2	-0.28651	0.1636	0.029585	0.08538	0.19473
EPHA3	0.304762	-1.0776	-0.55901	0.132118	-0.78814
EPHA4	-0.39811	-0.36063	0.2237	-0.0583	-0.09664
EPHA5	-0.695	-0.65171	0.300386	-0.23091	-0.12968
EPHA7	-0.82699	-0.83903	-0.18157	-0.62246	-0.16879
EPHA8	0.179463	0.349716	0.419643	0.403513	0.564613
EPHB1	0.124819	0.145744	0.205961	0.107174	0.229483
EPHB2	0.055284	0.061754	0.020338	0.124469	0.082838
EPHB3	-0.0331	-0.01242	0.076267	-0.12711	-0.11904
EPHB4	-0.19408	-0.12607	0.027495	0.215671	-0.10681
EPHB6	-0.36109	-0.4881	-0.43406	-0.34838	-0.17659
EPHX1	0.329213	0.792398	0.482443	0.329661	0.531442
EPHX2	0.343066	0.497823	0.361567	0.3504	0.146961
EPM2A	-0.33747	-0.2067	-0.24545	-0.26882	-0.27387
EPN2	0.07263	0.097547	0.149663	0.077844	0.129395
EPN3	-0.1757	-0.40203	-0.29828	-0.34073	-0.28694
EPO	-0.05511	-0.17983	-0.09657	0.031289	-0.04867
EPOR RGL3	-0.06214	-0.12145	-0.1139	-0.00566	-0.3686
EPRS	-0.05932	0.026291	-0.17369	-0.08441	-0.01633
EPS15	-0.14798	-0.38091	-0.40739	-0.37604	-0.20331
EPS15L1	-0.01565	0.024561	0.082168	0.07307	-0.03607
EPS8	0.214903	0.239154	-0.32372	0.057961	-0.10905
EPS8L2	0.12085	0.222585	0.267325	0.103175	0.29168
EPS8L3	0.012418	0.12588	0.19433	0.279637	0.235886
EPSTI1	0.496727	0.816686	0.845388	0.55792	0.625715
EPX	0.178215	0.119294	0.086839	-0.16409	0.107292
EPYC	-0.43058	-0.48074	-0.45213	-0.3818	-0.51778
ERAF	0.260493	-0.78987	-0.06016	0.075667	-0.00721
ERAL1	-0.43227	0.062894	-0.50854	-0.26728	-0.10726
ERAS	0.489383	0.713979	0.343374	0.423647	0.488965
ERBB2	-0.21613	0.561397	0.802526	0.476636	0.233033
ERBB2IP	-0.35313	-0.19237	-0.32085	-0.17281	-0.09713
ERBB3.	-0.25668	-0.28749	-0.21147	-0.11708	-0.05153
ERBB4	-0.21208	-0.02804	-0.29794	-0.04852	-0.25868
ERC1	-0.08787	0.063677	0.095614	-0.01584	-0.0139
ERC2	-0.20352	-0.05912	-0.3287	-0.08474	-0.26809
ERCC1	-0.21968	-0.35892	-0.3617	-0.27629	-0.198
ERCC2	-0.07021	0.006938	-0.34322	-0.02082	-0.00338
ERCC3	0.171736	0.181591	-0.08579	0.17178	0.030123
ERCC4	-0.193	-0.29553	-0.44398	-0.35651	-0.40937
ERCC5	-0.49363	-0.37216	-0.50713	-0.39336	-0.39045
ERCC6L	0.423318	0.599305	0.300564	0.468892	0.433078

ERCC8 NDUFAF2	-0.52192	-0.62098	-0.66926	-0.5723	-0.44203
EREG	0.328082	-0.72922	0.36311	0.055623	-0.30599
ERF	-0.35206	-0.29987	-0.25976	-0.18779	-0.24574
ERGIC2	-0.28911	-0.45551	-1.0284	-0.98361	-1.1384
ERGIC3	-0.03432	0.052847	-0.0135	0.060429	0.132438
ERH SLC39A9	-0.70554	-0.90623	-0.8453	-0.5875	-0.69137
ERICH1	0.05089	0.03451	0.143717	-0.00167	0.083507
ERLIN1	-0.30761	-0.29292	-0.57288	-0.41598	-0.52345
ERMAP	0.372517	0.256535	0.26892	0.031467	0.120293
ERMN	0.192805	-0.14227	-0.88121	-0.13316	-0.5266
ERMP1	0.094923	0.052453	-0.23855	-0.15883	-0.23828
ERN2	0.403281	0.603479	0.532229	0.406924	0.595379
ERO1L	0.171699	0.213156	0.137856	0.071711	0.200138
ERO1LB	-0.10865	-0.51057	-0.57377	-0.39878	-0.49962
ERP27	0.04884	0.335211	0.207721	-0.06809	-0.01537
ERRFI1	-0.09844	0.03216	0.054675	0.106538	0.119266
ESAM	0.150969	0.096975	0.177787	0.187479	0.006862
ESCO1	0.033708	0.123697	0.102488	0.013393	0.197795
ESD	0.270514	0.564659	0.344784	-0.17613	0.134068
ESM1	0.297934	-0.2169	-0.3604	0.218945	-0.46379
ESNA1	-0.58151	-0.27449	-0.67093	-0.38615	-0.60896
ESPL1	-0.11123	-0.29651	-0.39255	-0.32022	-0.2695
ESPNL	0.222795	0.234573	0.507245	0.20141	0.330351
ESR1	0.142262	-0.21741	0.179127	0.001397	-0.03592
ESR2	-0.14074	-0.42604	0.60766	0.128025	-0.30636
ESRRB	0.402651	-0.72145	-0.53824	0.267255	-0.5893
ESX1	0.00171	-0.02841	0.452792	0.422243	0.401085
ETAA1	0.040318	0.038871	0.009842	-0.59694	0.145638
ETF1	-0.13072	-0.25333	-0.2693	-0.11707	-0.10753
ETFA	0.13533	0.075938	0.196696	-0.09625	-0.04437
ETHE1	-0.034	-0.03769	0.083259	0.002253	0.175828
ETNK1	0.429562	0.660719	0.705391	0.228554	0.574198
ETNK2 AL592146.8	0.10229	0.082434	0.156726	0.051914	0.208349
ETS1	-0.77678	-0.2975	-0.27396	0.093891	-0.14877
ETS2	-0.14319	-0.19981	-0.15058	-0.09235	-0.04522
ETV1	-1.32421	-1.7372	-1.50226	-1.13813	-1.35873
ETV2	0.27095	0.39783	0.471374	0.137648	0.28704
ETV3	-0.19887	-0.32232	-0.07835	-0.21138	-0.07997
ETV3L	0.288736	0.353529	0.710583	-0.16122	0.351377
ETV4	-0.39016	-0.51509	-0.35113	-0.13038	-0.36198
ETV5	-0.54886	-0.18972	-0.37197	-0.38996	-0.47071
ETV6	-0.55555	-0.48517	-0.56232	-0.56479	-0.65759
EVC2 EVC	-0.01322	-0.08342	-0.08408	-0.02323	0.075284
EVI1	-1.08372	-0.06967	-0.59151	-0.55669	-0.5429
EVI5	-0.13684	0.384031	0.373995	-0.42616	0.081929
EVI5L	-0.33114	-0.49716	-0.20711	-0.1559	-0.1893
EVL	0.359794	0.343444	0.318928	0.03257	0.195934
EVPL	0.114796	0.133588	0.12476	0.106405	0.227521
EVX1	-0.23485	0.257488	0.693302	0.434139	0.375
EXDL1 AC012652.1	-0.03747	0.139907	0.126073	0.12946	0.246679

EXDL2	0.711293	0.744785	1.072699	0.489034	0.801723
EXO1	-0.34193	-0.83522	-0.6218	-0.42929	-0.41498
EXOC1	-0.26627	-0.37445	-0.58275	-0.36863	-0.64202
EXOC3	0.175593	0.114762	-0.04583	0.098928	0.115967
EXOC3L E2F4	0.006286	0.218719	0.019532	-0.0832	-0.07103
EXOC3L2	0.210177	0.130844	0.706705	0.340962	0.386431
EXOC4	-0.11767	-0.14129	-0.59116	-0.21986	-0.39468
EXOC5 C14orf108	-0.44703	-0.50616	-0.37621	-0.44148	-0.49024
EXOC7	0.209757	0.327356	0.343101	0.11581	0.106852
EXOC8 C1orf124	-0.35553	-0.48399	-0.506	-0.41176	-0.46395
EXOD1	-0.2319	-0.12323	-0.35292	-0.14379	-0.21108
EXOD1 LYRM1	-0.69878	-0.55436	-0.80417	-0.55782	-0.72239
EXOSC1 ZDHHC16	-0.28855	-0.59171	-0.51685	-0.45256	-0.45305
EXOSC10	0.05382	0.23329	0.291979	0.136494	0.124723
EXOSC2	0.049128	0.085187	0.160285	-0.01976	-0.10295
EXOSC4	-0.22675	-0.39396	-0.31848	-0.24192	-0.11375
EXOSC6 AARS	0.023098	0.343689	0.300724	0.158594	0.18414
EXOSC9	-0.08161	0.117524	-0.22284	0.09405	-0.14811
EXPH5	-0.5817	-0.44972	-0.62548	-0.55021	-0.6144
EXT1	-0.01315	-0.044	0.146454	-0.082	0.049443
EXT2	0.092658	0.179104	0.32183	0.097255	0.185615
EXTL1	-0.28426	-0.42744	-0.8416	-0.57828	-0.35803
EXTL2 SLC30A7	-0.95916	-0.818	-0.76336	-1.01133	-0.97866
EXTL3	1.687676	0.027256	-0.08748	0.064747	-0.09614
EYA3	-0.34961	-0.36464	-0.52051	-0.28877	-0.43167
EYA4	-0.21147	0.10429	-0.35891	-0.33941	-0.34719
EZH1	0.184246	0.177501	0.189076	-0.133	-0.00436
EZH2	0.16987	0.003764	0.190419	0.085811	0.084933
F10	0.156384	0.25682	-0.05225	0.237041	0.178519
F11	-0.08092	0.054552	-0.08511	-0.05567	-0.17286
F11R	-0.24402	0.305796	0.563815	0.548821	0.145554
F11R RP11-544M22.4 USF1	0.382464	0.379631	0.096071	0.395316	0.406731
F12	0.32623	0.204992	0.357764	-0.0032	0.252314
F13A1	-0.05427	0.465651	-0.09712	-0.07451	0.326687
F13B	-0.22853	-0.62514	-0.37544	-0.40919	-0.49665
F2	0.278749	0.28411	0.374612	0.021475	0.235314
F2R	0.229117	0.052265	0.124703	0.04652	0.055392
F2RL1	0.30544	0.180294	0.331691	0.415131	0.480592
F2RL3	0.288431	0.057866	0.103077	0.117328	0.327629
F3	-0.04925	-0.00559	0.198711	0.11823	0.092018
F5	0.141734	-0.40273	-0.05104	-0.30754	-0.25304
F7	0.231069	0.168844	0.491652	0.209595	0.239692
F8A3 H2AFB3	0.502121	0.546015	0.514902	0.557611	0.617688
F9	-0.32751	-0.17638	-0.47176	-0.28237	-0.20766
FA2H	0.196422	-0.03076	-0.10907	0.064229	-0.13893
FAAH	0.400536	0.432107	0.42159	0.292887	0.372431
FAAH2	-0.14542	-0.3596	0.043566	-0.09552	-0.10163
FABP1	0.501883	0.672759	0.770922	0.380091	0.487587
FABP2	-0.09829	-0.31126	-0.2981	-0.50777	-0.87643
FABP3	-0.21176	0.231237	0.360394	0.288269	-0.13004

FABP4	0.353318	0.523476	0.596974	0.328789	0.007856
FABP5	0.057706	0.407247	0.224847	0.00043	-0.02962
FABP6	0.386555	0.388313	0.699862	0.344538	0.384524
FABP7	-0.36437	1.39252	-0.63034	0.56518	-0.37056
FADD	0.425829	0.614781	0.569873	0.328843	0.600353
FADS6	0.090251	-0.07158	0.099511	0.374151	0.253531
FAF1 CDKN2C	-0.06762	0.048123	0.007539	-0.13329	0.069797
FAH	-1.02198	-0.62001	-0.91977	-0.695	-0.6943
FAHD2A	-0.10938	0.034071	-0.04058	-0.09058	-0.02307
FAHD2B	0.014682	-0.08473	-0.17295	-0.12627	-0.02442
FAIM	0.344959	0.152739	0.134715	-0.0086	0.101168
FAIM2	0.323273	0.424545	0.099966	0.174279	0.059643
FAIM3	-0.01062	-0.5085	-0.11771	-1.50086	-0.65339
FALZ	-0.05511	-0.07565	-0.02038	-0.05947	0.009459
FAM100A	0.239539	0.276259	0.225811	0.23193	0.180842
FAM100B	0.030252	-0.03584	-0.11132	0.029012	-0.10373
FAM101A	0.662859	0.736756	0.964412	0.407278	0.398359
FAM101B	0.450568	0.564146	0.398985	0.440383	0.366432
FAM102B	-0.0563	0.066635	-0.01399	-0.08755	-0.11403
FAM103A1	0.281033	-0.49876	-0.79964	-0.41374	-0.50977
FAM104A C17orf80	-0.14147	-0.43435	-0.49079	-0.42823	-0.33905
FAM104B	0.155531	-0.1997	-0.23982	0.148739	-0.11174
FAM105A	0.050284	0.43977	0.265628	0.314519	0.453689
FAM105B	0.483709	0.58745	0.634904	0.145552	0.418304
FAM107A	0.47189	0.691599	0.65599	0.537953	0.720872
FAM107B	0.65368	0.847189	0.78389	0.506475	0.516337
FAM108A4	-0.13704	-0.11919	-0.0689	-0.02713	0.155659
FAM108B1 C9orf85	-0.35294	-0.33515	-0.1876	-0.25656	-0.17697
FAM109A	0.008416	-0.00016	-0.25706	-0.03417	-0.16106
FAM109B	0.219403	0.266947	0.427393	0.272717	0.216075
FAM109B C22orf32	0.108684	0.119868	0.187131	0.072858	0.226027
FAM111A	0.252328	-0.28416	0.719372	0.294693	0.249455
FAM111B	-0.25494	-0.39561	-0.50442	0.307727	0.139982
FAM112A	0.62228	0.914938	1.095028	0.467944	0.649704
FAM113A VPS16	0.143935	0.213975	0.172112	0.115343	0.316495
FAM113B	0.575062	0.750355	-0.50283	0.364475	0.45251
FAM114A1	0.154031	0.362093	0.246839	0.242387	0.374218
FAM115B	-0.20321	-0.06642	-0.15243	-0.22179	-0.16036
FAM116A	0.300293	0.325863	0.472973	0.208764	0.255917
FAM116B	0.198976	0.068599	0.229799	0.035033	0.229647
FAM117A	-0.61423	-0.5723	-0.69778	-0.75453	-0.49444
FAM118A	0.07607	0.1248	0.126152	0.024482	0.038233
FAM119A	-0.13651	-0.10445	-0.07265	-0.11117	0.020297
FAM119B TSFM	-0.50491	-0.28987	-0.57774	-0.45052	-0.59508
FAM120AOS FAM120A	-0.07005	-0.02126	-0.04885	-0.09846	-0.03789
FAM120B	0.085948	0.193677	0.224986	0.04051	0.094803
FAM120C	0.194661	0.333205	0.578297	0.093847	0.074672
FAM122B	-0.28464	-0.43714	-0.47335	-0.29273	-0.29838
FAM122C	0.494317	0.263161	-0.3378	-0.06652	-0.30882
FAM123A	-0.03802	-0.13461	-0.289	-0.43606	-0.22488

FAM123B	0.003963	-0.13517	-0.15847	-0.09984	0.000243
FAM123C	-0.0326	0.252017	0.354137	0.041706	0.407867
FAM124A	-0.09818	-0.01864	0.044689	0.041813	0.046345
FAM124B	0.609837	0.716429	0.956608	0.528588	0.570255
FAM125A	0.042418	-0.08456	-0.03036	0.037825	-0.21137
FAM125B	-0.24157	-0.02497	-0.09156	-0.16872	-0.12722
FAM126A	0.013179	-0.55194	-0.4968	-0.29141	-0.39461
FAM126B NDUFB3	-0.6566	-0.67834	-0.7853	-0.66568	-0.83931
FAM127B	0.129583	-0.08435	0.043811	-0.29701	-0.26758
FAM129A	0.335229	0.41293	0.374123	0.325594	0.218352
FAM129C	0.214615	0.370103	0.527768	0.245388	0.237151
FAM12A	0.178869	-1.61484	-1.53254	-1.04563	-1.35714
FAM12B	-0.38719	-1.26173	-1.22452	-1.15897	-1.1047
FAM130A1	-0.0037	0.116317	0.05312	-0.01008	0.060874
FAM130A2	-0.29647	-0.59265	-0.57406	-0.51215	-0.29213
FAM131A	0.390389	0.14862	0.448332	0.40541	0.496175
FAM131B	-0.39043	-0.05077	-0.30464	-0.17326	-0.02077
FAM131C	-0.03987	0.136898	0.022284	0.105731	-0.05175
FAM134C TUBG1	-0.48802	-0.38463	-0.56488	-0.65006	-0.35774
FAM135A	-0.42331	-0.40091	-0.64994	-0.37323	-0.47513
FAM136A	0.073584	-0.08684	0.039235	-0.06488	0.115559
FAM139A	-0.79511	-0.70724	-1.44064	-0.47707	-0.72727
FAM13C1	-0.48019	-0.45749	-0.80086	-0.55375	-0.57555
FAM148A	0.146334	0.587752	0.866316	0.24349	0.391539
FAM148B	0.448052	0.536116	0.509575	0.500986	0.643189
FAM149A	0.030472	0.269558	-0.05222	0.019344	0.129371
FAM14A	-0.76191	-0.35279	-0.89189	-0.6853	-0.84344
FAM150A	0.062107	0.170807	0.411705	0.300671	0.458157
FAM150B	0.118307	0.264348	0.184023	0.178588	0.223883
FAM151B	0.193138	0.404663	0.379871	0.282805	0.362457
FAM152A	-0.03961	-0.26717	-0.17515	-0.10661	-0.17888
FAM152B XRCC6	-0.46155	-0.39384	-0.34327	-0.50369	-0.70533
FAM154A	0.441159	0.594499	0.891878	0.451595	0.617224
FAM18B	0.30223	0.355618	0.143439	0.14199	0.199511
FAM19A3	-0.57615	-0.71461	-1.01251	-0.79801	-0.73452
FAM19A4	0.267087	0.360248	0.594616	0.537932	0.495758
FAM19A5	0.351243	0.527142	0.5715	0.415493	0.49203
FAM20A	0.298416	0.406646	0.2675	0.241705	0.397506
FAM20B	0.211383	0.242054	0.218804	0.079021	0.183789
FAM20C	-0.02774	0.029079	0.07575	-0.04044	0.112474
FAM21B	-0.29818	-0.09839	-0.28251	-0.17478	-0.15573
FAM21C	-0.27186	-0.25389	-0.62917	-0.34672	-0.43329
FAM23B	0.41326	0.796057	0.837869	0.308322	0.612724
FAM24A	-0.37788	-0.3173	-0.27359	-0.27272	-0.45277
FAM24B	0.331423	-0.24324	0.279487	0.177007	-0.00232
FAM26A	0.309299	0.47272	0.453249	0.300265	0.355929
FAM26B	0.155185	0.083287	0.447842	0.002801	0.001735
FAM26C	0.236704	0.368631	0.487605	0.308919	0.418886
FAM26F	0.366094	0.510332	0.45591	0.180048	0.457005
FAM27E1 RP11-34H11.3	0.576267	0.77936	0.744708	0.646348	0.768538

FAM27L	0.278405	0.180749	0.225995	0.176365	0.35021
FAM29A	0.136539	0.258155	0.229843	0.115119	0.18181
FAM32A	-0.01509	0.059307	-0.0395	-0.06894	-0.07696
FAM33A. PRR11	-0.78344	-0.8964	-1.06207	-0.99804	-0.70931
FAM38A. AC138028.1	0.135348	0.076211	0.216018	0.092247	0.176048
FAM38B	0.094966	0.307148	-0.85347	-0.55073	-0.25728
FAM39B.	-0.3194	-0.40907	-0.64425	-0.27291	-0.23664
FAM3A	0.152758	-0.19623	-0.01749	-0.0606	-0.07797
FAM3B	0.070572	-0.0668	0.226412	0.32099	0.336795
FAM3C	-0.06749	-0.02763	-0.07543	-0.12859	-0.24568
FAM3D	0.30436	0.219706	0.457909	0.209972	0.099013
FAM40A	-0.00633	-0.02473	-0.11252	-0.26661	-0.21942
FAM40B	-0.21903	-0.06507	-0.1338	-0.17204	-0.25984
FAM43A	-0.01775	0.131242	0.070571	0.010225	-0.0189
FAM43B	-0.09271	-0.26972	0.171988	0.193179	-0.00901
FAM44A	0.942613	-0.42713	-0.3621	-0.42423	-0.29841
FAM44B	0.141519	-0.20518	0.2068	-0.04737	0.202646
FAM45B	0.0128	0.193796	0.151871	0.097814	0.029768
FAM46A	-0.53034	-0.45969	-0.54897	-0.32294	-0.32171
FAM46B	0.045439	0.285011	0.033351	0.15505	0.26518
FAM46C	-0.26282	-0.14504	-0.14911	-0.19056	-0.2703
FAM46D	-0.59919	-0.63873	-0.71924	-0.48192	-0.71342
FAM47A	0.33875	0.437305	0.447376	0.195918	0.383148
FAM47B	0.335676	0.394553	0.409571	0.240948	0.420328
FAM47C	0.404139	0.47934	0.446968	0.31309	0.411735
FAM48A	-0.13089	-0.1165	-0.11844	-0.11114	-0.39567
FAM49A	-0.04235	0.055841	0.625531	-0.0216	0.117363
FAM49B	-0.44929	-0.48949	-0.48572	-0.50911	-0.61239
FAM50B	0.374338	0.212473	0.468324	0.358805	0.387467
FAM53A	0.171144	0.167429	0.056159	0.155655	0.169868
FAM53B	-0.23012	-0.18031	-0.27181	-0.39047	-0.12011
FAM53C	-0.27246	-0.27394	-0.41476	-0.33217	-0.24599
FAM54A	-0.70002	-0.81794	-1.56342	-0.96304	-1.18937
FAM54B	-0.06165	-0.12139	-0.12066	-0.05348	-0.00635
FAM55A	0.059789	0.122641	-0.25962	-0.37327	-1.03296
FAM55B	-0.63349	-0.24047	-0.27705	-0.35709	-0.8687
FAM55C	0.015936	0.086181	-0.0756	0.000196	0.119668
FAM55D	-0.06637	-0.05479	-0.13643	-0.25378	-0.28895
FAM57A	0.013731	0.071909	-0.01348	0.06707	0.074311
FAM57B	-0.2695	-0.15983	-0.39263	-0.46438	-0.46201
FAM58A	0.159399	0.193749	0.416777	0.128523	0.237724
FAM59A	0.041334	0.120613	0.183801	0.055353	0.071771
FAM5B	-0.02665	-0.13277	-0.51749	-0.22043	-0.12234
FAM5C	-0.43272	-1.71629	-0.98687	-0.66441	-0.89246
FAM60A	-0.44144	-0.25837	-0.52345	-0.43294	-0.60084
FAM62A	-0.66869	-0.79105	-0.90523	-0.73011	-0.69904
FAM62B	-0.0953	-0.08729	-0.20508	-0.11925	0.114234
FAM62C	0.199989	0.509079	0.652678	0.500855	0.430363
FAM63A PRUNE	0.035727	-0.00634	-0.23305	-0.16169	0.125111
FAM63B	-0.02129	0.111972	-0.11656	0.011033	0.097266

FAM64A	-0.26941	0.002075	-0.37849	-0.24757	-0.18505
FAM65A	0.134632	0.020564	0.116975	0.03753	0.190259
FAM69A	-0.06059	-0.06167	0.029658	-0.02323	0.125463
FAM69B	-0.16904	-0.14952	-0.18542	-0.22996	-0.15547
FAM70A	-0.12786	-0.39632	-0.01703	-0.44242	-0.41272
FAM70B	0.064858	0.068046	-0.13888	-0.01133	-0.01812
FAM71A	0.138359	0.418324	0.403292	0.07275	0.365506
FAM71B	0.179483	0.342146	0.556985	0.266266	0.100832
FAM71D	0.48681	0.529275	0.83268	0.175412	0.038003
FAM71E1 C19orf63	0.381434	0.412794	0.47234	0.337104	0.335752
FAM71F1	0.306546	0.489858	0.410356	0.168239	0.245212
FAM71F2	0.277224	0.17527	0.161191	0.040452	0.086571
FAM73A	-0.10518	0.324051	-0.00843	0.182141	0.244291
FAM73B	-0.23394	-0.17961	-0.28302	-0.16676	-0.12661
FAM74A4	0.134409	-1.04296	-0.82416	-0.15368	-0.83447
FAM75A1	0.502393	0.075289	0.176838	0.055268	0.086941
FAM75A7	0.181071	0.225857	-0.18431	0.045559	-0.01424
FAM76A	-0.03619	0.042476	0.207614	-0.17016	0.137994
FAM76B CEP57	-0.29391	0.023807	-0.38657	-0.25728	-0.26231
FAM78A	0.249273	0.449741	0.734495	0.486448	0.527433
FAM78B	-0.21169	-0.49528	-0.51055	-0.38309	-0.17451
FAM80A	-0.22304	-0.16259	-0.25913	-0.12555	0.029087
FAM81A	-0.30449	-0.06233	-0.19122	0.130343	-0.11333
FAM81B	-0.39881	-0.50736	-1.25105	-0.69492	-1.03504
FAM82A	0.581753	0.757648	0.63872	-0.00196	0.219232
FAM82B CPNE3	0.217549	0.380159	0.322077	-0.1793	0.124457
FAM82C	0.11956	0.391181	0.278896	0.026414	0.065609
FAM83A	0.40031	0.514288	0.63767	0.548535	0.498162
FAM83B	0.10557	0.266311	0.380843	0.423924	0.42892
FAM83C	0.367129	0.548652	0.478575	0.359866	0.49379
FAM83D	0.367914	0.52158	0.716924	0.349388	0.451742
FAM83F	0.341551	0.226031	0.571222	0.461354	0.434312
FAM83H	0.189921	-0.0616	0.271067	0.264883	0.419957
FAM84A	-0.45735	-0.01017	-0.04045	-0.75966	0.133729
FAM84B	0.004079	0.072979	0.139204	-0.00848	0.137761
FAM86A	-1.039	-0.90535	-1.36279	-0.83794	-1.0515
FAM86C	-0.77014	-0.84562	-0.94657	-0.64278	-0.67699
FAM89A	0.00744	-0.41029	-0.20891	-0.42485	-0.4296
FAM8A1	0.351788	0.370516	0.501372	0.282914	0.405705
FAM90A1	0.410923	0.579292	0.637136	0.376176	0.610304
FAM92B	0.433659	0.563189	0.673107	0.363852	0.549219
FAM96A SNX1	-0.21184	-0.539	-0.63255	-0.2549	-0.30332
FAM96B CES2	-0.35302	-0.52104	-0.79514	0.050327	-0.42578
FAM98A	-0.52146	-0.68739	-0.71302	-0.45653	-0.56912
FAM98B	-0.15816	-0.13684	-0.4853	-0.23941	-0.48889
FAM98C	0.095954	0.008285	0.002206	-0.01705	0.029247
FAM9A	0.25974	0.522496	0.650012	0.235483	0.445774
FAM9B	-0.08265	0.109132	0.023704	0.113056	0.12221
FAM9C	0.324724	0.212374	0.334802	0.251859	0.129834
FANCA	-0.02555	-0.02868	-0.07639	-0.04058	-0.16297

FANCB MOSPD2	-0.06959	-0.45112	-0.0546	-0.35451	-0.56843
FANCC	0.079781	0.165805	0.0955	-0.03018	0.063148
FANCE	-0.00172	-0.10684	0.107391	-0.19243	-0.26622
FANCF	0.003371	-0.21783	-0.08215	-0.33977	-0.09937
FANCL	-0.21006	-0.17983	-0.32189	-0.52559	-0.10515
FANK1	0.184367	-0.10104	0.326909	0.219012	0.237395
FAP	-0.73909	-0.81064	-0.55292	-0.65799	-0.38242
FARP2	-0.15173	-0.24633	-0.12473	-0.10241	-0.12205
FARSA	0.259909	0.16314	-0.03127	0.160554	0.017622
FARSLB	-0.10791	-0.69955	-0.61994	-0.663	-0.75969
FAS	-0.47012	0.512127	0.531114	0.464388	0.574774
FASLG	0.086306	0.054925	0.084735	-0.05742	0.018349
FASN	-0.05108	-0.00039	0.060413	0.001353	-0.01476
FASTK TMUB1	0.043847	-0.04298	0.09542	0.100699	0.079021
FASTKD1	-0.33701	-0.20361	-0.43385	-0.46526	-0.15502
FASTKD3 MTRR	-0.17795	-0.10758	-0.16026	-0.13838	-0.21184
FAT	-0.1022	-0.04036	0.229422	-0.00751	0.265435
FAT2	0.123283	-0.09952	0.413712	-0.1259	0.190913
FATE1	-0.11882	-0.37618	-0.41853	0.046803	0.054098
FAU	-0.12815	-0.22723	-0.37207	-0.27585	-0.03644
FBL	-0.20902	-0.16856	-0.3706	-0.27484	-0.44611
FBLN1	-0.06259	0.171881	-0.00637	0.077798	0.151705
FBLN2	0.020506	-0.03017	0.047675	-0.01067	0.114611
FBLN5	-0.12595	-0.57882	-0.60922	-0.0955	-0.15572
FBLN7	0.027016	0.315483	-0.18985	0.237257	0.403703
FBN1	-0.15111	-0.10538	-0.21052	0.005478	-0.06345
FBN2	-0.72689	0.061286	-0.02156	0.006284	0.215787
FBP1	0.338462	0.650246	0.751878	0.437571	0.640932
FBP2	0.544791	0.685008	0.772538	0.563281	0.6501
FBRS	0.214048	0.340269	0.37256	0.272939	0.266372
FBXL10	-0.18031	-1.21044	-1.36495	-0.68552	-1.23157
FBXL11	-0.33443	-0.26596	-0.35916	-0.23968	-0.35118
FBXL12	-0.3191	-0.27232	-0.45309	-0.28993	-0.48281
FBXL13	-0.2852	0.484925	0.526928	-0.74846	-0.13135
FBXL13 ARMC10	-0.22368	-0.09237	-0.25189	-0.24799	-0.25283
FBXL14	-0.05139	0.099474	0.111352	0.110863	0.0764
FBXL16	0.048459	0.000823	0.104277	0.080322	-0.0014
FBXL17	0.060199	0.089749	0.08899	0.110622	0.076301
FBXL18	0.070414	0.131467	-0.36015	-0.19216	-0.20167
FBXL19 ORAI3	-0.38369	-0.2841	-0.41588	0.283536	-0.48369
FBXL2	0.16519	0.137109	0.264049	0.163109	0.207066
FBXL20	-0.0765	-0.18931	-0.26585	0.009005	-0.17618
FBXL21.	-0.19991	0.091522	-0.24379	-0.2848	-0.91399
FBXL22	0.535913	0.476614	0.519866	0.36193	0.434491
FBXL3	-0.05992	0.076929	-0.01629	-0.04048	-0.02557
FBXL4	0.19387	0.072224	0.196483	0.164993	-0.0752
FBXL5	0.122455	0.145231	0.249514	0.188713	0.291829
FBXL6 GPR172A	0.119109	-0.03658	-0.01304	0.107244	0.191266
FBXL8 HSF4	-0.26139	0.005037	-0.06274	-0.00764	-0.03345
FBXO11	-0.44084	-0.37383	-0.44865	-0.32977	-0.26636

FBXO15 C18orf55	-0.14136	-0.37983	-0.27948	-0.15687	-0.07646
FBXO16	0.017614	-0.20346	-0.8236	-0.55059	-0.81384
FBXO18	0.551108	0.769515	0.728679	0.2287	0.41305
FBXO2 FBXO44	0.136344	0.287719	0.131956	0.136944	0.192509
FBXO21	0.032947	0.055227	0.253632	0.146589	0.227068
FBXO22	0.156224	0.29864	0.329241	0.239166	0.234122
FBXO25	-0.20444	-0.01163	-0.37808	0.06919	-0.1196
FBXO27	0.228267	-0.12849	0.304884	0.245849	0.319482
FBXO28	-0.074	-0.0558	-0.0942	-0.2387	-0.11842
FBXO3	-0.21192	-0.32751	-0.3422	-0.24606	0.004793
FBXO30	-0.09788	0.155452	-0.02251	-0.03711	-0.12079
FBXO31	-0.1148	-0.05184	-0.10194	0.108311	0.11402
FBXO33	-0.22747	-0.06361	0.062792	-0.03373	-0.14733
FBXO34	-0.30706	-0.22708	-0.39617	-0.30509	-0.36458
FBXO38	-0.58856	-0.63872	-1.35989	-0.80141	-0.95375
FBXO4	0.442217	0.568726	0.278253	0.446723	0.182126
FBXO40	0.243203	0.633134	0.784121	-0.15029	0.130114
FBXO42	0.239574	0.130026	0.171243	0.173339	0.232126
FBXO43	0.290087	0.050844	0.746782	-0.11956	0.153785
FBXO47	0.669942	0.945241	0.821504	0.590274	0.571083
FBXO48 C2orf13	-0.25509	-0.29299	-0.24216	-0.24467	-0.04691
FBXO5	-0.16262	-0.02627	-0.18965	-0.11117	-0.15265
FBXO6	0.094593	0.146231	0.144527	0.048987	0.351521
FBXO7	-0.11067	0.053319	0.05296	0.004105	0.139507
FBXO8 KIAA1712	-0.65117	-1.14769	-0.88723	-0.83547	-0.78005
FBXW10	0.410686	0.461648	-0.34514	0.005022	-0.38747
FBXW11	-0.1566	-0.2232	-0.47603	-0.20505	-0.3301
FBXW12	0.574857	0.488169	0.813279	0.421725	0.50467
FBXW2	-0.35466	-0.16982	-0.26495	-0.27261	-0.16509
FBXW4	0.13191	0.132605	0.15517	0.000401	-0.0695
FBXW5 C8G	0.049298	0.126084	0.16578	0.1555	0.170625
FBXW8	0.032279	0.2002	0.083743	-0.02363	0.029246
FBXW9	0.151068	0.061201	0.161929	0.105477	0.060445
FCAMR	0.226452	-0.28611	0.117176	-0.46492	-0.01012
FCAR	0.271467	-0.3491	-0.30892	-0.18863	-0.14771
FCER1G	0.479313	0.616995	0.652917	0.35222	0.405118
FCER2	0.436981	-0.88932	-0.77784	-0.03713	-0.53011
FCGBP	-0.11436	-0.49377	-0.27613	-0.20427	-0.26786
FCGR1A	-0.76837	-1.71353	-1.85421	-1.66352	-1.46282
FCGR1B	-0.91621	-1.51309	-2.17588	-1.95559	-1.84705
FCGR2A	0.439753	-0.17053	0.178956	0.13434	0.267387
FCGR2B	0.075775	-1.85951	-1.61778	-1.34755	-0.97068
FCGR3A	0.133244	-0.99633	-1.24192	-0.79133	-0.25434
FCGR3B	0.081463	-1.36688	-1.44444	-1.19897	-0.43904
FCGRT	-0.0546	-0.24787	-0.17012	-0.19035	-0.0907
FCHO2	0.052089	0.096604	0.163403	0.132495	-0.06842
FCHSD1	0.049449	0.272443	0.14645	0.245441	0.223396
FCHSD2	-0.15661	-0.03659	-0.15849	-0.1262	-0.07445
FCN1	0.290202	0.535625	0.613965	0.524669	0.600554
FCN2	0.106422	0.191601	0.415446	0.137707	0.271293

FCN3	0.077398	0.072201	0.307564	-0.03397	0.186415
FCRL1	-0.55991	-1.13869	-0.98163	-0.9896	-0.95461
FCRL3	0.017493	-1.67967	-1.56374	-1.25119	-0.84775
FCRL4	-0.36818	-1.47893	-1.48651	-1.07049	-1.0757
FCRL5	-0.25861	-1.04731	-0.86624	-0.93755	-0.84283
FCRL6	0.374776	0.737477	0.624735	0.232377	0.467681
FCRLA	-0.53214	-0.85245	-0.95228	-0.72839	-0.74277
FCRLB	-0.2878	-1.10705	-1.0186	-0.74791	-0.49815
FDFT1	0.029248	0.09084	-0.16544	0.065703	-0.03634
FDPS	-0.34557	-0.5328	-0.84598	-0.52012	-0.39419
FDX1	0.207982	0.367878	0.320942	0.151799	0.282311
FDX1L RAVER1	0.136242	-0.23036	-0.62996	0.134559	-0.41688
FDXR	-0.71704	-0.67481	-1.11464	-0.86011	-0.78608
FECH	0.296086	0.511672	0.440394	0.22454	0.327635
FEM1A	0.042396	-0.08729	0.151079	0.1075	0.038554
FEM1B	-0.0264	-0.20908	-0.43974	-0.25441	-0.28637
FEM1C	0.151738	0.212485	0.312884	0.153235	0.099297
FER	0.131566	0.286578	0.272472	0.107547	0.465644
FER1L3	-0.4463	-0.03417	0.159652	-0.09691	-0.41939
FER1L6	-0.29304	-0.06064	-0.37382	-0.44039	-0.99512
FERD3L	0.095713	0.687707	0.842036	0.715351	0.678082
FERMT3	0.188925	0.21904	0.43734	0.287208	0.30201
FES	0.368167	0.441119	0.548302	0.26048	0.248351
FETUB	-0.47445	-0.73392	-0.70007	-0.50852	-0.85436
FEV	-1.19611	-0.62456	-1.2552	-0.7905	-1.09188
FEZ1 AP000708.1	0.032976	0.411811	0.498735	0.229932	0.140015
FEZ2	0.451617	0.549903	0.759886	0.414211	0.398945
FEZF1	-0.54617	-0.44965	0.249854	0.519946	0.232949
FEZF2	-0.449	-0.06641	0.479058	0.395046	0.176278
FFAR1	0.185043	0.296739	0.18526	-0.01762	0.308344
FFAR2	0.360879	0.105563	-0.14382	-0.05825	0.298909
FFAR3	0.640674	-0.21445	-0.41275	-0.26165	0.163687
FGA	-0.69984	-1.48552	-1.19926	-1.15491	-1.25247
FGB	-0.26012	-0.47934	-0.15879	-0.32841	-0.2854
FGD1	0.186088	0.239663	0.390539	0.274303	0.288856
FGD3	0.54346	0.700446	0.843269	0.416205	0.422174
FGD4	-0.58881	-0.34482	-0.84678	-0.71087	-0.60105
FGD5	0.5324	0.552866	0.457594	0.573622	0.573108
FGD6 VEZT	-0.20192	-0.00762	-0.07544	-0.07526	-0.20132
FGF1	0.131034	0.222313	0.147645	0.180945	0.113749
FGF10	-1.13004	-0.50167	-0.7013	0.291967	0.425851
FGF11	-0.03152	0.211975	-0.11271	-0.08127	-0.15161
FGF11 CHRNB1	0.017235	-0.10011	-0.38946	0.173354	-0.20051
FGF12	-0.01666	0.080023	0.112612	0.496811	0.20675
FGF16.	0.158754	0.171358	-0.04807	0.057144	0.226798
FGF17	-0.17329	0.218733	0.187836	-0.05255	0.243127
FGF18	-0.12753	-0.09302	0.029741	0.073981	0.119216
FGF19	-0.3688	0.111321	0.262681	-0.01265	-0.01482
FGF2	0.158288	0.466467	0.419078	0.315922	0.367034
FGF20	-0.13783	0.482986	0.398911	0.094429	0.324052

FGF21	0.177836	0.290755	0.446765	0.35881	0.441956
FGF22	0.146278	0.095703	0.035523	0.068713	0.123606
FGF23	0.471817	0.249584	0.471052	-0.62743	0.293421
FGF3	-0.02168	0.062259	-0.15815	0.040866	0.074297
FGF4	0.006728	0.152265	0.404786	0.346071	0.359931
FGF5	-0.64772	-0.4909	-0.39077	-0.24278	-0.13653
FGF6	0.592537	0.798918	0.93466	0.380169	0.707596
FGF8	-0.05629	-0.06489	0.144005	-0.0162	0.032422
FGF9	-0.78003	-0.95768	-0.52263	-0.06265	-0.65887
FGFBP1	-0.13017	0.227393	0.455854	0.002792	-0.68487
FGFBP3	-0.15359	-0.1866	-0.29994	-0.26906	-0.19178
FGFR1	0.221779	0.380727	0.651701	0.292775	0.265003
FGFR1OP	0.024884	0.019338	0.032597	5.72E-05	-0.06228
FGFR2	-0.0559	0.014107	-0.24018	-0.1505	-0.02007
FGFR3	-0.09674	0.187918	0.005094	0.044442	0.174207
FGFRL1	-0.13421	-0.12307	-0.22836	-0.15954	-0.00555
FGG	-0.08635	-1.32931	-0.77013	-0.58838	-0.86072
FGGY	0.163619	0.133737	-0.19842	-0.2418	0.084716
FGL1	-0.28484	-0.13112	-0.26777	-0.30465	-0.42876
FH	-0.08317	-0.35936	-0.46671	-0.37644	-0.19515
FHDC1	0.261275	0.308698	0.262748	-0.00092	-0.24444
FHL1	0.109633	0.038896	0.155261	0.091584	0.132687
FHL3	-0.00958	0.024273	0.148419	0.084449	0.24475
FHL5	-0.45647	-0.42261	-0.40688	-0.46146	-0.43279
FHOD1 SLC9A5	-0.37074	-0.31599	-0.38137	-0.29939	-0.48973
FHOD3	0.04003	0.080178	0.072734	0.022821	0.137193
FIBCD1	-0.08383	-0.10858	-0.00552	0.175497	0.055974
FIBP CCDC85B	-0.15379	0.011224	-0.22401	0.018432	-0.11519
FICD	-0.15293	-0.14765	-0.38753	-0.20295	-0.19405
FIGF PIR	-0.11231	0.237061	0.4443	-0.23644	0.364686
FIGLA	0.271686	0.486164	0.511735	0.325478	0.416784
FIGN	-0.59864	-0.44935	-0.46185	-0.43034	-0.30878
FIGNL1	0.268158	0.30594	0.411231	0.330385	0.435444
FILIP1	0.598013	-0.03971	0.119144	0.247889	-0.36742
FIP1L1	-0.10852	-0.19063	0.023392	-0.21697	-0.22011
FIS1	0.208721	-0.05432	-0.07135	-0.02193	0.189615
FIZ1 ZNF524	-0.39785	-0.28395	-0.32681	-0.26922	-0.38724
FKBP14 PLEKHA8	-0.32138	-0.47724	-0.84427	-0.3745	-0.6155
FKBP15 SLC31A1	-0.86337	-0.96751	-0.90477	-0.6179	-0.78524
FKBP1A	0.032646	0.238419	0.25573	0.005877	0.1139
FKBP2	-0.05753	-0.22326	-0.26632	-0.07228	-0.11737
FKBP3 FANCM	-0.99852	-0.79538	-0.42142	-0.80736	-0.90856
FKBP4	0.11698	0.215886	0.301804	0.116083	0.204396
FKBP5	0.16828	0.150716	0.336148	0.17471	0.15624
FKBP7 PLEKHA3	0.129362	0.12814	0.347208	0.061018	0.134397
FKBP9L.	0.203575	-0.34226	-0.1403	-0.07096	-0.42053
FKHL18	0.126228	0.343023	0.388351	0.226057	0.336892
FKTN	0.308011	-0.48153	-0.48413	-0.41699	-0.33945
FLAD1	-0.75434	-0.59464	-1.11916	-0.64406	-0.82172
FLAD1 LENEP	0.233981	0.6142	0.394919	0.399242	0.429549

FLCN	-0.3764	-0.2238	-0.6292	-0.33018	-0.35172
FLG	-1.07737	-1.47853	-1.47465	-1.35428	-0.97047
FLG2	-1.08242	-1.54586	-1.55299	-1.32265	-1.19585
FLI1	-0.60276	0.030179	-0.35761	-0.00349	-0.12145
FLII SMCR7	0.097701	0.230759	0.1402	0.434534	0.191353
FLNA	0.258877	0.236346	0.290556	0.262289	0.229077
FLNB	0.04073	0.325875	0.217414	0.052114	0.169056
FLNC	-0.08592	-0.26972	0.206673	-0.0952	-0.10192
FLOT1 IER3	0.070062	-0.21333	0.616118	0.343508	0.4098
FLOT2 DHRS13	-0.06516	0.096059	-0.05576	0.045697	0.155925
FLT1	-0.06442	-0.07193	0.264624	0.278425	0.128307
FLT3	0.142725	0.170446	0.489811	0.20529	0.335957
FLT3LG	0.225892	0.094429	0.371712	0.06495	0.075676
FLT4	-0.02497	0.127533	0.439449	0.238016	0.158756
FLVCR2	-0.47411	0.029385	-0.35409	-0.22627	-0.46196
FLYWCH1	0.327515	0.296309	0.411957	0.290742	0.280095
FLYWCH2	-0.13482	-0.26391	-0.4601	-0.21584	-0.31094
FMN2	-0.41036	-0.24378	-0.47703	-0.50426	-0.35551
FMNL1	0.168645	0.363387	0.51833	0.276621	0.41183
FMNL3	0.023493	0.070451	-0.08852	-0.14082	-0.12264
FMO1	0.313606	-0.97637	-0.82362	-0.70972	-0.55865
FMO2	-0.32016	-1.39673	-1.12914	-1.02803	-0.97385
FMO3	0.118527	-1.15625	-0.8418	-0.72151	-0.40597
FMO4	-0.69169	-1.11787	-1.0311	-1.03789	-0.91003
FMO5	0.150426	0.161362	0.278114	0.218617	0.401384
FMOD	0.369643	0.728133	0.710468	0.54662	0.523009
FMR1	0.098891	0.130044	0.291284	0.027657	0.26033
FMR1NB	0.545409	0.654937	0.818545	0.468525	0.590504
FN1	-0.53764	-0.21271	-0.45209	-0.12895	-0.40778
FN3K	0.370545	0.549411	0.540548	0.400275	0.396754
FNBP1	0.164025	0.260982	0.318223	0.12654	0.107124
FNBP4	-0.22997	-0.20529	-0.3098	-0.17539	-0.04044
FNDC1	-0.08535	0.150372	0.442949	0.326937	0.245492
FNDC3A	-0.33094	-0.29809	-0.1887	-0.22096	-0.36952
FNDC4 GCKR	0.183048	0.419592	0.204216	-0.09539	-0.06457
FNDC5	-0.03724	-0.00027	0.036804	-0.20014	-0.05574
FNDC7	0.831124	0.842749	1.107405	0.622379	0.771889
FNIP1	-0.14901	-0.19343	-0.23827	-0.18421	-0.2033
FNTA	-0.07818	-0.12687	-0.08418	-0.10742	-0.08865
FNTB	-0.08738	0.108721	-0.25399	-0.57851	-0.4498
FOLH1	0.196428	-0.28147	0.537632	0.124483	0.174074
FOLR1	-0.0559	0.085037	0.001655	-0.10891	-0.07956
FOLR2	0.23829	0.490959	0.19761	0.149283	0.315779
FOLR3	0.422866	0.252892	0.597053	0.286009	0.318172
FOS	-0.61789	-0.40514	-0.62205	-0.43289	-0.66706
FOSB.	-0.62205	-0.47299	-0.54581	-0.25216	-0.52571
FOSL1	0.068574	0.371117	0.415608	0.158523	0.365919
FOSL2	-0.09876	-0.15241	-0.08511	-0.00545	-0.06374
FOXA1	-0.25957	0.123535	0.104233	0.276239	0.318358
FOXA2	-0.43911	-0.04524	-0.06059	0.387002	0.347158

FOXB1	-0.17758	0.134291	0.746153	0.451288	0.477967
FOXB2	-0.25117	-0.00134	0.3194	0.268709	0.279563
FOXC1	-0.02421	-0.05498	0.014739	0.133537	0.182428
FOXC2	-0.11586	0.045894	0.274437	0.185255	0.262987
FOXD2	-0.12997	-0.85309	-0.82132	-0.21003	-0.23944
FOXD3	-0.29133	-0.15176	-0.01877	0.11101	-0.06718
FOXD4	0.417141	0.490685	0.846647	0.552324	0.736463
FOXD4L1	0.240055	0.493895	0.708696	0.601857	0.717282
FOXD4L3	0.225886	0.438629	0.840228	0.635924	0.586088
FOXD4L4	0.385119	0.277369	0.475606	0.110451	-0.04881
FOXE1	-0.10009	0.100478	0.536195	0.445575	0.550883
FOXE3	-0.28765	-0.33187	-0.05689	0.023858	0.14255
FOXF1	-0.04008	-0.05022	0.241029	0.051614	0.041544
FOXF2	-0.10854	0.168176	0.174131	0.242142	0.218004
FOXG1	-0.59184	0.099005	0.185852	0.170926	0.217246
FOXI1	0.375412	0.578893	0.715805	0.393923	0.455833
FOXJ1	0.259953	0.007639	0.522929	0.364359	0.301335
FOXJ2	0.068718	0.36366	0.335562	0.212327	0.183138
FOXJ3	-0.23701	-0.00278	-0.06357	0.02995	0.101262
FOXK1	0.072746	0.105483	0.18808	0.146265	0.187583
FOXK2	0.010705	0.128084	0.145871	0.092616	0.148206
FOXL1	0.261716	0.394132	0.391034	0.442791	0.471619
FOXL2 AC130416.4	0.112115	0.135011	0.353281	0.23323	0.484549
FOXM1 C12orf32	0.090608	-0.08625	-0.14903	-0.05329	-0.03642
FOXN1	0.158389	0.239814	0.17903	-0.02041	0.061659
FOXN2	-0.09476	-0.26024	-0.2561	-0.138	-0.0335
FOXN3	-0.003	-0.00987	-0.13814	-0.1172	-0.12863
FOXN4	-0.52619	-0.41089	-0.75357	-0.52744	-0.34725
FOXO1A	0.036624	-0.1588	-0.02528	0.023604	0.068992
FOXO3	-0.15255	-0.25734	-0.09599	0.001657	-0.12461
FOXO4	-0.2539	-0.83384	-1.01579	-0.71315	-0.84735
FOXP1	-0.76402	-0.59892	-0.75289	-0.35804	-0.75167
FOXP2	-1.01381	-1.25297	-1.46091	-1.01758	-1.17972
FOXP3	0.50643	0.674687	0.573162	0.40849	0.567295
FOXP4	-0.43814	-0.52178	-0.30236	-0.3509	-0.48936
FOXQ1	0.040531	0.241489	0.561315	0.239754	0.547324
FOXR1	0.178657	0.334284	0.449683	0.215813	0.327647
FOXR2	-1.00284	-0.22323	-0.97757	-0.63371	-0.87692
FOXRED2 EIF3D	0.378131	0.370675	0.242394	0.106236	0.159261
FPGS	-0.30719	-0.16572	-0.22115	-0.20881	-0.31186
FPR1	-0.60447	-1.59568	-1.48912	-1.32251	-1.54452
FPR2	0.221827	-0.7496	-0.61982	-0.40636	-0.55123
FPR3	-0.44968	-0.77898	-0.87994	-0.74823	-0.89204
FRAS1	-0.51042	-0.34453	-0.53726	-0.48185	-0.40484
FRAT1	0.218883	0.043848	0.138169	0.208878	-0.08572
FRAT2	0.186333	0.254769	0.247094	0.191154	0.234915
FREM2	-0.09593	-0.09951	-0.02734	0.361183	0.394285
FREQ	-0.08048	0.059353	0.107811	0.013032	-0.01559
FRG1	0.473477	0.151536	-0.00818	0.449339	0.434655
FRG1B.	0.53159	0.022988	0.251408	0.544145	0.491894

FRG2	-0.04992	-1.63102	-0.70369	-0.30296	-1.05511
FRK	0.600548	0.686846	0.776136	0.309895	0.530889
FRMD1	0.194685	0.139195	0.284119	0.231852	0.397771
FRMD3	-0.04498	0.162717	0.193777	0.160342	0.150126
FRMD4A	0.459843	-0.44276	-0.62447	-0.41124	-0.71251
FRMD5	-0.05122	-0.11287	-0.02932	-0.08003	-0.0833
FRMD6	-0.07996	-0.07112	0.009366	0.011449	-0.03654
FRMD7	-0.12878	0.258075	0.023195	-0.1131	-0.14509
FRMD8	0.125228	0.186614	0.106131	0.102375	0.147911
FRMPD4	0.211302	0.142381	0.344464	0.20979	0.311915
FRS2	0.227153	0.027241	-0.18136	0.17104	-0.05155
FRS3 PRICKLE4	-0.43092	-0.55678	-0.48814	-0.41592	-0.47929
FRY	-0.03862	0.334481	-0.13392	-0.02111	-0.08813
FRZB	0.20598	0.079283	-0.11118	-0.38885	-0.07682
FSCN1	-0.03967	0.110482	0.114854	0.062264	0.1564
FSCN2	0.309386	-0.07611	0.652277	0.364988	0.464797
FSCN3	0.386243	0.379451	0.194707	0.239741	0.293312
FSD1L	0.30882	0.17812	0.167849	0.06901	0.070328
FSHB	-0.58596	-0.87008	-0.8451	-0.70542	-0.5934
FSHR	0.098828	0.17438	0.283973	0.028112	0.076025
FSIP1	-0.54728	-0.70388	-0.59774	-0.187	-0.71033
FSIP2.	-0.40042	-0.41031	-0.48976	-0.40856	-0.31595
FST	-0.38824	-0.33332	-0.43644	-0.32675	-0.07366
FSTL1	-0.12744	0.22233	-0.11673	0.098137	0.150841
FSTL3	-0.21816	-0.39821	-0.08289	0.140104	-0.05096
FSTL4	0.08686	0.112736	0.353132	0.10895	0.221407
FSTL5	-0.07176	-0.3333	-0.14867	-0.21046	-0.10307
FTCD	0.337063	0.343395	0.486463	0.395522	0.437234
FTH1	0.116452	0.185006	0.14195	0.189111	0.22658
FTHL17	0.363203	0.57545	0.603446	0.400962	0.312553
FTL	0.093363	-0.03645	0.10871	0.175755	0.163721
FTMT	0.587076	0.705918	0.78709	0.487559	0.627841
FTS	-0.02885	-0.00957	-0.0836	-0.06728	-0.00799
FTSJ1	0.218645	-0.00591	0.333982	-0.05674	-0.05494
FTSJ2 NUDT1	-0.01027	-0.09338	-0.22503	-0.08818	-0.02809
FTSJ3 PSMC5	-0.61974	-0.89778	-1.08922	-0.96459	-0.72174
FUBP1	-1.16177	-1.17299	-1.18659	-1.16929	-0.6574
FUCA1	0.244134	0.090998	0.223518	0.320083	-0.05819
FUCA2	0.346656	0.254309	0.490513	0.47894	0.331147
FUK	-0.18214	-0.18955	-0.26427	-0.10611	-0.17139
FUNDC1	-0.10304	-0.08072	-0.27242	-0.08873	-0.01067
FUNDC2	0.107087	0.100885	0.455106	0.062816	0.164263
FURIN	0.471828	0.664126	0.756265	0.379826	0.589785
FUSIP1	-0.04109	0.189285	0.130616	0.1211	0.220513
FUT10	-0.02538	0.116772	-0.01195	0.078834	-0.1339
FUT2	0.023443	0.090311	0.29848	0.179744	0.511612
FUT3	0.529782	-0.54509	0.697963	-0.47968	0.392702
FUT4	0.226583	0.208091	0.432897	0.199424	0.317324
FUT5	0.354143	-0.22493	0.467618	-0.15148	0.233235
FUT5 NDUFA11 AC104532.2	-0.33228	-0.42589	-0.58113	-0.3007	-0.49405

FUT6	0.296882	-0.18713	0.078146	-0.00057	0.356617
FUT9	-0.45878	-0.25354	-0.40919	-0.4116	-0.50287
FUZ	-0.27293	-0.40065	-0.66936	-0.38721	-0.47139
FVT1	-0.03263	-0.22929	-0.08808	-0.11408	-0.06361
FXN	0.199635	0.294549	0.280777	0.163741	0.070981
FXR1	-0.58804	-0.27936	-0.58133	-0.35361	-0.30709
FXR2	-0.15359	0.095479	-0.0016	-0.02728	0.011613
FXYD1 FXYD5	-0.50424	-0.44777	-0.20656	0.263782	-0.27292
FXYD3	0.334734	0.283917	0.374112	0.118387	0.386413
FXYD4	0.307219	0.297944	0.632139	0.157934	0.284712
FXYD6	-0.33668	0.017881	-0.39101	-0.24015	-0.37111
FYB	0.127409	-0.78931	-0.76627	-0.5446	-0.46895
FYTTD1	0.094506	-0.013	-0.22197	-0.21694	-0.02467
FZD1	-0.40383	-0.3107	-0.50595	-0.24218	-0.39703
FZD10	-0.11715	0.030517	0.124247	0.111336	0.131631
FZD2	-0.22699	-0.04054	-0.09549	-0.22289	0.071457
FZD3	0.051344	0.253751	0.093411	0.105281	-0.05771
FZD4	0.046183	0.206007	0.181007	-0.00451	0.161404
FZD5	-0.10806	-0.12067	-0.09419	-0.15019	-0.12923
FZD7	-0.05651	0.090634	0.191165	0.090077	0.301981
FZD8	-0.08036	-0.04099	0.1147	-0.00545	0.005535
FZD9	0.301605	0.280717	0.361529	0.338939	0.442271
G0S2	-0.0134	0.197144	0.04511	0.337744	-0.09894
G3BP1	-0.5129	-0.35586	0.02219	-0.37993	-0.40739
G3BP2	-0.47297	-0.32721	-0.50242	-0.35854	-0.39109
G6PC2	0.328127	0.254157	0.305348	-0.1673	-0.02484
G6PC3	-0.02946	-0.28591	-0.13363	-0.3336	-0.15436
G6PD IKBKG	0.215015	0.045347	0.194684	0.057414	0.032958
GAA	0.056094	0.252446	0.306438	0.038089	0.204242
GAB3	0.168754	0.37712	0.192409	0.206448	0.065418
GAB4	0.380881	0.74965	0.454939	0.420315	0.598231
GABARAP	-0.80472	-0.38936	-0.94001	-0.67858	-0.44053
GABARAPL2	0.181624	-0.30485	-0.58788	-0.29801	-0.56712
GABARAPL3.	-0.02946	0.13489	0.019785	0.406293	0.09031
GABBR2	0.114725	0.124707	0.261722	0.186201	0.149618
GABPB2	-0.62052	-0.58432	-0.62222	-0.41751	-0.44282
GABRA1	-0.10553	-1.58092	-1.26184	0.161291	0.194582
GABRA2	-0.68643	-0.38834	0.132161	-0.58088	-0.37232
GABRA3	-0.25275	-1.00764	-0.17797	-1.23667	-1.62533
GABRA4	-0.35708	0.406724	0.551944	0.443993	0.082001
GABRA6	-0.19345	-1.14962	-0.66104	0.063986	-0.88757
GABRB1	0.159138	0.078912	-0.41307	-0.01062	-1.3729
GABRB2	-0.29557	0.148059	0.041621	0.084961	0.287101
GABRB3	-0.00327	0.172975	0.140462	0.115113	0.200083
GABRD	0.167942	0.030673	0.2862	0.217624	0.198715
GABRE	0.190241	-0.04113	0.084149	0.040126	0.25859
GABRG1	-0.34513	-0.50014	-0.35182	-0.24258	-0.36771
GABRG2	-1.33981	0.077258	-1.12537	-0.22525	-0.68712
GABRP	0.181888	0.157555	0.399665	0.02309	0.130046
GABRQ	0.329737	-0.05666	0.312122	0.204609	0.308755

GABRR1	0.723191	0.7818	0.982366	0.506346	0.461395
GABRR2	0.184813	0.495441	0.410373	0.167559	0.255678
GAD1	-0.74868	-0.11325	-0.43571	-0.2203	0.148949
GAD2	0.064735	0.177628	0.269796	0.40193	0.313976
GADD45A	-0.31386	-0.45453	-0.32127	-0.37385	-0.35911
GADD45B	-0.50568	-0.3715	-0.21019	0.456402	-0.24553
GADD45G	-0.2669	-0.25818	-0.29838	-0.19499	-0.26853
GADD45GIP1	0.316757	-0.00233	0.149978	0.11348	0.03544
GADL1	0.568067	0.30092	0.699502	0.262127	0.075539
GAGE2A	0.733818	0.969178	0.929549	1.038468	1.327929
GAK TMEM175	-0.08429	-0.17888	-0.21997	-0.03721	-0.2036
GAL	0.126892	0.291414	0.218498	0.198772	0.348382
GAL3ST1	0.164051	0.286481	0.290624	0.280198	0.04919
GAL3ST2	0.42029	0.494685	0.84917	0.457332	0.467344
GAL3ST3	0.012747	0.253685	-0.32779	-0.19493	0.111102
GAL3ST4	0.472506	0.078128	0.670017	0.338281	0.499592
GALC	0.116334	0.29062	-0.01375	-0.06087	0.054483
GALE	0.049875	0.033819	-0.04133	0.25051	0.219621
GALK1	-0.02274	0.017602	0.038853	-0.02211	0.000722
GALK2	0.2455	0.361637	0.260794	-0.06214	0.213813
GALM	0.290468	0.345984	-0.01054	-0.0004	-0.21129
GALNS TRAPPC2L	0.053235	-0.07193	-0.0961	0.053292	-0.20335
GALNT1	-0.08665	-0.23695	-0.49846	-0.4779	-0.23228
GALNT12	0.222626	0.490748	0.312746	0.084349	0.235534
GALNT13	-0.00539	0.372918	0.334639	0.125313	0.075734
GALNT14	0.197112	0.270233	0.149012	0.063669	0.068699
GALNT2	-0.07981	-0.26464	-0.16507	-0.26922	-0.18527
GALNT3	0.058234	0.061977	-0.14056	-0.21944	-0.0822
GALNT5	-0.19722	0.524945	-0.78814	-0.36388	-0.05602
GALNT7	-0.25843	-0.26584	-0.20604	-0.27169	-0.21026
GALNT8	0.289055	0.233793	0.355773	0.212538	0.038518
GALNT9	0.18014	0.087127	0.256278	0.137359	0.207709
GALNTL1	-0.00105	0.168171	0.147695	0.109423	0.172172
GALNTL2	-0.46132	-0.19847	-0.86926	-0.62637	-0.82524
GALNTL4	-0.16841	-0.28317	-0.31549	-0.04901	0.061564
GALNTL5	0.527613	0.421702	0.73961	0.301356	0.412721
GALP	0.363869	-1.30532	0.4237	0.136215	0.449612
GALR1	-0.20337	0.186346	0.270916	0.209647	-0.0755
GALR3	0.119813	-0.08412	0.300225	0.204533	0.405505
GALT	-0.04263	0.047981	0.211873	-0.00659	-0.07326
GAMT	0.170394	0.132055	0.156024	0.153951	0.289562
GAN	-0.35119	-0.11578	-0.21303	-0.11743	-0.26015
GANAB INTS5	0.035062	0.029724	0.141926	-0.00787	0.062034
GANC	-0.96113	-0.02016	-0.21843	-0.40265	-0.69603
GAP43	-1.21248	-0.49017	-1.92794	-1.1752	-1.86457
GAPDH	-0.32248	-0.27552	-0.26065	-0.23402	-0.17749
GAPDHS	0.374159	-0.15349	0.394802	0.351865	0.320423
GAPDHS TMEM147	-0.07705	0.052951	-0.09182	0.206548	-0.09227
GAPVD1	0.03837	0.136948	0.200286	0.100577	-0.12571
GARNL1	0.034218	0.169445	0.206428	-0.04723	-0.01082

GARNL3	0.329915	0.15916	-1.02964	-0.74998	-0.99025
GARNL4	0.410533	0.402583	0.47033	0.471486	0.476361
GARS	-0.54035	-0.48596	-0.81973	-0.39386	-0.5414
GART SON	-0.6764	-0.79902	-0.81004	-0.47151	-0.76146
GAS1	-0.22363	-0.06758	-0.15234	-0.04973	-0.1027
GAS2L1	0.018489	0.095801	0.110561	0.003411	0.137804
GAS2L2	0.282228	0.390275	0.566111	0.187404	0.301469
GAST	0.195794	0.349834	0.174815	0.138457	0.362758
GATA1	0.428756	0.336382	0.444552	-0.09925	0.297869
GATA2	-0.02008	-0.21434	-0.2091	0.167875	-0.0782
GATA4	-0.10822	-0.07594	-0.30399	0.148988	0.070533
GATA5	0.118812	0.172034	0.261563	0.27758	0.145101
GATA6	-0.40102	-0.32746	0.02115	0.424646	-0.06443
GATAD1	0.087208	0.18359	0.117854	0.009305	0.13915
GATAD2A	0.630632	0.867506	0.870581	0.431877	0.752592
GATAD2B	0.257002	0.537433	0.771131	0.241131	0.383958
GBA2 RGP1	-0.09636	-0.36609	-0.2934	-0.10204	0.019342
GBA3	-0.84869	-0.78388	-0.72645	-0.64861	-1.11092
GBAS	0.205591	0.139953	0.258285	0.146803	0.235838
GBE1	0.110396	-0.08548	0.021632	-0.08617	-0.01007
GBF1	-0.37324	-0.12687	-0.86748	-0.52899	-0.65315
GBGT1	0.351804	0.384609	0.53458	0.324989	0.464614
GBL	0.185647	0.200903	0.221891	0.064374	0.248172
GBP1	-0.09296	-0.13503	-0.03049	-0.43785	-0.23256
GBP2	0.296113	-0.38703	0.102106	-0.15472	-0.34451
GBP3	-0.53225	-0.12247	-0.12197	-0.45329	0.043772
GBP4	0.211327	-0.91465	0.163853	0.277578	0.380245
GBP6	-0.06208	0.199989	0.116336	-0.15145	0.019944
GBP7	-0.21088	0.012382	-0.57937	-0.69853	-0.25156
GBX2	0.046301	0.044977	0.216818	0.147145	0.22922
GC	-0.91041	-0.96397	-0.79221	-0.86758	-1.15716
GCA	0.194952	-0.05734	-0.37205	0.027416	-0.07886
GCC1	0.374454	0.452441	0.504579	0.384167	0.446585
GCC2	-0.25684	-0.04177	-0.25555	-0.0622	-0.09565
GCDH	-0.34148	-0.59116	-0.58563	-0.28639	-0.31802
GCET2	0.417852	0.510374	0.595554	0.039323	0.030141
GCG	0.408896	-0.33323	0.601156	-0.07373	-0.0083
GCH1	0.194172	0.201067	0.297346	0.215003	0.303081
GCHFR	0.299072	0.436603	0.601139	0.400114	0.463305
GCLC	0.179428	0.033278	-0.04079	-0.0386	-0.1752
GCLM	0.062675	0.283772	0.212136	0.101427	0.224087
GCM1	0.437737	0.704342	0.830797	0.403909	0.318278
GCM2	-0.00961	0.114393	0.455564	0.159839	0.213932
GCN1L1	0.020807	-0.10016	0.076648	0.065053	0.01872
GCN5L2 HSPB9	0.351417	0.368718	0.569692	0.311498	0.356537
GCNT1	0.076889	-0.09367	0.307647	-0.01688	0.021099
GCNT3	0.435778	0.40551	0.370904	-0.39205	-0.41679
GCNT4	0.430682	0.02591	0.450746	-0.17827	-0.18536
GCNT7	0.719426	0.8892	0.815442	0.548035	0.739834
GCSH	-0.01654	0.030083	0.039761	0.044175	-0.12874

GDA	0.337348	0.445477	0.591564	0.265747	0.325726
GDAP1	-0.16216	-0.3595	-0.08306	-0.34285	-0.27939
GDAP1L1	-0.23548	-0.09499	-0.27268	-0.32505	-0.26224
GDAP2 WDR3	-0.76204	-0.92746	-0.83019	-1.0153	-0.72953
GDE1 AC012621.8	-0.08368	0.024085	-0.10102	-0.18299	-0.17076
GDF1	0.10747	0.060396	0.286113	0.156819	0.258644
GDF10	0.103696	0.277399	0.331555	0.095996	0.20816
GDF11	-0.35695	-0.45759	-0.28483	-0.30707	-0.41752
GDF2	0.258749	0.302794	0.270452	0.24921	0.335438
GDF3	0.220494	-0.50928	-0.55129	-0.12244	0.047611
GDF5	0.537105	0.501702	0.565869	0.352654	0.369136
GDF5 CEP250	0.131269	-0.02359	0.035497	0.123607	0.046324
GDF6	-0.08862	-0.05751	-0.0017	-0.06006	-0.07376
GDF7	0.220342	0.033055	0.099038	0.232868	0.007518
GDF8	0.012537	0.063538	-0.13966	0.148961	-0.31136
GDF9 UQCRQ	0.282722	0.462138	0.338125	0.163049	0.232434
GDI1 FAM50A	-0.01261	0.091101	-0.03748	0.020534	0.125195
GDI2	-0.12267	-0.17425	-0.17659	-0.22972	-0.10086
GDPD1	0.040925	0.036294	0.109838	-0.16912	-0.10576
GDPD2	0.005425	0.199933	0.286471	0.086893	0.060261
GDPD4	-0.62069	-0.21645	-0.69827	-0.79103	-0.81047
GDPD5	-0.01259	0.087975	-0.099	0.006947	0.006347
GEM	0.248009	-0.06014	-0.36699	0.083902	0.073017
GEMIN4 AC087392.10	0.136178	0.139787	0.260545	0.105107	0.323252
GEMIN5	0.237358	0.28944	0.431754	0.190877	0.217042
GEMIN6	0.341826	0.225467	0.363731	0.050066	0.388168
GEMIN7	0.105592	0.257005	0.212043	0.059233	0.316547
GEMIN8	0.231605	0.020774	-0.22629	-0.10189	-0.10459
GFAP	-0.00485	0.243758	0.164352	0.330772	0.318654
GFI1	-0.0939	-0.00853	0.162151	0.239139	-0.1082
GFI1B	0.225783	-0.76746	0.361706	-0.03432	-0.15143
GFM2 TINP1	-0.26129	-0.32276	-0.18825	-0.06521	-0.39426
GFOD1	-0.35842	-0.1529	-0.13153	-0.39582	-0.35727
GFOD2	-0.85278	-0.96004	-1.01619	-0.80312	-1.01324
GFPT1	0.2413	0.337674	0.229991	0.138304	0.151322
GFPT2	0.082429	0.19071	0.09313	0.126795	0.12758
GFRA1	-0.46939	-0.14274	-0.21776	-0.45824	-0.11076
GFRA2	0.395306	0.525715	0.699235	0.531567	0.546285
GFRA3	0.009001	0.150523	-0.331	-0.00329	-0.49184
GFRA4	0.249693	0.03483	0.708616	0.477397	0.564708
GFRAL	-0.42071	-1.20897	-0.15881	-0.19677	-0.77152
GGA1	-0.23344	-0.32558	-0.55002	0.13315	-0.07481
GGA2	0.104661	-0.02994	0.100619	-0.0567	-0.03923
GGA3 MRPS7	-0.49841	-0.65965	-0.75291	-0.62325	-0.44622
GGCX	0.075855	-0.15166	-0.08537	0.012267	0.212312
GGH	-0.27487	-0.22795	-0.49794	-0.3815	-0.25268
GGN SPRED3	-0.23878	-0.42234	-0.58593	-0.04058	-0.40356
GGT6	0.371832	0.350992	0.679329	0.491624	0.660983
GGTL3 ACSS2	-0.00922	-0.07671	0.057668	-0.21631	-0.13608
GGTLA1	0.312355	0.040309	-0.05858	-0.76581	0.193853

GGTLA4	0.537414	0.546698	0.589033	0.468135	0.688502
GGTLC2	0.430986	0.365862	0.366448	0.327125	0.437294
GH1.	0.396247	0.566179	0.547473	0.624375	0.544962
GHDC	0.27186	-0.14117	-0.00363	-0.11949	-0.0442
GHITM	0.24899	0.226849	0.127536	0.121343	0.065297
GHR	-0.29207	-0.04345	-0.20199	-0.01903	-0.14723
GHRH	0.052504	0.432855	0.302489	-0.04663	0.206302
GHRHR	0.06098	-0.13069	0.037708	0.068577	0.307914
GHRL	0.402802	0.550793	0.509554	0.614472	0.43088
GHSR	0.19472	0.153018	0.683948	0.355346	0.475881
GIF	-0.35748	-0.90549	-0.6208	-0.60361	-0.15397
GIGYF1	0.308962	0.50399	0.530735	0.325586	0.411909
GIMAP1	-0.02406	-1.02146	-1.29764	-0.37815	-0.88678
GIMAP2	-0.43305	-0.091	-1.44633	-0.57866	-0.95991
GIMAP4	-0.13928	-1.72968	-2.14244	-0.52781	-1.51868
GIMAP5	-0.0381	-0.4448	-0.90092	-0.23427	-0.62329
GIMAP6	0.184774	-0.13999	-0.9109	-0.04372	0.089252
GIMAP7	0.21594	-0.14059	-0.93124	-0.25132	-0.54021
GIMAP8	0.452227	0.33389	0.562495	0.135494	0.375922
GIN1 HISPPD1	-0.58165	-0.66458	-0.57123	-0.42916	-0.56938
GINS2	0.204926	0.46597	0.362186	0.211675	0.466684
GINS3	0.309224	0.100702	-0.042	-0.11895	0.104002
GINS4	0.176483	0.028069	0.186759	-0.12978	0.010592
GIP	0.398779	-0.3188	0.10249	0.254496	0.229199
GIPC1	0.09044	0.314328	-0.42056	0.08353	0.149235
GIPC2	0.356383	0.614398	0.743784	0.355404	0.509104
GIPR	-0.00264	-0.00049	-0.07136	-0.24696	-0.27254
GIT1	0.026002	0.021785	-0.02398	-0.05307	0.124661
GIT2	-0.10968	-0.15158	-0.20971	-0.19995	0.006349
GJA1	-0.92976	-0.00942	-0.19353	-0.10962	-0.27923
GJA10	0.138687	-0.16379	0.332423	-0.12558	-0.00661
GJA3	0.379452	0.102854	0.405492	0.328841	0.386533
GJA4	0.369749	0.461484	0.679199	0.36306	0.431776
GJA8	0.134815	-0.5084	-0.32889	-0.87146	0.091421
GJA9	-0.71831	-0.48982	-0.81724	-0.51808	-0.52637
GJB1	0.56971	0.610286	0.468752	0.439065	0.531032
GJB2	-0.21653	-0.1905	0.26355	0.335081	0.200722
GJB3	0.090897	0.157289	0.294561	0.165574	0.165584
GJB4	0.31023	0.400696	0.422995	0.228445	0.265891
GJB5	0.044876	0.204258	0.241336	0.184782	0.175319
GJB6	-0.12315	-1.00747	-0.14037	0.404822	0.20901
GJC1	-0.04497	0.021481	-0.19646	-0.20234	-0.03303
GJC2	-0.03235	0.069568	0.077998	0.079329	0.021356
GJC3	0.030282	-0.10693	-0.4716	-0.33408	-0.05714
GJD2	0.093406	0.37883	0.292096	0.287311	0.208923
GJD4	0.458245	0.466937	0.733152	0.376993	0.378583
GK	0.090029	0.192494	-0.02033	-0.00645	-0.12825
GK2	0.564652	0.68882	0.833998	0.354626	0.561384
GK5	0.237096	0.239582	0.150926	0.045478	0.122346
GKAP1	-0.4077	-0.1881	-0.17692	-0.27531	-0.28854

GKN1	-0.16235	0.08997	-0.06854	-0.45137	-0.53369
GKN2	0.206541	0.473125	0.253889	-0.19875	-0.11187
GLA HNRPH2	-0.00512	-0.43123	-0.90154	-0.40276	-0.39273
GLB1	0.093698	0.285447	0.043053	0.135429	0.141898
GLB1L STK16	0.121115	0.085582	0.230754	0.049316	0.116725
GLCCI1	0.118843	-0.27137	-0.16327	-0.1584	-0.10873
GLCE	-0.73371	-0.48754	-0.95435	-0.04834	-0.80973
GLDC	-0.08351	0.259739	0.125149	-0.05213	0.171119
GLE1	0.060191	-0.02809	0.043138	0.020788	-0.13038
GLG1	-0.11422	-0.04027	-0.13137	0.002251	-0.04301
GLI1	-0.95249	-0.73282	-1.0886	-0.77701	-1.15538
GLIPR1	0.140219	0.538846	0.336097	0.200265	-0.41791
GLIS1	0.551381	0.879037	1.114324	0.67462	0.676863
GLIS2	0.24244	0.394077	0.329862	0.228635	0.344192
GLIS3	0.156228	0.134182	-0.17003	-0.25809	-0.58732
GLMN RPAP2	-0.17489	-0.04664	-0.09993	-0.19899	0.035569
GLO1	0.343219	0.516189	0.552096	0.308202	0.347948
GLOD4	0.200037	0.172707	0.115848	0.035932	0.03222
GLP1R	0.017074	0.349756	0.20712	0.209323	0.221711
GLP2R	0.357416	-0.30172	-0.43243	-0.25939	0.270092
GLRA1	-0.29655	-1.01539	-0.56773	-0.8133	-0.82667
GLRA2	0.150164	-0.82548	-0.91307	-0.55766	-0.65301
GLRA3	-0.27518	-0.60309	-0.68453	-0.11744	-0.47591
GLRA4	0.033715	0.438927	0.127454	0.041894	0.348798
GLRB	0.279115	0.106432	0.022777	0.07365	0.242785
GLRX	-0.5505	-0.30787	-0.37303	-0.33562	-0.34521
GLRX2	0.089298	-0.07452	-0.10513	-0.28897	-0.15632
GLRX5	-0.14571	-0.22004	-0.17695	-0.14185	-0.17797
GLS	0.145807	-0.04945	-0.15188	0.045625	0.134384
GLS2	0.361504	0.631241	0.436047	0.428594	0.48745
GLT1D1	0.296962	0.428109	0.374526	0.27199	0.493926
GLT25D1	0.15315	0.313633	0.286553	0.237978	1.64361
GLT25D2	0.499203	0.600928	0.550442	0.290796	0.444594
GLT6D1	0.67093	-0.49243	0.627854	0.45611	0.445092
GLT8D1 SPCS1	0.142992	0.191007	0.095139	-0.01378	0.120993
GLT8D2	0.211655	0.188639	-0.09277	0.64721	0.231997
GLTPD2	0.281544	0.279187	0.499725	0.338876	0.272631
GLTPP1	-0.14428	-0.01715	-0.03703	-0.22161	-0.14129
GLTSCR2	0.171932	0.196036	-0.09847	0.087758	-0.00571
GLUD1 FAM35B.	0.039604	0.051982	0.178113	0.042095	0.068022
GLUD2	0.035594	0.134555	0.419367	0.190416	0.204222
GLUL	-0.11243	-0.06447	-0.17468	-0.05969	-0.04452
GLULD1	-0.22658	-0.70372	-0.89695	-0.73152	-0.76664
GLYAT	-0.47979	-0.6145	-1.00061	-0.78799	-0.12327
GLYATL1	-0.35976	-0.7324	-0.46163	-0.69938	-0.42491
GLYATL2	-0.09885	-0.6416	-0.43372	-0.63511	-0.47008
GLYCTK	0.058132	-0.01383	-0.16578	0.005795	0.023468
GM2A	-0.60324	-0.68978	-1.41049	-0.59913	-0.86719
GMCL1L.	0.015922	0.022024	-0.19564	-0.05423	-0.05416
GMDS	0.011083	0.082925	0.025873	0.067354	0.054752

GMEB1	0.087958	0.180329	0.122657	-0.01392	-0.12473
GMEB2	0.322295	0.385039	0.483635	0.232145	0.41517
GMFB	0.099533	0.069601	0.000589	-0.02803	-0.13038
GMFG	0.371268	-0.21297	0.636717	-0.03773	0.480385
GMIP	-0.13006	-0.04747	-0.32406	-0.17834	-0.28792
GML	0.185193	0.297646	0.485925	0.275327	0.397324
GMNN	0.033519	0.215193	0.177939	0.067533	0.089418
GMPPA	-0.17778	-0.0128	-0.42915	0.073084	-0.26306
GMPR	0.015634	0.265978	0.277658	0.272053	0.295593
GMPS	-0.31944	-0.03298	-0.19521	-0.19941	-0.1795
GNA11	0.177516	0.036026	0.009743	0.071474	0.201565
GNA12	0.193729	0.108256	-0.08199	0.049152	0.161837
GNA13	-0.02604	-0.03435	-0.07412	-0.07301	-0.0069
GNA14	0.300944	0.235364	0.624052	0.461751	0.640694
GNA15	0.179666	0.522601	0.337422	0.302193	0.242527
GNAI1	0.151047	0.170355	0.084692	0.080527	0.066798
GNAI2	0.105867	0.150268	0.204633	0.073383	0.144208
GNAI3	-0.79715	-1.12576	-1.23866	-0.88736	-1.03222
GNAO1	-0.70632	-0.41389	-0.76837	-0.48367	-0.5898
GNAQ	-0.10202	-0.05347	-0.10206	-0.03457	-0.00582
GNAT1	0.214068	0.149533	0.157924	-0.05892	0.083787
GNAT2	0.477525	0.408855	0.413777	0.269846	0.523547
GNB1	-0.03309	-0.02318	0.106384	-0.02987	0.149923
GNB1L	-0.1072	-0.01967	-0.03854	-0.07096	0.09504
GNB2	-0.02386	-0.02586	-0.14018	-0.16484	-0.11418
GNB2L1	-0.58733	-0.48847	-0.76175	-0.52852	-0.63373
GNB4	-0.01618	-0.23384	0.563338	-0.08184	0.543547
GNE	0.24138	0.097486	-0.16389	0.102044	0.380904
GNG11	0.561444	-0.49006	-1.07604	-0.04077	0.694797
GNG12	0.177572	0.33741	0.365421	0.140566	0.247561
GNG13	-0.11544	-0.10587	-0.29938	0.025598	-0.09493
GNG2	0.356378	-0.18476	-0.01267	0.157329	-0.0446
GNG4	0.278353	0.77408	0.736085	0.441658	0.462144
GNG5 SPATA1	-0.14854	-0.23637	-0.2941	-0.13096	-0.01014
GNG8	-0.54603	-0.76009	-0.71112	-0.27092	-0.64996
GNGT1	0.066398	0.250661	0.176221	-0.48178	-0.41375
GNGT2 ABI3	0.347419	-0.47806	-0.74081	-0.23423	0.501614
GNL1 PRR3	-0.754	-0.68877	-0.97752	-0.60386	-0.87848
GNL2	-0.70395	-0.56733	-0.71719	-0.75157	-0.38092
GNL3L	-0.00066	-0.14647	0.061498	-0.32199	-0.1592
GNLY	0.073406	0.211741	0.303182	0.103695	0.286395
GNMT	0.333019	0.456414	0.416139	0.373096	0.372294
GNPDA1	0.203629	0.203716	0.314857	0.036331	0.022353
GNPDA2	0.190439	-0.10457	-0.10454	-0.22333	-0.14762
GNPNAT1	0.041101	0.024816	-0.0664	-0.06258	-0.02495
GNPTAB	-0.10253	-0.26601	-0.18234	-0.24775	-0.29014
GNRH2	0.035493	0.164579	0.001941	-0.03192	0.035779
GNRH2 MRPS26	0.071076	-0.00295	-0.14971	-0.02025	-0.00315
GNRHR	0.057909	0.045061	0.210662	-0.28793	0.027766
GNRHR2 PEX11B	-0.40668	-0.54937	-0.89308	-0.09259	-0.41509

GNS	0.271564	0.10618	-0.195	0.036429	-0.07912
GOLGA1	-0.07063	-0.04222	-0.02737	0.059721	0.101631
GOLGA2 C9orf119	-0.10878	0.00602	-0.14623	0.037677	-0.11504
GOLGA2L1	0.577094	0.606656	0.733517	0.333831	0.328542
GOLGA3	0.212983	-0.27822	-0.11311	0.063292	-0.03568
GOLGA5	-0.13973	0.19772	-0.25083	-0.32892	-0.24468
GOLGA6	0.48711	0.55048	0.427652	-0.04615	-0.34105
GOLGA6B	0.482316	0.39028	0.564862	0.167292	0.099429
GOLGA7	0.108149	0.307912	0.107926	0.05056	-0.03826
GOLGA8A	0.527962	0.727174	0.516887	0.446516	0.392639
GOLGA8B	0.331175	0.532415	0.605776	0.173896	0.354063
GOLGA8E	0.518098	-0.26147	-0.06947	-0.01015	0.155556
GOLGA8G	0.257491	-0.26305	-0.28876	-0.03419	0.268364
GOLGB1	-1.39545	-1.24915	-1.39656	-1.03434	-1.04606
GOLIM4	-0.18387	-0.10921	-0.11813	-0.17112	-0.05565
GOLM1	0.086383	0.367881	0.367206	0.170533	0.308342
GOLPH3	-0.15193	0.544337	0.091172	0.033129	0.242112
GOLPH3L	-0.80807	-0.96159	-1.44601	-1.31095	-1.04025
GON4L SYT11	0.10443	-0.30314	-0.41141	-0.24549	-0.38475
GOPC	0.194683	0.283053	-0.11693	0.179832	0.390161
GORASP1 TTC21A	-0.2198	-0.04405	-0.07981	-0.16227	-0.00988
GORASP2	0.099496	0.245193	0.345512	0.248998	0.322269
GOSR1	-0.14653	-0.22303	-1.38449	-0.39066	-0.9725
GOSR2	-0.2324	-0.9625	-0.85724	-0.81478	-0.88813
GOT1	-0.40112	-0.17371	-0.28409	-0.19004	-0.40135
GOT1L1	0.105335	0.585866	0.261416	0.115879	0.073624
GOT2	-0.02966	0.187903	0.041462	0.082076	-0.07175
GP2	-0.10901	-0.64142	-0.74194	-0.26446	-0.00567
GP5	0.557445	0.521001	0.606143	0.247694	0.302151
GP6	0.642253	-0.17202	0.422941	0.360616	0.29955
GP9	0.306559	0.56979	0.489067	0.399944	0.578437
GPA33	0.017436	-0.09206	-0.24983	-0.40797	-0.44467
GPAA1	-0.13933	-0.01756	-0.07943	-0.0015	-0.10285
GPAM	-0.40834	-0.1192	-0.24296	-0.22528	-0.30738
GPATCH1	-0.03188	0.098355	-0.17667	0.054307	0.029827
GPATCH2 SPATA17	-0.0982	-0.14559	-0.28178	-0.23333	-0.38324
GPATCH3	0.088701	0.160346	0.12196	0.182509	0.453059
GPATCH4	0.161153	0.288631	0.13479	0.172365	-0.01654
GPATCH8	-0.03355	-0.06954	-0.12103	-0.06621	0.003211
GPBP1	-0.63283	-0.61192	-0.50131	-0.45219	-0.53945
GPBP1L1	0.222344	0.260716	0.023659	-0.00346	-0.05079
GPC1	-0.06137	-0.1296	-0.06923	-0.05209	-0.14206
GPC2 STAG3	-0.00494	0.177953	0.245619	0.039029	0.448874
GPC3	-0.12412	-0.13602	-0.01529	-0.01072	0.112061
GPC4	0.121099	0.038065	0.303837	0.155735	0.200927
GPC5	-0.0092	0.149635	0.051758	0.161583	0.076392
GPD1	9.65E-05	0.132663	-0.35231	-0.25591	-0.20164
GPD1L	0.032614	0.045662	0.026861	0.217502	0.231591
GPHA2	0.582017	0.454655	0.915965	0.22919	0.481546
GPHB5.	0.358808	0.344619	0.392458	0.121063	-0.00171

GPHN	-0.12559	-0.01312	-0.19658	-0.11426	-0.06711
GPI	0.160909	0.336029	0.235749	0.194268	0.20507
GPIHBP1	0.175603	0.132494	0.04957	0.126889	0.104826
GPKOW	0.31769	0.271118	-0.17353	0.096312	0.073532
GPLD1	0.360701	0.545267	0.465159	0.292949	0.232051
GPN3 C12orf24	-0.44962	-0.47596	-0.55303	-0.43196	-0.59988
GPNMB	0.481894	0.37248	0.613385	0.186107	0.201875
GPR1	-0.19012	0.264325	0.199873	-0.06568	0.301068
GPR101	-0.03124	-0.34915	0.225099	-0.07736	-0.04572
GPR103	0.083406	0.239895	0.102512	0.139955	0.501874
GPR107	0.127799	0.114985	0.117988	0.040441	0.000613
GPR110	-0.02037	-0.54946	-0.74867	-0.75646	-0.85808
GPR111	-0.25778	-0.56175	-0.49978	-0.31498	-0.61504
GPR114	0.1563	0.262401	0.225656	0.143056	0.306711
GPR116	0.216636	-0.50123	-0.75289	-0.62209	-0.407
GPR119	0.133817	0.200092	-0.11973	0.227421	0.178643
GPR12	0.309354	0.484626	0.105449	0.195252	0.22731
GPR120	0.04316	0.077337	0.236197	0.334185	0.232357
GPR123	0.408218	0.365508	0.567701	0.38883	0.318066
GPR124	-0.02895	-0.04074	-0.00128	0.013084	0.111918
GPR125	0.108221	0.231082	0.21336	0.066867	0.13416
GPR126	-0.18633	-0.13658	0.170662	0.045057	0.12131
GPR128	0.287047	0.031274	0.245292	-0.20183	-0.91625
GPR132	0.033302	0.349482	0.548212	0.235477	0.262305
GPR133	0.304147	0.54682	0.713847	0.417196	0.725854
GPR135	0.344735	0.216014	0.469389	0.178056	0.280946
GPR137B	-0.14209	-0.10166	-0.17116	-0.21114	0.09032
GPR139	-0.05317	0.13019	-0.03871	-0.01975	-0.31192
GPR141	0.206109	0.497636	0.463546	-0.00036	0.034089
GPR143	0.229761	0.316071	0.32688	0.278713	0.170865
GPR144	0.178998	-0.03251	0.147322	0.203846	0.304918
GPR148	0.378115	0.253977	0.349602	0.312939	0.292047
GPR149	0.140113	-0.08993	0.337345	0.145526	-0.03305
GPR15	0.145966	0.339983	0.313218	-0.24336	-0.38219
GPR150	0.300646	0.574928	0.645248	0.532841	0.495066
GPR151	0.081354	0.141819	0.049129	-0.07479	0.136352
GPR152 CABP4	0.134058	0.496089	0.345103	0.351169	0.382526
GPR153	0.0351	0.17707	0.101978	0.131375	0.114461
GPR155	0.106169	-0.11208	-0.18176	-0.22154	-0.06627
GPR156	-1.06127	-0.91386	-0.94903	-0.88774	-1.01875
GPR157	0.098732	-0.04455	-0.22016	0.116359	0.119873
GPR158	-0.09842	-0.25061	0.040544	-0.11115	-0.16021
GPR160	0.016847	-0.18412	0.142104	0.125153	0.075342
GPR172B	0.244071	-0.04029	0.171497	0.237778	0.124725
GPR173	-0.0543	-0.1046	-0.22951	-0.0645	-0.05548
GPR174	-0.01778	-0.50755	0.150034	-0.42081	-0.31158
GPR175	0.359356	0.636846	0.473457	0.228871	0.518665
GPR176	0.373331	0.314501	0.366714	0.201758	0.294045
GPR177	-1.10488	-0.51455	-1.05124	-1.18314	-0.52616
GPR179	0.036531	0.146887	0.051547	-0.0377	0.038956

GPR180	0.277101	0.411136	0.42666	0.014758	0.598562
GPR182	0.245813	0.317602	0.392535	0.353924	0.257841
GPR19	0.235654	0.138112	-0.16293	0.052853	0.192862
GPR20	0.373081	0.780341	0.660675	0.515915	0.537258
GPR23	-0.21807	-0.50338	-0.95361	-0.59753	-0.29619
GPR25	0.304888	0.445408	0.647758	0.471936	0.44309
GPR26	0.09545	0.029123	0.419177	-0.04287	0.287319
GPR3	-0.27396	-0.19285	0.266214	0.296485	0.386242
GPR31	0.348341	0.340574	0.637873	0.193291	0.415436
GPR32	0.554125	0.409094	0.944004	0.366244	0.85493
GPR35	0.021676	0.020763	0.287416	0.161128	0.245281
GPR37	-0.38562	-0.23741	-0.67302	-0.29214	-0.32728
GPR37L1	0.203478	0.383059	0.156317	0.225957	0.243187
GPR39	0.225141	0.30375	0.279995	0.309355	0.084598
GPR4	0.412184	0.017015	0.22648	0.451483	0.057424
GPR42	0.479705	0.146713	0.079899	0.300275	0.662708
GPR44	0.425841	0.388105	0.188835	0.094666	0.094836
GPR45	0.580337	0.653247	0.776351	0.477785	0.500965
GPR50	0.247055	0.114539	0.265937	0.288626	0.180272
GPR55	0.15163	0.483066	0.465424	0.300066	0.334581
GPR56	0.065549	0.200043	-0.07622	0.183471	0.23516
GPR6	-0.06704	0.031971	-0.13684	0.102763	0.1037
GPR61	0.065467	0.281593	0.306932	-0.08866	0.168039
GPR62	0.20399	0.083368	0.174917	0.187142	0.198319
GPR63	-0.62525	-0.13302	-0.40167	-0.30229	-0.11429
GPR65	0.211165	0.314497	0.345157	-0.2477	-0.36108
GPR68	0.249804	0.344859	0.114692	0.245235	0.136709
GPR78	0.03814	-0.02626	0.335493	0.137225	0.095693
GPR83	0.20345	0.162498	0.305	0.255919	0.042186
GPR84	0.430899	0.688791	0.507436	0.127586	0.372848
GPR85	-0.00764	-0.60529	-0.88037	-0.25221	-0.72651
GPR88	0.527885	0.415728	0.279809	-0.13062	-0.22802
GPR89B	0.054882	0.176427	0.096694	-0.13968	0.013915
GPR97	0.101511	0.081453	0.202257	0.066243	0.069583
GPR98	-0.21017	-0.19527	-0.08865	-0.01441	0.103002
GPRASP2	0.132589	-0.09087	0.074389	0.015933	0.068514
GPRC5A	0.090588	-0.14223	-0.33111	-0.37419	-0.37664
GPRC5B	-0.01082	-0.09786	0.202731	0.148308	0.112729
GPRC5C	-0.20477	0.123628	0.157901	0.083324	0.099532
GPRC5D	0.235768	0.580335	0.409218	0.292283	0.206326
GPRC6A	-0.40049	-0.43739	-0.48995	-0.64658	-0.89777
GPRIN1	-0.01674	0.030663	0.148972	0.070086	0.154193
GPRIN2	0.436261	0.637105	0.584038	0.377063	0.598952
GPRIN3	0.175859	0.218455	-0.09712	-0.07894	0.146156
GPS2	0.330969	0.221698	0.312191	0.250757	0.269665
GPSM1	0.274219	0.390902	0.305589	0.156473	0.439017
GPSM2	-0.1864	0.025239	-0.142	-0.14642	-0.11589
GPSM3 NOTCH4	-0.28691	-0.04241	-0.18042	-0.17695	-0.0232
GPT2	-0.00779	0.188391	0.108112	0.132898	0.175214
GPX1	0.144646	-0.2148	-0.12034	-0.18133	-0.0828

GPX2	0.477066	0.710694	0.733939	0.459099	0.476428
GPX3	-0.17151	-0.28838	-0.63728	-0.42007	-0.46242
GPX4	0.086435	0.196215	0.122701	0.017696	0.056548
GPX7	0.348593	0.188923	0.362426	-0.04754	0.022989
GRAMD1A	-0.23925	-0.29999	-0.44644	-0.18503	-0.31178
GRAMD1C	0.275244	0.581263	0.493062	0.238572	0.344175
GRAMD2	0.26879	0.365532	0.566528	0.164617	0.433744
GRAMD3	0.325462	0.761082	0.737974	0.517038	0.537987
GRAMD4	0.502253	0.436469	0.359232	0.501197	0.407099
GRAP	0.21008	0.580374	0.505917	0.479775	0.368288
GRAP2	0.027597	0.061991	0.174016	-0.32803	-0.5349
GRASP	0.119155	0.122513	0.134327	-0.13417	-0.11767
GRB14	0.141987	0.428151	0.137341	0.122263	0.08796
GRB2	-0.26333	-0.56383	-0.5339	-0.48162	-0.33883
GRB7	0.266336	0.079381	0.45653	-0.02871	0.10773
GREM1	-0.03143	0.191453	0.271101	0.150505	0.306595
GREM2	0.142834	-0.00148	-0.05636	-0.14418	-0.32081
GRHL2	0.377926	0.657053	0.814604	0.547778	0.518798
GRHPR	0.283234	0.100523	-0.07599	-0.02631	-0.15615
GRIA1	-0.57033	-0.09295	0.301879	0.031071	-1.13487
GRIA2	0.112419	0.212532	0.271129	0.097018	-0.02438
GRIA3	-0.16548	-0.31659	-0.35398	-0.36679	0.242662
GRIA4	-0.5847	-0.34858	-0.67286	-0.45099	-0.79011
GRID1	0.049098	0.010433	0.203708	0.049396	0.056266
GRID2	-1.16545	-1.04051	0.628637	-0.73444	-0.27265
GRIK1	-0.46198	-0.64906	-0.30135	0.043315	-0.11185
GRIK2	0.290493	-1.04674	-0.98327	-0.33376	-1.00195
GRIK3	0.126635	0.021995	0.127444	0.016618	0.152355
GRIK4	0.392008	0.452091	0.595279	0.39763	0.557474
GRIK5	0.32305	0.504569	0.06924	0.034374	0.270594
GRIN1	0.073338	-0.05779	-0.31169	-0.12715	0.040073
GRIN2A	-0.06414	-0.07107	0.220918	0.162482	-0.02797
GRIN2B	-0.20324	0.383	-0.37183	0.522273	0.208188
GRIN2C	-0.14419	-0.33987	0.122218	0.069106	-0.05748
GRIN2D	-0.15604	-0.25217	-0.18476	-0.60065	-0.39091
GRIN3B	0.180069	0.084679	0.307279	0.210544	0.113404
GRINA	0.118933	0.109365	0.078026	0.071297	0.180679
GRIPAP1	0.255816	-0.10533	0.005746	-0.1158	-0.05692
GRK1	0.259762	-0.24229	0.444516	0.288633	0.365156
GRK5	-0.25625	-0.13338	-0.16995	-0.09565	-0.16135
GRK6	-0.36376	-0.26872	-0.43123	-0.18894	-0.33506
GRK7	0.584699	0.524955	0.766118	0.404242	0.65936
GRLF1	0.57788	0.758707	0.623176	0.586055	0.654695
GRM2	0.176096	0.034879	0.093427	-0.00702	0.125317
GRM3	-0.86283	-0.659	-1.18797	-0.23834	-0.74957
GRM4	0.348472	0.333343	0.644186	0.403372	0.664449
GRM5	0.222612	-0.36249	0.042072	-0.22293	0.038011
GRM6	0.096881	0.008684	0.313318	0.320511	0.301804
GRM7	-0.0242	0.222373	0.016234	0.074901	0.252654
GRM8	0.041164	-0.30544	-0.11248	-0.22034	-0.34709

GRN	0.069941	0.172167	0.176392	-0.02551	-0.07851
GRP	-0.51139	-0.33471	-0.5689	-0.187	-0.20992
GRPEL1	0.039719	-0.00175	0.031273	-0.02285	0.106557
GRPEL2	-0.51292	-0.68805	-1.22327	-0.78421	-1.04844
GRPR	-0.051	0.170429	-0.45597	-0.21025	-0.45809
GRSF1	-0.06876	-0.12642	-0.14682	-0.09584	-0.02187
GRTP1	0.31031	-0.16774	0.728365	0.362592	0.453292
GRWD1	-0.50851	-0.63077	-0.98681	-0.44831	-0.89992
GSC	-0.01298	0.292237	0.576542	0.482259	0.459537
GSC2	0.065827	0.157572	0.282389	0.122126	0.316183
GSDM1	0.232689	0.385971	0.519666	0.14221	0.430974
GSDMDC1	0.175355	-0.01339	0.485726	0.228663	0.263134
GSDML	0.624406	0.613965	0.773146	0.37203	0.437246
GSG1	0.344058	0.793113	0.736398	0.282106	0.539099
GSG1L	0.175323	0.290137	0.509271	0.214368	0.095216
GSH1	-0.29805	-0.01409	0.775739	0.522746	0.617881
GSH2	-0.03965	-0.36389	-0.40833	0.408962	0.037996
GSK3A AC006486.1	-0.36515	-0.32034	-0.36382	-0.22258	-0.18143
GSK3B	-0.62037	-0.56397	-0.66285	-0.38951	-0.41727
GSPT1	0.090924	0.24213	0.245013	0.285072	0.061751
GSPT2	0.05739	0.117591	0.348024	0.25708	0.11718
GSR	-0.15253	-0.12731	-0.1504	-0.19865	-0.15518
GSS	-0.22251	-0.29559	-0.52497	-0.41549	-0.45556
GSTA1	-0.18489	0.108432	-0.18419	-0.18189	-0.43846
GSTA2	-0.22347	0.109517	-0.20078	-0.28717	-0.55352
GSTA3	-0.0012	0.043063	-0.2092	-0.31801	-0.34281
GSTA4 AL162581.11	-0.42893	-0.37946	-0.31579	-0.20626	-0.42236
GSTA5	-0.18849	-0.11845	-0.42253	-0.3016	-0.77244
GSTK1	0.090551	0.084295	-0.01204	-0.01725	-0.02032
GSTM3	-0.34815	0.243122	-0.65352	-0.61399	-0.34198
GSTM4	0.089855	-0.01713	-0.08124	-0.02624	-0.0097
GSTM5	0.250217	0.28918	0.449705	0.33601	0.377645
GSTO1	-0.0455	0.006398	0.129703	0.218282	0.199177
GSTO2	0.071214	0.046068	-0.00833	0.082458	0.078444
GSTP1	-0.0572	0.394208	-0.07269	-0.02628	-0.01927
GSTT1	0.232505	0.43092	0.027193	0.16356	0.025232
GTDC1	0.01127	-0.04313	-0.32977	-0.36581	-0.0064
GTF2A1	-0.14083	0.066003	0.002495	-0.16959	-0.01916
GTF2A2	0.264963	0.265068	0.314518	0.173664	0.093498
GTF2B	0.053268	-1.85514	0.073915	-0.11585	-0.04665
GTF2E2	0.100435	-0.06607	0.168079	-0.05168	0.029688
GTF2F1	-0.31194	-0.15913	-0.29593	-0.24703	-0.29681
GTF2H2	0.211151	0.285933	0.027504	0.04852	0.027362
GTF2H4	-0.10852	0.034618	-0.19232	-0.01491	-0.20911
GTF2H4 VARSL	0.19504	0.254669	0.375819	0.379288	0.035271
GTF2I	-0.04722	-0.10131	-0.19664	-0.08403	-0.04883
GTF2IRD2	-0.01582	0.101212	-0.19215	-0.06599	-0.00368
GTF2IRD2B	-0.06317	0.020346	-0.24218	-0.15885	-0.03438
GTF3A	0.138572	0.176369	0.221679	0.142946	0.140324
GTF3C1 KIAA0556	-0.34792	-0.55417	-0.51361	-0.36852	-0.64276

GTF3C2	-0.1242	-0.3921	-0.35519	-0.21767	-0.19416
GTF3C3	0.339828	0.330382	0.435333	0.350112	0.46528
GTF3C5	-0.07274	-0.06178	-0.14703	-0.13361	-0.1492
GTPBP1	-0.31875	-0.17179	-0.19449	-0.35375	-0.30757
GTPBP10	-0.48953	-0.50808	-1.35487	-0.49056	-0.76803
GTPBP2	-0.38433	-0.3508	-0.6264	-0.25209	-0.45226
GTPBP3	-0.38596	-0.46238	-0.50538	-0.347	-0.47203
GTPBP5	0.422624	0.705644	0.771987	0.520441	0.694967
GTPBP6	0.298728	0.336642	0.495516	0.336087	0.449501
GTPBP8	0.242763	0.388905	0.374484	0.117028	0.290794
GTSE1	0.088065	0.329706	-0.05745	0.124236	0.089814
GTSF1	0.409943	0.186349	0.416187	0.327862	0.163898
GUCA1A	-0.38068	-0.39961	-0.63965	-0.71397	-0.54883
GUCA1B	-0.05346	0.177315	-0.09535	-0.04004	0.017893
GUCA1C	-0.30703	-0.22791	-0.83814	-0.53143	-1.18517
GUCA2A	0.168765	0.297573	0.314673	0.267669	0.238715
GUCA2B	0.370549	0.443222	0.42599	0.326314	0.048448
GUCY1A2	-0.14419	0.030093	-0.10386	-0.03873	0.165309
GUCY1A3	0.049323	-0.33799	-0.22413	-0.37413	-0.36496
GUCY1B2.	0.402642	0.667035	0.631078	0.250722	0.630644
GUCY2C	0.384794	0.746046	0.454344	0.376986	-0.03786
GUCY2D	0.031399	-0.19677	0.404982	0.092639	-0.01053
GUCY2F	0.154107	-0.4367	-0.65572	-0.40777	-0.46973
GUF1	-0.38358	-0.18429	-0.28309	-0.24404	-0.3612
GUK1	-0.04399	-0.23968	-0.19443	-0.06477	-0.07002
GULP1	-0.46042	-0.5916	-0.58658	-0.34082	-0.28194
GUP1	0.136535	0.206238	0.292988	0.120336	0.102124
GUSB	0.084023	0.160286	0.071063	-0.01265	-0.00989
GYG1	-0.06208	-0.13999	0.00494	-0.04006	-0.00936
GYG2	0.217015	0.463277	0.431531	0.30435	0.279132
GYLTL1B	0.079184	0.367016	0.37722	0.19357	0.365443
GYPA	-0.60135	-1.16356	-1.53475	-0.76171	-1.33163
GYPB.	-0.54307	-0.91784	-1.14338	-0.66199	-1.27304
GYPC	0.280518	0.574626	0.587585	0.379476	0.385423
GYS1 RUVBL2	-0.43658	-0.60451	-0.57608	-0.41603	-0.46469
GYS2	0.010452	0.209404	0.324701	0.116277	-0.35316
GZF1	0.234837	0.429628	0.517461	0.207954	-0.04747
GZMA	0.346999	-0.78666	-0.87895	-0.49973	-1.05401
GZMB	-0.80519	-1.30908	-1.23471	-0.83858	-0.99006
GZMH	-0.40557	-0.90678	-0.70301	-0.61142	-0.68883
GZMK	0.29087	-0.47957	-0.51102	0.011887	0.015785
GZMM	0.263813	0.291248	0.45116	0.292293	0.152374
H1F0	0.25657	-0.13008	0.174016	0.311663	-0.14691
H1F0 GCAT	-0.29014	-0.03795	-0.33041	-0.18828	-0.32669
H1FNT	0.249232	0.610193	0.417509	0.008495	0.039597
H1FOO	0.212925	0.237623	0.183446	0.059223	0.223489
H1FX C3orf47.	0.024382	-0.01265	-0.04564	-0.0521	0.093527
H2AFB2 F8A2	0.460808	0.503499	0.588198	0.512881	0.632279
H2AFJ	0.138668	0.579533	0.839949	0.65161	-0.15073
H2AFV	-0.28195	-0.32641	-0.4704	-0.18264	-0.11635

H2AFX	-0.21725	-0.39161	-0.44596	-0.13389	-0.28569
H2AFY	0.333594	0.541141	0.5244	0.27526	0.399143
H2AFY2	0.080308	0.101559	-0.10525	0.229854	0.035301
H2AFZ	-0.22285	-0.14156	-0.1189	-0.2135	-0.27049
H2BFWT	0.449665	0.398236	0.352164	0.247528	0.342308
H3F3A	-0.44037	-0.48559	-0.57987	-0.29059	-0.4808
H3F3B	-0.31506	-0.39931	-0.40377	-0.40878	-0.34458
H6PD	0.1663	0.348714	0.077743	0.144507	0.30034
HAAO	0.210652	0.244177	0.483472	0.17528	0.30851
HABP2	0.07579	0.266512	0.181891	-0.0535	0.22105
HABP4	-0.04894	-0.05354	-0.02429	-0.08968	-0.19279
HACE1	-0.12661	0.008967	-0.13626	-0.21544	-0.08621
HADH	-0.03762	-0.06277	0.039605	-0.08084	-0.26858
HADHA HADHB	-0.46432	-0.62516	-0.62154	-0.56482	-0.68043
HAGH FAHD1	0.067545	0.04241	0.101447	-0.06391	0.024283
HAGHL	0.240606	0.246604	0.456932	0.297302	0.237833
HAL	0.140216	0.098363	0.188378	-0.0811	-0.12202
HAMP	0.277653	0.178697	0.117862	0.037965	0.169494
HAND1	-0.27937	-0.22554	-0.07491	-0.21372	-0.27316
HAND2	-0.06865	-0.29329	-0.19879	-0.10764	-0.07516
HAO1	0.230954	-0.08057	0.333052	0.060087	-0.11533
HAO2	0.216131	-0.60743	-0.36073	-0.55951	-0.68754
HAP1	0.026662	0.265461	-0.06182	0.385946	0.064172
HAPLN1	0.350327	0.633074	0.585724	-0.26163	0.506378
HAPLN2	-0.12845	0.208692	0.118175	-0.31594	-0.21302
HAPLN3	0.195021	0.279993	-0.03495	0.127426	0.160213
HARS HARSL	-0.0416	-0.41184	-0.57685	-0.37742	-0.2463
HARS2	-0.12842	-0.24605	-0.43908	-0.21567	-0.29334
HAS1	0.231487	-0.8469	-0.09628	-0.06467	-0.69484
HAS2	-1.51695	-1.70572	-1.90224	-1.35434	-1.12882
HAS3	0.060222	0.217791	0.352909	0.125669	0.188187
HAT1	0.053599	0.149551	-0.09921	0.014602	0.184453
HAVCR2	0.255068	-0.0194	0.18442	0.009132	-0.4868
HAX1	-0.43282	-0.33457	-0.70439	-0.57402	-0.5366
HBA1	-0.02977	0.134302	0.354288	0.1823	0.114976
HBA2	-0.1067	-0.01114	0.250649	0.034087	0.090371
HBEGF	0.044163	-0.13921	-0.15688	-0.23781	-0.07531
HBM	0.093383	0.287924	0.226088	0.39847	0.304335
HBP1	-1.01697	-0.96112	-1.15174	-0.79529	-0.78636
HBQ1	0.399363	0.170978	0.595037	0.283188	0.534797
HBS1L	0.184413	0.269098	0.392809	0.181629	0.104376
HBXIP	-0.09809	-0.19969	-0.27914	-0.37383	-0.24775
HBZ	0.288895	0.324619	0.55834	0.092482	0.267771
HCCS	0.127795	-0.11422	-0.38694	-0.17898	-0.07346
HCFC1R1 THOC6	-0.24978	-0.36778	-0.31602	-0.28404	-0.40734
HCFC2	-0.09432	-0.03337	-0.07036	-0.09315	-0.20506
HCG9	-0.0667	-0.08043	-0.20617	-0.18179	0.211719
HCK	-0.22684	-0.12948	0.636704	0.484776	0.182428
HCLS1	0.389392	0.723804	0.607235	0.349155	0.484518
HCN1	0.01726	-0.00681	0.231236	0.203248	0.178281

HCN2	0.133681	0.154655	0.209882	0.159469	0.140314
HCN3	-0.28351	-0.25722	-0.71084	-0.6446	0.028804
HCN4	0.088638	0.163237	0.408999	0.111259	0.272706
HCP5	-0.18667	-0.65981	-0.75765	-0.13617	-0.01563
HCRT	0.078302	0.371506	0.227089	0.274489	0.370191
HCRTR1	0.419597	0.62851	0.746768	0.588983	0.558001
HCRTR2	0.329633	-0.32705	-0.20601	0.404376	-0.15757
HDAC1	0.386555	0.597239	0.150719	0.333323	0.246454
HDAC10	0.237695	0.12469	0.028466	0.219684	0.114414
HDAC11	0.038974	0.122295	-0.18954	0.167502	0.316852
HDAC2	-0.23521	-0.15752	-0.48751	-0.36165	-0.45898
HDAC3 C5orf16	-0.04955	-0.26251	-0.224	-0.18695	-0.21934
HDAC4	0.074423	0.039719	0.14715	0.086793	0.108037
HDAC5	-0.13297	-0.09296	-0.0347	-0.26567	-0.11705
HDAC6	-0.01396	-0.27139	0.127061	-0.16717	-0.30485
HDAC7	0.289114	0.6523	0.55981	0.372998	0.357796
HDAC8	-0.01278	-0.27543	-1.03012	-0.4187	-0.56432
HDAC9	-0.61588	-0.31672	-1.627	0.260178	-1.29372
HDC	0.130326	0.472491	0.704988	0.246286	0.230924
HDDC2	0.28731	0.188036	0.080019	-0.01009	0.271666
HDGF	-0.09592	-0.20999	-0.23308	-0.26663	-0.14654
HDGFL1	0.435086	0.682165	0.632625	0.52742	0.692918
HDHD1A	0.008488	0.07774	0.157688	0.040304	0.135259
HDHD2	-0.53085	-0.56892	-0.47466	-0.47314	-0.37326
HDHD3	0.322002	0.185732	-0.09212	0.007854	0.146698
HDLBP	0.103406	0.243396	0.23614	0.16101	0.218454
HDLBP SEPT2	-0.16332	-0.28203	-0.28072	-0.18358	-0.08646
HDX	0.139042	-0.3731	-0.13105	-0.39423	-0.12458
HEATR1	-0.03158	-0.04907	-0.32246	-0.30101	-0.19533
HEATR3	0.468298	0.506025	0.500072	0.344554	0.378906
HEATR5B CCDC75	-0.87514	-0.98558	-1.25441	-0.88236	-1.0135
HEATR6	0.002848	-0.78609	-1.26532	-0.88234	-0.68981
HEBP1 AC007688.15	-0.20552	-0.04515	-0.1888	-0.12563	-0.0549
HECA	0.143243	0.249758	0.271599	0.161699	0.301824
HECTD1	0.706545	0.165051	0.120789	0.146788	0.343805
HECTD2	-0.1309	-0.0062	0.013676	0.051113	0.049842
HECTD3 UROD	-0.30987	-0.37154	-0.37878	-0.19657	-0.25713
HECW2	-0.02804	-0.13341	-0.149	-0.29556	-0.1329
HELB	-0.67421	-0.59802	-0.74505	-0.63386	-0.77342
HELLS	-0.55279	-0.46586	-0.36196	-0.4067	-0.5027
HELT	-0.4304	-0.50229	0.063437	0.271566	0.125961
HELZ	-0.16126	-0.15378	-0.20859	-0.16034	-0.20247
HEMK2	0.324334	0.372278	0.595691	0.095866	0.284163
HEPH	-0.9791	-0.63542	-0.77579	-0.46019	-0.59061
HERC1	-0.00221	-0.07724	-0.12175	0.222963	-0.0397
HERC2	0.189483	0.494097	0.384059	0.110755	0.473601
HERC4	-0.14962	-0.22675	-0.24662	-0.24855	-0.1734
HERC5	0.101874	0.176623	0.114921	0.162606	0.559082
HERC6	0.321142	0.462697	0.298185	0.403855	0.211297
HERPUD1	0.10332	0.423617	0.317573	0.043776	0.451787

HERPUD2	0.03409	-0.07062	-0.49791	-0.17011	-0.05411
HES1	-0.58552	-1.03052	-0.96489	-0.60384	-0.89832
HES2	0.050046	0.129046	0.532903	0.226806	0.320947
HES2 ESPN	0.203235	0.118028	0.219238	0.243266	0.251512
HES4	0.033319	0.177652	0.096287	0.114867	0.176849
HES5	0.279551	0.159329	0.308715	0.329463	0.298406
HES6	0.025969	0.238086	0.18445	0.188643	0.233681
HES7	-0.88995	-0.46486	-0.97831	-0.45624	-0.50477
HESX1	-0.03317	0.499555	0.53928	0.161141	0.287219
HEXA	-0.223	-0.24486	-0.47302	-0.37072	-0.46123
HEXB	0.15686	0.254004	0.315359	0.225959	0.203011
HEXIM1	0.112757	0.067757	0.010658	0.006177	0.242024
HEXIM2	-0.07751	-0.16984	-0.12873	-0.29752	-0.15932
HEY1	0.0634	-0.03717	0.005795	0.09208	0.012879
HEY2	-0.31459	-0.22592	-0.02814	-0.07755	-0.18617
HEYL	0.463382	0.354132	0.607011	0.298386	0.441781
HFE2	-0.38643	-1.26655	-1.07359	-1.2368	-0.98072
HGF	0.402035	-1.06625	-0.5119	0.411031	-0.53698
HGFAC	0.295479	0.267323	0.382852	0.281623	0.374894
HHAT	0.170144	0.186421	-0.13492	0.186805	0.132121
HHEX	-0.05418	-0.00839	0.171346	0.054831	0.159791
HHIP	0.225291	0.095351	0.241327	0.210111	0.226666
HHIPL1	0.19804	0.276231	0.461893	0.324443	0.322649
HHIPL2 TAF1A	0.066748	0.235867	-0.0268	-0.12121	-0.00212
HHLA2	0.291272	0.49534	0.541215	0.01868	0.286383
HIAT1	-0.26851	-0.33369	-0.34851	-0.41809	-0.33099
HIATL2	-0.43918	-0.32861	-0.49841	-0.34001	-0.55666
HIBADH	0.085566	0.05481	-0.01099	0.018887	0.160278
HIBCH	-0.22696	-0.09787	-0.10566	-0.26921	-0.31731
HIC1	0.102568	-0.08949	0.21393	0.060391	-0.00969
HIC2	-0.09894	-0.08342	-0.08169	0.052006	-0.12825
HIF1A	-0.16559	-0.01752	-0.18772	-0.18764	-0.10523
HIF1AN	-0.09705	-0.17887	-0.56565	-0.6021	-0.35541
HIGD1A	-0.32672	-0.07697	-0.33044	-0.25582	-0.26443
HIGD1B	0.347392	0.629408	0.496567	0.249204	0.37971
HIGD2BP. BBS4	0.101799	0.168992	0.106646	0.108016	-0.28289
HINT1	0.24146	0.127973	0.231644	0.116287	0.060129
HINT2 C9orf127	-0.1	-0.03865	-0.15893	-0.2245	-0.38229
HINT3	-0.08428	0.058136	-0.19887	-0.19602	-0.24722
HIP1	0.061118	-0.03486	-0.04983	-0.1071	-0.06931
HIP1R	-0.29335	-0.43049	-0.3465	-0.2366	-0.31643
HIPK2.	0.535847	0.659914	0.624728	0.281996	0.656642
HIPK4	0.533602	0.699131	0.755399	0.524962	0.649868
HIRA MRPL40	-0.05309	0.24912	0.08436	0.103839	0.085074
HIRIP3 CCDC95	-0.35078	-0.29572	-0.49658	-0.22104	-0.18199
HISPPD1	-0.17714	0.064387	-0.0579	-0.05531	-0.08784
HIST1H1A HIST1H3A HIST1H4A	0.020076	0.333527	0.25282	0.098642	0.189955
HIST1H1D	-1.14684	1.054422	0.271197	-0.89099	-1.28082
HIST1H2AA HIST1H2BA	0.6315	1.00951	1.051469	0.799407	0.96385
HIST1H2BG HIST1H2AE	-1.03316	-1.01155	-0.97783	-0.65892	-0.90652

HIST1H2BJ HIST1H2AG	-1.35338	-1.22366	-1.14699	-0.97362	-1.01449
HIST1H2BK	-0.38351	-0.32012	-0.43958	-0.3458	-0.43035
HIST1H2BK HIST1H2AH	-0.71266	-0.51934	-0.82114	-0.43729	-0.6157
IIST1H2BL HIST1H2AI HIST1H3H	-0.70646	-0.76229	-0.77406	-0.55407	-0.71012
HIST1H3E	-0.85909	0.681866	-0.90659	-0.13493	0.551504
HIST1H3F HIST1H2BH	-0.61951	-0.43953	-0.6358	-0.36566	-0.62479
ST1H3G HIST1H2APS4 HIST1H2	-0.45333	0.088397	-0.85558	0.076287	-0.75971
HIST1H4A HIST1H4E	-0.31239	-1.27351	-1.61106	-0.22255	-0.64925
HIST1H4F	0.297598	0.835423	0.903939	0.474451	0.708112
HIST1H4G	0.446777	0.665997	0.81258	0.35403	0.476071
HIST1H4H	-0.80456	-0.70588	-0.91386	-0.51363	-0.86806
HIST2H2BE	-1.20203	-1.28934	-1.52715	-1.31717	-1.05986
HIST2H2BE BOLA1	-0.87229	-0.99401	-1.22494	-0.94487	-0.97066
HIST2H2BF HIST2H3D	-1.06661	-0.46152	-2.01826	-0.0869	-0.99008
ST2H3C HIST2H2AA3 HIST2H2B	-0.84977	-1.08238	-1.17784	-0.88832	-0.9209
HIST2H4A	-1.25222	-1.35863	-1.61568	-1.34216	-1.07477
HIST3H2A HIST3H2BB	-0.99109	-1.0885	-1.21063	-0.88072	-0.84183
HIST3H3	0.383028	0.531595	0.738098	0.393031	0.436357
HIST4H4	-0.2014	-0.58368	-0.69329	-0.51879	-0.39221
HIVEP1	-0.48223	-0.26838	-0.30687	-0.29782	-0.1523
HIVEP3	-0.09498	-0.17514	-0.29172	-0.32398	-0.13772
HJURP	-0.16503	-0.07533	-0.33802	-0.12165	-0.27846
HK2	0.15996	0.073329	-0.06139	-0.26205	-0.01349
HK3	0.584743	0.756716	0.883369	0.548561	0.682674
HKDC1	0.163387	0.627565	0.67685	0.351487	0.390832
HKR1	0.412509	0.62509	0.436367	0.547528	0.47385
HLA-A	-0.07019	-0.11754	-0.11869	0.237613	0.087822
HLA-DMA	0.381587	0.015658	0.356	0.02367	0.327262
HLA-DMB	0.30488	-0.48849	0.101617	-0.14254	-0.02983
HLA-DOA	0.346826	0.086148	0.431981	0.276126	0.448169
HLA-DOB	-0.1804	-1.26384	-0.93594	-0.83641	-0.80118
HLA-DPA1	0.320797	-1.29988	-1.17759	-0.98428	-0.98211
HLA-DQA1	0.232479	-0.80628	-0.48789	-0.61086	-0.83444
HLA-DQA2	0.292227	-0.70927	-0.36415	-0.45042	-0.33665
HLA-DRA	0.253071	-1.12638	-1.3619	-0.68605	-0.46852
HLA-DRB1	0.121149	-1.42898	-1.07127	-0.67051	-0.58067
HLA-DRB5	0.213078	-0.79938	-0.50414	-0.55298	-0.62435
HLA-E	-0.17381	0.187848	0.258176	0.373048	0.367619
HLA-F	0.094192	0.138801	0.251316	0.420765	0.398191
HLA-G	-0.04998	-0.29306	0.029673	0.061531	0.102874
HLCS	0.286767	0.386101	0.481609	0.311861	0.449526
HLF	-0.02692	0.301425	0.289889	-0.13404	0.141198
HLTF	0.238648	0.289518	0.437912	0.004517	0.423989
HLX	0.025885	0.582666	0.546439	0.31371	0.359964
HM13	-0.06668	-0.05913	-0.07837	-0.16065	-0.06863
HMCN1	-0.14922	-0.44276	-0.57421	-0.36751	-0.52442
HMG1L1	0.913332	0.899419	0.911593	0.688797	0.465996
HMG20A	-0.99069	-0.87492	-1.02657	-0.72988	-0.93871
HMG2L1	0.212902	0.223246	0.08807	0.028063	0.050442
HMGA1	0.006653	0.098364	0.149925	-0.02133	0.085546

HMGB1	-0.45445	-0.25817	-0.4394	-0.20473	-0.48677
HMGB1 USPL1	-0.47646	-0.6929	-0.63673	-0.38562	-0.56241
HMGB2	-0.75049	-0.99673	-0.76904	-0.68903	-0.79694
HMGB3	0.098113	0.158919	0.287684	0.215914	0.370846
HMGCL	0.38425	0.358147	0.650734	0.380566	0.425704
HMGCLL1	0.25502	0.13925	0.462716	0.538502	0.322911
HMGCR	-0.28933	-0.42231	-0.58447	-0.29906	-0.29373
HMGCS1	-0.29967	-0.42728	-0.23396	-0.39066	-0.58827
HMGCS2	0.037567	-0.77696	0.337093	-0.35414	0.008435
HMGN1	0.062941	0.034705	0.124821	0.025979	0.086277
HMGN2	-0.01005	-0.11998	-0.08648	-0.06776	0.105253
HMGN3	-0.11864	-0.0677	-0.27646	-0.34552	-0.14343
HMGN4	-0.69268	-0.58977	-0.5599	-0.5047	-0.78433
HMHA1	-0.01603	-0.24301	-0.23442	0.069079	-0.03347
HMHB1	0.600772	0.541	0.577455	0.409384	0.14971
HMOX1	0.123357	0.018645	0.123978	0.085223	0.103658
HMX2	-0.30527	-0.03342	-0.07368	0.074555	0.054565
HN1	-0.29802	-0.32133	-0.45413	-0.4858	-0.18304
HNF1A	0.328444	0.64382	0.550497	0.371357	0.449689
HNF1B	0.317734	0.148097	0.597003	0.378526	0.405556
HNF4G	-0.40744	-0.21465	-0.19689	-0.36781	-0.19898
HNMT	0.561711	0.51694	0.727363	0.502015	0.687904
HNRNPAB	0.027935	0.034783	-0.11567	0.033046	-0.07873
HNRNPC	-0.25824	-0.3432	-0.72972	-0.62192	-0.52466
HNRNPH1	-0.13241	-0.32644	-0.06393	-0.13241	-0.01507
HNRNPL	-0.23122	-0.07894	-0.2664	-0.2155	-0.1163
HNRNPR	0.913601	-0.15865	-0.105	0.020591	-0.00891
HNRNPU	-0.20398	-0.16474	-0.21355	-0.3132	-0.17245
HNRNPUL1	-0.31264	-0.51842	-0.40747	-0.4674	-0.55639
HNRPA0	-0.32133	-0.25165	-0.3051	-0.35065	-0.30033
HNRPA2B1 CBX3	-0.39963	-0.56091	-0.46957	-0.39407	-0.28476
HNRPA3	-0.11123	0.024561	-0.29284	-0.10919	-0.08668
HNRPD	-0.39912	-0.35025	-0.38963	-0.32752	-0.33106
HNRPDL ENOPH1	-0.20914	-0.22569	-0.18343	-0.15712	-0.14792
HNRPF	0.045278	0.080173	-0.00079	0.025992	0.145985
HNRPK RMI1	-0.66539	-0.50658	-0.5808	-0.49987	-0.62105
HNRPLL	-0.09027	-0.15782	-0.11868	-0.19953	-0.17048
HOMER1	-0.19575	-0.15966	-0.1441	-0.22053	-0.33052
HOMER2	0.314178	0.466778	0.409484	0.280024	0.590582
HOMER3	-0.11992	-0.13246	-0.33692	-0.3213	-0.17211
HOMEZ	-0.43373	-0.71006	-0.64972	-0.62231	-0.41942
HOOK1	-0.01416	0.264831	0.004566	0.342259	0.079998
HOOK2	0.257329	0.288387	0.244613	0.129128	0.27403
HORMAD1	0.478509	0.541341	1.001142	0.483778	0.642927
HORMAD2	0.386167	0.593331	0.707792	0.472077	0.375689
HOXA1	0.175664	0.150098	0.101202	0.258393	0.366599
HOXA11	-0.58127	0.334328	0.557045	0.55316	0.262477
HOXA13	-0.27946	-0.16342	-0.03291	0.087956	0.018593
HOXA2	-0.75546	-0.36621	0.104004	0.397519	0.483814
HOXA7	-0.38495	-0.14844	0.547298	0.216117	0.1589

HOXA9	-0.55407	0.073081	0.938528	0.620531	0.721019
HOXB1	0.330461	0.276399	-0.04576	-0.00293	0.271488
HOXB13	-0.17288	-0.5632	-0.31997	0.182375	-0.25987
HOXB2	-1.1884	-0.82592	0.060459	0.111796	0.343819
HOXB3	-0.98099	-0.99466	-0.57219	-0.38187	-0.23077
HOXB4	-0.84567	-0.1381	0.580046	0.350997	0.598232
HOXB5	-0.58596	-0.56842	-0.06232	0.429676	0.463543
HOXB6	-0.21514	-0.27852	-0.16345	0.412454	0.378724
HOXB6 AC103702.3	-0.4295	-0.54446	0.380903	-0.01138	-0.47359
HOXB7	-0.368	-0.2716	0.383024	0.484194	0.271635
HOXB8	-0.85598	-0.57448	0.29055	0.521057	0.511829
HOXB9	-0.73699	-0.32482	-0.29381	0.321246	0.066847
HOXC10	-0.37302	-0.16713	-0.54672	0.181278	-0.74062
HOXC11	-0.25272	-0.24258	0.249786	0.426086	0.433591
HOXC12	-0.24489	-0.34773	0.215789	0.226705	0.071072
HOXC13	-0.17371	0.00947	0.092039	0.254301	0.093557
HOXC8	-0.73738	-0.06115	0.055797	-0.22881	-0.33132
HOXC9	-0.64591	-0.39035	-0.59303	-0.22886	-0.54258
HP1BP3	0.581854	0.571994	0.749712	0.146127	0.362717
HPCA	0.085164	0.108296	0.176246	-0.13269	0.156959
HPCAL1	0.01949	-0.02366	-0.05684	0.066504	0.02931
HPCAL4	-0.10231	-0.21227	-0.23757	-0.12051	-0.12357
HPCL2 BTD	-0.91974	-0.56057	-1.36917	-1.16709	-0.82427
HPD	0.244134	0.618618	0.556627	0.534762	0.530922
HPDL	0.255218	0.338128	0.518014	-0.20199	0.243558
HPGD	-0.205	-0.24488	-0.08689	0.17398	-0.20013
HPRT1	0.04309	-0.07945	0.275648	-0.11735	-0.22594
HPS1	0.030863	0.131063	0.192324	0.01353	-0.01184
HPS3	0.063955	-0.08402	-0.17886	-0.01848	0.028745
HPS4	0.838774	0.969339	1.285352	-0.19418	0.45294
HPS4 SRRD	-0.22367	-0.27288	-0.23524	-0.24308	-0.3897
HPS5 GTF2H1	-0.52245	-0.52753	-0.4779	-0.28507	0.483746
HPS6	0.071711	0.05371	0.266107	0.096885	0.163515
HPSE	-0.06807	0.563118	0.168207	0.177565	-0.37924
HPSE2	-0.68918	0.35396	0.318694	0.440794	0.536276
HPX	0.258162	0.368855	0.167747	-0.02783	0.202663
HR	0.161769	0.166395	0.306631	0.127851	0.081136
HRAS LRRC56	0.037941	0.184858	0.092341	0.096602	0.140635
HRASLS	0.072489	-0.18009	-0.19573	-0.24437	-0.14888
HRASLS2	0.117154	0.300542	0.411243	-0.17333	0.052081
HRASLS3	0.153764	0.399946	-0.20388	0.338912	0.080511
HRASLS5	0.336103	0.308528	0.480603	0.497263	0.281237
HRB	0.087677	0.227897	0.40325	0.094993	0.254975
HRBL	-0.34293	-0.42759	-0.92561	-0.48726	-0.53287
HRC TRPM4	-0.26584	-0.35023	0.204078	-0.01647	-0.04405
HRG	0.555973	0.110335	0.472287	0.229283	0.150209
HRH1	0.478931	0.544091	0.757752	0.505266	0.285623
HRH2	0.490709	-0.52794	0.573361	0.069413	0.210657
HRH3	0.047769	0.092648	0.008499	0.048188	0.075429
HRH4	-0.19314	-0.03347	-0.32501	-0.46243	-0.11184

HRK	-0.3741	-0.00591	0.074085	-0.15269	-0.11682
HRNR	-0.89386	-1.37378	-1.18027	-0.84782	-0.94352
HRSP12 POP1	-0.39643	-0.67609	-0.85955	-0.86552	-0.53758
HS1BP3	-0.05386	0.109998	0.14212	0.171915	0.214753
HS2ST1	0.583938	0.631572	0.54515	0.566946	0.555548
HS3ST1	0.081549	0.549709	0.529203	0.38959	0.542884
HS3ST2	0.137665	0.329763	0.398632	0.224473	0.142402
HS3ST3A1	0.001442	0.146746	0.304056	-0.07126	0.166954
HS3ST3B1	-0.26539	0.170359	0.023577	-0.36557	-0.13539
HS3ST4	-0.10058	-0.2866	0.107374	-0.36484	0.014043
HS3ST5	0.494723	0.443076	0.529795	0.141483	0.439998
HS3ST6	-0.08802	-0.00982	0.16757	0.196263	0.185776
HS6ST1	0.14065	0.135654	0.322688	0.138378	0.316714
HS6ST2	-0.11529	0.261242	-0.20329	0.076999	0.188248
HS6ST3	0.10215	0.208437	0.223106	0.266689	0.059863
hsa-let-7i	-0.24428	-0.21537	-0.09076	-0.16985	-0.22215
hsa-mir-1302-2 WASH1	0.364601	0.5818	0.429875	0.338026	0.322182
hsa-mir-137	-0.25305	-0.33072	-0.22209	0.44959	-0.3198
hsa-mir-219-1 RING1	-0.20075	0.023279	-0.27894	-0.17026	-0.20816
hsa-mir-320a POLR3D	-0.14985	-0.03552	-0.1684	0.010769	-0.18706
hsa-mir-564 TMEM42	0.280908	0.228625	0.422627	0.530226	0.420621
hsa-mir-570 AC069513.28	0.22601	0.466537	0.19037	-0.0364	0.105693
hsa-mir-632 ZNF207	-0.21054	-0.12779	-0.09073	-0.05396	-0.09935
hsa-mir-639 GPSN2	0.022606	-0.03979	-0.06918	0.080461	-0.0215
sa-mir-99b hsa-let-7e AC018755.:	0.510253	0.499273	0.736917	0.497046	0.453371
HSBP1	0.155495	-0.01244	0.058567	0.23534	-0.01648
HSD11B2	0.091062	0.352083	0.138564	0.293987	0.204864
HSD17B1	0.119287	0.181777	0.71847	0.214445	0.170723
HSD17B10	0.123971	-0.38235	-0.32606	-0.1319	-0.35369
HSD17B12	-0.09177	0.453868	0.291951	-0.09162	0.159957
HSD17B13	-0.03656	0.078164	-0.13464	-0.1257	-0.08685
HSD17B14 PLEKHA4	-0.45723	-0.80411	-0.91266	0.01579	-0.78749
HSD17B2	-0.36787	-1.43455	-1.53993	-0.85165	-1.58046
HSD17B3	0.264256	0.51822	0.495447	0.154412	0.108075
HSD17B4	-0.21432	-0.08816	-0.24602	-0.0307	-0.33262
HSD17B6	0.341298	-0.10451	0.035291	-0.06089	-0.09595
HSD17B7	-0.05394	-0.2578	-0.21971	-0.32835	-0.03893
HSD3B1	-0.85808	-1.85043	-1.49761	-1.71874	-1.57826
HSD3B2	-0.6899	-1.34274	-1.155	-1.19911	-1.1213
HSDL1 LRRC50	-0.26773	-0.11958	-0.43877	-0.21568	-0.34806
HSDL2	-0.27286	-0.2782	-0.17077	-0.30097	-0.16514
HSF2	-0.35906	-0.28416	-0.44638	-0.36324	-0.17709
HSF2BP	-0.32995	-0.07955	-0.24381	-0.26065	-0.32442
HSF2BP KIAA0179	0.337158	0.317378	0.39845	0.224904	0.169993
HSFY1	-0.30424	0.145806	0.244203	-0.40533	0.151612
HSFY2	-0.23814	0.023458	0.150299	-0.37773	0.090146
HSP90AA2.	-0.13333	-0.0642	0.018675	-0.04611	-0.04206
HSP90AA2. WDR20	-0.42333	-0.38215	-0.3733	-0.25208	-0.34715
HSP90AB1	-0.50317	-0.4594	-0.82931	-0.46681	-0.56757
HSPA12B	0.158888	0.088008	0.125137	0.209978	0.158594

HSPA1B	0.383644	0.136904	0.470412	0.215192	0.496829
HSPA1L HSPA1A	-0.14142	-0.19061	-0.06032	0.524676	-0.14873
HSPA4	-0.12933	-0.13169	-0.29952	-0.05745	-0.16075
HSPA4L	-0.01601	0.06554	-0.20661	-0.03128	-0.0666
HSPA5	-0.32007	-0.335	-0.45233	-0.52132	-0.43908
HSPA6	0.273918	0.177772	0.434422	0.31307	0.180872
HSPA8	-1.06973	-0.60539	-0.97038	-0.6993	-0.98017
HSPA9B	0.113924	0.364125	0.275312	0.233072	0.219821
HSPB1	-0.24384	-0.22914	-0.45874	-0.25131	-0.23518
HSPB3	0.353104	0.079857	0.394252	0.393121	0.301106
HSPB6 C19orf55	0.053846	-0.08169	0.088318	0.138521	0.159687
HSPB7	0.690247	0.197061	0.421091	0.373477	0.320956
HSPB8	0.138687	0.335001	0.1203	0.3844	0.240716
HSPBAP1 DIRC2	-0.4067	-0.18873	-0.40648	-0.37008	-0.44361
HSPD1 HSPE1	-0.16285	-0.13725	-0.22895	-0.23227	-0.07513
HSPG2	0.165405	0.31003	0.416505	0.203381	0.20063
HSPH1	-0.6298	-0.30499	-0.73276	-0.49641	-0.57302
HTATIP	0.044328	-0.3396	-0.79496	0.075954	-0.49746
HTATIP2	-0.29482	-0.07731	0.628176	0.429185	0.263909
HTATSF1	-0.00115	-0.07507	0.290044	-0.02854	-0.12219
HTF9C RANBP1	-0.15691	-0.33743	-0.22869	-0.06954	-0.24833
HTN1	0.594069	-1.01912	-0.78915	-0.60427	-0.65889
HTN3	0.530372	-0.93003	-0.89905	-0.56979	-0.96046
HTR1A	-0.22935	-0.11726	0.255503	0.191049	0.104975
HTR1B	-0.27229	0.020819	0.419831	0.356927	0.284994
HTR1D	-0.07813	0.380742	0.734335	-0.07671	0.342395
HTR1E	0.216137	0.579265	0.377278	0.487617	-0.10689
HTR2A	0.551772	0.322439	0.062417	0.297444	0.115592
HTR2C	-0.10892	0.019693	0.220278	0.369934	0.463225
HTR3A	-0.12668	0.078684	-0.03636	-0.08507	0.029251
HTR3B	-0.32033	-0.24798	-0.78334	-0.30395	-0.66698
HTR3C	0.011455	-0.0383	-0.0935	-0.07048	-0.18768
HTR3D	0.60984	0.592021	0.486863	0.15623	-0.03659
HTR3E	0.662022	0.841112	1.079948	0.591953	0.452776
HTR4	0.154522	0.123815	0.271989	0.107996	0.126466
HTR6	-0.0675	0.172305	0.171547	0.171867	0.186173
HTR7	0.146927	0.267533	0.324539	0.211388	0.351887
HTRA1	0.070465	0.165742	0.279796	0.242712	0.2239
HTRA3	0.247756	0.574563	0.55201	0.495363	0.465191
HTT	-0.17005	-0.21769	-0.24378	-0.23765	-0.27985
HUNK	-0.0784	-0.01178	-0.01856	0.011736	0.044284
HUWE1	-0.46137	-0.09853	-0.51764	-0.30473	-0.21613
HYAL1	0.148267	0.273833	0.412596	0.243572	0.334182
HYAL2	0.213132	0.149741	0.279175	0.276713	0.205929
HYAL3 HYAL1	-0.34207	-0.24654	-0.55942	-0.35328	-0.24816
HYDIN	0.125179	-0.39778	-0.63828	0.116504	-0.58727
HYOU1	0.16174	0.206694	0.226706	0.206874	0.115959
IAH1	0.231488	0.287693	0.44406	0.292618	0.363207
IARS	0.005681	-0.3048	-0.46758	-0.32385	-0.38424
IARS2	0.375793	-0.08744	-0.29002	-0.10719	-0.17911

IBRDC2	-0.12056	-0.33336	-0.31047	-0.20887	-0.41127
IBSP	0.123259	0.152434	0.264237	-0.13461	-0.77698
IBTK	-0.06876	-0.08226	-0.04985	0.168179	-0.24213
ICA1	0.308197	0.40904	0.187298	0.164155	-0.12296
ICA1L	0.05565	0.130188	-0.06034	0.043603	-0.03429
ICAM1	0.136828	0.112175	0.4466	0.430906	0.514794
ICAM1 ICAM4	0.210719	0.315931	0.20116	0.232903	0.33192
ICAM2	0.367691	0.368208	0.396363	0.425323	-0.04357
ICAM3	0.270995	0.404583	0.464848	0.038551	0.379222
ICAM5	-0.53993	-0.34358	-0.50669	0.161755	-0.05152
ICK	-0.43265	-0.10055	-0.50633	-0.38838	-0.39982
ICMT	0.2767	0.218954	0.288349	0.12409	0.104497
ICOS	0.208173	0.157522	0.307941	-0.18314	0.135323
ICOSLG	-0.03707	0.153363	0.344969	0.218568	0.123766
ICT1	-0.17018	-0.71629	-0.89895	-0.58323	-0.54592
ID1	-0.34508	-0.16678	-0.47266	-0.38633	-0.3909
ID2	-0.32486	-0.34321	-0.39985	-0.27308	-0.35477
ID3	-0.7837	-0.8286	-0.78957	-0.30708	-0.4021
ID4	-0.20993	-0.39581	-0.32102	-0.14735	-0.20843
IDE	-0.03978	-0.14874	-0.16126	-0.13207	-0.03814
IDH1	-0.28404	-0.14133	-0.28013	-0.25709	-0.28526
IDH2	0.140299	0.135983	0.241269	0.128859	0.260738
IDH3A	0.038065	0.038494	-0.02658	0.011456	-0.02593
IDH3B	0.3258	0.305713	0.33781	0.253664	0.257681
IDH3G SSR4	-0.00234	-0.06591	0.167836	-0.15419	-0.05915
IDI1 WDR37	-0.24837	-0.18601	-0.29023	-0.28858	-0.16797
IDS	0.433745	0.377431	0.617754	0.248092	0.280606
IER3IP1	0.363356	-0.05603	0.152718	0.041734	0.419463
IER5	0.25154	0.208411	-0.1265	0.070439	0.376653
IER5L AL158151.16	-0.06236	0.102739	0.081241	0.090621	0.079727
IFI16	-0.30113	-1.11515	-1.03	-0.16509	-0.72445
IFI27	0.630728	0.74308	0.984249	0.352408	0.590022
IFI30	0.03798	-0.08169	0.076498	0.19125	0.145056
IFI35	0.425181	0.343051	0.570136	0.246301	0.166545
IFI44	0.122197	-0.1299	0.174074	-0.47379	-0.12953
IFI44L	0.055962	-0.08464	0.316424	-0.20955	0.452739
IFI6	-0.43676	-0.25988	-0.59366	-0.42436	-0.22319
IFIH1	-0.20315	-0.88987	0.554185	0.396251	0.363577
IFITM1	0.251182	0.205792	0.536241	0.285909	0.168271
IFITM5	0.157355	-0.26637	0.264138	0.251805	0.253949
IFLTD1	-0.25831	-0.12268	-0.362	-0.41786	-0.28626
IFNA1	-0.09778	-0.48353	-0.53583	-0.51689	-0.35245
IFNA13	0.014282	-0.28656	-0.45834	-0.35968	-0.36691
IFNA2	-0.59004	-0.29829	-0.61211	-0.44014	-0.62806
IFNA21	-0.41954	-0.37578	-0.73616	-0.57642	-0.75896
IFNA4	-0.70426	-0.59198	-0.74464	-0.92876	-0.97128
IFNA5	-0.40502	-0.48407	-0.7118	-0.56797	-0.34156
IFNA6	-0.16383	-0.02369	-0.14179	-0.16543	-0.10488
IFNAR1	-0.92437	-0.71474	-0.87209	-0.74707	-0.79252
IFNAR2	0.361011	-0.0887	0.122666	-0.28321	-0.00934

IFNB1	0.004362	0.000779	0.029755	-0.19523	-0.10237
IFNG	-0.06098	-0.01289	-0.08629	-0.2267	-0.40523
IFNGR1	-0.13986	-0.02997	-0.28907	-0.12562	-0.29022
IFNGR2	0.219705	0.352059	0.398831	0.211783	0.401064
IFNW1	0.258834	-0.09362	0.642994	0.124929	-0.02531
IFRD2 HYAL3	-0.10902	-0.01216	-0.14754	-0.2214	-0.03301
IFT172	0.120531	0.136263	-0.21561	0.033379	0.091827
IFT20 TNFAIP1	-0.41395	-0.21216	-0.57236	-0.2462	-0.20855
IFT52	-0.18231	-0.0421	-0.38796	-0.16111	-0.47682
IFT57	-0.47843	-0.54179	-0.66369	-0.41371	-0.47338
IFT80 SMC4	-0.75043	-0.74277	-0.844	-0.63633	-0.8372
IFT81	-0.14751	-0.13501	-0.26432	-0.29579	-0.2059
IFT88	-0.1576	0.037492	0.056273	-0.1651	-0.21315
IGBP1	-0.025	-0.23579	-0.12182	-0.36632	-0.27437
IGF1	-0.00898	0.252279	-1.45934	-0.16704	-0.93098
IGF1R	-0.02004	0.165477	0.141394	0.118999	0.08382
IGF2BP1	-1.05757	-0.56636	0.127745	-1.24623	-0.29654
IGF2BP3	-0.38901	-0.42872	-0.36787	-0.29318	-0.21723
IGF2R	0.115388	0.09185	0.079412	-0.0691	-0.00202
IGFALS	0.244665	0.158338	0.379787	0.228619	0.371237
IGFBP1	0.351123	0.514202	0.522608	0.413721	0.336093
IGFBP2	-0.21018	0.360483	0.262158	0.049674	0.246839
IGFBP3	-0.10143	-0.12543	0.226786	0.152383	0.107964
IGFBP4	0.152381	0.058599	0.21966	0.161909	0.213373
IGFBP5	-0.39341	0.132828	0.183822	-0.60988	-0.23226
IGFBP6	-0.20008	0.026135	0.161312	-0.06631	0.135248
IGFBP7	-0.05677	0.252635	0.554362	0.18144	0.272488
IGFBPL1	0.216993	0.190097	0.13394	-0.0337	0.004978
IGFL1	0.115927	-1.25358	-1.15029	-0.9805	-0.55789
IGFL4	0.155461	-1.57744	-2.04431	-1.36346	-1.74345
IGJ	-0.39702	-0.46732	-0.4795	-0.47213	-0.46121
IGLL1	0.207601	0.176011	0.35202	0.118209	0.043859
IGLL3	0.354268	0.496697	0.466814	0.227384	0.284914
IGSF1	-0.20127	-0.10516	-0.63503	0.147457	-0.00073
IGSF10	0.379223	0.299262	0.273191	0.180782	0.086519
IGSF11	0.244948	0.140025	-0.12802	-0.11286	0.016629
IGSF11 C3orf30	0.423728	0.731999	0.686428	0.45589	0.463727
IGSF2	-0.18744	-0.22882	-0.3118	-0.25248	-0.31561
IGSF21	-0.24878	0.092921	0.048276	0.073496	0.008538
IGSF22	0.065814	0.130107	0.125871	-0.14294	0.290629
IGSF3	0.101987	0.096729	0.005974	-0.19243	-0.0523
IGSF8	-0.16845	-0.42802	-0.47772	-0.17414	-0.10066
IGSF9	-0.35601	-0.24087	-0.46325	-0.35721	-0.19631
IHH	-0.10495	0.199386	0.268705	0.222254	0.123072
IHPK1	0.146816	0.033138	0.027738	0.092847	0.253865
IHPK2	-0.73219	-0.43292	-1.06144	-0.64424	-0.53471
IHPK3	-0.05815	-0.09055	-0.29055	0.081601	0.070548
IKBKAP C9orf6	-0.22078	-0.10216	-0.14653	-0.18323	-0.08366
IKBKB	-0.23205	-0.22714	-0.42708	-0.08825	-0.03649
IKBKE	-0.22292	0.063838	0.136917	0.145374	-0.01383

IKZF2	-1.51746	-0.7589	-0.14527	-0.10575	-1.24438
IKZF3	0.141269	-0.08255	-0.19411	0.164302	0.015722
IKZF4	0.203042	0.151812	0.077543	0.001912	0.053785
IKZF5 ACADSB	-0.43741	-0.34351	-0.39476	-0.42523	-0.33939
IL10	0.325983	0.397268	0.576283	-0.37338	0.598477
IL10RA	0.0731	0.168495	0.090892	0.100826	0.10101
IL10RB	0.088723	-0.17935	-0.54935	0.17299	-0.14185
IL11	-0.20324	-0.29422	-0.19044	-0.09822	-0.10203
IL11RA	0.167344	-0.9673	-0.5603	-0.31253	-0.49741
IL12A	0.327445	0.197063	0.230763	0.380497	0.400578
IL12B	0.392101	-0.01136	0.249241	0.094938	-1.06717
IL12RB1	0.281023	-0.57895	0.329375	-0.51429	-0.06637
IL12RB2	0.278182	0.592563	0.623883	0.009644	0.476509
IL13	0.262644	0.53575	0.467395	0.145666	0.377867
IL13RA1	0.089068	0.12119	0.227377	0.063607	0.125574
IL13RA2	0.857032	0.695952	0.858292	0.593356	0.083186
IL15	0.041139	-0.00972	0.450374	0.291795	0.039635
IL15RA	0.121407	0.149419	0.37945	0.307066	0.281928
IL16	0.357696	0.260491	0.717795	0.331238	-0.05057
IL17A	0.544262	0.609861	0.781705	0.461948	0.480492
IL17B	0.235511	0.454798	0.412592	0.327004	0.346617
IL17C	0.421937	-0.0628	0.413609	0.22815	0.376706
IL17D	0.067527	0.201272	0.396927	0.068659	0.240227
IL17F	0.4401	0.73677	0.66762	0.305525	0.406373
IL17RA	-0.07449	0.015885	0.118257	0.033012	0.146178
IL17RC	0.258698	0.059489	0.419952	-0.33178	0.507522
IL17RC CRELD1	-0.19292	-0.1206	-0.57849	-0.27036	0.082245
IL17RD	0.091041	0.508783	0.403338	0.046404	-0.05803
IL17RE	0.126358	0.334724	0.441063	0.254531	0.380705
IL17REL	0.057737	-0.09919	0.252783	0.160997	0.210654
IL18	0.391879	0.531881	0.792441	0.219232	0.463261
IL18R1	-0.21358	-0.34567	-0.34436	-0.27867	-0.13617
IL18RAP	-0.17915	-0.34989	-0.30876	-0.16717	-0.1433
IL1A	-0.31117	-0.12801	-0.52623	-0.72942	-0.80325
IL1B	0.179324	0.125771	-0.46576	-0.52748	-0.27578
IL1F5	0.120599	-0.36141	-0.23804	-0.27693	-0.12219
IL1F6	0.278724	-0.72239	-0.94927	-0.57476	-0.27674
IL1F8	0.146685	-0.91525	-0.69868	-0.5768	-0.45459
IL1F9	0.333799	-0.45758	-0.34811	-0.25687	0.050085
IL1R1	0.225076	0.139728	-1.10882	-0.92118	-0.08466
IL1RAP	-0.05179	-0.16907	0.105748	-0.03904	0.065406
IL1RAPL1	-0.2467	0.188701	0.694374	-0.79991	-0.91002
IL1RL2	0.219059	0.421187	-0.00927	0.026126	0.132829
IL20	0.641679	0.673336	0.747918	0.003062	0.584681
IL20RA	0.337532	0.508028	0.772658	0.418986	0.374412
IL20RB	0.375064	0.653358	0.50048	0.256609	0.492863
IL21	-0.07714	-0.69331	-0.40193	-0.39312	-0.68572
IL22	0.702843	0.825303	0.841923	0.56623	0.585775
IL22RA1	0.342642	-0.03925	0.312753	-0.357	0.031879
IL22RA2	0.06529	0.150351	0.01876	-0.12554	0.003557

IL23A	-0.6222	0.199475	0.153558	-0.08972	-0.18638
IL23R	0.474767	0.410223	0.276939	-0.0628	0.187631
IL24	0.00507	0.05954	0.015665	-0.27521	0.028271
IL25	-0.42333	-0.66733	-0.42757	-1.01702	-1.17741
IL25 CMTM5	0.372762	0.423545	0.530303	0.248884	0.234337
IL26	0.304567	0.275191	0.059135	-0.29993	-0.18859
IL27	0.319567	0.446707	0.46233	-0.10702	0.138652
IL28A	-0.12177	-0.77313	-0.34758	-0.45918	-0.13539
IL28B	-0.06903	-0.58791	-0.0576	-0.24814	0.154943
IL28RA	0.28973	0.431784	0.361851	0.261524	0.314266
IL29	-0.0671	-0.25755	0.494622	0.001042	0.223925
IL2RA	0.548927	0.714949	0.890928	0.343918	0.54216
IL2RB	0.286584	0.082023	0.370798	0.062826	0.168217
IL2RG	-0.08285	0.374916	-0.29185	0.118438	0.158021
IL3	0.467502	0.460166	0.594409	0.375949	0.570737
IL31	0.660648	0.659675	0.729159	0.553845	0.569596
IL31RA	-0.12978	-0.21968	-0.44596	-0.32343	-0.6057
IL32	0.392613	0.569262	0.403489	0.28176	0.497978
IL33	0.414926	0.151844	0.163462	-0.26226	-0.41668
IL3RA	0.359893	0.461414	0.70451	0.292696	0.590872
IL4	0.226841	0.048609	0.328963	0.026325	-0.04822
IL4I1	0.263455	0.38594	0.596807	-0.00557	0.485879
IL4I1 ATF5	-0.01011	-0.06732	-0.07018	-0.24226	-0.22382
IL4R	0.005339	-0.0587	-0.03411	-0.0554	0.050918
IL5	0.361873	0.365212	0.43866	-0.09808	0.085469
IL5RA	0.335322	-0.45311	0.427107	0.294459	0.120709
IL6R	0.130321	0.07408	0.147825	0.27709	0.280691
IL6ST	-0.28343	-0.25277	-0.08456	-0.17167	-0.14215
IL7	0.362701	-0.86813	-0.52631	0.219113	-0.46395
IL7R	-0.14636	-0.51299	-0.17033	-0.47068	-1.09201
IL8	-0.59168	-0.8607	-0.77196	-0.75805	-0.78972
IL8RA	-0.08199	-0.44249	-0.44195	-0.55592	-0.69114
IL8RB	0.38975	0.445103	0.597628	0.332406	0.323681
IL9	0.463579	0.829112	0.591614	0.341397	-0.28231
IL9R	-0.07316	0.480607	0.448418	0.078932	0.299096
ILDR1	0.236511	0.20107	0.49371	0.282634	0.442189
ILF2	-0.52962	-0.80631	-1.079	-0.81999	-0.86419
ILF3	-0.19653	-0.30531	-0.1904	-0.11115	-0.14581
ILI8BP	0.456707	0.56211	0.597095	0.388328	0.487829
ILKAP	0.165613	0.212331	0.434424	0.192981	0.214158
ILVBL	-0.35782	-0.29487	-0.4245	-0.46462	-0.46003
IMMP1L ELP4	-0.85796	-0.52258	-0.75306	-0.55272	-0.45595
IMMT	0.155068	0.115716	0.062125	0.115697	0.196444
IMP3	-0.17468	-0.16373	-0.11994	-0.23271	-0.26701
IMP3 SH3PX3	-0.03933	0.334591	0.063276	-0.02799	0.685216
IMPA1	0.177281	0.292947	0.287715	0.113719	0.19834
IMPA2	-0.19198	-0.02616	-0.00276	0.230539	0.087905
IMPACT	-0.03422	0.337213	0.261421	0.32117	0.281053
IMPAD1	-0.04982	-0.39379	-0.02171	-0.25125	-0.33964
IMPDH1	0.090919	0.199386	0.109601	0.015469	0.006779

IMPDH2 QRICH1	0.189602	0.116026	-0.21628	-0.17001	-0.01349
IMPG1	-1.09029	-0.71959	-1.01947	-0.72613	-1.22575
IMPG2	0.181153	0.203939	-0.24373	-0.22864	-0.50785
INA	-0.05966	0.058312	-0.19405	-0.00766	0.044399
INADL	0.248906	0.166377	0.111122	0.296279	0.373506
INDO	0.116249	0.002678	0.086148	-0.20905	0.021347
ING1	-0.52839	-0.55227	-0.41457	-0.39345	-0.40563
ING2	-0.13083	-0.11639	-0.15733	-0.02701	0.049513
ING3	-0.26581	-1.08249	-1.15985	-0.24263	-0.79554
ING4	-0.02626	-0.03859	-0.37654	-0.33322	-0.46783
ING5	-0.03419	0.031824	0.001771	0.059791	-0.05485
INHA	-0.12999	-0.4426	-0.44886	-0.1591	-0.42762
INHBA	0.220287	0.258304	0.29116	-0.07053	-0.28528
INHBC	0.007806	0.203421	0.008938	-0.11679	0.020017
INHBE	0.122107	0.209846	0.319957	0.063271	0.151333
INMT	0.233221	0.405938	0.489587	0.07663	0.454117
INOC1	-0.24003	-0.2346	-0.53612	-0.17792	-0.07387
INPP1	0.350301	0.23979	0.321218	0.212634	0.362507
INPP4B	-0.36228	-0.44144	-0.52601	-0.38224	-0.43484
INPP5A	0.10573	0.100225	0.150983	0.109992	0.103891
INPP5B	0.027599	-0.01145	0.077371	-0.11097	0.023531
INPP5E SEC16A	0.251564	0.112057	0.264756	0.163906	0.225675
INPPL1	-0.45039	-0.20645	-0.56733	-0.36682	-0.37576
INS TH	-0.02607	-0.07882	0.047002	0.021533	-0.02486
INSIG1	-0.05465	-0.07766	-0.07257	0.09832	0.080326
INSIG2	-0.03768	0.173192	-0.00509	-0.10173	0.082801
INSL4	0.31609	0.307539	0.09483	0.070904	-0.25524
INSL5	0.46204	0.668199	0.717432	0.314807	0.356254
INSL6	0.625552	0.750079	1.016932	0.572242	0.742583
INSM1	-0.09222	0.142016	0.340445	0.085641	0.193523
INSM2	0.051102	0.070959	0.152904	0.058088	0.094743
INSR	0.201454	0.238495	0.388869	0.106054	0.207303
INTS10	-0.20239	-0.15105	-0.36116	-0.05984	-0.12579
INTS12 GSTCD	0.060198	-0.50501	-0.35887	-0.38348	-0.42514
INTS3	-0.18156	-0.10737	-0.04964	-0.10821	0.019408
INTS4	-0.53519	-0.05816	-0.72877	-0.6631	-0.59125
INTS5	-0.04313	-0.08186	-0.46922	-0.1528	-0.2247
INTS6	-0.29858	-0.4114	-0.35708	-0.22895	-0.28682
INTS7 DTL	-1.13259	-1.12835	-1.58755	-1.2308	-1.19836
INTS8	-0.0932	-0.37916	-0.53779	-0.33054	-0.61141
INTS9 HMBOX1	-0.82009	-0.84665	-0.87051	-0.65892	-0.776
INTU	-0.49648	-0.58633	-0.32538	-0.08673	-0.36286
IPMK CISD1	-0.52151	-0.3886	-0.38592	-0.3225	-0.40675
IPO11	-0.12861	-0.03369	-0.14493	-0.1689	-0.2804
IPO13	-0.12975	-0.04268	-0.24517	-0.22273	0.089857
IPO4 TM9SF1	-0.07463	0.045686	-0.00656	-0.10863	-0.03942
IPO5	0.60577	0.475844	0.866913	0.584127	0.419684
IPO7	0.025571	0.191697	0.347268	0.062078	0.245488
IPO8	-0.06538	-0.17677	-0.00898	0.05081	0.096124
IPO9	-0.61406	-0.40466	-0.72558	-0.6917	-0.43976

IPP	0.250984	0.396615	0.497934	0.235173	0.221393
IPPK	-0.07157	-0.33438	-0.26242	-0.07885	-0.1612
IQCA	0.514207	0.652923	0.751242	0.488031	0.597591
IQCB1 EAF2	-0.41404	-0.57702	-0.74347	-0.46512	-0.26556
IQCD TPCN1	-0.29285	-0.30566	-0.53916	-0.23768	-0.21904
IQCE	0.254445	0.162709	0.419052	0.277293	0.264885
IQCG	-0.46112	-0.21916	-0.59809	-0.53152	-0.62153
IQCG LMLN	0.23575	0.107711	0.068223	-0.21359	0.118282
IQGAP1	0.103479	0.270715	0.120953	0.255866	0.103639
IQGAP2	0.107128	0.407291	0.944449	0.40135	0.311223
IQGAP3	0.006942	-0.01659	-0.13562	-0.22977	-0.09586
IQSEC1	-0.14124	-0.11363	-0.25343	0.127412	0.103062
IQSEC2	-0.07645	0.011932	0.215958	-0.02895	0.201895
IQSEC3	0.382861	0.604385	0.642906	0.531075	0.583972
IQUB	-1.44917	-1.16222	-2.17252	-1.31025	-1.55784
IRAK1 U52112.2	0.196592	0.148298	0.265463	0.150762	0.088506
IRAK1BP1	-0.00608	0.065245	-0.13645	-0.25582	-0.1042
IRAK2	0.380585	0.253596	0.605795	0.450896	0.516094
IRAK3	0.10316	0.136167	0.385924	0.19733	0.26345
IREB2	-0.31437	-0.5486	-0.6285	-0.47207	-0.59019
IRF1	0.119826	0.068655	0.169469	0.163792	0.128368
IRF2BP1	-0.11559	-0.04139	-0.30697	-0.13924	-0.10563
IRF2BP2	-0.10972	-0.07193	-0.02784	-0.03897	0.045301
IRF3 BCL2L12	-0.59618	-0.60441	-0.71819	-0.50148	-0.56646
IRF4	0.06535	0.140678	0.518513	0.363546	0.515925
IRF5	0.054984	0.167377	0.411362	0.310339	0.209547
IRF6	0.101208	0.404365	0.498012	0.315672	0.501993
IRF7 MUCDHL	0.059372	0.055375	0.072871	0.173469	0.112382
IRF8	0.269712	0.264116	0.697223	0.32657	0.36838
IRGC	0.402974	0.542345	0.66572	0.189222	0.112604
IRGQ ZNF576	-0.02275	-0.21667	-0.07378	-0.10559	-0.09187
IRS1	-0.13676	-0.29619	-0.19603	-0.01009	-0.18614
IRS2	0.066919	0.027876	0.132295	0.111741	0.212183
IRS4	0.008933	-0.19679	0.335174	0.305558	-0.17124
IRX1	-0.01927	0.228006	0.402461	0.170962	0.286835
IRX2 C5orf38	0.03691	0.060749	0.053451	0.064557	0.15401
IRX3	-0.22949	-0.31641	-0.31233	-0.18447	-0.09963
IRX4	0.010094	0.210866	0.556348	0.408042	0.444588
IRX5	-0.29939	0.043922	0.195351	-0.19558	0.069167
IRX6	-0.0907	0.082245	0.024941	0.205256	-0.03547
ISCA1	-0.02369	0.136349	0.002898	-0.05656	0.010102
ISG15	0.081894	0.226031	0.529067	0.311153	0.138576
ISG20	0.181437	0.175757	0.246997	0.040279	0.16953
ISG20L1	-0.05204	0.081365	0.099343	-0.02069	0.016434
ISG20L2 C1orf66	-0.16302	-0.87637	-0.82023	-0.39599	-0.56012
ISL1	-0.23988	-0.2354	-0.27107	-0.2386	-0.1751
ISL2	-1.01619	-0.58657	-0.78825	-0.57244	-0.64703
ISLR	0.331695	0.428804	0.515501	0.263776	0.274131
ISLR2	0.046552	0.168152	0.102831	-0.02774	0.553624
ISOC1	-0.10333	-0.18189	-0.25551	-0.13309	-0.10321

ISOC2	0.052544	-0.14357	-0.24747	-0.1496	-0.10848
ISX	-0.30643	-0.54677	-0.77343	-0.26685	-0.4421
ITCH	-0.01384	0.021968	-0.12445	-0.02555	0.00823
ITFG1 PHKB	-0.26248	-0.01479	-0.37514	-0.25191	-0.15343
ITFG2	-0.16173	-0.27839	-0.10805	-0.18557	-0.312
ITGA10	-0.27047	-0.2321	-0.37138	-0.52959	-0.32202
ITGA11	0.212499	0.390874	0.483756	0.268244	0.303278
ITGA2	0.083405	-0.1742	-0.12337	-0.00851	-0.07769
ITGA2B	0.413213	0.55244	0.538691	0.229283	0.355759
ITGA3	-0.18624	0.180442	-0.19095	-0.03494	-0.07025
ITGA4	0.325709	0.312509	0.257972	0.261691	0.411788
ITGA5	-0.1127	-0.29829	-0.27003	0.066679	-0.20786
ITGA6	-0.01524	0.282283	0.119648	0.181921	0.293466
ITGA7	-0.70262	-0.65413	-0.73114	-0.23345	-0.50458
ITGA8	0.144627	0.025298	0.313814	0.023647	-0.04626
ITGA9	0.07063	0.233283	0.082708	0.164793	0.041223
ITGAD	-0.19352	-0.93275	-0.80747	-0.71702	-1.01085
ITGAE	0.088877	0.110012	0.043193	0.046807	0.277583
ITGAM	0.20725	-0.88076	-0.64499	-0.45049	-0.69738
ITGAV	0.010442	0.031581	-0.08987	-0.0336	0.262964
ITGB1BP1 CPSF3	-0.13165	-0.11268	-0.28765	-0.17146	-0.26652
ITGB1BP3	-0.02029	-0.29261	0.199171	0.153883	-0.0307
ITGB2	0.548049	-0.64857	0.580754	0.372116	0.447386
ITGB3	0.267842	0.523179	0.438627	0.251375	0.289853
ITGB3BP EFCAB7	-1.39454	-1.22667	-1.5901	-1.50885	-1.02035
ITGB4BP	-0.22704	-0.2402	-0.55009	-0.09144	-0.17367
ITGB5	0.064551	0.192832	0.249803	0.261577	0.188588
ITGB6	0.260641	0.632727	0.569672	0.073332	0.338972
ITGB7	-0.22492	-0.01976	0.192879	-0.75104	-0.17993
ITGB8	-0.24683	-0.07454	-0.23265	-0.14889	0.037025
ITGBL1	0.205559	-0.35163	0.238184	-0.78479	-0.27254
ITIH1	0.550824	0.831058	0.713807	0.412686	0.506889
ITIH2	-0.29228	-0.5383	0.167824	-0.62617	-0.83949
ITIH3	0.277564	0.457996	0.490522	0.187408	0.461059
ITIH4	0.357129	0.425996	0.659693	0.445315	0.453788
ITIH5L	-0.60908	-0.10561	-0.4224	-0.15738	-0.24485
ITK	0.007823	-0.6046	0.18693	-0.07253	-0.44774
ITLN1	-0.1025	-1.45353	-1.31327	-1.09364	-0.92737
ITLN2	0.121987	-0.94953	-1.06516	-0.76656	-0.03447
ITM2A	0.069031	-0.73036	-0.30748	-0.08871	0.046141
ITM2B	0.177428	0.306612	0.263243	0.202662	0.316584
ITM2C	-0.14679	0.042209	-0.1607	-0.00946	0.016222
ITPA	-0.46735	-0.47601	-0.82841	-0.46274	-0.51716
ITPK1	-0.08615	-0.01824	-0.09193	-0.00137	0.007946
ITPKA	0.007061	0.028987	0.290685	0.165274	0.180233
ITPKB	0.035336	-0.02749	-0.12128	-0.23465	0.002088
ITPR1	-0.14745	-0.07576	-0.19598	-0.1584	-0.0951
ITPR2	-0.03515	0.048597	0.072072	0.102132	-0.03426
ITPR3	0.03957	0.234019	0.13324	0.33697	0.38508
ITSN2	0.206616	0.280511	0.239459	0.233534	0.292399

IVD	0.374005	0.168133	0.435477	0.233648	0.179102
IVL	-0.50407	-1.65292	-1.46691	-1.29676	-1.01944
IVNS1ABP	0.160779	-0.08274	-0.57609	-0.37735	0.344184
IWS1	-0.32048	-0.1022	-0.21002	-0.16857	-0.04897
IYD	0.481856	-0.36935	-0.70532	0.012093	-0.48184
IZUMO1	0.013747	0.442235	0.466367	0.436075	0.296718
JAG1	-0.20504	-0.1076	-0.06399	-0.02196	-0.03709
JAG2	0.021491	0.092494	0.12084	0.076347	0.0854
JAGN1	-0.16327	0.038514	-0.15941	-0.20184	0.035367
JAK2	0.0207	0.195755	0.181162	0.063849	0.035746
JAKMIP1	-0.10302	-0.11548	-0.01846	-0.17209	-0.16453
JAKMIP2	0.011197	-0.2848	-0.42142	-0.28147	-0.68689
JAKMIP3	0.152672	0.413844	0.430287	0.277039	0.465033
JAM2	-0.7504	-0.10268	-0.55419	-0.49716	-0.32595
JAM3	-0.26659	-0.00911	-0.17968	-0.17063	-0.22868
JARID1A	-0.79802	-0.75278	-0.98984	-0.93977	-0.9781
JARID1B	-0.08196	0.035333	-0.0446	-0.49882	-0.4906
JARID1C	-0.24499	-0.13097	-0.51833	-0.2507	-0.15279
JARID2	-1.28595	-1.1239	-1.24299	-0.98982	-1.38813
JAZF1	-0.14871	-0.22767	-0.46135	-0.47713	-0.29695
JDP2	0.294502	0.481808	0.339787	0.274114	0.531623
JHDM1D	-0.15417	-0.08342	-0.08414	-0.04548	-0.03536
JMJD1A	-0.57051	-0.47854	-0.66613	-0.5969	-0.42464
JMJD1B	-0.01301	0.128251	0.193895	-0.04709	-0.29518
JMJD1C	-0.58611	-0.34409	-0.49867	-0.50307	-0.26769
JMJD2A	-0.51665	-0.25194	-1.11679	-0.27093	-0.25864
JMJD2B	0.123787	0.060913	0.250608	0.093794	0.311313
JMJD2C	-0.15919	0.066939	-0.0386	0.010203	-0.00305
JMJD3 TMEM88	0.45602	0.609325	0.596732	0.509866	0.369563
JMJD5	0.19047	0.094276	0.194339	0.062205	0.145092
JMJD6 AC005837.1	0.151311	-0.13809	-0.14623	-0.1172	-0.06222
JOSD1	0.140665	0.150443	0.263055	0.089676	0.152091
JOSD2 AC008743.9	0.135971	-0.0826	-0.20536	0.099538	0.096901
JOSD3 C11orf54	-0.36586	-0.05349	-0.73086	-0.36752	-0.56623
JPH1	0.12279	0.099376	-0.07771	-0.10362	0.030613
JPH2	-0.29172	-0.12783	-0.05279	-0.01977	-0.27487
JPH3	-0.16938	0.140749	-0.12752	0.1934	0.071329
JPH4	-0.59044	-0.61475	-1.68939	-1.2594	-1.62217
JTB	0.043319	-0.1112	-0.30592	-0.15411	-0.22456
JUN	-0.33654	-0.18462	-0.41437	-0.40823	-0.30302
JUNB	-0.56738	-0.65837	-0.63862	-0.56281	-0.50767
JUND	-0.04428	0.011463	0.066899	0.004443	-0.00593
JUP	-0.09705	-0.14793	0.042964	0.049092	-0.20721
KAL1	0.046659	0.434033	0.208426	0.198219	0.299206
KANK2	0.450619	0.559843	0.789449	0.348806	0.511203
KANK4	0.331054	0.379584	0.60229	0.285443	0.233136
KARS TERF2IP	0.129106	0.171429	0.381459	0.207447	0.195606
KATNA1	0.369173	0.857882	0.721044	0.212496	0.277408
KATNAL1	-0.02063	-0.00534	-0.24946	-0.11802	0.028007
KAZALD1	0.422292	-0.99351	-0.30614	0.308183	-0.75771

KBTBD10	0.355135	0.421985	0.481447	0.096349	0.358154
KBTBD11	-0.07885	0.061752	0.006057	-0.03109	-0.00194
KBTBD3 AASDHPPT	-0.35158	-0.10801	-0.4084	-0.24365	-0.30914
KBTBD4 NDUFS3	-0.14982	-0.27053	-0.39564	-0.33502	-0.38778
KBTBD5	0.230522	0.419498	0.442137	0.356542	0.445317
KBTBD6	-0.10693	-0.41889	-0.5584	-0.43058	-0.70834
KBTBD7	0.298569	0.31446	0.420068	0.295498	0.264115
KBTBD8	-0.26599	-0.47232	-0.57132	-0.33276	-0.50895
KCNA1	-0.05781	0.060693	0.494852	0.521052	0.474648
KCNA2	-0.13514	-0.15354	-0.33457	-0.27802	-0.21653
KCNA3	0.427767	0.281715	0.344783	0.404911	0.53852
KCNA4	-0.01917	0.058283	0.582349	0.043574	0.15879
KCNA5	0.037727	-0.32969	-0.04731	0.090552	0.113155
KCNA7	0.322054	0.023398	0.55308	0.387923	0.305897
KCNAB2	0.23349	0.098173	0.251385	0.185915	-0.00059
KCNAB3 TRAPPC1 CNTROB	-0.15323	-0.04151	-0.39126	-0.11088	-0.21639
KCNB1	0.140612	0.262879	0.357133	0.324337	0.383744
KCNC1	0.160779	0.238488	0.211431	0.064907	0.20778
KCNC2	-0.50408	-0.68136	-0.34612	-0.45501	-0.27598
KCNC3	-0.88178	-0.58968	-0.96471	-0.01535	-0.3635
KCNC4	0.215308	0.274247	0.462341	0.273648	0.381483
KCND1	-0.29502	-0.52389	-0.23992	-0.00783	-0.37252
KCND3	-0.12134	0.126399	-0.01515	-0.20027	-0.04493
KCNE1	0.377408	0.669986	0.676328	0.270036	0.401005
KCNE1L	0.151999	0.185519	0.240767	0.208127	0.32696
KCNE2	0.271587	0.310808	0.027164	0.091298	0.191027
KCNE3	0.35835	0.380992	0.579307	0.34357	0.592416
KCNE4	0.622404	0.871501	0.884881	0.567942	0.599861
KCNF1	0.111681	0.400626	0.260242	0.226186	0.173602
KCNG2	0.250347	0.27563	0.483849	0.248364	0.380455
KCNG3 MTA3	-0.01014	0.040365	-0.02704	0.012314	0.124046
KCNG4	0.32621	-0.21401	-0.21699	-0.1352	-0.09771
KCNH1	0.097361	-0.15222	-0.02492	-0.23485	-0.23024
KCNH3	0.062498	0.188893	0.486041	0.025455	0.159264
KCNH4	-0.12852	0.132687	0.011579	-0.16299	-0.1414
KCNH8	-0.24275	-0.06923	-0.30025	-0.25083	-0.01378
KCNJ10	-0.59255	-0.84753	-1.01027	-0.56944	-0.74609
KCNJ11	0.12425	0.135119	0.212807	0.115984	0.067474
KCNJ12	0.242859	0.059985	0.006374	0.122347	0.302566
KCNJ2	-0.21095	-0.40848	-0.26502	-0.25049	-0.27629
KCNJ3	-0.33657	0.576921	0.556378	0.355542	0.414119
KCNJ6	-0.31916	-0.32011	0.031085	-0.31942	-0.18764
KCNJ8	-0.3801	-0.69514	-0.77118	-0.31235	-0.84988
KCNJ9	0.102318	-0.82269	-0.42503	-0.2522	-0.34451
KCNK1	0.000154	0.11531	0.018869	0.104513	0.398226
KCNK12	-0.14738	-0.07769	-0.01382	0.093717	-0.02559
KCNK13	-0.17154	0.067339	-0.05526	0.262912	-0.00969
KCNK15	0.07812	0.339229	0.28444	0.214868	0.211742
KCNK16	0.222028	0.035385	0.497412	0.162061	0.129576
KCNK17 KCNK16	0.121675	0.454246	0.404284	0.315941	0.296648

KCNK18	0.212053	-0.06585	0.398687	0.081232	-0.25719
KCNK3	0.139889	-0.11484	-0.2744	-0.10981	-0.22576
KCNK4	-0.00926	0.154064	0.081628	0.161114	0.186037
KCNK5	0.338866	0.348264	0.242902	0.229316	0.379224
KCNK6	0.145697	0.420748	0.547228	0.249261	0.351627
KCNK9	0.060672	0.120478	0.26627	0.094011	0.156456
KCNMA1	-0.22615	-0.11349	-0.17413	-0.16324	-0.18845
KCNMB4	-0.13564	-0.05013	-0.03464	-0.05494	-0.06293
KCNN1	-0.02153	-0.01634	0.367336	-0.05185	-0.17621
KCNN4	0.291885	0.660711	0.781965	0.312548	0.38652
KCNQ2	0.028656	0.10838	0.024449	0.002373	0.045746
KCNQ3	-0.3285	-0.08324	-0.05805	-0.12034	0.04148
KCNQ4	-0.00679	0.262455	0.271049	0.133344	0.137693
KCNQ5	-0.03076	0.0191	0.143308	-0.0715	-0.12075
KCNS1	0.411579	-0.04739	0.591189	0.059372	0.201797
KCNS2	0.074049	-0.11335	0.303599	0.230663	0.366055
KCNS3	0.028629	-0.0414	0.089998	0.057582	0.175185
KCNT2	-0.67889	-1.01238	-0.97479	-0.86267	-0.75278
KCNV1	-0.41553	-0.51331	-0.31531	-0.04943	0.065538
KCNV2	0.478172	0.486098	0.67389	0.384554	0.557416
KCTD10 UBE3B	-0.42501	-0.45902	-0.5747	-0.40358	-0.5969
KCTD11	0.087294	0.157605	0.088277	0.193313	0.230444
KCTD11 TMEM95	0.184517	0.219235	0.161581	0.046631	0.207411
KCTD12	0.069921	-0.02161	0.03096	-0.04825	0.163129
KCTD13	0.210845	-0.00656	0.004972	0.20781	0.031204
KCTD14	0.298978	0.545728	0.791418	0.338737	0.400131
KCTD15	-0.24963	0.076606	-0.27402	-0.18322	-0.28963
KCTD17	0.073223	0.270859	0.208742	0.111001	0.1604
KCTD18	0.003158	-0.07488	-0.17942	-0.13827	-0.10378
KCTD19 LRRC36	0.161986	0.343438	0.26559	0.21032	0.146912
KCTD20	0.027384	0.084294	-0.01474	0.058708	0.089098
KCTD21 USP35	-0.19413	-0.03023	-0.24504	-0.08656	-0.10959
KCTD3	-0.21337	-0.25651	-0.38523	-0.38971	-0.19274
KCTD5	0.272006	0.023866	0.031769	-0.00185	-0.05986
KCTD6	0.377176	0.34983	0.519209	0.218863	0.213554
KCTD7	0.055613	0.002861	0.065037	-0.07	0.105047
KCTD8	-0.08479	-0.01809	0.110446	-0.33455	-0.15888
KDELC1 BIVM	0.101138	0.022838	-0.04515	-0.05291	0.074962
KDELC2	0.054973	-0.10417	-0.14695	0.129742	0.244604
KDELR1	-0.28722	-0.5932	-0.95108	-0.59564	-0.65521
KDELR2	0.047796	0.074994	0.02577	0.071303	0.035003
KDELR3	0.08079	-0.02013	0.146263	0.173683	0.17488
KDR	-0.27864	-0.09774	-0.51498	0.215717	0.113163
KEAP1	0.098373	0.030996	0.115383	-0.02745	0.051578
KEL	-0.16076	-0.37423	-0.70419	-0.12068	-0.47047
KERA	-0.146	-0.23775	0.044086	-0.25749	-0.09428
KHDRBS1	0.0531	0.084244	0.108655	0.015261	0.092644
KHDRBS2	-0.19865	0.172183	0.642185	0.455402	0.062435
KHDRBS3	0.070884	0.213552	0.174579	0.082577	0.04906
KHK	-0.00521	0.223975	-0.26233	-0.02428	0.068083

KHSRP	-0.08478	-0.04009	-0.03558	-0.55272	-0.17537
KIAA0082	0.166179	0.27735	0.19438	0.246574	0.214632
KIAA0090 MRTO4	-0.49059	-0.71559	-0.92996	-0.39482	-0.50743
KIAA0100	0.049384	0.135826	-0.04299	-0.01628	0.010963
KIAA0101	-0.05796	-0.23913	-0.35944	-0.39588	-0.40996
KIAA0141	0.26415	0.100293	0.005158	0.147257	0.261579
KIAA0152	-0.21278	-0.33095	-0.20842	-0.32168	-0.21262
KIAA0157	0.128836	0.275304	0.38072	0.220998	0.309186
KIAA0174	-0.28804	-0.25661	-0.40154	-0.12784	-0.41153
KIAA0182	-0.38529	-0.28576	-0.55757	-0.28305	-0.40233
KIAA0195	-0.46523	-0.70111	-1.11723	-0.8831	-0.74577
KIAA0196	0.094558	0.10092	0.169286	-0.21109	0.104848
KIAA0196 NSMCE2	-0.37331	-0.67063	-0.55396	-0.61101	-0.67113
KIAA0232	0.071076	0.120526	0.022551	-0.00434	0.040232
KIAA0247	-0.00433	0.070833	-0.04206	0.035122	-0.03049
KIAA0317 FCF1	-0.59842	-0.74024	-0.52515	-0.6429	-1.02762
KIAA0319	-0.13536	-0.17788	-0.25654	-0.29162	-0.1138
KIAA0319L NCDN	-0.41803	-0.29304	-0.26937	-0.29649	-0.11645
KIAA0355	0.612565	0.763963	0.694805	0.342774	0.299843
KIAA0367.	0.492038	0.52115	-0.36842	0.248611	0.503819
KIAA0372 ARSK	-0.4488	-0.47865	-0.56126	-0.58364	-0.63339
KIAA0391	-0.27692	-0.40679	-0.77972	-0.39837	-0.69876
KIAA0406 C20orf77	-0.24171	-0.33304	-0.27734	-0.13938	0.060504
KIAA0408 C6orf174	0.493273	0.58173	0.807454	0.518156	0.753249
KIAA0409 ILK	-0.99452	-0.85997	-0.75205	-0.70814	-0.25243
KIAA0415	-0.02184	-0.16931	-0.05982	0.17495	-0.07603
KIAA0427	-0.22254	-0.10102	-0.41519	-0.31575	-0.08558
KIAA0430 NDE1	-0.47442	-0.55832	-0.912	-0.63344	-0.71598
KIAA0460	-0.23277	-0.70389	-0.66915	-0.50556	-0.63141
KIAA0467	0.359388	0.409336	0.529432	0.237542	0.363055
KIAA0494	-0.00019	0.026404	0.023322	0.055096	0.082681
KIAA0513	-0.0299	-0.06613	-0.20479	-0.09628	-0.28248
KIAA0528	-0.13121	-0.10456	-0.28535	-0.17275	-0.21688
KIAA0562 DFFB	0.100656	0.231504	0.17958	0.102905	0.141591
KIAA0564	-0.02918	-0.01348	0.045457	0.012081	-0.14674
KIAA0649	0.001547	-0.06049	0.163854	0.038776	0.174397
KIAA0753 TXNL5	-0.4946	-0.19961	-0.62208	-0.33319	-0.27676
KIAA0776	-0.69934	-0.51816	-0.88946	-0.59138	-0.82045
KIAA0802	0.63513	0.775866	0.982107	0.470418	0.599233
KIAA0831	0.116788	-0.28052	0.288413	-0.27301	-0.17532
KIAA0859	-0.23998	-0.38556	-0.7597	-0.90813	-0.62091
KIAA0892	0.239007	0.499586	0.47601	0.41068	0.285715
KIAA0907	-0.05224	-0.04294	-0.02634	0.004496	-0.34623
KIAA0922	0.043714	0.032529	0.13246	-0.01804	0.042131
KIAA0980	0.182791	0.115798	0.083994	0.221753	0.230407
KIAA1009	-0.26287	-0.3053	-0.63864	-0.4554	-0.55487
KIAA1012	-0.67356	-0.49027	-0.47547	-0.39061	-0.19863
KIAA1033	0.045936	-0.0061	-0.13058	-0.18895	0.008348
KIAA1143 KIF15	-0.12454	0.132044	-0.02206	-0.01222	0.053
KIAA1161	-0.07159	-0.10062	-0.0024	-0.19064	0.026986

KIAA1166	-0.0841	-0.49268	-0.76342	-0.1578	-0.50871
KIAA1191	0.23163	0.46006	0.234996	0.164127	0.521027
KIAA1199	0.057978	-0.02788	0.036483	-0.02814	0.036565
KIAA1217	-0.05085	0.293637	-0.12616	-0.07217	-0.086
KIAA1219	-0.3193	-0.31405	-0.29319	0.055262	-0.16559
KIAA1267	-0.03523	0.286178	0.239761	-0.01814	-0.21245
KIAA1274	0.011602	0.25	0.054853	-0.01524	0.093835
KIAA1279	-0.05518	-0.46679	-0.17294	-0.01721	-0.05623
KIAA1310	0.012493	0.043735	0.090166	0.073801	0.031462
KIAA1333	0.264702	0.037365	-0.0057	-0.00148	-0.17346
KIAA1407 QTRTD1	-0.48172	-0.68462	-0.71768	-0.41395	-0.5033
KIAA1409	0.390376	0.482664	0.579945	0.341038	0.45183
KIAA1429	-0.66002	-0.7397	-0.89533	-0.71325	-0.83767
KIAA1432	0.289027	0.356505	0.459902	0.109756	0.414781
KIAA1467	0.329209	0.064297	0.189842	0.093543	0.010318
KIAA1524 DZIP3	-0.58102	-0.69605	-0.80675	-0.61686	-0.83642
KIAA1529	0.037676	0.300028	-0.15199	0.255483	-0.13901
KIAA1530	0.092576	0.02207	0.226743	0.200227	0.200129
KIAA1539	-1.05019	-1.05039	-1.02189	-0.6109	-0.6945
KIAA1586	-0.33102	-0.45639	-0.757	-0.50957	-0.59448
KIAA1598	0.037864	0.052905	-0.17608	-0.08335	-0.20581
KIAA1600	0.065584	0.29788	0.136876	0.159015	0.240707
KIAA1602	0.351023	0.197259	0.472714	0.163565	0.468652
KIAA1609	0.09198	-0.00385	0.149284	-0.04808	-0.12565
KIAA1618	0.118752	0.077846	0.022432	0.113638	0.066611
KIAA1622	-0.09886	-0.15147	-0.12998	-0.03704	0.090464
KIAA1683	0.270505	0.315753	0.422635	0.307457	0.400062
KIAA1715	-0.18389	-0.16773	-0.3231	0.16835	-0.05118
KIAA1737	-0.20441	-0.23562	-0.17255	-0.18546	-0.15293
KIAA1754	0.467759	0.779108	0.801805	0.301184	0.602634
KIAA1754L	0.137908	-0.19358	-0.4365	-0.26875	-0.20746
KIAA1755	0.419599	0.472094	0.359051	0.332454	0.465831
KIAA1772	-0.01681	0.132427	0.375961	-0.16736	0.298589
KIAA1826	-0.09526	-0.17594	-0.20802	-0.15959	-0.23791
KIAA1841	-0.19073	-0.27036	-0.28207	-0.27295	-0.11127
KIAA1853	-0.49897	-0.92991	-0.51925	-0.51251	-0.47552
KIAA1913	-0.40219	-1.23813	-1.15528	-0.89046	-1.19847
KIAA1919	0.385471	0.259676	0.435925	0.290563	0.128231
KIAA1946	-0.14376	-0.06494	-0.20493	-0.21667	-0.04167
KIAA1958	-0.15531	-0.26271	-0.28849	-0.31494	-0.30223
KIAA1984 C9orf86	-0.00077	0.185382	0.187453	0.116461	0.119587
KIAA2022	0.161559	0.154025	0.239944	-0.0387	0.337567
KIAA2026	0.58118	0.355207	0.427414	-0.17469	0.263835
KIDINS220	0.283358	0.496102	0.275208	0.062245	0.105283
KIF11	0.105367	-0.48435	0.079234	0.017261	-0.04072
KIF12	-0.23092	0.298336	0.279652	-0.02208	0.231966
KIF13A	0.114499	0.060851	0.056636	0.00571	0.168211
KIF13B	-0.35854	-0.32522	-0.48294	-0.14412	-0.39577
KIF14	0.130753	-0.35217	-0.37688	-0.18831	-0.21103
KIF17	0.194071	0.228438	0.236144	0.203637	0.23032

KIF18A METT5D1	-0.37764	-0.25818	-0.45622	-0.36176	-0.28396
KIF1A	0.012008	0.062243	0.030752	-0.0053	-0.01517
KIF1B	-0.03103	0.073436	0.079113	0.090464	0.22627
KIF21A	0.031795	0.195879	0.035889	0.059278	0.071527
KIF21B	0.039202	0.094068	0.11922	-0.09878	0.027938
KIF22	-0.15734	-0.00616	-0.48579	-0.09537	-0.19273
KIF23	0.011493	-0.18362	-0.16699	-0.16252	-0.24578
KIF23 RPLP1	0.066715	-0.31986	-0.50868	-0.02406	-0.34162
KIF25	0.698501	0.671283	0.913534	0.522201	0.69339
KIF27	-0.07619	-0.09925	-0.17571	-0.15591	-0.15044
KIF2A	0.107505	0.194789	0.201651	-0.05645	0.02294
KIF2B	0.625322	0.85	0.492557	0.605789	0.479354
KIF2C	-0.44006	-0.22215	-0.39507	-0.3779	-0.19685
KIF3A	0.027561	0.143986	0.005938	-0.08019	-0.1518
KIF3B	0.185085	0.328729	0.246688	0.375013	0.431824
KIF3C	0.160848	0.327294	0.424312	0.340109	0.291861
KIF5A	-0.17835	-0.06332	-0.22353	-0.18517	-0.12721
KIF5B	0.101497	0.040832	-0.01442	0.094353	0.149515
KIF6	0.309093	0.054889	-0.05949	0.05192	-0.06491
KIF7	0.307163	0.306436	0.244319	0.209155	0.367105
KIF9 KLHL18	-0.40637	-0.23913	-0.66996	-0.51758	-0.20936
KIFAP3	-0.31467	-0.86066	-0.74243	-0.75683	-0.78502
KIFC1	-0.08915	-0.00749	-0.16093	-0.05145	-0.10376
KIFC3	0.162358	0.231251	0.484641	0.168631	0.25769
KIN ATP5C1	-0.60431	-0.62628	-0.7496	-0.48654	-0.53617
KIR3DL2	0.425963	-0.36749	-0.20583	0.305426	0.350593
KIRREL	-0.17933	-0.57463	-0.86221	-0.5956	-0.37885
KIRREL2	-0.13283	-0.06534	-0.10595	-0.26746	-0.08795
KISS1R	0.132653	-0.11496	-0.07304	0.143689	0.058682
KIT	0.04412	0.263419	0.327421	0.127135	0.151859
KITLG	-0.13941	0.190533	-0.07655	-0.20072	-0.163
KL	0.181836	0.395	0.448743	0.202671	0.380621
KLB	0.044654	0.300356	0.57984	-0.44311	-0.47712
KLC1	-0.00521	-0.09057	-0.1251	0.046433	-0.07445
KLC3	0.212561	0.215404	0.357504	0.233049	0.295679
KLF1	0.356185	0.417976	0.48363	0.334288	0.472879
KLF10	-0.39176	-0.38487	-0.27232	-0.50411	-0.37973
KLF11	-0.2084	0.092632	0.127875	0.023636	0.198592
KLF12	-1.07561	-0.74086	-0.92818	-0.61789	-0.86748
KLF13	0.003229	0.177949	0.112818	0.166363	0.175147
KLF14	-0.10409	0.015861	-0.20076	-0.08936	-0.00546
KLF15	-0.16747	0.001248	-0.00631	-0.10212	-0.06518
KLF16	0.07116	0.00576	0.08118	-0.00069	0.075697
KLF17	0.620367	0.773437	0.733127	0.466065	0.549153
KLF2	0.000414	0.307664	0.297545	0.209458	0.270775
KLF4	-0.3576	-0.17502	0.132111	-0.09265	-0.11477
KLF5	-0.01775	-0.16841	-0.11412	-0.03548	0.15997
KLF7	-0.3634	-0.57531	-0.59527	-0.36248	-0.37352
KLF8	-0.0525	0.331883	-0.45129	0.292905	0.347561
KLF9	-0.25064	-0.20618	-0.21931	-0.45918	-0.34871

KLHDC1	0.206515	0.361671	0.019399	0.071658	0.1293
KLHDC2	-0.20659	-0.07164	-0.18255	-0.16169	-0.16973
KLHDC3 C6orf153	-0.17711	-0.13274	-0.20521	-0.19283	-0.20063
KLHDC4	0.256763	0.161725	0.237377	0.160317	0.243254
KLHDC5	0.185377	0.214373	0.2068	0.171106	0.207615
KLHDC6	-0.05741	0.055492	-0.2931	-0.4753	-0.70475
KLHDC7A	0.23218	0.211814	0.282376	0.197089	0.251478
KLHDC7B	-0.13007	-0.01418	0.259874	0.081531	0.17709
KLHDC8A	0.367398	0.533942	0.818913	0.37058	0.515375
KLHDC9	-0.18632	-0.09524	-0.00532	0.30039	-0.30585
KLHL1	0.092813	0.537244	0.55811	0.283561	0.28938
KLHL11	0.060323	0.118988	0.168641	0.088689	0.285165
KLHL12	-0.1718	0.058285	0.023768	-0.14355	-0.023
KLHL13	-0.46907	-0.32329	-1.20945	0.078977	-0.70543
KLHL14	-1.329	0.564368	0.619469	0.221476	0.365523
KLHL15	0.825585	1.022784	0.988783	0.750513	0.771597
KLHL2	0.282544	0.411233	0.314469	0.219253	0.287727
KLHL20	-0.63157	-0.78283	-0.69649	-0.69549	-0.52946
KLHL21	0.087123	0.280323	0.37392	0.063052	0.011336
KLHL22	-0.30429	-0.25045	-0.30313	-0.26886	-0.27968
KLHL23	-0.25237	-0.06413	0.040946	-0.07595	-0.12066
KLHL24	0.045204	0.272693	-0.01186	0.156138	-0.00133
KLHL25	0.018228	0.118867	-0.07711	-0.02995	-0.0601
KLHL26	-1.05459	-0.7356	-1.26422	-1.01604	-1.0159
KLHL28 KIAA0423	-0.82554	-1.08211	-0.63766	-0.74658	-0.91586
KLHL3	-0.72881	0.182008	-0.17507	-0.72131	-0.82799
KLHL30	0.357148	0.366148	0.590919	0.356355	0.369248
KLHL31	-0.10953	0.023921	-0.25551	-0.37457	-0.18644
KLHL32	0.089653	0.49714	0.085755	0.033907	0.134527
KLHL34	0.227824	0.365215	0.52942	0.294831	0.291015
KLHL4	-0.24711	-0.45232	-0.62071	-0.3565	-0.2682
KLHL6	0.623019	0.852532	0.873584	0.584756	0.724055
KLHL7	-0.11272	-0.28556	-0.25226	-0.14631	-0.17782
KLHL8	0.069017	0.083808	0.196722	-0.12319	0.066836
KLHL9	-0.0638	0.007167	-0.09181	0.218261	0.194677
KLK1	-0.00089	-0.49863	-0.50815	-0.17351	0.022755
KLK10	0.189715	-0.42775	-0.29114	-0.04393	-0.23013
KLK11	0.294685	-0.61116	-0.4055	-0.46153	0.008178
KLK12	0.215979	-1.07747	-0.97868	-0.27349	-0.14246
KLK13	-0.01108	-0.44197	0.011982	0.111856	0.039605
KLK14	-0.0727	-0.91118	-0.02316	-0.57406	0.139516
KLK15	0.218585	-0.93373	0.165459	0.121829	0.271724
KLK2	-0.00956	-1.99289	-1.41457	-0.65963	-0.1087
KLK3	0.116512	-1.11345	-0.58152	0.053011	0.203659
KLK4	-0.00396	-0.71439	-0.49042	-0.11613	-0.13525
KLK5	0.007376	-0.68085	-0.58523	-0.42488	-0.28213
KLK6	0.114012	-0.36839	-0.29618	-0.13319	0.156375
KLK7	-0.02538	-0.5178	-0.39494	0.107348	-0.11135
KLK8 KLK9	-0.13169	-0.85767	-0.62259	-0.14979	-0.17464
KLK9	-0.07217	-1.40034	-1.08697	-0.98354	-0.66178

KLKB1	-0.32066	-0.14043	-0.26145	-0.19643	-0.3812
KLRA1	0.184306	0.263481	0.115321	-0.2189	0.012779
KLRB1	-0.0107	-0.6995	-0.33368	-0.86997	-0.68109
KLRC1	-0.52811	-0.93602	-0.78217	-0.77065	-0.83507
KLRC2	-0.58242	-1.11661	-0.90544	-1.1543	-1.25841
KLRC3	-0.58309	-1.10361	-0.97954	-0.99179	-1.09114
KLRD1	0.261897	-0.39906	-0.44525	-0.45819	-0.37088
KLRF1	-0.11797	-0.5835	-0.14762	-0.5485	-0.46379
KLRG1	0.451233	0.301789	0.797235	-0.05703	-0.4685
KLRK1	0.071782	-0.43292	-0.30131	-0.369	-0.50173
KMO	0.052931	-0.30134	-0.31847	-0.5028	-0.46827
KNCN	0.088646	0.067691	0.155561	-0.01643	-0.04122
KNDC1	-0.14397	-0.16215	-0.02723	-0.19361	-0.23193
KNG1	-0.16187	-0.27969	-0.30677	-0.2642	-0.30077
KPNA1	0.221545	0.125952	0.339981	0.072239	0.290669
KPNA2	-0.5417	-0.66393	-0.97264	-0.86846	-0.83179
KPNA3	-0.01275	0.026209	-0.0869	-0.08602	0.0729
KPNA4	-0.32577	-0.19325	-0.27419	-0.38855	-0.25782
KPNA5	0.22014	0.07936	-0.36102	0.066127	-0.34485
KPNA6	0.066204	-0.08059	-0.21621	0.046185	-0.02802
KPNB1	-0.57212	-0.34144	-0.68157	-0.56243	-0.45017
KPRP	0.22779	-0.75659	-0.60102	-0.54975	-0.27043
KPTN	0.011928	0.307156	-0.25626	0.151366	0.122279
KRAS	-0.16058	-0.10768	-0.34477	-0.05416	-0.20886
KRBA1	-0.07611	-0.00961	0.022975	-0.07723	0.122161
KRBA2	0.085233	0.225677	0.243944	-0.07547	0.248188
KRCC1	0.312607	0.370631	0.247681	0.241686	0.247402
KREMEN1	0.158928	0.31168	0.407596	0.200088	0.405204
KREMEN2	-0.33796	-0.16744	-0.26268	-0.27022	-0.30969
KRI1 CDKN2D	-0.09601	-0.04398	0.015677	0.081037	-0.25504
KRIT1 ANKIB1	-0.39129	-0.30573	-0.47646	-0.28773	-0.20868
KRR1	0.04772	0.211309	-0.14851	-0.37487	-0.5445
KRT1	0.017252	-0.95866	-1.21035	-1.12848	-0.96511
KRT10	-0.3479	-0.56607	-0.26357	0.087286	-0.32957
KRT10 TMEM99	-0.05183	0.135099	-0.39797	-0.8744	-0.23946
KRT12	0.42175	0.371484	-0.41803	-0.52851	-0.11023
KRT13	0.56645	0.895034	0.936392	-0.19059	0.750502
KRT14	0.402143	0.547409	0.458233	0.262959	0.568834
KRT15	0.183361	0.303946	0.310168	-0.71624	0.20056
KRT16	0.221803	0.124189	0.190205	0.036586	0.273131
KRT17	0.138891	0.293104	0.346262	-0.03044	0.163862
KRT18P19	0.49136	0.598219	0.708459	0.375904	0.598744
KRT19	0.333861	0.590612	0.707157	0.348718	0.416732
KRT2	0.157812	0.083152	0.030238	-0.02578	0.165793
KRT20	0.361256	0.314187	-0.462	-0.30795	-0.0577
KRT222P	0.153203	-0.32557	-0.57887	-0.37905	-0.71516
KRT23	0.345996	0.031591	-0.92129	-0.68398	-0.52895
KRT24	0.116856	0.04952	-0.09398	-0.06223	-0.11279
KRT25	0.207365	-0.35804	-0.62774	-0.52429	-0.53204
KRT26	0.432303	-0.1406	-0.97875	-0.82642	-0.75547

KRT27	0.165365	0.588613	-0.52867	-0.33353	-0.10376
KRT28	-0.41057	-0.80503	-0.79021	-0.74374	-0.99249
KRT3	0.354371	0.259676	0.184606	0.107897	0.213519
KRT31	0.803098	0.910817	0.573494	0.250976	0.577333
KRT32	0.147847	0.301647	-0.14859	-0.28669	0.025913
KRT33A	0.137365	0.18058	-0.19145	-0.08656	0.021847
KRT33B	0.301553	0.105256	0.034248	-0.0362	0.147428
KRT34	0.437976	0.430004	-0.21699	-0.04282	0.103809
KRT35	0.436196	0.44371	0.303326	-0.32626	0.345096
KRT36	0.441418	0.578365	0.441919	-0.34079	0.274988
KRT37	0.098449	0.15175	-0.12276	-0.3342	0.037118
KRT38	0.403819	-0.08725	-0.57722	-0.82567	-0.15962
KRT39	0.226962	0.114159	-0.44422	-0.27238	-0.17808
KRT4	0.396996	0.257782	-1.1519	-0.65551	-0.10456
KRT5	0.309002	0.434048	-0.1971	-0.11092	0.132846
KRT6A	0.096235	0.339493	-0.63909	-0.25871	0.014869
KRT6B	0.229976	0.408145	-0.17735	-0.03941	0.093009
KRT6C	0.314493	0.472995	-0.47048	-0.24804	-0.0985
KRT7	0.25582	0.657467	0.570441	0.432074	0.493693
KRT71	0.263349	0.166927	-0.63937	-0.57021	-0.02386
KRT72	-0.03156	0.27791	0.017832	0.121164	0.103732
KRT73	-0.03789	-0.08542	-1.02388	-0.4277	-0.17733
KRT74	0.086646	0.119444	-0.4768	-0.16264	0.048937
KRT75	0.407877	0.804272	-0.26118	0.272865	0.341161
KRT76	0.260062	-0.21075	-0.40117	-0.66133	-0.2998
KRT77	0.212354	-0.34478	-0.32115	-0.42966	-0.33407
KRT78	0.351736	0.283669	0.047498	-0.15303	0.139711
KRT79	0.202472	0.196518	0.163622	-0.01015	0.055458
KRT80	0.21258	0.302514	0.367106	0.206405	0.299362
KRT81	0.258173	0.473401	0.532104	0.299365	0.422351
KRT82	0.187918	0.200167	-0.41328	-0.13934	0.166032
KRT83	0.193426	0.474236	0.434188	0.051875	0.242985
KRT84	0.201611	0.469145	-0.30483	-0.30882	0.043591
KRT85	0.256423	0.301447	0.375219	0.178585	0.351696
KRT86	0.418007	0.843314	0.6892	0.408561	0.494243
KRT8P9	0.264057	0.261627	0.457938	0.126214	0.427474
KRT9	0.145808	0.379909	0.484039	-0.0335	0.488804
KRTAP11-1	-0.18626	-1.31822	-1.11942	-0.97291	-0.87312
KRTAP13-1	-0.27882	-1.52777	-1.26698	-1.13873	-1.39441
KRTAP13-2	-0.10691	-1.42471	-1.01118	-0.69424	-1.36591
KRTAP13-3	-0.65122	-1.49012	-1.21146	-0.97659	-1.25955
KRTAP13-4	-0.31492	-1.08679	-1.1413	-0.81713	-1.11624
KRTAP15-1	0.314608	-0.57484	-0.49557	-0.04973	-0.24544
KRTAP17-1	0.429142	0.637434	0.407701	0.380283	0.538345
KRTAP19-1	0.195811	-1.39172	-1.16737	-0.36543	-0.54881
KRTAP19-2	-0.00929	-0.81554	-0.52684	-0.70535	-0.38233
KRTAP19-3	0.065697	-0.84665	-0.62303	-0.28214	-0.16116
KRTAP19-4	0.041867	-1.02839	-0.7308	-0.66673	-0.49893
KRTAP19-5	0.570466	-0.90036	0.332988	0.20046	0.259563
KRTAP19-6	0.03438	-0.88089	-0.68396	-0.50995	-0.57588

KRTAP19-7	0.094623	-0.86141	-0.98099	-0.59432	-0.6495
KRTAP20-2	-0.23034	-0.92766	-0.87855	-0.63469	-0.78529
KRTAP21-1	-0.33771	-0.86145	-1.16304	-1.21587	-1.0494
KRTAP21-2	-1.0152	-0.86077	-0.94	-0.92618	-1.0557
KRTAP23-1	-0.15068	-0.87374	-0.98762	-0.5316	-0.6429
KRTAP2-4	0.596902	0.610466	-0.09067	0.232326	0.588114
KRTAP26-1	0.071104	-0.77299	-0.68835	-0.09289	-0.47521
KRTAP3-1	0.359275	0.247267	-1.03281	-0.61853	-0.2179
KRTAP3-2	0.218691	0.034182	-1.39493	-1.09288	-0.51176
KRTAP3-3	-0.27004	-0.59443	-0.93543	-0.80906	-0.97678
KRTAP4-1	0.188226	-0.23731	-0.77186	-0.66079	-0.7644
KRTAP4-12	0.01378	-0.20369	-0.5366	-0.40825	-0.33719
KRTAP4-14	0.033068	-0.38594	-1.15281	-0.78084	-1.10676
KRTAP4-2	0.373226	-0.15702	-1.99037	-1.24691	-0.28969
KRTAP4-4	-0.41971	-0.91866	-1.31959	-1.27091	-1.08803
KRTAP4-5	0.488989	-0.07013	-1.33407	-0.93991	-1.29081
KRTAP5-1 AP006285.2	0.076002	0.033925	0.190082	-0.20575	-0.00642
KRTAP5-10	-0.24647	-0.5798	-0.04904	-0.50132	-0.6936
KRTAP5-11 AP000867.5	0.044342	-0.15925	0.244015	-0.28601	-0.59448
KRTAP5-2	0.034888	-0.52758	0.141481	-0.56003	-0.47248
KRTAP5-6	0.299525	-0.49597	0.244744	-0.47086	-0.22618
KRTAP5-7	0.079338	-0.32739	-0.01047	-0.51213	-0.37367
KRTAP5-9	0.417607	0.205741	0.511478	0.118288	0.210603
KRTAP6-1	-0.42284	-0.92124	-0.80163	-0.54144	-0.46288
KRTAP6-2 KRTAP22-1	0.034094	-0.5259	-0.40184	-0.07236	-0.11098
KRTAP6-3	0.130952	-0.89791	-0.75804	-0.43409	-0.40422
KRTAP8-1	0.34087	-1.19716	-1.15226	-0.58612	-0.19456
KRTAP9-2	0.257372	-0.492	-1.84944	-1.51456	-1.79713
KRTAP9-3	-0.06849	-0.89494	-1.41892	-1.16395	-1.27474
KRTAP9-5	-0.14164	-0.86759	-1.2002	-1.07641	-1.2745
KRTCAP2 TRIM46	-0.7095	-0.9558	-1.03335	-0.82967	-0.78261
KRTDAP	0.397945	0.079558	-0.34462	-0.32356	0.563679
KS	0.117844	-0.02695	-0.18656	-0.25928	0.010819
KSP37 PROM1	0.353257	0.282166	0.464005	0.322998	0.233926
KSR1	0.263784	0.408538	0.459568	0.33295	0.424132
KSR2	0.271897	0.401826	0.468689	0.262276	0.267978
KTELC1	-0.0225	-0.07357	-0.06419	-0.17711	-0.1546
KY	-0.01829	0.150892	0.330639	0.399242	0.221565
KYNU	-0.47505	-0.52925	-0.66247	-0.52873	-0.37129
L1TD1	0.393147	0.621628	0.842124	0.592144	0.699519
L2HGDH ATP5S	-0.20719	-0.22745	-0.51579	-0.40581	-0.33594
L3MBTL	0.028421	0.069855	0.106745	0.032914	0.063021
L3MBTL2	0.073905	0.170484	0.164121	-0.1242	-0.23867
L3MBTL3	0.288534	0.574079	0.623223	0.459486	0.630888
L3MBTL4	0.197634	0.407362	0.158906	0.246089	0.427931
LACE1	0.181744	0.244644	-0.03664	0.145616	0.074868
LACRT	-0.84144	-1.40229	-1.64489	-1.19934	-1.34608
LACTB	-0.18149	0.117744	0.169996	-0.27133	-0.12272
LACTB2 AC020987.8	0.021242	0.313081	0.20307	-0.03343	0.103389
LAD1	0.34463	0.532968	0.676786	0.410333	0.5591

LAG3	0.358672	0.461812	0.460604	0.342069	0.465089
LAGE3	0.206318	0.092633	0.087059	-0.09764	0.00074
LAIR1	0.53619	-0.51206	-0.36489	-0.14901	0.393382
LAIR2	0.737729	-0.29414	0.368192	0.21259	0.425461
LALBA	0.033235	0.264198	-1.04998	-0.49119	-0.72043
LAMA1	0.127775	0.195418	0.170674	0.16698	0.261951
LAMA2	0.098696	0.302595	-0.01979	0.308752	0.144155
LAMA4	0.138261	0.622701	0.299947	0.356574	0.250326
LAMA5	0.152256	0.118071	0.238525	0.116118	0.200036
LAMB1	-0.25614	0.102219	-0.1005	0.006596	0.086173
LAMB3	0.13119	0.312658	0.204573	0.215684	0.073172
LAMB4	0.420735	0.740608	0.410712	0.181126	0.403151
LAMC1	-0.05988	-0.23187	-0.27447	-0.11061	0.000425
LAMC2	0.328062	0.775398	0.533665	0.44809	0.439987
LAMC3	0.172613	0.08145	0.438944	0.131631	0.321346
LAMP1	0.149142	0.175518	0.099023	0.109462	0.281696
LAMP2	0.255798	-0.21664	-0.19383	-0.25506	-0.45038
LAMP3	0.441235	0.502956	0.398535	0.371454	0.432597
LANCL1	-0.44116	-0.35816	-0.49908	-0.36253	-0.47124
LANCL2	0.256541	0.151687	0.165625	0.120728	-0.02374
LAP3	0.041366	0.092005	0.098459	0.06815	0.162483
LAPTM4A	-0.91795	-0.89109	-1.0011	-0.71035	-0.88206
LAPTM4B	0.304448	0.019296	0.11866	-0.08472	0.129616
LAPTM5	0.112479	0.342293	0.358966	0.204416	0.28337
LARGE	0.03233	0.038552	0.114313	0.127092	0.083863
LARP2	0.049423	0.106293	0.142651	0.012864	0.074173
LARP4	-0.25436	-0.1907	-0.63736	-0.48415	-0.51151
LARP5	0.498288	0.629371	0.480351	0.229258	0.382543
LARP6	0.183447	0.306067	0.423198	0.16261	0.240938
LARS	-0.17012	-0.02311	-0.19236	-0.25509	-0.18474
LARS2	-0.14596	0.070172	-0.41152	-0.06488	-0.1013
LAS1L	0.077517	-0.00043	-0.56259	-0.13409	-0.15283
LASP1	-0.1993	-0.28045	-0.2621	-0.15672	-0.16749
LASS2	0.163852	0.415323	0.344504	0.120677	0.209989
LASS3	0.441993	0.464882	0.378261	0.323793	0.422448
LASS5	0.236924	0.232753	0.409167	0.078515	0.127407
LASS6	-0.08789	0.012082	-0.04028	-0.14685	-0.02582
LAT2	0.295778	0.45731	0.752172	0.138342	-0.03125
LATS1	0.113081	0.098092	-0.12333	-0.02457	-0.03552
LATS2	0.236576	0.245094	0.517314	0.145558	0.251686
LAX1	0.51451	0.462803	0.674474	0.298543	0.187882
LAYN	0.23855	0.489349	0.487808	-0.008	0.474602
LBP	0.082514	0.154032	0.219435	0.020085	0.105152
LBR	-0.20697	-0.26631	-0.26341	-0.30842	-0.27124
LBX1	-0.45624	0.000412	-0.0336	0.210912	0.03787
LBX2	0.344973	0.576377	0.673913	0.379382	0.56497
LBXCOR1	-0.2767	-0.29133	0.653382	0.139258	0.034351
LCAT SLC12A4	0.339591	0.538349	0.515857	0.316	0.389508
LCE1A	-0.16596	-1.37998	-1.45646	-1.33758	-1.19411
LCE1C	-0.35572	-0.91528	-0.5371	-0.64362	-0.50751

LCE1D	-0.14167	-0.88073	-0.75677	-0.13398	-0.46053
LCE1E	-0.67281	-1.08762	-1.15041	-0.92855	-0.65525
LCE1F	-0.09856	-1.48676	-1.37459	-1.27063	-0.82259
LCE2A	-0.48995	-0.72241	-0.55356	-0.6405	-0.50023
LCE2B	-0.46047	-1.12487	-1.05353	-0.97298	-0.7596
LCE2C	-0.37209	-0.74974	-1.07954	-0.90221	-0.83435
LCE2D	-0.34265	-0.96332	-0.25115	-0.61392	-0.59489
LCE3A	0.236525	-0.65283	-0.4076	0.000258	0.187341
LCE3B	-0.10429	0.035192	-0.76705	-0.7557	-0.42315
LCE3C	-0.1271	0.19843	-0.67772	-0.68555	-0.48072
LCE3D	0.00964	-0.5673	-0.35118	-0.45911	-0.29208
LCE3E	-0.48385	-1.14347	-1.0675	-0.84959	-0.87067
LCE4A	-0.03685	-1.58087	-1.16894	-0.97322	-0.92627
LCE5A	-0.04195	-1.7512	-1.45157	-1.33237	-1.03048
LCK	0.351505	0.437568	0.44662	-0.0059	0.036449
LCMT1	-0.41198	-0.42135	-0.58156	-0.38122	-0.44493
LCMT2 ADAL	-0.48741	-0.32889	-0.41477	-0.36023	-0.45434
LCN1	0.443664	0.2096	0.572163	0.452606	0.639335
LCN10	0.186973	0.284349	0.455309	0.135465	0.324841
LCN12	0.170879	0.199591	0.271755	0.20147	0.119165
LCN2	0.217589	0.112134	0.270939	0.242504	0.406229
LCN6	0.362734	0.28146	0.624904	0.286319	0.410274
LCN8	0.143096	0.147832	0.204527	0.155671	0.318994
LCN9	0.165305	0.015481	0.275473	0.239692	0.064984
LCOR	-0.39831	-0.5267	-0.42754	-0.37801	-0.30276
LCORL	-0.04968	-0.2355	-0.09148	-0.07366	-0.06146
LCP1	0.284899	0.224265	0.173628	-0.0406	-0.30623
LCP1 AL139801.17	0.320062	-0.31222	-0.6391	-0.12004	-0.50954
LCP2	0.407304	0.492317	0.350194	0.353087	0.25185
LCT	0.879951	1.107459	0.886526	0.641304	0.813463
LCTL	-0.23691	0.225609	0.270173	0.218207	0.25418
LDB1	-0.46536	-0.55735	-0.5752	-0.4294	-0.4408
LDB2	-0.14682	0.427235	0.134496	-0.37789	-0.21102
LDB3	0.234166	0.494092	0.509131	0.339861	0.136481
LDHA	-0.04664	0.037027	0.048977	-0.14451	0.492299
LDHAL6A	0.278567	0.524893	0.51778	0.214692	0.219562
LDHB	-0.09352	0.259267	-0.07963	-0.16117	-0.17847
LDHC	0.478818	0.915916	0.877066	0.644741	0.817405
LDHD	0.232579	0.19051	0.20125	-0.07694	0.069503
LDLR	-0.26392	-0.2309	-0.39385	-0.36561	-0.24744
LDLRAD1	0.35801	0.318254	0.444994	0.188486	0.42683
LDLRAD2	-0.66383	-0.64044	-0.88225	-0.34063	-0.59949
LDLRAD3	-0.04284	0.112879	-0.07404	-0.07331	0.072484
LDLRAP1	-0.00891	0.076848	0.047729	0.142277	0.028555
LDOC1	0.078176	0.059865	0.143247	0.104564	0.018565
LDOC1L	0.084634	0.078552	0.236064	0.188488	0.138093
LEAP2	0.210544	-0.22553	-0.08253	-0.30894	-0.10323
LECT1	0.268311	0.630828	0.511824	0.372426	0.396793
LECT2	0.060104	-0.04093	-0.14961	-0.45273	-0.42186
LEF1	-0.33395	0.126346	-0.06593	0.243338	0.109158

LEFTY1	0.311487	0.408327	0.427972	0.298837	0.493396
LEFTY2	-0.03242	0.15937	-0.01616	-0.02082	0.051976
LEKR1	0.553229	-0.0673	0.369297	0.478144	0.401161
LELP1	-0.4957	-1.7065	-1.49244	-1.27098	-1.11192
LEMD1	0.080446	-0.40809	0.025826	-0.35817	-0.20292
LEMD2	0.326554	0.393305	0.558886	0.321127	0.363653
LEMD3	0.008135	-0.13613	-0.19225	-0.11737	-0.09764
LENG1 TMC4	-0.94215	-0.80019	-1.14761	-0.74032	-0.95928
LENG4 TSEN34	-0.43629	-0.43796	-0.47678	-0.3973	-0.35077
LENG8	-0.00091	-0.13081	-0.0479	-0.08033	0.018572
LENG9	-0.36897	-0.31928	-0.27347	0.337163	-0.45682
LEO1	-0.14818	-0.23403	-0.46866	-0.2067	-0.11893
LEP	0.340643	0.403773	0.681079	0.259651	0.356167
LEPRE1 C1orf50	-0.07345	-0.0138	-0.15443	-0.13356	-0.06269
LEPREL1	0.275215	0.206657	0.393627	0.353874	0.304189
LEPREL2 GNB3	0.197854	0.495161	0.562981	0.063501	0.49602
LEPROT	-0.76589	-0.39293	-0.7942	-0.74439	-0.1836
LEPROTL1	-0.0557	-0.12117	-0.34307	-0.23248	-0.31887
LETM1	0.221176	0.259602	0.14505	0.228783	0.322846
LETM2	-0.05194	-0.45598	-0.44896	-0.48845	-0.48344
LETMD1	-0.06158	-0.10744	-0.10309	-0.22517	-0.36283
LGALS1	-0.12155	-0.02636	-0.32653	0.277265	-0.069
LGALS12	0.257905	0.369158	0.128373	0.027049	-0.06842
LGALS13	-0.39841	-1.75165	-1.73321	-1.12118	-1.39983
LGALS14	-0.44981	-0.74858	-0.7445	-0.5953	-0.67976
LGALS2	0.308956	0.188977	0.361957	-0.02983	0.143783
LGALS3BP	0.226846	-0.28263	0.189714	0.245224	0.018896
LGALS4	0.284207	0.529649	0.512215	0.350374	0.456472
LGALS9	0.293948	0.434842	0.392284	0.232808	0.342634
LGI1	0.336022	0.116677	-0.3484	-0.46448	-0.37411
LGI2	0.032679	-0.1747	-0.14634	-0.0279	0.075066
LGI3	-0.14523	0.351595	0.305105	-0.01541	0.39065
LGI4	-0.14552	-0.22253	-0.17393	-0.34829	-0.21248
LGMN	-0.44553	-0.42334	-0.82275	-0.53395	-1.07261
LGR4	0.187064	0.585576	0.275677	0.240175	0.42377
LGTN	0.118963	0.096608	0.329985	0.228983	0.214878
LHB	0.212084	-0.16033	0.440452	0.379003	0.488397
LHFP	-0.5115	-0.35292	-0.58519	-0.31008	-0.40346
LHFPL1	-0.15114	-0.17388	-0.15248	0.102383	-0.16902
LHFPL2	0.357382	0.414356	0.362121	0.347185	0.40193
LHFPL3	-0.09358	0.297881	0.098878	0.196903	-0.11087
LHFPL4	-0.07761	-0.07613	0.014593	0.023748	0.045748
LHFPL5	0.795683	0.166754	0.375354	0.302776	0.156319
LHPP	0.293804	0.28163	0.186254	0.19767	0.176982
LHX1	-0.33431	-0.21819	-0.07048	0.26518	-0.35544
LHX2	0.152734	0.054164	0.220834	0.157543	0.313556
LHX3	0.162276	0.25087	0.445907	0.328037	0.427786
LHX4	-0.27964	-0.31472	-0.44442	-0.33501	-0.12823
LHX5	-0.0718	-0.07988	0.452726	0.29356	0.362057
LHX6	-0.22858	0.106195	-0.05222	0.056994	-0.00589

LHX8	-0.19443	1.05063	1.078442	0.716774	0.072081
LIF	-0.12008	-0.07021	0.091334	0.000235	0.216284
LIFR	0.599867	0.805938	0.639009	0.48205	0.48457
LIG1 AC011466.6	-0.63558	-0.41284	-0.93455	-0.36771	-0.48223
LIG3	-0.02904	0.188393	0.133545	-0.08846	-0.0035
LIG4	-0.30878	-0.06204	-0.54374	-0.05855	-0.19733
LIG4 ABHD13	0.093086	0.110285	0.210869	0.035116	0.048436
LILRA1	0.135794	-1.87177	-1.815	-0.78201	-1.00228
LILRA2	0.079772	-0.52382	-0.45338	-0.41044	-0.44788
LILRA3	0.054174	-1.18512	-1.12046	-0.78975	-0.86795
LILRA4	0.34459	-0.37819	-0.13282	-0.17485	0.06532
LILRA5	0.22574	-1.18677	-1.18815	-0.2825	-0.49853
LILRB1	-0.34532	-0.56077	-0.54796	-0.30911	-0.54653
LILRB2	0.315026	-1.95314	-1.74928	-0.97492	-1.43079
LILRB4	0.287288	-0.73425	-0.51256	-0.06824	-0.19863
LILRB5	0.241595	-1.08272	-0.95802	-0.85194	-0.60375
LIM2	0.317447	0.450591	0.5186	0.180188	0.262909
LIMA1	0.322064	0.232115	0.035751	-0.03988	0.16361
LIMCH1	0.024217	-0.01606	0.190039	0.109556	-0.02025
LIMD1	0.341464	0.629654	0.560942	0.343066	0.549864
LIMK1	-0.12273	-0.11031	-0.10344	-0.10305	0.041818
LIMS1	-0.21055	-0.9132	0.20355	0.190009	0.138919
LIMS3	0.151326	-0.68719	-0.17768	0.149754	0.011905
LIN28	-0.42	0.211544	0.372219	0.196733	0.34932
LIN28B	-0.71476	0.974396	0.409101	-0.6615	-0.91047
LIN37	0.210517	0.163609	-0.07141	0.026981	0.118453
LIN54	-0.58064	-0.68728	-0.58484	-0.49419	-0.74931
LIN7A	-0.02045	0.125748	-0.01578	-0.15654	-0.21732
LIN7B	0.042889	0.325931	-0.02881	0.181524	0.062679
LIN7C	-0.41749	-0.46765	-0.71451	-0.33788	-0.18803
LIN9	0.133745	0.173223	0.069409	-0.07179	0.162164
LINGO1	-0.14795	-0.11929	-0.12229	-0.14102	-0.09005
LINGO4	0.074175	0.293847	0.424402	-0.00041	0.063579
LINS1 ASB7	-0.44506	-0.43084	-0.37433	-0.35307	-0.31521
LIPA IFIT5	-0.07931	0.06695	-0.08601	-0.06123	-0.12244
LIPC	0.435001	0.832172	0.659538	0.578402	0.664977
LIPE	0.462502	0.304736	0.309102	-0.16794	0.287467
LIPF	-0.09236	-0.02946	-0.13566	-0.27491	-0.34549
LIPG	-0.14911	0.113631	0.188653	0.193001	0.101649
LIPH	0.438939	0.647917	0.735758	0.245325	0.324886
LIPI	-0.56333	-0.60788	-0.55122	-0.53069	-0.58261
LIPJ	0.565444	0.650278	0.798977	0.289863	0.40733
LITAF	0.079236	0.165458	0.194445	0.198083	0.32857
LIX1	-0.25526	-0.2253	-0.75888	-0.44543	-0.5858
LIX1L	-0.54469	-0.78982	-0.74533	-0.73727	-0.54443
LL22NC03-5H6.5	0.172962	0.1649	-0.05393	-0.09774	-0.05219
LLGL1	0.009525	0.328665	0.367726	0.184844	0.349148
LLGL2	-0.06361	0.002501	0.260975	0.140666	0.115298
LMAN1	-0.16596	0.076362	-0.23519	-0.13034	-0.04777
LMAN1L	0.364599	0.316919	0.438737	0.277825	0.447212

LMAN2	-0.08931	-0.0082	-0.1681	-0.11476	-0.06823
LMAN2L	-0.36878	-0.2323	-0.60119	-0.45369	-0.58006
LMBR1	-0.31769	-0.20494	-0.51212	-0.22182	-0.19829
LMBR1L	-0.10614	-0.0618	-0.26514	-0.26157	-0.07584
LMBRD1	-0.25898	-0.19874	-0.19615	-0.27727	-0.27472
LMBRD2 SKP2	-0.76525	-0.61278	-0.55117	-0.62898	-0.76402
LMCD1	0.219403	-0.24964	-0.65043	0.168323	0.00263
LMF1	0.083658	0.106491	0.058198	0.104155	0.222835
LMNA	-0.34274	-0.66825	-0.72933	-0.25131	-0.53954
LMNB1	-0.35205	-0.33841	-0.29733	-0.28593	-0.46571
LMNB2	0.100706	0.000705	0.065231	-0.13587	-0.10156
LMO1	-0.21271	-0.17494	-0.02595	-0.08732	-0.03686
LMO2	0.509346	0.605638	0.675564	0.335417	0.513376
LMO3	0.052215	0.394501	-0.28036	-0.12209	-0.04623
LMO4	0.008691	0.085028	0.195087	-0.15273	0.152138
LMO7	0.156512	0.236766	0.316837	0.066236	0.16404
LMOD1	-0.09056	-0.24397	-0.03887	0.10405	-0.55948
LMOD3	0.131761	0.054925	-0.07848	-0.13175	-0.16281
LMTK2	-0.02042	-0.18035	-0.22942	-0.17587	-0.36496
LMX1B	0.002993	0.138205	0.226222	0.211302	0.225705
LNX1	0.206775	0.389936	0.606983	0.326791	0.574301
LOH11CR2A	-0.33264	-0.17708	-0.32148	-0.30652	-0.47761
LOH12CR2. LOH12CR1	0.258799	0.414211	0.254246	0.114264	0.110148
LONP1 TMEM146	0.075055	-0.05731	0.016872	-0.00281	0.041919
LONRF2	0.210732	0.566686	0.427013	-0.28133	0.217568
LONRF3	0.14722	0.023702	0.332052	0.224766	0.047576
LOR	0.017476	-0.72185	-0.72774	-0.54601	-0.19884
LOX	-0.69801	0.359736	-0.39172	-0.64245	0.166153
LOXHD1	0.647947	0.287689	0.458874	0.309259	-0.16736
LOXL1	-0.01726	0.300126	0.376449	-0.08087	0.157294
LOXL2 ENTPD4	0.177571	0.378558	0.209435	0.212897	0.321205
LOXL3 DOK1	-0.32397	-0.23164	-0.4519	-0.01412	-0.00783
LOXL4	0.086889	-0.0277	0.314627	0.172548	0.370336
LPA	0.010374	-0.93712	-0.94614	-0.79567	-0.61239
LPAL2	0.317209	-0.06389	-0.21825	-0.36791	-0.43719
LPAR2	0.232215	0.350861	-0.02947	0.110833	0.458543
LPAR5	0.302831	0.678431	0.697231	0.417444	0.545732
LPGAT1	0.201414	0.113634	0.264992	-0.32634	0.24817
LPHN1	-0.07546	0.010085	0.171897	-0.06669	-0.08745
LPHN2	-0.47396	-0.36007	-0.38787	-0.29223	-0.16112
LPHN3	0.63991	0.631213	0.992142	0.421639	0.582979
LPIN1	0.337227	0.368694	0.40044	0.32348	0.378831
LPIN2	0.126132	0.468989	0.53365	0.120721	0.138944
LPIN3	0.1317	0.530826	0.606169	0.221211	0.018063
LPL	0.362463	0.241736	-0.35332	-0.37448	0.201274
LPO	0.044015	-1.27926	-1.5085	-1.60158	-0.92473
LPP	-0.12525	0.451725	-0.16898	0.218209	0.314365
LPXN	0.236102	0.214746	0.073979	-0.21178	0.104234
LRAP	0.271403	0.150852	-0.16784	-0.19313	-0.09954
LRAT	-0.28207	0.047856	0.304748	0.452879	0.390097

LRCH1	-0.11805	-0.13783	-0.34687	-0.15977	-0.22755
LRCH3	0.076095	0.058732	0.204203	0.263673	0.120656
LRCH4 FBXO24	-0.39293	-0.21771	-0.42064	-0.02511	-0.05775
LRDD	0.112588	0.073251	0.128458	0.138244	0.198516
LRFN2	-0.04898	0.122786	0.031587	0.021662	0.035116
LRFN3	0.012348	0.126786	0.148819	0.109411	-0.06786
LRFN5	-1.47241	-1.32939	0.359191	0.397244	-0.04082
LRG1	0.31719	-0.20634	0.44825	0.180785	0.386641
LRGUK	-0.3246	-0.16916	-0.70653	0.035719	-0.55791
LRIG1	-0.24409	0.129793	0.311735	0.068906	0.13485
LRIG2	-0.25044	-0.11019	-0.28272	-0.38593	-0.32092
LRIG3	0.086526	0.043725	0.06852	-0.05052	0.079978
LRIT3	0.327108	0.450359	0.36388	0.146044	0.271933
LRMP	0.440074	0.498684	0.412937	0.190669	-0.11739
LRP1	-0.38777	-0.45004	-0.44612	-0.2673	-0.41013
LRP10	-0.49476	-0.1296	-0.41395	0.092666	-0.39244
LRP11	0.111751	0.258683	0.086653	0.004918	0.108866
LRP12	-0.23936	-0.21792	-0.3112	-0.3058	-0.32601
LRP2	-0.00301	-0.08776	-0.27763	0.169729	-0.10317
LRP2BP	0.229917	0.324757	0.369307	0.142438	0.219725
LRP3	-0.09345	-0.10955	-0.11987	-0.08046	-0.04088
LRP4	-0.19863	0.291539	0.108986	0.051525	0.34158
LRP5	0.067081	0.195044	0.322295	0.226854	0.239455
LRP5L	0.394319	0.487768	0.409644	0.527572	0.58164
LRP6	0.0633	-0.22315	-0.01281	-0.12947	0.083718
LRP8	-0.06934	0.130794	0.004024	-0.17655	0.215471
LRPAP1	0.007557	0.019906	0.322366	0.182622	0.155298
LRPPRC	-0.06224	-0.07944	0.018579	0.044155	-0.01204
LRRC1	-0.28146	-0.28585	-0.14461	-0.12243	-0.23719
LRRC14	0.188059	0.447222	0.505774	0.366663	0.247857
LRRC15	0.132899	0.408918	0.799835	0.533835	0.517331
LRRC16	-0.28311	-0.02164	-0.24132	-0.0781	-0.15077
LRRC16B	0.18389	0.284759	0.232129	0.248576	0.364544
LRRC2 TDGF1	0.077703	0.384824	0.621913	0.28619	0.326236
LRRC20	-0.02396	0.02806	-0.08074	-0.04254	-0.15108
LRRC21	0.214632	0.195428	0.39904	0.168422	0.165397
LRRC22	-0.05733	-0.48737	0.064318	-0.34581	-0.50059
LRRC23	-0.3572	-0.26093	-0.76635	-0.60188	-0.59758
LRRC23 ENO2	0.2503	0.463139	-0.06575	0.197557	-0.1372
LRRC26	0.331675	0.223786	0.447942	0.304724	0.408038
LRRC29 TMEM208	-0.17728	-0.31237	-0.48545	-0.27205	-0.29388
LRRC3	-0.04956	0.164025	-0.07457	0.232279	0.18885
LRRC31	-0.29515	-0.08695	-0.33581	-0.47822	-0.83916
LRRC32	0.342677	0.310101	0.68587	0.33097	0.409512
LRRC33	0.322945	0.516786	0.617677	0.302631	0.417729
LRRC34	0.298865	0.259496	-0.02658	0.031418	-0.15897
LRRC37A2	0.321769	0.628079	0.182244	0.22713	0.146236
LRRC37A3	0.014328	-0.03538	0.053644	-0.1297	0.14948
LRRC37B	0.412796	0.530496	0.727398	0.495773	0.674207
LRRC37B2.	0.673846	0.93253	0.840235	0.541116	0.736615

LRRC39	0.096926	0.133976	-0.02674	-0.44801	0.077583
LRRC3B	-0.28906	0.202029	-0.02247	0.22007	0.350902
LRRC40 SFRS11	-0.66117	-0.61748	-0.69334	-0.69603	-0.41254
LRRC41 UQCRH	-0.22229	-0.72868	-0.42016	-0.43583	-0.29368
LRRC43 B3GNT4	0.170375	0.134444	0.158587	0.072407	0.053576
LRRC44 TNNI3K	0.38455	-0.10195	-0.39659	0.039538	-0.10997
LRRC45 RAC3	-0.00636	-0.04162	0.025601	-0.06861	-0.01094
LRRC47	0.331093	0.417769	0.380943	0.370059	0.369695
LRRC4C	-0.23573	-0.22923	-0.37203	-0.37705	-0.48632
LRRC51	0.210331	0.313168	0.065798	0.247111	0.115707
LRRC52	-0.41674	-0.39576	-0.68205	-0.08026	-0.45881
LRRC55	0.099031	0.219098	0.45371	0.130736	0.448152
LRRC57 CEP27	-0.28239	-0.47118	-0.70405	-0.297	-0.52362
LRRC59	0.178728	0.142052	0.130996	0.00962	-0.03688
LRRC6	0.325384	-0.0931	0.000572	-0.0075	0.063357
LRRC7	0.618048	1.025462	1.100242	0.55679	0.596477
LRRC8B	0.121275	0.376206	0.038274	-0.22287	0.252649
LRRC8C	0.011102	-0.14112	-0.00148	-0.1316	0.06912
LRRC8D	0.046152	0.179242	0.028079	0.073573	0.076294
LRRC8E	0.336082	0.246912	0.643315	0.332053	0.323932
LRRC9.	-0.37372	-0.01447	0.219456	-0.27613	-0.05374
LRRCC1	-0.52453	-0.26452	-0.63332	-0.22315	-0.48409
LRRFIP2	-0.74484	-0.22247	-0.70619	-0.71476	-0.3676
LRRIQ1	-0.44972	-0.39196	-0.4135	-0.35187	-0.42584
LRRK1	-0.33684	-0.30652	-0.26896	-0.05451	0.033361
LRRK2	0.058727	0.037612	-0.00925	0.018108	0.010795
LRRN1	-0.39271	-0.24005	-0.32463	-0.34802	0.057146
LRRN2	0.080828	0.125422	0.097966	0.027759	0.116718
LRRN4CL BSCL2	0.270255	0.190205	0.686385	0.403	0.413978
LSG1	-0.32977	-0.15891	-0.35942	-0.33073	-0.35234
LSM1 BAG4	0.751635	-0.1058	-0.03233	-0.01924	-0.02399
LSM10	0.044625	0.198785	0.158855	-0.0644	0.144224
LSM11	0.073054	-0.03682	0.066858	-0.08481	-0.08301
LSM12	0.071894	0.164971	0.095451	-0.1573	-0.07033
LSM14A	-0.10442	-0.11409	-0.23587	-0.12015	-0.00475
LSM14B	-0.16252	-0.14459	-0.24397	-0.18565	-0.14902
LSM2	-0.27136	-0.10258	-0.33023	-0.17567	-0.30754
LSM4	-0.20426	-0.06329	-0.18732	-0.15991	-0.18868
LSM5	-0.11415	-0.54228	-0.3676	-0.34379	-0.4465
LSM6	0.022625	-0.0972	-0.2176	-0.16196	-0.18198
LSM7. AC004410.1	-0.03479	0.070809	0.194791	0.010928	-0.09339
LSM8	-0.70223	-0.72296	-1.24289	-0.66792	-1.02985
LSMD1 CYB5D1	-0.30119	-0.28723	-0.36464	0.106345	-0.19162
LSR	-0.36379	0.036987	0.209652	0.451661	0.363334
LSS MCM3APAS.	-0.22118	-0.23947	-0.27173	-0.15238	-0.38149
LST1	-0.29641	-0.31145	-0.43247	-0.66548	-0.4162
LTA	0.599011	0.601977	0.489677	0.26284	0.420586
LTA4H	-1.00468	-0.84286	-1.27601	-1.05193	-1.136
LTB	0.251232	0.299678	0.183582	-0.09347	0.083883
LTB4DH	0.183734	0.088355	0.427943	0.412579	0.157105

LTBP2	0.005314	0.179205	0.637697	0.347033	0.178063
LTBP3	-0.16857	-0.25304	-0.32777	-0.20515	0.015316
LTBP4	0.407485	0.657697	0.768176	0.470195	0.48279
LTBR	0.256371	0.187832	0.589077	0.311031	0.488902
LTF	-0.09343	0.172794	-0.09958	0.176895	-0.09381
LTK	0.204912	0.456837	0.2491	0.291768	0.268818
LTV1	-0.07256	0.372384	0.05192	-0.00832	-0.04024
LUC7L	0.227666	0.418497	0.417313	0.295249	0.241787
LUC7L2	-0.45033	-0.44227	-0.64219	-0.36167	-0.45344
LUM	-0.48675	-0.72726	-0.73059	-0.50554	-0.98874
LUZP1	0.361578	0.463995	0.771547	0.34843	0.492973
LUZP4	0.564385	0.656256	0.760878	0.353779	0.235
LY6D	0.261907	0.518868	0.542308	0.209669	0.165617
LY6E	-0.05261	0.028049	0.131179	-0.06027	0.057686
LY6G5B	0.355027	0.52351	0.548691	0.260886	0.565534
LY6H	-0.14932	-0.09087	-0.17602	0.003974	0.111809
LY6K	0.161139	0.37567	0.57174	0.397123	0.49011
LY75	0.197623	0.459606	0.699219	0.373286	0.368316
LY86	-0.19922	0.056179	-0.22894	-0.01101	0.082082
LY9	0.12408	-0.9472	-0.80472	-0.78069	-0.51639
LY96	0.785584	0.783984	0.79548	0.055672	0.031904
LYAR ZNF509	-0.31753	-0.27167	-0.45614	-0.28566	-0.30737
LYCAT	0.041236	-0.09668	-0.43247	-0.12361	-0.32452
LYG1	0.561111	0.523332	0.482827	0.252079	0.295347
LYG2	0.284005	0.261973	0.620604	0.20243	0.252534
LYL1	-0.05397	-0.1528	-0.24513	0.003282	-0.03781
LYN	0.12209	0.150886	-0.18159	0.014238	-0.07564
LYPD1	-0.21397	-0.09713	-0.15959	-0.05594	-0.14144
LYPD2	0.19119	0.152616	0.324014	0.077788	0.129757
LYPD3	0.417366	0.164043	0.722883	0.102305	0.306087
LYPD4 DMRTC2	0.60455	0.779757	0.686683	0.619372	0.764799
LYPD5	0.213955	-0.17348	0.426411	-0.10534	-0.07034
LYPD6	-0.1234	-0.2193	-0.29417	0.116428	0.05688
LYPLA1	0.231616	0.213875	0.397301	0.181743	0.317611
LYPLA2	-0.46833	-0.07406	-0.27622	-0.09269	-0.03652
LYPLA3	-0.43023	-0.28074	-0.73006	-0.41386	-0.5243
LYPLAL1	0.161388	0.007422	-0.02564	-0.22362	-0.18934
LYRM2	0.294101	-0.10727	-0.34446	0.032779	0.117087
LYRM4 FARS2	-0.83102	-0.64686	-0.67644	-0.43595	-0.60282
LYRM7	-0.44424	-0.60161	-1.01479	-0.55922	-1.13791
LYSMD1 SCNM1	-0.71885	-1.04338	-1.82383	-1.18351	-0.92293
LYSMD2	0.279019	0.451028	0.318763	0.235732	0.281828
LYSMD3	-0.21316	-0.2367	-0.05101	-0.15946	-0.21676
LYST	-0.25897	-0.36838	-0.45762	-0.27903	-0.1551
LYZ	-0.05901	0.215365	-0.00032	-0.24092	-0.18031
LYZL1	0.277463	0.276787	0.326224	0.217047	0.11399
LYZL2	0.341164	0.48663	0.43566	0.252742	0.290365
LYZL4	-0.39252	-0.40461	-0.45366	-0.55105	-0.28645
LYZL6	-0.42805	-0.57892	-1.29955	-0.82103	0.136144
LZIC NMNAT1	-0.22044	0.118807	-0.13637	0.024451	-0.02346

LZTFL1	-0.03244	-0.15682	-0.2513	0.002973	0.073444
LZTR1	-0.02711	0.144097	-0.13273	0.020852	-0.09839
LZTS1	0.489802	0.560694	0.511195	0.539285	0.738898
LZTS2	0.113719	0.068218	0.082257	0.140086	0.103703
M6PR	-0.28542	-0.56373	-0.34142	-0.32758	-0.37027
M6PRBP1	0.090802	-0.08932	-0.01258	-0.04221	-0.02919
MACF1	0.438363	0.374668	0.448314	-0.07675	0.249692
MAD1L1	0.187078	0.308058	0.207804	0.149128	0.228735
MAD2L1	-0.29262	-0.7972	-0.49251	-0.38177	-0.33287
MAD2L1BP	0.369371	0.29836	0.200665	-0.48301	0.283084
MAD2L2	0.01888	0.044264	-0.01244	0.032688	-0.00984
MAD2L2 C1orf187	0.014825	0.121754	0.119954	0.218445	0.127539
MADCAM1	-0.29416	-0.30244	0.049601	-0.0875	-0.13795
MAEA	0.016922	0.115881	-0.01542	0.015311	-0.06595
MAEL	0.431923	0.433976	0.526535	0.312939	0.477828
MAF	-0.22074	-0.04912	-0.2878	-0.07531	-0.06872
MAF1 KIAA1875	0.317411	0.484098	0.463119	0.313318	0.364091
MAFA	-0.0642	-0.03216	0.014647	0.076982	0.085715
MAFB	-0.09052	-0.14575	-0.05083	-0.05133	-0.13243
MAG	0.255754	-0.59771	-0.3201	-0.2335	0.018959
MAGEA1	0.330195	0.103446	0.011211	0.206683	0.381154
MAGEA10	0.406001	0.220771	-0.28925	0.280417	0.016283
MAGEA2	0.323342	0.366535	-0.06474	0.379703	0.439879
MAGEA3	0.351558	0.545303	-0.65379	0.356829	0.390625
MAGEA5	0.255106	-0.44074	-0.56198	0.251584	-0.0056
MAGEA8	0.075337	0.003168	-0.09824	0.167317	0.264144
MAGEA9	0.403551	0.003616	-0.0149	0.397085	0.24645
MAGEB1	0.543577	0.561946	0.438627	0.429509	0.650264
MAGEB10	0.806761	0.83785	0.917141	0.622537	0.754422
MAGEB18	0.359486	0.430868	-0.48064	0.190259	0.111214
MAGEB2	0.243439	1.679981	0.302272	0.392339	0.517793
MAGEB3	-0.0685	-0.01306	-0.83491	0.005654	-0.45259
MAGEB4	-0.05029	0.127082	-0.78515	0.043147	-0.26497
MAGEB6	0.135838	0.25383	-0.02777	0.138496	0.173915
MAGEC1	0.416433	-0.06256	-0.37697	0.184467	0.190421
MAGEC2	0.286274	-0.2155	-0.23127	0.290049	0.173847
MAGED2	-0.06968	-0.24825	-0.32899	-0.38732	-0.37264
MAGED4B	-0.07517	-0.29281	-0.15023	-0.55348	-0.38759
MAGEE1	0.18326	-0.09738	0.017938	-0.09635	-0.22709
MAGEE2	0.207344	0.18983	0.195838	0.397781	0.081032
MAGEF1	-0.41719	-0.42237	-0.67363	-0.32269	-0.16287
MAGEH1	0.058327	-0.02644	-0.16949	0.168121	-0.08926
MAGI1	-0.00483	0.0648	0.032404	-0.10201	0.038878
MAGI3	-0.09999	-0.12461	-0.12594	-0.13887	-0.13977
MAGIX.	0.358117	0.338513	0.378004	0.17501	0.305184
MAGOH	0.113185	-0.34193	-0.52217	0.010318	-0.38018
MAGOHB	-0.10274	-0.47263	-0.86205	-0.69713	-1.05636
MAGT1	0.22607	-0.21912	-0.78906	-0.15881	-0.24116
MAK	0.551402	0.549831	0.493449	0.267463	0.287866
MAK10	-0.06271	-0.04331	-0.03067	-0.17818	-0.10643

MAL	0.067867	0.264554	0.602329	0.311252	0.334477
MAL2	0.194705	0.095756	0.462669	0.346671	0.422407
MALL	0.054524	-0.19805	0.016188	0.015759	-0.08055
MALT1	0.110728	0.187488	-0.08513	-0.08605	-0.07153
MAMDC1	0.556222	0.43188	-0.19214	0.162162	0.421856
MAMDC2	0.198671	0.585421	0.730349	0.49366	0.523678
MAMDC4	0.144472	0.375816	0.461829	0.258079	0.463093
MAMLD1	-0.44057	-0.65572	-0.65234	-0.24148	-1.10346
MAN1A1	-0.16366	0.001782	-0.13159	-0.04964	-0.11658
MAN1A2	-0.22038	-0.0951	-0.11031	-0.07041	-0.09802
MAN1B1	0.019496	0.090908	-0.07517	0.118733	0.132677
MAN1C1	0.124065	0.048882	0.211633	0.127151	0.182074
MAN2A1	0.005383	0.076198	-0.00356	-0.20957	-0.09205
MAN2A2	0.32148	0.569903	0.527372	0.519384	0.760997
MAN2B1	0.062791	-0.19763	-0.34948	-0.2412	-0.31548
MAN2B2	0.006475	-0.04075	0.004798	-0.07595	-0.0511
MAN2C1	0.015019	-0.0153	-0.12211	0.07666	-0.08598
MANBA	-0.17759	0.499448	0.191692	0.22641	0.373344
MANBAL	0.255286	0.154547	0.253576	0.130275	0.313596
MANEA	-0.42863	-0.2862	-0.32502	-0.32344	-0.26856
MANEAL	0.017771	0.048234	-0.07068	-0.08944	-0.00391
MANEAL YRDC C1orf122	-0.20874	-0.08741	-0.09177	-0.12311	0.008805
MANSC1	0.09536	0.239561	-0.23188	-0.00782	-0.04758
MAOA	0.169891	-0.44114	-0.12889	-0.54517	-0.36283
MAOB	0.237141	0.232256	-0.07242	0.111746	0.300391
MAP1A	-0.82367	-0.73972	-0.7416	-0.74717	-0.77387
MAP1LC3B	-0.01467	-0.03246	0.065744	0.142761	0.212377
MAP1LC3C	0.217374	0.395246	0.689473	0.38227	0.300825
MAP1S	0.192297	0.195218	0.127249	0.002204	0.214041
MAP2	-0.39623	-0.61721	-0.5551	-0.74934	-0.8029
MAP2K1	-0.08213	0.003597	0.134486	0.031701	0.055304
MAP2K1IP1	0.2027	0.254059	0.240183	0.073156	-0.01976
MAP2K2	-0.11132	-0.02521	-0.16852	-0.1191	-0.17157
MAP2K4	0.042262	0.051709	0.074947	-0.00577	0.287956
MAP2K5	-0.23839	-0.14719	-0.36139	-0.22238	-0.2774
MAP2K6	0.282496	0.420472	0.229435	0.199975	0.390852
MAP2K7	-0.09025	-0.12585	-0.05475	-0.12573	-0.27352
MAP3K10	0.170346	0.060643	0.077384	0.143023	0.12776
MAP3K12 TARBP2	-0.62057	-0.78917	-0.83229	-0.7407	-0.7007
MAP3K13	0.336966	0.33944	-0.11979	-0.05486	0.366935
MAP3K15	0.080874	0.245328	-0.05771	0.063043	0.011375
MAP3K19	-0.10006	-0.10631	0.018417	0.155492	0.109204
MAP3K2	0.041497	0.007291	-0.14244	-0.21057	-0.1117
MAP3K4	-0.21218	-0.03074	-0.1368	-0.22516	-0.20131
MAP3K5	-0.07804	-0.03055	-0.14372	0.129188	0.061954
MAP3K6	-0.14915	-0.16829	-0.17048	0.002836	-0.0691
MAP3K7	-0.67459	-0.36584	-0.48182	-0.4603	-0.48729
MAP3K7IP1	0.189879	0.316305	-0.02583	0.003444	0.046274
MAP3K8	0.095574	0.016899	0.075736	0.109356	0.110404
MAP3K9	-0.08345	-0.1603	-0.07676	-0.19721	0.040394

MAP4K1 EIF3K	0.096493	0.24088	0.407265	0.20972	0.377995
MAP4K2 MEN1	0.029891	0.026835	-0.11802	-0.0701	0.082405
MAP4K3	-0.0698	-0.14783	-0.18399	-0.06027	-0.16029
MAP4K4	0.222377	-0.0428	0.057277	0.167526	-0.00138
MAP4K5	0.000208	0.008385	0.166806	0.147553	-0.04664
MAP6	-0.02335	0.070576	0.130489	-0.03554	0.126171
MAP6D1	0.226766	0.288172	0.347402	0.304843	0.186623
MAP7	0.20194	0.335824	0.040761	0.389778	-0.07022
MAP7D2	0.285811	0.305147	0.426331	0.135122	0.350993
MAP9	-0.00023	0.157929	0.114241	0.004514	-0.27934
MAPK1	0.038401	0.27646	0.2016	0.080724	0.055999
MAPK11	-0.00299	0.146357	0.042483	0.047877	-0.0243
MAPK12	0.044067	0.161268	0.18999	-0.00273	0.025107
MAPK13	0.183732	0.367964	0.546478	0.290343	0.41801
MAPK14	0.176828	0.175494	0.034757	0.272816	0.227445
MAPK15	0.277572	-0.06537	0.177274	0.239051	0.111696
MAPK1IP1L	0.139065	0.14898	0.221203	0.112659	0.266454
MAPK4	0.414301	0.547906	0.524047	0.335548	0.368441
MAPK6	0.295	0.205434	0.399499	0.215782	0.18962
MAPK7	-0.23602	0.063469	-0.25513	-0.19214	-0.08252
MAPK8	0.482739	0.561708	0.432255	-0.03964	0.287424
MAPK8IP1	0.147329	0.239107	0.307942	0.204422	0.223861
MAPK8IP2	0.187435	0.109945	0.175018	0.118969	0.211006
MAPK8IP3	-0.0918	-0.19277	-0.1656	-0.0325	0.040337
MAPK9	0.350769	0.494839	0.752975	0.406482	0.530463
MAPKAP1	-0.07649	-0.02792	-0.08587	0.026411	-0.09332
MAPKAPK3	-0.17094	-0.02607	-0.29155	-0.19135	0.022886
MAPKAPK5	-0.1242	-0.07055	-0.04883	-0.02588	-0.0878
MAPKBP1	-0.05677	-0.00128	-0.15531	-0.18504	-0.55828
MAPRE1	0.203718	0.091311	0.07178	-0.01098	0.075908
MAPRE2	-0.49608	-0.33582	-0.33417	-0.32398	-0.22637
MARCKS	-1.92534	-1.31738	-1.50823	-1.76411	-1.77099
MARCKSL1	0.062052	0.006826	-0.11414	0.084651	0.031605
MARCO	0.151588	-0.67809	-0.41302	-0.39268	-0.21327
MARK1	-0.19541	0.206745	0.275698	-0.066	-0.19955
MARK3	0.070784	0.067087	0.133172	0.078376	0.075555
MARK4	-0.23218	-0.29161	-0.41341	-0.16562	-0.32964
MARS2	-0.41398	-0.53891	-0.47814	-0.46461	-0.58467
MARVELD1	0.058837	0.100667	0.040933	0.082002	0.284141
MARVELD3	-0.12278	0.171581	0.305367	0.197204	0.073038
MAS1	0.601707	0.084951	-0.00134	0.049131	0.371276
MAS1L	0.119165	-0.78464	-0.57612	-0.44416	-0.5444
MASP1	0.021073	-0.66974	-0.101	0.102418	-0.13807
MASP2	0.118995	0.364051	0.396157	0.256697	0.262599
MAST1	-0.01058	-0.00573	-0.13572	-0.06467	-0.00963
MAST2	-0.44906	-0.52003	-0.72767	-0.33131	-0.42445
MAST4	0.077978	0.320766	0.37451	0.192549	0.380702
MAT1A	0.143063	0.166435	0.229869	0.233401	-0.70124
MAT2A	-0.21629	-0.35495	-0.13432	-0.3201	-0.12307
MATN1 AL137857.29	0.087871	-0.07279	0.188001	-0.18464	-0.09662

MATN2	0.125314	0.135205	0.151764	0.300898	0.236822
MATN3	0.097045	0.236995	0.259869	0.237552	0.469262
MATN4	0.223176	0.282012	0.394	0.331503	0.385912
MAX	-0.19324	-0.27026	-0.23172	-0.13454	-0.0813
MAZ	-0.27623	-0.30012	-0.12145	-0.25871	-0.3861
MBD1	-0.2643	-0.08373	-0.1736	-0.09191	0.200324
MBD2	-0.25338	-0.1902	-0.23143	-0.26621	-0.08268
MBD3	-0.23136	-0.31478	-0.30812	-0.3255	-0.20993
MBD3L1	0.124079	-1.21915	-0.51129	-0.28742	-0.41572
MBD3L2	0.638427	-0.9591	-0.83429	-0.32973	-0.04365
MBD4 IFT122	-0.31911	-0.11059	-0.28163	-0.28441	-0.33916
MBD5	0.506252	0.888674	0.765841	0.102246	0.380035
MBIP	0.216392	-0.32768	-0.89077	-0.25381	-0.36506
MBL2	0.152287	0.303141	0.440277	-0.03748	0.316971
MBNL2	0.49434	-0.8208	-0.75952	-0.56525	-0.48991
MBNL3	0.100969	0.456614	0.139764	0.049502	0.158675
MBOAT2	-0.02926	-0.04301	0.092872	-0.03386	0.068329
MBOAT5 C1S	-0.1262	-0.10171	-0.37997	-0.21607	-0.51334
MBTD1	0.178101	0.390006	0.166132	-0.09125	0.146635
MBTD1 UTP18	-0.1887	-0.19525	-0.43945	-0.39728	-0.21232
MBTPS1	-0.09045	0.118384	-0.02408	0.0914	0.094213
MC2R	0.379103	-0.92105	-0.82894	-0.32505	-0.22036
MC3R	0.099783	0.168835	0.20148	0.03153	0.191521
MC4R	0.044584	0.259207	0.054613	-0.08138	-0.00915
MC5R	0.457461	-0.26601	0.459219	0.180754	0.407143
MCAM	0.015983	0.08835	-0.05558	0.021556	-0.04709
MCART2	0.376743	0.585408	0.571662	0.217029	0.31841
MCART6	-0.14642	-0.50257	0.070029	-0.52334	-0.72008
MCAT	0.384937	0.331669	0.610707	0.30693	0.419724
MCC	-0.51887	-0.46533	-0.67943	-0.60262	-0.6572
MCCC1	-0.44069	-0.23427	-0.47451	-0.44763	-0.60323
MCCC2	0.210284	0.137896	-0.05491	0.000124	-0.04622
MCEE MPHOSPH10	-0.17816	-0.39741	-0.32179	-0.3647	-0.05117
MCF2	0.102201	-0.32569	0.03515	-0.18353	-0.03304
MCF2L	0.320787	0.115552	0.119254	0.125716	0.296931
MCFD2	0.101482	-0.16551	-0.11198	-0.1797	-0.20892
MCHR1	0.294746	0.264418	0.341482	0.127667	0.388737
MCHR2	0.235543	0.121192	0.230205	0.442686	0.340409
MCL1	-0.08936	-0.01725	-0.0785	-0.19076	-0.20178
MCM10	-0.31814	-0.35209	-0.61136	-0.30014	-0.40178
MCM2	0.19255	0.242715	0.078242	0.161725	0.362153
MCM3	-0.3069	-0.82116	-1.0225	-0.65972	-0.91256
MCM3AP C21orf57	0.047479	0.032367	0.122289	-0.03676	0.086809
MCM5	0.214951	0.160331	-0.06958	0.095998	0.165002
MCM6	0.219603	0.167176	0.282888	0.178235	0.211214
MCM7	-0.35496	-0.4823	-0.7671	-0.32511	-0.43191
MCOLN1	0.069279	-0.07639	0.07782	-0.09919	-0.04346
MCOLN1 PNPLA6	-0.26706	-0.08338	-0.07734	-0.02267	-0.2489
MCOLN2	0.059626	0.327365	0.069107	0.133611	0.209235
MCRS1	-0.14259	-0.02955	-0.11825	0.003012	-0.09127

MCTP2	0.434048	0.114088	0.945647	0.096428	0.583932
MDC1	0.056122	-0.02011	-0.19036	-0.16134	0.025129
MDFI	-0.11175	-0.13172	-0.12098	0.017251	0.042637
MDFIC	0.451079	-0.11469	0.358039	0.362957	0.512994
MDGA1	-0.19085	-0.13806	-0.11299	-0.10526	-0.26229
MDH1B FASTKD2	-0.7061	-0.75432	-1.1838	-0.86027	-1.02401
MDM1.	0.152252	-0.13264	-0.07541	-0.1377	-0.2045
MDM2	0.021148	0.101801	-0.30578	0.034944	0.038198
MDM4	0.392109	0.077968	-0.12695	0.060949	0.110538
MDN1	-0.2368	-0.20564	-0.24414	-0.05364	-0.17918
ME1	0.281959	0.28143	0.160899	0.174103	0.381284
ME2	-0.12461	-0.07617	-0.23975	-0.01718	0.069277
ME3	0.259798	0.225463	0.614304	0.089647	0.485454
MEA1 KLHDC3	-1.51198	-1.26106	-1.96442	-1.28792	-1.58358
MECP2	0.166861	0.25198	0.395843	0.179556	0.226531
MECR	-0.07316	0.040746	-0.22673	-0.3549	0.128721
MED10	0.038364	-0.22889	-0.48987	-0.46474	-0.2462
MED11	-0.08208	0.118039	-0.22051	-0.0124	-0.20407
MED12	0.097303	0.263075	-0.47376	-0.07095	-0.55993
MED13	-0.10436	-0.16362	-0.22079	-0.27876	-0.09127
MED13L	0.00862	-0.04037	-0.00715	-0.02528	0.096224
MED14	-0.1672	0.092819	-0.17387	0.042527	0.14358
MED15	0.04368	0.089252	0.075533	0.013715	-0.16196
MED16	0.155285	0.106129	0.084945	0.004109	0.046261
MED17	0.025529	0.016707	-0.387	-0.14912	-0.25998
MED18	0.280977	0.238053	0.238159	0.229217	0.108204
MED19 TXNDC14	-0.65826	-0.55655	-0.76297	-0.5728	-0.32868
MED21	-0.22046	-0.08672	-0.41516	-0.04364	-0.31548
MED22 RPL7A	-0.29271	-0.14403	-0.43432	-0.11934	-0.33587
MED25	-0.14305	-0.51068	-0.45597	-0.29741	-0.23267
MED26	-0.05992	0.007962	-0.07273	0.051671	0.060497
MED27	-0.67635	-0.75004	-0.64891	-0.79383	-0.80411
MED28	-0.1468	-0.29938	-0.49124	-0.29806	-0.41702
MED30	0.079996	-0.1557	0.020944	-0.24622	-0.21126
MED31 AC004706.4	0.066634	-0.0599	-0.00539	0.095079	0.10724
MED4	-0.03652	0.002126	-0.07595	-0.05225	-0.45342
MED6	-1.25546	-1.28308	-1.88924	-1.19285	-1.8771
MED8 KIAA0467	-0.46707	-0.72242	-0.92468	-0.58684	-0.08305
MED9	0.144954	0.049643	0.103957	0.098591	0.176331
MEF2A	0.500739	0.644731	0.832175	0.280255	0.716126
MEF2B RFXANK	-0.24431	-0.35115	-0.4666	-0.22999	-0.25097
MEF2C	-1.83246	-1.62525	-1.87973	-0.00299	-1.63448
MEF2D	-0.54555	-0.46444	-0.56683	-0.59043	-0.41819
MEFV	-0.04269	-0.42813	-0.18396	-0.20722	-0.19646
MEGF10	-0.74267	0.677675	0.605542	0.518335	0.710817
MEGF11	-0.13613	0.257215	0.32189	0.24689	0.255587
MEGF6	-0.00414	-0.114	-0.03259	-0.02692	-0.05823
MEI1	0.526852	0.482425	0.65958	0.480864	0.552953
MEIS1	-1.01622	-1.30141	-1.05332	-0.75049	-0.62034
MEIS2	-0.9584	-0.97885	-0.874	-0.23619	-0.86027

MEIS3	0.101943	-0.0151	0.125886	-0.25984	-0.297
MELK	0.178436	0.146903	0.162826	0.129489	0.094062
MEMO1	-0.1889	-0.19304	-0.26849	-0.1145	-0.11465
MEN1	-0.10589	-0.12849	0.074648	0.077877	0.050496
MEOX1	-0.33152	0.145734	0.448572	-0.24168	0.07422
MEOX2	-1.32452	-0.63327	0.22784	-0.01354	-0.10704
MEP1A	0.388006	0.022527	-0.41561	-0.42432	-0.57386
MEP1B	-0.20322	-0.08755	-0.1414	-0.45274	-0.22335
MEPE	0.030975	0.125863	0.145716	-0.24699	-0.00304
MERTK	-0.19679	-0.18926	-0.26809	-0.08041	-0.21467
MESDC1	0.094655	0.2276	0.181749	0.064495	0.158863
MESDC2	-0.33163	-0.35861	-0.39398	-0.36945	-0.43751
MESP1	0.173596	0.367333	0.373452	0.285952	0.370171
MESP2	0.375957	0.269426	0.431043	0.317546	0.357108
MET	-0.12872	-0.10523	0.133724	0.306815	0.361547
METAP1	0.116917	0.278141	0.077464	-0.16299	-0.03619
METAP2	-0.4145	-0.4519	-0.59844	-0.47624	-0.62903
METRN	0.110507	0.28528	0.398374	0.193992	0.228566
METRNL	0.002447	0.006522	0.077483	0.058304	0.068897
METT10D	-0.19346	0.033263	-0.6228	-0.25636	-0.22423
METT11D1	0.059897	0.047312	0.094212	0.136127	0.093741
METTL1 FAM119B	-0.32059	-0.36206	-0.4624	-0.37082	-0.34182
METTL10	-0.02112	-0.20927	-0.23745	0.001037	0.104779
METTL2A	-0.02118	-0.50741	-0.54065	-0.69334	-0.57963
METTL2B	-0.12504	-0.36052	-0.52367	-0.64023	-0.60496
METTL3	0.02315	-0.05242	-0.31984	-0.27044	-0.3293
METTL4 KNTC2	-1.29576	-1.15215	-1.13288	-0.9985	-0.62583
METTL5	0.144249	0.191211	0.248884	0.171796	0.172389
METTL6 EAF1	0.10907	0.082003	0.42093	0.030656	0.229701
METTL7A	-0.28485	-0.84604	-0.63872	-0.5235	-0.81951
METTL7B	-0.37329	-0.47279	-0.6628	-0.58894	-0.53995
METTL8 C2orf37	0.141507	-0.05363	0.124547	-0.03069	0.117473
MEX3C	-0.27909	-0.27661	-0.21694	-0.17434	-0.1331
MFAP1 WDR76	0.301668	0.111362	-0.1129	-0.37626	0.021804
MFAP2	0.084807	0.204673	0.15991	0.17071	0.359092
MFAP4	-0.19986	-0.61563	-1.10184	-0.67007	-0.52858
MFAP5	0.340195	-1.11472	0.27371	-0.49011	-0.17187
MFGE8	-0.08688	-0.01932	0.081592	-0.05759	-0.07521
MFHAS1	0.030137	0.090848	0.141498	0.115079	0.085004
MFI2	0.233859	0.251783	0.216003	0.18125	0.273526
MFN1	-0.38059	-0.43231	-0.46224	-0.26712	-0.46763
MFN2	0.167126	0.020432	0.010969	0.035638	-0.02739
MFNG	0.323829	0.134366	0.261337	0.208343	0.17579
MFSD1	0.084883	0.184585	0.175226	0.113453	0.211413
MFSD2	-0.03447	0.230716	0.160093	0.092814	0.185642
MFSD3	0.229435	0.195429	0.250113	0.139473	0.302755
MFSD4	-0.03539	0.081461	0.062735	-0.07288	0.155539
MFSD5	-0.39244	-0.36972	-0.44575	-0.20329	-0.30412
MFSD7	0.089201	0.310436	0.241571	0.06836	0.261982
MFSD8 C4orf29	0.032588	-0.07069	-0.0465	-0.0315	-0.10849

MFSD9	-0.30935	-0.05322	-0.20533	-0.17282	-0.19711
MGAM	0.53461	-0.5763	-0.96252	0.165645	-0.17312
MGAT1	0.174254	0.129093	0.256015	0.157097	0.180052
MGAT3	0.119392	0.09876	-0.0212	0.047588	0.129228
MGEA5	-0.3677	-0.45467	-0.5233	-0.41883	-0.38051
MGLL	0.007159	-0.09299	-0.10992	-0.04696	-0.00186
MGMT	0.364507	0.247149	0.643925	0.377574	0.531575
MGP	0.576856	-0.92451	-1.17289	-0.08459	-0.75443
MGRN1	0.060401	0.410064	0.219782	0.175166	0.131451
MGST2	-0.12797	0.376756	0.236711	0.477116	0.332275
MGST3	-0.42355	-0.34035	-0.36681	-0.33771	-0.16081
MIA	0.176642	0.450006	0.349473	0.21991	0.356698
MIA2	0.265329	0.430637	0.295914	-0.07826	0.140775
MIB1	-0.21685	-0.16444	-0.1743	-0.09209	0.025339
MIB2	0.006658	0.039798	0.07536	0.108133	0.095173
MICAL1	0.035945	0.168938	0.176556	0.074082	0.171207
MICAL2	-0.32955	-0.00268	0.078379	-0.06192	0.056865
MICALCL	0.249911	0.607216	0.314512	0.083198	0.389614
MICALL1	0.016046	0.124764	0.103208	0.096365	0.209121
MICALL2	-0.07612	-0.08561	-0.22025	-0.06016	-0.09566
MICB	-0.34043	-0.78157	-0.23969	0.247973	-0.46003
MID1IP1	0.083467	-0.10154	0.100829	-0.28118	-0.13726
MID2	0.14131	0.263147	-0.13681	-0.02966	0.01703
MIDN	0.058134	-0.04233	0.036241	0.068768	0.14951
MIER2	0.111862	0.335797	0.406985	0.329406	0.376727
MIER3	0.051123	-0.02009	0.034507	0.01147	0.082777
MIF	-0.08011	0.201002	0.063952	0.033244	0.07026
MIF4GD	0.121247	-0.07043	0.02194	-0.05026	0.049258
MINA	0.620878	0.05951	0.244122	0.032319	0.058938
MINPP1	0.094088	0.25666	-0.11019	-0.05184	0.143824
MIP	-0.13221	0.037678	0.033646	-0.11516	-0.12823
MIPEP AL445985.10	0.227001	-0.00097	-0.19763	-0.33934	-0.38511
MIPOL1	0.287085	-0.00848	0.710155	0.599212	-0.21908
MITD1 MRPL30	-0.06899	0.047444	-0.2341	-0.13634	-0.0161
MIXL1	0.265565	0.322156	0.649924	0.438757	0.416733
MIZF	-0.24987	-0.08237	-0.53792	-0.3457	-0.50456
MKI67	-0.22954	-0.06293	-0.26924	-0.17116	-0.308
MKI67IP	-0.43363	-0.29302	-0.75724	-0.5503	-0.40415
MKKS C20orf94	-0.13342	-0.0822	-0.14566	-0.0993	-0.1171
MKL1	-0.18319	-0.10765	0.003315	-0.10438	-0.18209
MKL2	0.153802	0.114775	0.22454	0.069844	0.137067
MKLN1	-0.41508	-0.33358	-0.76625	-0.45075	-0.34904
MKNK2	0.061139	-0.0278	-0.03473	-0.04557	0.073247
MKRN1	0.026994	-0.10508	-0.02565	-0.06019	-0.11357
MKRN2	-0.05389	0.242941	0.217537	0.12541	-0.2332
MKRN3	0.165337	0.063365	0.204319	0.317314	0.646579
MKX	-0.11587	0.213135	0.472884	0.351326	0.337881
MLC1	-0.09265	0.529176	0.61602	0.420411	0.417361
MLF1	-0.14478	-0.52452	-0.42309	-0.66005	-0.57162
MLF1IP	0.171944	0.056264	-0.04926	0.100377	0.026107

MLF2	-0.50529	-0.47991	-0.58402	-0.50607	-0.3799
MLH3	0.294597	0.556241	0.496009	0.353638	0.100887
MLKL	-0.08109	-0.22988	-0.19044	0.006892	-0.09075
MLL	-0.61085	-0.30919	-0.42656	-0.33857	-0.50074
MLL2	0.407101	0.373828	0.388573	0.121578	0.313784
MLL5	-0.9001	-0.8165	-1.21279	-0.76668	-0.91096
MLLT1	-0.02959	0.105014	0.207296	0.070675	0.153933
MLLT10	-0.38533	-0.37723	-0.41975	-0.38063	-0.37
MLLT3	-0.87268	-0.70201	-1.00366	-0.82215	-0.81687
MLLT6	-0.2802	-0.51647	-0.39166	-0.39341	-0.57976
MLN	0.569658	0.766397	0.878525	0.516698	0.729342
MLNR	0.225832	0.308138	0.463482	0.204981	0.279065
MLPH	-0.05255	0.249051	0.324318	0.091582	-0.04988
MLSTD1	-0.41656	-0.42473	-0.73943	-0.63086	-0.59806
MLSTD2	-0.05433	-0.13979	-0.14133	-0.05556	0.176391
MLX	0.305038	0.211542	0.184411	0.1635	0.014853
MLXIPL	0.111728	0.115524	0.251691	0.202716	0.113974
MLYCD	0.068295	0.027848	-0.00568	0.059473	0.047383
MLZE	-0.01896	-0.22423	-0.27543	-0.45457	-0.58309
MMAA	0.629728	0.892737	0.94185	0.378094	0.67272
MMAB MVK	-0.14683	-0.07788	-0.16204	-0.1886	-0.44045
MMD	0.086275	-0.15292	0.032288	-0.12571	0.011023
MMD2	-0.54017	-0.25214	-0.34263	-0.24652	-0.2666
MMEL1	0.032323	0.118761	0.164532	-0.01153	0.144121
MMP1	0.301714	-0.47117	-0.42032	0.11957	-0.34886
MMP10	-0.56883	-0.41329	-0.6561	-0.16974	-0.63832
MMP11	0.124537	0.296864	0.210882	0.241495	0.350016
MMP12	0.39126	-0.44654	-0.35801	0.04345	-0.63953
MMP13	0.777242	0.888258	1.228411	0.53384	0.461072
MMP14	0.209379	-0.43956	0.844706	0.468059	-0.03008
MMP15	0.027871	0.053638	0.202907	0.157747	0.035044
MMP16	-0.86533	-0.91111	-0.35448	-0.71196	-0.54713
MMP17	0.180593	0.217379	0.111191	0.267542	0.265041
MMP19	0.152631	0.372877	0.279349	-0.02773	0.103453
MMP2	-0.10634	-0.02374	-0.18568	0.020873	-0.05734
MMP20	0.366454	0.14859	-1.01227	-0.43717	-0.83507
MMP21	0.60172	0.781093	0.83099	0.579349	0.58804
MMP23B	0.162028	0.207364	0.403547	0.253315	0.427185
MMP24	-0.23756	0.193402	-0.17635	-0.30399	-0.16493
MMP25	-0.10147	-0.04533	-0.06823	0.253465	0.124024
MMP27	-0.19455	-0.54698	-1.05571	-0.45689	-0.87153
MMP28	0.147226	0.365314	0.360158	0.189408	0.31958
MMP3	0.173796	-0.14903	-0.64087	0.298026	-0.73109
MMP7	-0.02825	0.280248	-0.30715	0.070356	-0.29017
MMP8	-0.77192	-0.49331	-0.7503	-0.42309	-0.74695
MMP9	-0.39372	-0.53558	-0.52865	-0.5161	-0.51339
MMRN1	-0.06224	0.281433	-0.01378	-0.17121	-0.46893
MMRN2 SNCG	0.17753	0.0896	0.226004	0.020896	0.108484
MMS19 UBTD1	-0.17939	-0.19382	-0.1807	-0.00523	-0.13589
MN1	-0.18316	-0.0202	0.0816	0.013132	0.001198

MNAT1	-1.34907	-0.97398	-1.11896	-0.93545	-1.13244
MND1	0.161166	0.119555	0.150372	0.057152	0.302613
MNDA	-0.49506	-0.72141	-0.45839	-0.55287	-0.40543
MNS1	0.370454	-0.15327	-0.38513	0.153279	0.015532
MNT	-0.33649	-0.03622	-0.27217	-0.26379	-0.04324
MNX1 AC006357.5	0.073556	0.296784	0.329447	0.163151	0.217077
MOAP1 C14orf109	0.063026	0.089969	0.003506	0.188898	0.197654
MOBKL1A	0.138618	0.04589	-0.02522	0.061724	0.010949
MOBKL1B	-0.17962	0.068342	-0.22741	-0.13345	-0.0262
MOBKL2A C19orf36	0.23625	0.061326	-0.04692	0.222816	0.266961
MOBKL2C	0.087179	-0.31719	-0.14945	0.047561	0.071595
MOCOS	-0.04145	0.416773	0.415586	0.490051	0.46869
MOCS1	0.486602	0.823779	0.606845	0.632007	0.986267
MOCS2	0.084063	-0.09954	-0.28535	-0.15916	-0.10829
MOG	-0.08677	-0.69601	-0.97769	-0.50868	-0.5749
MOGAT1	0.365377	0.45383	0.501058	0.389141	0.369467
MOGAT2	0.094761	0.228584	0.485286	0.129706	0.286831
MOGAT3	-0.01237	-0.59726	0.288237	-0.04609	0.12518
MON1A	0.231453	0.427308	0.376824	0.235341	0.260384
MON1B	-0.53005	-0.4465	-0.2821	-0.09555	-0.29796
MON2	-0.37241	-0.6053	-0.47231	-0.16584	-0.56628
MORC1	0.422372	0.508951	0.648823	0.35465	0.349625
MORC2	0.291295	0.764387	0.48623	0.473991	0.544565
MORC3	0.128054	0.086345	0.189398	0.151267	-0.10814
MORC4	0.138985	0.249502	-0.17319	0.207714	0.204689
MORF4L1	-0.03368	0.281295	0.167592	0.128391	0.160993
MORF4L2	-0.06132	-1.16351	-0.0614	-1.01645	-1.37428
MORN3	0.081508	0.264604	0.205785	0.122902	0.256681
MOS	0.142545	-0.32911	-0.09335	-0.03566	-0.10045
MOSC1	0.367604	0.421301	-0.02527	0.062693	0.292827
MOSC2	0.058277	0.627685	-0.60476	0.487085	0.629806
MOSPD1	0.197731	0.290705	0.316469	0.133184	0.079788
MOSPD3	-0.16428	-0.2981	-0.67411	-0.17341	-0.46595
MOV10	-0.50659	-0.53235	-0.51778	0.015551	-0.45007
MOV10L1	0.417259	0.377072	0.721335	0.34836	0.526118
MOXD1	0.230074	0.077185	0.264407	0.252297	0.350213
MPDU1	0.291562	0.310521	0.48635	0.141288	0.288129
MPDZ	0.135613	0.174263	0.03503	-0.07186	0.194154
MPG	0.110638	-0.01466	0.158053	0.091763	0.285337
MPHOSPH1	-0.71256	-0.78078	-0.87308	-0.50923	-0.759
MPHOSPH6	0.055073	0.094791	0.21037	0.144528	-0.11798
MPHOSPH8	0.070053	0.125122	0.00793	0.009133	-0.01258
MPHOSPH9	0.22763	0.479674	0.602658	0.062527	0.3183
MPI	0.059401	0.109493	-0.07003	-0.04552	0.053204
MPL	0.272015	0.08712	0.059284	-0.04951	-0.02133
MPN2	0.255325	0.318029	0.384543	0.248292	0.317927
MPND	-0.21283	-0.23246	-0.37708	-0.19952	-0.21359
MPO	-0.13801	-0.745	-0.7383	-0.61459	-0.17949
MPP1	0.35787	0.138417	-0.02216	0.149669	0.251433
MPP2	-0.17561	-0.48694	-0.39841	-0.60819	-0.11001

MPP3	0.30086	0.616271	0.370151	0.258983	0.306893
MPP4	-1.21859	-0.8827	-1.32338	-1.03069	-1.01236
MPP5	-0.00815	0.090013	-0.1538	-0.10914	-0.10182
MPP6	-0.26834	-0.28545	-0.34401	-0.14871	-0.1948
MPP7	0.545645	-0.00703	-0.29748	-0.35128	0.082075
MPPE1	0.222535	0.34235	0.292617	0.118074	0.228291
MPPED2	0.533939	0.008076	0.626689	0.365961	0.10624
MPV17	-0.92245	-0.75211	-1.12216	-0.76484	-1.1326
MPV17L	0.205752	0.459513	0.431552	0.338148	0.403503
MPZ	-0.23606	-0.41515	-0.38184	-0.36796	-0.30421
MPZL1	0.039766	-0.22339	-0.15273	0.104418	-0.21921
MPZL2	-0.21282	-0.56894	-0.42575	-0.05844	-0.74118
MPZL3	-0.35893	0.098405	-0.10283	0.118523	-0.51169
MR1	0.212152	-0.70084	-0.12273	-0.00136	0.078855
MRAP	0.035031	-0.08863	-0.16978	-0.15406	-0.18557
MRAS	0.310901	0.529766	0.425505	-0.00548	0.340986
MRC1	0.502256	0.672871	0.728071	0.41355	0.732146
MRC1L1	0.473572	0.751957	0.741298	0.475708	0.627351
MRC2	-0.28398	-0.0904	-0.22254	-0.15627	-0.30023
MRE11A ANKRD49	-0.79734	-0.39603	-0.80312	-0.54457	-0.71544
MREG	-0.00141	0.210928	0.070623	0.044776	0.238024
MRFAP1	-0.40274	-0.46451	-0.56265	-0.26961	-0.3933
MRFAP1L1	-0.74018	-0.23036	-0.88245	-0.06444	-0.8505
MRGPRD	0.278797	0.399284	0.374002	0.295333	0.453404
MRGPRE	0.271029	0.391903	0.381197	0.37431	0.219694
MRGPRF	0.16255	0.334067	0.101978	0.105772	0.255408
MRGPRG	0.213562	0.23577	0.22893	0.286636	0.389387
MRGPRX1	0.025257	-0.19841	-0.07034	-0.1748	-0.28492
MRGPRX2	0.027733	-0.63629	0.119337	-0.2062	-0.59343
MRGPRX4	-0.14449	-0.72678	-0.18577	-0.41485	-0.49326
MRM1	-0.1839	-0.18233	-0.31312	-0.38099	-0.42334
MRO	-0.41335	-0.65998	-0.69403	-0.79817	-0.60427
MRPL1	-0.40168	-0.52981	-1.18528	-0.88268	-1.02852
MRPL10 LRRC46	-0.53202	-0.64454	-0.98709	-0.7773	-0.87546
MRPL11	0.051997	-0.01733	0.608969	-0.18796	0.036711
MRPL12	0.00547	-0.22804	-0.10596	-0.00056	-0.02196
MRPL13 MTBP	-0.86254	-1.14039	-0.95046	-1.0778	-1.06644
MRPL14 TMEM63B	-0.20534	-0.24963	-0.25286	-0.17084	-0.23777
MRPL15	0.240289	0.492737	0.375277	0.211195	0.328483
MRPL16	-0.28001	0.076924	-0.41471	-0.00501	-0.24542
MRPL17	0.027522	-0.18366	-0.11926	-0.08353	-0.02677
MRPL19	0.010804	0.20979	0.157381	0.042243	0.228648
MRPL2 KLC4	-0.60913	-0.56968	-0.79683	-0.71098	-0.62722
MRPL20	0.066171	0.073862	-0.09147	0.120836	-0.06173
MRPL21 IGHMBP2	-0.61975	-0.73821	-0.84114	-0.72512	-0.89144
MRPL22	0.187361	0.074305	0.149748	-0.05384	0.23379
MRPL23	-0.05203	0.00784	0.067696	0.012748	0.024098
MRPL24	-0.32183	-0.36261	-0.60645	-0.60244	-0.40323
MRPL27 EME1	0.042448	-0.83677	-1.11804	-0.63456	-0.70271
MRPL28	0.311318	0.195883	0.161491	0.035102	0.066644

MRPL3	-0.18627	-0.1564	-0.16374	-0.4738	-0.34366
MRPL33	0.315544	0.099076	0.174944	-0.03766	-0.03145
MRPL35	0.382629	0.347638	0.445689	0.315767	0.41592
MRPL36 NDUFS6	-0.24632	0.468054	0.504106	0.358236	0.457747
MRPL38	0.336915	0.405808	0.558017	0.221235	0.247916
MRPL39	-0.10924	-0.53318	-0.69129	-0.50563	-0.62467
MRPL4	0.07882	0.027622	-0.00856	0.094172	-0.06961
MRPL42	-0.14958	0.103933	-0.0514	-0.27773	-0.12073
MRPL43 PEO1	-0.01122	-0.70653	-0.54345	0.016749	-0.32509
MRPL44	-0.13209	-0.14789	-0.13564	-0.17117	-0.18048
MRPL45	-0.09425	0.126346	0.072372	-0.14461	-0.24252
MRPL46 MRPS11	-0.70363	-0.44605	-0.70779	-0.48036	-0.76453
MRPL47 NDUFB5	-0.43889	-0.5304	-0.75654	-0.46584	-0.69037
MRPL48	0.59845	0.76395	0.526873	0.443999	0.238465
MRPL50 ZNF189	-1.06137	-1.11241	-1.21563	-1.00678	-0.6603
MRPL51 NCAPD2	-0.61543	-0.72001	-0.89455	-0.62312	-0.76583
MRPL52	0.114188	0.435198	0.10575	0.156588	0.051929
MRPL53	0.041311	0.085318	-0.11877	-0.05928	0.109244
MRPL55	0.052779	-0.24552	-0.53026	-0.17031	-0.26478
MRPL9 OAZ3	0.165267	0.274434	0.096779	0.10583	0.000686
MRPS10	0.022935	-0.18112	-0.04897	-0.21476	-0.31507
MRPS14	-0.33414	-0.32414	-0.82604	-0.66494	-0.58131
MRPS15	-0.42483	0.146696	-0.31803	-0.42584	-0.06086
MRPS16	-0.40365	-0.2424	-0.51916	-0.53487	-0.65207
MRPS17	-0.07503	-0.027	-0.15498	0.004096	-0.09421
MRPS18A	-0.23829	-0.3911	-0.47299	-0.45143	-0.75115
MRPS18B C6orf134	0.266726	-0.03105	-0.28314	-0.1595	-0.22352
MRPS22	-0.08437	-0.13903	-0.32249	-0.10646	-0.22726
MRPS23	-0.29188	-0.48688	-0.60189	-0.58642	-0.40587
MRPS24	0.017323	0.504508	0.30541	0.278152	0.35628
MRPS25	0.055113	0.353394	0.156038	0.078551	0.219852
MRPS27 PTCD2	-0.45262	-0.551	-0.78594	-0.40464	0.385172
MRPS28	-0.41967	-0.40304	-0.7662	-0.6708	-0.66061
MRPS30	-0.51904	-0.36011	-0.41466	-0.40009	-0.56059
MRPS31	-0.11083	-0.14389	0.025669	-0.07091	-0.34662
MRPS33	-0.42126	-0.57702	-1.0269	-0.53614	-0.64676
MRPS35	0.049834	0.157962	-0.2745	-0.14811	-0.01618
MRPS5	0.066915	-0.00585	-0.22683	-0.13161	0.059516
MRPS9	-0.27301	-0.11144	-0.79799	-0.18388	-0.14167
MRS2L	0.231098	-0.03762	-0.09182	-0.18769	-0.31438
MRVI1	0.339038	0.474967	0.472932	0.147319	0.426069
MS4A1	-1.05358	-1.74459	-1.75711	-1.34993	-0.92032
MS4A10	0.526513	0.466232	0.543692	0.157642	0.388417
MS4A12	0.150529	-0.55552	-0.55809	0.029191	0.25241
MS4A13	0.353874	-0.11348	0.250099	0.28393	0.368185
MS4A15	0.521647	0.388729	0.527689	0.378761	0.304816
MS4A2	-1.12158	-1.42046	-1.07428	-0.9991	-0.816
MS4A3	0.388947	-1.16052	-0.30675	-0.60188	-0.34739
MS4A4A	-0.14054	-1.21636	-1.09229	-0.78741	-0.44102
MS4A5	0.355195	-0.57287	-0.30331	0.114619	0.295486

MS4A6A	-0.49062	-1.26229	-1.16726	-1.09213	-0.75093
MS4A6E	-0.67574	-1.19406	-1.02767	-0.96845	-0.72015
MS4A7	-0.57715	-1.08487	-0.81443	-0.97356	-0.49751
MS4A8B	-0.79998	-0.92401	-1.51051	-1.2383	-1.0331
MSC	-0.3744	0.216848	0.278723	0.130561	0.241526
MSH2	0.142411	0.207538	0.049029	0.062086	-0.06025
MSH3	-0.07821	-0.36303	-0.35195	-0.30102	-0.41304
MSH4	0.560239	0.696614	0.667486	0.416233	-0.15663
MSH5 C6orf26	0.249646	0.246264	0.393683	0.096089	0.244336
MSH6	0.100193	0.125239	-0.0282	0.026943	0.259897
MSI1	-0.07566	-0.05183	0.107441	0.05855	0.152399
MSI2	-0.42483	-0.62275	-0.71933	-0.67754	-0.25277
MSL2L1	-0.39103	-0.15137	-0.47125	-0.19071	-0.38593
MSL3L1	-0.00673	-0.16478	0.081809	-0.026	-0.01528
MSMB	0.418724	0.532203	0.474707	0.084108	-0.35648
MSN	0.005534	-0.05035	0.251182	0.173384	0.186379
MSR1	0.454489	0.304857	0.263708	0.009946	0.160273
MSRA	-0.03317	-0.02655	-0.08319	-0.25347	-0.09749
MSRB2	0.047179	-0.32408	-0.5448	-0.46406	-0.51329
MSRB3	0.18665	0.322549	0.4634	0.246502	0.085637
MST1R	0.33114	0.552728	0.640994	0.453752	0.417101
MSTO1	0.056426	0.081388	0.04786	0.050535	0.157785
MSTP9. RNF123	0.215391	0.046079	-0.11354	-0.06689	0.065485
MSX1	0.017323	-0.03145	-0.01886	0.045485	-0.0405
MSX2	-0.06646	-0.19777	0.159184	0.186112	0.166114
MT1A	0.145346	0.43542	0.517259	0.480388	0.465218
MT1B	0.310241	0.070037	0.46049	-0.51821	0.054431
MT1E	0.081596	0.504271	0.514947	0.28563	0.523967
MT1F	-0.09372	0.24834	0.318485	0.251588	0.209062
MT1G MT1H	0.090649	-0.04178	0.473383	0.290189	0.172973
MT1M	-0.06934	0.102557	0.433574	0.263173	0.230576
MT1X	0.081022	0.079177	0.154682	0.15687	0.259974
MT2A	-0.22623	0.157342	-0.06305	0.113971	0.053044
MT3	0.294051	0.469732	0.751982	0.421447	0.442237
MT4	0.137542	0.188581	0.429307	-0.20761	0.052802
MTA1	0.019451	0.051005	0.00843	-0.10783	0.044176
MTA2 EML3	-0.06605	-0.24395	-0.02001	-0.10828	0.228334
MTA3	-0.17738	0.039811	-0.17075	-0.04619	0.077927
MTCH1 AL356983.33	-0.04759	0.065911	0.09502	0.041271	0.153122
MTCH2	-0.07822	0.023511	0.111258	-0.01188	0.09359
MTCP1 BX470111.4	0.105317	-0.33984	-0.00996	-0.22952	-0.27796
MTDH	0.065997	0.021845	-0.33771	-0.02714	0.025721
MTERF	0.309181	-0.23702	-0.12276	0.010058	-0.38216
MTERFD1 PTDSS1	-0.41134	-0.37941	-0.48469	-0.56413	-0.42182
MTERFD2	-0.25223	-0.1041	-0.29551	-0.31979	-0.40211
MTERFD3	-0.19056	-0.06738	-0.29668	-0.32981	-0.27719
MTF1	-0.72102	-0.51641	-0.77679	-0.44863	-0.35268
MTF2	-0.7663	-0.64804	-0.5661	-0.85207	-0.45515
MTFMT	0.239191	0.406453	0.501415	0.195655	0.364343
MTFR1	-0.2834	-0.19332	-0.41867	-0.24911	-0.1915

MTG1	0.133152	0.09761	0.255534	0.066714	0.118596
MTHFD1	-0.56219	-0.57678	-1.03215	-0.54455	-0.53205
MTHFD1L	0.079191	0.162266	0.108281	-0.08218	0.132685
MTHFD2	0.205844	0.232448	0.318252	0.164647	0.310145
MTHFD2L	-0.24032	-0.41497	-0.43013	-0.39949	-0.30474
MTHFR KIAA2013	-0.25493	-0.22042	-0.36334	-0.1545	-0.08685
MTHFS	0.142588	0.10001	0.260153	-0.05307	-0.01387
MTHFSD	0.359145	0.259517	0.333882	0.437529	0.262518
MTIF2	0.109655	0.330028	0.189949	0.093973	0.230737
MTIF3	-0.02109	-0.02829	-0.04761	-0.06018	-0.12789
MTL5	-0.02376	0.15424	0.038572	0.247306	0.301017
MTM1	0.235272	0.191218	0.440204	0.186992	0.296612
MTMR1	0.08071	-0.47856	-0.17788	-0.44526	-0.2554
MTMR10	-0.11497	-0.04455	0.105053	0.00884	0.074164
MTMR11	-0.2524	-0.36805	0.267861	0.370872	0.445816
MTMR12	-0.02024	-0.0758	-0.01251	-0.00447	-0.05761
MTMR14	0.00577	0.210105	0.240559	-0.03635	0.020716
MTMR15	0.209107	1.170401	0.249849	0.036963	0.204023
MTMR2	-0.59936	-0.27896	-0.50002	-0.41332	-0.57937
MTMR4	-0.60608	-0.93543	-1.02327	-0.97029	-0.85371
MTMR6	0.177319	0.239047	0.039458	-0.01859	0.032237
MTMR7	-0.01206	0.27801	-0.32547	-0.08225	-0.01465
MTMR8	-0.24632	-0.66784	-0.05591	-0.23469	-0.6028
MTMR9	0.057773	0.218867	0.356587	0.341137	0.131749
MTNR1A	0.356717	0.430492	0.441607	0.294975	0.45747
MTNR1B	0.338206	0.714949	0.624231	0.540431	0.662865
MTO1	0.104528	0.156903	-0.12554	-0.07655	-0.01649
MTP18	0.104284	0.087014	0.100869	0.141635	0.072616
MTPN	0.016837	-0.38729	-0.74977	-0.47588	-0.299
MTR	-0.11055	0.071505	-0.08356	0.068458	-0.14115
MTRF1	-0.561	-0.55316	-0.80748	-0.68911	-0.79309
MTRF1L	-0.67439	-0.49996	-0.66071	-0.61376	-0.71977
MTSS1	0.028741	0.313982	0.315464	0.211639	0.192661
MTTP	-1.08251	-0.53723	-0.90718	-0.76325	-1.09657
MTX2	0.151295	0.038564	-0.06438	-0.08241	0.217493
MTX3	-0.22584	-0.31311	-0.64183	-0.3574	-0.28789
MUC1	0.154737	0.177134	-0.00056	0.038195	-0.07673
MUC13	0.081571	-0.04192	0.518268	0.234597	0.374437
MUC17	0.154918	0.145406	0.175872	-0.00164	0.137952
MUC2	0.091082	0.229139	0.568242	0.224332	0.179151
MUC4	0.304346	-0.44798	0.424224	0.32769	-0.09479
MUC7	-0.03092	-0.45768	-0.35157	-0.43554	-0.15825
MUCDHL SCT AP006284.2	0.234484	0.3592	0.228831	0.3288	0.386884
MUCL1	-0.56138	-1.12428	-0.64367	-0.70946	-0.75818
MUM1	-0.0079	0.003627	0.092555	0.137766	0.255009
MUM1L1	0.383257	-0.31154	-0.123	-0.28477	-0.10749
MUSK	0.258706	0.5053	0.529893	0.338385	0.393313
MUT CENPQ	-1.05771	-0.76049	-1.27384	-0.98472	-1.5049
MUTYH TOE1	-0.07815	-0.26029	-0.46157	-0.29102	-0.12812
MVD	-0.15023	0.044048	-0.21562	0.00883	-0.10036

MX1	0.220827	0.167467	0.367072	0.233368	0.384289
MX2	0.421463	-0.63292	0.372328	0.456497	0.297339
MXD1	-0.11256	-0.13785	-0.17247	-0.05113	-0.00276
MXD3	-0.00209	-0.11784	0.08165	0.042718	-0.03593
MXD4 AL158068.18	0.011092	0.084917	0.049513	0.020466	0.018942
MXRA5	0.642309	0.798826	0.344837	0.488952	0.397514
MXRA7	0.012772	0.008701	-0.35944	-0.03179	-0.02915
MXRA8	0.138745	0.064928	-0.22095	0.20822	0.212797
MYADM	0.02045	-0.05654	-0.31161	0.050446	0.099065
MYADML.	0.487745	0.849935	0.886322	0.513909	0.587248
MYB	-0.23856	-0.20727	-0.29846	-0.15307	-0.22379
MYBBP1A	-0.01105	0.257343	0.208053	0.004988	0.190695
MYBL2	-0.00423	0.175397	0.044624	0.224612	0.199219
MYBPC1	0.209774	0.155837	-0.40063	-0.05779	-0.39463
MYBPC2	0.092377	0.046018	0.134383	0.017228	-0.00318
MYBPC3	0.426368	0.489172	0.527407	0.31722	0.435403
MYBPH	0.214478	0.341967	0.21164	0.255767	0.352855
MYC	-1.22031	-1.46044	-1.13384	-1.05664	-1.15264
MYCBP GJA9	-0.14116	-0.27741	-0.26466	-0.33522	-0.32622
MYCBP2	-0.14737	-0.06561	-0.06765	-0.11353	-0.08309
MYCBPAP	0.147986	0.252049	0.434579	0.294592	0.414134
MYCL1	0.078002	0.124756	0.050605	-0.05353	0.126339
MYCN	-0.03014	-0.02514	-0.02961	-2.10328	-4.23119
MYCT1	0.263433	0.391781	0.127604	-0.09605	-0.15404
MYEF2	0.193556	0.398274	0.426922	0.202247	0.23362
MYEOV	0.461273	0.616866	0.498373	0.377922	0.524163
MYEOV2	0.283111	0.292686	0.430208	0.362498	0.450062
MYF5	0.496844	0.654224	-0.0383	0.458962	0.445635
MYF6	0.237523	0.557746	-0.05041	0.048307	0.064837
MYH1	0.091791	-0.43612	-0.73015	-0.71816	-0.41973
MYH10	-0.36949	-0.15599	-0.37595	-0.30674	-0.14987
MYH11	-0.24281	0.261822	0.039765	0.317241	0.066356
MYH13	0.562405	-0.73303	-0.68049	-0.65161	-0.46225
MYH14	0.096726	0.097633	0.380147	0.134727	0.311425
MYH2	0.494846	-0.50622	-0.50662	-0.36723	0.311998
MYH4	-0.47444	-0.42247	-0.57976	-0.65125	-0.36747
MYH7B	0.454312	0.437643	0.546975	0.237023	0.35741
MYH8	0.375398	-0.83396	-1.14997	-1.33614	-0.70088
MYH9	-0.0068	0.14514	0.058752	0.125279	0.1123
MYL2	0.603133	0.636614	0.574621	0.700917	0.672632
MYL3	0.298524	0.302812	0.552073	0.155599	0.203169
MYL4	0.00058	-0.00421	-0.0996	-0.20642	-0.25507
MYL5	0.25183	0.164821	0.390224	0.198325	0.358206
MYL6B MYL6	-0.98135	-0.82231	-1.33642	-1.21139	-1.12681
MYL7	0.134014	0.050197	-0.13655	-0.13319	-0.21094
MYL9	0.177142	0.436276	0.565555	0.349897	0.387637
MYLIP	-0.0496	-0.07559	0.051016	0.090192	0.136511
MYLK2	0.171635	-0.00019	-0.01756	0.027458	0.113461
MYLK3	0.161545	0.354662	0.344554	0.379295	0.363621
MYLK4	0.348875	0.418348	0.303295	0.289168	0.150101

MYNN	-0.55933	-0.60483	-0.53623	-0.45461	-0.59391
MYO15A	0.271382	0.07048	0.411739	0.2745	0.116605
MYO16	-0.10907	-0.03999	0.908109	-0.12576	-0.064
MYO18B	0.59346	0.77683	0.670617	0.595251	0.771299
MYO19	-0.17094	-0.16807	-0.16818	-0.16675	-0.22467
MYO1A	0.141516	-0.01857	0.065951	-0.03214	0.030889
MYO1B	-0.20447	-0.07596	-0.13141	-0.04371	-0.06502
MYO1C	0.200431	0.340293	0.449044	0.140811	0.190213
MYO1D	-0.11176	-0.1894	-0.02187	0.034219	0.11948
MYO1F	0.325943	-0.50692	-0.11281	-0.40911	-0.43283
MYO1G	0.493008	0.504879	0.503942	0.314989	0.376616
MYO3A	0.013362	-0.25301	0.245967	0.186587	-0.1471
MYO3B	-0.36524	-0.29502	-0.47282	-0.32093	0.043544
MYO5A	-0.22777	-0.06852	-0.01276	-0.0161	-0.10532
MYO5C	0.125191	0.245227	0.191123	0.165183	0.068044
MYO6	-0.13655	-0.01577	-0.115	-0.03081	-0.04497
MYO9A SENP8	-0.35419	-0.22568	-0.26044	-0.13377	-0.11712
MYO9B	0.377302	0.522594	0.503624	0.287056	0.518112
MYOC	0.287822	0.324901	-0.0397	-0.19979	-0.18317
MYOCD	0.113396	0.173847	0.378494	0.289361	0.421049
MYOD1	0.020056	-0.03496	0.581843	0.476197	0.479373
MYOG	0.460138	0.635979	0.566094	0.241699	0.600006
MYOM1	0.461542	0.656276	0.802897	0.197249	0.018004
MYOM2	0.218839	0.630192	0.681379	0.566036	0.772708
MYOM3	0.290289	0.452005	0.569111	0.058365	0.406784
MYOT	-0.15644	-0.48017	-0.24419	-0.64772	-0.41402
MYOZ1	0.284431	0.351401	0.62699	-0.0435	0.042925
MYOZ2	-0.6559	-0.84327	-0.83916	-0.69571	-0.72215
MYOZ3	-0.25736	-0.22022	-0.54306	-0.52456	-0.44851
MYPN	0.520885	0.520543	0.639251	0.292249	0.519369
MYRIP	-0.04547	0.345105	0.20128	0.084088	0.198723
MYST1	-0.27241	-0.18206	-0.57928	-0.18884	-0.35506
MYST2	-0.29506	-0.19205	-0.37968	0.064874	0.125109
MYST3	-0.19658	-0.02526	-0.29238	-0.15982	-0.17376
MYT1	0.553363	-0.6259	-1.56209	-0.99413	-1.35671
MYT1L	0.473883	-0.83015	-1.2999	-1.11991	-1.29481
MZF1 AC016629.7	0.021745	-0.48847	-0.52793	-0.16851	-0.3195
N4BP1	-0.03699	-0.19008	-0.14451	0.159444	-0.12267
N4BP2L1	0.501617	0.782111	0.772947	0.484415	0.431804
N6AMT2	0.248796	0.456904	0.44963	0.118893	0.03319
NAAA	0.067818	0.28864	0.358515	0.255142	0.18253
NAALAD2	-0.64065	-0.18034	-0.12813	0.13538	-0.67225
NAALADL1	0.453195	0.353894	0.260078	0.219923	0.167241
NAB1	-0.15316	-0.14919	0.018713	0.031024	-0.08362
NAB2	-0.11802	-0.15293	-0.05984	-0.13852	0.021845
NACA2	0.674327	0.960702	0.850197	0.563051	0.774325
NACAP1.	0.108683	0.091729	-0.01635	-0.20682	-0.20805
NADK	-0.00085	-0.17623	-0.01407	-0.07555	0.102122
NADSYN1	0.273933	0.403309	0.372853	0.337423	-0.16514
NAF1	0.101881	-0.01413	0.183214	0.127232	0.085255

NAG	-0.73892	-0.46802	0.358494	-0.3145	-0.58554
NAGA	0.052953	0.134502	0.093947	-0.11856	-0.09282
NAGK	-0.29808	-0.01474	-0.35524	-0.20196	0.07036
NAGLU	0.544322	0.480923	0.559781	0.59353	0.434355
NAGPA	0.217696	0.038189	0.202478	-0.0721	0.021366
NAIP	0.591887	0.491052	0.609498	0.153414	0.374433
NALP5	0.540496	-0.98002	-0.61062	0.053185	0.174826
NALP6	0.308162	-0.39086	0.322041	0.15993	0.193933
NANOGP8.	0.20081	-1.0578	-0.93861	-0.79475	-0.15491
NANOS1	0.018325	0.01669	-0.22836	-0.08034	-0.2422
NANOS2	0.168685	0.199874	0.277978	0.140721	0.289549
NANP	0.141199	0.085255	0.039514	-0.00372	0.165708
NANS	0.136706	0.202353	0.204843	0.153137	0.312522
NAP1L1	-0.1631	-0.24178	-0.30101	-0.24715	-0.17304
NAP1L2	0.04957	-0.15633	0.025786	0.133047	-0.79748
NAP1L3 FAM133A	-0.47172	-0.64493	-0.10972	-0.72672	-0.83692
NAP1L4	0.083201	0.129922	0.085001	0.001819	0.24311
NAPA	-0.04135	-0.14764	-0.20467	-0.10947	-0.217
NAPB	-0.3341	-0.26628	-0.39688	-0.26268	-0.39804
NAPEPLD	-0.00349	-0.11517	-0.00285	0.053354	0.065615
NAPG	0.318863	0.462827	0.693432	0.320702	0.261169
NAPRT1	0.200955	0.050668	0.545108	0.232349	0.166058
NAPSA	0.433233	0.516632	0.536263	0.077671	0.226722
NARF	0.16232	0.140862	0.140049	-0.01887	0.064808
NARG1L	-0.02569	0.203045	0.060666	0.054043	0.033903
NARG2	-0.13889	0.30939	0.069835	0.125544	0.132013
NARS2	-0.49214	-0.38157	-0.93773	-0.54214	-0.94434
NASP	-0.45711	-0.24844	-0.50264	-0.21092	-0.24355
NAT1	0.077847	-0.04496	0.090961	-0.09068	-0.2474
NAT10	-1.05395	-0.77573	-1.25158	-0.64319	-0.62123
NAT11	-0.05621	0.086084	-0.08614	0.004955	0.021823
NAT12	-0.22037	-0.09185	-0.19069	-0.15247	-0.22903
NAT13 ATP6V1A	-0.78568	-0.68452	-0.91005	-0.6964	-0.80655
NAT14	0.023152	0.144161	0.108099	0.160739	0.070809
NAT2	0.005894	0.088003	0.066674	-0.19625	-0.16359
NAT5	0.08161	0.123184	0.149298	0.016774	0.120841
NAT8 ALMS1L.	0.354693	0.33685	0.359767	0.296704	-0.18604
NAT8L	0.117207	0.225891	0.322016	0.196251	0.268444
NAT9 TMEM104	-0.27875	-0.28359	-0.54002	-0.35567	-0.53085
NAV1	-0.25272	0.078561	0.189892	-0.56199	0.07458
NAV2	-0.16291	-0.18132	-0.08974	-0.07967	0.046331
NAV3	-0.52968	-0.48499	-0.74793	-0.51864	-0.82572
NBL1	-0.01414	0.284597	0.44194	0.139878	0.363674
NBN	-0.10006	-0.1866	-0.00839	-0.26723	-0.13523
NBPF14	0.357601	0.764628	0.021525	-0.29978	-0.02583
NBPF15	0.717734	0.7989	0.811098	0.423074	0.636777
NBPF3	-0.12212	-0.3369	-0.3185	-0.02433	-0.14354
NBPF4	0.405045	-0.11111	0.023374	-0.06983	-0.19377
NCALD	-0.9416	-0.19542	-0.44995	-0.66196	-0.56729
NCAM1	-0.19335	0.120865	-0.15835	-0.19576	0.023469

NCAM2	-0.08393	0.193003	0.087044	0.147455	0.359935
NCAN	0.097283	-0.44464	0.205856	0.308384	0.132732
NCAPD3 VPS26B	-0.2744	0.012204	-0.19017	-0.04187	-0.17822
NCAPG2	-0.0039	0.121523	-0.00883	0.01475	-0.08753
NCAPH	-0.16469	-0.29118	-0.1688	-0.12944	0.152923
NCBP2	-0.19047	-0.19322	-0.30534	-0.12453	-0.11213
NCF1C.	0.423728	0.591936	0.70425	0.365597	0.596402
NCF2	0.001391	-0.15201	-0.30487	-0.32275	-0.29592
NCF4	0.033159	0.088414	-0.13543	-0.14515	-0.04014
NCK1	-0.34602	-0.42607	-0.40723	-0.14558	-0.20761
NCKAP1	0.294756	0.108074	-0.08006	-0.05949	0.166784
NCKAP1L	0.231358	-0.51715	-0.78302	-0.78385	-0.53158
NCKIPSD	0.190942	0.114867	-0.06045	0.255059	0.131745
NCL	-0.26444	-0.17617	-0.24466	-0.23227	-0.16877
NCLN	-0.23622	-0.31944	-0.39994	-0.33956	-0.37135
NCOA1	-0.27678	-0.32757	-0.50086	-0.54971	-0.63918
NCOA2	-0.27777	-0.15763	-0.17535	-0.21848	-0.20895
NCOA3	-0.02895	-0.37286	-0.70074	-0.46197	-0.60693
NCOA4	-0.15054	-0.0796	-0.20532	-0.11475	-0.34885
NCOA5	0.25721	0.200577	0.300908	-0.07554	-0.26685
NCOA6	0.294273	0.01189	-0.03977	-0.02686	-0.0137
NCOR1 PIGL	0.219369	0.405893	0.440424	0.342046	0.361108
NCOR2	-0.30176	-0.66605	-0.68375	-0.52761	-0.63872
NCR1	0.241218	-0.52604	-0.46757	-0.38346	-0.43707
NCR2	0.220932	0.170663	0.263758	0.11147	0.057919
NCR3	0.155555	0.350381	0.049941	-0.12379	0.328551
NCUBE1	0.244984	0.161606	0.140647	0.138259	0.026716
NDE1	0.12563	0.092227	0.164185	0.082613	0.064788
NDEL1	-0.09272	-0.02902	-0.07862	-0.23576	-0.1319
NDFIP1	0.324272	0.481434	0.387499	0.318759	0.418282
NDFIP2	0.037348	0.044512	-0.08801	-0.1025	-0.1717
NDN	0.239765	-0.02815	0.456048	0.215556	0.312872
NDNL2	-0.56905	-0.32158	-0.54055	-0.14885	-0.54428
NDP	0.023383	0.846819	0.088175	0.546682	-0.00125
NDRG1	-0.39924	-0.44767	-0.18338	-0.35491	-0.41761
NDRG2	-0.16481	0.233354	0.258955	0.120653	0.299397
NDRG3	0.248669	0.42761	0.373826	0.32125	0.252193
NDST1	0.41097	0.531179	0.719003	0.330555	0.435273
NDST2 AC022400.9	-0.18888	-0.09954	-0.00019	-0.10012	-0.20877
NDST3	-0.54312	-0.57147	-0.42028	-0.21727	-0.3995
NDST4	-0.09069	0.519633	-0.22478	-0.54006	-0.85392
NDUFA10	0.149219	0.035126	0.075076	0.194909	0.150339
NDUFA12	0.134545	0.131933	-0.04227	0.035253	-0.10994
NDUFA3	0.165587	0.096845	0.026778	-0.07381	0.025653
NDUFA4	-0.27817	-0.13753	-0.42355	-0.18652	-0.31056
NDUFA4L2	-0.19379	-0.48279	-0.31373	-0.35731	-0.49803
NDUFA5	-0.22506	-1.37909	-1.68744	-1.07758	-1.66359
NDUFA6	-0.21203	0.056424	-0.08486	-0.08095	-0.26102
NDUFA8 C9orf18	-0.04371	-0.39231	-0.37246	-0.43594	-0.60898
NDUFA9	-0.76327	-0.35459	-0.40078	-0.65061	-0.67517

NDUFAB1	0.07319	-0.0132	0.001653	-0.02952	0.05362
NDUFAF1	0.188063	0.259894	-0.58095	-0.03812	-0.25685
NDUFB1 CPSF2	-0.29301	-0.09451	-0.62026	-0.30823	-0.34748
NDUFB10	0.137838	0.05554	-0.0388	0.10622	-0.02806
NDUFB11 RBM10	0.008085	-0.39597	0.088705	-0.35496	-0.34723
NDUFB2	0.157902	0.220392	0.239607	-0.00524	0.225517
NDUFB4	-0.18077	-0.17826	-0.32833	-0.162	-0.30232
NDUFB6	-0.16772	-0.03493	-0.17189	-0.27542	-0.27978
NDUFB7	0.126698	0.136761	-0.11326	-0.02599	0.142858
NDUFC2	0.062987	0.163273	-0.01902	-0.17879	-0.08053
NDUFS1 EEF1B2	-0.22678	-0.08772	-0.12169	-0.06247	-0.26056
NDUFS4	-0.35884	-0.49033	-0.69481	-0.41233	-0.7352
NDUFS5	0.193853	0.179899	-0.06653	-0.10883	-0.01472
NDUFS7	0.050677	0.054553	-0.11451	-0.02147	0.088126
NDUFS8	0.292071	-0.18183	-0.35441	-0.02862	-0.00299
NDUFV2	-0.04348	0.02558	-0.05007	-0.10693	-0.04752
NDUFV3	0.253714	0.068771	0.109143	0.233942	0.124183
NEB	0.36504	0.496184	0.333605	0.207478	-0.13946
NECAB1	0.393588	0.545181	0.497657	0.363857	0.40382
NECAP2	-0.03959	0.013546	0.110916	-0.03144	0.080386
NEDD1	0.07322	0.061217	0.348529	0.002515	-0.16919
NEDD4L	-0.09587	0.049221	-0.06179	0.031921	0.015918
NEDD8 GMPR2	-0.41773	-0.35138	-0.56205	-0.48772	-0.55354
NEDD9	-1.03005	0.283999	-0.07521	0.033793	0.068961
NEF3	0.091737	0.182915	-0.16652	-0.21715	-0.306
NEFH	0.062336	0.2416	0.376363	0.272699	0.348769
NEFL	-0.45339	-0.36471	-0.44889	-0.3272	-0.40878
NEGR1	-0.2397	-0.1478	-0.18791	-0.18142	-0.06692
NEIL1	0.559883	1.014325	0.790399	0.518316	0.411952
NEIL2	0.158574	-0.19358	-0.19676	-0.09932	-0.01532
NEIL3	0.249096	0.199563	0.14014	-0.09098	0.004358
NEK1	-0.91654	-1.05726	-1.15297	-0.86812	-1.14432
NEK2	0.249802	-0.27268	-0.30787	0.027166	-0.0435
NEK3	0.25816	0.435891	0.378181	-0.00676	0.092994
NEK4	-0.04054	0.184865	-0.06668	-0.09085	0.062052
NEK5	0.240802	0.388266	0.47295	0.319125	0.483793
NEK6	0.217794	0.39759	0.496456	0.307734	0.450973
NEK7	-0.05237	-0.01082	-0.25935	-0.76199	-0.45041
NEK9	0.309987	-0.05993	-0.2563	0.245928	0.218801
NELF PNPLA7	0.049824	-0.06477	-0.06285	0.108867	0.084365
NELL1	-0.17731	0.169833	0.022035	-0.06413	-0.00346
NELL2	-0.42298	-0.35952	-0.305	-0.45789	-0.27263
NENF	0.114945	0.132967	0.120515	0.094752	0.156616
NEO1	0.007475	-0.03529	-0.13371	-0.06793	-0.1051
NES	-0.1839	-0.52238	-0.05328	-0.44051	0.216867
NET1	-0.22776	0.795109	0.85398	0.439904	-0.41148
NETO1	0.029936	0.112986	0.096575	-0.03629	0.143321
NETO2	0.177685	0.223735	0.325848	0.204204	0.254553
NEU1 AL671762.10	-0.43563	-0.24482	-0.88956	-0.33758	-0.77295
NEU2	0.429457	0.739807	0.811562	0.437885	0.435503

NEU3	0.264339	0.26972	0.167998	-0.08032	0.158609
NEU4	0.184596	0.372933	0.364504	0.240009	0.30606
NEURL	0.068513	0.148098	0.230343	0.159421	0.196107
NEURL2 CTSA	-0.35667	-0.50337	-0.48503	-0.3579	-0.51413
NEUROD1	-0.77492	0.26882	0.168199	0.202778	0.11304
NEUROD2	-0.13313	-0.24126	-0.20442	-0.41661	-0.28615
NEUROD6	0.422641	-0.97532	-1.16111	0.1752	-0.81283
NEUROG1	-0.0808	-0.10853	0.382865	0.424508	0.438218
NEUROG2	-0.10364	-0.1726	-0.11745	-0.1037	-0.14756
NEUROG3	0.081193	-0.00378	0.343387	0.329073	0.386161
NF2	0.107337	0.074853	0.186735	0.116139	0.170405
NFAM1	0.315523	0.378494	0.675937	-0.04327	0.358251
NFASC	-0.02363	-0.10246	-0.78515	-0.41687	0.00354
NFAT5	-0.05351	-0.05293	0.209245	0.189572	-0.1828
NFATC2	0.058509	-0.06995	-0.07907	0.086678	0.194222
NFATC2IP	-0.03108	-0.06213	0.106582	0.102923	-0.06021
NFATC3	-0.08647	-0.1583	-0.02021	-0.20541	-0.14651
NFATC4	-0.67788	-0.79289	-0.60554	-0.47813	-0.68801
NFE2	0.237422	0.325126	0.356466	-0.04399	0.058953
NFE2L1	-0.46945	-0.61759	-0.67039	-0.52713	-0.515
NFE2L2	-0.29736	-0.27083	-0.36103	-0.35266	-0.11726
NFE2L3	0.397522	0.407933	0.72339	0.343373	0.256444
NFIA	0.000311	-0.14606	-0.03435	-0.09243	-0.0236
NFIB	-1.00882	-0.70306	-0.99403	-0.67444	-0.81303
NFIL3	0.09974	0.070356	0.110474	0.011426	0.10376
NFIX	0.207035	0.299082	0.251346	0.3217	0.401421
NFKB1	0.167404	-0.12416	-0.0206	-0.14611	-0.22297
NFKB2	-0.36816	-0.65029	-0.5439	-0.41735	-0.55046
NFKBIA	0.151035	0.089381	0.047099	0.120284	0.157059
NFKBID HCST	-0.09929	0.279378	0.093186	-0.14239	0.14847
NFKBIE	0.106194	0.058072	0.163143	0.122254	0.062499
NFKBIL2 GPT	0.183098	0.048848	0.188503	0.036849	0.209307
NFRKB	0.454017	0.532189	0.480917	0.128175	0.452667
NFS1 C20orf52	-0.31119	-0.3487	-0.65313	-0.39615	-0.68236
NFU1	0.287674	0.248199	0.213324	0.204608	0.308332
NFX1	-0.01458	0.098534	0.031987	0.102227	-0.16646
NFXL1	-0.51083	-0.35578	-0.39662	-0.32943	-0.55952
NFYB	0.202741	0.375319	0.381016	-0.21662	0.323268
NGB	0.358192	0.463299	0.598497	0.310097	0.371957
NGEF	0.458676	0.538978	0.358539	0.214578	0.302959
NGF	-0.0196	0.141986	0.31024	0.207743	0.071703
NGFR	-0.28799	-0.13678	-0.20737	0.063398	-0.0188
NGFRAP1	-0.02644	-0.12239	-0.00749	-0.1184	-0.17592
NGLY1	-0.23158	0.118225	-0.08177	-0.15782	0.036071
NGRN	-0.21166	-0.23054	-0.25474	-0.22929	-0.18134
NHEDC1 NHEDC2	0.562773	0.311923	0.994337	0.626075	0.163955
NHEDC2	0.274507	0.061837	-0.09793	-0.15259	-0.03915
NHEJ1	0.025942	-0.0449	0.025633	-0.1663	-0.04147
NHEJ1 SLC23A3	0.253832	-0.00362	0.226293	-0.01713	-0.04521
NHLH1	0.234262	-0.00669	0.193696	-0.71988	-0.11237

NHLH2	0.297875	-0.34158	-0.08355	-1.0029	-0.64239
NHLRC1	0.505847	0.598195	0.748863	0.508909	0.53989
NHP2L1	0.458469	0.447614	-0.05417	0.118586	0.566701
NHP2L1 CTA-216E10.9	0.059697	0.323136	0.230596	0.079244	0.158125
NHS	0.213139	0.022712	0.33835	0.156059	-0.07228
NHSL2	0.148355	0.180796	0.316946	0.226022	0.164297
NICN1	0.102318	0.254079	0.121689	0.110613	0.020686
NID2	-0.32915	0.156873	-0.35417	0.106076	0.084328
NINJ1	0.016651	0.114311	0.041145	0.016711	-0.02452
NINJ2	0.451214	0.750943	0.940836	0.593021	0.538216
NIPA1	-0.05618	0.012304	0.139081	0.213029	0.017994
NIPA2	-0.09684	-0.09275	0.078385	0.197622	-0.15119
NIPBL	-0.68204	-0.77308	-0.80061	-0.63244	-0.5191
NIPSNAP1	0.098071	0.107715	0.137479	0.009595	-0.17336
NIPSNAP3A	-0.31694	-0.41805	-0.27212	-0.40982	-0.41927
NIPSNAP3B	0.367722	0.302359	-0.08176	0.389838	0.226096
NIT1 DEDD	-1.03762	-1.06751	-1.28435	-1.0375	-0.99259
NIT2	0.418931	0.63174	0.362194	0.392911	0.544794
NKAIN1	0.19223	0.091001	0.298455	0.199893	0.272027
NKAIN3	-0.33903	0.454349	0.519974	-0.24375	0.123733
NKAP	0.230697	0.336627	-0.03311	0.021628	0.180311
NKAPL	0.416267	0.782919	0.842446	0.536607	0.485165
NKD1	-0.36473	-0.40533	-0.48287	-0.18086	0.130108
NKD2	0.038495	0.113969	0.107957	0.076291	0.217327
NKG7	-0.16342	-0.22332	-0.39048	-0.4193	-0.3282
NKIRAS1 RPL15	-0.43997	-0.47404	-0.49454	-0.41299	-0.36256
NKPD1	0.127282	-0.03764	0.445401	0.203072	0.264827
NKRF	-0.08296	-0.08347	0.06705	-0.06818	-0.04588
NKTR	-0.16017	0.038355	-0.13367	-0.18468	-0.24075
NKX2-2	-0.82155	0.084153	0.514466	0.475163	0.486047
NKX2-3	-0.3386	0.007562	0.071676	0.32026	0.433337
NKX2-5	-0.37734	-0.3433	-0.09883	0.170474	-0.08628
NKX2-8	-0.42677	-0.40823	0.530368	0.426967	0.398725
NKX3-1	-0.04652	-0.04684	0.328765	0.162381	0.171031
NKX6-1	-0.41836	-0.34975	-0.2623	-0.04975	-0.14573
NKX6-2	0.033426	0.199501	0.141431	0.161623	0.361049
NKX6-3	0.315426	0.615514	0.702524	0.394127	0.446236
NLE1	0.015756	0.256857	0.286011	0.235222	0.031507
NLGN2	0.100277	0.401209	0.293277	0.155564	0.126609
NLGN2 SPEM1	0.410155	0.414351	0.488996	0.428682	0.49039
NLGN3	0.200998	0.449777	0.246178	0.196522	0.164114
NLGN4X	-0.61073	-0.17404	-0.5582	-0.64801	-0.24083
NLGN4Y	-0.14633	0.018929	0.141687	-0.13057	0.095852
NLK	-0.13884	-0.13213	-0.27821	-0.1429	-0.10095
NLRC3	0.189068	0.216561	0.350331	0.082569	0.165389
NLRC4	0.341011	0.294473	0.407994	-0.19388	-0.40105
NLRC5	-0.06191	0.000816	-0.40814	-0.25349	-0.23537
NLRP1	0.287417	0.202219	0.179345	-0.10113	0.275267
NLRP10	0.22395	-0.37213	-0.78892	-0.63273	-0.02738
NLRP11	0.679256	-1.28701	-1.02224	-0.60534	0.104195

NLRP13	0.198703	-0.54626	-0.78212	-0.41045	0.079201
NLRP14	-0.57116	-0.8766	-1.18363	-1.20301	-1.05554
NLRP2	0.450798	0.18707	0.797177	0.481598	0.489149
NLRP3	0.354216	-0.13328	0.010505	0.096407	0.151965
NLRP4	0.546653	0.455836	0.833058	0.235715	0.235963
NLRP7	0.394963	-0.25514	-0.45682	-0.16317	0.219483
NLRP8	0.255268	-0.90566	-0.91665	-0.16083	-0.68241
NLRP9	0.136705	-0.74382	-0.83012	-0.5521	-0.70471
NMB	0.221824	0.149118	0.066485	0.239838	0.071117
NMBR	0.159033	0.152023	0.209289	0.418693	0.502677
NMD3	0.085582	0.103066	0.002103	-0.17347	-0.15828
NME3 MRPS34 EME2	-0.01853	-0.02284	0.090641	0.003738	-0.01501
NME4	0.158415	0.176856	0.108161	0.073192	-0.02178
NME5 BRD8	-0.19781	-0.40658	-0.5864	-0.18261	-0.65131
NME6	-0.074	0.543229	-0.16326	-0.05699	0.15474
NME7 BLZF1	-1.40887	-1.62951	-2.03341	-1.83824	-1.7698
NMI	0.238672	-0.17999	0.472158	0.31496	0.192071
NMNAT3	0.302198	0.695029	0.891666	0.200881	0.590721
NMRAL1 HMOX2	0.350836	0.415087	0.281612	0.348971	0.365549
NMS	0.315316	-0.50023	0.23648	-0.16286	-0.00956
NMU	0.215457	0.54242	0.003654	0.435578	0.207619
NMUR1	0.396487	0.572527	0.427907	0.401502	0.580394
NMUR2	0.488893	0.244176	0.478801	0.263279	-0.04377
NNMT	-0.02079	0.232688	0.5775	0.143629	-0.18487
NNT	-0.31655	-0.32384	-0.48069	-0.36819	-0.53324
NOB1	0.170976	0.126289	0.161093	-0.02357	0.371311
NOC2L KLHL17	0.083068	0.069337	0.136785	0.181348	0.145581
NOC3L	0.140897	0.332095	0.134216	0.005985	0.108623
NODAL	-0.01118	0.335549	0.537005	0.403838	0.36093
NOG	-0.10859	-0.06201	0.03965	-0.07737	0.066998
NOL1	-0.26743	-0.30986	-0.73916	-0.25171	-0.41746
NOL10	-0.0033	0.101935	-0.05686	-0.02038	-0.13928
NOL11	-0.59931	-0.74319	-1.00373	-0.87151	-0.85397
NOL12	-0.004	0.145491	-0.0604	0.142738	0.176387
NOL14 GRK4	-0.15161	-0.13644	-0.13595	-0.3071	-0.24992
NOL3	-0.06452	0.075587	0.192266	-0.05529	-0.11429
NOL4	-0.67516	-0.5436	-0.6082	-0.54844	-0.38854
NOL5A	-0.09394	0.003932	0.055189	-0.00058	-0.07211
NOL6	-0.16047	-0.62022	-0.90331	-0.4873	-0.66867
NOL7	-0.0207	0.118105	-0.06376	-0.15168	-0.03406
NOL8 CENPP	-0.66291	-0.63639	-0.7807	-0.57869	-0.6986
NOL9 TAS1R1	0.332105	0.276206	0.404876	0.345218	0.299646
NOLA1	-0.1086	-0.36942	-0.32963	-0.25893	-0.42582
NOLA2	0.374953	0.51694	0.481431	0.312658	0.072019
NOLA3 C15orf55	0.320481	0.408998	0.391814	0.203525	0.279602
NOLC1	-0.48281	-0.36688	-0.22242	0.173447	-0.30336
NOM1	-0.07893	0.004605	-0.02274	-0.00266	0.057927
NOMO1	-0.23792	0.036121	-0.20293	-0.25933	-0.23847
NOMO2	-0.28197	-0.02269	-0.40915	-0.24541	-0.18853
NOMO3	0.119675	0.007041	-0.07372	-0.27289	-0.33456

NONO	0.06452	-0.41519	-0.45555	-0.27187	-0.49155
NONO ITGB1BP2	0.247655	0.453804	0.576398	0.209004	-0.05844
NOS1	0.140642	-0.27614	0.286083	0.14963	0.03664
NOS1AP	0.031122	0.137814	0.08504	0.06583	0.115911
NOS2A	0.308897	0.357574	0.504011	0.237232	0.17765
NOS3	0.577879	0.673577	0.612376	0.343979	0.393562
NOSIP	0.168857	-0.31773	0.286991	-0.00384	0.241457
NOSTRIN	0.275716	0.333014	0.562087	0.155597	0.13154
NOTCH1	-0.15426	-0.02714	-0.15077	-0.00356	0.030681
NOTCH2	-0.53558	-0.45726	-0.51682	0.13107	-0.11481
NOTCH2NL	-0.23602	-0.55282	-0.53714	0.063698	-0.08747
NOTCH3	-0.06243	-0.11472	-0.06929	-0.02941	-0.03634
NOTCH4	0.002339	-0.53549	-0.51168	-0.51191	-0.15424
NOTUM	0.14409	0.283816	0.295219	0.269598	0.260747
NOV	-0.21639	0.486721	0.17746	-0.36159	0.002991
NOVA1	-0.59312	-0.43051	-0.64506	-0.27209	-0.6384
NOVA2	-0.28499	-0.3808	-0.22943	-0.26714	-0.18147
NOX1	-0.58893	0.045251	-0.71414	-0.36136	-0.27609
NOX3	0.4085	0.537414	0.873484	0.243968	0.408108
NOX4	-0.30139	0.312015	-0.23058	-0.06701	-0.37284
NOX5	0.379433	0.452301	0.66109	0.426207	0.52053
NOXO1 GFER	0.11756	0.116157	0.301815	0.166513	0.135428
NPAL1	0.198393	0.111296	-0.06909	0.076502	-0.1343
NPAL2	0.40634	0.094333	0.139078	0.135612	0.135631
NPAL3	0.267817	0.625964	0.313854	0.385396	0.194689
NPAS1	-0.27168	-0.03601	-0.12243	0.153517	0.048435
NPAS2	-0.11923	-0.0444	0.252174	0.147385	0.344165
NPAS3	-0.19022	-0.13807	-0.17571	-0.14667	-0.08226
NPAS4	0.114498	-0.14779	-0.5843	-0.08338	-0.4603
NPAT ATM	-1.15451	-0.7345	-1.22803	-0.86384	-1.34046
NPB	0.320644	0.526169	0.367147	0.264419	0.357813
NPBWR1	0.241953	0.480538	0.781839	0.416467	0.651105
NPC1	-0.28397	-0.32447	-0.30408	-0.15662	-0.2254
NPC1L1	0.174935	0.288469	0.553292	0.20869	0.299353
NPC2 ISCA2	-0.44548	-0.28666	-0.52436	-0.28498	-0.24383
NPDC1	0.137572	0.039648	-0.1123	-0.03161	0.010936
NPEPPS	0.155206	0.09246	-0.08642	-0.08831	0.076516
NPFF	0.362389	0.441121	0.12907	0.248297	0.064359
NPHP1	-0.32411	-0.87817	-0.86127	-0.29152	-0.47009
NPHP4 KCNAB2	-0.13462	-0.07914	-0.21799	-0.03186	-0.02067
NPHS1	0.327453	0.247899	0.549679	0.324708	0.233112
NPHS2	0.341374	-0.06046	0.460973	0.455115	0.414659
NPL	0.320568	0.476288	0.28374	-0.14529	0.011127
NPM1	-0.84076	-0.43239	-0.91799	-0.53333	-0.3761
NPM2	0.240668	0.334385	0.459068	0.38032	0.340441
NPM3	-0.09736	-0.1483	-0.52769	0.144378	-0.24823
NPNT	0.12957	0.059157	0.307096	0.199566	0.371641
NPPC	0.114299	0.107818	0.345732	0.211677	0.148961
NPR1	-0.54448	-0.74714	-0.41555	-0.19807	-0.6733
NPR2	0.169396	0.128959	0.182549	0.092572	0.167897

NPR3	-0.44095	0.203124	0.249498	0.001343	0.378964
NPSR1	-0.54888	-0.41858	-0.82149	-0.61778	-0.80131
NPTN	-0.13867	0.118446	0.122517	-0.07385	-0.00226
NPTX1	0.152564	0.152078	0.241017	0.11856	0.291413
NPTX2	0.325866	0.37841	0.502616	0.37549	0.527534
NPY	-0.11274	0.061637	0.262002	0.293409	0.157364
NPY1R	-0.18826	-0.46284	-0.04702	-0.5918	-0.51261
NPY2R	-0.22773	-0.11361	0.17229	0.289608	-0.38307
NPY5R	0.233028	-0.02509	0.496648	0.190359	0.225102
NQO1	-0.08947	-0.48151	-0.22096	-0.03779	-0.05762
NQO2	0.07313	0.036403	0.055757	0.10519	0.148992
NR0B1	0.224293	0.176031	0.337097	0.176052	0.098772
NR0B2	0.226862	0.24916	0.212362	0.23403	0.233421
NR1D1	-0.55971	0.194957	-0.47688	-0.3425	-0.24756
NR1D2	-0.2188	0.023057	-0.42148	-0.46822	-0.2274
NR1H2	-0.13454	-0.17894	-0.32277	-0.18107	-0.15154
NR1H3	0.177528	0.420046	0.552977	0.361746	0.29394
NR1H3 MADD	-0.02231	0.040792	-0.00906	0.06367	0.198501
NR1H4	0.077063	-0.74626	0.018235	-0.06683	-1.16262
NR1I3 AL590714.27	-0.00171	0.008651	-0.27471	-0.07355	-0.27029
NR2C1	0.112195	0.122228	0.137868	0.063322	-0.00068
NR2C2	-0.25645	-0.23449	-0.48342	-0.19108	-0.16158
NR2C2AP	0.101951	-0.29199	-0.38423	-0.06331	-0.07066
NR2E1	-0.80766	0.187079	0.144987	0.514955	-0.28872
NR2E3	0.208548	0.233784	0.313101	0.170931	0.296439
NR2F1	-1.5012	-1.47889	-1.22189	-1.01461	-1.42242
NR2F2	-0.24501	-0.46714	-0.33645	-0.40256	-0.23758
NR2F6	-0.02292	-0.12894	0.071622	0.126879	-0.05374
NR3C2	-0.2704	-0.30822	-0.1294	-0.06308	-0.07809
NR4A2	-0.10379	-0.30549	0.137471	-0.05992	0.123494
NR5A1	0.26073	0.398681	0.462115	0.292955	0.41336
NR5A2	-0.0428	-0.73581	-0.23688	-0.48203	-0.37034
NR6A1	-0.42426	-0.17777	-0.47417	-0.30934	-0.2913
NRAP	0.283491	0.45446	0.482448	0.063088	0.01389
NRAS CSDE1	-0.7526	-0.59598	-0.81788	-0.92267	-1.00864
NRBF2	0.388367	0.300327	0.468314	0.138409	-0.01642
NRBP1	0.143671	0.206678	0.256528	0.118964	0.060629
NRBP1 KRTCAP3	0.494367	0.652781	0.655615	0.485656	0.702242
NRBP2	0.310969	0.422513	0.464321	0.174318	0.313193
NRD1	-0.37917	-0.31166	-0.83177	-0.50531	-0.2505
NRF1	-0.07712	0.013333	-0.12962	-0.08474	0.038657
NRG2	-0.047	0.024519	-0.15958	-0.12985	-0.07908
NRG3	0.009832	0.014627	0.083614	0.108463	0.220762
NRG4	0.22268	-0.208	0.203876	-0.34223	-0.44771
NRGN	0.256708	0.139015	0.248007	0.076026	0.229225
NRIP1	0.378153	-0.83887	-0.50259	0.00052	0.13942
NRIP2	0.043176	-0.16812	-0.30254	-0.1288	-0.42291
NRIP3	0.019645	0.03496	0.104939	0.21853	0.156273
NRK	0.29528	-0.19908	0.214841	0.4804	0.483192
NRL	0.214868	0.109686	-0.34353	-0.09929	-0.01932

NRL WDR23	-0.95362	-0.62562	-1.06529	-0.78848	-0.86957
NRM	0.161376	0.318335	0.272905	0.020539	-0.00602
NRN1	-0.7257	-0.68421	0.0098	0.220078	-0.53151
NRP1	-0.32063	-0.13298	-0.11432	-0.14359	-0.07521
NRP2	0.00467	0.505341	0.271088	0.136052	0.385315
NRSN1	0.097642	-0.04926	0.08651	-0.08766	-0.17052
NRSN2	-0.30027	-0.3199	-0.31184	-0.22874	-0.25097
NRTN	0.444395	0.376591	0.400973	0.486944	0.418807
NSBP1	0.042294	-0.65222	0.408793	0.60856	-0.37843
NSBP1 SH3BGRL	-0.07651	-0.15981	-0.16418	-0.18567	-0.2277
NSD1	0.04166	-0.07201	0.068915	0.02854	-0.14234
NSFL1C	0.53223	0.493716	0.655993	0.009033	0.132505
NSL1 TATDN3	-0.07027	-0.47318	-0.67046	-0.50283	-0.46153
NSMAF	0.009129	0.080877	-0.06373	-0.08439	-0.01481
NSMCE4A	0.309724	0.237159	0.42709	0.248821	0.377425
NSUN2 SRD5A1	-0.15196	-0.17185	-0.19938	-0.09101	-0.15218
NSUN4	0.065273	-0.10803	-0.29684	-0.0583	0.014632
NSUN5	0.345339	0.522491	0.441696	0.318227	0.450642
NSUN5B	0.166246	0.592742	0.339251	0.300101	0.383647
NSUN5C	0.207033	0.210067	0.006354	0.185052	0.161214
NSUN6	-0.34703	-0.34857	-0.46453	-0.49481	-0.56217
NSUN7	0.344599	0.397067	0.246847	0.160369	0.162014
NT5C	0.236975	0.111255	0.225647	0.137445	0.271622
NT5C1A	-0.09582	0.229801	-0.19245	0.123804	-0.04127
NT5C2	0.048641	-0.00493	0.051284	-0.04184	0.151934
NT5C3	0.457316	0.518899	0.486919	0.088704	0.3006
NT5C3 BBS9	-0.10551	-0.21237	-0.17885	-0.05659	0.090235
NT5C3L KLHL10	0.203632	0.272267	0.233583	0.224246	0.207008
NT5DC3	-0.18457	0.031066	-0.18698	0.229852	0.146619
NT5E	-0.28189	0.077948	0.092824	0.295811	-0.00761
NT5M	0.137504	0.193956	0.052955	0.07466	0.272058
NTF3	0.439903	-0.30572	0.080769	0.264378	0.508355
NTF4	-0.02386	-0.53798	-0.07584	-0.28815	0.112734
NTHL1 TSC2	0.14183	-0.22883	-0.34768	-0.06965	-0.20302
NTN1	0.026982	0.207278	0.067637	0.097283	0.077373
NTN2L	0.083352	0.062429	-0.07722	0.081073	0.154522
NTN4	-0.20081	0.12582	-0.1843	-0.04737	-0.05819
NTNG1	-1.24298	0.49082	-0.41822	-0.13972	0.229764
NTNG2	0.549132	0.559469	0.903001	0.411205	0.672864
NTRK1	-0.15043	-0.40147	-0.43451	-0.39891	-0.28428
NTRK2	-0.08106	-0.11814	-0.11868	0.127656	0.114749
NTRK3	-0.62659	-0.32091	-0.4831	-0.47087	-0.49086
NTS	-0.91571	-0.60957	-0.61061	-0.75812	-0.92321
NTSR1	0.096861	0.310895	0.511875	0.15049	0.261687
NTSR2	0.261334	0.603762	0.579029	0.416385	0.505335
NUAK1	0.051519	0.480164	0.385722	-0.03121	0.411948
NUAK2	-0.03755	0.186379	0.225229	-0.0952	0.162667
NUB1	0.229843	0.323103	0.356	0.198848	0.238948
NUBP1	-0.57038	-0.51322	-0.96485	-0.8848	-0.99797
NUBPL	0.332817	-0.01328	-0.26447	-0.07244	-0.15654

NUCB2	0.052394	-0.03648	-0.13709	0.080884	0.089834
NUCKS1	-0.43082	-0.51412	-0.62873	-0.36871	-0.3891
NUDC	0.070894	-0.05456	-0.40309	-0.4609	-0.07648
NUDCD1 ENY2	-0.31797	-0.36666	-0.30198	-0.47322	-0.38406
NUDCD2 HMMR	-0.68528	-0.55631	-0.53112	-0.47381	-0.4636
NUDCD3	-0.47475	-0.55339	-0.80613	-0.47985	-0.5755
NUDT10	0.226441	0.245567	0.333827	0.193013	0.092166
NUDT11	-0.02302	0.037758	0.178869	0.085033	-0.04205
NUDT12	0.147306	0.141128	0.096134	-0.11824	0.51023
NUDT13	0.557167	0.591097	0.482543	0.286118	0.423707
NUDT14	-0.0334	-0.05665	0.025052	0.092843	0.218382
NUDT15	-0.12617	-0.01509	-0.21977	-0.11438	-0.0839
NUDT16	-0.08212	0.254417	-0.11188	0.141029	0.037762
NUDT16L1	0.004908	0.105946	0.133144	0.037039	0.096194
NUDT16P.	0.424346	0.590722	0.798208	0.439003	0.589222
NUDT17	0.142937	-0.2547	-0.06723	0.065667	-0.19074
NUDT18	0.215968	0.067579	-0.14261	-0.06435	0.18378
NUDT21 OGFOD1	-0.76685	-0.86175	-1.0339	-0.67038	-0.81127
NUDT3	0.024488	0.082696	0.078033	0.132635	-0.00954
NUDT4	-0.02232	0.061228	0.060884	0.293761	0.061026
NUDT5 CDC123	-1.11012	-0.875	-1.29054	-0.82176	-1.06059
NUDT6 SPATA5	-0.57729	-0.61644	-0.47414	-0.36435	-0.39989
NUDT8	-0.13042	-0.16171	-0.11897	-0.13546	-0.09523
NUDT9	-0.20318	-0.19162	-0.21185	-0.20316	-0.13281
NUF2	-0.45758	-0.60902	-0.84935	-0.62115	-0.54582
NUFIP1 KIAA1704	-0.21936	-0.39826	-0.44349	-0.38319	-0.61598
NUFIP2	-0.71567	-0.47829	-0.6598	-0.61288	-0.37904
NUMA1 LRRC51	-0.12478	-0.09798	-0.61576	-0.05141	-0.39779
NUMBL	-0.27437	-0.31893	-0.22694	-0.20784	-0.2058
NUP107	-0.33191	-0.32687	-0.75077	-0.60977	-0.65264
NUP133	0.11575	0.107026	-0.21684	-0.14733	-0.19078
NUP153	-0.21761	-0.29195	-0.17529	-0.196	-0.30673
NUP155	-1.13566	-1.00341	-0.43203	-0.44643	-1.09152
NUP160	-0.13051	0.061052	0.031773	0.037437	-0.18383
NUP205	0.046017	-0.23219	0.068419	-0.04007	-0.98587
NUP210	0.044098	0.177409	0.001178	0.02075	0.116234
NUP210L	0.251366	0.423913	0.756585	-0.45348	-0.21021
NUP35	0.214476	-0.25516	-0.69627	-0.48105	-0.29977
NUP37 C12orf48	0.078522	-0.02614	-0.14203	-0.18109	-0.01384
NUP43	-0.05908	-0.00614	-0.22391	-0.21674	-0.30151
NUP50	-0.10553	-0.21902	-0.25863	-0.12678	-0.3036
NUP54	-0.73734	-0.60503	-0.85966	-0.5846	-0.72406
NUP62CL CXorf41	0.238211	0.199878	0.522214	0.134031	-0.13517
NUP85	-0.22494	-0.45666	-0.6925	-0.46367	-0.38976
NUP88 RPAIN	-0.43274	-0.15439	-0.70932	-0.36121	-0.24638
NUP98 AC090587.1	0.089874	0.038159	0.057864	0.045529	0.201464
NUPL1	0.287431	0.305114	0.398562	0.28306	0.302817
NUTF2	0.099895	0.000522	0.086772	0.055591	0.201455
NVL	-0.06513	-0.19213	-0.44979	-0.41498	-0.10417
NXF1	0.065663	0.065777	-0.61031	-0.2474	0.072988

NXF3	0.111305	0.284586	0.00022	0.162208	0.328833
NXF5	0.229923	0.328239	-0.07044	0.164375	-0.14537
NXN	-0.14829	-0.2186	-0.32649	-0.21746	-0.25474
NXNL1	0.30018	0.352094	0.66533	0.492148	0.410208
NXNL2	-0.78116	-0.62353	-0.87665	-0.50244	-0.30852
NXPH1	-0.10044	-0.00596	0.413759	-0.01169	-0.1118
NXPH3	-0.16752	-0.32756	-0.59835	-0.2004	-0.11067
NXPH4	-0.09563	-0.01371	0.051263	-0.05209	0.009968
NXT1	-0.1213	-0.09825	-0.01315	-0.05333	-0.14641
NXT2	0.671634	0.735592	0.412137	0.340299	0.562108
NYX	0.356475	0.525336	0.063329	0.195806	0.099171
OAF	-0.00396	0.158392	0.059198	0.200581	0.281809
OAS1	-0.37401	-0.50853	-0.05672	0.000249	-0.45323
OASL	0.366253	0.06639	0.114747	-0.06468	-0.06358
OAT	0.251222	0.200277	-0.07935	0.034652	0.10492
OAZ1	-0.41877	-0.34588	-0.48593	-0.41909	-0.43435
OAZ3	0.430855	0.620037	0.557164	0.440168	0.368514
OBFC1	-0.23352	-0.24532	-0.37521	-0.21499	-0.38644
OBFC2A	-0.03727	-0.26003	-0.24766	-0.09666	0.043594
OBFC2B	0.301132	-0.03325	0.438933	0.243608	0.0581
OBP2A	0.203394	-0.02222	0.392129	0.262209	0.345357
OBP2B	0.209544	-0.03661	0.436721	0.334431	0.392614
OCA2	0.136519	0.102586	-0.04445	-0.13155	0.041289
OCEL1	-0.02489	-0.1001	-0.42269	-0.07356	-0.23008
OCIAD1	0.070031	-0.10934	-0.37087	-0.40846	-0.23367
OCIAD2	0.064462	0.318234	0.167295	0.091511	0.025926
OCLN	-0.10334	0.193195	0.157875	0.175729	0.149207
OCM	0.357133	0.691175	0.488122	0.22979	0.544243
OCRL	0.316196	0.162049	-0.25895	-0.08796	-0.12835
ODC1	0.173506	0.207706	0.397445	0.086869	0.355273
ODF1	0.474376	0.665903	0.567002	0.547924	0.663161
ODF2	0.148362	0.201339	0.024163	0.187117	-0.00311
ODF2L	-0.10767	-0.10185	-0.15086	-0.49944	-0.22262
ODF3	-0.28699	-0.96332	-1.04767	-0.33517	-0.60418
ODF3L1	0.022135	0.160355	0.249221	-0.0787	-0.01452
ODF4	0.225708	0.229751	0.473737	0.090282	0.15845
ODZ1	-0.20796	0.433001	-0.59236	0.09431	-0.11869
OGDH	-0.01815	-0.27545	-0.56929	0.094414	-0.2045
OGDHL	0.093765	-0.04659	-0.23283	-0.14144	-0.15182
OGFOD2 ARL6IP4	0.082975	0.093152	0.025824	0.152318	0.028187
OGFR	0.090405	0.078531	-0.00864	0.095024	0.143313
OGFRL1	-0.01345	0.121501	0.224866	0.040886	0.348775
OGG1	0.164575	0.159231	0.072642	-0.04456	0.179553
OGT	-0.01552	-0.23934	0.07481	-0.43369	-0.47753
OIP5 NUSAP1	-0.24855	-0.23036	-0.31493	-0.12401	-0.38844
OIT3	0.31021	0.321966	0.506387	0.115672	0.133386
OLA1	0.301599	0.296755	0.439334	0.195748	0.500312
OLAH	0.033936	0.121933	0.15048	-0.15655	-0.4917
OLFM1	0.146101	-0.06327	0.056516	-0.05915	-0.04625
OLFM2	-0.32833	-0.43451	-0.12873	-0.036	-0.07795

OLFM3	-0.57771	-0.12722	-0.76003	-0.69452	-0.85619
OLFM4	-0.03492	0.265601	0.373833	0.111697	-0.32418
OLFML1	-0.09499	0.218732	0.229227	-0.13693	0.32766
OLFML2A	0.344483	0.351034	0.682774	0.413293	0.441256
OLFML2B	-0.21149	0.105996	-0.10365	0.178083	-0.18881
OLFML3	-0.04033	-0.35731	-0.51126	-0.50414	-0.57334
OLIG1	0.038179	0.143011	0.491331	0.25743	0.353659
OLIG2	-0.50292	-0.45161	0.253829	0.383313	0.346926
OLIG3	-0.65242	0.045362	0.230109	0.406073	0.589638
OLR1	0.693326	-0.86495	-0.8809	-0.48842	0.155229
OMA1	-0.40637	-0.52057	-0.54529	-0.46728	-0.24583
ONECUT1	-0.19765	-0.03461	-0.0514	0.055068	0.193156
ONECUT2	-0.26351	-0.30888	-0.3936	-0.30588	-0.05836
OOEP DDX43	0.503028	0.55293	0.776938	0.595128	0.727076
OPA1	0.015966	0.29109	0.078047	0.124027	0.040543
OPA3	-0.0453	-0.30271	-0.40926	-0.38792	-0.26578
OPHN1	0.183043	0.260432	0.311685	0.118724	0.387703
OPLAH	0.307001	0.234654	0.517298	0.407723	0.42927
OPN1LW	0.281837	0.292437	0.269531	0.321098	0.304433
OPN1MW	0.189171	0.207431	0.007415	0.039641	0.362192
OPN1SW	0.159329	0.281403	0.139624	0.160371	0.171295
OPN4	0.089847	0.127974	0.129803	0.117426	0.17018
OPRD1	0.253373	0.372014	0.558706	0.275989	0.362566
OPRK1	0.108282	-0.15282	0.396893	0.250361	0.325395
OPRM1	-0.06976	0.346443	-0.39656	0.164712	-0.21537
OPRS1	-0.17373	-0.03465	-0.42106	-0.22862	-0.08869
OPTC	0.187612	0.296561	0.37276	0.204902	0.16721
OR10A2	-0.20767	-0.6397	-0.90753	-0.61396	-0.45622
OR10A3	0.15825	-0.79081	-0.86395	-0.6284	-0.14621
OR10A4	-0.47773	-0.69881	-0.79486	-0.46711	-0.43125
OR10A5	-0.02324	-1.2057	-1.58023	-1.4269	-1.03347
OR10A6	-0.17289	-0.61314	-0.5849	-0.30311	-0.18295
OR10A7	-0.36358	-0.77515	-0.84955	-0.81528	-0.94497
OR10AD1	0.275412	0.557195	0.691813	-0.33549	-0.70869
OR10AG1	-0.0952	-0.63527	-0.44009	-0.37827	-0.23373
OR10G2	0.353251	-1.66187	-1.94277	-1.36129	-1.37058
OR10G3	0.282392	-0.64194	-0.75573	-0.63243	-0.82129
OR10G4	0.265989	-0.39262	-0.70885	-0.40367	-0.55555
OR10G7	0.340423	-0.04581	-0.66496	-0.41716	0.048162
OR10G8	0.455911	-0.39701	-0.82676	-0.47196	0.001413
OR10G9	0.44808	-0.38756	-0.92946	-0.61951	-0.71615
OR10H1	0.322482	-0.82369	-0.10987	0.308116	0.598552
OR10H2	0.520994	-0.89838	-0.3952	0.218546	0.317233
OR10H3	-0.45542	-1.29104	-1.20033	-1.1177	-1.46548
OR10H4	-0.6701	-1.42731	-1.4388	-1.23074	-1.42763
OR10H5	0.541671	-0.86956	-0.04668	0.307618	0.707917
OR10K1	0.133886	-0.98861	-0.96775	-0.51575	-0.3872
OR10K2	-0.4241	-2.15337	-1.6166	-1.72006	-1.4921
OR10P1	0.168492	-0.20113	-0.85092	-0.50066	-0.36957
OR10Q1	0.604289	-0.04607	0.34791	0.442759	0.397746

OR10R2	-0.71893	-1.30466	-0.98054	-1.07742	-1.06695
OR10S1	0.199649	-0.25113	-0.85445	-0.58111	-0.91602
OR10T2	-0.46772	-0.72933	-0.5182	-0.51145	-0.34845
OR10V1	0.113198	-0.91597	-0.66687	-0.79614	-0.57611
OR10X1	-0.41086	-0.72596	-0.50826	-0.65653	-0.482
OR10Z1	-0.98991	-0.97228	-0.97389	-0.96969	-0.89359
OR11G2	-0.2072	-0.93736	-0.6626	-0.64998	-0.66917
OR11H1	-0.83123	-1.35631	-1.14074	-1.27738	-1.09695
OR11H13P.	-0.90283	-1.6241	-1.26566	-1.0601	-1.04746
OR11H4	-0.38603	-1.26044	-0.58484	-0.96411	-0.93239
OR11H6	-0.58558	-0.63461	-0.62028	-0.40732	-0.53815
OR11L1	0.29012	-0.7662	-0.19012	-0.17051	0.088161
OR13C2	-0.65735	-0.47386	-1.34138	-0.90989	-1.52665
OR13C3	0.471013	0.630895	-0.65432	0.400487	-0.02
OR13C4	-0.59669	-0.42732	-0.98303	-0.56721	-1.1205
OR13C5	-0.65264	-0.50887	-1.40789	-0.8621	-1.93167
OR13C8	0.538405	0.524874	0.114396	0.454844	0.1569
OR13C9	-0.7875	-0.58657	-1.14554	-0.76224	-1.19478
OR13D1	-0.29242	-0.18974	-0.3499	-0.3276	-0.3651
OR13F1	0.212908	0.385647	-0.41463	0.14292	-0.37689
OR13G1	-0.60608	-1.3461	-1.41172	-1.099	-0.90577
OR13H1	-0.72734	0.045531	-0.52975	-0.23205	-0.6681
OR13J1	0.326265	0.598278	0.577211	0.346342	0.561231
OR14K1.	0.086038	-1.15042	-1.0372	-0.82871	-0.93921
OR1A1	-0.11055	-0.4241	-0.40194	-0.42633	-0.32862
OR1A2	-0.27754	-0.49998	-0.8929	-0.83056	-0.63426
OR1B1	0.269653	-2.6629	-1.95301	-1.36296	-1.68144
OR1D2	-0.15775	-0.38504	-0.62708	-0.56075	-0.38929
OR1D4	0.218156	-1.29766	-1.56469	-1.2232	-1.26229
OR1E1	-0.24844	-0.38141	-0.6847	-0.67777	-0.42802
OR1E2	-0.15993	-0.84796	-1.23883	-1.01647	-0.6036
OR1F1	0.069019	-1.01342	-0.36016	-0.18781	-0.15311
OR1G1	-0.39252	-1.24299	-1.74705	-1.47457	-1.11883
OR1I1	0.325785	-0.74363	0.320178	0.057771	0.44285
OR1J1	0.101753	-0.20044	0.201868	-0.16277	-0.25922
OR1J2	0.051757	-0.86984	-0.56333	-0.72837	-0.91307
OR1J4	0.026817	-0.43992	-0.10746	-0.4779	-0.52207
OR1K1	0.783059	0.590425	0.803461	0.264706	0.492962
OR1L1	0.130409	-0.70411	-0.68863	-0.45836	-0.72052
OR1L3	-0.35669	-1.37684	-1.07415	-0.93965	-0.99979
OR1L4	-0.4528	-0.91161	-0.8883	-0.58999	-0.96852
OR1L6	0.638302	0.257527	0.447498	0.217989	0.148324
OR1L8	0.075289	-0.8199	-0.84943	-0.60917	-0.93348
OR1M1	0.288569	-0.44177	-0.38088	0.073155	0.137635
OR1N1	0.240013	-0.83429	-0.54484	-0.38806	-0.95861
OR1N2	-0.47695	-0.94772	-0.71745	-0.69747	-0.76016
OR1Q1	-0.36591	-0.86033	-0.84939	-0.82686	-0.9201
OR1S1	0.098847	-1.50597	-1.45549	-1.06459	-0.81402
OR1S2	0.122357	-1.32213	-1.36672	-1.02501	-0.68042
OR2A1	0.340431	-0.28275	-0.64965	0.035052	-0.17029

OR2A5. ARHGEF5	0.29843	0.546864	0.181989	0.246755	0.193309
OR2AE1	0.041292	-0.38994	-0.88644	-0.41555	-0.46002
OR2AG1	-0.11703	-0.57283	-0.97888	-0.74193	-0.29866
OR2AG2	0.33652	-0.72411	-1.12254	-0.50153	-0.1372
OR2AT4	-0.43487	-0.22781	-0.57022	-0.66955	-0.68005
OR2B11	0.462308	0.311922	0.209417	0.42832	0.489366
OR2B2	0.189683	0.200486	0.226604	-0.07829	0.038051
OR2B3	-0.87242	-0.95347	-0.73566	-0.66711	-0.98428
OR2B6	-0.20683	-0.05156	-0.2269	-0.25412	-0.38241
OR2C1	0.520994	-0.66761	-0.14961	-0.03934	-0.25766
OR2D2	0.364419	-1.72158	-1.59508	-1.3927	-1.18138
OR2D3	-0.43546	-0.37206	-0.49978	-0.39069	-0.1592
OR2F1	0.041492	-0.87112	-0.68987	0.030455	-0.33218
OR2G2	0.447363	-0.58422	-0.26183	-0.32819	-0.07922
OR2G3	-0.13561	-1.12017	-1.08166	-1.01283	-0.90471
OR2G6	0.084854	-0.89437	-0.46797	-0.64861	-0.19294
OR2H1	-0.14229	-1.06748	-1.19657	-0.79091	-0.70179
OR2K2	0.345313	0.174036	0.499446	0.093981	-0.1833
OR2M2	0.100971	-1.93986	-1.24702	-1.32481	-1.26844
OR2M3	-0.24291	-1.72909	-1.40879	-1.41053	-1.51413
OR2M4	-0.01206	-1.65499	-1.24639	-1.13498	-1.43024
OR2M5	-0.11681	-1.467	-0.81307	-1.09751	-0.95976
OR2M7	-0.36401	-1.19849	-0.8145	-0.96184	-0.85013
OR2S2	0.217936	0.002428	0.198452	0.058476	-0.29315
OR2T1	0.519198	-0.44258	0.562231	-0.14526	0.125107
OR2T10	-0.022	-0.92139	-0.53016	-0.74899	-0.49055
OR2T11	0.454004	-2.64519	-1.52382	-1.21759	-0.31479
OR2T12	0.330809	-0.21177	-0.06169	0.119434	-0.25778
OR2T2	-0.21631	-1.2049	-0.78094	-0.99826	-0.69451
OR2T27	-0.01107	-2.18312	-0.78254	-1.56434	-1.57566
OR2T29	0.078154	-1.53628	-1.0981	-1.23791	-0.9231
OR2T3	-0.04746	-1.73995	-1.24373	-1.14344	-0.98691
OR2T33	0.310117	-0.4124	-0.34724	0.007051	-0.04451
OR2T34	-0.03647	-1.77177	-1.14611	-1.24069	-0.93464
OR2T35	-0.18772	-1.22051	-0.9738	-1.02221	-0.78703
OR2T4	-0.1956	-0.81243	-0.43515	-0.46565	-0.39254
OR2T5	0.253646	-1.72114	-1.35971	-1.2939	-1.12472
OR2T6	-0.17807	-1.76303	-1.03453	-1.25772	-1.06676
OR2V2	0.388646	-0.31267	-0.19155	-0.02604	0.23347
OR2W1	-0.41388	-0.43095	-0.41262	-0.4486	-0.74928
OR2W5	-0.00464	-1.507	-1.27634	-0.64535	-0.73822
OR2Y1	0.347058	-0.90192	-0.54806	0.019425	-0.22822
OR2Z1	0.497045	-1.59598	-1.281	-0.63212	-0.10136
OR3A1	0.537989	-0.31926	-0.31006	-0.1314	0.12243
OR3A3	0.158753	-0.48289	-0.81783	-0.39493	-0.27784
OR3A4	0.683418	-0.90041	-1.41054	-0.59943	-0.32372
OR4A15	-0.54513	-0.92831	-0.6226	-0.58655	-0.4381
OR4A16	-0.36659	-1.38442	-1.06544	-0.93563	-0.79401
OR4A4P.	-1.0799	-1.2582	-1.00724	-0.90014	-0.9813
OR4B1	0.105542	-1.3327	-1.17927	-0.89732	-0.94717

OR4C11	-0.24557	-0.43863	-0.41866	-0.30454	-0.36829
OR4C13	-1.26812	-1.5157	-1.25072	-0.97456	-0.75387
OR4C15	-0.74505	-0.82084	-0.63962	-0.70886	-0.26869
OR4C16	-0.74619	-0.90426	-0.64815	-0.82164	-0.74401
OR4C3	-1.60828	-1.55367	-1.70604	-1.28333	-1.17078
OR4C46	-0.64797	-0.99537	-1.1096	-0.90491	-0.60762
OR4C6	-0.70185	-0.98734	-0.88661	-0.76295	-0.50632
OR4D1	0.008684	-1.08316	-0.94774	-1.12737	-0.75814
OR4D11	0.037055	-0.73685	-0.87877	-0.73321	-0.61071
OR4D2	-0.0737	-0.83295	-0.53729	-0.74688	-0.80493
OR4D5	0.204012	0.312271	0.080087	-0.24408	-0.07593
OR4D6	-0.02093	-1.23968	-1.08164	-0.9753	-0.60242
OR4D9	0.834448	-0.30125	-0.45704	-0.49899	-0.14472
OR4F15	-0.40774	-1.24307	-0.72306	-0.73435	-1.31313
OR4F16	-0.63775	-1.43819	-0.6181	-1.09912	-1.8396
OR4F17	0.088949	-0.88961	-0.16634	-0.62615	-1.09764
OR4F21	-0.66804	-1.41354	-0.61387	-1.03811	-1.91311
OR4F29	-0.61615	-1.41813	-0.67415	-1.05896	-2.02285
OR4F4	0.194492	-0.90817	-0.17439	-0.52652	-1.11328
OR4F5	0.128998	-1.07235	0.009745	-0.40325	-0.77871
OR4F6	-0.47028	-0.91137	-0.69038	-0.65592	-0.79982
OR4K1	-0.10391	-0.78344	-0.72616	-0.68151	-0.85451
OR4K13	-0.28315	-1.07033	-0.82143	-0.72775	-0.9052
OR4K14	-0.42941	-0.90236	-0.66482	-0.66296	-0.57705
OR4K15	-0.67034	-1.99205	-1.75689	-1.64253	-1.16158
OR4K17	-0.39211	-0.53892	-0.1524	-0.35593	-0.33476
OR4K2	-0.62292	-0.5079	-0.6635	-0.7132	-0.86586
OR4K5	-0.93021	-1.00583	-0.79434	-0.67108	-1.08232
OR4L1	-0.44759	-0.29073	-0.27695	-0.25311	-0.4432
OR4M1	-1.2063	-2.41508	-2.13022	-1.70886	-2.38363
OR4N2	-0.70885	-1.87134	-1.45354	-1.20225	-1.72019
OR4N5	-0.80361	-1.15519	-1.06194	-1.07986	-1.10442
OR4P4	-0.4796	-0.75591	-0.65382	-0.34664	-0.41861
OR4Q3	-0.68722	-0.91071	-0.86812	-0.58999	-1.18298
OR4S1	-0.03139	-1.78945	-1.44212	-1.35404	-0.64517
OR4S2	-0.07325	-0.51072	-0.41738	-0.34256	-0.19182
OR4X1	-0.64684	-1.33609	-1.20919	-0.88258	-0.78019
OR4X2	-1.50367	-1.44202	-1.28845	-1.25916	-1.02456
OR51D1	-0.16354	-1.20768	-1.55465	-1.17187	-0.48813
OR51E1	-0.39308	-0.60677	-0.55356	-0.56143	-0.34328
OR51E2	0.079205	-0.55272	-0.41126	-0.45294	-0.23517
OR51L1	-0.10614	-1.22433	-1.04768	-1.16768	-0.64846
OR51V1	0.148331	-0.92455	-0.67648	-0.55102	-0.34432
OR52A1	-0.58048	-0.44551	-0.51376	-0.53632	-0.24813
OR52A4	-0.28098	-1.18142	-1.01716	-0.95321	-0.72422
OR52A5	0.294632	-0.69255	-0.54944	-0.6264	-0.31693
OR52E2	-0.116	-1.23474	-0.87197	-0.88112	-0.66339
OR52E4	-0.49876	-0.85963	-0.81189	-0.71705	-0.53776
OR52E5.	-0.50054	-1.04665	-1.19847	-0.8933	-0.79618
OR52I1	-0.00353	-0.35024	-0.20628	-0.33541	-0.36397

OR52I2	-0.59688	-1.25522	-1.08194	-1.07923	-0.82406
OR52J3	-0.01348	-0.85711	-0.64544	-0.5511	-0.40504
OR52K1	-0.45004	-1.09509	-1.0107	-0.8818	-0.65581
OR52K2	-0.69642	-1.18467	-1.0694	-0.93146	-0.48984
OR52L1	-0.63766	-0.9596	-0.84809	-0.83968	-0.57477
OR52M1	-0.08813	-1.14897	-0.69699	-0.64624	-0.56653
OR52W1	-0.21073	-0.64594	-0.49291	-0.43593	-0.33273
OR56A1	-0.36145	-1.28361	-0.97626	-0.93686	-0.53169
OR56A3	-0.54908	-0.57082	-0.42138	-0.47131	-0.24686
OR56A4	-0.00874	-0.95964	-0.87574	-0.76842	-0.65466
OR56B4	0.000136	-0.80818	-0.54202	-0.51887	-0.39993
OR5A1	0.046533	-0.0627	-0.18735	-0.17278	-0.11869
OR5A2	-0.62958	-1.06162	-0.75472	-0.72965	-0.49954
OR5AC2	-0.00651	0.083571	-0.29676	0.082498	-0.35591
OR5AK2	-0.41284	-0.1575	-0.40957	-0.56142	-0.2076
OR5AN1	-0.89403	-1.17703	-0.94004	-0.91603	-0.60533
OR5AP2	-0.32416	-1.15366	-0.902	-0.80493	-0.26048
OR5AR1	-0.1975	-0.34468	-0.37999	-0.51251	-0.01652
OR5AS1	-0.5757	-0.78384	-0.63576	-0.69612	-0.51652
OR5AT1	0.19839	-0.92969	-0.43945	-0.42409	-0.24014
OR5AU1	-0.4602	-1.02688	-0.91139	-0.84285	-1.07184
OR5B12	-0.06638	-1.1054	-0.88438	-0.86644	-0.48591
OR5B17	-0.26571	-0.64577	-0.47937	-0.50648	-0.42686
OR5B2	-0.52512	-0.56135	-0.58921	-0.48425	-0.27555
OR5B21	-0.24206	-0.77803	-0.70605	-0.54002	-0.431
OR5B3	0.021649	-0.50941	-0.49487	-0.48996	-0.29092
OR5BF1	-0.18216	-1.25876	-0.85809	-0.98706	-0.83696
OR5BU1	0.112689	-1.29891	-0.83519	-0.79918	-0.8841
OR5C1	0.51724	0.608124	0.821265	0.444526	0.765833
OR5D13	-0.41089	-0.59306	-0.23172	-0.29746	-0.09163
OR5D14	-0.69984	-0.7774	-0.79582	-0.651	-0.41018
OR5D16	-0.74634	-0.77123	-0.72148	-0.56649	-0.40364
OR5D18	0.158539	-0.32656	-0.18361	-0.14147	0.193895
OR5F1	-0.13437	-0.82062	-0.58065	-0.43288	-0.52027
OR5H1	-0.71211	-0.98007	-1.44022	-0.78496	-1.43546
OR5H14	-0.33174	-0.57306	-0.71853	-0.55721	-0.84659
OR5H15	-0.74903	-1.12964	-1.69574	-0.86327	-1.5247
OR5H2	-0.28389	-0.52743	-0.42852	-0.39789	-0.69737
OR5H6	-0.41176	-0.96952	-1.53354	-0.3935	-1.36829
OR5I1	-0.02631	-0.77817	-0.52848	-0.63933	-0.40729
OR5K1	-0.66372	-0.64345	-0.97136	-0.82983	-1.02826
OR5K2	-0.19097	-0.29923	-0.49408	-0.57008	-0.54694
OR5K3	-0.28054	-0.11437	-0.3507	-0.3069	-0.31032
OR5K4	0.093382	-0.00063	-0.58334	-0.29632	-0.23556
OR5L1	-0.46948	-0.54297	-0.4052	-0.41951	-0.22919
OR5L2	-0.42354	-0.69981	-0.52887	-0.51671	-0.36336
OR5M3	0.217374	-0.61175	-0.68911	-0.50706	-0.00824
OR5M8	0.421667	-0.64258	-0.65137	-0.34821	0.075011
OR5M9	0.244254	-0.31999	-0.30048	-0.24137	0.129376
OR5P2	0.219405	0.319913	0.027518	-0.19058	0.284102

OR5P3	-0.14763	-0.02927	-0.84096	-0.8129	-0.09138
OR5R1	0.06471	-0.64076	-0.59269	-0.59645	-0.37526
OR5T1	0.149821	-0.76894	-0.52215	-0.39195	0.092158
OR5T2	-0.6009	-0.6522	-0.49708	-0.55582	-0.34387
OR5T3	-0.78622	-0.8321	-0.73768	-0.81008	-0.48858
OR5W2	-0.52979	-0.39607	-0.54196	-0.49073	-0.42886
OR6A2	-0.1435	-0.72208	-0.9465	-0.77461	-0.49117
OR6B2	0.14283	-0.48525	-0.3367	-0.04709	-0.18906
OR6B3	0.315991	-0.67331	-0.40243	-0.15501	-0.10308
OR6C1	0.287663	-0.15469	-0.5071	-0.3908	-0.07697
OR6C2	-0.36821	-0.46254	-0.50721	-0.54017	-0.44593
OR6C3	-0.19108	-0.56685	-0.48231	-0.63125	-0.37994
OR6C4	-0.40723	-1.12758	-1.11369	-0.9077	-1.22647
OR6C65	0.366311	-0.40428	-0.42847	-0.45695	-0.3452
OR6C68	-0.33364	-0.71755	-0.69916	-0.8102	-0.75685
OR6C70	0.094742	-0.79872	-0.68832	-0.65677	-0.66439
OR6C74	-0.19635	-0.92669	-0.75904	-0.90352	-1.13001
OR6C75	0.431284	-0.2237	-0.3474	-0.30882	-0.08944
OR6C76	-0.10193	0.141978	0.141455	-0.31489	-0.37511
OR6F1	-0.02959	-1.02602	-0.92032	-0.56525	-0.31602
OR6K2	-0.28842	-1.37202	-1.21178	-1.02789	-0.91746
OR6K3	-0.03696	-0.66442	-0.44725	-0.66948	-0.54295
OR6K6	0.386621	-1.39431	-0.98669	-0.99069	-0.5536
OR6N1	-0.51873	-1.46992	-1.19931	-1.03743	-1.24819
OR6N2	-0.4477	-1.02251	-0.90922	-0.78024	-0.70325
OR6S1	0.383448	0.0043	-0.81553	-0.40942	-0.26479
OR6T1	0.19864	-0.22421	-1.07433	-0.49025	-0.51789
OR6Y1	-0.78159	-1.46471	-1.25481	-1.20655	-1.13388
OR7A10	0.102764	-0.76016	-0.88985	-0.66044	-0.80397
OR7A17	0.043798	-1.08316	-0.87815	-0.84322	-0.99386
OR7C1	0.252098	-0.64005	-0.51672	-0.72655	-0.62171
OR7C2	0.564804	-1.53655	-1.6595	-0.58603	-0.30344
OR7D4	0.119356	-0.65017	-0.28617	-0.44955	-0.19002
OR7G1	0.211287	-0.69096	-0.48736	-0.37197	-0.37497
OR7G2	0.389461	-0.78873	-0.52493	-0.32118	-0.43347
OR7G3	0.0506	-0.76181	-0.92171	-0.74296	-0.88349
OR8A1	-0.01286	-0.70003	-0.93025	-0.73594	-0.95966
OR8B12	-0.01561	-0.17896	-0.75989	-0.34433	-0.49641
OR8B2	-0.07332	-0.50929	-0.65445	-0.52634	-0.6474
OR8B3	0.100869	-0.33485	-0.28931	-0.2987	-0.29387
OR8B4	0.21836	-0.42355	-1.00567	-0.44948	0.362641
OR8B8	0.369354	-0.42355	-0.8567	-0.35245	0.218444
OR8D1	0.03364	-0.74372	-1.22864	-0.71765	-1.03957
OR8D2	-0.73569	-0.82522	-1.41187	-0.99063	-1.42697
OR8D4	0.049155	-0.19123	-0.37901	-0.36692	-0.66274
OR8H1	0.184643	-0.3063	-0.31154	-0.28879	0.092021
OR8H2	0.182462	-0.9688	-0.83349	-0.44606	-0.18779
OR8I2	-0.245	-0.77705	-0.65251	-0.53133	-0.28538
OR8J1	-0.86475	-0.71639	-0.75905	-0.65459	-0.49382
OR8K1	-0.47743	-0.7636	-0.79934	-0.80205	-0.37873

OR8K3	0.167366	-0.34064	-0.48767	-0.40579	0.015277
OR8S1	0.477903	0.512636	-0.382	-0.01889	-0.14945
OR8U8	-0.44653	-0.76277	-0.44876	-0.48	-0.40221
OR9A2	-0.28619	-1.15324	-1.66892	-0.31768	-1.22705
OR9G4	0.179487	-0.34396	-0.37752	-0.49335	-0.19706
OR9G9	-0.14849	-0.77109	-0.8031	-0.71762	-0.40849
OR9K2	-0.73663	-1.14803	-1.25414	-1.18132	-1.09987
OR9Q2	0.547739	-0.64165	-0.79074	0.03814	0.179617
ORAI1	0.052681	-0.02149	0.061527	0.064747	0.198561
ORAI2	0.056271	0.026805	0.28287	0.1349	0.065308
ORAOV1	0.262983	0.176851	0.275544	0.269664	0.261831
ORC1L PRPF38A	-0.75331	-0.69641	-0.89485	-0.68968	-0.3723
ORC2L	0.010116	-0.29392	-0.369	-0.20077	-0.18865
ORC4L MBD5	-1.22388	-1.2103	-1.21939	-1.05352	-0.71066
ORC5L	0.191221	-0.41507	-0.47449	-0.38888	-0.22569
ORM1	0.443982	0.62564	0.669881	0.621292	0.73303
ORMDL1 PMS1	-0.20873	-0.32292	-0.32479	-0.32331	-0.28344
ORMDL3	0.035857	0.073628	-0.06876	0.207885	0.285673
OSBP	-0.36944	-0.43299	-0.49473	-0.46459	-0.15648
OSBP2	0.115055	0.245891	0.192298	0.138111	0.170734
OSBPL10	0.316847	0.462453	0.492294	0.282282	0.239265
OSBPL11	-0.42044	-0.33341	-0.60161	-0.42817	-0.6545
OSBPL2	-0.21399	-0.10881	-0.15798	-0.04318	-0.2118
OSBPL3	-0.10727	-0.06133	-0.18123	0.071428	0.113168
OSBPL5	0.004413	0.091779	-0.01192	-0.02685	0.19816
OSBPL7	-0.38017	-0.50365	-0.69102	-0.60622	-0.44795
OSBPL8	0.129727	0.091258	0.080785	0.050901	0.122541
OSGEP NP	-0.56196	-0.4537	-0.80288	-0.53366	-0.59259
OSGIN2	0.270439	0.520423	0.48899	0.168093	0.524923
OSM	0.343296	0.559581	0.674673	0.320889	0.497441
OSMR	0.209098	0.130343	0.505724	0.348304	0.346774
OSR1	-0.24623	-0.09544	-0.02767	-0.00937	0.305323
OSR2	-0.56849	-0.41115	-0.19692	-0.41649	-0.03293
OSTM1	0.177035	0.393235	0.216407	0.093083	0.278292
OSTN	0.269808	-0.47711	-0.57389	-0.14271	-0.7631
OTOP1	0.340014	0.156322	0.265135	0.352364	0.218841
OTOP3	0.078164	0.016566	0.392725	0.163443	0.235197
OTOR	0.541299	0.459695	-0.25579	0.24904	-0.53699
OTOS	0.449256	0.10591	0.765675	0.121036	0.462555
OTP	0.203165	0.402406	0.528276	0.173023	0.315697
OTUB1	0.104884	0.142747	0.113788	0.19082	0.190956
OTUB2	0.395101	0.292674	0.250182	0.225306	0.152462
OTUD4	0.72185	0.799908	0.889949	0.321378	0.52455
OTUD5	0.027891	-0.04712	-0.15984	-0.12049	-0.04272
OTUD6A	0.473125	0.442511	0.697644	0.335869	0.611449
OTUD6B	-0.75006	-0.48453	-0.83175	-0.93841	-0.7725
OTUD7A	0.264423	0.274672	0.389811	0.084765	0.102673
OTUD7B	-0.2037	-0.37707	-0.44882	-0.46176	-0.2694
OTX1	-0.3426	-0.23281	0.229323	0.046371	-0.06604
OVCH1	0.346292	0.605726	0.470566	0.408912	0.474492

OVCH2	0.124009	-0.3238	-0.60893	-0.36434	-0.16486
OVGP1	0.077858	-0.13115	-0.11944	-0.28111	-0.32879
OVOL1	0.084645	0.228368	0.478822	0.288813	0.338019
OVOL2	-0.40517	-0.0674	0.207698	0.21288	-0.05638
OXA1L	0.004763	0.235708	0.196638	0.039708	-0.01996
OXCT1	0.148854	0.092961	0.141317	0.046023	0.284879
OXER1	0.069472	0.344553	0.383446	0.03285	0.488637
OXGR1	0.20848	0.583649	0.689634	0.530989	0.232271
OXR1	-0.5119	-0.51451	0.0619	-0.42667	0.14205
OXSR1	-0.12642	0.002152	-0.31248	-0.05911	-0.22427
OXT	0.492779	0.507287	0.425838	0.267705	0.237831
OXTR	0.559711	0.543813	1.097677	0.556494	0.661257
P2RX1	0.175284	0.195121	0.331891	0.099831	0.221935
P2RX2	0.048835	0.087276	0.340845	0.130207	0.430238
P2RX4	0.289394	0.23134	0.250195	0.301014	0.314139
P2RX5	-0.069	-0.03278	0.494288	0.189414	0.190409
P2RX7	0.072725	-0.5834	-0.65797	-0.21029	-0.62938
P2RY10	-0.78558	-0.48722	-0.71078	-0.30953	-0.40781
P2RY2	0.147615	0.168691	0.270606	0.312752	0.26841
P2RY4	-0.21232	0.030497	-0.18035	0.11463	-0.11418
P2RY8	0.420425	0.387281	0.322393	0.291108	0.378626
P4HA1	0.128385	0.059121	-0.06027	-0.00774	-0.09765
P4HA2	0.135773	0.269251	0.351685	0.269239	0.322259
P4HA3	0.358723	0.547159	0.567581	0.503008	0.520244
P4HB	-0.3128	-0.43	-0.40732	-0.33882	-0.20908
PA2G4	-0.28119	-0.38603	-0.53498	-0.41122	-0.34189
PABPC1	-0.05811	-0.101	-0.12045	-0.20141	-0.05677
PABPC1L2A	0.244912	0.404441	0.365744	0.20408	0.464872
PABPC3	0.287603	0.655772	0.563508	0.335193	0.49933
PABPC4	0.028391	0.082745	0.14191	0.087236	0.210985
PABPC5	0.12138	0.053948	0.284504	-0.0494	0.319234
PABPN1	0.452336	0.564522	0.503122	0.215864	0.49082
PACS1	-0.04971	-0.08855	0.075229	0.114311	-0.04257
PACS2	0.052525	0.106091	0.190846	0.114224	0.205233
PACSIN1	0.174818	0.237999	0.287571	0.337103	0.401526
PACSIN2	-0.33938	-0.07691	-0.1754	-0.34276	-0.54903
PACSIN3	-0.27766	-0.30128	-0.39083	-0.16386	-0.07201
PADI1	0.384414	-0.72824	0.230029	-0.11065	0.142121
PADI2	0.234945	0.185857	0.459567	0.481427	0.266814
PADI3	0.386691	0.536167	0.577877	0.343443	0.394111
PADI4	0.286976	-0.98079	0.249092	-0.04398	0.159601
PADI6	0.088596	-0.04063	0.310565	-0.00992	0.102121
PAEP	0.211054	0.254525	0.322548	0.122637	0.175168
PAF1 MED29	-0.18441	-0.10773	-0.3883	-0.25805	-0.37186
PAFAH1B1	-0.08649	0.039051	0.05522	-0.16293	-0.22739
PAFAH1B2	-0.27192	-0.08618	-0.37652	-0.26451	-0.59035
PAFAH1B3 PRR19	0.030078	0.129213	-0.18423	-0.14292	-0.02182
PAFAH2	-0.15147	-0.04182	-0.03375	0.171036	-0.02156
PAG1	0.133315	0.31295	0.090591	0.140851	0.262476
PAGE1	0.531807	0.452353	0.611159	0.315167	0.487281

PAGE3	0.335764	0.466885	0.153422	0.208109	0.345055
PAGE4	0.28543	0.256004	-0.21484	-0.08601	0.04174
PAGE5	0.679278	0.8154	0.84377	0.51228	0.579523
PAH	0.150869	0.3564	0.53394	0.391163	0.345056
PAIP1	0.118914	0.284926	0.118707	0.072166	0.223971
PAIP2	-0.20517	-0.35658	-0.3617	-0.17957	-0.52021
PAK1	-0.15314	-0.19968	-0.2285	-0.04685	-0.20547
PAK2	0.21239	0.304479	0.356872	0.160703	0.182138
PAK3	-0.01247	-0.30456	-0.29923	-0.35441	0.095337
PAK4	-0.25716	-0.13341	-0.06602	0.075562	-0.09702
PAK7	-0.33518	-0.38829	-0.6112	-0.46145	-0.57431
PALB2 DCTN5	-0.14435	-0.29072	-0.21354	-0.2844	-0.17984
PALLD	-0.02914	0.227855	0.319467	-0.0089	0.238573
PALM	-0.00448	0.134298	0.114323	-0.02117	0.008632
PALMD	0.334787	0.111039	0.092393	0.034875	0.179616
PAM	-0.4657	-0.53997	-0.47327	-0.30071	-0.40515
PAN2	-0.14624	-0.41655	-0.70128	-0.37561	-0.58525
PAN3	-0.25066	-0.39005	-0.83028	-0.65072	-0.75149
PANK1	-0.28328	-0.05614	-0.16895	-0.26068	-0.10664
PANK2	-0.25287	-0.11065	-0.22105	-0.12238	-0.251
PANK3	-0.17818	-0.39956	-0.46683	-0.43974	-0.67274
PANK4	-0.07749	0.084144	-0.0142	0.006511	0.147786
PANX1	-0.29757	-0.16095	-0.37372	-0.27116	-0.24353
PANX2	0.228679	0.270829	0.139127	0.163183	0.184543
PANX3	0.485611	0.323951	0.250308	0.037197	0.254468
PAOX	0.077499	0.2628	0.231625	0.120446	0.165719
PAPD1	-0.11726	-0.14534	-0.36299	0.07965	-0.03957
PAPD4	-0.12318	-0.33913	-0.09718	-0.25207	-0.40839
PAPLN	-0.01484	0.135736	0.43552	0.259847	0.288698
PAPOLA	-0.27995	-0.12797	-0.11132	-0.2116	-0.23403
PAPOLG	-0.11548	-0.77686	-1.19099	-0.87848	-0.65537
PAPPA	-0.32583	-0.47199	-0.00287	-0.51867	-0.43495
PAPPA2	0.220285	0.106292	0.193761	-0.02045	-0.0176
PAPSS1	-0.0251	0.131998	0.057931	0.0723	0.142149
PAPSS2	0.275476	0.307712	0.3355	0.332137	0.528518
PAQR3	0.022181	0.231633	0.122562	0.016025	0.181125
PAQR5	0.100088	0.235171	0.334336	0.177697	0.167787
PAQR6	0.107307	0.345278	0.267953	0.298637	0.272787
PAQR7	0.067022	-0.02005	0.040299	-0.27752	-0.11842
PAQR8	-0.21171	-0.24378	-0.1838	-0.01671	-0.67932
PAQR9	0.053013	0.060908	0.104946	0.181679	0.098692
PARD3	-0.06254	-0.07766	-0.08687	-0.01194	-0.15701
PARD3B	0.087783	0.303258	0.288658	0.207308	0.181528
PARD6B	0.014133	0.301087	0.081182	0.050653	-0.03964
PARD6G	0.106142	0.080877	0.247024	0.082414	0.205795
PARK2 PACRG	0.085147	0.15288	-0.11448	0.124054	-0.04099
PARK7	-0.29245	-0.20819	-0.28063	-0.0476	-0.11653
PARL	-0.08234	0.144207	0.127847	0.024105	-0.26182
PARP1	0.254626	0.267066	0.380454	0.243381	0.317874
PARP10	0.520142	0.550117	0.588866	0.354778	0.359308

PARP11	-0.17393	0.299974	-0.2301	0.047759	-0.32202
PARP12	0.547052	0.801901	0.849375	0.465535	0.454341
PARP14	-0.12973	0.103197	-0.08731	-0.39355	-0.7746
PARP15	0.164549	0.070418	0.273629	-0.17418	-0.98144
PARP16	-0.15138	-0.36094	-0.50071	-0.39691	-0.32206
PARP4	-0.22708	-0.09307	-0.20135	-0.20872	-0.22776
PARP6	0.427564	0.640143	0.546018	0.09046	0.229013
PARP8	-0.62417	-0.58043	-0.53433	0.087533	-0.54506
PARP9 DTX3L	0.198991	0.028199	0.502793	0.113279	0.173613
PARS2	-0.3724	-0.20262	-0.47475	-0.44559	-0.24094
PARVA	0.046892	0.411692	0.467091	0.237588	0.309967
PARVG	0.440185	0.23755	0.310506	0.073447	0.136989
PASD1	0.472018	0.700053	0.563518	0.469231	0.543747
PASK PPP1R7	-0.01328	0.126871	0.031591	0.011002	-0.04831
PATL1	0.483225	0.694397	0.703269	0.107927	0.17411
PATZ1	-0.2363	-0.15456	-0.22948	-0.05715	-0.19477
PAWR	-0.29857	-0.15842	0.198681	-0.05667	0.257953
PAX2	-0.75133	-0.65169	-0.29667	0.090799	-0.77038
PAX3 CCDC140	-0.43982	-0.12578	0.086023	0.207577	0.204077
PAX4	-0.03305	-0.33889	-0.30992	0.005452	-0.35968
PAX5	-0.33188	-0.1821	0.577094	0.090861	0.480353
PAX6	-0.60292	0.180483	-0.01626	-0.21512	-0.30613
PAX7	-0.42096	-0.16935	0.334115	0.17051	0.224454
PAX8	0.384641	0.485731	0.319568	0.083389	0.257769
PAX9	-0.45537	0.326808	0.121752	0.5096	0.650512
PAXIP1	0.100968	0.233638	0.156337	0.144564	0.228356
PBEF1	-0.07089	-0.10863	-0.17786	-0.08723	-0.14415
PBK	0.385738	0.563003	0.297967	0.326097	0.406495
PBLD HNRPH3	-0.55893	-0.57161	-0.6778	-0.59095	-0.76271
PBRM1	-0.16369	-0.03583	-0.42798	-0.47921	-0.41971
PBRM1 GNL3	-0.59004	-0.3665	-0.56613	-0.6599	-0.4041
PBX2 GPSM3	-0.53422	-0.32807	-0.67512	-0.30781	-0.53103
PBX3	-0.06427	-0.09451	0.105087	0.025065	0.12064
PBX4	0.072486	0.214146	0.260563	0.221538	0.165212
PBXIP1	-0.94962	-0.90188	-1.31682	-1.06521	-0.63291
PCAF	0.127821	0.451683	0.34492	0.243516	0.297426
PCBD1	0.344459	0.456063	0.463987	0.171011	0.266592
PCBD2	0.135215	0.195563	0.190302	0.230324	0.25442
PCBP1	-0.85689	-0.78838	-0.78596	-0.63827	-0.48067
PCBP2	-0.34384	-0.21485	-0.42444	-0.28713	-0.44603
PCBP3	0.58349	0.783721	1.028974	0.603457	0.657936
PCCA	0.06523	0.058555	-0.01891	-0.04128	0.096861
PCCB	0.051148	0.108268	0.193752	0.014239	-0.03626
PCDH1	-0.37678	-0.37635	-0.30636	-0.21244	-0.43763
PCDH10	-0.76108	-0.77445	-0.61459	-0.26823	-0.94686
PCDH12	-0.09254	-0.25333	-0.31688	-0.28305	-0.17655
PCDH15	0.292016	0.085145	0.116865	0.053585	-0.16476
PCDH17	-1.34172	-1.11797	-0.77092	-0.75596	-1.07248
PCDH18	-1.22346	-1.53615	-1.24824	-0.97386	-1.3214
PCDH19	0.509705	0.665488	0.676753	0.652214	0.86861

PCDH20	-0.13318	0.082787	-0.18279	0.389155	-0.1694
PCDH21	0.060767	0.062919	0.308723	0.25766	0.274796
PCDH24	0.231425	0.332567	0.283503	0.243867	0.272384
PCDH7	-0.50344	-0.49897	-0.52718	-0.2709	-0.28258
PCDH8	-0.55619	-0.4475	-0.25233	-0.17608	-0.24433
PCDH9	-0.64924	-0.51777	-0.53859	-0.40246	-0.52098
PCDHA1	0.259571	0.663723	0.439872	0.401594	0.517142
PCDHB1	0.196684	0.594308	0.783225	0.637687	0.066161
PCDHB10	-0.13087	0.067229	0.41341	0.077398	-0.00165
PCDHB11	-0.01067	0.599416	0.737278	0.361692	0.49461
PCDHB12	-0.33906	0.210472	0.29829	0.121306	0.340096
PCDHB13	-0.11951	0.637394	0.727523	0.438806	0.4717
PCDHB14	-0.52659	0.642606	-0.30788	0.240734	0.410328
PCDHB15	-0.23427	0.797487	0.91695	0.586522	0.55367
PCDHB16	-0.4412	0.366393	0.206172	-0.02431	0.019683
PCDHB2	-0.25743	0.531305	0.652792	0.345885	-0.05038
PCDHB3	0.204388	0.435786	0.054486	0.675608	0.802981
PCDHB4	0.241621	1.010454	0.512052	0.595637	-0.35859
PCDHB5	0.296108	0.198448	0.965458	0.456825	0.491915
PCDHB6	0.099486	0.600812	0.799595	0.788836	0.228242
PCDHB7	-0.20162	0.484989	0.268786	0.340392	0.098334
PCDHB8	0.055918	0.81238	0.813181	0.399775	0.542259
PCDHGA1	-0.28866	0.488166	0.242345	0.089789	-0.20753
PCF11	-0.8186	-0.50458	-1.07067	-0.97189	-0.97912
PCGF1	-0.16632	-0.32864	-0.40668	-0.38498	-0.17949
PCGF2	-0.29072	-0.15547	-0.24677	-0.34758	-0.29292
PCGF3	0.078284	-0.34609	-0.23192	-0.22521	-0.2365
PCGF5	-0.16964	-0.29796	-0.61302	-0.14687	-0.20945
PCGF6	0.352383	0.290593	0.37621	0.078267	0.261678
PCID2 CUL4A	-0.14162	-0.1262	-0.0172	0.041192	-0.06508
PCK1	0.357898	0.428719	0.549706	0.244216	0.272972
PCM1	-0.13996	0.006595	-0.06284	-0.13201	-0.1015
PCMT1	0.092671	0.200371	0.323705	0.047673	0.15796
PCMTD1	0.060385	0.210618	0.037247	-0.00294	-0.01217
PCMTD2	0.185888	0.136257	0.18228	0.120511	0.12138
PCNA	0.191527	0.149832	0.111401	0.08703	0.09496
PCNA CDS2	-0.00067	0.12404	0.108625	0.146758	0.151893
PCNP	-0.51797	-0.34497	-0.59639	-0.50144	-0.54594
PCNX	0.30387	0.289111	0.631941	0.24353	0.300403
PCNXL2	0.487721	0.799106	0.666855	0.414777	0.448473
PCOLCE	0.214617	-0.04023	-0.20891	-0.13237	0.057102
PCOLCE2	0.225208	-0.22041	0.24925	-0.01043	0.159686
PCP2	0.077254	-0.28666	-0.03811	-0.33527	-0.37999
PCP4	0.249869	0.134112	0.171733	0.165458	-0.1982
PCSK1	-0.54944	-0.22603	-0.25044	0.229892	-0.43978
PCSK1N	0.177435	0.074641	0.162898	-0.00241	-0.1283
PCSK2	-0.46525	-0.36641	-0.48494	-0.34155	-0.44282
PCSK4 REEP6	-0.10739	-0.25382	-0.218	-0.15671	-0.17149
PCSK5	-0.47031	-0.34305	0.057251	-0.31153	-0.34188
PCSK6	0.07036	0.289678	0.348277	0.299665	0.27907

PCSK7 RNF214	-0.04513	-0.22344	-0.47537	-0.1507	-0.35922
PCSK9	0.207755	0.053112	0.613077	0.188418	0.462766
PCTK1	0.077664	-0.15534	-0.33298	-0.18868	-0.10795
PCTK2	-0.05842	-0.06221	-0.24728	-0.2345	-0.24972
PCTK3	0.123493	-0.03264	0.222303	-0.01819	0.120425
PCTP	-0.19412	-0.29852	-0.31963	-0.5017	-0.26945
PCYOX1	0.140922	0.204916	0.091594	0.11417	0.065955
PCYOX1L	-0.1204	-0.0659	0.015741	-0.12121	-0.23084
PCYT1A	-0.06792	0.020802	-0.2194	-0.14401	-0.13213
PCYT1B	0.212496	-0.35778	-0.89513	-0.66926	-0.16354
PCYT2 SIRT7	0.3275	0.527417	0.377356	0.237143	0.251711
PDAP1 BUD31	-0.47143	-0.33474	-0.64416	-0.40351	-0.50909
PDCD1	0.110008	0.053983	0.196407	-0.0302	0.083581
PDCD10 SERPINI1	-0.5757	-0.65349	-0.63584	-0.42978	-0.54135
PDCD1LG2	-0.65393	-0.43684	-0.19316	-0.33284	-0.19838
PDCD2	-0.08396	-0.01687	-0.00435	-0.03627	-0.04236
PDCD2L	0.376678	0.250252	0.089253	0.157869	0.188468
PDCD4	-0.16617	-0.29819	-0.39595	-0.1747	-0.0998
PDCD5	0.04827	-0.04356	-0.07879	-0.07492	-0.05525
PDCD6IP	-0.09012	0.08448	-0.25557	-0.43686	-0.21337
PDCD7	0.151681	0.469711	-0.03618	-0.03056	-0.41308
PDCL	-0.30714	-0.20214	-0.4828	-0.30727	-0.50356
PDCL3	0.003696	0.027813	0.01886	-0.01951	0.212555
PDDC1	-0.0159	0.015555	-0.03405	0.005732	0.071489
PDE10A AL121789.38	-0.10303	0.034442	-0.00262	0.101649	0.067071
PDE11A	-0.1721	-0.40766	-0.84061	-0.31712	-0.33776
PDE12	0.112224	0.11525	-0.16694	-0.01713	0.133548
PDE1A	-0.57441	0.529956	0.220842	-0.66756	-0.00229
PDE1B	0.035094	-0.12973	-0.11281	0.13195	-0.04667
PDE1C	-0.31923	0.101439	-0.37789	-0.00291	-0.12488
PDE2A	-0.267	-0.18405	-0.01625	-0.28836	-0.11481
PDE3A	-0.34788	-0.34881	-0.36375	-0.25206	-0.24443
PDE3B	0.295645	0.272725	0.27918	0.172106	0.187045
PDE4A	0.264375	0.069044	0.442441	0.08384	0.126431
PDE4C	0.193779	0.170554	0.515239	0.186465	0.282523
PDE4D	0.744817	0.928575	0.661249	0.110276	0.726306
PDE5A	-0.25208	-0.46225	-0.39784	-0.36257	-0.32093
PDE6A	0.021651	0.097901	-0.02857	-0.2174	-0.11347
PDE6B	0.219865	-0.13344	0.456469	0.325655	0.44661
PDE6C	0.390506	0.259326	0.477607	0.052396	-0.56218
PDE6D	-0.52478	-0.31299	-0.53314	-0.27138	-0.34636
PDE6G	0.204982	0.202316	0.08679	-0.13957	-0.00691
PDE6H	0.166512	0.078344	0.080496	-0.33884	0.052502
PDE7B	0.132504	0.250169	-0.02001	0.13326	-0.00538
PDE8A	0.225268	0.234621	0.206735	0.292674	0.35686
PDE8B	-0.05254	-0.162	-0.01868	-0.06864	0.083529
PDE9A	0.004233	-0.09304	0.013734	-0.02678	-0.0369
PDGFA	-0.15313	-0.11848	-0.11718	-0.10512	-0.08902
PDGFC	-0.14595	-0.19404	0.014484	-0.04334	-0.01025
PDGFRA	-0.53814	-1.18215	-1.17088	-1.02716	0.551244

PDGFRB	-0.28251	-0.35871	-0.36063	-0.3028	-0.35527
PDGFRL	0.066296	-0.11433	-0.11983	0.036733	-0.31311
PDHA1	0.207069	0.213697	0.064632	0.190386	0.176957
PDHA2	0.530995	0.713434	0.69528	0.324083	0.609694
PDHB	0.1872	0.263048	0.316664	0.150803	0.263775
PDIA4	-0.16932	-0.19469	-0.48711	-0.14308	-0.39239
PDIA5	-0.00145	-0.0232	-0.06483	-0.17748	-0.15293
PDIA6	-0.02846	0.140148	0.112919	0.064695	0.039226
PDIK1L	-0.00387	0.134153	0.135957	0.060808	0.154944
PDK1	0.11182	0.005054	-0.17219	-0.00488	0.055701
PDK2	-0.28498	-0.42185	-0.70192	-0.55644	-0.54012
PDK3	0.171966	0.118999	0.264856	0.031727	0.048738
PDK4	0.301132	0.283052	0.083748	0.313728	0.498098
PDLIM1	-0.01596	0.17435	0.316566	0.168574	0.102949
PDLIM2	0.13062	0.070835	0.181272	0.23823	0.254919
PDLIM3	0.104285	0.008995	-0.0608	-0.11641	-0.23545
PDLIM4	0.041599	0.159853	0.047722	0.096134	0.049705
PDLIM5	0.118429	0.063599	-0.09705	0.03031	-0.1063
PDLIM7	0.021032	-0.19412	0.229306	0.23363	-0.0469
PDPK1	-0.01535	-0.19677	-0.08801	0.063852	-0.07114
PDPK1 AC141586.2	-0.25497	-0.09032	-0.42252	-0.22123	-0.26629
PDPN	0.188131	0.775414	-0.22753	0.366418	0.484642
PDRG1	-0.08763	-0.1946	-0.14672	-0.08483	-0.12257
PDS5A	-0.03233	-0.14401	-0.28696	-0.24172	-0.10591
PDS5B	-0.18638	-0.17643	-0.23849	-0.29406	-0.29346
PDSS1	0.040527	0.276739	0.102556	0.057533	0.153849
PDSS2	-0.1744	-0.02817	0.015517	-0.11684	-0.13617
PDX1	-0.23781	0.230722	0.422236	0.364229	0.407729
PDXP	-0.16443	-0.04492	-0.14453	-0.21554	-0.2403
PDYN	-0.08492	0.220428	0.394085	-0.08466	0.036377
PDZD11 KIF4A	0.014484	-0.30604	-0.60592	-0.20784	-0.21308
PDZD3	0.390911	0.231637	0.195118	0.203947	0.259253
PDZD4	-0.04271	-0.02197	0.205969	0.06731	0.056663
PDZD7 SFXN3	-0.04732	-0.06419	-0.25228	-0.12594	-0.2202
PDZD8	0.040038	0.133446	0.098679	0.025619	0.146419
PDZK1IP1	0.163418	0.407539	0.266	-0.00759	0.255921
PDZRN3	-0.06368	0.094684	0.016434	-0.02096	0.093658
PDZRN4	-0.17408	0.044872	-0.42343	-0.41103	-0.87829
PEA15	-0.30317	-0.37609	-0.52543	-0.15329	-0.21871
PEBP1	0.208174	0.118603	0.133882	0.076976	0.273177
PECI	-0.3911	-0.3381	-0.54348	-0.21792	-0.11223
PECR TMEM169	-0.19511	-0.14157	-0.36977	-0.2676	-0.29947
PEF1	-0.04695	-0.03029	-0.25112	-0.22252	0.152541
PEG3	0.104671	0.418211	0.360427	0.126759	0.492752
PELI1	0.618014	0.451945	0.736583	0.079018	0.496865
PELI2	-0.26112	-0.23653	-0.02497	-0.15786	-0.24549
PELI3	-0.08681	0.057772	-0.10336	0.015441	0.09053
PELO	-0.26064	-0.51935	-0.80096	-0.63782	-0.4536
PELP1.	-0.14453	0.23932	-0.05542	-0.07422	0.017365
PENK	0.178644	0.06325	0.309498	0.511017	0.404515

PEPD	0.133966	0.353113	0.146161	0.147844	0.181258
PER1	-0.07625	-0.02309	-0.4	-0.30768	-0.27421
PER2	0.112295	0.000745	0.241524	0.022313	0.113901
PERLD1	-0.17081	-0.0296	-0.19799	0.166128	-0.46139
PERP	0.345671	0.035041	0.12553	0.4564	0.319802
PES1	-0.28113	-0.10334	-0.18571	-0.22588	-0.40109
PES1 TCN2	-0.26291	-0.245	0.344379	-0.27643	-0.30514
PET112L	0.087895	0.04246	-0.22559	-0.27793	-0.38767
PEX1 AC005156.1	-0.14553	-0.08579	-0.28957	-0.18587	-0.16486
PEX11A WDR93	0.188502	0.197823	0.272747	0.307485	0.334213
PEX11G	0.51288	0.443708	0.488637	0.575301	0.287653
PEX12	-1.04086	-0.85422	-1.05551	-0.80553	-0.976
PEX16	-0.29972	-0.30873	-0.3145	-0.19606	0.009052
PEX19	0.077083	-0.49663	-0.65003	-0.59447	-0.04778
PEX26	0.050769	-0.30368	-0.35758	-0.17799	-0.27544
PEX5	-0.19727	-0.0898	-0.20716	-0.21735	0.018755
PEX5L	0.070214	-0.01231	0.004422	0.152293	0.112225
PEX6	0.144208	-0.00647	0.006995	0.269316	-0.05308
PEX7	0.013892	0.161916	-0.08186	0.090509	-0.0854
PF4	0.276046	-0.1277	-0.45619	0.033001	-0.04164
PF4V1	0.174685	-0.42663	-0.34219	-0.25731	-0.16372
PFDN1	-0.70619	-0.52689	-0.81841	-0.40424	-0.77499
PFDN2 NIT1	-0.19905	-0.37242	-0.87034	-0.48565	-0.52771
PFDN4	0.127406	0.043556	0.125625	-0.03908	0.027287
PFDN5	0.229494	0.17919	0.054388	-0.01052	0.028763
PFDN5 C12orf10	-0.16017	-0.14641	-0.2794	-0.26449	-0.20084
PFKFB1	-0.1422	0.024298	-0.6741	-0.96038	0.129776
PFKFB3	-0.03051	-0.03669	-0.02028	0.024653	0.058564
PFKFB4	-0.27869	-0.08405	-0.3721	-0.08326	-0.15078
PFKL	0.095816	0.24188	0.379828	0.18797	0.246886
PFKM	-0.21128	-0.27116	-0.5388	-0.54176	-0.46933
PFKP	-0.06843	-0.01055	-0.2234	0.019118	-0.06853
PFN1 ENO3	-0.21175	-0.31584	-0.5973	-0.43619	-0.2471
PFN2	-0.20119	-0.25715	0.024814	-0.18103	-0.13268
PFN3	0.201211	0.574145	0.668763	0.340052	0.468646
PFN4 AC008073.6	-0.00516	-0.05506	-0.03558	-0.03803	0.109365
PFTK1	0.129884	0.375817	0.847828	-0.04875	-0.57637
PGA5	0.378608	0.296383	0.67218	0.142662	0.393798
PGAM1	0.042685	0.283454	0.070332	0.152468	0.237176
PGAM2	0.145021	0.23385	0.173434	0.120725	0.108719
PGAM5	-0.0674	-0.18596	-0.06329	0.112448	0.04737
PGAP1	-0.79121	-0.77113	-0.68817	-0.64734	-0.73011
PGBD1	-0.37418	-0.17933	-0.3794	-0.36075	-0.38554
PGBD2	-0.31967	-0.25613	-0.25568	-0.44502	-0.31166
PGBD5	0.001766	0.304975	0.386475	0.19324	0.356479
PGC	0.352828	0.5555	0.596291	0.302226	0.504487
PGD	-0.01627	0.139864	-0.05953	0.089253	0.092471
PGDS	0.352269	0.230348	0.472761	-0.05968	0.063663
PGF	0.164511	0.110617	0.401183	0.180755	0.238773
PGGT1B	0.283897	0.26032	-0.01347	0.072361	0.031441

PGK1	0.235407	-0.00131	0.208432	-0.11401	0.082997
PGK2	0.39223	-1.20181	-0.88128	0.22262	-0.59877
PGLS	0.028374	0.040369	-0.16377	-0.13454	0.067935
PGLYRP1	0.393472	0.484945	0.71162	0.391729	0.539665
PGLYRP2	0.066726	-0.03732	0.03728	-0.15863	0.308066
PGLYRP3	-0.07606	-1.08659	-1.27842	-1.02899	-0.79411
PGLYRP4	0.0031	-0.92679	-1.18346	-0.78016	-0.42415
PGM1	0.278485	0.521656	0.366102	0.238894	0.325801
PGM2	-0.2405	-0.18116	-0.31552	-0.13478	-0.36059
PGM2L1	-0.2243	0.065414	-0.23196	-0.17887	-0.16288
PGM3 RWDD2A	-0.0995	0.220559	-0.06793	0.06142	0.069556
PGM5	-0.4296	-0.28783	-0.14088	-0.2694	-0.11019
PGR	-0.22463	-0.40562	-0.48488	-0.30692	-0.64312
PGRMC1	-0.02914	0.02987	0.107786	0.079885	-0.11587
PGRMC2	-0.51253	-0.36254	-0.49869	-0.35651	-0.31929
PGS1	0.156424	-0.13389	-0.24254	-0.21167	0.044111
PH-4	0.022056	0.131567	0.710489	0.095302	0.197015
PH-4 WDR6	-0.07174	0.023535	-0.19931	-0.20606	0.063248
PHACTR2	0.439411	0.23034	0.653585	0.274135	-0.19431
PHACTR4	-0.43093	-0.2399	-0.3444	-0.21262	0.014984
PHB	-0.23962	-0.59702	-0.51276	-0.2215	-0.64521
PHB2 EMG1	-0.37738	-0.53954	-0.72009	-0.34175	-0.50073
PHC1	-0.62637	-0.40772	-0.76699	-0.5373	-0.47006
PHC3	-0.13517	-0.15686	-0.21278	-0.18173	-0.24551
PHCA	0.214081	0.275283	0.329956	0.273187	0.142776
PHEX	0.264904	0.713667	0.356335	0.321231	0.607072
PHF1	-0.26466	-0.40726	-0.41421	-0.27996	-0.374
PHF10	-0.14307	-0.07944	-0.28061	-0.21394	-0.22278
PHF11	0.235459	0.513307	0.293802	0.220503	0.24606
PHF12	-0.33366	-0.23983	-0.41553	-0.39193	-0.28283
PHF13	-0.21063	-0.04509	-0.25487	-0.13044	-0.05386
PHF14	0.2078	-0.09969	-0.77052	-0.45175	-0.52598
PHF15	-0.36718	-0.4648	-0.38027	-0.3548	-0.33038
PHF16	0.065477	0.051664	0.26531	0.111678	0.154477
PHF17	0.04059	0.055015	0.087756	-0.01361	0.049732
PHF19	0.133521	0.091828	0.175282	0.008962	-0.04599
PHF2	0.092414	-0.05546	0.160372	0.114721	0.173222
PHF20L1	0.040829	-0.11999	-0.08373	-0.52545	-0.07082
PHF21A	-0.4111	-0.2005	-0.42108	-0.32222	-0.25449
PHF21B	-0.12698	-0.06812	-0.18166	-0.15307	-0.01196
PHF3	-0.01425	-0.03287	-0.17337	-0.28054	-0.18988
PHF5A ACO2	-0.46595	-0.32748	-0.47017	-0.44277	-0.72208
PHF6	0.046057	-0.36005	-0.79016	-0.43092	-0.48328
PHF8	0.086871	-0.18243	0.254581	-0.25573	-0.46832
PHGDH	-0.16564	-0.46267	-0.55513	-0.45097	-0.5071
PHIP	-0.30422	-0.26644	-0.46212	-0.40519	-0.45984
PHKA1	0.252101	0.222446	0.369422	0.186206	0.129919
PHKA2	0.092825	0.095264	0.250269	0.065724	0.186083
PHKG1	0.418644	-0.12275	0.005133	0.306292	0.11226
PHKG2	0.079107	0.004342	-0.31197	-0.16427	-0.11085

PHLDA1	-0.11666	-0.1268	0.237963	0.134379	0.080431
PHLDA2	0.094349	0.053997	0.346327	0.26216	0.376437
PHLDA3	0.101643	0.23118	0.104798	0.090533	0.314822
PHLDB1	-0.01861	-0.01003	-0.06485	-0.06883	0.229195
PHLDB3	-0.01025	0.008046	-0.1223	-0.13661	-0.09498
PHLPP	-0.24807	-0.10296	-0.26459	-0.12798	-0.09244
PHLPPL	0.645311	0.962641	1.013946	0.464698	0.619065
PHOSPHO1	0.001224	-0.50002	-0.65758	-0.08672	-0.16079
PHOX2A	-0.12474	0.041207	-0.25733	-0.15356	-0.2577
PHOX2B	-1.65315	-1.95133	-1.30271	-1.60278	-0.00635
PHPT1	-0.37141	-0.5074	-0.12787	-0.28717	-0.43404
PHTF1	-0.18726	-0.06279	-0.16933	-0.2048	-0.09263
PHTF2	-0.26882	-0.17075	-0.60083	-0.35799	-0.37125
PHYH	0.252149	0.161971	0.309684	0.20978	0.272816
PHYHD1	0.368913	0.443083	0.442224	0.344373	0.377908
PHYHIP	-0.07484	-0.18185	-0.25048	-0.33397	-0.18871
PHYHIPL	0.011323	-0.12331	-0.0576	-0.13354	-0.01979
PI15	-0.65432	-1.10049	-0.95087	-0.61406	-0.97506
PI16	-0.01137	0.195997	0.203326	-0.0356	-0.06044
PI3	-0.30657	-0.71475	-0.67449	-0.51469	-0.71267
PI4K2A	0.035149	0.081672	0.293879	0.133097	0.138485
PI4K2B	-0.13841	0.126339	0.020843	-0.13655	-0.13635
PI4KA SNAP29	-0.11996	-0.11942	-0.07878	-0.07301	-0.07971
PI4KB	-0.29659	-0.53072	-0.59667	-0.95994	-0.6424
PIAS1	-0.03909	0.041479	0.281687	0.176974	0.233402
PIAS2	-0.23328	-0.15101	-0.0635	-0.14242	0.106013
PIAS3	-0.57463	-0.68542	-1.10584	-0.5906	-0.52872
PIAS4	0.069796	0.120203	0.179356	0.05192	0.125892
PIB5PA	-0.22833	-0.49207	-0.39754	-0.23819	-0.30142
PICALM	-0.30134	-0.3045	-0.29953	-0.22223	-0.3428
PICK1	0.105718	0.166128	0.053779	0.124951	0.171534
PID1	0.13683	0.099567	0.144434	0.203901	0.263387
PIGA	0.060399	-0.15559	0.307624	-0.27669	-0.38523
PIGB	-0.02672	-0.31774	-0.30778	-0.21997	-0.29707
PIGF CRIPT	-0.72367	-0.87199	-1.05439	-0.78241	-1.03161
PIGH	0.068925	0.299035	0.267233	0.027645	0.189546
PIGK	-0.55641	-0.5111	-0.60936	-0.83797	-0.29298
PIGM	-0.73109	-1.13211	-1.46853	-0.42718	-0.99846
PIGN KIAA1468	-0.30978	-0.3198	-0.46282	-0.36846	0.02219
PIGO	0.224944	1.263734	0.268323	0.177921	0.179481
PIGP TTC3	-0.07979	0.078809	-0.10804	-0.05571	0.141656
PIGQ	0.014801	-0.09045	0.013873	0.068182	0.029816
PIGR	0.589197	0.398607	0.635037	-0.36553	0.092581
PIGS	-0.29343	-0.24224	-0.59114	-0.53786	-0.21007
PIGT	-0.52437	-0.44756	-0.56364	-0.39858	-0.56108
PIGV	-0.24201	-0.28899	-0.13071	0.046724	0.088539
PIG-Y	-0.06919	0.17431	0.098599	0.053529	0.037926
PIGZ	-0.43745	-0.25365	-0.31968	-0.20561	-0.28683
PIH1D1 ALDH16A1	0.143951	-0.0931	-0.14685	-0.09895	-0.0605
PIH1D2 C11orf57	-0.55062	-0.24739	-0.56837	-0.36961	-0.61577

PIK3AP1	0.185616	0.431332	0.259616	0.288911	0.24055
PIK3C2A	-0.04035	-0.08013	-0.39075	-0.35822	-0.18033
PIK3C2B	0.395027	0.634385	0.431348	0.323352	0.459375
PIK3C2G	0.386873	0.218604	-0.196	0.057125	0.055893
PIK3C3	-0.17452	0.194359	-0.1054	-0.23419	-0.15092
PIK3CA	-0.35764	-0.21393	-0.21826	-0.23513	-0.13086
PIK3CB	0.327723	0.220847	0.22543	-0.15816	-0.15759
PIK3CD	-0.05278	0.119318	-0.01026	0.081636	0.21064
PIK3CG	0.542754	0.82617	0.814034	0.038777	0.453885
PIK3IP1	0.046153	0.432958	0.390037	0.126939	0.126426
PIK3R2	-0.05915	-0.122	-0.16607	-0.12375	-0.31217
PIK3R3	-0.55619	-0.30213	-0.39981	-0.38916	-0.15634
PIK3R4	-0.51217	-0.27046	-0.56656	-0.18498	-0.44736
PIK3R5	0.421875	-0.22719	-0.02241	0.081747	0.308938
PILRA	0.244089	0.250701	0.311305	0.096607	0.35335
PILRB	0.335965	0.489779	0.41674	0.204272	0.253947
PIM1	-0.39624	-0.24812	-0.47223	-0.37002	-0.27427
PIM2	0.00965	-0.22403	-0.14142	-0.13345	-0.22527
PIM3	0.223735	0.345076	0.30928	0.248838	0.305371
PIN1	0.117797	-0.02492	-0.18126	0.043298	-0.12668
PIN4	0.108762	-0.28012	-0.99494	-0.3945	-0.48297
PINK1	0.299512	0.274218	0.158463	0.131774	0.258399
PIP	-1.13026	-1.03359	-1.28365	-0.86645	-0.97276
PIP4K2C	0.142876	0.285343	0.28233	0.004953	0.153392
PIP5K1A	0.110704	-0.15057	-0.3728	-0.02832	-0.19111
PIP5K2A	0.106271	0.159219	0.193594	0.046217	0.26564
PIP5K2B	-0.13642	-0.11875	-0.32848	-0.24326	-0.25211
PIP5K3	-0.43834	-0.31869	-0.37552	-0.37686	-0.45149
PIP5KL1	0.096566	-0.1108	0.096625	-0.05664	-0.07881
PIPOX	0.22405	0.348526	0.46502	0.158419	0.402005
PIR BMX	0.42978	-0.21093	-0.25856	-0.02242	0.05998
PISD	0.099418	0.34244	0.241132	0.240206	0.263064
PITPNA	-0.08004	-0.0019	-0.0753	0.037739	0.053109
PITPNB	-0.26908	-0.26363	-0.34218	-0.2366	-0.26941
PITPNC1	-0.19569	-0.33692	-0.17583	-0.33882	-0.21491
PITPNM1	0.018885	-0.02898	-0.14193	0.032705	-0.09649
PITPNM3	-0.02823	0.038102	-0.00663	-0.13205	-0.06679
PITRM1	-0.39583	-0.17317	-0.50919	-0.16773	-0.41412
PITX1	-0.22442	-0.08059	0.130981	-0.1095	0.10102
PITX3	-0.06654	0.038485	-0.06178	0.116841	0.071726
PIWIL1	0.247186	0.331682	0.114402	0.162918	0.249302
PIWIL2	0.476473	0.699571	0.753497	0.477562	0.547646
PIWIL4	0.341309	0.634659	0.804681	0.463999	0.395649
PJA1	0.146178	0.116873	-0.03318	-0.14606	-0.01304
PKD1	-0.03119	0.025449	0.011194	-0.06547	-0.03928
PKD1L2.	0.529677	0.787218	0.999747	0.49319	0.41393
PKD2	-0.05391	0.234695	0.215334	0.130344	0.153132
PKD2L1	-0.13086	0.007765	0.1274	0.049835	-0.71039
PKD2L2	0.386855	0.591772	0.553135	0.530494	0.18423
PKDREJ	0.134445	0.351193	0.34007	0.229339	0.249545

PKHD1	0.025275	0.401011	0.314245	0.246848	-0.26636
PKHD1L1	0.497395	0.568549	0.436812	0.386834	0.282928
PKIA	-0.50913	-0.74227	-0.59335	-0.69465	-0.65498
PKIB	0.266021	-0.10157	-0.34027	-0.6875	-0.60006
PKIG	0.320758	0.549077	0.520395	0.190324	0.436252
PKLR	0.27567	0.528203	0.424277	0.22656	0.407111
PKM2	-0.61601	-0.57729	-0.48557	-0.47083	-0.48054
PKMYT1	0.093703	0.028666	0.013465	0.055764	-0.03919
PKN2	-0.39713	-0.19561	-0.17412	-0.33403	-0.08408
PKN3	0.146015	0.285244	0.162295	0.13265	0.228988
PKNOX1	-0.25677	-0.15694	-0.16161	-0.1596	-0.22532
PKNOX2	0.345631	0.464533	0.464352	0.316357	0.414593
PKP1	0.323148	0.682806	0.776562	0.424233	0.331559
PKP2	0.282666	0.458062	0.373057	0.399618	0.27338
PKP3	0.100897	0.380903	0.408407	0.204415	0.294769
PLA2G10	-0.02631	0.086328	0.356888	0.22788	-0.1007
PLA2G12A	-0.2948	-0.05446	0.046003	-0.12254	-0.36537
PLA2G12B	0.333336	0.571082	0.555878	0.190514	0.399581
PLA2G1B	0.419421	0.380488	0.597	0.21888	0.253186
PLA2G2A	-0.21522	-1.06059	-0.62895	-0.74606	-0.51594
PLA2G2D	0.288844	-0.31795	0.214865	-0.19783	0.112261
PLA2G2E	0.177528	0.035034	0.051593	0.007153	0.14856
PLA2G2F	0.398428	0.24467	0.452257	-0.0413	0.255574
PLA2G3	0.007179	0.187182	0.084411	0.035519	0.106091
PLA2G4A	0.063645	0.102429	-0.0591	-0.03458	-0.13593
PLA2G4B	-0.19996	0.25866	-0.16249	0.097271	-0.03732
PLA2G4C	0.369647	0.456338	0.428132	0.358246	0.308271
PLA2G4F	0.314745	0.334643	0.185638	0.089879	0.279127
PLA2G5	0.114355	0.315648	0.363481	0.0656	0.164471
PLA2G6	0.069972	0.16303	0.185565	-0.0187	-0.23673
PLA2G7	0.162076	0.277217	0.227651	0.22773	0.43501
PLA2R1	0.060057	0.577568	0.576515	0.345107	0.47968
PLAC1	-0.54706	-0.26265	-0.87183	-0.52976	-0.30774
PLAC1L	0.221737	-1.14187	-0.1434	-0.90992	-0.10554
PLAC8	0.224326	0.46652	0.490557	0.103094	0.218871
PLAC8L1	-0.2432	-0.24762	-0.48887	-0.59032	-0.5037
PLAC9	0.294939	9.69E-05	-0.0643	0.344798	0.257118
PLAG1 CHCHD7	-0.24566	-0.30244	-0.20468	-0.25214	-0.32972
PLAGL1	0.259438	0.487781	0.616097	0.281722	0.435006
PLAGL2 POFUT1	0.001119	-0.11052	0.058736	-0.0412	-0.04383
PLAT	0.110383	0.109915	0.086054	0.189428	-0.44801
PLAU	0.122612	0.475431	0.171787	-0.01413	0.339792
PLAUR	0.31924	-0.22517	0.347801	-0.09201	-0.2113
PLB1	0.410663	0.434215	0.640857	0.291202	0.449399
PLCB1	-0.22482	-0.31552	-0.08789	-0.12446	-0.2485
PLCB2	0.12099	0.091125	0.223619	-0.00833	-0.03959
PLCB3	-0.46212	-0.33947	-0.5584	-0.4633	-0.33077
PLCB4	-0.09523	0.0618	-0.01094	-0.00831	-0.0224
PLCD1	0.055761	0.2607	0.204185	0.351682	0.310282
PLCD3	-0.02523	0.113194	0.014444	-0.06449	0.115347

PLCD4	-0.39332	-0.59406	-1.01334	-0.42251	-1.15003
PLCE1	-0.29072	-0.47617	-0.62982	-0.49072	-0.89048
PLCG1	0.109987	0.108506	0.171993	0.135161	0.096256
PLCH1	0.244294	0.410977	0.691213	0.391892	0.361804
PLCH2	0.287174	0.359156	0.482037	0.240496	0.384901
PLCL2	0.23156	0.507856	0.365431	0.089646	0.396182
PLCXD1	0.154046	-0.02834	-0.10605	0.056114	0.025858
PLCXD3	-0.26123	-0.4519	-0.70519	0.445664	-0.5394
PLCZ1 CAPZA3	0.298109	0.465249	0.067504	-0.2021	-0.71576
PLD1	0.684172	0.715571	0.928479	0.476798	0.729686
PLD2	0.052137	-0.18981	-0.1376	-0.01901	0.193974
PLD3	0.507586	0.594871	0.424045	0.299713	0.427638
PLD4	0.208602	0.208721	0.169025	0.115777	0.202716
PLD5	-0.17119	-0.1884	-0.32456	-0.23071	-0.09035
PLDN	-0.82421	-0.72497	-0.80983	-0.67834	-0.81393
PLEK	0.577629	0.809494	0.85957	0.385717	0.552884
PLEK2	0.347842	0.574626	0.558374	0.509879	0.422078
PLEKHA2 HTRA4	0.229486	0.464105	0.633038	0.393403	0.491459
PLEKHA4	0.35866	0.454522	0.085906	0.339087	0.209183
PLEKHA5	-0.08577	-0.04562	-0.01932	-0.03995	-1.6E-05
PLEKHA6	-0.23359	-0.25197	-0.31876	-0.40753	-0.24321
PLEKHA7	-0.13341	0.160425	-0.00534	0.108411	0.152583
PLEKHA9 TMEM16F	-0.51342	-0.29143	-0.31732	-0.30067	-0.30342
PLEKHB1	0.537202	0.481791	0.667307	0.572373	0.521056
PLEKHC1	-0.17001	-0.15916	-0.16772	-0.09303	-0.14277
PLEKHF1	0.265935	0.416252	0.298357	0.274454	0.278184
PLEKHF2	-0.02393	0.171332	0.179523	0.010622	0.15498
PLEKHG1	0.118031	0.26792	-0.05078	0.139845	0.100837
PLEKHG2	-0.00075	-0.07731	0.013569	-0.03156	-0.00342
PLEKHG3	0.168213	0.114081	0.340171	0.098392	0.098638
PLEKHG4	-0.23616	-0.06789	-0.31155	-0.20545	-0.06823
PLEKHG4B	0.225135	0.023475	0.522721	0.358122	0.35898
PLEKHG6	0.159355	0.274555	0.331836	0.191841	0.265254
PLEKHG7	0.611216	0.443881	0.670211	0.229714	0.028353
PLEKHH1	0.147152	0.21053	0.331875	0.238398	0.197678
PLEKHH2	0.121403	0.154903	0.303349	0.224923	0.432836
PLEKHH3	-0.49924	-0.51847	-0.61254	-0.76507	-0.40164
PLEKHJ1 SF3A2	-0.00896	-0.0039	-0.14565	-0.01147	-0.11477
PLEKHK1	-0.36636	-0.05092	-0.21839	0.035646	-0.34152
PLEKHM1	-0.05633	-0.06282	-0.16379	-0.27705	-0.17126
PLEKHN1	0.24622	0.144428	0.334422	0.073958	0.199624
PLEKHO1	-0.17362	-0.35303	-0.40012	-0.31255	-0.15279
PLEKHQ1	-0.43282	-0.49036	-0.4609	-0.23944	-0.15255
PLG	0.481297	0.224914	0.220022	-0.12748	0.121956
PLGLB2	0.395719	0.138027	0.368437	0.154478	0.097275
PLIN	-0.21571	-0.14772	-0.50819	-0.41176	-0.53055
PLK1	0.008983	0.291874	0.292903	0.201743	0.397059
PLK2	-0.01198	0.077006	-0.03111	0.012116	0.082452
PLK3	0.100341	-0.08978	0.133815	0.09609	-0.00601
PLK4	-0.07408	-0.11912	-0.11965	-0.0858	-0.21091

PLLP	0.291176	0.265055	0.555595	0.112682	0.386387
PLOD1	0.002841	-0.04534	0.211705	0.104429	0.13195
PLOD2	0.008892	-0.08014	0.307168	0.115232	0.211519
PLOD3 ZNHIT1	-0.3478	-0.2461	-0.57675	-0.39779	-0.31629
PLP1	-0.08292	0.18181	-0.13661	0.251244	-0.02573
PLP2	0.358714	0.368128	0.283905	0.106047	0.38483
PLRG1	-0.60757	-0.8999	-0.82768	-0.81565	-0.9674
PLS1	-0.06909	-0.03821	-0.00087	-0.10129	0.060846
PLS3	-0.20522	-0.13032	-0.43427	0.050929	-0.24042
PLSCR1	0.02466	0.077469	0.10796	-0.07539	-0.03039
PLSCR2	-0.13741	-0.77968	-0.85238	-0.42733	-1.20136
PLSCR3	0.058947	0.156949	0.185888	0.117527	0.110065
PLSCR4	-0.05863	0.298178	0.11473	-0.23411	-0.02517
PLTP	-0.03541	-0.02376	-0.33841	-0.13171	-0.03066
PLUNC	0.009772	-0.65594	-0.55138	0.115717	-0.42843
PLVAP	0.419931	0.00691	0.532974	0.347504	0.365918
PLXDC1	-0.05481	-0.25694	0.000462	0.044936	0.040337
PLXDC2	-0.40367	-0.29151	-0.31597	-0.18289	-0.05134
PLXNA1	0.105249	0.160372	0.253698	0.180689	0.122606
PLXNA2	-0.28933	-0.44373	-0.37464	-0.28357	-0.25841
PLXNA3	0.020233	0.008594	0.298462	0.168923	0.151338
PLXNB1	0.262027	0.089815	0.356365	0.065215	0.142024
PLXNB3	0.307634	0.237246	0.409756	0.385564	0.352883
PLXNC1	0.039265	-0.02614	0.035104	0.045852	0.061491
PLXND1	0.112005	0.237854	0.36043	0.027907	0.156839
PM20D1	0.550336	0.946284	0.550535	0.742745	0.207865
PM20D2	0.211398	-0.18333	-0.14723	-0.08598	-0.14613
PMAIP1	-0.32447	-0.03152	-0.49905	-0.12082	0.041922
PMF1	0.349712	0.423432	0.449959	0.299628	0.20411
PMFBP1	-0.0791	0.001686	0.069969	-0.06571	-0.02392
PMM1	-0.00653	-0.27781	-0.25294	-0.06144	-0.04181
PMP2	0.284259	0.379165	0.128392	-0.0598	-0.163
PMPCB	-0.33648	-0.34845	-0.61413	-0.51162	-0.54382
PMS2 JTV1	-0.21674	-0.29207	-0.48705	-0.26648	-0.21928
PMS2L1.	0.696998	0.750108	0.805759	0.496964	0.737986
PMS2L1. PILRB	-0.53681	-0.31446	-1.2283	-0.59993	-0.50183
PMS2L3	0.060137	-0.18697	-0.483	-0.19626	-0.21646
PMS2L5 STAG3L2	-0.42416	-0.53309	-0.94693	-0.6924	-0.64762
PMVK	-0.30454	-0.34918	-0.66018	-0.40591	-0.3811
PNCK	0.232345	0.294082	0.335088	0.155798	0.263698
PNKP	-0.15523	-0.04694	-0.1687	-0.11123	0.013693
PNLDC1	0.412507	0.376085	0.641293	0.550956	0.621488
PNLIP	0.450299	0.462933	0.530591	0.411831	-0.06253
PNLIPRP1	0.032716	0.280262	0.298664	-0.09859	-0.78794
PNLIPRP2	0.556475	0.388078	0.916634	0.644797	0.357791
PNLIPRP3	4.99E-05	-0.74484	-0.00892	-0.35862	-0.88444
PNMA2	-0.17157	-0.03649	-0.20474	-0.24925	-0.0922
PNMA3	0.14071	0.215249	0.367714	0.139531	0.267526
PNMA5	-0.06271	-0.33195	-0.1485	-0.09354	0.007339
PNMA6A	0.236439	0.134766	0.45	0.347307	0.328226

PNMAL1	0.259946	0.546109	0.266421	0.130338	0.335318
PNMT	0.168427	-0.00765	0.06828	-0.03919	0.098651
PNN	-0.28956	-0.39822	-0.08151	-0.28181	-0.37316
PNOC	0.275792	0.3538	0.51431	0.090215	0.138774
PNPLA2	-0.0974	0.029749	0.111012	-0.01418	0.082725
PNPLA3	0.036682	0.205936	0.254545	0.070831	0.12891
PNPLA4	0.376948	0.452356	0.36534	0.478033	0.578894
PNPLA5	0.232512	0.515238	0.379617	0.293588	0.296381
PNPLA7 MRPL41	-0.17925	-0.38643	-0.4935	-0.25418	-0.33949
PNPLA8	-0.46529	-0.51814	-1.0778	-0.62521	-0.71578
PNPO	0.591168	0.021046	-0.03469	-0.13282	0.05918
PNPT1	0.017754	0.035805	0.015372	-0.18488	0.0354
PNRC1	-0.625	-0.56374	-0.55971	-0.47152	-0.44474
PNRC2	-0.39374	-0.54675	-0.56414	-0.23494	-0.31482
PODN	0.059224	0.169309	0.220677	0.14036	0.163889
PODNL1	-0.00081	0.008532	0.245966	0.157219	0.075158
PODXL	0.040575	0.063333	0.001093	0.046495	0.045593
PODXL2	0.109672	0.175788	0.095191	-0.35009	0.254989
PODXL2 ABTB1	-0.05973	0.006041	-0.06033	0.080663	0.083198
POF1B	-0.03257	0.211303	0.154188	-0.23465	-0.0695
POFUT2 BX322557.1	-0.30461	-0.31154	-0.1853	-0.07797	-0.30172
POGK	0.000985	-0.05849	-0.18422	-0.10541	-0.02278
POGZ	-0.07282	-0.17343	-0.13107	-0.26047	0.067583
POLA1	0.124324	-0.22541	0.303697	-0.19289	-0.2944
POLA2	0.045102	0.003001	-0.09161	-0.06201	0.074624
POLB	-0.17394	0.002376	-0.36259	-0.26242	-0.48216
POLD1	-0.09679	0.001789	-0.19415	-0.03613	-0.19806
POLD2	0.165314	0.195877	-0.02558	0.191229	0.165301
POLD3	0.198321	0.254994	0.154575	-0.03748	0.160017
POLD4	-0.21872	-0.42656	-0.28058	-0.52593	-0.65613
POLDIP2 C17orf32	-0.2607	-0.09247	-0.13597	-0.17569	-0.09479
POLDIP3 Z93241.11	0.001491	0.06194	0.127502	-0.17049	-0.23847
POLE PXMP2	0.122799	0.058425	0.117196	0.067607	0.155303
POLE2	0.32977	0.367026	0.553051	0.329916	0.442857
POLE3 C9orf43	-0.02574	0.098216	0.000972	-0.02572	0.016939
POLE4	0.397578	0.160135	0.383849	-0.17513	0.347554
POLG	-0.20167	-0.156	-0.24179	-0.20672	-0.39743
POLG2	-0.67983	-0.98257	-1.48472	-1.14929	-1.04888
POLI	0.125424	-0.02828	-0.15659	-0.1005	0.009863
POLL RP11-529I10.4	-0.21711	-0.45183	-0.4579	-0.07665	-0.27038
POLM	-0.19667	-0.13566	-0.36542	-0.12987	-0.21605
POLN C4orf15	0.451152	0.492286	0.533172	0.175967	0.386482
POLQ	-0.37397	-0.2808	-0.47306	-0.38468	-0.39082
POLR1A PTCD3	-0.90167	-0.72315	-1.0862	-0.79695	-0.62954
POLR1B	0.002305	-0.13019	-0.26596	-0.05083	0.032473
POLR1D	-0.56215	-0.47988	-0.46458	-0.38086	-0.43503
POLR1E	0.112983	0.161284	0.385341	0.050266	0.051726
POLR2C	-0.17758	-0.27412	-0.47841	-0.24695	-0.48057
POLR2D	0.1958	0.27388	0.162161	0.271129	0.128328
POLR2E	-0.04007	-0.31661	-0.39729	-0.24663	-0.44144

POLR2F	0.300536	0.302666	0.330718	0.112907	0.281606
POLR2G	-0.243	-0.16064	-0.2139	-0.11358	0.034963
POLR2I TBCB	-0.22049	-0.34033	-0.47871	-0.41975	-0.28412
POLR2J	0.019101	-0.07569	-0.38272	-0.16231	-0.0164
POLR2K	-0.30306	-0.29696	-0.53953	-0.48018	-0.36722
POLR3A	-0.0308	0.049006	-0.02914	-0.19723	-0.30569
POLR3C ZNF364	-0.08277	-0.6195	-0.37998	-0.25819	-0.52368
POLR3E	-0.11779	-0.25746	-0.21526	-0.20208	-0.31449
POLR3GL ANKRD34A	-0.57136	-0.68533	-0.93636	-0.74886	-0.65294
POLR3H	0.070552	0.255102	0.287611	0.000352	-0.05028
POLR3K C16orf33	0.187007	0.161871	0.059982	0.048152	0.002121
POLRMT	-0.15507	-0.07046	-0.04557	-0.21759	-0.18258
POLS	-0.21085	-0.18554	-0.24522	-0.18887	-0.30278
POM121	-0.24932	-0.16863	-0.44568	-0.23637	-0.29028
POM121L2	0.143456	0.080122	0.579946	0.543981	0.394542
POMC	-0.32616	0.190785	0.117624	0.12899	0.204139
POMP	-0.17998	-0.42557	-0.51619	-0.35473	-0.63193
POMT1	0.185604	0.025879	0.110773	0.09581	0.218728
POMT2 GSTZ1	-0.23093	-0.02208	-0.11621	-0.19412	-0.24768
POMZP3	-0.00414	-0.20506	-0.55645	-0.31768	-0.37701
PON1	0.258466	0.750264	0.771543	0.394369	0.445462
PON2	0.306035	0.441443	0.473319	0.542157	0.549886
PON3	0.314439	0.529548	0.460602	0.381083	0.446975
POP4	-0.74277	-0.80396	-0.94079	-0.71094	-0.54539
POP5	0.212433	-0.50974	-0.32318	-0.322	-0.53802
POP7	-0.17287	-0.11178	-0.51207	-0.17522	-0.23091
POPDC2	0.378544	-0.29465	-0.18941	0.063593	-0.06262
POPDC3	0.021223	0.028814	0.067141	-0.03807	0.026085
PORCN	0.317988	0.464512	0.358867	0.18239	0.267485
POSTN	-0.25477	-0.38271	-0.75198	-0.38031	-0.65129
POT1	-0.1672	-0.13112	-0.47806	-0.26578	-0.28818
POU1F1	0.514115	-0.07758	-0.39473	0.098817	-0.56742
POU2AF1	0.519173	0.562053	0.760621	0.535498	0.516739
POU2F1	-0.503	-0.80848	-0.63902	-0.4766	-0.45037
POU2F2	-0.39578	0.256312	0.410959	0.126352	0.322275
POU2F3	0.093285	0.44099	0.268619	0.231727	0.4241
POU3F1	0.080849	0.060526	0.210927	0.132474	0.259208
POU3F2	-0.60723	-0.40316	-0.57385	-0.4702	-0.52422
POU3F3	-0.182	-0.12831	0.037427	0.151432	0.207027
POU3F4	0.126618	-0.22557	0.655953	0.357374	0.578958
POU4F1	-0.07991	-0.08145	0.077127	0.049028	-0.04115
POU4F2	-0.43034	-0.34877	-0.22089	-0.3706	-0.13544
POU4F3	-0.36132	-0.43976	0.870586	0.227482	0.659418
POU6F1	-0.05452	-0.02128	-0.04357	0.019086	-0.02507
POU6F2	0.267814	-0.13631	-0.62494	-0.08944	-0.43388
PPA1	-0.05698	-0.10976	-0.30464	-0.37656	-0.30664
PPA2	0.165265	0.105085	0.286758	0.068637	0.190042
PPAP2A	0.043637	0.013445	-0.1407	-0.01493	-0.06609
PPAP2B	-0.51394	-0.35194	-0.60205	-0.65565	-0.26653
PPAP2C	0.116528	-0.00755	0.257806	0.18776	0.245825

PPAPDC1A	0.057853	0.147906	0.040145	0.182047	0.102511
PPAPDC1B.	-0.11851	-0.08355	-0.21678	-0.09248	-0.18176
PPAPDC3	0.506728	0.488403	0.734606	0.303654	0.520435
PPAPR5	-0.24688	-0.33958	-0.51242	-0.37572	-0.39346
PPARBP	0.081059	0.214143	-0.12542	0.15082	0.05764
PPARD	0.164026	-0.0126	-0.12053	-0.11096	-0.00823
PPARGC1A	-0.33768	-0.35777	-0.33928	-0.42526	-0.4576
PPARGC1B	0.056777	0.275645	0.117511	0.053727	0.106797
PPAT PAICS	-0.27401	-0.25716	-0.31138	-0.19164	-0.19445
PPBP	0.128196	-0.82011	-0.65322	-0.63155	-0.7315
PPCDC	-0.34246	-0.42488	-0.75892	-0.33548	-0.56141
PPEF1	0.211937	0.463279	0.4173	0.142208	0.501062
PPEF2	0.412803	0.321655	0.528675	0.162724	0.248706
PPFIA1	0.015644	0.017512	0.148993	0.137405	0.069577
PPFIA3	-0.24906	-0.15552	-0.22187	-0.19612	-0.18343
PPFIA4	0.36272	0.629324	0.695923	0.413034	0.430571
PPFIBP1	-0.46307	-0.41755	-0.67834	-0.3788	-0.48811
PPFIBP2	-0.1432	-0.25506	-0.21927	0.064965	-0.24751
PPIA	0.085229	0.088624	0.067789	0.095881	0.082793
PPIAL4	0.080383	0.370163	-0.2258	-0.38564	-0.27078
PPIB	0.041606	-0.09935	0.119095	0.114891	0.068769
PPIC	-0.11582	-0.13114	0.008355	0.019639	0.077221
PPIE	0.087056	0.233574	0.277831	0.310296	0.293611
PPIF	-0.01296	0.062802	-0.14257	-0.04308	-0.01466
PPIG	0.238402	0.484949	0.190424	0.092075	0.160459
PPIH	-0.34339	-0.07242	-0.504	-0.47148	-0.21786
PPIL1 C6orf89	0.137329	0.215545	-0.01698	-0.05647	0.132255
PPIL2	0.201711	0.262082	0.230139	0.322977	0.299336
PPIL3 NIF3L1	-0.88076	-0.98528	-1.10965	-0.94645	-1.10486
PPIL4	0.148634	0.071708	0.118749	0.007721	-0.09784
PPIL5	-0.15149	-0.17052	-0.52129	-0.03365	-0.2753
PPIL6 SMPD2	-0.15245	0.042743	-0.00851	0.005345	0.092493
PPL	0.16885	0.140582	0.362189	0.182983	0.182817
PPM1B	-0.76904	-0.65021	-0.86017	-0.62705	-0.68608
PPM1D	-0.24037	-0.54586	-0.328	-0.49209	-0.14426
PPM1E	0.048174	0.241703	0.077894	-0.08258	0.157006
PPM1F	0.227375	0.184002	0.087194	0.096855	0.13218
PPM1G	0.168467	0.1775	0.234163	0.021037	0.031617
PPM1J	-0.15512	-0.25561	-0.24165	-0.24919	-0.10668
PPM1K	-0.52986	-0.35148	-0.43081	-0.30103	-0.42846
PPM1L	-0.22186	0.218564	-0.0032	-0.03535	0.019743
PPM1M	0.047201	0.296986	0.240552	0.297599	0.246218
PPM2C	-0.24564	-0.13923	-0.25364	-0.2444	-0.1498
PPOX	-0.56461	-0.58481	-1.14356	-0.59985	-0.60145
PPP1CA	0.132654	0.13283	0.163483	0.194116	0.222963
PPP1CB	-0.03932	-0.10395	-0.29016	-0.17359	-0.24164
PPP1CC	-0.45409	-0.06416	-0.39356	-0.16941	-0.40997
PPP1R10 MRPS18B	-1.49197	-1.3263	-1.70571	-1.19982	-1.35518
PPP1R11	-1.36904	-1.09016	-1.41176	-1.00496	-1.24549
PPP1R12A	-0.41856	-0.31131	-0.13947	-0.20041	-0.22781

PPP1R12C	-0.19112	-0.14662	-0.12324	-0.09284	-0.09688
PPP1R13B	-0.07764	-0.19917	-0.23148	-0.03668	-0.0902
PPP1R13L CD3EAP	-0.46872	-0.52033	-0.5426	-0.12544	-0.21839
PPP1R14A	0.287739	0.377377	0.557476	0.238684	0.324376
PPP1R14B	0.108211	0.031408	0.230396	0.066673	0.220819
PPP1R14C	-0.0251	0.112261	0.263912	0.001311	0.192166
PPP1R14D	0.309798	0.401596	0.475286	0.260274	0.342122
PPP1R15A	0.04466	0.135162	-0.11359	0.205266	0.111963
PPP1R15B	-0.17434	-0.12832	-0.42942	-0.17314	-0.08578
PPP1R16B	-0.01863	0.185497	0.141272	0.150247	0.080231
PPP1R1A	0.006539	-0.01625	-0.06169	0.25973	0.408058
PPP1R1B	-0.12787	0.118346	0.140467	-0.09346	0.135679
PPP1R1B STARD3	-0.13487	-0.08318	-0.19229	-0.02658	-0.01511
PPP1R2P3.	0.11646	0.001501	-0.07729	0.196074	-0.11955
PPP1R3A	-0.08762	-0.87699	-1.13257	-0.70884	-0.85546
PPP1R3B	0.034566	-0.22216	-0.04983	-0.0778	-0.21739
PPP1R3C	0.304197	-0.224	0.064392	-0.23471	-0.29837
PPP1R3F	-0.10761	-0.55506	0.031475	-0.33812	-0.27923
PPP1R8	-0.02483	0.016849	-0.09931	0.013416	0.093517
PPP1R9B	0.034231	0.100107	0.092255	-0.00842	0.120271
PPP2CA	-0.13682	0.109567	-0.18485	0.144191	-0.12261
PPP2CB	-0.20685	-0.2288	-0.3019	-0.20152	-0.28412
PPP2R1A	-0.56936	-0.47	-0.53402	-0.62095	-0.52552
PPP2R1B	-0.39249	-0.12505	-0.59857	-0.37227	-0.59099
PPP2R2D	0.492576	0.807021	0.778269	0.458737	0.516348
PPP2R5A	0.022411	0.202185	0.032656	-0.01439	-0.06716
PPP2R5B	-0.19992	-0.17889	-0.12065	-0.20232	-0.13136
PPP2R5C	0.240274	0.364273	0.00432	-0.25036	-0.37944
PPP2R5D	0.259471	-0.02559	0.00204	0.129456	0.148502
PPP2R5E	-0.49102	-0.65608	-0.70751	-0.56309	-0.42243
PPP3CA	-0.38782	-0.43224	-0.47377	-0.23681	-0.40474
PPP3CB	-0.55153	-0.5866	-0.63681	-0.51467	-0.70538
PPP3CC	0.252247	0.447263	0.450933	0.268215	0.366142
PPP4C	0.065024	0.193237	0.126772	0.044781	0.063678
PPP4R1	-0.07528	0.012641	-0.13124	0.006112	-0.0631
PPP5C	-0.27109	-0.16682	-0.46505	-0.29616	-0.54096
PPP6C	-0.08312	0.079493	-0.09004	-0.1501	-0.12429
PPRC1	-0.27961	-0.01589	-0.13591	-0.08399	-0.24213
PPT1	0.238042	0.179202	0.166402	0.026236	0.136199
PPTC7	-0.04819	-0.06946	-0.17474	-0.12716	-0.24072
PPY	0.435796	0.375023	0.476353	0.496141	0.47362
PPYR1	0.295903	0.164164	0.210706	0.173789	0.040025
PQLC1	-0.13216	-0.13145	0.028242	-0.04032	0.127876
PQLC3	-0.09741	0.004065	0.075244	0.161588	0.021891
PRAF2 WDR45	0.13926	0.088562	-0.06803	0.00257	0.042402
PRAM1	0.442042	0.383856	0.493166	0.285766	0.337166
PRAME LL22NC03-63E9.3	0.497957	0.267697	0.101296	0.38945	0.472017
PRAMEF15	-0.19342	-1.23505	-1.13604	-0.74742	-0.69214
PRAMEF3	0.128648	-0.8069	-1.17391	-0.24043	-0.02215
PRAMEF5	-0.08666	-1.84253	-1.75285	-1.15378	-1.14975

PRAMEF8	-0.73832	-2.0922	-1.95438	-1.0713	-1.29821
PRAP1	0.078244	0.100599	0.173072	0.1015	0.075326
PRB3	-0.03029	-0.75161	-0.59658	-0.55136	-0.69938
PRC1	-0.1318	-0.09933	-0.14601	-0.10319	-0.26298
PRCC	-0.5196	-0.8432	-1.03966	-0.73474	-0.63301
PRCP	-1.04042	-0.60356	-1.13237	-0.97192	-0.92142
PRDM11	-0.19054	0.194992	0.189725	0.131665	0.180919
PRDM12	-0.23698	-0.07686	0.446935	0.266581	0.160032
PRDM13	-0.41049	0.260591	0.779392	0.508993	0.417415
PRDM14	-0.20871	0.181115	0.599502	0.269824	0.517094
PRDM15	0.031365	0.002408	0.02342	0.006957	-0.2036
PRDM16	-0.11936	-0.00665	0.309275	0.120271	0.124234
PRDM4	-0.2134	-0.30223	-0.16406	-0.23788	-0.22783
PRDM5	0.279987	0.3129	0.250079	0.257745	0.477858
PRDM7.	0.019703	-0.53244	-0.32451	-0.0144	-0.68354
PRDM9	0.010249	-0.95381	-0.80505	0.173894	-0.33702
PRDX1	-0.50384	-0.35194	-0.34511	-0.49622	-0.27464
PRDX2	0.176496	0.277106	0.229031	0.226169	0.198754
PRDX3	-0.07537	-0.05201	-0.27773	-0.28429	-0.31732
PRDX4	0.354481	0.244119	0.486637	0.082994	0.233517
PRDX6	-0.34657	-0.38037	-0.36027	-0.34943	-0.22494
PREB	0.209721	0.152743	0.361365	0.087399	0.220734
PREI3	-0.22381	-0.28754	-0.5382	-0.45974	-0.57917
PRELID2	0.342839	0.368025	0.441808	0.431388	0.306506
PRELP	-0.36912	-0.2447	-0.57331	-0.33909	-0.22862
PREP	0.143223	-0.20332	0.150872	-0.07424	0.022506
PRF1	0.233548	0.333681	0.539673	0.225734	0.296712
PRG2	-0.13908	-0.26899	-0.90892	-0.48856	-0.12621
PRG3	0.08889	0.032089	-0.30961	0.108628	0.091393
PRG4	0.35579	-0.33469	0.256898	0.011089	-0.36974
PRH1	0.440396	0.168923	0.112792	-0.07131	0.101961
PRICKLE1	-0.02348	-0.03167	-0.16302	-0.10852	-0.12001
PRICKLE2	-0.43367	0.468352	0.285252	0.445379	-0.11394
PRICKLE3	0.435391	0.644396	0.689108	0.405923	0.637385
PRIM1	-0.00358	0.042278	0.00229	0.059398	0.03334
PRIM2	-0.60087	-0.82444	-0.65063	-0.55305	-0.81681
PRIMA1	-0.05049	0.34532	0.39508	0.358162	0.311021
PRKAA1	-0.339	-0.52743	-0.51649	-0.24782	-0.37172
PRKAA2	-0.24525	-0.0132	-0.74835	-0.49908	-0.15427
PRKAB1	-0.059	-0.26054	-0.20238	-0.19694	-0.27538
PRKAB2	-0.04519	0.05447	0.042979	0.031325	0.019303
PRKACG	0.378231	0.410462	0.638502	0.362217	0.533765
PRKAG1 MLL2	0.092808	-0.63594	-0.58269	-0.62138	-0.7893
PRKAG2	-0.42893	-0.33867	-0.63028	-0.3965	-0.3864
PRKAG3	0.211485	0.191408	0.478318	0.172803	0.150523
PRKAR1A	-0.43518	-0.38369	-0.46852	-0.60257	-0.26715
PRKAR2A	-0.07144	-0.13869	-0.5099	-0.25049	0.033298
PRKAR2B	0.08149	0.249594	-0.07832	0.061249	0.098033
PRKCA	-0.14367	-0.30841	-0.15126	-0.28188	-0.10699
PRKCB1	-0.09983	-0.07908	0.078701	0.164651	0.090077

PRKCD	-0.09081	0.002247	-0.11166	-0.06116	-0.02303
PRKCDBP	0.184939	-0.09476	0.306387	0.311267	0.184685
PRKCE	0.108105	-0.04116	0.035204	0.037919	0.031344
PRKCG	-0.50368	-0.5198	-0.36158	-0.5267	-0.54093
PRKCH	0.168044	0.119665	0.391291	0.309785	0.485947
PRKCI	-0.3398	-0.19217	-0.37125	-0.33603	-0.2288
PRKCQ	0.08268	0.142448	-0.20661	0.142094	-0.01744
PRKD1	-0.06663	-0.0007	0.199299	0.141376	-0.0773
PRKD2	-0.54402	-0.80251	-0.57929	-0.30303	-0.45785
PRKDC MCM4	-0.2354	-0.16001	0.026685	-0.07757	-0.03296
PRKG2	0.028332	0.170539	-0.41649	-0.02218	-0.20447
PRKRA	0.030099	0.152835	0.217676	0.096377	0.216969
PRKRIR AP002360.4	-0.03958	0.115853	0.073182	0.12314	-0.04949
PRKX	0.217305	0.231475	0.238152	0.202578	0.321641
PRKY	0.277329	0.241626	0.374871	0.031971	0.306808
PRL	-0.09191	-0.13995	-0.17281	-0.30815	-0.23117
PRLH	-0.05427	0.089759	-0.07651	0.042845	0.01345
PRLHR	0.111884	0.071307	0.323646	0.292123	0.509683
PRLR	0.581504	-0.95173	0.661765	0.445997	0.087741
PRM1	0.408138	0.378932	0.655432	0.417618	0.407121
PRM2	0.186014	0.261109	0.373988	0.277293	0.401357
PRM3 AC009121.8	0.25562	0.383556	0.315341	0.183747	0.176688
PRMT1	-0.72473	-0.64622	-0.9959	-0.46897	-0.84714
PRMT2	-0.06482	0.045846	-0.17968	-0.14523	-0.15832
PRMT3	0.057624	0.111483	-0.0585	0.001547	0.07431
PRMT5	-0.88193	-0.83405	-1.03699	-0.56133	-0.94339
PRMT6	0.182733	0.275661	0.319551	0.120112	0.274304
PRMT8	-0.45137	-0.3338	-0.35838	-0.36482	0.13583
PRND	0.483852	-0.27935	0.265025	-0.0022	-0.01354
PRNP	0.110505	0.245138	0.277009	0.09675	0.223911
PRNPIP	0.222049	0.190374	0.151379	0.147489	0.24143
PRNT	0.107221	-0.29815	0.116322	0.05918	-0.01913
PROC	0.379625	0.555147	0.495867	0.292555	0.43997
PROCR	-0.05993	0.222519	0.497739	-0.24035	-0.25759
PRODH	0.221605	0.202433	0.314419	-0.09315	0.138393
PROK1	0.42331	-0.12012	0.462256	-0.64962	0.154027
PROK2	-0.05428	0.061417	0.082701	0.13356	0.260594
PROKR1	-0.08807	0.129974	0.031713	-0.0146	0.003538
PROKR2	0.167254	0.411417	0.313012	0.227739	0.165426
PROL1	-0.32618	-0.29991	-0.34511	-0.38368	-0.55627
PROM1	0.313597	0.495937	0.280401	0.185951	0.156208
PROM2	0.266984	0.25803	0.507371	0.290577	0.131733
PROP1	-0.11597	-1.22111	-0.98155	-0.57888	-1.08075
PROS1	-0.20142	-0.18695	-0.24919	-0.22495	-0.21007
PROSC	0.133113	0.252826	0.415846	0.072498	0.086615
PROX1	-1.34412	-1.44944	-1.59638	-1.23065	-1.28446
PROZ	0.292179	0.217138	0.552635	0.392406	0.246065
PRPF18	-0.67825	-0.47674	-0.75414	-0.55208	-0.90117
PRPF19	-0.06223	-0.36363	-0.25229	-0.38967	-0.16293
PRPF3	0.026959	-0.25093	-0.14197	-0.26518	-0.10278

PRPF38B	-0.61836	-0.49124	-0.44484	-0.62472	-0.66721
PRPF39	-0.24651	-0.05915	-0.05596	-0.07516	-0.26829
PRPF8	0.231537	0.259372	-0.0277	0.029948	0.282312
PRPH	0.05513	-0.16304	0.201761	0.150052	-0.09574
PRPH2	0.419219	0.76465	0.59561	0.370977	0.387527
PRPS1	0.06697	-0.24062	-0.1074	-0.12842	-0.24388
PRPS2	0.088328	-0.16122	-0.02514	-0.1404	-0.04996
PRPSAP1	-0.11595	-0.1468	-0.31288	-0.20156	-0.13136
PRPSAP2	0.019098	0.0427	0.00503	-0.10052	0.270269
PRR10	-0.07844	0.370281	0.071508	0.013127	-0.3281
PRR13	0.231567	0.059713	0.090384	0.045619	-0.16408
PRR14	-0.21616	0.194625	0.103346	0.114793	0.166096
PRR15	0.062431	0.403192	0.386443	0.172579	0.228021
PRR16	-0.29158	-0.32057	-0.38347	-0.27446	-0.2397
PRR18	0.157185	0.150432	0.125376	0.163683	0.220058
PRR4	0.332673	0.256804	0.293717	-0.24489	0.073027
PRR6	0.153742	0.455171	0.489229	0.245491	0.351433
PRR7	-0.07731	0.022259	-0.03098	0.012226	0.095436
PRRC1	0.204306	-0.06194	-0.17317	-0.14242	-0.13217
PRRG2	0.162858	0.094869	-0.13992	-0.21296	0.302999
PRRG3	0.012007	-0.54714	-1.11706	0.216246	0.0125
PRRG4	0.00238	0.069955	0.55334	0.379867	0.250039
PRRT1 EGFL8	-0.56846	0.203749	-0.68277	-0.2212	-0.63217
PRRT2	-0.33559	-0.46007	-0.47898	-0.31925	-0.46478
PRRT2 C16orf53	-0.21332	-0.11412	-0.39343	-0.23608	-0.20358
PRRX1	-0.46624	-0.11975	0.056373	0.272037	-0.11621
PRRX2	-0.01999	0.125113	0.120475	0.031836	0.255978
PRSS1	-0.26707	-0.54728	-1.21674	-0.0941	-1.22867
PRSS12	0.029854	-0.37111	-0.19261	-0.14375	-0.05047
PRSS16	0.387659	-1.60997	-0.37553	-0.75861	-0.09085
PRSS2	-0.29566	-0.38888	-1.1525	-0.20114	-1.0161
PRSS21	0.386322	0.233041	0.389112	0.283054	0.437223
PRSS22	-0.08847	-0.38244	0.063088	-0.10701	-0.33408
PRSS23	0.146035	0.398302	0.370404	0.248539	0.466167
PRSS27	0.040357	0.233878	0.156913	0.173593	0.089183
PRSS3	0.126313	0.098452	-0.41116	-0.11788	-0.3446
PRSS33	-0.26292	-1.01391	-0.37291	-0.45944	-0.49883
PRSS35	-0.09773	-0.19632	-0.09845	-0.19666	-0.15359
PRSS36	0.16729	0.010836	0.371627	-0.16647	0.083811
PRSS7	-0.43781	-0.42803	-0.63927	-0.5808	-0.69211
PRTFDC1	0.165683	-0.05935	0.335997	-0.2824	0.056992
PRTG	-0.1107	0.015225	-0.06744	-0.021	0.00036
PRTN3	0.264666	0.13239	0.517387	0.238755	0.151275
PRUNE2	-0.08125	-0.3279	-0.076	-0.2907	-0.22542
PRX	0.211312	0.446783	0.47061	0.202598	0.465409
PRY	0.04945	0.174182	0.315019	-0.02073	0.160947
PSAP	0.038493	0.041583	0.160171	0.147985	0.055243
PSAT1	-0.01782	0.1007	-0.17274	0.109176	-0.27609
PSCA	0.286831	0.433237	0.074759	0.245059	-0.03493
PSCD1	-0.03212	-0.15495	-0.20836	-0.22076	-0.08914

PSCD2	-0.11643	-0.09088	-0.05013	-0.03329	-0.18829
PSCD3	-0.04748	-0.25667	-0.14442	-0.01538	-0.04994
PSCD4	0.537013	0.704097	0.712816	0.326114	0.517459
PSCDBP	0.655573	0.662047	0.219332	0.441909	0.485733
PSD FBXL15	-0.45885	-0.6755	-0.80656	-0.57578	-0.68189
PSD2	-0.02533	0.025789	0.065898	0.045621	0.038081
PSD4	0.478788	0.411427	0.260954	-0.05535	0.254396
PSEN1	-0.02808	0.053238	0.156995	-0.03002	-0.05955
PSEN2	-0.00943	0.297834	-0.09111	0.088542	0.012499
PSG9	0.099195	-1.22125	-1.12953	-0.54411	-0.32451
PSIP1	-0.41184	-0.14872	-0.5535	-0.50231	-0.41446
PSKH1	-0.1807	-0.20559	-0.35907	-0.32812	-0.40227
PSKH2	0.49691	0.112709	0.624962	0.26556	0.479551
PSMA1	-0.22033	-0.09557	-0.50633	-0.26679	-0.06303
PSMA2 MRPL32	-0.71603	-0.9127	-1.02999	-0.68007	-0.62195
PSMA3	-1.26262	-0.98262	-1.2676	-1.00866	-1.21056
PSMA4	0.033759	-0.29744	-0.0497	-0.20777	-0.26383
PSMA5	-0.67707	-0.68258	-0.60847	-0.73489	-0.72925
PSMA7 SS18L1	0.021163	0.081699	0.127264	0.131124	0.03642
PSMA8	0.551342	0.83561	0.699984	0.603851	0.682696
PSMB1 TBP	-0.65802	-0.60137	-0.99089	-0.79887	-1.0337
PSMB10	-0.35419	-0.2696	-0.61705	-0.28212	-0.4152
PSMB2	-0.12653	-0.54159	-0.21258	-0.45552	-0.11482
PSMB3	-0.80095	-0.74031	-1.26427	-0.60473	-1.07154
PSMB4	0.000597	-0.13505	-0.38109	-0.08662	-0.05492
PSMB5	0.087014	0.174545	0.000911	-0.18614	-0.12525
PSMB6	-0.09051	0.066143	-0.07063	-0.25633	-0.13136
PSMB7	0.146016	0.52222	0.254971	0.164372	0.245608
PSMB8 PSMB9	0.165523	0.21038	0.38144	0.460632	0.335938
PSMB9	-0.2334	-0.08533	-0.10777	0.409157	0.073482
PSMC1	-0.03052	0.037225	-0.14287	-0.19546	-0.22126
PSMC2	-0.47402	-0.30364	-0.85835	-0.65219	-0.64671
PSMC3	-0.58412	-0.43818	-1.14936	-0.57238	-0.5295
PSMC3IP	-0.37489	-0.63848	-0.56096	-0.70816	-0.54439
PSMC4	-0.12815	-0.24116	-0.44134	-0.31596	-0.30195
PSMC6	-0.22106	-0.07694	-0.4041	-0.13204	-0.36685
PSMD10 ATG4A	0.038341	-0.36995	0.05699	-0.37843	-0.38214
PSMD11	0.005683	-0.18181	-0.37668	-0.13263	-0.16486
PSMD12	0.347684	0.438596	0.320922	0.128092	0.243964
PSMD2	0.06047	0.146047	-0.16284	-0.00133	-0.05354
PSMD3	-0.78757	-0.68439	-0.74878	-0.68588	-0.64815
PSMD4	-0.09669	0.23868	0.259841	0.371116	0.006323
PSMD5	0.164703	0.066229	0.249286	0.48538	0.233507
PSMD6	0.451783	0.203921	0.158281	-0.02405	0.156843
PSMD7	-0.5281	-0.3879	-0.58984	-0.10888	-0.43868
PSMD8	-0.11072	-0.13681	-0.15678	-0.24283	-0.18724
PSMD9	0.340135	0.018397	0.051328	-0.23858	-0.13135
PSME1	0.053603	-0.11028	-0.2127	0.010788	0.183798
PSME2 RNF31	-0.5226	-0.48447	-0.53774	-0.41254	-0.31907
PSME3	-0.61347	-0.93342	-0.98091	-1.09035	-0.767

PSME4	0.112409	0.239276	0.396219	0.193736	0.314893
PSMG3	0.172407	0.21791	0.042741	0.131806	0.111739
PSORS1C1	0.199757	-0.08716	-0.17788	-0.41695	0.039254
PSPC1	0.106491	0.049674	0.184049	0.046871	0.031738
PSPH CCT6A	0.060517	-0.06268	0.053676	0.147862	0.191118
PSPN	0.096254	0.278655	0.30312	0.38184	0.322094
PSTK	0.427064	0.32638	0.237153	0.085003	0.208988
PSTPIP1	0.099074	0.287578	0.145878	0.109135	0.103757
PSTPIP2	0.150287	0.229282	0.191068	0.16325	0.314505
PTAFR	0.408211	0.596912	0.706942	0.554344	0.311348
PTBP1	0.015227	0.022454	0.048439	-0.01551	0.051324
PTBP2	-0.13581	0.045893	-0.10197	-0.26082	-0.06885
PTCD1 CPSF4.	-0.2521	-0.40394	-0.67566	-0.13627	-0.4323
PTCH1	-0.21204	-0.12955	0.005012	-0.05612	-0.04325
PTCH2	0.030701	0.162932	0.125626	0.113855	0.244837
PTCHD1	-0.0147	0.047892	0.210006	-0.04399	-0.12978
PTCHD3	0.331652	0.397452	0.347759	0.368554	0.47137
PTDSS2	0.072142	0.269898	0.149705	0.197008	0.36294
PTEN	-0.35977	-0.23119	-0.32214	-0.13627	-0.16333
PTER	0.172876	0.753229	0.790066	0.551797	0.521234
PTF1A	-0.00861	-0.09343	0.123036	0.146526	0.29487
PTGDR	0.286194	0.631738	0.78525	0.559452	0.589581
PTGER1	0.055267	0.004062	0.100318	0.073089	-0.03981
PTGER2	0.220125	-0.23449	-0.34315	0.293074	-0.29203
PTGER3	-0.03836	0.469214	0.771951	0.128021	0.373234
PTGER4	-0.44926	-0.12278	-0.13942	0.039983	-0.04402
PTGES	0.373059	1.046583	0.354684	0.163943	0.072026
PTGES2	0.082962	0.070521	0.011825	-0.07639	0.007184
PTGES3	-0.30817	0.018307	-0.07981	-0.26169	-0.07113
PTGFR	-0.00377	-0.02032	-0.19082	-0.10816	-0.2208
PTGFRN	-0.08484	0.374865	0.025092	0.142146	0.389462
PTGIR	0.082777	-0.42341	0.370332	0.473128	0.214142
PTGIS	-0.20669	-0.10421	-0.08039	-0.01304	-0.08759
PTGS1	0.229492	0.16657	0.473318	0.13998	-0.02722
PTGS2	-0.09533	-0.83261	-0.47648	0.094281	-0.00786
PTH	-0.33337	-0.44523	-0.34986	-0.41877	-0.08749
PTH2	-0.29519	-0.83169	0.375913	-0.03356	-0.08741
PTHLH	-0.09306	0.169608	0.575953	-0.1564	-0.03529
PTHR2	0.236331	0.512186	0.363779	-0.06542	0.177084
PTK2	-0.1993	-0.35052	-0.10683	-0.31615	-0.17141
PTK2B	0.228325	0.622223	0.44175	0.352269	0.488097
PTK6	0.161345	0.224436	0.184372	0.209552	0.110564
PTK7	-0.07133	0.045442	0.035275	0.009431	0.031344
PTMS	-0.19676	-0.22816	-0.20867	-0.27047	0.011899
PTN	-0.18856	0.019118	-0.1253	0.112603	-0.27646
PTOV1	0.039126	0.036086	-0.14057	-0.01979	-0.01217
PTP4A1	-0.02669	0.350783	0.634309	0.186595	0.246087
PTP4A2	0.074285	0.107517	0.106879	0.106801	0.129106
PTP4A3	0.323567	0.673812	0.747177	0.494354	0.494129
PTPLA	0.276029	0.271051	0.153622	0.14086	0.197879

PTPLAD1	0.351345	0.420654	0.280662	0.230597	0.335361
PTPLAD2	0.343685	0.480029	0.685682	0.432658	0.643116
PTPLB	-0.50819	-0.40727	-0.6174	-0.39395	-0.58241
PTPN1	-0.17875	-0.10202	-0.23733	-0.19847	-0.07597
PTPN11	0.041337	-0.01768	0.136895	0.053073	0.089248
PTPN12	-0.43081	-0.49594	-0.72495	-0.3175	-0.51439
PTPN13	-0.13183	-0.06858	-0.17724	-0.00341	-0.03847
PTPN14	-0.09491	0.200585	0.204575	0.20994	0.172939
PTPN18	0.160807	0.115535	0.190467	0.096522	0.342339
PTPN2	0.111958	0.1765	0.154759	0.077624	0.12557
PTPN20B	0.3308	0.58609	0.531276	0.514527	0.444559
PTPN22 HIPK1	0.057205	0.441451	0.475826	0.040502	0.36439
PTPN23	-0.07666	-0.11279	-0.18917	-0.18395	0.033634
PTPN3	0.741022	1.150733	1.094837	0.56646	0.458667
PTPN4	-0.04252	0.096825	-0.01309	-0.16905	-0.00566
PTPN5	-0.10526	-0.06377	0.209182	0.018057	-0.02487
PTPN6	0.405779	0.636414	0.697093	0.390734	0.72494
PTPN7	0.350878	0.36786	0.411002	0.341472	0.315765
PTPN9	0.085884	0.322868	0.053224	0.049843	0.228931
PTPRB	-0.1051	0.217584	-0.02167	0.458967	0.173709
PTPRC	0.007634	-0.82244	-0.41571	-0.61901	-0.3516
PTPRCAP CORO1B	0.197704	0.491299	0.270771	0.287976	0.324497
PTPRF	-0.03741	-0.20003	-0.05048	-0.27404	-0.27748
PTPRH	0.22686	0.20331	0.51529	0.085438	0.383475
PTPRJ	0.199803	0.305523	0.500559	0.129115	0.301047
PTPRK	-0.28836	-0.21619	-0.04721	-0.19902	-0.17626
PTPRM	-0.02402	-0.03961	0.084344	-0.02972	0.174147
PTPRN	0.030448	-0.27406	-0.28476	-0.3051	-0.13715
PTPRN2	-0.23629	-0.21646	-0.29193	-0.17282	-0.12564
PTPRT	-0.20381	0.166728	0.078339	0.098152	0.175123
PTPRU	0.05036	0.165163	0.242393	-0.17154	0.011651
PTPRZ1	-0.19135	0.149207	-0.01748	0.199058	0.105574
PTRF	-0.08743	0.178886	0.207918	0.319925	0.225708
PTRH2 TMEM49	-0.8408	-1.1146	-1.41257	-1.20059	-0.75948
PTS	-0.26114	-0.16815	-0.44985	-0.15416	-0.46485
PTTG1IP	0.063039	0.087208	0.109308	0.091742	0.04744
PUF60	0.312286	0.334355	0.171851	-0.17256	0.448896
PUM1	-0.04188	0.23727	0.111671	-0.05357	0.114754
PUM2	0.09598	0.305139	0.148212	-0.02699	-0.06258
PUNC	-0.06387	-0.05079	-0.2728	-0.03002	-0.2641
PURA	-0.29379	-0.29857	-0.24516	-0.08221	-0.23225
PURB	-0.26205	-0.38209	-0.57343	-0.31269	-0.43223
PURG WRN	-0.36939	-0.35644	-0.49741	-0.42467	-0.38788
PUS1	-0.24886	-0.35677	-0.2805	-0.20681	-0.29514
PUS10 PEX13	-0.92474	-0.92988	-0.96813	-0.80367	-0.50155
PUS3 DDX25	-0.18928	0.060407	-0.20443	-0.17298	-0.27653
PUS7	0.172332	0.368395	0.283423	0.14547	0.199354
PUS7L IRAK4	-0.07244	-0.31481	-0.46202	-0.33691	-0.4596
PUSL1	-0.02023	-0.01508	0.027124	0.074056	0.123609
PVALB	0.412828	0.266924	0.506342	0.12317	0.38038

PVR	-0.00901	-0.12196	-0.03076	-0.04792	0.007324
PVRL1	-0.05448	0.170416	0.195905	0.074529	0.029235
PVRL2	-0.55629	-0.71815	-0.74939	0.014478	-0.59498
PVRL3	-0.17389	-0.20042	-0.22045	-0.17496	-0.05111
PVRL4	0.152794	0.323328	0.36635	0.247097	0.410121
PWP1	-0.35925	-0.22825	-0.36039	-0.16396	-0.09433
PWP2H	0.001955	-0.02402	-0.06502	0.161353	-0.04667
PXK	-0.04497	0.014535	-0.32745	-0.1812	-0.01444
PXMP3	-0.41969	-0.241	-0.47519	-0.3931	-0.42929
PXMP4	0.499971	0.088551	-0.15901	-0.03242	-0.4024
PXN.	0.033324	-0.08178	0.053594	0.081169	0.063535
PYCR1	0.047564	0.198341	0.180587	0.124273	0.140071
PYCR2	0.085107	0.110043	0.081428	-0.04843	0.043667
PYCRL	0.052113	-0.16274	-0.29539	-0.26411	0.059519
PYGB	-0.01165	0.024957	0.148434	0.097982	0.002418
PYGL	0.172153	0.140535	0.290157	0.181575	0.328296
PYGM	0.47351	0.424511	0.591086	0.252609	0.388357
PYGO1	0.230337	0.268769	0.114024	-0.31929	0.312903
PYGO2 SHC1	-0.02152	-0.03389	-0.08562	-0.00534	0.11189
PYHIN1	-0.15495	-1.38829	-1.06459	-0.84209	-0.72863
PYROXD1	0.113356	-0.23932	-0.26498	-0.11475	-0.24297
PYY NAGS	0.04335	-0.19352	0.232463	0.285666	-0.16
PYY2.	0.593006	0.295842	0.353399	0.507271	0.662742
PZP	-0.28546	-1.34192	-0.01647	-1.12641	-1.04762
QDPR	-0.21684	-0.0699	-0.48108	-0.30741	-0.64259
QKI	-0.22945	-0.07503	0.004837	-0.14445	-0.13692
QPCT	0.226288	0.360766	0.499787	0.29957	0.323124
QPRT	-0.13955	0.02849	-0.0764	-0.22528	-0.48969
QRFP	0.421696	0.65136	0.613155	0.561095	0.668749
QRICH1 CCDC36	0.09003	0.275108	0.072344	0.092117	0.272612
QRICH2	0.328272	0.675098	0.545726	0.444985	0.480868
QSER1	0.333106	0.472877	0.317571	-0.05738	0.251456
QSOX1	0.041331	-0.14735	-0.3995	-0.30106	0.138477
QSOX2	0.163887	-0.21296	0.042689	-0.18501	0.113769
QTRT1	0.170888	0.146214	0.086562	0.057996	-0.10391
R3HDM2	0.380026	0.403263	0.324827	-0.24818	-0.09001
R3HDML	0.29343	0.636321	0.982615	-0.11236	0.368178
RAB10	-0.23859	-0.30776	-0.28171	-0.22678	-0.20249
RAB11A	-0.35365	-0.28726	-0.50096	-0.40156	-0.49803
RAB11FIP2 CASC2.	0.178503	-0.03423	-0.12724	0.143326	0.120761
RAB11FIP3	0.046465	0.301135	0.21714	0.041742	0.098813
RAB11FIP4	0.141247	0.100026	0.205937	0.089934	0.163859
RAB11FIP5	-0.04619	0.06418	-0.02611	0.038944	0.114786
RAB12	0.170763	0.211529	0.410212	0.235057	0.222326
RAB13	-0.48607	-0.41285	-0.77632	-0.30993	-0.59344
RAB14	0.105322	-0.11966	-0.25544	-0.18885	-0.138
RAB15	0.041306	0.054117	0.024953	0.026599	0.086053
RAB17	-0.14636	-0.21254	-0.12342	-0.07207	-0.13846
RAB18	-0.37784	-0.41391	-0.70013	-0.58972	-0.59415
RAB19	0.705256	0.706988	0.692337	0.599624	0.696832

RAB2	-0.08246	0.017154	0.012754	0.110722	0.017988
RAB20	0.120634	0.196517	0.330328	0.170377	0.266405
RAB21	-0.05799	-0.00188	0.047035	0.098302	0.069139
RAB23	0.071024	0.182183	0.137666	-0.03588	0.21264
RAB24 PRELID1	0.096982	-0.22947	-0.32176	-0.00073	-0.14366
RAB25	0.310205	0.377535	0.558937	0.346459	0.435313
RAB26	-0.06591	-0.06442	0.087095	-0.00028	-0.03361
RAB27B	0.000621	0.295164	0.158106	0.421971	0.300923
RAB28	-0.29138	-0.38745	-0.31862	-0.3209	-0.28087
RAB2B TOX4	-0.76395	-0.51788	-1.20782	-0.67488	-0.87019
RAB30	-0.54124	-0.27412	-0.75534	-0.71068	-0.65591
RAB32	0.183877	0.183038	0.263454	0.288559	0.291277
RAB33A	0.153241	0.19514	0.143859	-0.00543	-0.15637
RAB33B	-0.24719	-0.58732	-0.45145	-0.33919	-0.37556
RAB34 RPL23A	-0.26884	0.217736	0.271966	-0.16939	0.207146
RAB35	0.039932	0.159106	0.132581	0.050991	0.062645
RAB38	-0.57075	0.387205	0.252041	0.423757	0.468559
RAB39	-0.08825	-0.03991	-0.1883	-0.21176	-0.26352
RAB39B	0.198395	0.207319	-0.16159	-0.12208	-0.03734
RAB3A	-0.40844	-0.15807	-0.18861	-0.27822	-0.14933
RAB3B	0.067412	-0.13496	0.127119	-0.02389	-0.34264
RAB3C	-1.22109	-1.0602	-1.20749	-0.98769	-1.31623
RAB3D	0.107765	0.228017	0.057801	0.076052	0.0756
RAB3GAP1	0.082639	0.18898	0.269321	0.011092	0.129464
RAB3GAP2	-0.65506	-0.98006	-1.00291	-0.80582	-0.71058
RAB3IL1	0.155019	0.293063	0.397302	0.078235	0.256477
RAB3IP	-0.03535	-0.1706	-0.1166	-0.06331	-0.1634
RAB40A	-0.34445	-0.21621	-0.43293	-0.34897	-0.29845
RAB40AL	0.595042	0.870561	0.772095	0.516931	0.709171
RAB40B	-0.08017	-0.18325	-0.16159	-0.11601	0.066041
RAB40C	-0.2565	-0.35129	-0.40676	-0.30489	-0.14415
RAB42	0.270662	0.309467	0.120327	0.288439	0.289567
RAB4A	0.595025	0.683266	0.654183	0.223157	0.508851
RAB4B	-0.92525	-1.02616	-1.17886	-0.91029	-1.03006
RAB5B	-0.51238	-0.74643	-0.91418	-0.53989	-0.78381
RAB5C	0.07052	0.133641	0.157299	0.314744	0.326502
RAB6A	0.043297	0.14438	0.020985	0.014984	-0.06291
RAB6B	-0.14873	0.072297	-0.0871	-0.07666	-0.16147
RAB6IP1	-0.11476	-0.05422	-0.21274	-0.14566	-0.18611
RAB7A	0.095358	0.135947	0.115247	-0.06983	0.082638
RAB7L1	-0.72348	-0.51188	-1.01289	-0.58461	-0.59914
RAB8B	-0.54948	-0.04124	-0.634	0.012348	-0.40071
RAB9A	-0.27724	0.01568	-0.3235	-0.16163	-0.17371
RAB9B	0.25094	0.279401	0.201587	0.00563	0.147471
RABEP1	0.126973	0.07721	0.019235	0.056589	0.005452
RABEP2	-0.22195	-0.16273	-0.2659	-0.12426	-0.15626
RABEPK	-0.05097	-0.40489	-0.42518	-0.47742	-0.66966
RABGEF1	-0.02784	-0.13053	-0.23508	-0.04161	-0.15295
RABGGTA	-0.29678	-0.38755	-0.59794	-0.43151	-0.38996
RABGGTB	-0.38581	-0.37804	-0.57114	-0.51891	-0.2063

RABIF	0.193162	0.193026	0.176427	0.159367	0.062126
RABL2A	-0.11794	0.047283	-0.19723	-0.14065	-0.09232
RABL3 GTF2E1	-1.07645	-0.81795	-1.11223	-0.63353	-0.96319
RABL4	-0.15618	0.040301	-0.38338	-0.18122	-0.26347
RABL5	0.268136	-0.07647	0.05019	0.068291	-0.10864
RAC1	0.033849	0.062132	-0.04782	0.20264	0.374832
RAC2	0.207594	0.041427	0.277085	-0.00452	-0.12511
RACGAP1	-0.00098	-0.05447	-0.20371	-0.33309	-0.27134
RAD1	-0.25894	-0.25436	-0.43936	-0.38116	-0.488
RAD17 MARVELD2	0.408959	0.598795	0.714738	0.395741	0.581277
RAD18	-0.05717	-0.15756	-0.68195	-0.50796	-0.53829
RAD21	-0.38252	-0.45914	-0.29457	-0.41733	-0.40251
RAD23A	0.345814	0.330061	0.437049	0.097375	0.264974
RAD23B	-0.22832	-0.08921	-0.19082	-0.13817	-0.15648
RAD50	0.414002	0.262027	0.064959	0.089818	0.391557
RAD51	-0.20307	-0.26701	-0.40623	-0.11879	-0.27712
RAD51L1	0.143132	-0.04891	0.105031	0.222048	-0.03014
RAD51L3 FNDC8	0.131381	0.316431	0.187645	0.090719	0.09102
RAD52	-0.08067	0.077911	0.149412	0.00158	-0.09996
RAD54B	0.067752	-0.23201	-0.27728	-0.38478	-0.39261
RAD54L2	0.306761	0.315307	0.519917	0.012685	0.258038
RAD9A	0.077085	0.105893	-0.08692	0.051538	-0.11696
RAE1	-0.04513	0.238823	0.186991	0.094199	0.243141
RAET1E	0.175074	-0.17216	0.14573	-0.27204	-0.37669
RAET1G	-0.42471	-0.27679	-0.29712	0.131644	-0.06731
RAET1L	-0.4277	0.452923	0.519652	0.588734	0.428754
RAF1	-0.00038	0.156284	-0.07114	0.008592	0.203366
RAG1	-0.28166	-0.35949	-0.21547	-0.3391	0.017091
RAG1AP1	-0.53281	-1.07384	-1.12637	-0.73601	-0.77527
RAG2	-0.36197	-0.40434	-0.6371	-0.62341	-0.3677
RAG2 C11orf74	0.229719	0.220705	0.295495	-0.13665	0.17175
RAGE	0.171112	-0.06938	-0.18566	-0.18145	-0.08026
RAI1	-0.17474	0.016351	-0.05922	-0.27908	0.0406
RAI14	-0.07326	-0.15708	0.893537	-0.17974	-0.31706
RAI16	0.073815	0.256489	0.244818	0.134437	0.074773
RAI2	-0.10589	0.512827	0.046111	0.045157	-0.04713
RALA	-0.08669	-0.04097	0.039031	0.040107	0.217542
RALB	-0.24622	-0.17973	-0.19167	-0.27761	-0.06916
RALBP1	-0.0568	0.154473	-0.03664	0.012152	0.127759
RALGDS	0.274193	0.036683	0.171032	0.140387	0.143244
RALY	-0.66558	-0.57684	-0.72422	-0.49822	-0.58972
RAMP1	0.329617	0.35603	0.439799	0.4001	0.377375
RAMP3	0.254961	0.432953	0.604474	0.385924	0.359145
RAN	0.132106	0.081787	0.284895	0.151144	0.264736
RANBP10 TSNAXIP1	-0.16475	-0.14535	-0.08034	-0.07414	-0.11763
RANBP17	0.163967	0.214805	0.04952	0.058224	-0.08573
RANBP2	0.323067	0.390499	0.520807	0.266633	0.386537
RANBP3	0.001143	0.084544	-0.2092	-0.01627	0.096009
RANBP3L	-0.18527	-0.98018	-0.29738	-0.69985	-1.00543
RANBP6	0.008544	-0.22897	-0.34308	0.055224	-0.54498

RANBP9	-0.0963	0.050522	0.108383	-0.03205	0.020798
RAP1A	0.218937	0.233333	0.231268	-0.01028	-0.03675
RAP1B	-0.20227	0.002099	-0.40248	-0.26726	-0.03617
RAP1GAP	0.19206	0.301211	0.50381	0.174052	0.178004
RAP1GDS1	-0.13449	-0.14956	-0.24002	-0.24351	-0.1957
RAP2A	-0.17209	-0.13291	-0.17337	-0.10696	-0.01645
RAP2B	-0.56483	-0.08833	-0.26578	0.136522	0.092845
RAP2C	0.0162	-0.10016	-0.02443	-0.15509	-0.18351
RAPGEF3	0.032456	0.087246	0.189215	0.197336	0.128438
RAPGEF4	0.24163	0.233619	0.332638	0.112206	-0.03221
RAPGEF6	-0.62091	-0.38566	-0.4704	-0.4666	-0.46235
RAPGEFL1	0.078608	0.198969	0.180975	-0.033	0.273443
RAPH1	-0.13088	-0.15534	-0.04943	-0.14538	-0.13027
RAPSN	0.255573	0.453743	0.265672	0.328839	0.273165
RARB	-1.06814	-0.56328	-1.03287	-0.85531	-0.5545
RARG	-0.31809	-0.36856	-0.314	-0.00145	-0.14458
RARRES1	0.311675	0.447205	0.409344	0.278444	0.398041
RARRES2	0.300296	0.357163	0.498041	0.26974	0.351007
RARRES3	0.362555	0.514896	0.601857	0.082817	0.3976
RARS	0.066609	0.184215	0.072963	-0.03574	-0.17363
RARS2 ORC3L	-0.58686	-0.63143	-0.68728	-0.35896	-0.92165
RASA1	-0.40749	-0.37646	-0.42562	-0.29235	-0.46622
RASA2	-0.35221	-0.08417	-0.02413	-0.14771	0.01427
RASA3	0.037298	0.011891	-0.07397	-0.05517	-0.07957
RASAL1	0.161096	-0.23462	0.288719	0.193861	-0.06534
RASD1	0.012247	0.045133	0.21685	0.280618	0.139972
RASD2	-0.09177	-0.1217	-0.04468	-0.0264	0.135394
RASEF	0.009356	0.419197	0.49846	0.240513	0.315833
RASGEF1A	0.012912	-0.14081	-0.21311	0.124739	0.266341
RASGEF1B	-0.03017	0.185371	0.070609	0.096568	-0.0839
RASGRF2	0.108444	0.264486	0.504578	0.314075	0.262198
RASGRP1	0.054139	0.143045	0.344818	0.080644	0.116498
RASGRP2	-0.12808	0.005276	-0.24944	0.14625	0.103366
RASGRP3	-0.01472	0.330411	-0.18202	-0.06024	-0.31772
RASGRP4	0.292943	-0.1195	0.688576	0.054152	0.090038
RASIP1 IZUMO1	0.243012	0.4565	0.433491	0.378942	0.478297
RASL10A	0.039323	0.178925	0.292526	0.203551	0.31849
RASL10B	-0.00301	0.08846	0.078055	0.000242	-0.03523
RASL11A	0.29471	0.518827	0.418108	0.320494	0.327407
RASL11B	0.19103	0.441413	0.397257	0.284259	0.302363
RASL12	0.208209	0.424048	0.409324	0.141208	0.333789
RASSF1	0.18083	0.201598	0.173633	0.154559	0.184215
RASSF1 ZMYND10	0.25467	0.428027	0.545788	0.393108	0.427063
RASSF3	-0.07327	-0.21532	-0.15988	-0.03246	-0.13935
RASSF6	0.343086	-0.83644	-0.77279	0.152749	-0.13731
RASSF8	-0.01754	-0.08393	0.119624	0.19295	0.19479
RAVER1 ICAM3	-0.55567	-0.61526	-0.69326	-0.07616	-0.63603
RAVER2	-0.17197	-0.28914	-0.13968	-0.28844	-0.30799
RAX	-0.18333	0.124131	0.197602	0.342711	0.365063
RB1CC1	-0.14825	-0.09015	-0.0992	-0.06978	-0.09231

RBBP6	-0.01863	0.037161	0.020944	0.040427	0.035565
RBBP7	-0.03338	-0.27825	-0.19092	-0.04403	-0.12784
RBBP8	-0.29038	-0.37258	-0.57336	-0.40113	-0.38077
RBBP9	-0.24342	-0.4863	-0.39235	-0.27661	-0.41678
RBCK1	-0.01847	-0.20733	-0.24276	-0.19798	0.09407
RBJ	-0.3643	-0.28759	-0.43112	-0.29455	-0.32993
RBKS BRE	0.016109	0.188029	-0.2916	-0.21216	-0.11052
RBL1	0.105152	-0.07261	-0.00721	-0.06566	0.097798
RBL2	0.029497	0.118251	0.162707	0.131094	0.16517
RBM11	-0.13399	-0.31947	-0.06941	0.010235	-0.09007
RBM12B	0.010351	-0.09877	-0.0745	-0.04572	-0.02117
RBM13	-0.00971	-0.03259	-0.11856	-0.10546	-0.18943
RBM14	-0.08464	-0.02846	-0.06938	0.006239	0.039806
RBM15	-0.73929	-0.67784	-0.65184	-0.79813	-0.6769
RBM15B	0.193878	0.098579	0.354652	0.260125	0.410188
RBM16	0.28169	0.199584	0.282598	0.21644	0.227758
RBM17	0.190557	0.242545	0.503024	0.080943	-0.15056
RBM18 MRRF	-0.38992	-0.68549	-0.95454	-0.67208	-0.82445
RBM19	-0.23621	-0.18978	-0.00233	0.013401	-0.134
RBM22	-0.50156	-0.78942	-0.82508	-0.79795	-0.78751
RBM23	-0.48859	-0.71663	-1.02539	-0.25951	-0.67661
RBM24	-0.23725	0.796224	0.788125	-0.9789	0.70844
RBM25	-0.67467	-0.55343	-1.15135	-0.825	-1.11094
RBM26	-0.30274	-0.24542	-0.04429	-0.12491	-0.07428
RBM28 AC010655.7	0.463862	0.359547	0.432542	0.13385	0.063679
RBM34	0.104072	0.090072	0.115688	0.098	-0.12804
RBM35A	-0.71939	-0.42984	-0.36913	0.305927	0.200036
RBM35B	0.197399	0.264995	0.440688	0.200195	0.393706
RBM38	0.276896	0.353279	0.465984	0.124184	0.189083
RBM39	-0.97044	-0.95724	-1.25038	-0.92071	-1.12378
RBM4	-0.61941	-0.41051	-1.03656	-1.02207	-1.57123
RBM41	0.071398	-0.31687	-0.65084	-0.34597	-0.36218
RBM42	0.167635	-0.07857	-0.09839	0.034479	-0.11742
RBM43	0.40997	0.320866	0.222475	0.471909	0.262669
RBM45	0.220443	0.208139	-0.00704	-0.02197	0.002493
RBM46	0.652937	0.828663	0.821783	0.554632	0.804944
RBM47	0.501446	0.510688	0.851484	0.477725	0.188426
RBM4B	0.027596	-0.24248	-0.40064	-0.11465	-0.0741
RBM5	-0.03231	0.134527	-0.15274	-0.09131	-0.10912
RBM6	0.115457	0.207297	0.155736	-0.05452	0.19584
RBM8A	-0.08541	-0.31017	-0.9069	-0.36937	-0.33928
RBM9	-0.94343	-0.90995	-1.14507	-0.94632	-1.05551
RBMS1	-0.45666	-0.58433	-0.58618	-0.40593	-0.5243
RBMS2	-0.38464	-0.33633	-0.62266	-0.41206	-0.45497
RBMS3	-1.01913	-0.71981	-1.01259	-0.95991	-0.47507
RBMX	0.197374	-0.01403	0.281209	-0.08549	-0.21108
RBMX2	0.009631	-0.28787	-0.46231	-0.17565	-0.24637
RBMXL2	0.143669	0.287355	0.423046	0.141335	0.340501
RBMY1F	0.621866	0.296022	0.3294	0.773133	0.205281
RBMY1J	0.467479	0.162788	0.35674	0.653819	0.375189

RBP1	0.259811	0.125958	-0.27074	0.581407	0.13854
RBP2	-0.00825	-0.02315	-0.0427	-0.32139	-0.1384
RBP3	0.142788	0.116005	0.127635	0.184422	-0.00871
RBP4	0.051121	0.198204	0.442805	0.403433	0.415991
RBP5 CLSTN3	-0.28648	-0.30363	-0.44125	-0.30403	-0.3893
RBP7	0.048071	-0.04182	0.420073	0.167353	0.214043
RBPMS	0.090222	0.1944	0.123989	0.225283	0.444239
RBPMS2	-0.13029	0.003569	0.062964	-0.01195	0.121511
RBPSUH	-0.24838	-0.33386	-0.29749	-0.27356	-0.28958
RBX1	-0.19501	-0.04246	0.003599	-0.07012	-0.0251
RC3H1	0.43848	0.678433	0.810858	0.398037	0.361244
RC3H2	0.537932	0.568	0.702928	0.302003	0.459311
RCAN3	0.085665	0.059326	-0.05284	0.20525	0.102358
RCBTB1	0.146732	0.143431	0.169859	-0.00353	0.02176
RCBTB2	0.0745	0.068999	-0.2712	0.058196	-0.01832
RCC1	-0.56773	-0.54942	-0.7111	-0.21417	-0.19077
RCC2	0.10071	0.011227	0.146927	0.030003	0.191367
RCCD1	0.373667	0.302426	0.3273	0.343227	0.34242
RCHY1 THAP6	-0.51559	-0.28865	-0.37936	-0.36983	-0.49219
RCL1	-0.22181	0.003259	-0.54855	-0.38607	-0.27532
RCN1	0.325384	0.429597	0.478191	0.357152	0.438428
RCN2	-0.12584	-0.06706	-0.20583	-0.12241	-0.27062
RCN3	0.43833	0.636273	0.647151	0.446255	0.594495
RCOR1	-0.25809	-0.37085	-0.30433	-0.24501	-0.14493
RCOR2	0.106113	0.087396	0.296781	0.13976	0.248052
RCOR3	-0.62798	-0.62417	-0.76554	-0.52292	-0.52025
RCSD1	0.25015	0.875915	0.694499	0.60456	1.35233
RCVRN	0.390943	-0.54512	0.174133	0.388488	0.565583
RD3	0.164504	-0.73407	-1.16833	-0.09977	-0.7892
RDBP SKIV2L	-0.37434	-0.81014	-0.72039	-0.48829	-0.50991
RDH11	-0.00732	0.23987	0.13522	0.114817	0.013436
RDH12	0.221185	0.434467	0.441158	0.008908	-0.6293
RDH13 EPS8L1	0.013135	-0.62744	-0.32978	-0.47839	-0.06777
RDH5	0.25111	0.012877	0.171706	0.120781	0.271087
RDH8	0.480428	-1.12561	-1.15353	-0.3392	-0.67458
RDM1	0.247692	0.388591	0.217832	0.0785	0.120221
RDX	-0.12298	0.105716	0.032633	-0.03532	0.182925
REC8	0.16921	-0.06773	-0.26916	-0.16583	0.3285
RECK	0.077763	0.365437	0.258257	0.199045	0.183683
RECQL	-0.9109	-0.85754	-1.15147	-0.891	-1.00449
RECQL4 LRRC14	0.074639	-0.15329	-0.01279	0.115361	0.12676
RECQL5 SAP30BP	-0.22052	-0.38493	-0.54239	-0.37344	-0.53247
REEP1	0.098123	0.344777	0.373626	0.152189	0.372668
REEP2	-0.04041	0.085973	0.067864	-0.1006	0.093571
REEP3	0.274584	0.474146	0.398601	0.040139	0.662953
REEP4	-0.25995	-0.29831	-0.25815	-0.16961	-0.1649
REEP5	0.192622	0.137293	0.120939	0.041318	0.073068
REG1A	-0.42868	-0.10289	-0.03475	-0.19801	0.10325
REG1B	-0.98684	-0.38698	-0.64881	-0.59791	-0.35657
REG3A	-1.14593	-1.11417	-1.09104	-0.79375	-0.6735

REG3G	0.090557	0.528318	0.45005	-0.05735	0.127103
REG4	0.621188	-1.62815	0.804839	-1.04427	-0.09104
REL	-0.19762	-0.24667	-0.24092	-0.25718	-0.2573
RELA	0.102547	-0.00565	-0.0053	0.034508	0.250178
RELB	0.004338	-0.15969	-0.04977	-0.18656	0.040916
RELN	-0.03046	-0.02592	-0.0672	-0.02771	-0.05466
RELT	0.176387	0.251154	0.221598	0.232036	0.238896
REM2	-0.01008	0.027078	-0.22442	0.047789	0.062511
RENBP	0.053413	0.260556	0.357698	0.197088	0.210456
REPIN1	0.1873	0.206729	0.546082	0.332324	0.170768
REPS1	0.066313	0.140057	0.113946	-0.05614	-0.01333
REPS2	0.140375	0.16178	0.313344	0.076914	0.255513
RERE	-0.27381	-0.04248	-0.34733	-0.02492	-0.0512
RERG	-0.10155	-0.04109	0.240824	0.100368	-0.25058
RERGL	-0.59016	-0.58222	-0.54918	-0.51598	-0.67729
REST	-0.01958	0.065082	0.096127	0.094057	0.202403
RET	-0.11787	-0.16903	-0.17523	0.082574	-0.042
RETN	0.081263	-0.39299	-0.02782	0.151431	0.317727
RETNLB	-0.03078	-0.20905	-0.09746	-0.21889	-0.9523
RETSAT RBED1	0.015393	-0.79363	-1.23464	-0.5896	-0.35455
REV1L	0.005157	-0.0399	-0.04622	-0.04916	0.037685
REV3L	-0.24822	-0.06615	-0.1873	-0.19583	-0.20495
REXO1	0.002397	-0.16205	-0.15568	-0.15186	-0.16195
REXO2	-0.38664	-0.08732	-0.37584	-0.24232	-0.2139
REXO4	0.065311	0.08087	0.119277	0.041528	0.027654
RFC1	0.159307	0.283078	0.389435	0.137794	0.223417
RFC2	-0.0464	-0.24669	-0.3844	-0.15877	-0.15869
RFC3	-0.51733	-1.08961	-1.29411	-1.06094	-1.25927
RFC4	-0.56608	-0.22085	-0.55511	-0.37613	-0.44744
RFC5	0.109976	0.217648	0.284577	0.104525	0.090918
RFESD	0.322003	0.503818	0.362839	0.170535	0.343386
RFK	-0.07191	-0.06643	-0.07745	-0.08191	-0.0339
RFNG GPS1	-0.00231	-0.13595	-0.04386	0.010083	0.113293
RFPL1	0.437453	0.323664	0.241028	0.04886	0.036202
RFPL2	0.341807	0.197233	0.564362	0.332222	0.420115
RFPL3	0.301636	0.142991	0.552807	0.074999	-0.05033
RFPL4B	0.711345	0.890452	1.194788	0.509997	0.815484
RFT1	-0.47397	-0.05994	-0.77595	-0.5453	-0.46966
RFTN1	0.152477	0.111173	-0.43578	0.02876	0.116148
RFTN2	-0.61674	0.347296	0.737254	0.26886	-0.21266
RFWD2	-0.14296	-0.16377	-0.29711	-0.18387	-0.0686
RFWD3	-0.19866	0.021826	-0.28185	0.233572	0.085516
RFX1	-0.32706	-0.17485	-0.61311	-0.38023	-0.39483
RFX2	0.00492	0.248463	0.077705	0.280871	0.242233
RFX3	-0.3937	-0.23623	-0.39205	-0.30017	-0.2418
RFX5	-0.38254	-0.2595	-0.58745	-0.57106	-0.37915
RFXAP	-0.18343	-0.16259	-0.62621	-0.40509	-0.63217
RFXDC1	-0.1963	0.010011	0.193477	0.269765	0.295017
RG9MTD1	0.189658	0.1901	0.236591	-0.08915	-0.02045
RG9MTD2	-0.69342	-0.53261	-0.67866	-0.52471	-0.65816

RGL1	0.288359	0.336799	-0.02731	-0.35045	0.213289
RGL2	-0.65303	-0.62019	-0.76154	-0.60188	-0.78365
RGL3	0.18687	0.174306	0.085165	0.283983	0.029483
RGL4	0.64431	0.848337	0.868876	0.711185	0.921457
RGMA	-0.01579	0.109644	0.300557	0.021015	0.189802
RGMB	-0.5009	-0.56387	-0.40097	-0.33403	-0.5497
RGN	0.211789	0.359835	0.239942	0.148194	0.300966
RGPD1	0.405242	-0.13467	0.263971	-0.27946	-0.36149
RGPD5	0.183253	-0.03377	0.253946	0.048776	0.081526
RGR	0.219852	0.277445	0.594395	0.319633	0.274705
RGS1	0.185593	0.332604	0.33926	0.00318	0.093923
RGS11	0.186397	0.09942	0.340775	0.246558	0.361688
RGS13	0.055241	-1.16174	-0.80553	-0.53684	-0.65653
RGS14	0.394347	0.481373	0.633586	0.18898	0.498378
RGS16	0.094851	0.090703	-0.57013	-0.00045	-0.37599
RGS17	-0.07577	0.040684	-0.12999	-0.10146	-0.06137
RGS18	0.273662	0.480179	0.26194	0.102437	0.367313
RGS19 OPRL1	0.155151	-0.02848	-0.07357	0.119536	0.213097
RGS2	-0.29357	-0.31527	-0.16952	-0.34787	-0.16463
RGS21	0.115127	-0.00576	-0.63452	-0.30991	-0.05179
RGS22	0.270023	0.442273	0.578851	0.24926	0.402817
RGS4	0.387794	-0.12756	0.193806	0.257985	-1.07651
RGS5	-0.46113	-0.97391	-0.80273	-0.64893	-0.64536
RGS7	0.095799	0.179702	0.154301	0.091825	0.026823
RGS7BP	-0.15772	-0.22203	0.037077	0.166213	0.038372
RGS8	0.099594	-0.02535	-0.34272	-0.9334	-0.08153
RGS9	0.178296	0.334772	0.057927	-0.0308	0.171307
RGSL2.	-1.37612	-1.46299	-1.71262	-1.30393	-1.42389
RGSL2. RGSL1	0.103607	0.196594	0.027933	-0.19487	-0.05373
RHAG	-0.07796	-0.71273	-0.69362	-0.19457	-0.82919
RHBDD1	0.100071	0.344413	0.101559	-0.20199	0.219484
RHBDD2	0.387306	0.153647	0.071824	0.03432	-0.13998
RHBDD3 EWSR1	-0.45642	-0.50407	-0.39129	-0.39338	-0.50996
RHBDF1	0.044176	-0.00868	-0.04199	0.102663	0.130588
RHBDF2	0.212122	0.129359	0.164597	0.268767	0.17941
RHBDL1	0.104893	0.256595	0.145543	0.126638	0.201101
RHBDL2	0.363872	0.685709	0.376584	0.278891	0.377991
RHBDL3	0.085225	0.131126	0.118313	0.044155	0.212935
RHBG	0.223522	0.547007	0.522462	0.307608	0.340063
RHCE	0.252347	0.36807	0.472286	0.276775	0.225557
RHCG	0.289446	0.30017	0.453888	0.200757	0.177115
RHD	0.171143	0.271493	0.288228	0.060632	0.131065
RHEB	-0.18637	-0.04004	-0.42725	0.086938	0.015226
RHEBL1	0.074141	-0.04431	-0.03792	-0.15178	-0.12009
RHO	0.441064	0.45836	0.485155	0.181006	0.431383
RHOA TCTA	0.087277	-0.03064	-0.24322	-0.11626	-0.01555
RHOB	-0.10373	0.000796	0.006639	-0.03377	-0.06798
RHOBTB2	-0.19578	-0.04767	-0.01504	-0.09303	0.039874
RHOBTB3	-0.02231	-0.29808	-0.3237	-0.14252	-0.34893
RHOC	-0.03066	0.010592	0.079249	0.023445	0.20605

RHOD	0.369035	0.462162	0.476841	0.290159	0.335483
RHOF	0.121145	0.286405	0.352838	0.200132	0.285725
RHOG	-0.1028	0.001354	0.053999	-0.04476	0.085004
RHOH	0.530321	1.031684	1.026488	0.782343	0.755477
RHOJ	-0.34692	-0.50542	-0.40944	-0.37443	-0.28839
RHOU	0.325212	0.251216	0.494712	0.216225	0.299866
RHOV	-0.01355	0.062837	0.103006	0.011684	0.073915
RHOXF1	0.167762	0.309764	0.193125	0.191646	0.287122
RHOXF2	0.56437	0.635035	0.395435	0.491155	0.599536
RHPN1	0.197756	0.359866	0.282054	0.294654	0.280498
RHPN2	0.081366	0.14376	0.082454	0.147366	0.174728
RIC3	0.128756	-0.01808	-0.39737	-0.16639	0.073533
RIC8B	-0.18648	-0.24922	-0.41572	-0.13658	-0.3758
RIF1	-0.1573	-0.16514	-0.24061	-0.08307	0.06658
RILP PRPF8	0.283377	0.393707	0.360175	0.296069	0.193854
RILPL1	0.077101	0.052838	0.182381	0.056309	0.128323
RILPL2	0.086912	-0.12705	0.013309	0.00199	0.010807
RIMBP2	0.050771	0.168391	0.598226	0.141306	0.426125
RIMS1	-0.33407	-0.30206	-0.10675	-0.02899	-0.3282
RIMS2	0.207776	0.34708	0.315322	-0.08479	-0.06897
RIMS3	0.099434	0.215887	0.085687	-0.00113	0.117822
RIMS4	0.051385	0.035268	-0.10405	0.038624	0.003965
RIN1	-0.30385	-0.30316	-0.36213	-0.10724	-0.11532
RIN2	0.427075	0.67925	0.717202	0.455678	0.584409
RIN3	0.306835	0.068566	0.482153	0.318808	0.437318
RINT1	0.235759	0.174786	0.410044	0.097538	0.085396
RIOK1	0.409777	0.539648	0.679883	0.196566	0.386106
RIOK2	-0.83486	-0.45434	-0.99839	-0.78377	-0.8471
RIOK3	-0.13341	-0.36651	-0.48408	-0.07686	-0.36737
RIPK1	0.416589	0.648915	0.781423	0.309363	0.587924
RIPK2	-0.22199	-0.18818	-0.21313	-0.22192	-0.16141
RIPK3	-0.0435	-0.10395	0.329784	0.145205	0.260796
RIPK4	-0.22173	0.033489	0.268619	0.280093	0.391118
RIPK5	0.185689	0.318153	0.338275	0.012516	0.099163
RIPX	-0.20238	-0.32951	-0.54262	-0.3318	-0.37364
RIT1	0.157805	0.190967	0.21055	0.054071	-0.00314
RIT2	0.427878	-0.45048	-0.82908	-0.04002	-0.62258
RKHD1	-0.1535	-0.02328	-0.14227	-0.10063	-0.30776
RKHD3	-0.44555	-0.45516	-0.49087	-0.3867	-0.30267
RLBP1	0.392724	0.53071	0.41733	0.243906	0.515928
RLBP1L1	-0.58529	-0.35826	-0.54172	-0.63384	-0.64725
RLBP1L2	-0.14976	0.085011	0.139725	-0.07097	0.075338
RLF	0.239286	0.305552	0.231484	0.111091	0.15554
RLN1	0.199276	-0.27534	-0.49353	-0.39285	-0.50005
RLN2	0.160544	-0.40065	-0.86464	-0.45109	-0.66517
RLN3	0.219968	0.346737	0.382312	0.218231	0.441992
RLN3 IL27RA	0.036381	-0.21806	-0.10186	-0.18779	-0.1218
RLTPR	0.171743	0.324294	0.336543	0.222306	0.093314
RMND1 C6orf211	-0.565	-0.43785	-0.64913	-0.47357	-0.7436
RMND5A	-0.2144	-0.25003	-0.14527	-0.05537	-0.05431

RMND5B	-0.03301	0.000939	0.227476	0.037323	0.109766
RNASE1	0.365829	-0.62922	-0.5676	-0.24732	-0.19006
RNASE10	0.213504	0.294932	-0.87032	-0.72534	-0.57558
RNASE11 RNASE12 AL163195.5	0.068034	0.519861	-1.04639	-0.69968	-1.07149
RNASE13	-0.21639	0.299913	-0.02996	-0.60975	0.161472
RNASE2	0.119809	-1.86522	-1.53276	-0.85336	-0.85174
RNASE3	0.161541	-1.33814	-1.13465	-0.81458	-0.80188
RNASE6	0.167149	-0.54285	-0.22609	-0.26154	-0.06458
RNASE7	0.22423	0.337348	0.378382	-0.23392	0.300571
RNASE8	0.016685	0.2379	0.029457	-0.40296	-0.09297
RNASE9	-0.00133	0.15557	-0.9229	-0.746	-0.72123
RNASEH1	0.158865	0.109013	0.200162	0.029062	0.151911
RNASEH2A	0.323257	0.287552	0.299883	0.247326	0.294306
RNASEH2B	0.198303	0.259011	0.269653	0.10511	0.360382
RNASEH2C	0.28878	0.407879	0.474461	0.210422	0.28017
RNASEL	0.25033	0.385999	0.32488	0.083609	0.252861
RNASET2	0.027176	0.205701	0.134582	-0.03713	0.164078
RND1	-0.26396	-0.14678	-0.15504	-0.28524	-0.27251
RND2	-0.33287	-0.24456	-0.91907	-0.34946	-0.16658
RND3	-0.1929	-0.05137	-0.3922	-0.33823	-0.12099
RNF10	0.316803	0.358023	0.353165	0.212029	-0.05554
RNF103	0.185654	0.303772	0.259365	0.11604	0.356116
RNF11	-0.22092	-0.1419	-0.06856	-0.3445	-0.1622
RNF111	0.159617	-0.13009	-0.45205	-0.19302	-0.54599
RNF113A NDUFA1	0.068032	-0.31284	0.380584	-0.33414	-0.27041
RNF12	0.213373	0.344788	0.552322	0.156984	0.215016
RNF122	0.026091	-0.13229	-0.01903	0.08946	0.040687
RNF123	0.379374	0.225305	0.431962	0.363179	0.288915
RNF125	0.260408	0.138798	0.346705	0.260947	0.336184
RNF126	0.22636	0.268988	0.220812	0.194365	0.160016
RNF13	0.087281	0.036253	-0.3007	-0.4712	-0.24843
RNF130	0.296076	0.298823	0.490426	0.233819	0.255435
RNF135	0.537104	0.680956	0.905833	0.571614	0.685652
RNF138	0.014185	0.172876	0.09862	0.043839	0.144132
RNF139	-0.60213	-0.76387	-0.7403	-0.60048	-0.59828
RNF141 AC009532.9	0.059254	-0.27707	-0.2282	-0.11799	0.000418
RNF144A	0.294283	0.374056	0.559976	0.163586	0.258611
RNF145	-1.46479	-1.65213	-1.72361	-1.49237	-1.68895
RNF146	-0.40997	-0.2592	-0.55793	-0.41421	-0.59299
RNF149	0.287268	0.373325	0.408203	0.131997	0.316911
RNF150	-0.30121	-0.49807	-0.49958	-0.24557	-0.46783
RNF152	-0.52603	-0.36115	-0.79165	-0.31003	-0.50605
RNF157	0.024656	0.190137	0.138406	0.045108	0.240315
RNF165	0.044841	-0.0202	0.134102	0.073807	0.105465
RNF166 C16orf84	0.102638	0.097409	0.310598	0.136573	0.189546
RNF168	-0.34142	-0.34027	-0.34151	-0.22823	-0.28796
RNF17	0.575765	0.494372	0.818592	0.551622	0.553619
RNF170 HOOK3	-0.02766	0.089371	-0.01423	0.023067	-0.12237
RNF175	0.233158	0.329085	0.320838	0.243215	0.311359
RNF180	0.11619	-0.16912	0.226037	0.409131	0.346377

RNF181	-0.20247	-0.32981	-0.5178	-0.49515	-0.35109
RNF182	-0.31372	-0.20495	-0.20832	-0.26816	-0.177
RNF183	0.350105	0.477691	0.650922	0.256	0.310311
RNF185	0.121243	0.067202	-0.24466	-0.05652	-0.04802
RNF186	0.527191	0.423634	0.725359	0.316392	0.675932
RNF19B	-0.00637	0.064221	0.065332	0.107509	0.167533
RNF2	0.206903	0.293787	0.247112	0.265679	0.196261
RNF208	0.332441	0.356252	0.58691	0.363591	0.549678
RNF212	0.191701	0.186399	0.294146	0.208824	0.21782
RNF213	0.312434	0.351748	0.4982	0.523113	0.511959
RNF215	0.048832	-0.08729	-0.09675	-0.0332	0.017535
RNF216	0.019001	0.379172	-0.06543	0.140756	0.086263
RNF217	-0.50519	-0.35387	-0.83612	-0.54088	-0.79291
RNF24	0.060285	0.019371	0.097136	0.042609	0.038383
RNF25 STK36	-0.45348	-0.69097	-0.89845	-0.76231	-0.99664
RNF26	0.061315	0.225019	0.224877	0.129074	0.101319
RNF31 IRF9	-0.01705	0.021832	-0.10811	0.055977	0.168966
RNF32	0.148184	0.239639	0.309666	0.404302	0.362403
RNF34	-0.16436	-0.61183	-0.56296	-0.31131	-0.34004
RNF38	-0.13676	-0.25617	-0.41761	-0.36105	-0.16679
RNF39	-0.12578	0.410974	0.582636	0.229709	0.334654
RNF4	-0.01723	0.051928	-0.02338	0.014342	0.03285
RNF41	-0.07806	-0.13864	-0.37219	0.031119	-0.34055
RNF43	-0.52749	-0.78088	-0.47855	-1.26886	-1.19365
RNF44	-0.63866	-0.46665	-0.65002	-0.64555	-0.53684
RNF6	-0.1418	-0.05604	-0.19535	-0.28412	-0.23474
RNF7	-0.02276	-0.00756	0.123411	-0.06525	-0.15384
RNF8	0.000273	0.127494	0.110673	-0.06744	-0.11623
RNFT1	0.162406	0.197913	-0.01296	0.136672	0.254262
RNGTT	-0.15469	-0.26813	-0.36198	-0.15428	-0.48141
RNH1	-0.03956	0.079242	-0.04702	-0.0058	0.319104
RNPEP	0.343492	0.412967	0.430577	0.356739	0.250031
RNPEPL1	0.08723	-0.11483	0.025087	0.00677	0.155188
RNPS1	-0.08012	0.122298	-0.04338	0.047562	0.020163
RNUXA	0.218265	0.185746	0.492414	0.177784	0.197435
ROBO2	-0.39141	0.609903	-0.28645	0.060703	-0.07993
ROBO3	-0.68191	0.154888	-0.02816	0.230701	0.0214
ROBO4	-0.12831	0.24816	0.316028	0.253489	0.002134
ROCK1	0.06396	0.071131	0.23188	0.145053	0.315481
ROCK2	-0.05959	0.092891	-0.09229	0.129806	0.126485
ROD1	0.000309	-0.04047	0.066802	0.067923	0.027048
ROGDI AC020663.7	-0.00283	0.050431	0.281957	0.074044	0.165365
ROPN1	0.108412	0.441465	0.450972	0.106258	0.041523
ROPN1B	-0.08184	0.061758	-0.03916	0.011426	-0.09728
ROPN1L	-0.02819	-0.11799	-0.21873	0.190823	-0.12823
ROR1	-0.19389	-0.20489	-0.14709	-0.17244	0.038412
ROR2	0.070193	0.34016	0.225519	0.195248	0.193747
RORB	-0.4583	-0.37088	-0.446	-0.31914	-0.31123
ROS1	0.410137	-0.61129	-0.69932	-0.55721	-0.51987
RP1	0.293972	-1.10369	-0.74731	-0.99562	-1.16161

RP11-114H20.1	0.035332	0.034076	-0.80512	-0.26542	-0.42564
RP11-119B16.1	-0.03778	0.146676	-0.04539	0.023758	0.043652
RP11-11C5.2 C13orf34	-0.34131	-0.31253	-0.25161	-0.31105	-0.34308
RP11-16L6.1	0.098602	0.089127	0.129934	0.198187	0.239298
RP11-216L13.5	0.257332	0.35017	0.412426	0.263946	0.417216
RP11-265F14.1	0.452795	0.567179	0.64719	0.323331	0.662909
RP11-265F14.2	0.692199	0.801273	0.599164	0.29929	0.4213
RP11-269H4.1	-0.04796	0.109155	0.17852	0.11161	0.190411
RP1-127L4.6	0.282373	-0.08266	0.333414	0.127313	0.108389
RP11-295F4.1	-0.03635	-0.4795	-0.37032	-0.29373	-0.4538
RP11-29B2.3	0.475146	0.732503	0.911582	0.348243	0.274929
RP11-35F15.2	0.332696	0.410519	0.185999	0.129674	0.103593
RP11-369J21.6	-0.09356	-0.1708	-0.30815	-0.16517	-0.12044
RP11-402L1.1 ZNF485	0.309466	0.458656	0.368975	0.137879	0.230189
RP11-410N8.4	0.376444	0.47873	0.375325	0.095705	0.276294
RP11-438H8.8	0.376575	0.481831	0.561812	0.428431	0.439867
RP11-462B18.5	-0.05184	0.282608	0.489561	0.279906	0.554887
RP11-47G11.1	0.521608	0.564747	0.703012	0.420046	0.534751
RP11-535K18.2	0.337088	0.260614	0.625508	0.397073	0.346221
RP11-63L7.3	0.304133	0.298456	0.437023	0.226143	0.285712
RP11-68I18.1	-0.00123	-0.01601	0.157348	0.001507	0.004087
RP11-738I14.8	0.031093	0.280918	0.255943	0.129328	0.376098
RP11-85G18.4	0.09307	0.358838	0.143245	0.035549	-0.13263
RP11-90L1.3	0.120734	0.152259	0.132561	0.241407	0.332599
RP11-93B14.6	0.080232	0.219552	0.173855	0.153294	0.193808
RP11-94I2.2	0.489452	0.223804	0.064787	-0.25652	0.200992
RP13-36C9.1	0.439423	0.533038	0.617549	0.355309	0.315946
RP13-36C9.3	0.231828	0.434981	0.240495	0.432842	0.383273
RP13-36C9.6	0.501541	0.160426	0.119495	0.346229	0.615482
RP13-36C9.7	0.214181	0.367871	0.14518	0.273539	0.344063
RP2	0.076934	-0.25067	0.150069	-0.26276	-0.3203
RP3-402L9.2	0.182184	0.149822	-0.00604	-0.22957	0.072671
RP3-422G23.2 HEBP2	-0.08826	0.203812	0.168401	0.052784	0.016057
RP3-474I12.8	0.433758	0.477034	0.258782	0.34677	0.255878
RP4-604K5.1 HS2ST1	-1.18696	-1.01947	-1.0898	-1.16033	-0.62673
RP4-695O20__B.10	0.482378	0.152991	-0.25269	0.285487	-0.09508
RP4-788L13.1	0.00402	-0.15228	-0.49709	-0.52748	-0.3295
RP5-1000E10.4	-0.01286	0.223629	-0.01572	-0.10462	-0.2731
RP5-1022P6.2	0.185938	0.434711	0.388748	0.216088	0.256668
RP5-1077B9.4	0.091716	0.136851	0.062401	0.099386	0.099653
RP5-1091N2.8	0.243053	0.531028	0.212991	0.230616	0.256549
RP5-1187M17.10	0.21118	0.425534	0.450667	0.272846	0.287236
RP5-990P15.2	-0.06587	0.048422	-0.17849	-0.11495	0.127199
RP6-166C19.11	0.674668	0.727829	0.822205	0.336412	0.814282
RP6-213H19.1	-0.02369	-0.0733	0.06622	-0.07791	0.114944
RPA2	-0.06011	-0.4266	-0.52328	-0.51728	0.003832
RPA3	-0.3334	-0.43272	-0.53125	-0.30248	-0.27373
RPAP1	0.339613	0.319714	0.466016	0.272884	0.168413
RPAP3	0.170496	-0.30283	-0.69609	-0.12337	-0.58714
RPE	-0.67052	-0.68621	-0.76846	-0.57269	-0.76188

RPE65	-0.42558	-0.31546	-0.60936	-0.57097	-0.22615
RPEL	0.313149	0.272965	0.668239	0.643676	0.177824
RPGR	0.126356	-0.1001	0.014815	-0.17556	-0.07605
RPGRIP1	0.409465	-0.172	0.658929	0.126722	0.445388
RPGRIP1L FTO	-0.3755	-0.34917	-0.33968	-0.48361	-0.40077
RPH3A	-0.40319	-0.52458	-0.60638	-0.73395	-0.66741
RPH3AL	0.259634	0.179227	0.146028	0.041222	0.280261
RPIA	-0.08612	-0.0336	-0.12843	0.066928	0.040576
RPL10	0.065237	-0.5147	-0.10374	-0.44144	-0.39282
RPL10A	0.182903	-0.12045	-0.11317	-0.12603	-0.18703
RPL10L	0.41862	0.681203	0.629486	0.472709	0.668016
RPL11	0.067143	-0.28842	-0.5206	-0.10044	-0.16457
RPL12 LRSAM1	-0.40512	-0.43521	-0.54394	-0.4308	-0.42101
RPL13	0.249167	0.357139	0.275367	0.173469	0.211446
RPL13A	-0.41114	-0.25549	-0.50165	-0.3977	-0.59467
RPL14	0.18739	0.186116	0.258123	0.107604	0.084079
RPL18 SPHK2	-0.81536	-0.78797	-0.83943	-0.71476	-0.69787
RPL18A	0.12004	0.08166	0.153487	0.047723	-0.03277
RPL19	0.102752	-0.1958	-0.39009	-0.10241	-0.32627
RPL21	-0.69527	-0.53434	-0.75043	-0.44127	-0.46894
RPL22	-0.16355	0.149781	0.079769	0.025842	0.163899
RPL23	-0.04749	-0.23115	-0.23236	-0.34612	-0.70671
RPL24	-0.02325	-0.28727	-0.67197	-0.23747	-0.54588
RPL26	-0.09817	0.212105	-0.12942	-0.04141	-0.0379
RPL27A	-0.30534	-0.4733	-0.70134	-0.47388	-0.28225
RPL28	-0.35017	-0.23858	-0.43805	-0.26829	-0.25449
RPL29	-0.1426	0.186677	-0.09928	-0.0999	0.083847
RPL3	-0.49028	-0.18813	-0.19772	-0.47622	-0.49138
RPL30	-0.86255	-0.75846	-0.97311	-0.75363	-0.7428
RPL31	-0.11067	-0.066	0.255885	-0.01971	0.09277
RPL32	0.071494	0.074076	-0.02139	-0.22339	-0.16195
RPL34	-0.03759	-0.5557	-0.34539	-0.35996	-0.31364
RPL35 ARPC5L	-0.07483	-0.11536	-0.14726	-0.03906	-0.05969
RPL36	-0.17599	-0.17398	-0.32829	-0.29634	-0.45956
RPL36A	0.099589	-0.25941	-0.8541	-0.40852	-0.45523
RPL36AL MGAT2	-0.36791	-0.43922	-0.40272	-0.23495	-0.31187
RPL37	-0.36059	-0.76567	-0.40813	0.099283	-0.1151
RPL37A	-0.38096	-0.68437	-0.55756	-0.25336	-0.25432
RPL38	-0.09401	-0.55243	-0.3527	-0.17613	-0.65359
RPL39	-0.07953	-0.3425	-0.50641	-0.34775	-0.42471
RPL39L	0.223207	0.02116	-0.03377	-0.09795	-0.01982
RPL3L	0.176358	-0.05726	0.115195	0.010581	0.046843
RPL4 ZWILCH	-0.75222	-0.85225	-0.87781	-0.80904	-1.04287
RPL5	-0.12158	-0.19858	-0.363	-0.36636	-0.12336
RPL6	0.020215	0.113735	-0.11616	-0.02805	-0.04799
RPL7	-0.29344	-0.26939	-0.15138	-0.19984	-0.10147
RPL8	-0.11371	-0.35764	-0.28064	-0.30769	0.028363
RPL9 LIAS	-0.57237	-0.54838	-0.852	-0.58186	-0.71405
RPLP0	-0.2322	-0.36898	-0.35479	-0.43738	-0.33022
RPLP2	-0.04853	-0.07288	-0.04756	0.006172	0.055153

RPN1	0.088018	0.176387	0.388249	0.183162	0.206555
RPP14	0.105415	0.175697	0.262369	0.114936	0.076712
RPP21	-0.01919	-0.07734	-0.42469	-0.3369	-0.33676
RPP25	0.085678	0.049305	-0.22193	0.02136	-0.27805
RPP40	-0.16367	-0.14339	-0.25339	-0.07261	-0.06115
RPRM	-0.31105	-0.17625	0.04488	-0.11731	0.038271
RPRML	0.043263	0.139156	0.096484	-0.06277	0.016247
RPS10	0.269166	0.368956	0.310014	0.173542	0.089505
RPS11	-0.30102	-0.11071	-0.53282	-0.2617	-0.61824
RPS12	-1.05922	-0.79275	-0.7342	-0.3719	-0.94631
RPS13	-0.40426	-0.51239	-0.76869	-0.35885	-0.01242
RPS14	-0.22971	-0.12804	-0.15844	-0.11494	-0.19813
RPS15	0.023928	0.135251	0.094935	0.051287	0.152485
RPS15A	0.038243	-0.18992	-0.20863	-0.06447	-0.08607
RPS16	-0.11842	-0.36212	-0.17977	-0.1079	-0.25589
RPS17	0.227253	-0.10906	-0.09166	0.135606	0.166624
RPS18 B3GALT4	0.149095	-0.03006	0.311723	0.270325	0.112593
RPS19	-0.37029	-0.29521	-0.36126	-0.3976	-0.44288
RPS19BP1	0.084399	0.079903	0.048266	-0.15438	-0.10665
RPS2	0.036223	0.145649	0.139429	0.029201	0.044351
RPS20	0.173645	0.31856	0.239335	0.192155	0.382133
RPS21	-0.20837	-0.34784	-0.46433	-0.27317	-0.44421
RPS23	-0.21202	-0.27028	-0.34223	-0.40667	-0.38087
RPS24	-0.38998	-0.03337	-0.31235	-0.19341	-0.23739
RPS25 TRAPPC4	-0.79753	-0.6714	-1.30099	-0.76674	-1.20233
RPS26	-0.2426	-0.19765	-0.87726	-0.31116	-0.41115
RPS27	0.045606	0.012544	-0.02589	-0.14325	-0.06575
RPS27L	-0.30209	-0.28794	-0.44838	-0.18908	-0.25796
RPS3	-0.07055	0.216232	0.028805	-0.16274	-0.27016
RPS3A	0.097926	-0.11343	0.056286	0.268061	-0.07253
RPS4X	0.109609	-0.01774	-0.11058	0.01267	0.184157
RPS4Y1	0.298356	0.201036	0.313017	0.333763	0.296957
RPS4Y2	0.626651	0.125464	0.86531	0.526179	0.360016
RPS5	-0.21731	-0.24616	-0.40646	-0.13572	-0.19774
RPS6	-0.58076	-0.79767	-1.34721	-0.86985	-0.63319
RPS6KA3	-0.15429	-0.11127	0.058983	-0.15343	-0.16169
RPS6KA4	0.213532	0.133665	0.157974	0.075895	0.171956
RPS6KA5	-0.45429	-0.49295	-0.57442	-0.41426	-0.45892
RPS6KB2	0.0785	0.11436	0.045547	0.167789	-0.04323
RPS6KC1	0.288346	0.07679	0.142611	0.226291	-0.08931
RPS6KL1	0.442987	0.338011	0.27142	0.288456	0.481039
RPS7	0.070462	0.36627	0.202551	0.197775	0.153895
RPS8	0.197551	0.059403	0.167569	-0.03609	-0.08657
RPS9	-0.07675	-0.20706	-0.16861	0.040015	-0.30759
RPSA	-0.05271	0.03313	-0.35948	-0.28279	-0.01588
RPUSD1 CHTF18	0.131354	-0.09004	0.133411	0.076339	0.058591
RPUSD2	-0.42826	-0.3284	-0.53736	-0.13061	-0.59444
RPUSD3	0.175537	0.087139	0.193756	-0.07079	0.134754
RPUSD4 FAM118B	-0.73754	-0.4398	-1.05797	-0.75043	-1.23954
RRAD	0.103261	0.247209	0.463113	0.191761	0.242241

RRAGA	-0.10239	-0.08448	-0.2001	-0.11671	-0.22165
RRAGB	0.058069	-0.06298	0.101131	-0.18627	-0.12975
RRAGC	-0.21814	-0.24037	-0.23994	-0.13135	-0.09339
RRAGD	-0.03786	-0.05571	-0.0148	-0.04513	-0.12223
RRAS	-0.28607	-0.41914	-0.37288	-0.24962	-0.28923
RRAS2	-0.04364	0.088767	0.211722	0.071718	-0.03281
RRBP1	-0.19992	-0.18217	-0.19936	-0.06939	-0.15863
RREB1	0.218154	0.292535	0.000761	-0.0505	0.253727
RRH	0.043187	0.153105	-0.11145	-0.14561	-0.1393
RRM1	-0.10401	0.060629	-0.10313	-0.02812	0.062187
RRM2	-0.20619	-0.39534	-0.18418	-0.17346	0.0578
RRM2B	-0.28637	0.025309	0.011682	-0.09882	-0.0631
RRP1	0.113019	0.141317	-0.07442	0.124969	0.257195
RRP12	-0.14428	-0.42463	-0.34305	-0.30338	-0.21433
RRP15	-0.3041	-0.45545	-1.06447	-0.1845	-0.57663
RRP9 PARP3	0.068638	0.169262	-0.00827	0.060557	0.122272
RRS1	0.04719	-0.17035	-0.47136	-0.27759	-0.39305
RS1	0.487835	0.739228	0.896063	0.301029	0.489402
RSAD1	0.220237	0.308004	0.133954	0.159492	0.278908
RSAD2	0.315002	0.189685	0.529707	0.260287	-0.59977
RSBN1	0.244393	0.19917	0.294712	0.00447	0.241803
RSBN1L	-0.30165	-0.3067	-0.57592	-0.21617	-0.25525
RSF1 C11orf67	-0.62171	-0.49084	-0.70993	-0.55057	-0.58987
RSHL1 SYMPK	0.089302	0.262803	0.129305	0.202913	0.17203
RSHL2	-0.18341	-0.12291	-0.54726	-0.45895	-0.26521
RSHL3	-0.89847	-0.60387	-1.09033	-0.44206	-1.02755
RSL1D1	-0.05425	-0.26313	-0.20059	-0.25265	-0.25246
RSPH10B	0.690617	0.93283	0.947434	0.60168	0.472197
RSPO1	-0.19909	-0.10948	0.034628	0.167525	0.108113
RSPO2	-0.34283	-0.19641	0.369811	-0.45079	0.091807
RSPO3	-0.57776	-0.18191	-0.23449	-0.00778	-0.31523
RSPO4	-0.01708	0.121462	0.281885	0.175639	0.312933
RSRC1	-0.45047	-0.07688	-0.5117	-0.17801	-0.64193
RSRC2 KNTC1	-1.36478	-1.33893	-1.48405	-1.22382	-1.52651
RSU1	-0.33046	-0.16945	-0.47966	-0.27861	-0.3823
RTBDN	0.206491	-0.31941	-0.5996	0.064214	-0.27831
RTCD1	-0.3786	-0.33711	-0.387	-0.44213	-0.46107
RTDR1 RAB36	0.224661	-0.02703	0.041613	0.166941	0.09402
RTF1	-0.32613	-0.30639	-0.32685	-0.16052	-0.37952
RTKN	0.271496	0.453396	0.54772	0.297466	0.305566
RTN2	0.341978	0.584688	0.839426	0.474376	0.72244
RTN2 AC138534.3	-0.11811	-0.1021	0.359593	0.010236	0.243199
RTN3	-0.08825	0.140079	0.152769	0.060302	0.030196
RTN4IP1 QRSL1	0.028033	0.116516	-0.18477	-0.04365	-0.13922
RTN4R	0.056932	0.162216	0.190857	0.030491	0.074556
RTN4RL2	-0.13445	-0.27645	-0.22234	-0.11175	0.025712
RTP2	0.142665	-0.18956	-0.47977	0.079142	-0.01904
RTP3	0.305467	0.438951	0.36873	0.154158	0.313754
RTP4	0.730685	0.532267	0.277032	0.520642	0.478963
RTTN	-0.31728	-0.51784	-0.92172	-0.70202	-0.50288

RUFY1	0.258611	0.278292	0.407474	0.058262	0.141848
RUFY2	0.122774	0.211578	0.226011	0.069989	0.055121
RUFY4	0.337331	0.409784	0.430021	0.146903	-0.03367
RUNDC2A	-0.00312	-0.44293	-0.28254	-0.20154	-0.39447
RUNDC3A	-0.38602	-0.58142	-0.45418	-0.68085	-0.35962
RUNX1	-0.34041	-0.12233	-0.10967	-0.15164	0.060264
RUNX1 AF015262.2	-0.23759	0.315069	0.652384	0.555695	-0.12287
RUNX2	-0.64331	-0.49314	-0.5224	-0.30159	-0.33675
RUSC2	0.082182	0.057828	0.275416	0.139029	0.126845
RUVBL1	0.233009	0.347067	0.268438	0.113162	0.318722
RWDD1	-0.32229	-0.13201	-0.20469	-0.31081	-0.38528
RWDD3	0.244662	0.11728	0.366274	0.328073	0.409428
RWDD4A C4orf41	-0.17635	-0.28854	-0.30555	-0.09874	-0.2869
RXFP1	0.451026	0.102814	0.790207	0.293827	0.379014
RXFP2	0.038053	0.116753	-0.05025	-0.2734	-0.0641
RXFP3	-0.05678	-0.2762	0.235135	0.465643	-0.0039
RXFP4	0.408206	0.768452	0.621166	0.400926	0.31059
RXRA	-0.05334	0.081562	0.208189	0.061905	0.08428
RXRB SLC39A7	-0.46551	-0.48263	-0.49327	-0.20478	-0.39782
RXRG	0.213831	0.231831	-0.2133	0.050329	0.127312
RYBP	-0.24589	-0.33459	-0.31561	-0.24937	-0.41001
RYK	0.102795	0.122985	0.222261	0.070277	0.160415
RYR1	-0.1595	-0.2124	0.383488	0.143526	0.259261
RYR2	-0.04998	-0.11838	0.034343	-0.07536	0.069472
RYR3	-0.17131	-0.34927	-0.1946	-0.06605	-0.07582
S100A11	0.146793	0.274677	0.07721	0.544074	0.282686
S100A12	0.01189	-0.4079	-1.4537	-0.97655	-0.4196
S100A13	-0.08382	0.208861	0.32716	0.23296	0.328314
S100A13 C1orf77	0.161718	0.090084	0.07295	0.053451	0.074408
S100A14	0.231136	0.160694	0.292251	0.075535	0.248465
S100A16	-0.13974	-0.02664	0.037214	-0.02414	0.139972
S100A2	-0.50862	-0.34301	-0.22842	-0.27349	-0.31574
S100A5	0.150555	0.354911	0.349079	0.187501	0.36342
S100A6	0.492942	0.0343	0.215654	0.140918	-0.02162
S100A7	-0.75048	-1.37152	-1.76436	-1.5541	-1.08264
S100A7A	-0.59565	-1.14713	-1.54398	-1.21399	-0.89417
S100A8	-0.05152	-0.86934	-0.71271	-0.55436	-0.49647
S100A9	0.133587	-0.39559	-1.03614	-0.80058	0.184576
S100B	0.286323	0.563347	0.327162	0.518552	0.41769
S100P	0.510043	0.65762	0.820653	0.481799	0.728101
S100Z	0.383809	0.648758	0.725067	0.286963	0.504585
S1PR2	-0.19577	-0.13766	-0.17502	-0.14881	-0.22224
S1PR4	0.41262	0.400534	0.608394	0.435891	0.452305
S1PR5	-0.06958	-0.01838	-0.10507	0.059355	-0.01938
SAA1	0.413824	0.272229	0.518111	-0.19178	-0.32577
SAA2	0.41273	0.152831	0.57637	-0.08051	0.065533
SAA4	-0.00798	-0.64116	-0.51264	-0.58812	-0.39686
SAAL1	0.34498	-0.23637	0.016531	-0.01163	-0.03922
SACM1L	-0.57612	-0.33325	-0.72522	-0.40082	-0.46241
SACS	0.441365	0.794451	0.934179	0.404142	0.520949

SAE1	0.091327	0.330109	0.140755	-0.00305	0.067735
SAFB2 SAFB	-0.12542	-0.04296	0.046745	0.037228	-0.15305
SAG	0.470401	0.525452	0.696094	0.42639	0.586272
SAGE1	0.504675	0.584083	0.367937	0.529534	0.568556
SALL1	-0.49717	0.019829	0.328852	-0.19808	0.041336
SALL2	-0.17011	-0.5614	0.062049	-0.21392	-0.45161
SALL3	-0.08897	0.059574	0.241829	0.124834	0.208436
SALL4	0.129023	0.482114	0.151905	-0.4368	-0.03372
SAMD11	0.086163	0.220631	0.085603	0.000642	0.0929
SAMD12	-0.05329	0.141431	0.012443	-0.01054	-0.02266
SAMD13	0.166031	0.0469	0.02587	-0.03124	0.012538
SAMD14	-0.04274	-0.21471	-0.28492	-0.05105	0.018403
SAMD4A	-0.09558	-0.32449	-0.32942	-0.28982	-0.18766
SAMD4B	-0.32547	-0.25777	-0.40923	-0.16606	-0.26634
SAMD7	-0.64018	-0.25169	-0.6972	-0.46785	-0.58265
SAMD8	0.038355	-0.06394	0.140906	0.088142	0.181163
SAMD9	-0.38435	-0.44101	-0.72054	-0.56838	-0.76412
SAMD9L	0.075211	0.011004	-0.15683	-0.18082	-0.42572
SAMDC1	-0.21054	0.113715	0.53257	0.015879	-0.02232
SAMHD1	-0.04586	0.137334	0.142928	0.141481	-0.02418
SAMM50	-0.22591	0.098194	-0.29079	-0.13653	-0.44823
SAMSN1	0.064214	0.006821	-0.37972	0.100022	0.174287
SAP130	-0.11843	-0.18084	-0.1443	-0.05564	0.20305
SAP18	-0.16553	-0.12495	-0.1264	-0.11522	-0.40436
SAP30	-0.57093	-0.59553	-0.4027	-0.42167	-0.46332
SAP30L	-0.3572	-0.21938	-0.2492	-0.169	-0.35876
SAPS1	-0.3445	-0.26336	-0.4247	-0.23399	-0.36769
SAPS2	-0.18512	-0.12025	-0.06094	-0.16604	-0.14358
SAR1A	-0.19771	-0.32646	-0.19152	-0.17271	-0.17487
SAR1B	0.112419	0.126721	0.129882	0.134505	0.106513
SARDH	0.443268	0.492632	0.703018	0.211635	0.475935
SARS	0.154349	0.08959	0.146354	-0.22155	-0.19753
SARS2 MRPS12	-0.26765	-0.35192	-0.18886	-0.11879	-0.22691
SART1	0.337864	0.257312	0.004517	0.081012	0.118428
SART3 ISCU	-0.79905	-0.89818	-0.69771	-0.6712	-0.78364
SASH1	-0.52917	-0.26008	0.144789	-0.53137	0.226049
SASH3	0.312213	0.732994	0.152891	0.503619	0.506684
SASS6 CCDC76	-0.40925	-0.74126	-0.88354	-0.54937	-0.59739
SAT1	0.254607	0.049444	0.30709	-0.04253	0.035591
SAT2 SHBG	0.000735	0.09648	0.015778	0.023302	-0.03944
SATB1	-1.52784	-0.87844	-1.32994	-1.34614	-0.93585
SATB2	-0.72881	-0.90569	-1.02231	-0.48815	-1.02755
SATL1	0.270382	0.60549	-0.21139	-0.08211	0.212261
SAV1	-0.0674	-0.24764	-0.26	-0.2495	-0.37487
SBDS TYW1	-0.83343	-0.79229	-1.22924	-0.73896	-0.92863
SBF1	0.083098	-0.04764	-0.03647	0.020245	0.149669
SBF2	0.03261	0.09153	0.086454	-0.03948	0.027821
SBK1	-0.2186	-0.29889	-0.47753	-0.31505	-0.29264
SBNO1	0.40817	0.564151	0.645071	0.000955	0.316677
SBNO2	-0.31598	-0.43413	-0.49525	-0.46815	-0.39234

SBSN	0.274911	-0.84091	-0.70628	-0.41168	0.019519
SC22CB-5E3.3	0.224191	0.290608	0.203158	0.220016	0.278794
SC4MOL	-0.42538	-0.22834	-0.32922	-0.23237	-0.34031
SC5DL	-0.15465	0.047011	-0.41759	-0.27269	-0.37209
SCAMP1	0.013184	-0.16123	-0.14592	-0.0606	-0.04204
SCAMP2	0.133214	0.059839	-0.33743	-0.05608	-0.03119
SCAMP3 CLK2	-0.27271	-0.29798	-0.57676	-0.37992	-0.48253
SCAMP5	0.102395	-0.27099	-0.30189	-0.28689	-0.44636
SCAND1	-0.17335	-0.2742	-0.22322	-0.12037	-0.2114
SCAND2.	0.046355	-0.15321	-0.48653	-0.04374	-0.32191
SCAP	0.169814	0.405237	0.428569	0.252233	0.568069
SCAPER	-0.25559	-0.02214	-0.30788	-0.27268	-0.43423
SCARA3	0.165042	0.319377	0.332003	0.272619	0.411576
SCARA5	0.277109	0.253171	0.26949	0.060755	-0.11668
SCARB1	0.060549	0.180507	0.070658	0.138079	0.040068
SCARB2 AC034139.7	0.156162	0.085048	0.109315	0.074732	0.18913
SCARF1 RILP	0.157012	0.087626	0.548655	0.326731	0.349601
SCARF2	-0.05274	0.001651	-0.30557	-0.11688	-0.17037
SCCPDH	0.335822	0.267325	0.420817	0.217458	0.288619
SCD	-0.45795	-0.25695	-0.22079	-0.23376	-0.32649
SCD5	-0.04181	-0.04051	0.089802	0.041971	0.110296
SCEL	0.122953	0.049403	-0.06745	-0.24144	-0.0658
SCFD1	-0.05777	-0.65492	-0.97377	-0.22898	-1.26676
SCFD2	-0.39174	-0.50106	-0.7771	-0.61159	-0.8017
SCG2	-0.3026	-0.59449	-0.62868	-0.61718	-0.63265
SCG3	-0.72004	0.081463	-0.83401	-0.495	-0.35184
SCGB1A1	0.059542	0.413948	0.129022	-0.16954	0.262029
SCGB1C1	0.119197	-0.45266	-0.14155	-0.14738	-0.01689
SCGB1D1	-0.08793	-0.82933	-0.79295	-0.51074	-0.54549
SCGB1D2	-0.46224	-0.6174	-0.6789	-0.51151	-0.34724
SCGB1D4	-0.15647	-0.39302	-0.69857	-0.41793	-0.31211
SCGB2A1	0.327839	0.128778	0.210115	0.108264	-0.22445
SCGB2A2	0.253844	0.372746	0.227759	0.025631	0.166975
SCGB3A1	0.102603	0.051958	0.388284	0.234768	0.331779
SCGB3A2	-0.39655	-0.97447	-0.23972	-0.71026	-1.01805
SCGN	-0.54019	-0.70233	-0.94249	-0.45409	-0.50725
SCHIP1	-0.00405	-0.50599	-0.55928	-0.69976	-0.23123
SCIN	0.495928	0.56728	0.577766	0.328756	0.507134
SCLT1 C4orf33	-0.0702	-0.04909	0.019882	-0.16395	-0.17429
SCLY	-0.11724	-0.16413	-0.17583	-0.15834	0.011739
SCML1	0.276803	0.024369	0.292635	-0.20254	-0.18775
SCML2	0.065809	0.373058	0.345353	-0.11026	0.388246
SCML4	0.317056	0.271759	0.501112	0.270214	0.253963
SCN10A	0.285678	0.10279	0.438034	0.092271	-0.00628
SCN11A	0.306505	0.55365	0.399233	0.409515	0.257119
SCN1A	0.132358	0.085963	0.081852	-0.11129	-0.24853
SCN1B.	-0.05993	-0.00064	-0.15758	-0.08665	-0.14826
SCN1B. HPN	0.382143	0.361217	0.380971	0.340953	0.430393
SCN2A2	-0.3061	-0.13445	-0.42754	-0.22949	-0.29833
SCN2B	0.292699	0.69732	0.614457	-0.13057	0.097773

SCN3A	-0.30856	-0.27332	-0.59101	-0.51251	-0.14903
SCN3B	-0.14901	0.131091	0.005597	0.03452	-0.05995
SCN4A	0.258083	0.377797	0.17646	0.299978	0.261191
SCN4B	0.07973	0.289249	0.294753	0.115455	0.235465
SCN5A	-0.08053	0.093365	0.012381	-0.07827	-0.02513
SCN7A	-0.59779	-0.37291	-0.71522	-0.77063	-0.50481
SCN9A.	0.148899	0.366182	0.507588	0.016784	0.115983
SCNN1A	0.484405	0.440435	0.537937	0.156447	0.420292
SCNN1B	-0.15939	0.155652	0.26456	0.505732	0.025988
SCNN1D	0.253788	0.038368	0.165624	0.235036	0.360971
SCNN1G	0.203535	0.2052	0.302576	0.355908	0.160608
SCO1 C17orf48	-0.20898	-0.15158	-0.27646	-0.14217	-0.14983
SCO2 ECGF1	0.022532	0.089017	0.154115	0.117872	0.152444
SCOC	0.076383	-0.08	-0.15348	-0.20978	0.081299
SCOTIN	0.090017	0.336484	0.058071	0.16208	0.057248
SCPEP1	0.01463	0.088404	-0.25592	-0.18203	-0.03375
SCRIB	0.106469	-0.03701	0.198775	0.114825	0.198098
SCRN1	0.124121	0.09106	0.029219	0.160453	0.087144
SCRN2	0.064943	0.031171	-0.16695	-0.13518	-0.09734
SCRT1	-0.01484	-0.26113	-0.18734	-0.29515	-0.15862
SCRT2	-0.48386	-0.52112	-0.52861	-0.50392	-0.25513
SCTR	0.286229	0.704635	0.598387	0.465032	0.546483
SCUBE1	0.083907	0.141952	0.299092	0.261947	0.330145
SCUBE2	-0.09753	0.013817	0.133815	0.081458	0.183775
SCUBE3	-0.08566	0.082179	0.196072	-0.00867	0.002059
SCYL1	0.16197	0.234212	-0.31105	0.017706	0.164166
SCYL1BP1	-0.52124	-0.53868	-1.09496	-0.61696	-0.79268
SDAD1	0.148773	0.125275	0.154502	-0.14022	-0.07447
SDC1	0.157227	0.183852	0.295342	0.118589	0.072856
SDC2	-0.41715	-0.19828	-0.28592	-0.59447	-0.35025
SDC4	0.086295	0.111365	0.062619	0.195014	0.243597
SDCBP	-0.10222	-0.0161	-0.49056	-0.10718	-0.1424
SDCCAG1	-0.08332	-0.55656	-0.67114	-0.17615	-0.29866
SDCCAG3 PMPCA	-0.10457	-0.26886	-0.25341	-0.16314	-0.19696
SDCCAG8	-0.83858	-1.01906	-1.1315	-1.01175	-0.96965
SDF2 SUPT6H	-0.59942	-0.71788	-1.30006	-0.88663	-0.75292
SDF2L1	-0.15916	-0.19341	-0.38828	-0.17958	-0.04893
SDF4 B3GALT6	0.164568	0.215645	0.299358	0.059822	0.271299
SDHB	-0.36957	-0.50722	-0.59321	-0.05694	-0.12244
SDHC	0.197781	0.235869	0.153842	0.322491	0.22109
SDK1	0.055144	-0.13549	-0.02692	0.144268	0.204082
SDK2.	0.295122	0.63371	0.488984	0.331742	0.555427
SDPR	0.246203	0.575785	0.378232	0.272567	0.522607
SDS	-0.0646	-0.54107	-0.27943	-0.55524	-0.35957
SDSL	-0.05069	-0.08211	-0.19948	-0.19415	-0.18376
SEC11A	-0.21247	-0.28834	-0.39196	-0.14879	-0.35757
SEC11L3	-0.3073	-0.27565	-0.27716	-0.17328	0.008247
SEC13L1	0.020702	0.062918	0.146708	-0.0408	0.096076
SEC14L1	-0.06937	0.040275	0.086274	-0.04448	0.129383
SEC14L2	0.186489	0.073149	0.193158	0.063738	0.16504

SEC14L3	-0.06337	-0.07802	-0.34376	-0.37441	-0.35727
SEC14L4	0.26908	0.532959	0.456349	0.313228	0.463278
SEC16B	0.474247	0.226112	0.672464	0.358682	-0.17725
SEC22A	-0.13565	-0.20509	-0.1801	-0.23777	-0.19897
SEC22C	-0.35013	-0.05515	-0.27556	-0.35789	-0.17954
SEC23A	-0.02703	0.120304	-0.04017	-0.25884	-0.31873
SEC23B	-0.10809	0.042866	-0.32741	-0.19848	-0.2496
SEC23IP	-0.22676	-0.20057	-0.27126	-0.09302	-0.07708
SEC24B	-0.19564	-0.22805	-0.1133	-0.09698	-0.18542
SEC24C	-1.12183	-0.75082	-1.42772	-0.92515	-1.17181
SEC24C FUT11	0.0246	0.067252	0.0669	-0.00833	0.089235
SEC24D	-0.3277	-0.55026	-0.44388	-0.4408	-0.39284
SEC31A	-0.41782	-0.51681	-0.53371	-0.39372	-0.42037
SEC31A THAP9	-0.89526	-0.8113	-0.70916	-0.80743	-0.77805
SEC31B	0.330115	0.497516	0.416387	0.418458	0.149302
SEC31B NDUFB8	-0.32055	-0.19603	-0.42786	-0.57047	-0.47277
SEC61A1	-0.47467	-0.4121	-0.4892	-0.3006	-0.34333
SEC61A2	0.071777	0.226156	0.072217	0.103807	0.20866
SEC61G	-0.22107	-0.55792	-0.85126	-0.08535	-0.4024
SEC62	0.098117	0.432805	0.055892	0.056863	-0.10355
SEC63	-0.02985	-0.23264	-0.10051	-0.25228	-0.13115
SECISBP2	0.255442	0.024605	0.21041	0.114672	0.135859
SECTM1	0.156354	0.201897	0.471708	0.173155	0.4585
SEDLP. OFD1	-0.05363	-0.00099	-0.24457	-0.20975	-0.22994
SEH1L	-0.5506	-0.32177	-0.51805	-0.44236	-0.38159
SEL1L	0.036044	0.060779	0.121834	0.070754	0.024307
SELE	0.438438	-1.04864	-0.86754	-0.52838	-0.62299
SELENBP1	0.147243	0.125133	0.644754	0.219876	0.132777
SELL	-0.63951	-1.33821	-1.18225	-1.18563	-0.99678
SELM	0.064118	0.293891	0.063269	0.174438	0.223676
SELP	0.236825	-1.08239	-0.89758	-0.91477	-0.85574
SELPLG	0.407265	0.661914	0.589291	0.485774	0.618043
SEMA3A	-0.7609	-0.62479	-1.24915	-0.55271	-0.74171
SEMA3B	0.205735	0.304911	0.368308	-0.01001	0.266817
SEMA3C	0.263791	0.504707	0.37609	0.386787	0.427434
SEMA3D	0.137824	0.287387	0.073498	-0.23467	-0.12928
SEMA3E	-0.0558	-0.30886	0.769107	-0.50135	-0.77602
SEMA3F	-0.00989	0.242681	0.255593	0.10154	0.100467
SEMA3G	-0.05451	0.197115	0.007011	0.126677	0.029333
SEMA4A	0.078203	0.480153	0.429492	0.254208	0.416762
SEMA4C	-0.02336	-0.00487	0.002094	0.005058	0.111386
SEMA4F	0.229602	0.272547	0.093928	0.145132	0.170187
SEMA4G	0.313463	0.443635	0.442643	0.11707	0.205826
SEMA5A	-0.07727	0.264065	0.112728	0.258869	0.254349
SEMA5B	-0.21779	-0.30518	-0.29305	-0.197	-0.26451
SEMA6A	-0.42482	-0.24343	-0.27665	-0.24275	-0.2405
SEMA6B	0.240213	-0.08638	0.030361	0.213021	0.180136
SEMA6C	-0.41177	0.032043	-0.64296	-0.16847	-0.07034
SEMA6D	-0.29825	0.053846	-0.16549	-0.0514	-0.11404
SEMA7A	0.05791	0.191778	0.46319	0.078369	0.38476

SEMG1	-0.72382	-1.00123	-0.82324	-0.74362	-0.92184
SEMG2	-0.17923	-0.97342	-0.9995	-0.61856	-0.8891
SENP1	-0.50282	-0.54252	-0.78536	-0.48474	-0.67919
SENP2	0.055336	-0.03868	-0.34791	-0.11947	-0.26109
SENP5	0.312256	0.134679	-0.0329	-0.00536	-0.03207
SENP6	-0.31425	-0.22548	0.551013	-0.17478	-0.2989
SENP7	0.212289	-0.27876	-0.39493	-0.42442	-0.57433
SEPHS1	-0.32927	-0.26924	-0.22136	-0.24948	-0.31653
SEPN1	-0.00753	-0.1953	-0.31628	-0.10867	-0.00601
SEPSECS	-0.20603	-0.19255	-0.51294	-0.28052	-0.21238
SEPT10 ANKRD57	0.004724	0.01429	0.214925	0.197482	0.159958
SEPT3 WBP2NL	0.350419	0.312915	0.155002	0.352293	0.141727
SEPW1	0.070233	0.07135	-0.04838	0.125579	0.134385
SEPX1	-0.1523	0.084128	-0.23637	-0.20662	-0.03572
SERAC1 GTF2H5	0.021625	0.153345	-0.00885	-0.01956	0.134921
SERBP1	-0.38746	-0.31057	-0.23314	-0.37473	-0.10624
SERF1A	-0.51481	-0.48678	-0.8882	-0.48188	-0.61779
SERGEF	-0.0416	-0.20002	-0.00331	-0.10731	-0.09475
SERHL	0.13946	0.14991	0.263022	0.09074	0.201711
SERHL2	0.204717	0.344984	0.312934	0.27085	0.10398
SERINC1	-0.58999	-0.26861	-0.71053	-0.70108	-0.7684
SERINC2	0.196024	0.132629	0.223153	0.177177	0.159807
SERINC3	-0.31952	-0.26643	-0.37265	-0.38144	-0.34966
SERP1 EIF2A	-0.51514	-0.58483	-0.47253	-0.46856	-0.53159
SERP2	0.007115	0.1374	0.030546	0.108316	0.332731
SERPINA1	0.204724	0.292613	-0.20019	0.022013	0.079574
SERPINA10	0.133801	-0.01669	-0.6812	-0.43613	-0.5468
SERPINA12	-0.12226	-0.1866	-0.49829	-0.35785	-0.5495
SERPINA13.	0.232633	0.242551	0.061377	0.197996	0.099084
SERPINA3	0.358097	-0.40183	-0.25489	-0.22211	-0.52165
SERPINA4	0.10518	-0.23665	-0.35002	-0.11128	-0.48563
SERPINA5	0.253661	0.219754	-0.618	0.37858	0.110629
SERPINA6	0.441916	-0.04606	-0.98607	-0.47811	-0.82697
SERPINA7	-0.5864	-0.54764	-0.86747	-0.41173	-0.6675
SERPINA9	-0.68636	-1.46623	-1.66123	-1.24935	-1.85662
SERPINB1	0.304869	-0.03161	0.142044	0.325734	0.392727
SERPINB10	0.086138	-0.49978	-0.30741	-0.41653	-0.11737
SERPINB11.	0.322795	0.034453	-0.34871	-0.1315	-0.0464
SERPINB12	-0.10842	-0.57627	-0.51617	-0.69693	-0.51914
SERPINB13	0.630637	0.41889	0.140854	0.093796	0.234792
SERPINB2	0.102798	-0.3599	-0.5177	-0.68642	-0.65674
SERPINB3	-0.35054	-0.5987	-0.49634	-0.49618	-0.4878
SERPINB4	-0.17259	-0.43403	-0.4395	-0.40081	-0.42999
SERPINB5	0.588019	0.72227	0.813261	0.610279	0.728693
SERPINB6	0.108487	0.143723	0.066797	0.167472	0.073508
SERPINB7	-0.95971	-1.04296	-0.79612	-0.86664	-0.43552
SERPINB8	0.246167	0.312977	0.446678	0.083029	0.142364
SERPINB9	0.195807	0.186496	0.16962	0.288426	0.384294
SERPINC1	0.377047	0.633427	0.540406	0.2019	0.325221
SERPINE1	-0.45666	-0.34409	-0.22857	-0.45026	-0.04446

SERPINE2	-0.09883	-0.16398	0.339241	0.009913	0.110126
SERPINF1	0.530167	0.219678	0.52871	0.356109	0.482929
SERPINF2	0.414137	0.576042	0.655663	0.544586	0.675646
SERPING1	0.048383	0.270473	0.217469	0.066389	0.310002
SERPINH1	0.017513	-0.11106	-0.40649	-0.30817	-0.04264
SERPINI2	-0.43344	-0.77269	-0.69021	-0.66599	-0.75462
SERTAD1	-0.0407	0.015255	-0.12881	-0.10118	-0.01215
SERTAD2	-0.14172	-0.14805	-0.08511	-0.17814	-0.11687
SERTAD3	0.045278	0.117966	0.128933	-0.06236	-0.01626
SESN1	-1.24978	-0.83623	-1.38383	-0.94147	-1.01109
SESN2	0.013104	-0.22777	-0.25949	-0.08631	-0.0286
SESN3	-0.70357	-0.33967	-0.63158	-0.27468	-0.36265
SET	-0.02003	0.128316	0.081115	0.009097	-0.05515
SETBP1	0.523944	0.796116	0.675287	0.46334	0.554058
SETD1A	0.089155	0.271117	0.13464	0.137425	0.258395
SETD1A HSD3B7	-0.00391	0.410828	0.19381	0.190438	0.045353
SETD2	0.545171	0.450485	0.504353	0.376011	0.48841
SETD3 CCNK	-0.40153	-0.40553	-0.36628	-0.40607	-0.34228
SETD6	0.272575	-0.07187	-0.11564	-0.20508	-0.09173
SETD7	-0.17222	-0.16212	-0.17554	-0.11098	-0.18447
SETD8	-0.18837	-0.11974	-0.20908	0.143112	0.080943
SETDB1	0.165683	-0.03296	0.135273	-0.03662	0.233031
SETX	0.019294	0.183118	0.221394	0.140674	0.027378
SEZ6	-0.14978	0.015914	-0.25194	-0.17223	-0.07974
SEZ6L	-0.05778	0.170794	-0.03582	-0.21152	-0.01831
SEZ6L2 ASPHD1	-0.38958	-0.57313	-0.38768	-0.50431	-0.59536
SF1	-0.33637	-0.26877	-0.26941	-0.28575	-0.07532
SF3A1 AC004997.2	-0.05411	0.076351	-0.11077	0.090347	-0.11496
SF3A2 AMH	0.080054	0.137335	0.292246	0.110745	0.32173
SF3A3	0.108107	0.011227	0.206811	0.096622	-0.04807
SF3B1	-0.3882	-0.57506	-0.77907	-0.59417	-0.66618
SF3B2	0.077354	-0.14715	-0.14233	-0.06309	-0.06251
SF3B4	-0.41244	-0.63016	-0.54844	-0.58905	-0.57173
SF3B5	-0.39818	-0.24559	-0.23746	-0.19499	-0.30571
SF4 KIAA0892	-0.22806	-0.29685	-0.40547	-0.18741	-0.25948
SFMBT1	0.111141	0.156554	0.079304	0.110475	0.309202
SFMBT2	-0.03853	0.493705	0.114561	0.156595	-0.20413
SFN	0.273068	0.337208	0.452263	0.299309	0.266547
SFPQ	-0.06203	-0.16286	-0.20316	-0.06663	-0.00865
SFRP1	0.017851	0.221596	0.221589	0.117164	0.29536
SFRP2	-0.0431	0.282746	0.344711	0.383908	0.320349
SFRP4	-0.14808	-0.15853	-0.14111	-0.13607	-0.00294
SFRP5	0.183449	0.316011	0.437363	0.290569	0.24056
SFRS1	-0.74742	-0.65256	-1.21656	-1.01967	-0.81682
SFRS10	0.115506	0.071162	-0.03541	0.005473	0.039788
SFRS12IP1 SDCCAG10	-0.81633	-0.7821	-1.19089	-0.68203	-0.99174
SFRS14 ARMC6	-0.09048	0.032628	-0.13528	-0.20082	-0.21846
SFRS15	-0.1192	-0.1552	-0.38396	-0.20294	-0.34141
SFRS16	0.256484	0.000887	0.166807	0.237643	-0.01881
SFRS18	-0.38665	-0.09114	-0.48488	-0.48609	-0.48659

SFRS2 MFSD11	-0.16415	-0.41565	-0.23652	-0.31326	-0.03901
SFRS2IP	0.239397	0.217222	-0.0098	-0.03841	-0.01154
SFRS3	-0.43675	-0.38407	-0.64717	-0.34029	-0.35295
SFRS4	0.049185	0.150631	0.096477	0.108953	0.218353
SFRS5	-0.10994	-0.14041	-0.21918	-0.10712	-0.18653
SFRS6	-0.1533	0.076649	-0.04136	-0.10676	0.016208
SFRS7	-1.03126	-0.78117	-1.09426	-0.54968	-0.95679
SFRS8	-0.1287	-0.23908	-0.11913	-0.0338	-0.15941
SFRS9 DYNLL1	0.157501	0.237824	0.133521	0.077827	0.118239
SFT2D1	0.124237	0.105198	-0.07679	0.070479	-0.0695
SFT2D2	0.046539	-0.19759	-0.21819	-0.02531	0.126518
SFT2D3	0.185093	0.066716	0.391909	0.347428	0.193421
SFTPA1B	0.082954	-0.19645	-0.09301	-0.04763	-0.16144
SFTPA2B	-0.10921	-0.15186	-0.26369	-0.2196	-0.56696
SFTPB	0.073306	0.187271	0.284923	-0.05316	0.151926
SFTPC	-0.14608	0.225122	0.074641	-0.12877	0.171923
SFXN1	0.04463	-0.04725	-0.06119	-0.0629	0.034997
SFXN4	0.263679	0.393495	0.165123	0.273892	0.055851
SFXN5	-0.02085	0.022298	-0.34911	0.076863	0.325914
SGCB	0.316319	-0.23608	0.280533	0.440477	-0.03853
SGCD	-0.06208	-0.47973	0.016063	-0.32482	-0.63444
SGCE PEG10.	-0.12708	-0.10507	-0.03211	-0.17563	-0.17103
SGCG	0.400681	0.101122	-0.51793	-0.39285	-0.37267
SGIP1	0.093546	-0.27366	-1.04572	-0.13362	-0.52287
SGK1	-0.07313	0.020522	-0.09122	0.019361	-0.1314
SGMS1	-0.01964	-0.11601	0.035438	0.007628	0.052477
SGMS2	0.372412	-1.15228	-0.03658	-0.19317	-0.70144
SGOL1	-0.99605	-0.66752	-1.02434	-0.82785	-0.59269
SGOL2	-0.15834	-0.38868	-0.61049	-0.34015	-0.50715
SGPL1	-0.22495	0.146397	-0.27126	-0.43246	-0.2515
SGPP1	0.138022	0.181913	0.097399	0.08031	0.145869
SGPP2	0.181624	0.601093	0.632787	0.520101	0.588284
SGSH SLC26A11	-0.0414	-0.12289	-0.18098	-0.01499	0.058601
SGSM1.	0.248237	0.431494	0.404962	0.358397	0.278084
SGSM3	0.186293	0.392964	0.385346	0.246607	0.159723
SGTA THOP1	0.219192	0.231321	0.110538	0.194536	0.236456
SGTB NLN	-0.28441	-0.20664	-0.18327	-0.24081	-0.17959
SH2B1	-0.21289	-0.20551	-0.42704	-0.11924	-0.14935
SH2B2.	0.240145	0.252938	0.079059	0.136379	-0.11697
SH2B3	0.022946	0.159398	0.104717	0.077553	0.065109
SH2D1A	-0.09602	0.072663	-0.90002	0.059413	-0.21368
SH2D1B	0.164346	-0.71516	0.072681	-0.63458	-0.50819
SH2D2A NTRK1	-0.05818	0.054651	0.106906	0.096563	0.295567
SH2D3A	-0.21052	-0.03282	0.465765	0.406896	0.320639
SH2D4A	0.180705	0.149241	0.318458	0.276941	0.381348
SH2D4B	0.16735	-0.11431	-0.21212	0.161986	-0.15344
SH2D6	0.14491	0.163782	0.086325	0.103565	0.23912
SH3BGR	0.270674	0.340749	0.42959	0.219706	-0.31982
SH3BGRL3	0.285489	0.216445	0.320027	0.246359	0.101405
SH3BP2	0.028401	-0.14283	-0.09886	0.011675	-0.07541

SH3BP4	0.028105	0.049308	-0.04401	-0.02069	-0.02511
SH3BP5	0.153724	0.287627	0.304109	0.203672	0.23584
SH3BP5L	-0.43749	-0.63036	-0.76816	-0.66455	-0.74078
SH3D19	0.186383	0.06868	0.204492	-0.23698	-0.12853
SH3GL1 CHAF1A	0.070632	-0.08956	-0.10755	0.020494	0.011721
SH3GL2	0.160907	0.229019	0.30744	0.293011	0.324065
SH3GL3	0.139011	0.056086	0.056958	0.191012	0.161501
SH3GLB1	-0.23455	-0.00453	-0.09385	-0.10175	0.041809
SH3GLB2	-0.0432	-0.23915	-0.18375	-0.27222	-0.09388
SH3PXD2A	-0.24723	-0.24279	-0.36792	-0.27635	-0.28189
SH3PXD2B	0.108735	0.18019	0.08214	-0.08923	0.052018
SH3RF1	-0.03454	0.189554	0.132161	0.068459	0.190704
SH3RF2	-0.50083	0.868527	0.706182	0.529717	0.286079
SH3TC1	0.361974	-0.00296	0.424048	0.426583	0.273211
SH3TC2	-0.11885	-0.22901	-0.00387	-0.19196	-0.52752
SH3YL1 ACP1	-0.05729	0.22962	0.29409	0.232821	0.328578
SHANK1	0.310608	0.059362	0.203112	0.285708	0.337064
SHARPIN MAF1	0.023332	-0.01623	0.147687	-0.00947	0.138934
SHC1	-0.29458	-0.49392	-0.41237	-0.06149	-0.13071
SHC1 CKS1B	-0.90874	-1.22472	-1.07319	-0.73501	-0.71229
SHC3	-0.03508	0.295306	0.441765	0.016248	-0.27325
SHCBP1	-0.40853	-0.27124	-0.60028	-0.53211	-0.56503
SHD	-0.05353	-0.13548	-0.19984	-0.0983	-0.04222
SHE TDRD10	0.122324	0.487039	0.548637	0.345173	0.34511
SHF	-0.08476	-0.04941	-0.16075	-0.14125	-0.19722
SHFM1	0.007512	0.237181	-0.0112	-0.27263	-0.02134
SHH	-0.71712	-0.47762	-0.10988	0.4603	-0.14987
SHMT1	0.140924	0.188782	0.16129	0.036806	0.151822
SHMT2	-0.72403	-0.43537	-0.54255	-0.68521	-0.7622
SHOC2	-0.16262	-0.11708	-0.21418	-0.50399	-0.48385
SHOX	0.024937	0.132672	0.454955	0.307196	0.348959
SHPRH	-0.21362	0.008498	-0.28321	-0.2303	-0.06939
SHQ1	-0.97027	-0.80105	-1.20877	-0.92559	-1.28517
SHROOM1	0.328805	0.134471	0.277157	0.135334	0.255798
SHROOM2	0.224625	0.313614	0.391892	0.256738	0.339955
SHROOM3	-0.25889	0.298158	0.582473	0.013967	-0.52054
SHROOM4	0.085986	0.205761	0.302973	0.144598	0.293688
SI	-0.52274	-0.42903	-0.46664	-0.47844	-0.61185
SIAE SPA17	0.374795	0.174334	-0.47012	0.393874	0.440712
SIAH2	-0.27774	-0.20267	-0.42273	-0.27268	-0.39244
SIDT2	-0.38347	-0.2243	-0.44706	-0.08983	-0.46784
SIGIRR TMEM16J	0.046197	0.15328	0.1836	0.16267	0.124765
SIGLEC1	0.33625	0.165231	0.45594	0.125134	0.306245
SIGLEC10	-0.21227	-0.26859	-0.1023	-0.15485	0.130613
SIGLEC11	0.325948	-0.62516	-0.76641	-0.29881	-0.04938
SIGLEC12	0.050595	-0.63668	-0.51119	-0.56107	-0.27851
SIGLEC15	-0.09041	-0.03378	-0.13798	-0.16678	-0.2882
SIGLEC6	-0.15413	-0.66226	-0.50148	-0.37604	-0.51331
SIGLEC7	0.471058	-0.27765	-0.28653	-0.18364	0.380459
SIGLEC8	0.099976	-0.28986	-0.48149	-0.27825	-0.09504

SIGLEC9	0.339958	-0.46553	-0.6267	-0.50155	0.135782
SIL1	0.036306	0.308613	0.161591	0.010597	0.054279
SILV CDK2	-0.78993	-0.91011	-1.21751	-0.97589	-1.00728
SIM1	-0.59379	-0.22941	0.123517	0.091656	0.449268
SIM2	0.087084	0.15669	0.277042	0.255984	0.155571
SIN3A	-0.5116	-0.52959	-0.46053	-0.37351	-0.34894
SIN3B	0.488811	0.387038	0.673857	0.343978	0.441317
SIP1	-0.24203	-0.15951	0.028603	-0.17782	-0.31129
SIPA1L1	0.541012	0.430792	0.363237	0.236735	0.303657
SIPA1L2	0.789934	1.266287	1.086807	0.532254	0.764816
SIRPA	0.196164	0.246272	0.212323	0.142396	0.176985
SIRPB1	4.88E-06	0.149385	0.067632	-0.38747	-0.16454
SIRPD	-0.14171	0.121894	-0.24808	-0.46981	-0.19145
SIRPG	-0.25835	0.071915	-0.09655	-1.00882	-0.7093
SIRT1	0.067512	0.081548	0.217587	-0.26646	-0.12674
SIRT2 NFKBIB.	-0.5004	-0.33201	-0.66346	-0.67764	-0.62924
SIRT3 PSMD13	-0.26096	-0.31305	-0.32228	-0.25393	-0.10305
SIRT4	0.110561	-0.16493	-0.49199	-0.27106	-0.51176
SIRT5	-0.11127	-0.13872	-0.11889	-0.05134	0.220526
SIRT6	0.099455	0.041914	-0.08403	-0.03076	-0.12737
SIRT7 MAFG	0.329274	0.021585	-0.01125	0.139999	0.07511
SIT1	0.23219	0.431127	0.454213	0.067741	0.350885
SIVA1	0.188789	-0.00936	-0.00324	0.200444	0.419543
SIX1	-0.3373	-0.18084	0.033661	-0.2179	-0.15734
SIX2	-0.14124	0.008293	0.109735	0.000917	0.110244
SIX3	-0.24303	0.3578	0.06857	0.049166	0.160135
SIX4	-0.43656	-0.20719	-0.29105	-0.33049	-0.36238
SIX5	-0.33952	-0.24388	-0.36819	-0.33226	-0.36887
SIX6	-0.36953	0.307076	-0.27984	0.242712	0.15863
SKAP1	0.239124	0.107209	0.407781	0.259276	0.40975
SKAP2	-0.60451	-0.33656	-1.18371	0.509873	-0.81012
SKI	-0.07677	0.004857	0.1025	0.014727	0.085973
SKIP	-0.08689	-0.01661	-0.00527	0.054654	0.053296
SKP1A	0.038925	0.105083	-0.01987	0.038433	-0.08279
SLAIN1	0.371028	0.649827	0.711753	0.357027	0.884725
SLAMF1	0.287308	-0.68745	-0.58441	-0.64745	-0.38172
SLAMF6	0.567041	-0.41276	-0.6472	-0.42344	-0.08728
SLAMF7	-0.72081	-1.14861	-1.16112	-1.04987	-0.98287
SLAMF8	0.136616	0.442269	0.290615	0.208297	0.309994
SLAMF9	0.290623	0.288437	0.327157	0.043011	0.121487
SLBP	0.099536	0.166525	0.054894	0.097968	0.247189
SLC10A1	0.231089	0.302207	0.101761	0.058589	0.075788
SLC10A2	-0.18188	0.461947	0.61787	0.153324	-0.40384
SLC10A3	0.202187	0.296128	0.378427	0.072492	0.174096
SLC10A4	0.10562	-0.13266	-0.26887	0.356478	-0.36048
SLC10A5	0.333102	0.222709	0.020815	-0.00535	-0.18596
SLC10A6	0.442007	0.458858	0.287596	0.20933	0.234584
SLC10A7	-0.63171	-0.73642	-0.50371	-0.33367	-0.48602
SLC11A1	0.269126	0.565973	0.46264	0.202078	0.340462
SLC11A2	0.068738	0.101207	-0.00604	0.139298	0.047127

SLC12A1	-0.20491	-0.10644	0.055586	-0.16204	-0.12769
SLC12A2	0.094255	0.142269	0.153069	0.079872	0.128287
SLC12A3	0.212482	0.449967	0.48807	0.310037	0.146343
SLC12A4	-0.10382	0.01012	-0.04747	0.040124	0.200135
SLC12A5	0.228728	0.285776	0.500149	0.253137	0.108125
SLC12A6	0.616781	0.904195	0.9817	0.601675	0.378692
SLC12A7	0.0278	-0.00577	0.108795	0.081209	0.080345
SLC12A9	-0.14337	-0.13535	-0.52327	-0.23085	-0.26694
SLC12A9 TRIP6	0.062909	-0.32428	0.541826	0.453304	0.426365
SLC13A1	-0.46319	-0.11496	-0.08821	-0.59794	-0.31776
SLC13A2	0.136295	0.422804	0.528594	-0.05299	0.465766
SLC13A3	0.303675	0.245924	0.235545	0.221815	0.380227
SLC13A4	0.425517	0.842327	0.778981	0.552097	0.869241
SLC13A5	-0.1007	0.077915	0.364641	0.200564	0.260004
SLC14A1	0.220216	-0.02714	0.095566	-0.40659	-0.16491
SLC15A1	0.111729	0.094458	-0.04293	0.097548	0.132387
SLC15A2	0.236878	0.694245	0.366538	0.315237	0.148742
SLC15A3	0.072155	0.221518	0.349157	0.138034	0.134952
SLC15A4	0.141601	0.091033	0.270061	0.129235	0.156993
SLC16A1	-0.12496	-0.15975	-0.07008	-0.33225	-0.37375
SLC16A10	-0.07061	-0.00712	-0.21096	-0.27535	-0.12354
SLC16A11	0.033246	-0.27903	-0.06591	-0.01527	-0.20681
SLC16A12	0.177298	0.26338	0.301158	0.256095	0.315584
SLC16A13	-0.21806	-0.06977	-0.31377	0.056923	0.02614
SLC16A14	0.424348	0.397815	0.227646	0.084432	0.112163
SLC16A2	0.003291	0.141282	0.070243	0.065047	0.022318
SLC16A3	-0.10558	-0.05892	0.323831	0.131212	0.212519
SLC16A4	0.350256	0.334326	0.057765	-0.33443	0.128974
SLC16A5	0.310509	0.343807	0.474919	0.438577	0.319931
SLC16A6	-0.14867	0.009886	-0.00129	-0.25598	-0.02299
SLC16A7	-0.2355	-0.22277	-0.4045	-0.3035	-0.41922
SLC16A8	0.139492	0.329312	0.426002	0.330404	0.432363
SLC16A9	0.447785	0.273335	0.445467	0.178293	0.115371
SLC17A1	0.418234	0.751025	0.778233	0.265898	0.308288
SLC17A2	0.781645	0.778009	0.908829	0.417085	0.315006
SLC17A3	0.178308	0.369285	0.509054	0.212635	0.173751
SLC17A4	-1.31354	-0.82531	-0.76741	-1.06998	-1.6657
SLC17A5	0.223468	0.205175	0.125168	0.127549	0.130128
SLC17A6	-0.59928	0.922513	0.882256	0.741172	0.684551
SLC17A7	0.078282	-0.44111	-1.05955	-0.7341	-0.6069
SLC17A8	-0.06504	0.321796	0.339616	-0.03959	-0.0082
SLC18A1	-0.18457	-0.14049	-0.2913	-0.27047	-0.52651
SLC18A2	0.042102	0.227095	0.239717	0.19198	0.24081
SLC19A1	0.074828	0.06961	0.037615	-0.05856	-0.00881
SLC19A2 Z99572.1	-0.68234	-0.19785	-0.71211	-0.31676	-0.20675
SLC19A3	0.262181	0.379908	0.125865	0.151793	0.096856
SLC1A1	-0.02765	0.134532	-0.26957	-0.13887	0.106624
SLC1A2	-0.37055	0.110275	-0.32625	-0.20971	0.145305
SLC1A3	-1.15802	-0.75907	-0.92382	-0.85827	0.1856
SLC1A4	0.155159	0.200147	0.122125	0.222622	0.089084

SLC1A5	-0.15961	-0.07373	0.059589	-0.25303	-0.13604
SLC1A6	0.265744	-0.41494	0.038748	0.001028	0.380046
SLC1A7	0.183312	0.404549	0.303454	0.126168	0.236723
SLC20A1	-0.19218	-0.15392	-0.18825	-0.17792	-0.00075
SLC20A2 C8orf40	0.090804	0.045086	0.189737	-0.0627	0.120085
SLC22A1	0.337439	-0.14545	0.630674	0.32821	0.49124
SLC22A11	0.541375	0.628291	0.651069	0.534467	0.619497
SLC22A12	0.199836	0.279018	0.245136	0.080613	-0.0182
SLC22A13	0.124228	0.093432	-0.063	-0.3204	0.085288
SLC22A14	0.262725	0.382435	0.475117	0.107748	0.262765
SLC22A15	0.186502	0.211147	0.26886	0.190084	0.313136
SLC22A16	0.330646	0.186449	0.278033	0.20744	0.278983
SLC22A17	-0.06307	0.068015	-0.09921	-0.14439	-0.14925
SLC22A2	0.493627	-0.18094	-0.0067	0.255917	0.498625
SLC22A23	0.192155	-0.33822	-0.13728	0.009489	-0.09917
SLC22A24.	-0.25564	-0.45567	-0.27705	-0.41622	-0.35067
SLC22A25	-0.03045	-0.80883	-0.98555	-0.85911	-0.23496
SLC22A3	0.122905	0.215893	0.620126	0.307749	0.464947
SLC22A4	-0.25691	-0.17715	-0.22347	-0.1472	-0.24684
SLC22A5	0.320259	-0.05371	-0.35637	-0.21207	-0.15202
SLC22A6	-0.01172	0.094062	0.242296	0.191842	0.240156
SLC22A7	0.087309	-0.02237	0.089601	0.08354	0.07818
SLC22A8	0.336279	0.508843	0.561439	0.419931	0.450287
SLC22A9	0.170734	-0.47279	-0.40049	-0.53352	-0.02366
SLC23A1	0.327111	0.089704	-0.13742	0.276336	-0.18979
SLC24A2	0.21733	0.025798	0.456019	0.027423	-0.33894
SLC24A3	0.061935	0.145001	0.170959	0.066666	0.060116
SLC24A4	-0.25728	-0.47814	-0.26256	0.067205	0.018359
SLC24A5	0.467745	0.518249	0.571235	0.373448	0.326476
SLC24A6	0.330444	0.357535	0.33819	0.222448	0.311074
SLC25A1	-0.11045	-0.23872	-0.23114	-0.08395	-0.07951
SLC25A10	0.173957	0.128183	0.039378	0.064054	0.070543
SLC25A11 RNF167	-0.53751	-0.22736	-0.39317	-0.39131	-0.38566
SLC25A12	0.294096	0.421348	0.353229	0.268827	0.160474
SLC25A13	0.097345	0.083631	0.023151	-0.03649	0.150352
SLC25A14	0.441874	0.02665	0.371799	-0.05963	-0.12193
SLC25A15	0.132247	0.254529	0.188334	0.171671	0.130659
SLC25A16	0.281497	0.116344	0.010446	0.213182	0.096529
SLC25A17	-0.07947	-0.12677	-0.27961	-0.2514	-0.49973
SLC25A18	0.34106	0.595373	0.87127	0.503514	0.51133
SLC25A19	-0.19256	-0.15989	-0.22056	-0.42552	-0.17707
SLC25A2	0.704541	0.656402	0.705533	0.560508	0.734098
SLC25A20	0.267568	0.198019	0.043219	0.026795	0.080218
SLC25A21	-0.09845	-0.02567	0.468669	0.308557	0.20365
SLC25A22	0.035245	-0.39221	-0.40682	-0.1432	0.057224
SLC25A23	-0.24773	-0.14739	-0.26719	-0.16952	-0.12637
SLC25A26	-0.20202	-0.09697	-0.11047	-0.24981	-0.1916
SLC25A28	-0.33824	-0.14226	-0.1472	-0.1794	-0.05875
SLC25A29	-0.22233	-0.08297	-0.16531	-0.20286	-0.09169
SLC25A3	-0.01432	-0.02459	-0.16571	-0.20624	-0.23752

SLC25A30	-0.12689	-0.16951	-0.46026	-0.28071	-0.25515
SLC25A31	0.531988	0.674452	0.915733	0.522421	0.588861
SLC25A32 WDSOF1	-0.54497	-0.53854	-0.74216	-0.57787	-0.67332
SLC25A33	-0.05751	0.068682	0.06102	0.017545	0.036105
SLC25A34	0.328643	0.261715	0.468824	0.33947	0.3661
SLC25A36	0.063354	-0.16389	-0.31674	-0.17884	-0.25344
SLC25A37	-0.07583	0.092479	0.036681	0.091345	0.076535
SLC25A38	0.16648	0.406223	0.05232	-0.06262	0.057662
SLC25A39	0.073969	0.187222	0.078903	-0.00505	-0.13154
SLC25A4	0.266069	-0.07946	-0.19682	-0.07513	-0.06955
SLC25A40 DBF4	-0.287	-0.31258	-0.4763	-0.25754	-0.11599
SLC25A41	0.277723	0.393406	0.490247	0.08855	0.183355
SLC25A42	0.131351	0.207939	-0.01053	0.282985	0.152664
SLC25A43	0.193819	-0.04269	-0.2454	0.043869	-0.02516
SLC25A44	0.175509	-0.42765	-0.37098	0.001322	-0.16005
SLC25A44 PMF1	0.02315	0.112576	-0.08022	-0.00052	-0.15774
SLC25A45	-0.11436	-0.08365	-0.25774	-0.09494	0.050655
SLC25A46	-0.5395	-0.8166	-0.96307	-0.54387	-0.79132
SLC25A5	0.099137	0.134358	0.103407	-0.02127	0.065524
SLC25A6	-0.00196	0.018135	0.047384	-0.02952	0.037822
SLC26	0.033226	0.125462	0.229314	0.006964	0.206253
SLC26A1	0.096541	0.06133	0.023983	-0.006	0.153466
SLC26A1 IDUA	0.317361	0.21917	0.296999	0.306226	0.40064
SLC26A3	-0.46945	-0.41822	-0.7539	-0.6572	-0.68882
SLC26A4	0.110949	0.56608	0.645471	0.157196	0.329149
SLC26A5	0.281119	0.467637	0.646874	0.255931	0.30394
SLC26A7	-0.50573	-0.32094	-0.57718	-0.78365	-0.68746
SLC26A8	0.395864	0.679763	0.701682	0.379317	0.560716
SLC26A9	0.261858	-0.1598	0.234992	0.048619	0.017129
SLC27A1	-0.82226	-0.57753	-0.95347	-0.63038	-0.70803
SLC27A2	0.313458	0.454415	0.390647	0.398595	0.151236
SLC27A3	0.344177	-0.148	-0.10035	0.362306	-0.06387
SLC27A4	0.159894	0.146437	0.192254	0.183837	-0.02954
SLC27A5	0.322438	0.208792	0.315609	0.15612	0.187347
SLC27A6	0.017299	-0.16072	0.151945	0.296764	-0.00273
SLC28A1	0.390056	0.535763	0.574958	0.310953	0.409864
SLC28A2	0.735414	0.822589	0.860626	0.635286	0.857036
SLC29A1	0.163357	0.041993	0.034223	0.09622	0.137339
SLC29A2	0.036939	-0.02368	-0.07087	-0.06562	-0.03829
SLC29A3	0.206752	0.219139	0.024413	0.220852	0.130938
SLC29A4	-0.01775	-0.22845	-0.2173	-0.10491	0.031429
SLC2A1	0.0648	0.118294	-0.08222	-0.05655	0.004079
SLC2A10	0.234462	0.206963	0.256032	0.223555	0.101519
SLC2A11	0.0479	0.314495	-0.25112	0.198344	-0.21543
SLC2A12	-0.09187	0.02427	-0.13193	-0.21889	-0.25439
SLC2A13	0.179356	0.091694	0.113243	0.142542	0.118855
SLC2A14	0.208278	0.497976	0.489364	0.348296	0.370959
SLC2A2	0.242024	0.274654	0.020805	-0.21642	-0.00888
SLC2A3	0.08736	-0.03026	-0.10002	0.027438	-0.13289
SLC2A4	-0.07374	-0.11853	-0.28706	-0.05267	0.104022

SLC2A4RG	-0.0252	-0.09105	0.031838	0.084796	0.183192
SLC2A5	0.556467	0.549775	0.537898	0.338515	0.470225
SLC2A6	0.139108	0.15995	0.370467	0.246731	0.197265
SLC2A7	0.233724	0.412212	0.643008	0.130513	0.366925
SLC2A8	-0.261	-0.22449	-0.23891	-0.24704	-0.29306
SLC30A1	-0.14728	-0.14923	-0.14696	-0.13041	-0.09022
SLC30A10	0.372177	0.437238	0.489637	0.316896	0.378086
SLC30A2	0.015239	0.175071	0.282629	0.248409	0.064995
SLC30A3	-0.56019	-0.2171	-0.3482	-0.09932	-0.3697
SLC30A5	0.128018	0.303768	0.257306	0.207964	0.167394
SLC30A8	0.172791	0.387343	-1.19247	0.348005	-0.82759
SLC30A9	-0.1766	-0.12521	-0.23288	-0.10508	-0.31347
SLC31A2	0.114014	0.051007	0.05858	0.017782	0.148006
SLC32A1	-0.11604	-0.2301	0.095756	0.176413	0.107292
SLC33A1	-0.075	0.063106	0.028218	0.13154	-0.0475
SLC34A1	0.475784	0.426991	0.484729	0.194363	0.525166
SLC34A2	0.503243	0.331262	0.663449	0.621916	0.531974
SLC34A3	0.247308	0.131217	0.396178	0.18311	0.068614
SLC35A1	0.171209	0.171091	0.172741	0.266421	0.094293
SLC35A2	0.170742	0.039849	-0.16217	0.016388	-0.14416
SLC35A3	-0.17244	-0.44637	-0.75176	-0.58343	-0.62344
SLC35B1	-0.11964	-0.34782	-0.50739	-0.51809	-0.29864
SLC35B2 NFKBIE	0.181483	0.229785	0.379241	0.23597	-0.08203
SLC35B3	0.35893	0.298196	0.25122	0.210492	0.214201
SLC35B4	0.05743	0.582756	0.111159	0.163909	0.135208
SLC35C1	0.057403	0.0639	0.180201	0.028032	0.07264
SLC35C2	0.049656	0.064299	-0.0793	-0.19524	-0.06435
SLC35D1	0.011727	0.141128	0.073205	-0.09659	0.055447
SLC35D2	0.527014	0.676599	0.846303	0.63278	0.52094
SLC35D3	0.05013	0.220213	0.054812	0.188409	0.1431
SLC35E1	-0.07437	0.176302	-0.05135	-0.03783	-0.03711
SLC35E3	0.013857	-0.31755	-0.48558	-0.38207	-0.58045
SLC35E4	0.020081	-0.04775	-0.10701	0.071974	0.04367
SLC35F1	-0.00779	0.09559	0.172115	-0.06348	0.048423
SLC35F2	-0.31352	-0.04755	-0.26293	-0.10078	-0.18335
SLC35F3	0.097309	0.347655	0.455878	0.312373	0.341756
SLC35F5	0.103526	-0.37314	-0.17052	-0.25474	-0.2108
SLC36A1	-0.21053	-0.27492	-0.24127	-0.33901	-0.29085
SLC36A2	-0.06318	-0.44274	-0.11864	-0.34151	-0.66081
SLC36A3	-0.0239	-0.38947	-0.08081	-0.18658	-0.52219
SLC36A4	0.010326	0.276375	-0.02343	0.009292	-0.04313
SLC37A1	0.182794	0.324027	0.444861	0.165421	0.113721
SLC37A2	0.107277	0.271106	0.392926	0.241232	0.289016
SLC37A3	-0.05027	-0.02985	0.031669	0.094367	0.042257
SLC37A4	0.005089	0.040514	0.009078	-0.08808	0.007252
SLC38A1	-0.44764	-0.22337	-0.34337	-0.29277	-0.27724
SLC38A10	0.218264	-0.0295	0.123468	0.023584	0.075354
SLC38A11	0.286529	0.393461	0.335378	0.201973	0.170751
SLC38A2	-0.20927	-0.21088	-0.08984	-0.23749	-0.2103
SLC38A3	-0.03966	0.052973	0.039462	-0.07476	0.024071

SLC38A4	-0.0121	-0.12646	-0.57437	0.037907	-0.60054
SLC38A5	0.298895	0.531614	0.313862	0.431518	0.303105
SLC38A7	0.249972	0.261401	0.243525	0.209129	0.022688
SLC38A9	-0.17434	-0.11144	-0.32064	-0.15623	-0.30047
SLC39A1 CREB3L4	-0.25323	-0.51089	-1.06058	-0.86571	-0.87887
SLC39A10	0.035932	0.214038	0.314029	0.128234	0.037029
SLC39A11	0.087063	0.155533	-0.03702	0.083963	0.162642
SLC39A12	0.220941	0.348583	0.237203	0.11661	-0.46871
SLC39A13	-0.006	-0.13604	0.052708	-0.09359	-0.05014
SLC39A14	0.205192	0.454256	0.38421	0.398861	0.328736
SLC39A2	-0.59223	-0.69298	-0.79638	-0.64756	-0.87426
SLC39A3	-0.23265	0.242934	-0.01628	0.134384	0.299842
SLC39A4	0.098395	0.331834	0.40149	0.178517	0.18736
SLC39A5	0.394038	0.446819	0.251178	0.101329	0.487801
SLC39A6 STATIP1	-0.02692	0.108237	-0.01227	0.064608	0.115905
SLC39A7 HSD17B8	-0.01104	0.020456	-0.19624	-0.12658	-0.08315
SLC39A8	-0.12823	0.151711	0.067058	-0.0174	-0.08437
SLC3A1	0.409741	0.558075	0.858975	0.401259	0.380995
SLC3A2	-0.62257	-0.68695	-0.64353	-0.24698	-0.42181
SLC40A1	0.059013	0.521661	0.536915	0.485408	0.413399
SLC41A1	-0.33531	-0.34241	-0.21948	-0.30506	0.001944
SLC41A2	0.224593	0.418332	0.448913	-0.001	0.205241
SLC43A1	-0.27779	-0.06734	-0.15735	-0.2898	-0.05321
SLC43A2	-0.10123	-0.08373	-0.19954	-0.11921	0.061702
SLC43A3	0.349998	0.552099	0.763433	0.464805	0.468426
SLC44A1	-0.06131	-0.11448	-0.12881	-0.06028	-0.08801
SLC44A2	0.10575	0.175916	-0.004	0.167162	0.001942
SLC44A3	0.245805	0.293568	0.384997	0.340448	0.442424
SLC44A5	-0.51564	-0.38384	-0.96269	-0.99126	-0.9747
SLC45A2	0.450887	0.462262	0.717995	0.402399	0.257663
SLC45A3	0.014284	0.204734	0.061148	0.129485	0.05769
SLC46A1	0.29008	0.272872	0.046784	0.067442	0.231494
SLC46A2	0.202357	0.164455	0.340835	0.311323	0.290395
SLC46A3	-0.14913	0.256514	-0.0792	0.374408	-0.06325
SLC47A1	0.325743	0.298984	0.471239	0.278684	0.379087
SLC47A2	0.06725	0.194296	0.389512	0.224181	0.293422
SLC4A1	0.294775	0.288858	0.299896	0.22936	0.250891
SLC4A10	-0.10744	0.575431	-1.04741	-0.92826	-0.44126
SLC4A11	-0.08831	0.008618	0.43144	0.275869	0.263416
SLC4A3	0.220626	0.364202	0.348281	0.201668	0.389691
SLC4A4	0.181358	-1.37842	-0.82247	-0.99177	-0.84972
SLC4A7	-0.2003	-0.0756	-0.47429	-0.37437	-0.31304
SLC4A8	0.025072	0.15967	0.063336	0.163001	-0.02091
SLC4A9	1.066476	0.032811	-0.25181	-0.17927	-0.23514
SLC5A1	-0.2243	0.245693	0.262565	0.28156	0.292093
SLC5A11	0.285811	0.454474	0.517783	0.218178	0.373555
SLC5A12	-0.19399	0.309247	0.353853	0.019512	0.228038
SLC5A2	0.05785	-0.19247	0.00592	-0.41827	-0.0535
SLC5A4	-0.17685	-0.38791	-0.42004	-0.39173	-0.49143
SLC5A5	0.260996	-0.11955	0.242553	0.255351	0.161876

SLC5A6 C2orf28	-0.27245	-0.16882	-0.2708	-0.19271	-0.25644
SLC5A7	0.104389	0.064131	0.347886	0.338417	0.17541
SLC5A8	-0.09264	-0.29882	0.059048	0.423494	0.007797
SLC5A9	-0.20618	-0.03954	-0.08179	-0.13327	-0.03662
SLC6A1	-0.13998	-0.05739	-0.11395	-0.11944	-0.18885
SLC6A10P. AC142086.3	0.250346	0.087319	0.255587	0.224224	0.306414
SLC6A11	0.262002	0.301336	0.448888	0.253615	0.186379
SLC6A12	0.407887	0.328379	0.553377	0.428851	0.259054
SLC6A13	0.373325	0.618239	0.780815	0.412031	0.529521
SLC6A14	-0.31725	0.347944	-0.20964	0.118147	-0.26077
SLC6A15	-0.11791	0.031884	-0.23279	-0.2787	-0.294
SLC6A16	0.473956	0.660467	0.828311	0.521727	0.560667
SLC6A17	0.166952	0.285968	0.458216	0.239485	0.257224
SLC6A18	0.434239	-0.12003	0.526928	0.398547	0.662725
SLC6A19	0.233152	-0.09043	0.264789	0.229291	0.348931
SLC6A2	-0.17138	-0.113	-0.12133	-0.08826	-0.29635
SLC6A20	0.098185	0.274495	0.286761	0.322067	0.167754
SLC6A3	0.242165	0.111659	0.423862	0.209872	0.379878
SLC6A4	0.098702	0.41875	0.176424	0.379485	0.310534
SLC6A5	-0.30332	0.835076	0.922304	0.557477	0.67512
SLC6A6	-0.03528	0.151237	0.126477	0.176944	0.16823
SLC6A7	0.140378	0.338655	0.485979	0.20095	0.258416
SLC6A8	-0.05852	-0.20418	-0.241	-0.08681	-0.10862
SLC7A1	0.081111	0.108155	0.213602	0.145	0.152268
SLC7A10	0.197828	0.265184	0.497537	0.287526	0.254992
SLC7A11	0.043862	-0.71926	0.372051	-0.09715	-0.65943
SLC7A13	-0.30755	-0.84826	-0.60191	-0.78199	-0.66488
SLC7A14	0.225812	0.287474	0.295441	0.206627	0.270014
SLC7A3	0.059987	-0.27701	0.071617	0.048751	-0.15335
SLC7A4	0.145488	0.145858	0.339083	0.162675	0.196741
SLC7A5	-0.12629	0.062552	-0.09733	-0.1845	0.127053
SLC7A6	0.035509	-0.02997	-0.07323	-0.05302	0.01376
SLC7A6OS PRMT7	-0.01595	-0.16632	-0.26121	-0.09817	-0.16386
SLC7A7	0.325798	-0.68932	0.448	-0.17104	0.137033
SLC7A9	0.354483	0.407476	0.642529	0.393327	0.505007
SLC8A1	0.191775	-0.06407	-0.5671	-0.25809	-0.75275
SLC8A2	-0.41198	-0.39047	-0.75885	-0.3517	-0.55673
SLC8A3	0.359947	-0.01789	0.495719	0.275836	0.136955
SLC8A3 C14orf112	-0.09585	0.036811	-0.14425	-0.06849	-0.12569
SLC9A1	0.039984	0.042418	-0.15642	-0.16627	-0.19445
SLC9A10	0.466782	0.628595	0.666439	0.28037	0.119091
SLC9A11	0.332922	0.347459	0.232879	0.107647	-0.27738
SLC9A2	0.190569	0.141364	0.426999	0.480943	0.444504
SLC9A3	-0.107	-0.03256	0.281897	0.477514	0.077852
SLC9A3R1	0.21569	0.000894	0.100475	0.13873	0.07462
SLC9A3R2	0.080953	0.103432	0.114888	0.101078	0.185495
SLC9A4	-0.6511	-0.9508	-0.85819	-0.75424	-0.45493
SLC9A6	0.349149	-0.12717	-0.30342	-0.2759	-0.12972
SLC9A7	0.108543	0.185803	0.17613	0.124725	0.280795
SLC9A8	-0.25455	-0.12346	0.115964	-0.09029	-0.61904

SLC9A9	-0.33016	-1.05452	-0.96587	-0.30396	-0.49677
SLCO1B1	-0.27706	-0.17735	-0.57245	-0.30968	-0.45778
SLCO1B3	-0.00065	0.309153	0.132654	-0.05529	-0.0404
SLCO1C1	-0.22626	0.523037	0.355207	0.213701	-0.17752
SLCO2A1	-0.3936	0.407008	-0.05437	0.234796	0.030094
SLCO2B1	-0.2567	0.059987	-0.28994	-0.49154	-0.13675
SLCO3A1	-0.24092	-0.10632	-0.19066	-0.19233	-0.19913
SLCO4C1	0.246614	0.406594	0.221346	0.290114	0.140905
SLCO5A1	-0.10021	-0.60033	-0.49939	-0.20507	-0.42939
SLCO6A1	0.612245	0.811065	0.875116	0.416095	0.579643
SLFN11	-0.2748	-0.16023	0.199598	0.160997	0.010704
SLFN12	0.182105	-0.04483	0.104231	0.123357	0.453018
SLFN13	-0.19254	-0.13584	0.628499	0.513955	0.315594
SLFN5	-0.36659	-0.59532	0.353881	0.448755	0.350656
SLFNL1	0.248288	0.266763	0.48603	0.247021	0.17924
SLIT1	0.083332	0.251732	0.365359	0.128158	0.228704
SLIT2	-0.57137	-0.2442	-0.30455	-0.36778	0.57849
SLIT3	0.047026	-0.37205	-0.31343	-0.1382	-0.30877
SLITRK1	-1.18109	0.45655	-0.9787	-1.24636	-1.27787
SLITRK3	-1.02192	0.111954	-0.70414	0.066422	-0.63241
SLITRK4	0.018383	0.486439	0.691343	-0.3545	0.459264
SLITRK5	-0.25839	-0.18753	-0.05107	-0.26332	-0.16507
SLITRK6	0.025907	-0.68744	-0.41769	-0.36452	-0.47642
SLK	-0.15128	-0.07441	-0.14081	-0.06839	-0.07252
SLMAP	0.151287	0.424837	0.56939	0.272464	-0.0389
SLN	0.376418	-0.49937	0.756647	0.394789	-0.66848
SLPI	-0.24829	-0.85081	-0.71632	-0.48811	-0.79933
SLTM	-0.05281	-0.02788	0.017309	-0.05187	-0.00413
SLU7 PTTG2.	-0.21169	-0.21187	-0.43788	-0.35269	-0.59238
SLURP1	0.062819	0.064342	0.238428	0.009907	0.164611
SMAD1	-0.37262	-0.4682	-0.4353	-0.43143	-0.37868
SMAD2	-0.06864	-0.14526	-0.17112	-0.08541	-0.13169
SMAD3	-0.24576	-0.34295	-0.24835	-0.12493	-0.17
SMAD4	0.218864	-0.16589	-0.03742	-0.20503	0.107593
SMAD5OS	0.047426	0.241182	0.021637	0.073459	0.063036
SMAD6	-0.65679	-0.59051	-0.56639	-0.3427	-0.49756
SMAD7	-0.19599	-0.30984	-0.17025	-0.18431	-0.02447
SMAD9	0.039237	0.126162	0.229327	0.143654	0.204996
SMAP1	0.095182	-0.05375	0.046614	0.00511	0.013058
SMAP2	-0.34618	-0.43772	-0.46163	-0.4753	-0.30086
SMARCA1	0.101485	0.229571	0.165542	-0.02252	-0.1371
SMARCA2	-0.4163	-0.10704	-0.28981	-0.3321	-0.25989
SMARCA4	-0.02664	-0.00377	-0.01786	0.089742	0.119494
SMARCA5	-0.20539	-0.4089	-0.42123	-0.29899	-0.35596
SMARCAD1	-0.24673	-0.44602	-0.67963	-0.41117	-0.67464
SMARCAL1	0.392617	-0.13978	-0.00417	-0.34058	-0.46794
SMARCB1	0.157489	0.134921	0.1145	0.19765	0.272803
SMARCC1	-0.07471	0.156477	-0.13021	-0.18129	-0.05358
SMARCC2	-0.38995	-0.55805	-0.51432	-0.49861	-0.58344
SMARCD1	-0.09799	-0.22963	-0.39897	-0.35594	-0.53952

SMARCD2	0.050078	-0.29327	-0.04987	0.088434	-0.12851
SMARCE1 KRT222P	-0.14021	-0.23726	-0.29439	-0.09915	-0.04769
SMC1A RIBC1	0.026703	-0.35815	-0.55415	-0.07622	-0.46367
SMC1B RIBC2	0.337438	0.432408	0.627574	0.105606	0.293411
SMC2	-0.66951	-0.79299	-0.93525	-0.69407	-0.82941
SMC3	-0.39979	-0.40573	-0.54205	-0.35826	-0.4893
SMC5	-0.00975	-0.0335	-0.51273	-0.36537	-0.25054
SMC6	-0.39602	-0.33927	-0.40081	-0.28212	-0.30757
SMCP	-1.0257	-1.812	-1.45438	-1.52508	-1.57718
SMCR7L	0.094437	-0.18643	0.17275	0.126206	-0.28879
SMCY	0.042892	0.166054	0.309662	-0.27669	0.21243
SMEK1	0.069459	-0.08193	-0.01197	-0.02688	-0.06981
SMEK2	-1.02922	-0.68945	-0.89348	-0.74123	-0.35049
SMG5 C1orf85	-1.00153	-1.25587	-1.6092	-1.25722	-1.39137
SMG6 SRR	-0.04828	-0.06612	-0.18195	-0.03116	0.019653
SMG7	-0.61773	-0.84602	-0.67714	-0.65107	-0.62358
SMN1	0.204705	0.20474	0.100537	0.08097	0.080432
SMN2	0.194026	0.008733	0.053911	0.014752	0.055577
SMNDC1	-0.00116	-0.07391	0.000422	-0.19227	-0.17639
SMO	-0.07549	0.076139	-0.26777	-0.18643	0.065098
SMOC1	-0.04463	0.071627	0.03897	0.052625	0.275486
SMOC2	0.160171	0.260548	0.16474	0.176164	0.317452
SMPD3	0.073598	-0.07816	-0.23863	0.02762	0.102955
SMPD4 FAM128B	-0.11698	-0.27221	-0.25516	-0.14317	-0.08593
SMPDL3A	-0.0741	0.290815	-0.37462	-0.17464	0.025898
SMPDL3B	-0.00883	0.103718	0.202981	-0.03608	-0.08559
SMPX	0.229558	0.496692	0.182089	0.237601	0.502974
SMR3A	-0.12033	-0.96416	-0.79745	-0.76882	-0.62437
SMR3B	0.406299	-0.74957	-0.73719	-0.33834	0.149611
SMS	0.262653	0.48881	0.289788	0.113529	0.213277
SMTN	-0.42796	-0.3359	-0.72167	-0.26298	-0.37051
SMTNL2	0.107474	0.205971	0.324634	0.204188	0.268605
SMU1	-0.04674	-0.05928	-0.50305	-0.07299	-0.26331
SMUG1	-0.40818	-0.83763	-0.88433	-0.75672	-0.77411
SMURF1	0.158581	-0.09844	-0.3641	-0.0351	-0.05753
SMURF2	-0.00763	-0.03228	-0.00766	-0.0774	0.03295
SMYD1	0.480806	0.575831	0.740553	0.359841	0.50985
SMYD2	-0.20537	-0.09829	-0.17456	-0.23646	-0.10073
SMYD3	-0.09802	-0.26604	-0.39714	-0.70702	-0.45572
SMYD4 RPA1	-0.13171	0.060984	-0.24762	-0.15663	-0.23254
SMYD5	0.305615	0.303024	0.459152	0.140768	0.18683
SNAI1	0.241712	0.175128	0.128352	0.07937	0.153864
SNAI2	-0.18026	-0.15292	-0.14447	-0.20869	-0.19688
SNAI3	0.114467	0.03767	0.097893	0.137781	-0.04028
SNAP23	0.100811	0.026535	0.038438	0.038319	-0.16546
SNAP25	-1.00352	-0.79224	-0.98002	-0.62829	-0.89516
SNAP91	0.181992	0.247655	0.172546	0.119216	0.167123
SNAPC1	-0.08903	-0.31527	-0.62989	-0.46377	-0.75663
SNAPC3	0.186768	0.070998	0.160975	0.141378	0.220801
SNAPC4	0.285213	0.479858	0.426266	0.343654	0.195861

SNAPC5	-0.06966	-0.40716	-0.28555	0.166212	-0.41607
SNAPIN	0.20247	0.019465	0.070832	-0.01002	0.048402
SNCA	0.045646	0.359396	-0.27632	0.139945	0.333623
SNCAIP	-0.14186	0.108688	0.138153	-0.02076	-0.17025
SNCB	-0.26004	0.031661	0.502285	0.341866	0.428916
SNF1LK	0.25579	0.142223	0.171935	0.280912	0.347729
SNF1LK2	-0.02077	-0.04607	-0.10273	-0.10278	-0.09227
SNF8	0.124156	-0.04931	0.183541	0.074882	0.198416
SNFT	-0.12683	-0.10174	-0.19434	-0.18266	-0.08558
SNIP1 RP3-423B22.5 DNALI1	0.073802	0.079881	-0.11858	0.203899	0.225774
SNN	0.13332	0.177181	0.276535	0.156077	0.090978
SNPH	-0.03912	0.300525	-0.02958	-0.13145	0.185399
SNRK	0.077535	0.437201	0.233145	0.216601	0.239234
SNRP70	0.179355	0.246662	0.072839	0.204274	0.203689
SNRPA1	-0.12746	-0.37017	-0.33098	-0.26145	-0.36236
SNRPB	-0.09108	-0.02857	0.083259	0.035711	-0.05284
SNRPB2	-0.82082	-0.51223	-0.05275	-0.05135	-0.54261
SNRPC	-0.28675	-0.38187	-0.71736	-0.53529	-0.7125
SNRPD1	0.180782	0.298826	0.332293	0.211652	0.27533
SNRPD2 QPCTL	-0.38227	-0.0792	-0.2085	-0.068	-0.19723
SNRPE	0.043303	-0.71092	-0.23609	-0.1731	-0.37915
SNRPF	-0.40536	0.162022	-0.32493	0.216039	-0.10035
SNRPG	-0.60333	-0.30766	-0.62677	-0.33989	-0.10827
SNTA1	-0.14767	-0.27652	-0.21396	-0.06081	-0.00695
SNTB1	-0.34511	0.332809	0.080269	0.213545	0.310233
SNTB2	0.145614	0.108402	0.172933	0.141617	0.251491
SNTG2	-0.12326	0.167084	0.031715	0.103861	0.166544
SNUPN	0.108201	0.003459	-0.16398	-0.05301	0.019483
SNW1 C14orf178	-0.04377	0.088237	-0.08912	-0.17057	-0.24829
SNX10	0.23379	0.280169	-0.04336	0.111727	0.006803
SNX11	0.130777	-0.08047	-0.40163	-0.15299	-0.02651
SNX12	0.065672	-0.11947	0.312688	-0.16894	-0.24311
SNX14	-0.33465	-0.46225	-0.47672	-0.28197	-0.47596
SNX15	-0.16517	-0.11929	-0.0409	-0.1737	-0.19535
SNX15 SAC3D1	-0.13262	0.006525	0.115101	-0.04383	0.196927
SNX16	0.158228	0.173136	0.095536	-0.00132	0.09724
SNX18	0.287474	0.261192	0.372658	0.211914	0.282272
SNX19	0.37998	0.385703	0.465905	0.341306	0.414795
SNX2	0.204166	-0.04258	-0.04263	-0.09899	-0.30689
SNX20	0.10598	0.319713	0.357733	0.182154	0.118784
SNX22	0.117742	0.257692	-0.00626	0.155422	0.085945
SNX24	0.169876	0.357281	0.461674	0.31265	0.271144
SNX25	-0.12697	-0.26166	-0.03926	0.040662	-0.04982
SNX27	-0.42336	-0.56731	-0.75308	-0.69696	-0.49114
SNX3	0.246416	0.121754	0.18015	0.093049	0.139117
SNX31	0.334093	0.567776	0.408424	0.332306	0.307474
SNX32	-0.11187	-0.24666	-0.55194	-0.48627	-0.60214
SNX4	0.026921	-0.00612	-0.14431	-0.07232	-0.11786
SNX5 C20orf72	-0.18159	-0.04665	-0.03388	-0.06533	-0.1454
SNX6	0.234743	0.312371	0.24707	0.250457	0.082965

SNX7	0.112583	-0.10737	-0.13976	0.240503	0.181494
SNX8 CHST12	-0.12161	-0.36317	-0.35962	-0.25804	-0.33561
SNX9	-0.01338	0.114164	0.158614	0.12651	0.250343
SOAT1	-0.02387	0.075169	-0.05626	0.12992	0.091013
SOBP	-0.4382	-0.40934	-0.50514	-0.28151	-0.46667
SOCS1	-0.06265	-0.05292	-0.07428	-0.04393	-0.14264
SOCS3	-0.13139	-0.18574	-0.12282	-0.06911	0.003898
SOCS5	-0.00879	0.015147	-0.13045	-0.09004	-0.14545
SOCS6	0.031989	0.046495	0.061384	-0.04983	0.074631
SOCS7	-0.01945	-0.12393	-0.03104	0.059401	0.040829
SOD1	0.135123	0.214822	-0.0581	0.003062	0.050152
SOD2	-0.01359	-0.00356	-0.01632	-0.12274	-0.00525
SOHLH1 KCNT1	0.260469	-0.01579	0.216677	0.185285	0.199889
SOHLH2	0.199026	0.176955	0.341824	0.145041	0.199806
SOLH	0.076792	-0.0612	0.182571	0.086448	0.064045
SORCS1	-0.03526	-0.06343	-0.03558	-0.10453	-0.09694
SORCS2	0.152699	0.241543	0.284256	0.218897	0.283355
SORCS3	-0.13702	-0.09721	-0.19854	-0.05156	0.284234
SORD	0.022958	0.110965	0.27643	-0.02122	0.06014
SORL1	0.284043	0.50019	0.464175	0.381877	0.422175
SORT1	-0.06481	0.230806	0.131945	0.108577	0.084843
SOS1	-0.01715	-0.04572	0.140009	0.040004	0.088189
SOS2	0.199692	0.064857	0.240744	0.137775	0.178702
SOST	0.509588	0.575346	0.438153	0.523338	0.475027
SOSTDC1	0.469473	0.051965	-0.88791	-0.69046	-0.80122
SOX1	-0.11694	0.242441	0.266036	0.168088	0.30527
SOX11	-0.25668	-0.37328	-0.11517	-0.17736	-0.12088
SOX12	-0.43477	-0.35407	-0.26058	-0.13232	-0.12832
SOX13	0.117158	0.157296	0.326566	0.261687	0.195349
SOX14	-0.55168	-0.21941	0.39403	0.342941	0.471312
SOX15	0.15017	0.11948	0.401375	0.353968	0.441094
SOX17	-0.17697	0.031353	0.535342	0.424209	0.577336
SOX18	0.067474	0.042345	0.333104	0.080828	0.065326
SOX2	-0.70898	0.015735	0.41294	0.039971	0.170165
SOX21	-0.21127	0.276148	0.387499	0.32398	0.125184
SOX3	0.0343	0.543852	0.61168	0.361086	0.522212
SOX30	0.578486	0.642467	0.749848	0.490004	0.775884
SOX4	-1.03384	-0.93538	-1.02927	-0.94657	-1.05784
SOX7	0.108738	0.056148	0.200668	0.132233	0.192346
SOX8	-0.08266	0.114893	0.404732	0.056354	0.12225
SOX9	-0.24727	-0.39068	-0.44061	-0.44944	-0.19833
SP1	-0.77153	-0.6578	-0.8578	-0.6618	-0.80914
SP100	-0.1137	-0.26887	0.083971	0.250611	-0.13191
SP110	0.053125	-0.17347	-0.4999	0.066695	-0.50412
SP140	0.190947	0.315233	0.200926	-0.00431	-0.29249
SP3	-0.29814	-0.15925	-0.30742	-0.15738	-0.15879
SP4	-0.40024	-0.41632	-0.39718	-0.28542	-0.23347
SP5	-0.07771	-0.13563	0.001232	0.082812	0.143618
SP6	-0.52865	-1.35181	-1.02896	-1.25072	-0.9276
SP8	-1.02018	-1.28895	0.119459	-0.6505	-0.45219

SPACA1	0.524798	0.54256	0.787078	0.364395	0.577258
SPACA3	-0.12392	-0.17873	-0.20461	-0.2733	-0.16864
SPAG1	0.054227	-0.25535	-0.11338	0.004283	-0.06642
SPAG17	0.067982	-1.1016	0.087876	0.241621	-0.55394
SPAG4	0.091225	0.234935	0.155037	0.119489	0.098809
SPAG4L	0.070076	-0.68083	-0.52601	-0.16738	-0.22175
SPAG5	-0.20293	0.141631	-0.27658	0.123338	0.031383
SPAG6	0.08322	0.295431	0.429146	0.436831	0.446308
SPAG7 CAMTA2	-0.61103	-0.45657	-0.97065	-0.43292	-0.58298
SPAG8	0.099956	0.043403	-0.04703	0.312195	0.057913
SPAM1	-0.49385	-0.17116	-0.5984	-0.43592	-0.70541
SPANXA2	0.439035	-0.65242	-0.56731	0.589647	0.243919
SPANXB2	0.158744	-0.85015	-1.13362	0.316906	0.008249
SPANXC	0.363177	-0.84302	-1.05815	0.204278	0.124849
SPANXD	0.329264	-0.7451	-0.71902	0.388917	0.239609
SPANXN2	-0.41619	-0.88866	-1.0896	-0.25529	-0.6042
SPANXN3	0.100875	-1.33569	-1.90865	-0.0591	-0.41596
SPANXN4	-0.14153	-0.98935	-1.34138	-0.0041	-0.29273
SPARC	-0.51067	-0.62331	-0.61967	-0.28398	0.161414
SPARCL1	0.138883	0.179227	-0.17021	0.017586	-0.29002
SPAST	-0.3201	-0.44657	-0.25668	-0.21179	-0.3225
SPATA12	0.433869	0.189992	0.621871	0.102859	0.093148
SPATA16	0.182569	-0.05607	0.408077	0.117309	0.267496
SPATA18	0.222033	0.56362	0.563112	-0.06842	0.567838
SPATA19	-0.04852	-0.23676	-0.76525	-0.38579	-0.70366
SPATA2	-0.09174	-0.05238	-0.2632	-0.03004	-0.14681
SPATA20	0.26531	0.211303	0.108153	0.065278	0.14297
SPATA21	0.11283	0.371536	0.096356	0.048235	0.152686
SPATA22	0.499815	0.300859	0.63874	0.455212	0.499635
SPATA4	-0.0922	-0.08335	-0.25994	-0.36858	-0.2346
SPATA6	-0.20987	-0.3083	-0.86768	-0.07002	-0.26125
SPATA8	0.312123	-0.18528	-0.26449	0.195394	0.016675
SPATA9	0.235728	0.417327	0.284022	-0.07622	0.100279
SPATC1	0.268549	0.551443	0.413215	0.34599	0.385193
SPATS2	-0.2519	-0.07767	-0.41632	-0.37695	-0.31494
SPBC25	-1.09194	-0.97499	-1.69198	-0.97732	-0.92763
SPC24	0.186263	0.203665	0.193294	0.217192	0.351024
SPCS3	-0.24082	-0.62223	-0.28976	-0.23567	-0.3851
SPDEF	0.246347	0.066588	0.353999	0.285117	0.445837
SPDYC	0.459222	0.651828	0.774021	0.450202	0.6278
SPEF2	0.0588	0.53617	0.351439	0.220312	0.269817
SPERT	-0.67511	-0.62118	-0.75747	-0.78475	-0.97339
SPG11	0.457047	0.358456	0.408705	0.250745	0.197055
SPG20 AL139377.8	-0.21052	-0.35893	0.410047	-0.35186	0.263699
SPG21	0.157978	0.17544	0.306329	0.066409	0.098869
SPG3A	-0.51744	-0.45946	-0.84908	-0.74537	-0.70736
SPG7	0.092536	0.15075	0.24734	0.176546	0.12784
SPHK1	0.175946	-0.00791	0.123426	0.264681	0.31981
SPHKAP	0.346624	0.647151	0.852766	0.507394	0.62791
SPI1	0.05061	0.21369	0.071124	0.05395	0.154803

SPIB	0.246983	0.193714	0.558297	0.416017	0.341501
SPIC	0.028114	-0.06811	-0.23589	-0.16312	-0.31235
SPIN1	-0.08714	-0.01502	-0.20175	-0.09176	0.147159
SPIN2A	0.23079	0.362562	0.261172	0.206119	0.21647
SPIN2B	0.115715	-0.22481	-0.35732	-0.23022	-0.26593
SPIN3	0.147045	-0.02375	0.010813	0.299368	-0.46761
SPIN4	0.089463	0.101105	0.058219	-0.00061	-0.05069
SPINK1	0.078934	-0.19187	0.306932	-0.08272	-0.14266
SPINK2	0.134474	-0.01945	-0.0672	0.008421	0.03217
SPINK4	0.365298	0.268967	-0.33096	-0.07509	-0.04078
SPINK5	0.245557	-0.34239	0.647772	-0.33422	-0.73881
SPINK6	-0.08773	0.029579	0.102307	-0.26189	-0.07887
SPINK7	0.304069	-0.49585	0.382843	-0.38723	-0.60802
SPINT1	0.11015	0.499175	0.471654	0.377505	0.342468
SPINT2	0.064504	0.441952	0.587201	0.350332	0.526794
SPIRE2	-0.12987	-0.16385	-0.13256	0.050061	-0.01028
SPN	0.269013	0.524357	0.383513	0.190717	0.517086
SPNS1	-0.16349	-0.28676	-0.28672	-0.14648	-0.14053
SPNS1 LAT	0.093778	0.112819	0.230063	-0.02164	0.175773
SPNS3	0.456362	0.614224	0.542749	0.338636	0.306059
SPO11	0.446334	0.717813	0.774008	0.405835	0.603665
SPOCD1	0.482807	0.712242	0.790174	0.522069	0.606249
SPOCK1	0.10874	0.182766	0.070548	0.267105	0.396635
SPOCK2	0.401132	0.534875	0.676148	0.015241	0.518198
SPON1	0.202039	0.479057	0.56864	0.261725	0.421391
SPON2	0.20098	-0.00567	0.409415	0.377946	0.336122
SPOP	-0.21607	-0.09929	-0.54446	-0.40208	-0.56762
SPOPL	0.003917	-0.14285	0.07927	-0.09989	0.066347
SPP1	-0.07639	0.353698	-0.12783	-0.16149	-0.36355
SPP2	0.04899	0.015531	0.208913	-0.11001	-0.02829
SPR	0.046301	-0.01699	-0.05823	-0.00377	0.056238
SPRED1	-0.32217	-0.20847	-0.2228	-0.21295	-0.17326
SPRED2	-0.3256	-0.29499	-0.45701	-0.28863	-0.13344
SPRN.	0.076029	0.269955	0.283875	0.171575	0.153496
SPRR1A	-1.13432	-2.01406	-1.91466	-1.67038	-1.38993
SPRR1B	-0.66848	-1.29616	-1.44021	-1.22237	-1.10552
SPRR2D	-1.30878	-1.85194	-1.7611	-1.24178	-1.41107
SPRR2G	-0.90636	-1.50892	-1.57232	-1.07802	-1.20519
SPRR3	-0.21017	-1.19728	-0.94371	-0.82844	-0.71899
SPRR4	0.057665	-1.97186	-1.70842	-1.02513	-0.44808
SPRY2	-0.17214	-0.26626	-0.10495	-0.11923	-0.20752
SPRY3	-0.55271	-1.55375	-2.02647	-1.18719	-1.18045
SPRY4	-0.18853	-0.39459	-0.21481	-0.00928	-0.31277
SPRYD3	0.156625	0.003327	-0.02307	0.080497	-0.12191
SPRYD4	0.180304	0.196528	-0.27403	0.031331	-0.14814
SPRYD5	-0.05256	-1.15456	-1.10975	-0.87621	-1.08554
SPSB1	-0.05701	-0.20929	-0.08082	-0.0491	-0.07951
SPSB2	0.093314	-0.12981	-0.27039	-0.19186	-0.18525
SPSB3 NUBP2	0.076248	0.115771	0.026432	0.031455	-0.05163
SPSB4	0.161261	0.19865	0.137805	0.208348	0.179823

SPTAN1	-0.11123	-0.33995	-0.12884	-0.23329	-0.14011
SPTB	0.225572	0.253349	0.31151	0.146824	0.122257
SPTBN1	-0.41458	-0.43968	-0.57023	-0.3862	-0.16937
SPTBN2	0.463267	0.645902	0.679133	0.597151	0.745745
SPTBN4 SHKBP1	-0.14472	-0.06121	0.008387	0.320634	0.10279
SPTBN5 AC020659.5	0.270479	0.230123	0.265956	0.205674	0.157968
SPTLC1	0.15098	-0.14434	-0.44247	0.015231	-0.51939
SPTLC2	-0.4003	-0.30007	-0.51261	-0.40717	-0.45914
SPTLC2L	0.130449	-0.82739	0.313117	-0.37187	-0.82867
SPTY2D1	0.028418	-0.04322	-0.13445	-0.1531	0.052432
SPZ1	-0.87909	-1.03525	-1.17587	-1.07931	-0.99117
SQLE	0.043765	0.064625	-0.1431	-0.05887	0.107816
SQRDL	0.167731	0.223524	0.134684	0.278276	0.076523
SQSTM1	0.252678	0.187141	0.336452	0.206532	0.345303
SRA1 APBB3	0.413245	0.589767	0.659173	0.40554	0.40855
SRBD1	-0.48813	-0.92864	-1.10173	-0.99874	-1.16575
SRC	0.280762	0.377748	0.304225	0.140749	0.364473
SRCAP	0.385452	0.640991	0.747707	0.123317	0.378861
SRCRB4D	0.481069	0.364375	0.444934	0.352403	0.524607
SRCRB4D ZP3	0.019738	0.167494	0.089484	0.08281	-0.03425
SRD5A2	0.158551	0.055379	0.534403	0.415297	0.438895
SRD5A2L	-0.06893	-0.02636	0.0453	0.00867	0.131658
SREBF1	0.100232	0.004397	0.074045	-0.0667	0.187061
SRF	-0.25122	-0.31236	-0.10262	-0.08668	0.015738
SRFBP1	-0.28141	-0.31935	-0.43757	-0.44758	-0.56051
SRGAP1	-0.59468	-0.53428	-0.57143	-0.11778	-0.5906
SRGAP2.	0.476802	0.634877	0.442842	0.070144	0.156734
SRGAP3	-0.85667	-0.02032	-0.7646	-0.39213	-0.43257
SRGN	0.387904	0.603632	0.532062	0.18153	0.223254
SRM	-0.12815	-0.08134	-0.13066	0.006245	-0.15363
SRMS	0.170867	0.370657	0.497427	0.233232	0.243172
SRP14 AC021755.9	0.004987	0.078194	-0.15258	-0.05127	-0.05161
SRP19	-0.05065	0.033879	-0.21904	-0.11313	0.021902
SRP54	-0.54425	-0.54928	-0.64438	-0.47443	-0.57089
SRP68 GALR2	0.025093	0.167267	0.029213	0.011735	-0.03174
SRP72	-0.7625	-0.51326	-0.6141	-0.39372	-0.5077
SRP9	0.29433	0.240952	0.369354	0.178659	0.177992
SRPK1	-0.0936	-0.17748	-0.11268	-0.18382	-0.09592
SRPK3	0.184437	0.427219	0.353624	0.300557	0.210734
SRPR FOXRED1	-0.35295	-0.14371	-0.45788	-0.18984	-0.42093
SRPRB	-0.28885	0.048219	0.12359	-0.01804	-0.16984
SRPX	0.284297	0.458116	0.197607	0.147547	-0.31005
SRPX2	0.249044	0.146145	0.310944	0.238116	0.280548
SRRM1	0.025028	-0.01587	0.053089	0.121392	0.053728
SRRM2	0.014689	-0.03129	-0.08124	0.051626	-0.00344
SRXN1	0.258887	0.41368	0.361908	0.23862	0.23508
SRY	-0.02124	0.152142	0.315449	0.116738	0.301341
SS18	-0.22433	-0.3418	-0.40966	-0.23415	-0.16736
SSB	-0.19637	-0.02841	-0.16693	-0.1917	-0.1206
SSBP1	-0.19538	-0.37527	-0.9468	-0.57893	-0.45509

SSBP2	-0.43221	-0.30625	-0.35452	-0.39121	-0.38265
SSFA2	-0.31664	-0.2729	-0.19948	-0.22428	-0.0392
SSH1	0.005264	0.290519	0.353379	0.231415	0.391972
SSH2	-0.61778	-0.42443	-0.81869	-0.39255	-0.15473
SSH3	0.258443	0.34845	0.320041	0.33877	0.316293
SSH3BP	-0.10581	-0.33506	-0.19133	-0.27424	-0.34647
SSPN	0.113928	0.164103	-0.0392	0.033271	0.088223
SSR1	-0.14151	0.104612	0.036213	-0.03986	-0.04773
SSR2	0.176211	0.087314	-0.23892	-0.06357	-0.16282
SSR3	0.297457	-0.10381	0.540344	0.285468	-0.44272
SSRP1 P2RX3	0.298079	0.284363	0.440494	0.11624	0.26976
SSSCA1	-0.27673	-0.30287	-0.47507	-0.12898	-0.14331
SST	-0.51195	-0.71579	-0.77323	0.247744	-1.03991
SSTR1	-0.46877	-0.71049	-0.31538	0.035626	0.83934
SSTR2	-0.43072	-0.13839	-0.06946	-0.51246	-0.09509
SSTR3	-0.02735	0.010569	-0.23139	-0.00238	-0.27071
SSTR4	0.465961	-0.06503	0.086006	0.261189	0.548682
SSTR5	0.404455	0.127023	0.345135	0.314918	0.391264
SSU72	0.035172	-0.1093	0.10147	0.234753	-0.18393
SSX2 RP11-552J9.12	0.715499	0.528693	0.469414	0.398784	0.463343
SSX2IP	-0.17167	-0.16526	-0.18109	-0.23811	-0.12216
SSX5 RP11-552E4.2	0.609567	0.33156	0.170028	0.21234	0.548092
SSX6	0.175687	0.258196	-0.11691	0.254022	0.269847
ST13 XPNPEP3	-0.17377	0.135329	0.012554	-0.10121	-0.34137
ST14	0.1642	0.238608	0.386234	0.100158	0.21538
ST18	0.556032	0.061689	0.19024	0.366958	0.552352
ST20	-0.19702	0.067833	-0.30313	-0.4736	-0.6011
ST3GAL1	-0.25496	-0.17081	-0.16252	-0.13814	-0.0857
ST3GAL2	-0.08213	0.031125	-0.1726	-0.04344	-0.06599
ST3GAL3	-0.12529	-0.2324	-0.15348	-0.129	-0.06504
ST3GAL4	0.106867	0.080204	0.046098	0.097616	0.199819
ST3GAL5	0.018259	0.082119	-0.13213	-0.04038	0.029251
ST3GAL6	-0.28064	-0.16513	-0.12187	-0.17082	-0.22622
ST5	-0.18231	0.19044	0.126616	0.295541	0.106127
ST6GAL2	0.05462	-0.07221	-0.02669	0.050417	0.134537
ST6GALNAC1	0.393584	0.314171	0.275794	0.081632	0.441188
ST6GALNAC2	0.077586	0.061994	0.352136	0.188185	0.196154
ST6GALNAC3	0.043579	-0.15442	-0.0866	-0.09508	0.034931
ST6GALNAC4	0.172137	0.010464	0.087606	-0.19455	-0.11797
ST6GALNAC5	-0.05364	-0.05798	-0.71153	-0.80298	-0.01875
ST6GALNAC6	0.105428	0.323058	0.409254	0.23136	0.220539
ST7	-0.20202	-0.2639	-0.57479	-0.51408	-0.33584
ST7L CAPZA1	-0.83453	-0.58327	-0.6641	-0.78805	-0.75355
ST8SIA1	-0.20059	-0.05886	-0.39282	-0.2102	-0.11592
ST8SIA2	-0.21049	-0.00716	0.032257	-0.03858	-0.08431
ST8SIA4	-0.19824	0.262758	0.033904	-0.0013	0.086412
ST8SIA5	-0.16401	0.2328	0.168502	0.321818	0.338157
ST8SIA6	-0.0411	-0.05675	0.010509	0.007471	0.058072
STAB1	0.199315	0.327294	0.451693	0.282747	0.050984
STAB2	0.363967	0.226962	0.459043	0.268679	-0.45225

STAC	-0.18779	-0.06537	-0.24113	-0.2651	-0.07824
STAC2	-0.00885	0.06723	-0.1236	0.045081	0.009222
STAC3	0.173694	0.209652	0.282925	0.051664	0.32457
STAG1	-0.03891	-0.08697	-0.09363	-0.03883	0.105679
STAG2	-0.31527	-0.81327	-0.35619	-0.64576	-0.87374
STAG3	0.670163	0.71793	0.93409	0.595758	0.55416
STAM	-0.51532	-0.55356	-0.42721	-0.19119	-0.29187
STAM2	-0.04867	0.10832	-0.34867	-0.11501	-0.28058
STAMBP	-0.23149	-0.22564	-0.43002	-0.35287	-0.54225
STAP2	0.411979	0.356625	0.637027	0.302421	0.403912
STAR	0.380333	0.72785	0.833757	0.474243	0.512859
STARD10	-0.53008	-0.25229	-0.72789	-0.54146	-0.59673
STARD3NL	-0.23061	-0.47106	-0.76016	-0.45186	-0.3189
STARD4	-0.29049	-0.66173	-0.68024	-0.52062	-0.58233
STARD5	0.092873	-0.05294	-0.28459	0.016525	-0.28251
STARD6	0.228313	0.549612	0.381699	0.01274	0.079443
STARD7 AC012307.8	-0.01717	0.082447	0.118349	0.01073	0.064176
STARD8	0.388477	0.21909	0.133186	-0.24359	0.220609
STAT1	0.246315	0.037031	0.098658	-0.11337	-0.26907
STAT2	-0.69746	-0.74377	-1.10901	-0.84975	-1.11481
STAT3	0.105998	0.058926	0.071672	-0.15441	-0.08103
STAT4	0.5619	0.683511	0.881749	0.467687	0.404169
STAT5A	0.136447	0.229172	0.262751	0.115285	0.328662
STAT5B	0.153817	0.188993	0.270912	0.241642	0.183693
STAT6	-0.12712	0.032753	0.26651	-0.09279	0.01028
STATH	-0.21951	-0.70625	-0.49261	-0.46995	-0.22462
STAU1	0.104663	0.359256	0.24124	0.10288	0.089376
STBD1	-0.06277	-0.14408	-0.20255	0.13607	0.209525
STC1	-0.45072	-0.65978	-0.70369	-0.68502	-0.92074
STC2	-0.16697	0.003825	0.216601	0.367508	-0.10344
STCH	-0.54454	-0.36946	-0.46099	-0.447	-0.52149
STEAP1	0.126389	0.400367	0.118384	0.154629	0.067085
STEAP2	0.179379	0.652026	0.241674	0.476087	0.353195
STEAP3	0.322634	0.312267	0.485143	0.379381	0.356042
STEAP4	0.168409	0.360513	0.350073	0.215463	0.396569
STIL	-0.02627	-0.02344	-0.23365	-0.003	-0.03111
STIM1	-0.09886	-0.01772	-0.13743	-0.08885	-0.14515
STIM2	-0.44653	-0.52156	-0.35056	-0.27492	-0.49921
STIP1	-0.41866	-0.30048	-0.40094	-0.26749	-0.02886
STK10	-0.0911	0.114891	0.080295	0.013963	-0.02566
STK11	0.163493	0.138997	0.332066	0.251623	0.332217
STK11IP	-0.18295	-0.49301	-0.88757	-0.20675	-0.38446
STK17A	-0.04983	-0.13708	-0.34874	-0.0498	-0.2929
STK17B	-0.1924	-0.12917	-0.29139	-0.15791	-0.21649
STK19 C4B	0.335404	0.161268	0.380457	-0.2863	0.126917
STK25	0.061331	0.115846	0.275466	0.120368	0.140318
STK3	-0.01582	0.063899	0.168111	0.055983	0.174641
STK31	0.596557	0.899984	0.913152	0.437899	0.568346
STK32A	-0.18659	-0.16701	-0.24542	-0.05614	-0.21769
STK32B	0.043466	0.308474	0.293618	0.349185	0.296137

STK32C	-0.00253	0.018343	0.071418	0.108911	0.228826
STK32C LRRC27	0.045211	-0.01753	-0.04607	0.119244	0.121365
STK33	-0.07776	-0.25793	-0.10016	-0.1456	-0.06069
STK35	-0.64196	-0.43001	-0.54992	-0.28991	-0.21461
STK38	-0.18529	-0.17038	-0.29971	-0.33772	-0.47827
STK38L	0.351448	0.439042	0.498609	0.199571	0.328463
STK39	-0.06993	0.230474	0.291451	-0.00399	0.177183
STK4	-0.0071	-0.03667	0.247302	0.05974	-0.04057
STK40	-0.57751	-0.46571	-0.47663	-0.40639	-0.32761
STMN1	0.153733	0.247361	0.233153	0.048482	0.32781
STMN2	-0.55426	-0.46343	-0.93329	-0.71765	-0.89462
STMN4	0.061669	-0.26897	-0.26704	-0.34865	-0.34919
STOM	0.301858	0.468828	0.669424	0.401036	0.21476
STOML1 PML	0.042483	-0.08722	-0.18824	0.017756	-0.10797
STOML2	-0.90407	-0.73515	-0.90359	-0.60804	-0.72873
STOML3	0.179547	0.264888	0.316743	-0.29393	-0.02035
STON2	-0.21563	-0.33894	-0.26622	-0.36042	-0.5169
STOX1	0.189285	0.246343	0.329346	0.228986	0.227489
STOX2	-0.21357	-0.26047	-0.37648	0.019862	-0.07811
STRA13 LRRC45	-0.03452	-0.15367	-0.18401	-0.0676	0.056678
STRA6	0.292174	-0.30757	-0.50713	-0.4169	-0.42997
STRA8	0.139798	0.191165	0.195474	-0.08996	-0.36078
STRAP	-0.0239	-0.05199	-0.36049	0.040111	-0.11215
STRBP	-0.23856	-0.08922	-0.21827	-0.10232	-0.09274
STRN	-0.38465	-0.36524	-0.4352	-0.26062	-0.31139
STRN3 AP4S1	-0.28686	-0.26184	-0.23865	-0.18472	-0.26889
STRN4 FKRP	-0.13829	-0.175	-0.05702	-0.14717	-0.06218
STS	0.31561	0.053823	0.217055	0.159771	0.140282
STT3A	-0.82251	-0.48681	-1.09325	-0.63413	-0.87401
STT3B	0.049294	-0.04385	-0.03809	-0.03116	0.065895
STUB1	-0.12158	-0.07647	-0.23761	-0.11369	-0.18817
STX10 IER2	-0.4853	-0.38908	-0.45304	-0.30735	-0.51606
STX11	0.144595	0.222841	0.167214	0.197772	0.297176
STX17	0.195587	-0.38996	-0.64342	-0.18698	-0.29602
STX18	-0.33365	-0.25964	-0.36624	-0.32645	-0.60504
STX1A	0.016758	0.013275	0.074412	0.034687	0.122087
STX1B2	0.119416	-0.11399	-0.39138	-0.1663	-0.066
STX2	0.021406	0.044452	-0.03153	-0.03123	-0.01082
STX3	-0.35288	-0.19968	-0.15197	-0.11621	-0.13437
STX4	-0.653	-0.54656	-0.68526	-0.49718	-0.37346
STX5	-0.26747	-0.60395	-0.8098	-0.34846	-0.37396
STX6	-0.03642	-0.01391	-0.07969	-0.08464	0.115362
STX7	-0.26315	-0.26334	-0.50577	-0.46326	-0.58306
STX8 WDR16	-0.70701	-0.50291	-0.75191	-0.27893	-0.36956
STXBP1	0.025252	0.091883	0.194812	0.162769	0.057907
STXBP2	0.3124	-0.15984	0.445684	0.275979	0.309825
STXBP3	-0.17785	-0.1254	-0.3043	-0.43807	-0.26826
STXBP5	-0.49746	-0.18287	-0.4793	-0.40287	-0.44164
STXBP6	-0.02126	-0.39183	0.139879	0.298922	0.328777
STYK1	-0.01773	0.048363	0.602726	0.382795	0.234036

STYX	-0.30113	-0.06592	-0.14059	-0.17612	-0.26447
STYXL1 MDH2	-0.1156	-0.20094	-0.38496	-0.0863	-0.13944
SUB1	0.128923	0.391929	0.461272	0.098075	0.183107
SUCLA2	0.182973	0.441764	0.306535	0.284323	0.244372
SUCLG1	-0.57722	-0.66411	-0.73286	-0.60851	-0.40769
SUCLG2	-0.02724	0.060567	-0.04912	0.319833	0.138215
SUDS3	-0.03977	0.101456	0.091631	0.020337	0.16354
SUGT1	-0.24184	-0.33793	-0.43324	-0.31118	-0.34156
SUHW4.	0.368804	0.422408	0.515601	0.113852	0.398835
SULF1	-0.47353	-0.53502	-0.57242	0.050556	-0.27969
SULF2	-0.15534	-0.14745	-0.08102	-0.14292	-0.22951
SULT1A1	0.38466	0.475585	0.55029	0.295968	0.273399
SULT1A2	0.153168	0.388107	0.395508	0.077657	0.238344
SULT1A2 SULT1A1	0.110715	0.342545	0.437376	0.022248	0.249313
SULT1A4	0.493571	0.574356	0.541458	0.436563	0.471758
SULT1B1	0.032902	-0.15004	-0.19724	0.03089	-0.18621
SULT1C1	-0.24893	-0.4963	-0.02033	-0.29864	-0.1573
SULT1C2	-0.01045	-0.30193	-0.57501	-0.19793	-0.27956
SULT1C3	0.204294	0.214589	0.515604	0.122485	0.039601
SULT1E1	-0.98109	-1.0627	-0.82395	-0.89258	-1.17744
SULT2A1	0.365921	-0.68277	-0.55159	-0.45898	-0.09091
SULT4A1	0.30395	0.267884	0.336917	0.170131	0.324944
SUMO1	0.272314	0.269621	0.522426	0.403507	0.042101
SUMO3	0.095552	0.18701	0.325142	0.093788	0.246992
SUNC1	0.366649	0.118988	0.291467	-0.42125	-0.48415
SUOX	0.056174	-0.48039	-0.47388	0.265406	-0.34605
SUPT16H	-0.91918	-0.69264	-0.9662	-0.56292	-0.91827
SUPT3H	-0.0209	0.192773	0.185928	0.004479	0.072915
SUPT3H RUNX2	-0.04219	-0.5338	-0.49851	-0.40159	-0.52601
SUPT4H1 RNF43	-0.79829	0.036737	-0.95742	-0.60772	-0.54343
SUPT5H	-0.49722	-0.50788	-0.95847	-0.61999	-0.70576
SUPT7L SLC4A1AP	-0.34065	-0.39036	-0.5457	-0.35597	-0.51723
SUPV3L1	-0.01152	-0.57643	-0.68385	-0.28053	-0.31841
SURF4	-0.20927	-0.2083	-0.22477	-0.02971	-0.1407
SURF4 C9orf96	0.117417	0.291183	0.121772	0.122863	0.238208
SURF6	-0.05723	0.30399	0.187806	0.073326	0.020127
SUSD1	0.123604	0.063186	0.120794	0.151628	0.011519
SUSD2	0.134582	0.418079	0.517074	0.128125	0.211706
SUSD3	0.244925	0.414496	0.409342	0.302165	0.284177
SUSD4	-0.3821	-0.65428	-0.5404	-0.36499	-0.23937
SUV39H1	-0.20176	-0.35945	-0.19921	-0.28173	-0.35108
SUV39H2	-0.34231	-0.11095	-0.23476	-0.28729	-0.20103
SUV420H1	-0.06158	0.075582	-0.13303	-0.0371	0.020269
SUV420H2	-0.47984	-0.39952	-0.47583	-0.47484	-0.54597
SUZ12	-0.43677	-0.13753	-0.82816	-0.32235	-0.15723
SV2A	-1.1026	-1.17477	-1.46562	-1.08885	-1.06416
SV2B	-0.00058	0.110354	0.207637	0.356763	0.119032
SV2C	-0.41175	-0.1871	-0.71152	-0.22597	-0.44003
SVOPL	0.119692	-0.55994	0.29422	0.337245	0.399918
SYAP1	-0.12785	-0.04191	-0.24417	-0.06925	-0.046

SYCE1	0.217743	0.356439	0.429237	0.34912	0.228171
SYCP1	0.629823	0.472237	1.011805	0.527937	0.778608
SYCP2	0.292466	0.312166	0.490478	0.227306	0.373244
SYCP2L	0.133975	-0.01363	0.464554	0.17716	0.25632
SYCP3	0.473952	0.740119	0.75991	0.478569	0.35848
SYDE1	-0.17784	0.042524	0.033278	0.118829	0.219814
SYF2	-0.56842	-0.5027	-0.73465	-0.63669	-0.48951
SYK	0.066524	0.194136	-0.00132	0.245516	0.171544
SYMPK FOXA3	-0.3432	-0.62286	0.135273	-0.04758	-0.07128
SYNC1	0.50047	0.624882	0.629126	0.480067	0.492496
SYNCRIP	-0.35677	-0.26709	-0.23582	-0.275	-0.24766
SYNGAP1	0.10867	-0.02784	-0.17803	-0.09093	-0.26509
SYNGR2	-0.06325	-0.06569	-0.0807	-0.02611	0.019634
SYNGR3	0.043288	0.117886	0.004997	-0.11393	-0.15705
SYNJ1	-0.34119	-0.12901	-0.40213	-0.08844	-0.17686
SYNJ2	0.145176	0.252687	0.451967	0.257557	0.277911
SYNJ2BP	0.358035	0.558467	0.680096	0.35717	0.544585
SYNPO	-0.18645	-0.39742	0.077995	0.368097	-0.57514
SYNPO2	-0.20484	-0.41121	-0.25509	-0.29784	-0.32346
SYNPO2L	-0.01932	0.280247	0.097586	-0.0183	0.159081
SYP	0.340025	0.205405	0.168606	0.119024	-0.04154
SYPL1	0.27114	0.355774	0.291545	0.364704	0.366282
SYT1	0.20696	0.478152	0.270373	-0.06208	0.268016
SYT10	0.359217	0.084228	0.49497	0.444223	0.282787
SYT12	0.110374	0.314067	0.275136	0.072632	0.317773
SYT13	0.133299	0.088363	0.140285	0.078762	0.224744
SYT14	-0.04846	-0.14188	-0.16755	-0.21651	-0.13564
SYT15	0.05864	0.290681	0.336342	0.237534	0.268664
SYT16	0.248972	-0.96853	0.088758	-0.27595	-0.82027
SYT2	0.052862	0.227262	0.250727	0.006861	0.056693
SYT3	0.308013	-0.32488	0.783926	-0.08132	-0.25528
SYT4	0.150157	0.069957	-0.44243	-0.27605	-0.15961
SYT5	-0.15504	-0.18382	-0.35278	-0.08037	-0.27442
SYT6	-0.1051	-0.00107	0.11133	-0.16959	0.231076
SYT7	0.028304	-0.01354	0.140107	-0.00369	0.059869
SYT8	0.049876	0.111233	0.076105	0.0211	0.093806
SYT9	-0.00709	0.187792	0.340797	0.219324	0.362482
SYTL1	0.166689	0.365816	0.195766	0.244519	0.356191
SYTL3	0.43334	0.514821	0.847328	0.570961	0.434874
SYTL4	0.018103	-0.04901	0.209179	-0.16542	0.193756
SYTL5	0.519755	0.751967	0.4708	0.295462	0.572206
SYVN1	-0.35574	-0.31068	-0.25262	-0.25984	-0.12985
T	-0.09264	-0.08436	0.437536	0.444286	0.3984
TAAR1	0.158084	-0.56492	-0.74713	-0.68334	-0.8199
TAAR5	0.097348	-0.28033	-0.6804	-0.417	-0.6141
TAAR6	0.616605	-0.32491	-0.22902	-0.01114	-0.19569
TAAR8	0.215688	-0.40654	-0.55823	-0.26854	-0.39338
TAC1	0.070337	0.680285	0.432429	0.552299	0.405949
TAC3	0.230237	0.012834	0.01585	0.03914	0.032294
TAC4 AC027801.10	0.196708	-0.86769	-0.01519	-0.21455	-0.32492

TACC1	-0.09341	-0.0222	-0.04369	-0.04062	0.146466
TACR1	0.403376	0.567792	0.380858	0.510082	0.394711
TACR2	0.313475	0.270378	0.508935	0.353354	0.465478
TACR3	0.279312	0.146024	0.037629	0.364	-0.18388
TACSTD1	0.406703	0.460347	0.548773	0.180663	0.351915
TACSTD2	0.80294	0.593161	0.629691	0.443267	0.45171
TADA1L	-0.13253	-0.19221	-0.36755	-0.39308	-0.27583
TADA3L ARPC4	-0.2006	0.055711	-0.31499	-0.1972	0.04864
TAF1	-0.09589	-0.24219	-0.88521	-0.34513	-0.44893
TAF10 TPP1	-0.11223	-0.22077	-0.28675	-0.13723	-0.06436
TAF11 ANKS1A	-0.46945	-0.28912	-0.46632	-0.3557	-0.51458
TAF12	0.140336	0.019816	-0.32063	-0.3845	-0.17201
TAF13	0.034455	0.230424	-0.09205	-0.03695	-0.02155
TAF15	-0.30809	-0.16229	-0.30316	-0.28354	-0.30693
TAF1A	0.001022	-0.22607	-0.4443	-0.22953	-0.02352
TAF1B	0.084695	0.071014	-0.17972	-0.16511	-0.35299
TAF1C	0.131515	0.144572	0.10822	0.085719	0.181863
TAF1L	0.395959	0.838061	0.369158	0.505353	0.508022
TAF2	0.192936	-0.05535	-0.1077	-0.10119	-0.22732
TAF4	-0.01458	0.010845	0.164684	0.069158	0.1769
TAF5	0.427323	0.246999	0.227439	0.119252	0.231728
TAF5L KIAA0133	-0.24838	-0.16701	-0.19727	-0.21728	-0.06884
TAF6 CNPY4	-0.67066	-0.49832	-1.27095	-0.72203	-0.40423
TAF6L	0.142559	0.191504	0.024873	0.013041	0.195834
TAF6L TMEM179B	0.055905	0.054343	-0.03665	0.117198	0.171991
TAF7	-0.30138	-0.18878	-0.53677	-0.00936	-0.30744
TAF7L	0.789535	0.892545	1.042451	0.544423	0.748423
TAF8	0.313145	0.4281	0.412333	0.21403	0.195178
TAF9B	0.185365	0.03341	0.032108	-0.00482	0.030965
TAGAP	0.308849	-0.36982	0.651629	0.289583	-0.26913
TAGLN	-0.12465	0.13578	0.422733	0.118283	0.216717
TAGLN2	-0.32405	-0.32359	-0.61803	0.042626	0.104549
TAGLN3	-0.35272	0.189553	-0.63173	-0.56162	-0.32876
TAL1	-0.54512	0.234331	0.949424	0.404498	0.600152
TAL2	0.471028	0.426501	0.597399	0.556999	0.409241
TALDO1	0.099781	0.317313	0.110313	0.153125	0.205456
TANC1	-0.22309	-0.27015	-0.24634	-0.08716	-0.03163
TANK	0.034704	-0.03696	-0.14807	-0.29493	-0.12478
TAOK1	-0.41578	-0.48312	-0.50865	-0.37074	-0.30084
TAOK3	-0.40951	-0.28517	-0.31466	-0.35032	-0.47464
TAP2	0.391612	0.329191	0.426844	0.271306	0.343857
TAPBP ZBTB22	-0.2266	-0.25643	-0.4627	0.119692	-0.22319
TAPT1	0.222214	0.145047	0.121269	0.067987	0.201652
TARBP1	0.189037	0.106521	0.028761	-0.18164	0.100827
TARDBP	0.202985	0.32468	0.379453	0.261666	0.393856
TARS	-0.37147	-0.7692	-0.63373	-0.76398	-0.48195
TARS2	-0.12596	-0.31874	-1.07275	-0.46474	-0.7346
TARSL2	0.103878	0.418451	0.342817	0.291897	0.245492
TAS1R1 ZBTB48	0.414065	0.574824	0.294934	0.395177	0.314923
TAS1R2	-0.12814	-0.20056	-0.29982	-0.16171	-0.06958

TAS1R3	0.265968	0.088187	0.300598	0.213132	0.363457
TAS2R1	-0.33426	-0.46733	-0.31236	-0.40399	-0.32719
TAS2R10	0.102397	-0.11141	0.061043	-0.31713	0.029776
TAS2R16	-0.20562	-0.94109	-1.46416	-1.03695	-0.98337
TAS2R3	0.481383	0.559576	0.357873	0.067482	0.378001
TAS2R38	0.322591	-0.61975	-0.5785	-0.06947	-0.3765
TAS2R4	-0.05941	-0.17127	-0.16044	-0.28678	-0.16367
TAS2R42	-0.52062	-0.4314	-0.69286	-0.74357	-0.67511
TAS2R60	0.401769	0.627633	-1.11585	0.47535	-0.48826
TAS2R7	-0.38653	-0.24952	-0.74788	-0.44149	-0.70221
TAS2R8	0.295148	-0.04299	0.195326	-0.05086	-0.15681
TAS2R9	-0.74641	-0.90827	-0.91029	-0.84386	-0.99267
TASP1	-0.10618	0.252423	0.242731	-0.02599	-0.13014
TAT	-0.55964	-1.1048	-0.97805	-0.71655	-1.07682
TATDN1 NDUFB9	-0.47552	-0.89909	-0.59224	-0.64595	-0.49636
TATDN2	0.002948	-0.00401	-0.00859	-0.0462	0.091274
TAX1BP1	-0.56457	-0.44643	-0.95225	-0.21878	-0.2912
TAX1BP3 TMEM93	-0.01572	0.064377	0.072056	0.039842	0.189455
TBC1D10A	0.06725	0.175733	0.076107	0.203494	-0.00051
TBC1D10C	-0.30803	-0.18712	-0.07129	-0.44851	-0.20272
TBC1D13	0.202005	0.206771	0.149185	0.039894	0.136925
TBC1D14	0.098182	0.107201	0.006398	0.119837	0.10308
TBC1D15	-0.07747	0.217517	-0.05503	-0.04395	0.144511
TBC1D16	-0.09932	-0.27333	-0.30444	-0.42188	-0.12084
TBC1D19	-0.14173	-0.32885	-0.39184	0.120338	-0.46138
TBC1D2	-0.31157	0.028634	-0.19443	0.051634	-0.05443
TBC1D20	0.288036	0.500867	0.755135	0.249078	0.492856
TBC1D21	-0.35231	-0.56448	-0.91671	-0.87351	-0.71738
TBC1D22A	-0.48924	-0.22735	-0.70036	-0.45752	-0.84928
TBC1D23	-0.02319	0.063153	0.126378	-0.02845	-0.04013
TBC1D24	0.00094	-0.00994	0.142649	-0.01844	-0.07785
TBC1D28	0.233395	0.26823	0.308355	0.207507	0.26665
TBC1D29	0.400413	0.325805	0.381764	0.143387	0.331791
TBC1D2B	0.12796	0.040628	0.0751	-0.0197	0.21543
TBC1D4	-0.20539	-0.1995	-0.21215	-0.13663	-0.19116
TBC1D5	-0.05084	-0.16376	-0.5736	-0.48538	-0.57003
TBC1D7	-0.54995	-0.63288	-0.80805	-0.63975	-0.61743
TBC1D8	0.477362	0.6056	0.781756	0.433657	0.565216
TBC1D8B	-0.00678	-0.32276	0.529174	-0.03739	0.014073
TBC1D9	-0.25532	-0.19675	-0.12213	-0.13232	-0.22117
TBC1D9B	0.192246	0.343533	0.285588	0.243754	0.302441
TBCA	0.103313	0.268526	0.096744	-0.01047	0.244547
TBCC	-0.61993	-0.32549	-0.38998	-0.39357	-0.40743
TBCCD1	-0.34565	-0.17891	-0.26473	-0.15618	-0.20589
TBCE	-0.13284	-0.45141	-0.39115	-0.4198	-0.38302
TBCEL	0.101949	-0.03346	-0.41398	-0.1301	0.112957
TBK1	-0.21627	-0.16955	-0.12995	-0.1333	-0.36256
TBKBP1	-0.08322	-0.04167	0.249251	-0.33921	0.166185
TBL1X	0.14355	0.053066	0.134024	0.066374	0.007109
TBL1XR1	-0.55232	-0.43507	-0.42372	-0.40733	-0.42732

TBL1Y	0.166303	0.303669	0.187556	0.179784	0.058134
TBL2	0.162774	0.234116	-0.1288	0.299775	0.250042
TBL3	0.047755	-0.1267	-0.11305	0.051041	-0.09124
TBPL1	-0.57308	-0.32406	-0.42085	-0.35151	-0.5002
TBPL2	0.16383	0.546839	0.396587	0.104145	0.318429
TBR1	-0.13682	-0.57751	-0.51389	0.102812	-0.30727
TBRG1	-0.23722	0.024716	-0.16064	-0.14861	-0.15156
TBRG4	-0.18503	-0.3501	-0.51486	0.007095	-0.18996
TBX1	-0.00246	-0.05123	0.360471	0.217459	0.161831
TBX10	0.191509	0.142385	0.251042	0.211611	0.295488
TBX15	0.141158	-0.23424	0.4092	0.435736	0.636473
TBX19	0.432146	0.753961	0.6715	0.341021	0.533131
TBX2	-0.06863	-0.08682	-0.12601	-0.08637	-0.11225
TBX20	-0.16484	-0.01055	-0.09746	0.022498	-0.19956
TBX21	0.241207	0.047847	0.186753	0.143575	0.253848
TBX22	-0.04314	-0.42518	0.16954	0.042267	-0.2058
TBX3	-0.40098	-0.56897	-0.24328	-0.25559	-0.30597
TBX4	-0.01419	0.306522	0.564302	0.354518	0.412347
TBX5	-0.56615	-0.39868	0.285978	0.251864	0.272631
TBX6 ZNF688	0.166574	-0.09582	-0.10765	0.014795	0.025134
TBXAS1	0.096835	0.084969	-0.11095	0.021324	-0.1329
TC2N	0.037393	0.033673	0.237232	0.251149	0.235322
TCAP	0.232599	0.369722	0.290049	0.225446	0.195274
TCEA1P2.	0.047195	0.30535	0.126827	0.114846	0.174488
TCEA3	0.194354	-0.20431	0.39596	0.170969	0.307725
TCEAL1	-0.04886	-0.35405	-0.49843	-0.38036	-0.265
TCEAL2	0.152953	-0.03332	0.156126	0.175057	4.09E-05
TCEAL3	-0.05347	-0.30872	-0.24124	-0.20608	-0.36818
TCEAL4	0.341809	-0.22928	-0.43806	-0.2777	-0.22204
TCEAL5	0.155152	0.700659	-0.82628	-0.40482	-0.48703
TCEAL6	0.140548	-0.09402	-0.00046	-0.08223	-0.17703
TCEAL7	-0.2618	0.319771	-0.61863	-0.25994	-0.12628
TCEAL8	-0.13813	-0.40856	-0.96836	-0.56253	-0.67893
TCEB1	-0.36762	-0.3377	-0.42055	-0.3708	-0.53594
TCEB3	0.205461	0.083697	0.161006	0.230014	0.068416
TCERG1	-0.09968	-0.06816	-0.3313	-0.35824	-0.28287
TCERG1L	0.076127	-0.01678	0.008187	0.131745	0.223504
TCF15	0.197098	0.368728	0.341227	0.180042	0.309225
TCF20	0.228081	0.598385	0.618037	0.236479	0.473999
TCF21	0.038649	0.004257	0.542045	0.427602	0.734676
TCF23	0.144375	0.316509	0.307874	0.141602	0.33601
TCF25	-0.09654	-0.19419	-0.21775	-0.07519	-0.00433
TCF3	0.062496	0.174069	0.309349	0.060682	0.069443
TCF4	-0.89865	-0.69215	-0.82088	-0.88718	-0.85267
TCF7	0.161402	0.36844	0.362599	0.204414	0.360055
TCF7L1	-0.09296	-0.04818	-0.10913	-0.09142	-0.1048
TCF7L2	-1.2449	-1.09165	-1.50567	-1.15224	-1.3795
TCFL5	-0.00324	0.069854	0.175818	0.08031	0.084272
TCHHL1	-0.3698	-1.88291	-1.78885	-1.90034	-1.60198
TCHP	-0.45171	-0.26261	-0.48325	-0.35162	-0.59118

TCL1A	0.179253	0.547512	0.593446	0.381221	0.147572
TCN1	0.01614	-0.86743	-0.73481	-0.70328	-0.47171
TCOF1	0.020495	0.142336	-0.00365	-0.06373	-0.41818
TCP1 MRPL18	-0.52587	-0.31726	-0.37477	-0.33512	-0.51567
TCP10L	0.81688	0.049109	1.234097	0.724179	0.615329
TCP10L2	0.350481	0.282727	0.678075	0.365754	0.356285
TCP11	0.306248	0.591215	0.519158	0.273627	0.592606
TCP11L1	-0.09291	0.018614	-0.00269	-0.00089	-0.04476
TCP11L2	-0.23728	-0.14734	-0.01576	-0.22011	-0.21956
TCTEX1D1	0.326869	0.464012	0.665175	0.395842	0.227142
TCTEX1D2	0.063469	-0.09857	-0.13751	-0.02548	-0.06153
TCTEX1D4	-0.33432	-0.31124	0.364483	0.209856	0.002453
TCTN1	0.193244	-0.08838	0.202238	0.015784	0.019894
TCTN2	0.307339	0.362094	0.088458	0.158284	0.126821
TCTN3	0.10146	0.240755	0.376783	0.13659	0.27049
TDG	-0.33851	-0.47971	-0.48693	-0.4075	-0.39289
TDO2	0.443307	0.37134	0.513811	0.028798	-1.22125
TDRD1	0.59679	1.160767	0.931378	0.687412	0.601085
TDRD5	0.26517	0.441156	0.735761	0.41566	0.436365
TDRD6	0.288651	0.408006	0.537083	0.305125	0.496833
TDRD7	-0.36503	-0.46133	-0.35581	0.065948	-0.25158
TDRD9	0.463709	0.431071	0.596315	-0.04127	0.718104
TDRKH	-0.7015	-0.71007	-0.90271	-0.80366	-0.36799
TEAD1	0.42009	0.345867	0.633356	0.238202	-0.18951
TEAD2 DKKL1	-0.83098	-1.03245	-1.09219	-0.63224	-0.70273
TEAD3	-0.14298	-0.13077	-0.09048	-0.02842	-0.16455
TEAD4	-0.25736	-0.19743	-0.14805	-0.21739	-0.17563
TECTA	0.858225	0.78866	0.95314	0.55402	0.638822
TECTB	0.792933	1.062691	0.887208	0.671194	0.646339
TEDDM1	0.509664	0.197045	0.581542	-0.02252	0.04467
TEF	-0.20189	-0.23497	-0.06843	-0.15616	-0.2205
TEGT	-0.47216	-0.4539	-0.74135	-0.52475	-0.51239
TEK	0.162402	0.344691	0.34036	0.11641	-0.55247
TEKT1	0.253361	-0.20092	0.1262	0.430336	0.309882
TEKT2	-0.01181	0.230399	0.213895	-0.00584	0.171321
TEKT2 ADPRHL2	-0.0576	-0.04752	-0.0186	0.098505	0.174428
TEKT3	0.113347	0.16	0.037189	0.303065	0.197365
TEKT4	0.352167	0.401175	0.459016	0.348474	0.49676
TEKT5	0.227377	0.23159	0.145876	0.124657	-0.12455
TELO2	0.212095	0.315188	0.240636	0.182086	0.17766
TENC1	-0.33673	-0.25478	-0.35462	-0.09323	0.005766
TEP1	-0.40757	-0.10927	-0.52043	-0.47286	-0.56512
TEPP	-0.22802	-0.30283	-0.56786	-0.20527	-0.16094
TERF1	0.330186	0.14471	0.002892	-0.12426	0.012295
TERF2	0.109547	0.133917	0.080109	-0.02695	0.076336
TERT	0.152697	0.225366	0.268195	0.216033	0.288617
TESC	0.05854	0.170809	0.293679	0.116194	0.25956
TESK1	-0.2598	-0.19467	-0.11675	-0.17051	-0.14896
TESK2	-0.30724	-0.15971	-0.22332	-0.28918	-0.18008
TET1	-0.12569	-0.4	-0.31159	-0.1359	-0.15454

TET2	0.420428	0.81624	0.624965	0.298708	0.489265
TETRAN	-0.04934	-0.40912	-0.40149	-0.13	-0.27956
TEX10	-0.12008	-0.06916	-0.16004	-0.27624	-0.22652
TEX11	0.584361	0.50972	0.870986	0.419687	0.753229
TEX12	0.630624	0.759301	0.842229	0.580964	0.798975
TEX13B	0.229043	0.533552	-0.02627	0.172061	0.397841
TEX14 RAD51C	0.345866	0.463038	0.575186	0.496766	0.495928
TEX15	0.039446	0.229703	0.359812	0.00602	-0.06597
TEX2	-0.08283	-0.04224	-0.07776	-0.22438	0.001375
TEX261	-0.14449	-0.23242	-0.12417	-0.13308	-0.0551
TEX264	0.190328	0.418398	0.458319	0.150265	0.368223
TEX28 TKTL1	0.280663	0.585242	0.545076	0.414269	0.588783
TEX9	-0.17345	-0.0836	-0.50504	-0.40062	-0.39059
TF	0.066702	0.322442	0.206547	0.204054	0.326618
TFAM	-0.22262	-0.38252	-0.32891	-0.1514	-0.09165
TFAP2B	0.358423	0.235332	-1.08786	0.422934	0.0056
TFAP2C	-0.3024	-0.153	0.149005	0.33433	-0.06198
TFAP2D	-0.79741	-0.30487	-0.24037	-0.29225	-0.25542
TFAP2E	0.147665	0.297935	0.417967	0.281839	0.387686
TFAP4	-0.07972	-0.13877	-0.25913	-0.25082	-0.25786
TFB1M	0.815613	0.727094	0.802981	0.492419	0.489585
TFB2M C1orf71	-0.08016	-0.40454	-0.62735	-0.46088	-0.47503
TFCP2	0.479763	0.075632	-0.33161	0.046122	0.014817
TFCP2L1	-0.05795	0.124681	0.264129	0.092124	0.001586
TFDP1	0.072069	0.192678	0.172105	0.048975	0.140151
TFDP2	-0.1921	-0.0957	-0.53674	-0.26898	-0.71061
TFDP3	0.433169	0.490491	0.600977	0.267954	0.361139
TFE3	0.253682	0.321976	0.456535	0.125826	0.143879
TFEB	-0.03199	0.137998	-0.01217	0.028616	-0.00984
TFEC	-0.71446	-0.80024	-1.02771	-0.66844	-0.88724
TFF1	0.370331	0.528205	0.694979	0.315905	0.537718
TFF2	0.210592	-0.06102	0.189377	0.338564	0.26464
TFF3	0.39143	0.219581	0.592677	0.468727	0.574724
TFG	0.06829	-0.00437	-0.18462	-0.11168	-0.11386
TFIP11	-0.79523	-0.72582	-0.88195	-0.5133	-0.8644
TFPI	0.165647	0.01934	0.527186	0.153125	0.334357
TFPI2	0.334892	0.620986	0.150143	0.545926	0.453771
TFPT PRPF31	-0.38531	-0.62303	-0.73502	-0.24954	-0.9941
TFR2	0.186342	0.152051	0.079529	0.161766	0.214596
TFRC	0.148312	0.268013	0.148589	0.075732	0.225181
TGDS	-0.28815	0.039112	-0.10865	-0.06816	-0.31588
TGFA	-0.15838	0.000229	0.132109	0.222309	0.164983
TGFB1 B9D2	-0.30212	-0.11016	-0.29731	-0.24654	-0.23828
TGFB1I1	0.422614	0.025581	-0.06576	0.473488	0.054486
TGFB2	-0.07072	0.640393	0.864952	0.657754	0.599017
TGFB3	0.189613	0.231394	0.379645	0.036332	0.339218
TGFBI	0.033316	0.612945	0.606346	0.298486	0.490617
TGFBR1	-0.00908	0.163802	-0.00733	-0.17229	0.04284
TGFBR2	0.065718	0.23436	0.318415	0.179416	0.274182
TGFBR3	0.027494	0.34286	0.105278	0.158613	0.160466

TGFBRAP1	0.157527	0.12095	0.14478	-0.00897	0.083009
TGIF2	0.144564	0.261712	0.301944	0.22012	0.174076
TGIF2LX	0.76964	0.615337	0.34527	0.641502	0.599623
TGIF2LY	0.77312	0.685859	0.271213	0.740464	0.533366
TGM1	0.414754	0.2306	0.426079	0.135361	0.234808
TGM2	0.236108	0.07755	0.285623	0.218592	0.127637
TGM3	0.348454	0.398513	0.693809	-0.37143	0.186255
TGM4	0.460793	0.477067	0.337889	0.046036	-0.09923
TGM5	0.171054	0.02378	0.341501	-0.06009	-0.05902
TGM6	0.404172	0.640477	0.516817	0.189405	0.368193
TGM7	0.372249	0.693546	0.696536	0.231815	0.095056
TGOLN2	-0.278	-0.43724	-0.59963	-0.47495	-0.27648
TH	0.181409	0.424679	0.319892	0.206104	0.212852
TH1L	0.014285	0.200256	-0.01336	0.090105	0.196206
THADA	-0.11497	-0.13118	-0.28076	-0.13711	-0.24128
THAP1	-0.13933	0.055144	-0.20944	-0.04552	-0.10336
THAP10 LRRC49	-0.21073	-0.23848	-0.30251	-0.15614	-0.19344
THAP11	0.183811	0.147373	0.21285	0.284758	0.28092
THAP3	0.149527	0.264761	0.431155	0.262745	0.204734
THAP4 ATG4B	-0.0213	0.202767	0.085522	0.184834	0.227861
THAP5 DNAJB9	-0.15578	-0.45904	-0.77322	-0.33565	-0.29748
THAP7	-0.30342	-0.33516	-0.39062	-0.29272	-0.38241
THAP8 WDR62	-0.18336	-0.23285	-0.22598	-0.24991	-0.39641
THBD	0.054117	0.249241	0.467529	0.339895	0.523224
THBS1	-0.118	-0.08621	0.375084	0.139437	0.186211
THBS2	0.208339	0.50014	0.500339	0.441205	0.457609
THBS3 MTX1	-0.50975	-0.67983	-0.74027	-0.70542	-0.48271
THBS4	0.095476	0.431055	0.70052	0.304467	0.38596
THEG	0.234153	0.18303	0.418886	0.379123	0.270009
THEM4	-0.11024	0.411983	0.331648	0.503277	0.242613
THEM5	0.400221	0.463272	0.472804	0.261112	0.587547
THEX1	0.261241	0.377159	0.215168	0.264594	0.165526
THG1L	-0.29926	-0.4884	-0.72015	-0.49217	-0.59882
THNSL2	0.260993	0.431184	0.587679	0.254318	0.360254
THOC1	-0.41902	-0.46295	-0.51969	-0.32873	-0.2655
THOC2	-0.32445	-0.08441	-0.53036	-0.2139	-0.31964
THOC3	-0.10751	-0.07387	-0.24955	-0.22039	-0.44407
THOC4 ANAPC11	0.021351	-0.04955	-0.03259	-0.00904	0.014806
THOC5	-0.13035	-0.30941	-0.66561	-0.33467	-0.29289
THOC7 ATXN7	-0.00539	0.19757	0.03318	0.057229	-0.05868
THPO CHRD	0.210313	0.222718	0.243052	0.148027	0.176334
THRA	-0.03333	0.212842	0.23501	0.151636	0.318352
THRAP3	-0.62526	-0.74351	-1.15962	-0.7034	-0.73428
THRAP4	0.181912	0.422837	0.614783	0.33033	0.17278
THRB	-0.34164	0.108364	-0.16033	-0.14248	0.015951
THRSP	0.153779	0.284166	0.167985	0.114501	0.139527
THSD1	0.057682	0.141424	0.151066	-0.01549	0.094607
THSD3	0.105885	0.179643	0.285526	0.166501	0.282253
THTPA	0.159968	0.061005	0.165105	0.163788	0.029047
THUMPD1	0.17176	-0.02484	-0.14101	-0.18874	-0.15111

THUMPD2	0.227842	0.40564	0.244209	0.207006	0.210547
THUMPD3	-0.27089	0.002115	-0.51677	-0.38366	-0.08344
THY1	-0.18696	0.023391	-0.17572	-0.04634	0.081973
THYN1 ACAD8	-0.2664	-0.11429	-0.31207	-0.11714	-0.26618
TIA1	-0.20797	-0.30355	-0.28196	-0.31767	-0.17091
TIAL1	-0.37749	-0.11567	-0.28306	-0.24394	-0.33182
TIAM1	0.003533	-0.07394	0.039673	0.0091	-0.13847
TICAM1	0.284124	0.506424	0.535205	0.356944	0.409046
TICAM2	-0.5362	-0.43279	-0.42594	-0.41469	-0.59952
TIE1	0.25771	0.224133	0.405031	0.10859	0.213102
TIFA	0.295201	0.448834	0.230807	0.340698	0.632715
TIGD1 EIF4E2	0.002544	-0.1322	-0.22695	-0.2291	-0.09971
TIGD2	0.878604	1.023684	0.625855	0.348686	0.844771
TIGD3	0.264427	0.320466	0.262004	0.341844	0.353635
TIGD4 ARFIP1	-0.80032	-0.84618	-0.75248	-0.5622	-0.66699
TIGD6 AC011382.4	0.010823	0.031974	0.216959	-0.00267	0.121284
TIGD7	0.28884	0.481483	0.361245	-0.05928	0.20441
TIGD7 ZNF75A	0.198262	0.254766	0.604487	0.249522	0.152184
TIMD4	-0.4076	-0.46176	-0.40106	-0.23179	-0.63972
TIMELESS	-0.01353	-0.01718	-0.35827	-0.38187	-0.38411
TIMM10	-0.87613	-1.02505	-1.18354	-0.87859	-0.65511
TIMM13 LMNB2	0.202312	0.047947	0.169191	0.169218	0.180054
TIMM17A	-0.27466	-0.2443	-0.84478	-0.32923	-0.32
TIMM17B PQBP1	0.111975	-0.01302	0.222505	-0.12572	-0.18495
TIMM22	-0.06895	0.187952	0.012572	-0.0653	0.076457
TIMM23 RP11-481A12.5	-0.45794	-0.43166	-0.55118	-0.43417	-0.44622
TIMM44	-0.23466	-0.23084	-0.40338	-0.44731	-0.47866
TIMM50	0.151082	0.219748	0.390706	0.143415	0.116156
TIMM8A	0.162143	0.077545	0.18781	-0.27027	1.248686
TIMM8B SDHD	-0.7738	-0.57587	-1.05745	-0.67874	-0.94151
TIMM9 KIAA0586.	-0.96181	-0.65533	-1.11733	-0.93068	-1.16326
TIMP2	-0.0355	-0.14712	-0.15465	-0.13468	0.190023
TINAG	0.209894	-0.18657	-0.63827	-0.09948	-0.45163
TINAGL1	-0.36107	0.433863	0.362814	0.249414	0.102789
TINF2	-0.00388	-0.02388	-0.1235	-0.10191	-0.17266
TIPARP	0.027188	0.128659	-0.03426	0.037661	-0.09323
TIPIN	0.219232	0.204419	-0.24163	-0.1424	-0.11509
TIPRL	0.120612	-0.07925	-0.2735	-0.13908	-0.1277
TIRAP	0.17056	0.195708	0.084458	0.098736	0.175357
TITF1	-0.32025	-0.02318	0.220245	0.380211	0.16293
TJAP1	-0.61054	-0.4588	-0.9623	-0.66557	-0.97735
TJP1	-0.02996	0.120357	-0.0162	0.278278	0.135218
TJP2	-0.11525	-0.36764	-0.31066	-0.25819	-0.36052
TK1 AFMID	0.030665	0.030569	-0.01577	-0.07674	-0.0106
TK2	0.275766	0.276588	0.308661	0.175469	0.391087
TKT	0.204877	0.057177	0.192053	0.114858	0.298688
TKTL2	0.654514	0.42277	0.711204	0.459062	0.348285
TLCD1 NEK8	-0.27243	-0.21452	-0.46365	-0.18551	-0.15052
TLE1	0.117035	0.197903	0.301463	0.061788	0.272086
TLE2	-0.22726	-0.33299	-0.12389	-0.19771	-0.05867

TLE3	-0.19471	-0.25014	-0.1818	-0.19803	-0.24544
TLE4	-0.44947	-0.39572	-0.48891	-0.3554	-0.38249
TLE6	-0.26284	-0.59054	-0.77119	-0.70428	-0.71116
TLK1	-0.42283	-0.43165	-0.49667	-0.3944	-0.37063
TLK2	0.151567	0.023399	0.06528	-0.04771	0.054831
TLL1	-0.1352	-0.4466	-0.30808	-0.41173	-0.41368
TLL2	0.056795	0.10221	0.057429	-0.05358	0.044339
TLN1 CREB3	-0.67142	-0.61392	-0.49366	-0.57545	-0.74872
TLN2	0.591817	0.577264	0.691077	0.391314	0.49159
TLR1	0.519571	0.605237	0.548011	0.200606	0.268773
TLR10	0.501115	0.767198	0.789455	0.3121	0.075256
TLR3	0.582139	-0.51046	0.824866	0.219314	-0.30306
TLR4	-0.44098	0.002139	0.181451	0.19604	-0.00648
TLR5	0.137698	0.336007	0.045945	0.014773	0.097008
TLR6	0.275286	0.417352	0.304666	-0.01649	-0.07033
TLR7	-0.40103	-0.35177	-0.38028	-0.25189	-0.23918
TLR8	0.687313	0.832569	0.719641	0.615988	0.664539
TLX2	-0.09497	-0.19381	-0.27418	-0.2682	-0.04441
TLX3	-0.11989	-0.02818	-0.11181	0.027613	0.541814
TM2D1	-0.37403	-0.14839	-0.54904	-0.39403	-0.22215
TM2D2 ADAM9	-0.44979	-0.33404	-0.48166	-0.28423	-0.55711
TM2D3	0.175221	-0.06178	-0.11905	-0.13833	-0.00605
TM4SF1	0.011566	0.368431	0.592786	0.46174	0.551064
TM4SF18	0.34862	0.58028	0.720256	0.280504	0.393031
TM4SF19	0.537383	0.726967	0.80461	0.505591	0.623646
TM4SF20	0.423642	0.506316	0.475696	-0.00914	0.127912
TM4SF4	0.275555	-0.45372	-0.35481	-0.19638	-1.12079
TM4SF5	0.210613	-1.32614	-0.02445	-0.34067	-0.52857
TM6SF1	0.166746	0.041357	0.300133	0.137012	0.253567
TM7SF3	0.150987	0.106205	0.311072	-0.01522	0.104383
TM7SF4	0.471983	0.481345	0.675899	0.384251	0.426854
TM9SF1	-0.02652	-0.07904	-0.11231	-0.02913	-0.03364
TM9SF2	-0.10263	-0.11465	-0.1221	-0.13855	-0.2203
TM9SF3	-0.10122	-0.16425	-0.2119	-0.11666	-0.23763
TM9SF4	0.435776	0.59272	0.823903	0.420034	0.52812
TMBIM4	0.177261	-0.23263	-0.04959	0.104484	0.057138
TMC1	0.791391	0.782171	0.591717	0.480824	0.658446
TMC2	0.161544	0.403876	0.538248	0.231649	0.414001
TMC4 LENG4	0.28811	0.182413	0.439897	0.283428	0.238961
TMC5	0.667837	0.404399	0.697681	0.302713	0.480312
TMC6 TMC8	0.252123	0.351291	0.46286	0.202159	0.374745
TMC7	0.428312	0.494228	0.561204	0.332412	0.239537
TMCC2	-0.63708	-0.55377	-0.61826	-0.65216	-0.66292
TMCC3	0.066524	0.224825	0.365423	0.076478	0.092805
TMCO1	-0.59526	-0.52782	-1.21913	-0.74159	-0.6365
TMCO2	-0.18092	-0.23433	-0.15965	-0.2549	-0.16557
TMCO4	0.24751	0.414636	0.460664	0.28609	0.37704
TMCO5	-0.02453	0.134962	-0.15669	-0.20284	-0.63845
TMCO7	0.500009	0.636601	0.576267	0.260736	0.698671
TMED1	0.245139	0.212294	0.104233	-0.11365	0.262982

TMED10	-0.43025	-0.28982	-0.62861	-0.27919	-0.55982
TMED2	-0.27449	-0.43988	-0.42817	-0.29457	-0.36031
TMED3	0.147494	-0.17553	0.138189	-0.07606	-0.19558
TMED4	0.05248	-0.007	-0.27389	-0.00766	0.053272
TMED5 CCDC18	-0.27555	-0.3644	-0.29621	-0.27568	-0.10188
TMED6	0.61808	0.67376	0.917046	0.434635	0.552006
TMED8 C14orf174	-0.1646	-0.23523	-0.29001	-0.16575	-0.28709
TMED9	0.138744	-0.41904	-0.70187	0.024405	-0.10182
TMEFF1 AL354917.8	0.510667	0.542703	0.528667	0.186981	0.099401
TMEFF2	-0.51765	-0.21633	-0.40899	-0.33195	-0.02594
TMEM1	-0.00359	-0.00538	0.091259	0.071068	-0.05309
TMEM10	0.355346	0.322624	0.580032	0.4236	0.014354
TMEM100	-0.50511	-1.33158	-1.40034	-1.77696	-1.86059
TMEM101	-0.00653	-0.17372	-0.09438	0.347374	-0.13201
TMEM102	-0.21173	-0.01953	-0.14343	0.116112	0.338343
TMEM103	0.103635	0.198613	0.0353	0.104871	0.147167
TMEM105	0.273539	0.358045	0.36971	0.297298	0.390436
TMEM106B	-0.48348	-0.76394	-0.76466	-0.61648	-0.55613
TMEM106C	-0.25632	-0.00845	-0.59662	-0.23219	-0.32289
TMEM107	-0.08234	0.082337	-0.24137	0.074897	0.110265
TMEM108	0.003192	-0.02492	-0.42906	0.047263	-0.14723
TMEM109	-0.53013	-0.42512	-0.50137	-0.37529	-0.32421
TMEM11	-0.09758	0.184513	0.140702	0.003392	0.237542
TMEM111 AC022007.5	-0.44349	-0.27586	-0.55676	-0.29484	-0.21841
TMEM112B NCAPH2	-0.20692	-0.08295	-0.30144	-0.20265	-0.07069
TMEM115	0.206004	0.115692	-0.06885	0.188527	0.14751
TMEM116 ERP29	-0.0734	-0.08035	-0.35044	-0.20071	0.006677
TMEM117	0.095971	0.065398	0.213845	-0.04073	0.052852
TMEM119	0.081367	-0.29265	-0.36305	0.025032	-0.12853
TMEM120A	0.28326	0.211997	0.25638	0.115413	0.011616
TMEM121	-0.04585	-0.26743	-0.24277	-0.0843	-0.25816
TMEM123	-0.45794	-0.19439	-0.43149	-0.09444	-0.35361
TMEM125	0.401854	0.598257	0.352323	0.377775	0.208491
TMEM126A	-0.69663	-0.37449	-0.88056	-0.69616	-0.93334
TMEM126B	-1.79925	-0.87514	-1.61257	-1.2698	-1.37787
TMEM127 WDR39	-0.03619	-0.02639	-0.00127	-0.08963	0.133386
TMEM128	-0.09383	-0.17334	-0.06983	0.069232	-0.15568
TMEM129 TACC3	-0.05626	-0.00346	0.099009	0.019351	0.013641
TMEM130	-0.24197	0.20313	-0.03929	-0.08148	0.155061
TMEM132A	0.003268	0.124413	0.072331	0.034929	0.086687
TMEM132D	0.047613	0.345666	0.165439	0.310049	0.311141
TMEM133	0.385662	0.55865	-0.24849	0.021297	0.05609
TMEM135	-0.24929	-0.02118	-0.18263	-0.13284	-0.27465
TMEM136	-0.05606	0.088588	-0.02174	-0.0677	-0.0413
TMEM139	0.590957	0.56546	0.753007	0.649444	0.422568
TMEM139 CASP2	0.205749	0.286298	0.287423	0.293149	0.305447
TMEM140	0.486986	0.525192	0.543796	0.250907	0.124324
TMEM143 SYNGR4	-0.20272	0.126247	-0.14638	0.030823	0.079305
TMEM144	0.331438	0.661541	0.947718	0.540214	0.379637
TMEM145	-0.04039	-0.07987	-0.1532	-0.13445	-0.21814

TMEM149 U2AF1L4	0.296788	0.735154	0.599179	0.493594	0.675716
TMEM14A	0.370648	0.279814	0.358506	0.307721	0.450886
TMEM14C	-0.12725	0.01549	-0.43061	-0.2829	-0.27398
TMEM14D	0.126978	0.078856	-0.4124	-0.1063	-0.10311
TMEM150	0.062546	0.007579	-0.10406	0.044342	0.089069
TMEM151A	-0.03306	-0.00032	0.055712	0.040723	0.036313
TMEM154	0.244452	0.595981	-0.02078	0.374246	0.25906
TMEM155	0.005026	-0.15179	-0.13546	-0.04203	0.247889
TMEM156	0.105829	0.067786	0.294329	-0.05038	0.1072
TMEM157	0.172181	-0.31705	0.097352	-0.09809	-0.12831
TMEM160	0.137114	-0.53944	-0.39181	-0.20773	-0.15514
TMEM161A	-0.22832	-0.12046	-0.55469	-0.32427	-0.44421
TMEM161B	-0.49187	-0.74499	-0.84666	-0.48675	-0.52952
TMEM162	0.226458	0.120417	0.133118	0.000672	0.051971
TMEM163	0.158291	0.133242	0.321735	0.164171	0.378798
TMEM164	-0.12318	-0.34716	-0.18876	-0.33273	-0.17263
TMEM165	-0.1747	-0.12592	-0.14763	-0.16133	-0.16071
TMEM166	0.589123	0.697087	0.642465	0.218891	0.252459
TMEM167 XRCC4	-0.27144	-0.41786	-0.30212	-0.22849	-0.21747
TMEM168	0.227907	0.016574	-0.34567	0.242421	0.019831
TMEM16B	0.342597	-0.05909	-0.80458	-0.40543	-0.12336
TMEM16D	-0.33568	0.461051	-0.62351	0.056784	-0.1784
TMEM16E	-0.44834	-0.33494	-0.67408	-0.47469	-0.26497
TMEM16G	0.109593	0.311175	0.316824	0.198881	0.254259
TMEM16H	0.215148	0.032959	0.144503	0.207152	0.148222
TMEM16J	0.070935	-0.03077	0.11843	0.145133	0.145763
TMEM16K	-0.51114	-0.24895	-0.54027	-0.48356	-0.48832
TMEM17	-0.135	-0.16849	-0.21568	-0.04888	0.005335
TMEM170	0.100107	-0.13611	0.128173	0.01739	-0.06904
TMEM171	-0.08241	0.193178	0.28883	0.128886	0.079593
TMEM173	0.029975	0.802781	0.728137	0.408836	0.464382
TMEM176B TMEM176A	0.025146	0.227708	0.396529	0.266295	0.395214
TMEM177	-0.07086	-0.18758	-0.39679	-0.35762	-0.02767
TMEM178	-0.41266	0.155591	0.102374	0.121015	-0.15791
TMEM179	0.212019	0.360241	0.251094	0.002173	0.389213
TMEM18	-0.2584	-0.0513	-0.13933	-0.2636	0.005677
TMEM180	0.246414	-0.0167	0.237158	-0.06678	-0.04446
TMEM182	0.37085	0.606994	0.598871	0.169155	0.391664
TMEM183A	-0.00662	-0.08792	-0.29723	-0.17329	-0.12113
TMEM184B	0.249766	0.42316	0.108765	0.057775	0.149897
TMEM187	-0.09635	-0.0993	-0.15787	-0.04246	-0.11728
TMEM19	0.204562	0.094627	0.068188	-0.0395	0.015692
TMEM190	0.365633	0.546263	0.430812	0.246653	0.6461
TMEM194	-0.81206	-0.70725	-1.09104	-0.76574	-0.99334
TMEM195	0.362169	0.234572	0.243692	-0.27692	0.248977
TMEM196	-0.19448	0.142172	0.502329	-0.09214	0.119704
TMEM2	-0.39732	-0.17503	-0.15378	-0.19157	-0.06473
TMEM20	0.086549	0.149913	0.182608	0.036769	0.05718
TMEM200B	0.035462	-0.03805	0.237802	0.259496	0.16551
TMEM201	0.198552	0.053552	0.143481	0.173934	0.267711

TMEM203 NDOR1	0.122845	-0.32229	-0.35141	-0.14008	-0.35621
TMEM205 AC024575.6	-0.23502	-0.11354	-0.84395	-0.14957	-0.36737
TMEM206	0.382666	0.486561	0.352159	0.348052	0.417622
TMEM207	-0.22131	-0.69953	-0.24368	-0.26434	-0.22433
TMEM209 C7orf45	0.307574	0.271755	0.316249	-0.08295	0.037047
TMEM22	-0.10293	-0.35692	-0.29997	-0.28818	-0.26117
TMEM26	0.227808	0.593306	0.61964	0.494873	0.549214
TMEM27	-0.05646	0.195979	0.189412	0.162489	0.184926
TMEM28	0.161632	0.244491	0.407453	0.255836	0.273709
TMEM29	0.613011	0.098156	0.444305	0.37232	-0.11685
TMEM30A	-0.53895	-0.63965	-0.58119	-0.56313	-0.54842
TMEM30B	-0.01138	0.301151	0.387569	0.333521	0.438285
TMEM32 RP11-274K13.2	-0.20814	-0.80483	-0.35071	-0.51654	-0.78488
TMEM33	0.276252	-0.05289	0.529709	0.152075	0.317359
TMEM34	-0.4565	-0.41109	-0.67568	-0.43445	-0.35427
TMEM35	0.202306	0.330098	-0.16946	-0.1296	0.2882
TMEM37	0.151166	0.427462	0.55556	0.364703	0.510608
TMEM38A	-0.19004	-0.16666	-0.41361	-0.00401	-0.39161
TMEM38B	-0.01333	-0.21128	-0.3749	-0.38388	-0.19712
TMEM39A	0.004467	-0.30944	-0.05991	-0.40973	-0.46341
TMEM39B	-0.29036	-0.32142	-0.46046	-0.32622	-0.13783
TMEM40	0.184092	0.160568	0.339153	0.099901	0.126607
TMEM41A	-0.03214	-0.05329	0.144	-0.15966	-0.34946
TMEM41B	0.01113	0.093492	-0.0597	0.020827	0.214701
TMEM44	-0.07267	-0.07056	0.037291	-0.17736	-0.25909
TMEM45A	-0.26816	0.204395	-0.26295	0.2219	-0.31109
TMEM45B	0.106668	0.131399	0.17989	0.128778	0.144851
TMEM46	0.113831	0.190539	0.403038	0.212514	0.313905
TMEM47	0.065395	0.069291	0.066667	0.16925	0.075759
TMEM48	-0.56852	-0.20726	-0.4615	-0.40791	-0.35601
TMEM5	-0.17766	-0.24089	-0.40635	-0.10927	-0.30526
TMEM50A	-0.45405	-0.5875	-0.70904	-0.38387	-0.33834
TMEM50B	-0.07845	-0.02063	0.002417	0.030091	-0.01206
TMEM52	0.133753	0.239903	0.01318	0.135554	0.209792
TMEM53 AL122004.17	-0.04786	-0.18701	-0.27298	-0.18	0.073681
TMEM54	0.183734	-0.01139	0.270265	0.294144	0.346069
TMEM55A	-0.18441	-0.56009	-0.52992	-0.42536	-0.41566
TMEM56	0.03402	0.078359	0.01179	-0.13239	0.057319
TMEM57	-0.27127	-0.19583	-0.24968	-0.1741	0.061397
TMEM58	-0.24217	-0.21973	-0.28169	0.169296	0.175246
TMEM59 C1orf83	-0.38792	-0.39417	-0.34558	-0.43004	-0.18946
TMEM59L	-0.23478	-0.0673	-0.1443	-0.04074	-0.14076
TMEM60 PHTF2	-0.56456	-0.55382	-0.70835	-0.32922	-0.44128
TMEM61	0.335836	0.438213	0.438672	0.413255	0.338175
TMEM62	0.326873	0.415607	-0.0526	0.419432	0.392841
TMEM62 CCNDBP1	-0.01341	-0.12119	-0.06995	-0.09617	-0.25512
TMEM63A	0.037781	0.032893	0.318512	0.32209	-0.00368
TMEM63C	-0.14694	-0.14869	-0.28388	-0.21063	-0.23812
TMEM64	-0.00646	0.038931	0.058321	0.020304	0.057768
TMEM65	-0.05202	-0.21863	-0.01192	-0.13955	-0.12587

TMEM66	-0.04491	-0.15413	-0.21009	-0.2311	-0.1887
TMEM67	0.074836	-0.68209	-0.75219	-0.70607	-0.84132
TMEM68 TGS1	-0.64313	-0.77854	-0.56698	-0.63619	-0.60236
TMEM69	-0.87222	-1.77021	-2.4079	-1.32171	-1.31068
TMEM70	0.066996	0.068423	-0.0813	-0.07647	0.051888
TMEM71	-0.12101	0.233669	0.767967	0.33938	-0.11519
TMEM74	-0.00128	0.099335	-0.22354	-0.18806	-0.21081
TMEM77 CEPT1	-0.23987	-0.11633	-0.18812	-0.38929	-0.19418
TMEM8	0.066511	0.134169	0.048257	0.062017	0.138624
TMEM82	0.158434	0.253242	0.148002	0.109689	0.109105
TMEM85	0.106481	0.1055	-0.4912	0.42947	0.035045
TMEM86A	0.522601	0.430941	0.496959	0.257817	0.262809
TMEM86B SAPS1	0.081437	0.36838	0.383546	0.323875	0.487662
TMEM87A GANC	-0.42922	-0.53203	-0.61301	0.054886	-0.41554
TMEM87B	-0.14135	-0.18358	-0.17888	-0.18978	-0.1597
TMEM89	0.116326	0.134741	0.312301	0.190609	0.031417
TMEM9	0.070631	0.095935	0.109583	0.057054	0.173914
TMEM92	-0.05938	0.207043	0.495033	0.128474	0.155575
TMEM97	-0.25917	-0.11517	-0.33749	-0.28237	-0.13482
TMEM98	0.046034	0.158507	0.067756	0.098704	0.206351
TMEM9B	0.059482	-0.02483	0.025319	0.166183	0.173907
TMEPAI	0.097122	0.333308	0.275277	0.187753	0.276213
TMF1	-0.67648	-1.05463	-1.02581	-0.87529	-1.06535
TMIE	-0.15492	-0.11139	-0.14564	-0.0995	-0.09476
TMIGD1	0.677442	0.684684	0.681914	0.287685	0.318479
TMIGD2 FSD1	0.268799	0.186641	0.227309	0.153832	0.281008
TMLHE	0.281183	0.220028	0.114905	0.022687	0.266751
TMOD1	0.446112	0.785026	0.854814	0.203432	0.764819
TMOD2	-0.012	0.175653	0.101641	0.067472	0.239018
TMOD3	-0.23745	0.280521	0.090832	0.072727	-0.05266
TMOD4 VPS72	0.430583	0.466015	0.403755	0.184962	0.153367
TMPO	-0.51399	-0.38305	-0.52193	-0.29197	-0.48867
TMPRSS11A	-0.06747	-0.48026	-0.43653	-0.42678	-0.45867
TMPRSS11B	0.623953	-0.70533	-0.61225	-0.32857	-0.07427
TMPRSS11D	-0.26538	-0.47159	-0.50259	-0.54735	-0.72434
TMPRSS11E	-0.25536	-0.58928	-0.38979	-0.51933	-0.75319
TMPRSS12	0.360084	0.540952	0.6741	0.435814	0.615747
TMPRSS13	0.232695	0.260276	0.582172	0.257778	0.258619
TMPRSS2	-0.08109	-0.19946	0.186258	0.150333	0.138026
TMPRSS4	0.163911	0.088809	0.400586	0.158747	0.420833
TMPRSS5	-0.42904	-0.0724	-0.29172	-0.27405	0.023176
TMPRSS6	0.387025	0.140632	0.644435	-0.01464	0.06455
TMPRSS9	0.774251	0.05324	0.835636	0.482899	0.899835
TMSB10	-0.24607	-0.26972	-0.41528	-0.23055	-0.07212
TMSB4X	0.150105	0.018492	0.509746	-0.10746	0.374702
TMSB4Y	0.057959	0.168348	0.238441	0.324981	0.108012
TMSL3	0.395329	0.608111	0.565284	0.335796	0.521994
TMSL8	0.373093	-0.12954	0.072789	-0.14482	-0.32174
TMTC1	0.188513	0.354148	0.280446	0.104352	0.15722
TMTC2	-0.60027	-0.6064	-0.48269	-0.43599	-0.54152

TMTC4	0.409698	0.473237	0.555408	0.252786	0.46904
TNC	-0.55715	-0.39231	-0.68018	-0.36095	-0.39922
TNF	0.233994	0.572499	0.435916	0.431748	0.624854
TNFAIP2	0.251376	0.275287	0.375287	0.305741	0.374882
TNFAIP3	-0.28526	0.157997	0.24668	0.403977	-0.04405
TNFAIP6	-0.00843	-0.46101	-0.4348	-0.46552	0.053151
TNFAIP8L1	0.205395	-0.09144	-0.33818	-0.13101	-0.10329
TNFAIP8L2	0.201545	0.197061	0.095705	0.020673	-0.00338
TNFAIP8L3	0.228049	-0.54067	-0.16953	-0.24829	-0.56083
TNFRSF10A	0.31331	0.396206	0.322473	0.600371	0.320152
TNFRSF10B	-0.11636	0.307756	0.208462	-0.01646	0.299251
TNFRSF10C	0.2503	0.65309	0.4785	0.327057	0.429815
TNFRSF10D	0.291313	0.399703	0.604446	0.393635	0.46786
TNFRSF11A	0.084027	0.261186	0.213707	0.162763	0.349549
TNFRSF11B	0.010474	-0.04704	-0.2494	0.20172	0.040253
TNFRSF13B	0.085222	0.373468	0.226104	0.220661	0.341991
TNFRSF13C	-0.07396	-0.03406	-0.03346	0.032838	-0.04073
TNFRSF14	0.36278	0.329527	0.24582	0.071968	0.356588
TNFRSF17	0.760061	0.864075	0.639302	0.03333	0.106874
TNFRSF18	0.008401	0.012722	0.349283	0.124211	0.153264
TNFRSF1A	-0.36196	-0.29967	-0.35985	-0.16454	0.03212
TNFRSF1B	0.365313	0.414879	0.541853	0.338558	0.478581
TNFRSF21	-0.37559	-0.55279	-0.4866	-0.52964	-0.75203
TNFRSF25 PLEKHG5	0.132972	0.050888	0.304496	0.10746	0.160746
TNFRSF4	0.238697	0.207222	0.241631	0.096342	0.297458
TNFRSF9	0.462367	0.395012	0.677025	0.421283	0.473887
TNFSF10	0.007425	-0.62879	0.000306	-0.0171	-0.51333
TNFSF13 EIF4A1	-0.37898	-0.10896	-0.29435	-0.25105	-0.03815
TNFSF13B	-0.09057	-0.06546	-0.19007	-0.25863	-0.50364
TNFSF14.	0.373385	-0.09788	0.235317	0.261534	0.212268
TNFSF15	0.049389	-0.2078	0.22599	-0.17782	-0.84336
TNFSF18	-0.45688	-0.48458	-0.05734	-0.66567	-0.21539
TNFSF4	0.381378	0.56373	0.777867	0.284427	0.436646
TNFSF8	0.492133	0.58354	0.835403	0.439515	0.22427
TNFSF9	-0.35739	-0.61184	-0.43091	0.192601	-0.50709
TNIK	-0.59668	-0.54304	-0.4855	-0.38387	-0.50462
TNIP1	-0.17523	-0.00299	0.016368	0.022625	-0.10451
TNIP2	0.076066	0.194205	0.184987	0.062594	0.053957
TNIP3	0.300139	0.606601	0.422528	-0.02101	-0.03947
TNK1	0.176285	-0.00013	0.351844	0.330444	0.244226
TNKS	-0.42122	-0.36478	-0.38728	-0.35719	-0.6995
TNKS2	-0.04052	-0.11781	-0.01345	-0.13028	0.107817
TNMD	-0.62905	-0.60572	-0.66071	-0.33896	-0.45394
TNN	0.14396	0.181978	0.366503	0.148649	0.187364
TNNC1 NISCH	0.023276	0.221967	0.076821	0.175007	0.118525
TNNC2	0.423352	0.4417	0.335918	0.35512	0.348902
TNNC2 SNX21	0.06175	0.208603	-0.22297	-0.00824	0.175306
TNNI1	0.006269	0.064232	-0.00633	-0.11499	0.073408
TNNI2	0.176887	0.393945	0.429344	-0.00288	0.236657
TNNI3	0.104631	0.096363	0.303835	0.091519	0.090332

TNNI3K	-0.09071	0.040136	-0.07315	-0.47789	0.073927
TNNT1	-0.24426	-0.80162	0.337817	-0.44191	-0.33258
TNNT2	0.078599	-0.22378	-0.17856	-0.16429	-0.17099
TNNT3	0.320001	0.546087	0.477633	0.228683	0.336479
TNP1	0.312643	0.589306	0.532238	0.378188	0.430011
TNP2	0.339341	0.276918	0.144813	0.212486	0.217671
TNPO2	-0.44296	-0.50965	-0.58978	-0.45732	-0.57383
TNPO3	-0.4967	-0.19711	-0.8712	-0.36646	-0.56457
TNR	-0.40779	-0.76305	-0.27097	-0.41844	-0.56717
TNRC4 AL589765.19	-0.48002	-0.51865	-0.84311	-0.69225	-0.64165
TNRC6C	0.691138	0.445061	0.48432	0.031762	-0.12417
TNS1	0.101028	-0.35501	-0.51018	-0.09867	-0.36587
TNS3	0.378767	0.678749	0.465243	0.494843	0.394449
TNS4	0.135804	0.140949	0.193684	0.092624	0.025972
TOB1	0.057205	0.342988	0.099941	0.963049	-0.47687
TOB2	-0.34147	-0.19696	-0.17529	-0.42437	-0.49149
TOLLIP	0.101844	0.185197	0.320978	0.152998	0.296131
TOM1	0.119771	-0.49233	-0.18986	-0.1557	-0.08643
TOM1L1	-0.36953	-0.48788	-0.61811	0.369965	0.007619
TOM1L2 LRRC48	-0.21249	-0.19096	-0.66221	-0.4106	-0.15406
TOMM20	0.240338	0.300753	0.274346	0.257952	0.263013
TOMM22	0.236979	0.058685	-0.00428	-0.01405	-0.0337
TOMM34	-0.56472	-0.47722	-0.36218	-0.35972	-0.34713
TOMM40	-0.12815	-0.09819	-0.27259	-0.22886	-0.31373
TOMM7	-0.03089	-0.10189	-0.29923	-0.16984	-0.16858
TOMM70A LNP1	-0.95125	-1.08989	-1.00171	-0.81636	-0.95373
TOP1	-0.23652	-0.17318	-0.22652	-0.24826	-0.20188
TOP1MT	0.134529	0.165174	0.231386	0.066762	0.101569
TOP2B	-0.04899	-0.02546	-0.01467	-0.01424	0.169179
TOP3A SMCR8	-0.25204	-0.13007	-0.43935	0.00331	-0.15765
TOP3B	0.088447	-0.14359	-0.03321	-0.16277	-0.05626
TOPBP1	-0.31943	-0.38676	-0.48124	-0.35959	-0.44768
TOPORS	-0.19675	-0.28161	-0.44644	-0.2829	-0.29887
TOR1A	0.22946	0.059282	0.319855	-0.10949	0.015552
TOR1AIP1	-0.14508	-0.28372	-0.54245	-0.50271	-0.41427
TOR1B	-0.02565	-0.01018	-0.01929	-0.10278	-0.05218
TOR3A	0.103609	0.46121	0.225698	0.216001	0.313427
TOX AC087698.5	-0.53184	-0.47022	-0.6292	-0.39624	-0.48407
TP53 WDR79	-0.43347	-0.10896	-0.39246	-0.40974	-0.25971
TP53AP1. CROT	-0.1767	-0.29398	-0.53479	-0.39843	-0.28065
TP53BP1 MAP1A	-0.38388	-0.36391	-0.44398	-0.28926	-0.61013
TP53BP2	-0.08719	-0.01031	-0.01053	-0.13336	-0.04514
TP53I11	0.160618	0.244839	0.215414	0.120812	0.210752
TP53I3	0.138796	-0.09281	0.012819	-0.08308	-0.14356
TP53INP2	-0.33072	-0.17003	-0.36261	-0.22861	-0.36751
TP63	0.593105	-0.98712	-0.71187	0.241314	-0.01181
TPBG	-0.75194	-0.68576	-0.8951	-0.37536	-0.42543
TPCN2	0.14538	0.115558	0.129302	0.110904	0.197929
TPD52	0.116671	0.498473	0.180648	-0.15146	0.349272
TPD52L1	-0.11652	0.15268	0.213808	0.229771	0.222283

TPD52L3	0.470023	0.733607	0.745031	0.495254	0.184462
TPH1	-0.06632	-0.47817	-0.06227	-0.20124	0.004328
TPH2	0.061643	0.259035	-0.12639	0.506428	-0.77856
TPI1	0.155865	0.305019	0.220864	0.151588	0.125115
TPK1.	0.097403	0.376832	0.221788	0.017456	0.175164
TPM2	0.118455	0.214545	0.165951	0.166097	0.055335
TPMT AOF1	-0.33182	-0.32004	-0.40543	-0.22388	-0.31163
TPO	0.102168	0.521965	0.836204	0.56989	0.717863
TPP1	-0.43262	-0.73404	-0.93835	-0.60895	-0.5404
TPP2	-0.33337	-0.23283	-0.25935	-0.21332	-0.17636
TPPP	0.002528	0.057948	-0.12418	-0.02793	-0.11503
TPPP2	0.285826	0.474194	0.235419	-0.36672	0.127277
TPPP3	0.126676	0.106663	0.197131	0.073475	0.118563
TPR C1orf27	-0.2935	-0.46397	-0.29594	-0.32732	-0.2352
TPRG1	-0.49212	-0.6689	-0.63186	-0.49472	-0.55855
TPRG1L	-0.1583	-0.04426	-0.10075	0.017223	-0.05517
TPRKB	-0.07271	-0.19933	-0.17524	0.06749	0.131717
TPRX1	0.348781	0.197268	0.227137	0.347498	0.380267
TPSB2	0.302713	-0.25281	-0.16931	0.330012	0.19845
TPSD1	0.053463	-0.18989	-0.03482	0.302836	0.240095
TPSG1	0.207446	-0.27483	-0.22182	0.279388	0.313864
TPST1	0.128413	0.053272	-0.15512	0.179777	0.041543
TPT1 AL627107.32	-0.31376	-0.15212	-0.26146	-0.21421	-0.19129
TPTE	0.051415	0.043608	0.171837	0.108143	0.164707
TPTE2	-0.0053	-0.38395	-0.38654	-0.64776	-0.29641
TPX2	-0.12901	-0.02782	-0.35646	-0.07203	-0.28838
TRABD	0.00013	-0.04554	0.120628	0.073733	0.076805
TRADD	0.183588	0.501221	0.617254	0.183841	0.410882
TRADD FBXL8	-0.18399	-0.17525	-0.28788	-0.19875	-0.28508
TRAF1	-0.03119	0.093983	-0.02722	-0.12875	0.021525
TRAF2	0.089897	0.099717	0.03968	0.104347	0.084599
TRAF3	0.056696	0.120539	0.18412	0.067316	0.186207
TRAF3IP1	0.118575	0.185362	0.041815	-0.00325	0.17591
TRAF3IP2	0.059556	0.479125	0.603496	0.475943	0.450087
TRAF3IP3	-0.05053	0.19302	-0.00382	-0.09933	-0.10074
TRAF4	-0.06958	0.075433	0.105111	0.033421	0.072192
TRAF6	-0.38404	-0.15173	-0.2735	-0.08383	-0.06696
TRAFD1	-0.06658	-0.18966	-0.14258	-0.22632	-0.14171
TRAIP	-0.32483	-0.1599	-0.72598	-0.1209	-0.21908
TRAK1	0.309085	0.724472	-0.34909	0.512104	-0.14584
TRAK2 ALS2CR2	0.142993	-0.01576	-0.01223	-0.1464	-0.1431
TRAM1	0.29573	0.41953	0.556814	0.275039	0.462467
TRAM1L1	0.214202	0.016983	0.09845	0.38604	-0.15092
TRAM2	-0.37138	-0.2726	-0.15574	-0.01001	0.119978
TRAP1	0.038963	-0.04175	0.064284	0.098632	-0.04777
TRAPPC3	-0.3736	0.013976	-0.14545	-0.17342	-0.06226
TRAPPC6A BLOC1S3	-0.15162	-0.19413	-0.20531	-0.135	-0.15409
TRAPPC6B	-0.77243	-0.65452	-0.76576	-0.78779	-0.93346
TRAPPC9	0.562646	0.206461	0.737755	0.121757	0.543544
TRAT1	0.278286	0.235777	0.140036	-0.18008	-0.94317

TRDMT1	0.018973	0.168663	0.038604	0.033142	0.113556
TRDN	0.392152	-0.61814	-0.84481	-0.2263	-0.46806
TREM1	0.119749	0.033446	0.175365	-0.07342	-0.51348
TREM2	-0.04454	0.119917	0.085731	0.060203	-0.09826
TREML1	0.093153	0.061164	-0.00615	-0.16469	-0.28953
TREML2	0.211826	0.283434	0.499852	0.308982	0.040049
TREML4	0.122121	0.068256	0.321109	0.225633	-0.62959
TRERF1	-1.07993	-0.94441	-0.99907	-0.88464	-1.05852
TREX2	0.214897	0.28603	0.326438	0.243052	0.336508
TREX2 UCHL5IP	0.184102	0.100877	0.358149	0.104284	0.022341
TRFP BYSL	-0.61775	-0.67428	-0.74027	-0.48518	-0.69801
TRH	0.206388	0.438155	0.735206	0.38612	0.477613
TRHDE	-0.20366	-0.21556	-0.13089	-0.12411	0.064725
TRHR	0.296847	0.418332	0.461207	0.058071	-0.26979
TRIAP1 GATC	-0.28275	-0.38522	-0.48789	-0.28477	-0.41463
TRIB1	-0.05122	-0.08945	0.067195	-0.15536	-0.02024
TRIB2	-0.28651	-0.24275	-0.27282	-0.14678	-0.43687
TRIB3	0.126974	0.320454	0.337316	0.193507	0.34684
TRIM11	-0.26324	-0.19064	-0.25489	-0.30372	-0.04156
TRIM14	0.316778	0.193739	0.477572	0.390073	-0.00366
TRIM15	0.483388	0.511289	0.561967	0.295985	0.353321
TRIM16L	-0.34883	-0.22406	0.001683	0.014265	-0.3428
TRIM17	0.032408	0.237356	0.098776	0.041475	0.133766
TRIM2	-0.03652	0.795094	0.849146	0.604637	0.57889
TRIM21	0.159005	-0.88747	-0.70444	-0.30899	-0.38808
TRIM23 AC008560.6	-0.5398	-0.69492	-0.75292	-0.48342	-0.66896
TRIM24	-0.1493	-0.02347	-0.0947	-0.05095	-0.17529
TRIM25	0.17744	0.00873	0.101527	0.280544	0.360662
TRIM26	-0.87404	-0.70891	-0.95568	-0.04597	-0.8328
TRIM27	0.079696	0.210952	0.148699	0.075986	0.162525
TRIM28	-0.04341	-0.15908	-0.02051	-0.04537	0.058276
TRIM29	0.361678	0.571207	0.709067	0.400518	0.423292
TRIM3	-0.22903	-0.23633	-0.37833	-0.08141	-0.0577
TRIM31	-0.80156	-0.99604	-0.79416	-0.83415	-0.94465
TRIM33	0.024121	0.103253	0.132932	-0.05131	0.126289
TRIM35 PTK2B	-0.22767	-0.144	-0.32799	-0.26191	-0.07527
TRIM36	0.118179	0.290743	0.096868	0.202216	0.429552
TRIM37	-0.2624	-0.52773	-0.50664	-0.52277	-0.31671
TRIM39	-0.17161	-0.24587	-0.21165	-0.1274	-0.25148
TRIM4	0.147308	-0.1674	0.428004	-0.39449	-0.27063
TRIM40	0.009687	-1.29074	-1.04536	-1.20066	-0.98624
TRIM41	-0.38279	-0.41478	-0.53029	-0.36948	-0.52187
TRIM42	0.180991	-0.44795	0.019359	-0.10709	-0.41311
TRIM43	0.390442	-0.1258	0.042223	-0.21228	-0.26933
TRIM44	-0.05558	0.359745	0.616965	-0.16187	0.139012
TRIM45	0.037259	0.248486	0.110356	0.195877	0.344666
TRIM47	0.125631	0.175073	0.329905	0.311501	0.320615
TRIM48	-0.45951	-0.60783	-0.42306	-0.39786	-0.63916
TRIM50 FKBP6	0.712856	0.899769	0.833256	0.71017	0.869422
TRIM52	-0.184	-0.09561	-0.05648	-0.20275	-0.14203

TRIM53	-0.22496	-0.8547	-0.56945	-0.38657	-1.10231
TRIM54	0.325592	0.715589	0.597391	0.465847	0.566789
TRIM55	0.596782	0.18646	-0.69829	-0.13982	-0.36485
TRIM56	-0.08565	-0.09867	0.346376	-0.13848	-0.15976
TRIM58	0.309723	0.294216	0.489724	0.341916	0.248646
TRIM59	-0.11464	0.33514	0.5408	0.256347	0.180623
TRIM62	-0.33877	-0.25571	-0.33573	-0.26417	-0.04607
TRIM63	0.490134	0.404133	0.780226	-0.2809	0.503524
TRIM65	0.282552	0.471104	0.546008	0.328871	0.511513
TRIM67	0.186253	0.137464	0.22186	0.119939	0.299162
TRIM68	-0.07635	-0.105	-0.38703	-0.21485	-0.18552
TRIM69	0.207485	0.612206	0.440599	0.220155	0.379061
TRIM71	0.084845	0.395312	0.459308	0.241923	0.607245
TRIM74 AC005488.2	0.501385	0.640071	0.782728	0.586774	0.785621
TRIM8	0.023467	-0.11905	-0.13773	-0.11777	-0.04875
TRIM9	-0.20872	-0.2882	-0.29095	-0.27169	-0.12894
TRIML1	0.275972	-0.0101	0.095223	0.205494	-0.14183
TRIML2	0.470561	0.498668	0.666493	0.495092	0.616744
TRIO	0.016485	0.08396	-0.00705	0.025344	-0.03137
TRIP10	-0.1048	-0.10197	-0.06152	0.368386	0.004107
TRIP11 AL049872.3	-0.46088	-0.34015	-0.49536	-0.31243	-0.43716
TRIP12 FBXO36	-0.58395	-0.46344	-0.34894	-0.40266	-0.52148
TRIP4	0.233277	0.47056	-0.03488	0.322362	0.250755
TRIT1	0.196509	0.422701	0.372036	0.178099	0.283795
TRMT1 BTBD14B	-0.14597	-0.37169	-0.07465	-0.05522	-0.21427
TRMT11	-0.02423	0.403253	0.236053	-0.2438	-0.13485
TRMT12	-0.06935	-0.39586	-0.1066	-0.39011	-0.3772
TRMT5 SLC38A6	-0.71568	-0.70464	-0.97743	-0.66804	-0.90019
TRMT6 MCM8	-0.60949	-0.81731	-0.78808	-0.66865	-0.92668
TRMU	-0.04105	0.182316	-0.05279	-0.03819	-0.22932
TRNT1	0.350107	0.257457	0.334467	0.186779	0.19986
TRO	-0.17798	-0.21135	-0.36291	-0.31465	-0.41293
TROAP	-0.04884	-0.26092	-0.23495	-0.21095	-0.24949
TRPA1	0.308917	0.339236	0.118394	0.245546	-0.24166
TRPC1	-0.20451	-0.13145	-0.13484	-0.14267	-0.04338
TRPC3	0.387409	0.308419	0.391584	0.293044	0.354292
TRPC4	-0.20858	0.131768	0.035907	0.304176	0.440043
TRPC4AP	0.013663	0.065982	0.166058	-0.01044	0.109083
TRPC5	-0.02501	0.479076	0.235874	0.276477	0.360116
TRPC6	0.281368	0.119354	0.226169	0.377813	0.160694
TRPC7	0.496248	0.641046	0.619388	0.445424	0.585613
TRPM1	0.269396	0.260728	0.041186	0.111421	-0.07353
TRPM2	0.498585	0.111286	0.425304	0.395878	0.492859
TRPM5	-0.01428	0.173511	0.216704	0.081589	0.071512
TRPM6	-0.25367	0.328684	-0.23183	0.151582	-0.03763
TRPM7	0.063634	-0.07745	-0.18428	-0.28165	-0.45132
TRPM8	0.14175	0.18377	0.020617	-0.37049	-0.14248
TRPS1	-0.72726	-0.76373	-0.71366	-0.7664	-0.26298
TRPT1 NUDT22	-0.02263	-0.29236	-0.22818	-0.09071	-0.07919
TRPV2	-0.15073	-0.20528	-0.07232	-0.23385	-0.13069

TRPV3	0.481483	0.29667	0.528257	0.43912	0.44734
TRPV4	0.319428	0.426474	0.4437	0.307577	0.506922
TRPV5	0.331599	-0.37236	-0.70435	0.227714	-0.50704
TRPV6	0.192954	0.11202	-0.17358	0.264883	-0.06816
TRRAP	0.108104	0.020812	0.090174	0.126904	0.285891
TRSPAP1	0.005646	0.116388	0.037687	-0.21044	0.24629
TRUB1	-0.43592	-0.3307	-0.27761	-0.28498	-0.44526
TRUB2 COQ4	-0.51758	-0.46737	-0.55129	-0.26566	-0.57323
TSC1	-0.2292	-0.31656	-0.31644	-0.10162	-0.19835
TSC22D2	-0.04312	-0.05411	0.224189	0.074186	0.108574
TSC22D4	0.206049	0.138275	0.152623	0.088232	0.208456
TSEN2	-0.04491	0.162328	0.035315	0.039724	0.246902
TSG101	-0.57958	-0.5302	-0.7311	-0.56118	-0.2837
TSGA10 LIPT1	-0.02326	-0.30685	-0.59833	-0.49546	-0.41498
TSGA10IP	0.017846	0.119195	0.204914	-0.00643	0.179597
TSGA13	0.01589	0.151979	0.10354	-0.30636	-0.62596
TSGA14	-0.06847	0.516156	0.558848	0.151693	0.091499
TSGA2	-0.12856	-0.014	-0.20343	0.164081	-0.04912
TSHB	-0.31919	-0.14125	-0.05464	-0.46283	-0.22763
TSHR	0.324993	0.420039	0.283127	0.256574	0.428891
TSHZ2	-0.57781	-0.56215	-0.65818	-0.56304	-0.60501
TSHZ3	0.529163	0.724149	0.705016	0.548712	0.568981
TSKU	-0.38622	-0.22776	-0.54837	-0.39571	-0.46119
TSLP	0.071388	0.159355	0.139234	-0.10214	-0.43068
TSN	-0.28634	-0.00237	-0.02015	-0.24875	-0.23423
TSNARE1	0.114816	-0.05961	0.155008	-0.06679	-0.05517
TSNAX	-0.01625	-0.06871	-0.05848	-0.10689	-0.02632
TSP50	0.263207	0.316899	0.533794	0.344337	0.422038
TSPAN1	0.282016	0.373331	0.383335	0.099482	0.195361
TSPAN12	-0.83797	-0.48217	-0.93615	-0.6328	-0.8102
TSPAN13	-0.07831	0.080026	0.068214	-0.09834	0.035405
TSPAN14	0.181811	0.382073	0.243462	0.133604	0.273109
TSPAN15	0.237297	0.174193	0.295754	0.220388	0.220561
TSPAN16	0.267956	0.219881	0.306694	-0.01708	0.440374
TSPAN17	-0.08316	-0.20384	-0.14008	0.022722	0.057614
TSPAN18	0.199148	0.528219	0.508686	0.259121	0.309107
TSPAN2	-0.31033	-0.15726	-0.39593	-0.04955	-0.05256
TSPAN3	0.110117	0.121259	0.310631	0.086017	0.359173
TSPAN31	0.151315	-0.46626	-0.58386	-0.24749	-0.65884
TSPAN33	0.048527	-0.02014	-0.19326	0.019038	-0.06285
TSPAN5	-0.31249	-0.04319	-0.28084	-0.22337	-0.33964
TSPAN6	0.085991	0.697688	0.170147	-0.23094	-0.17869
TSPAN7	0.160909	0.044214	0.217322	0.002908	-0.02378
TSPAN9	-0.12116	0.109921	0.056278	0.025412	0.000609
TSPO	0.470596	0.150412	0.255739	0.288122	0.475448
TSPY1	0.399613	-0.01661	-0.06065	0.263378	0.144813
TSPYL1	-0.17831	0.067723	-0.42747	-0.33348	-0.44546
TSPYL2	-0.15294	-0.29336	-0.16827	-0.23845	-0.18346
TSPYL4	-0.47676	-0.26002	-0.56711	-0.43269	-0.66755
TSPYL5	0.462769	0.580582	0.447345	0.523554	0.201278

TSR1 RUTBC1.	-0.01121	-0.07245	0.042358	-0.10745	-0.0233
TSR2	0.250834	0.31742	-0.06449	0.066508	0.016613
TSSC1 TTC15	0.258424	0.362124	0.219097	0.157573	0.281174
TSSC4	0.399568	0.345419	0.529404	0.411376	0.480711
TSSK1	0.277702	0.236409	0.394542	0.137351	0.214838
TSSK3	0.138311	0.107189	0.506966	0.241223	0.174676
TSSK4	0.288703	0.470928	0.455897	-0.05261	0.208171
TSSK6 NDUFA13	0.309698	0.41511	0.424056	0.365991	0.521142
TST MPST	-0.33568	-0.08688	-0.22734	-0.13086	-0.15665
TSTA3	0.126812	-0.25738	-0.14588	-0.14877	-0.05011
TTBK1	-0.08948	-0.24423	-0.20864	-0.30003	-0.52195
TTBK2	-0.27369	-0.12721	-0.17115	0.033072	-0.40777
TTC12	0.067825	0.215109	-0.15949	-0.0822	-0.03163
TTC13 ARV1	-0.12579	-0.27596	-0.5424	-0.29735	-0.26286
TTC14	0.079052	0.31813	-0.09914	-0.01098	-0.05089
TTC17	0.150776	0.187674	-0.17289	0.130827	0.111689
TTC18	0.281205	-0.02884	0.016932	0.000147	-0.4717
TTC21B	-0.19292	-0.09855	-0.25569	-0.21052	-0.03448
TTC22	0.331525	0.49518	0.523114	0.405184	0.429799
TTC23 LRRC28	-0.61841	-0.22334	0.188901	-0.16247	0.470998
TTC25	0.143022	-0.10681	-0.55899	-0.2438	-0.47808
TTC26	-0.01889	0.178906	-0.09625	0.005262	-0.19622
TTC27	0.105016	0.345582	0.233293	0.24721	0.307314
TTC29	0.08493	-0.78842	-0.37279	-0.1547	-0.48798
TTC3	-0.02558	-0.00807	0.058899	-0.20099	0.005348
TTC30B	0.003559	-0.19475	-0.20099	0.147553	-0.04538
TTC32	-0.71812	-0.56598	-0.79931	-0.53908	-0.84326
TTC33	0.338377	0.278375	0.40036	0.163416	0.199893
TTC35	-0.55983	-0.73035	-0.67947	-0.92832	-0.88088
TTC36 TMEM25	0.156798	0.105157	0.080838	0.184806	0.036119
TTC5	-0.44037	-0.36623	-0.62363	-0.48872	-0.61683
TTC6	-0.36372	-0.52476	-0.40146	-0.36668	-0.91443
TTC7A	-0.0332	0.247766	0.379479	0.221992	0.232807
TTC7B	0.007448	-0.04836	-0.14448	-0.02885	-0.14347
TTC8	-0.50672	-0.73942	-0.6317	-0.52786	-0.87741
TTC9B	0.00225	-0.05762	0.023131	0.091452	0.073807
TTF1	-0.00686	-0.1711	-0.37081	-0.30586	-0.44096
TTF2	-0.55383	-0.42208	-0.58964	-0.56455	-0.68127
TTK	-0.26392	0.047349	-0.50147	-0.61655	-0.43285
TTL	0.108404	-0.03404	0.143598	0.02099	0.262339
TTLL1	0.488261	0.461843	0.434246	0.321673	0.243083
TTLL11	0.101359	0.142723	0.167599	0.146001	0.162377
TTLL12	-0.06659	0.063651	0.181833	-0.03233	-0.04224
TTLL13	0.159344	0.089455	-0.09391	-0.07267	-0.0028
TTLL2	0.333058	0.552137	0.532573	0.480021	0.460069
TTLL4	0.203547	0.075279	0.140688	0.097371	0.112336
TTLL6	0.228524	0.282053	-0.81864	-0.14352	-0.1042
TTLL7	-0.37842	-0.16728	0.136529	-0.37961	-0.09509
TTN	0.333756	-0.22526	0.466676	-0.44384	-0.5803
TTPA	0.011452	0.062469	0.258252	0.298216	0.132745

TTR	0.451574	0.555838	0.533374	0.153575	0.197723
TTRAP THEM2	-0.17762	-0.01724	-0.21526	-0.03601	-0.21721
TTTY14.	0.227791	0.310632	0.265938	0.284326	0.291647
TTYH1	0.416149	-0.27836	0.194975	0.265894	0.083541
TTYH3	-0.00972	-0.28917	-0.06645	-0.05628	-0.10975
TUBA1	0.096146	0.239401	0.269205	-0.06516	0.09447
TUBA1A	-0.36047	-0.37363	-1.06206	-0.63221	-1.02808
TUBA1B	-0.12475	-0.67264	-0.78625	-0.34345	-0.8358
TUBA1C	0.067129	0.144834	0.368553	0.276528	0.153004
TUBA3C	0.343277	0.383283	0.50366	0.436538	0.643175
TUBA3D	0.544579	0.630959	0.740828	0.500318	0.558029
TUBA3E	0.373518	0.74459	0.642752	0.339879	0.477496
TUBAL3	0.095261	0.14287	0.027989	0.020077	-0.07525
TUBB	-0.75009	-0.51686	-0.68928	-0.37899	-0.66668
TUBB1	0.54513	0.786893	0.797623	0.44321	0.662815
TUBB2A	-0.21366	-0.04881	-0.27676	-0.23416	-0.17031
TUBB2B	-0.2639	-0.38798	-0.40435	-0.28685	-0.26151
TUBB2C	-0.05813	0.110983	-0.04518	0.027312	0.11711
TUBB3	-0.02496	0.121325	0.097746	0.145088	-0.13117
TUBB4	0.119961	-0.27278	0.175766	0.368252	0.331023
TUBB4Q	0.53229	0.362145	0.441649	0.185773	0.349668
TUBB6	0.07017	0.141892	0.189926	0.100147	0.245172
TUBD1 RPS6KB1	-0.08035	-0.44804	-0.59382	-0.42663	-0.27921
TUBE1 Z99289.1	-0.24395	-0.06621	-0.55526	-0.52962	-0.5684
TUBG2	0.155947	0.03967	0.011167	-0.07693	-0.08005
TUBGCP2 ZNF511	0.082906	0.011509	0.069499	0.057772	-0.00544
TUBGCP3	0.28411	0.314309	0.251931	0.272178	0.085608
TUBGCP5	0.36457	0.018254	-0.0174	-0.01737	0.219483
TUBGCP6 HDAC10	-0.08228	-0.10937	-0.29688	-0.11571	-0.24073
TUFM	0.127494	-0.08792	-0.09265	0.154319	-0.12116
TUFT1	-0.10841	-0.33847	-0.66157	0.048568	-0.37331
TULP1	0.111945	0.374786	0.417057	0.25147	0.285887
TULP2	0.29586	0.493078	0.404985	0.272491	0.265098
TULP3	0.014214	-0.08636	-0.14387	-0.00453	0.1223
TULP4	0.59945	0.720604	0.767792	0.372712	0.57067
TUSC1	0.18847	0.047081	0.10997	0.076844	0.12909
TUSC2	0.272979	0.248925	0.201109	0.172011	0.305041
TUSC3	0.187329	0.185154	0.18877	0.030461	0.147907
TUSC4 CYB561D2	0.075524	0.04727	-0.03623	0.02493	0.011996
TUSC5	0.123059	-0.27552	0.407177	0.158158	0.266859
TUT1	-0.43541	-0.63873	-0.78837	-0.56747	-0.29282
TWF1	-0.35154	-0.30683	-0.34696	-0.36524	-0.41232
TWIST1	-0.70786	-0.66925	-0.59406	0.13309	0.200462
TWISTNB	0.200727	0.301743	-0.09681	0.158583	0.066955
TWSG1	-0.17049	-0.00962	-0.29092	-0.18877	0.029639
TXK	0.461012	0.572537	0.390584	0.150055	-0.04083
TXLNA	0.059619	-0.05984	-0.13989	-0.07612	0.158124
TXLNB	0.128858	0.536478	-0.72872	-0.00233	-0.4399
TXN	-0.19191	-0.17779	-0.05705	-0.05429	-0.20139
TXN2	0.037536	0.345501	-0.02391	-0.03482	-0.19024

TXNDC1	0.117012	0.207076	0.176744	-0.10473	-0.12532
TXNDC10	-0.03306	-0.19075	-0.2014	-0.34669	-0.0532
TXNDC11	0.179468	0.036622	-0.01054	0.13755	0.134308
TXNDC12	-0.11773	-0.09027	-0.16943	-0.17019	-0.05402
TXNDC12 BTF3L4	-0.73311	-0.56704	-0.75798	-0.68629	-0.26807
TXNDC13	-0.26253	-0.16922	-0.17784	-0.10593	-0.33109
TXNDC14 C11orf31	-0.14774	-0.06794	-0.19458	-0.28909	-0.08304
TXNDC16	-0.44445	-0.17843	-0.30467	-0.12735	-0.26821
TXNDC2	0.563368	0.528899	0.599853	0.174127	0.453741
TXNDC3	0.806025	1.03013	1.006239	0.539592	0.657102
TXNDC4 INVS	-0.47946	-0.69146	-0.71053	-0.66406	-0.71227
TXNDC6	-0.30253	-0.48784	-0.4449	-0.37051	-0.52701
TXNDC8	-0.80029	-0.68759	-0.66438	-0.70918	-1.00173
TXNDC9 EIF5B	-0.37342	-0.26274	-0.46689	-0.39241	-0.13962
TXNIP	-0.48193	-0.46937	-1.25859	-0.3919	-0.69628
TXNL1	-0.09809	-0.19272	-0.22015	-0.21707	0.0345
TXNL3	0.035838	-0.0532	-0.06541	-0.08586	0.135357
TXNL4A	0.071705	-0.00111	0.217276	0.133065	0.216679
TXNL4B DHX38	-0.85931	-0.92145	-1.0263	-0.5763	-1.04478
TXNRD2 COMT	-0.19235	-0.16414	-0.2378	-0.25844	-0.30002
TYR	0.338827	0.004795	0.36509	-0.08698	-0.55741
TYRO3	-0.48344	-0.4362	-0.40425	-0.27231	-0.4063
TYROBP	0.09142	0.460688	0.473506	0.40627	0.33664
TYRP1	-0.01482	-0.39516	-0.57144	-0.48128	-0.75163
TYSND1	-0.01731	0.00716	0.033053	-0.0958	-0.1037
U2AF1	0.067514	0.080433	0.339295	0.266227	0.291987
U2AF1L4 PSENEN	-0.34718	-0.28763	-0.62578	-0.27969	-0.3986
U2AF2	-0.30206	-0.30362	-0.23924	-0.22601	-0.39569
U2AF2 EPN1	0.24013	0.020701	-0.02137	0.10576	-0.22691
U47924.1 C12orf57 PTPN6	-0.04717	0.048463	-0.12817	-0.0937	-0.08556
U66059.1	-0.46574	-0.76842	-1.16772	-0.53502	-0.80185
UAP1	-0.104	-0.1179	-0.51349	-0.02893	-0.11596
UAP1L1	0.220431	0.162662	0.610582	0.315242	0.371642
UBA2	-0.10831	-0.11475	0.024205	-0.00588	-0.02459
UBA3	0.126067	-0.06429	-0.11693	-0.09822	-0.25298
UBA52	-0.21976	-0.62745	-0.59109	-0.27118	-0.52293
UBA6	-0.43427	-0.55844	-0.63289	-0.44454	-0.66918
UBA7	0.529398	0.239466	0.721294	0.20916	0.389599
UBAC1	-0.06451	0.028427	-0.10428	0.115029	-0.00095
UBAP1	-0.99349	-0.56582	-1.07461	-0.81041	-1.15548
UBAP2	-0.30105	-0.32008	-0.23914	-0.06922	-0.16049
UBASH3A	0.496264	0.311289	0.872134	0.419084	0.541859
UBASH3B	0.160806	-0.03405	0.033747	0.103658	-0.02191
UBB	-0.07976	-0.06294	-0.11258	-0.17308	-0.30007
UBE2B	-0.30596	-0.74164	-0.80178	-0.61975	-0.65263
UBE2C	0.062555	0.236932	-0.21795	0.033672	0.007128
UBE2CBP DOPEY1	-0.03322	0.101777	0.014321	-0.1037	-0.05247
UBE2D1	0.070777	0.040202	0.131391	-0.07755	0.026115
UBE2D2	0.040686	0.17009	0.023676	0.024319	-0.04217
UBE2D3	-0.95692	-0.83796	-0.84175	-0.77284	-0.91721

UBE2D3 CISD2	0.048883	-0.22842	-0.35261	-0.07014	0.134092
UBE2E1	-0.18601	0.076157	-0.43387	-0.25964	-0.09594
UBE2E2	-0.25661	-0.23757	-0.41238	-0.12617	-0.11745
UBE2E3	0.321092	0.607123	0.641064	0.433112	0.544916
UBE2F	1.799489	0.052439	0.081991	0.02173	0.098359
UBE2G1	0.296315	0.324193	0.364157	0.175555	0.277544
UBE2G2	-0.34934	-0.22619	-0.3578	-0.21693	-0.13961
UBE2H	0.258062	0.186604	0.273438	0.079711	0.111413
UBE2J2	-0.02152	-0.03014	-0.1167	0.077283	0.038589
UBE2K	-0.14172	-0.08127	-0.21599	-0.16242	-0.1611
UBE2L3	0.177585	0.270254	0.200385	0.083169	0.075344
UBE2L6	-0.02631	0.165384	-0.01269	-0.01569	0.074585
UBE2M	-0.20782	-0.09585	-0.07306	-0.18081	-0.04345
UBE2N	-0.44821	-0.05794	-0.25356	-0.37985	0.110451
UBE2NL	0.353703	0.218755	0.267237	0.239316	0.298164
UBE2O AANAT	-0.10612	-0.04168	-0.22908	-0.30374	-0.30765
UBE2Q1	-0.26405	-0.35982	-0.36039	-0.2962	-0.37788
UBE2Q2	0.12586	0.161598	0.095646	0.142415	0.153093
UBE2R2	-0.06365	-0.08986	-0.05013	-0.00975	0.104524
UBE2S	0.077004	0.030787	0.008823	0.111544	-0.06672
UBE2T	0.105639	0.071237	-0.47753	-0.23379	-0.42469
UBE2U	0.59186	0.776601	1.01754	0.72026	0.666907
UBE2V2	0.283001	0.285328	0.336243	0.198681	0.296072
UBE2Z	-0.25337	-0.11471	-0.37923	-0.37115	-0.14613
UBE3C	0.110197	0.041037	-0.11195	-0.04826	-0.05338
UBE4A	0.046381	0.102603	0.003831	0.163519	0.166234
UBE4B	-0.08402	0.064736	0.063431	0.050003	-0.03328
UBL3	-0.19556	-0.31493	-0.1593	-0.17626	-0.14776
UBL4A	0.102028	0.207443	0.296915	0.130375	0.235913
UBL4B	0.727773	0.636941	0.707871	0.605301	0.812077
UBL5	-0.57408	-0.49093	-0.82613	-0.38726	-0.54094
UBL7	-0.50144	-0.25637	-0.66047	-0.29229	-0.32388
UBLCP1	0.130793	-0.7739	-0.94163	-0.78225	-1.17379
UBN1	0.241593	0.070427	0.105311	-0.14652	0.08728
UBOX5	0.070264	-0.07253	-0.46738	0.046675	0.01158
UBP1	-0.31334	-0.07693	-0.23496	-0.18225	-0.08089
UBQLN1	-0.20619	-0.35366	-0.46576	-0.29909	-0.2845
UBQLN2	-0.12049	-0.30094	0.292785	-0.24772	-0.19702
UBQLN4 RP11-336K24.9	-0.36503	-0.4541	-0.60586	-0.50057	-0.34499
UBR1	-0.76894	-0.84274	-1.09994	-0.58036	-0.9501
UBR2	-0.20684	-0.1861	-0.38265	-0.35609	-0.4295
UBR3	0.350899	0.567002	0.618929	0.122619	0.369487
UBR4	-0.65888	-0.90736	-0.76227	-0.51557	-0.60828
UBR5	-0.55419	-0.6233	-0.66191	-0.53833	-0.50759
UBTD2	0.005278	-0.1171	-0.04825	-0.05339	-0.1603
UBTF	-0.03275	-0.24688	0.046158	-0.15184	-0.06943
UBXD1	0.023694	0.049893	-0.1526	-0.0317	-0.18563
UBXD2	0.326755	0.405307	0.554385	0.396384	0.414715
UBXD3	0.221563	0.282706	0.37186	0.10218	-0.07223
UBXD4	-0.04011	-0.06157	0.035539	-0.03673	-0.20004

UBXD6	0.16449	0.151521	0.02555	0.118696	0.065822
UBXD8	-0.42605	-0.16512	-0.54626	-0.39266	-0.36503
UCHL1	-0.61662	-0.00249	-0.46269	-0.45844	-0.5862
UCHL3	-0.27832	-0.20278	-0.45415	-0.54491	-0.68707
UCHL5 TROVE2	-0.3207	-0.39203	-0.59205	-0.31638	-0.29789
UCHL5IP	0.07747	-0.13504	0.141664	0.423921	0.014376
UCK1	0.22468	0.269337	0.322866	0.176545	0.193057
UCK2	-0.25931	-0.06929	-0.30635	-0.37275	0.145255
UCKL1 ZNF512B	-0.03667	-0.13049	-0.08592	0.001464	-0.3051
UCN	0.24123	0.369529	0.061045	0.133779	0.017629
UCN2 COL7A1	0.082676	0.377025	0.471481	0.196886	0.280968
UCN3	0.5518	0.777193	0.512212	0.553826	0.172863
UCP1	0.158394	0.60449	0.382796	0.247757	0.206511
UCP2	-0.24936	0.116839	-0.11671	-0.10828	-0.23665
UCP3	0.115326	0.273499	-0.20822	-0.47195	-0.28793
UEVLD	-0.56173	-0.83118	-0.9051	-0.68692	-0.64703
UFC1	0.190334	0.011979	-0.01868	0.047834	-0.26598
UFC1 USP21	0.149076	-0.34493	-0.43498	-0.01533	-0.29057
UFD1L CDC45L	-0.47618	-0.60388	-0.69016	-0.4481	-0.49782
UFM1	-1.46483	-1.35881	-1.42491	-1.18145	-1.31896
UFSP1 ACHE	0.182213	0.00827	-0.09667	0.158194	0.120101
UFSP2	0.229733	0.249039	0.372507	0.198186	0.141444
UGCG	-0.09801	-0.09809	-0.05987	-0.09374	-0.00705
UGCGL1	-0.24968	-0.3388	-0.40887	-0.25274	-0.11787
UGCGL2	0.273502	0.452295	0.566306	0.209356	0.031069
UGDH	-0.15615	-0.00849	-0.07948	-0.15906	-0.1065
UGP2	-0.4665	-0.18991	-0.2691	-0.19867	-0.05974
UGT2A3	-0.65458	-1.37522	-1.06307	-0.87461	-1.10501
UGT2B10	-0.04342	-1.7323	-1.51671	-1.16275	-1.50672
UGT2B11	-0.76676	-0.97032	-0.82361	-0.87363	-0.78723
UGT2B11 AC021146.7	-0.19038	-2.05129	-1.6754	-1.56176	-1.68369
UGT2B17	-0.46373	-0.64464	-0.37782	-0.56092	-0.65279
UGT2B28	-0.0577	-1.91575	-1.49237	-1.35171	-1.64294
UGT2B4	0.233538	-0.49928	-0.30699	-0.26394	-0.01384
UGT3A1	-0.43183	-0.3169	0.38241	0.173345	0.021067
UGT3A2	-0.21476	-0.41214	0.168464	0.026024	-0.30532
UGT8	-0.01581	0.30574	0.042395	0.167581	0.037369
UHMK1	0.016398	0.229901	0.266197	0.187871	-0.03375
UHRF1	-0.17486	-0.09921	-0.06835	-0.04357	-0.15205
UHRF1BP1	-0.22548	-0.07673	-0.2664	-0.23303	-0.06507
UHRF1BP1L	0.121152	0.194292	0.067744	0.014151	0.110525
UHRF2	0.210393	0.367916	0.528415	0.243886	0.328329
UIMC1	0.118263	-0.06841	-0.07475	-0.02213	-0.1073
ULBP1	-0.65659	0.175656	0.120145	0.032117	-0.07335
ULBP2	-0.50019	-0.37008	-0.46766	-0.23621	-0.20187
ULBP3	-0.33742	-0.09381	0.173411	-0.27744	-0.10083
ULK1	-0.23881	-0.5262	-0.56253	-0.40845	-0.49337
ULK2	0.245966	0.301659	0.388945	0.27894	0.441971
ULK3	0.121962	0.281774	0.29185	0.157075	0.146159
UMOD	-0.35165	-0.81936	-0.86909	-0.62627	-0.73951

UMPS	-0.61037	-0.66672	-1.00468	-0.75303	-0.83836
UNC119	-0.20488	-0.2816	-0.73478	-0.46107	-0.34456
UNC13D	0.194575	0.167556	0.080008	0.22257	0.191485
UNC45B	0.081378	0.002245	-0.27477	-0.52428	-0.17722
UNC5A	0.056745	0.064684	0.115324	0.075272	0.012656
UNC5B	-0.20653	-0.24771	-0.19792	-0.1935	-0.16599
UNC5C	-0.39101	-0.50897	-0.50034	-0.29109	-0.32921
UNC5CL	0.2229	-0.23405	0.166714	0.00814	-0.17843
UNC5D	0.270645	-0.5656	0.166157	-0.18595	-0.61267
UNC84A	0.627986	1.058919	1.036233	0.651796	0.798528
UNC84B	0.080353	0.194197	0.134065	0.144393	0.223789
UNC84B DNAL4	-0.28459	-0.03918	-0.2227	-0.24375	-0.54006
UNC93A	0.091398	0.403817	0.294849	0.008858	0.119638
UNC93B1	0.10606	-0.03158	-0.03221	-0.04714	-0.04436
UNG	-0.08056	0.049063	-0.15599	-0.11781	-0.08548
UNQ9391	0.180216	0.18999	0.476911	0.061927	0.098784
UPB1	0.230498	0.501925	0.284045	0.396591	0.376122
UPF1	0.208109	0.119782	0.074612	0.015961	0.210877
UPF2	-0.10015	-0.1462	0.107078	-0.08367	-0.1608
UPF3A	-0.20709	-0.14665	-0.24602	-0.07371	-0.20309
UPF3B	0.071095	0.103739	-0.38817	-0.10565	-0.0313
UPK1A	0.143882	0.173922	0.291809	0.01536	0.067366
UPK1B	0.417899	0.531421	0.74821	0.363315	0.440213
UPK2	-0.14508	-0.11733	-0.18101	-0.2333	-0.2083
UPK3A	0.330893	0.407023	0.492676	0.44457	0.489324
UPK3B	0.043423	-0.14245	0.106313	0.108534	0.178461
UPP1	-0.1483	-0.14593	-0.14956	0.169119	0.085569
UPRT	-0.17656	-0.46094	-0.75205	-0.19703	-0.77211
UQCR	-0.14294	-0.11193	-0.48247	-0.29425	-0.45429
UQCRB	-0.13395	0.000588	-0.17019	-0.34532	-0.51118
UQCRC1	0.080039	0.147059	0.080891	0.132643	0.080862
UQCRC2	-0.78262	-0.71106	-1.12539	-0.50127	-0.94652
UQCRFS1	0.113121	0.050624	-0.13885	-0.04045	-0.17716
URB1.	-0.08802	-0.15049	-0.42757	-0.14325	-0.19508
URM1	-0.46303	-0.39429	-0.36838	-0.4296	-0.57111
UROC1	0.135632	0.10153	0.201533	0.054348	0.045443
UROS BCCIP	-0.48511	-0.33889	-0.44291	-0.34941	-0.34556
USE1	0.144183	-0.15626	-0.35943	0.084456	0.047965
USF1	-1.56079	-1.29292	-1.72341	-1.22449	-1.56513
USF2	0.056598	-0.10737	0.168654	0.029886	0.061077
USH1C	0.06478	0.375062	0.540312	0.166842	0.299192
USH1G OTOP2	-0.01556	0.036467	0.163339	0.28256	0.095173
USH2A	-0.09613	0.317936	0.109162	-0.19811	0.096148
USHBP1	0.225282	0.594456	0.674458	0.412329	0.391759
USMG5P1. PDCD11	-0.25691	-0.49467	-0.94395	-0.45004	-0.58269
USO1	-0.77347	-0.67915	-0.73105	-0.4926	-0.74806
USP1	-0.14484	0.000962	0.062212	-0.1246	-0.0326
USP10	0.2329	0.227795	0.328287	0.249654	0.261712
USP11	0.377847	0.014356	-0.18584	-0.22046	-0.14657
USP12L	-0.19231	-0.07733	-0.40481	-0.18328	-0.2988

USP13	-0.12889	-0.04129	-0.3564	-0.14833	-0.26239
USP14	-0.35472	-0.12892	-0.23905	-0.28642	-0.09233
USP15	-0.48579	-0.91138	-0.89575	-0.85174	-0.80791
USP16	-0.19171	0.253145	0.243333	-0.04316	0.151314
USP18	0.305787	0.538246	0.641219	0.344157	0.239418
USP25	0.036305	-0.03605	-0.02463	-0.11285	-0.05121
USP26	-0.00578	0.290926	-0.08437	-0.13059	-0.0818
USP28	0.149004	0.391656	0.370482	0.22256	0.208229
USP29	0.118986	0.496558	0.570536	0.396888	0.487066
USP3	-0.07401	-0.1311	-0.11258	0.034509	0.098982
USP30	0.326698	0.031007	0.079781	0.031811	0.067085
USP31	0.015169	0.166887	0.312948	0.106714	0.1434
USP32	-0.45258	-0.69052	-0.92168	-0.69427	-0.6827
USP33	-0.15177	-0.14063	-0.11346	-0.30519	-0.0713
USP36	-0.04856	-0.11767	0.016701	-0.01243	-0.0239
USP37 RQCD1	-0.75583	-0.63967	-0.85016	-0.6007	-0.7963
USP38	-0.30193	-0.61065	-0.50991	-0.44375	-0.38202
USP39	0.088254	0.35674	0.110194	0.201597	0.184728
USP4	-0.02059	0.081957	-0.3151	-0.16579	0.070558
USP40	0.280827	0.627312	0.437692	-0.06253	0.171346
USP41	0.138804	0.281649	0.205367	0.307925	0.287973
USP42	0.004792	-0.03566	0.088159	-0.03057	0.003664
USP44	-0.18406	0.675228	0.826355	0.348866	-0.04607
USP47	0.031608	0.113291	-0.11689	-0.2453	0.263285
USP48	-0.15474	-0.05209	-0.15206	-0.0008	0.160272
USP49	-0.42208	-0.28252	-0.53081	-0.35962	-0.29478
USP50	0.720676	0.756591	1.072657	0.390068	0.155606
USP51	-0.19855	-0.65909	-0.45429	0.238398	-0.80369
USP53	0.099874	0.033671	-0.06268	-0.23551	0.022161
USP54	-0.27114	-0.42056	-0.63829	-0.30901	-0.35855
USP6	0.476751	0.101022	0.131604	-0.17518	0.085767
USP7	0.037463	-0.02902	0.005155	0.02007	0.028426
USP8	-0.3054	-0.15715	-0.1836	-0.21987	-0.15339
USP9X	-0.23801	-0.11135	-0.27432	-0.10293	-0.16309
USP9Y	0.223476	0.234493	0.259443	0.059116	0.101859
UST	-0.32479	-0.16698	0.110523	-0.32674	-0.23322
UTF1	0.014976	0.269878	0.348202	0.278251	0.401857
UTP11L	-0.32663	-0.20049	-0.18738	-0.60891	-0.15704
UTP14A	0.330359	0.136788	0.215006	0.091766	0.174703
UTP14C	0.6364	0.825999	0.841034	0.302303	0.397977
UTP20	-0.60333	-0.43255	-0.285	-0.22772	-0.50546
UTP3	-0.6556	-0.34482	-0.57792	-0.49227	-0.38202
UTP6	0.159688	-0.00268	-0.29933	-0.08516	-0.03175
UTRN	0.615892	-0.08719	-0.49059	-0.05428	-0.16886
UTS2	0.461857	0.563649	0.726758	0.340419	0.546982
UTS2R	0.213759	0.063109	0.341121	0.205268	0.176003
UTY	0.275361	-0.08304	0.177515	0.446069	-0.05858
UVRAG	-0.43726	-0.25432	-0.41752	-0.35488	-0.49952
UXS1	-0.05022	0.104607	-0.03359	-0.18526	-0.01594
UXT	0.343116	-0.25161	0.050893	-0.6686	-0.17234

VAC14	-0.17296	-0.13871	-0.11274	-0.05695	-0.44678
VAMP1	-0.26498	-0.1671	-0.40833	-0.44724	-0.28577
VAMP2	0.024598	2.88E-05	-0.50783	0.081074	-0.06144
VAMP4	-0.56388	-0.65785	-0.68368	-0.6392	-0.50432
VAMP5	0.160956	0.242087	0.273474	0.186627	0.464343
VAMP7	0.467552	-0.10831	-0.29159	-0.2413	-0.59292
VAMP8	0.264927	0.650424	0.573155	0.316162	0.593973
VANGL2	-0.18338	-0.11351	-0.43031	-0.4393	-0.10173
VAPA	-0.00927	0.026906	0.150113	0.046123	0.08919
VAPB	0.107779	0.122878	-0.07209	-0.03772	0.002834
VARS	-0.00492	-0.02131	-0.09142	-0.01963	0.047699
VASH1	0.101913	0.003484	0.124503	0.148918	0.096764
VASH2	-0.46018	-0.28832	-0.523	-0.42387	-0.27607
VASP	-0.39397	-0.3158	-0.29566	-0.2781	-0.325
VAT1	0.079786	-0.06363	0.01054	-0.01769	0.098665
VAV1	0.386323	-0.3021	-0.07317	0.242606	0.376463
VAV2	-0.07994	0.135093	0.079046	-0.02246	0.027737
VAV3	-0.04278	0.04844	-0.02212	-0.06939	0.115232
VAX1	-0.35865	0.133789	0.119676	0.317328	0.23203
VAX2	-0.38973	-0.37214	-0.23991	-0.19422	-0.1186
VBP1	0.297847	0.222324	0.087787	1.440334	0.091288
VCAM1	-0.5039	0.013911	0.459343	0.022946	-0.16047
VCL	-0.04413	0.036892	-0.01965	-0.10571	-0.02426
VCPIP1	-0.77132	-0.51679	-0.76536	-0.42518	-0.77119
VCX	0.249076	-0.17929	-0.29241	-0.12598	-0.36325
VCX2	0.372581	0.629911	0.506717	0.438247	0.454472
VCX3A	0.718098	0.569195	0.323738	0.508915	0.523411
VCX3B	0.394394	-0.06599	0.108833	0.287028	0.188465
VCY	0.418925	0.512717	0.664361	0.56763	0.51291
VCY1B	0.419665	0.666336	0.355346	0.400271	0.559639
VDAC1	0.078365	0.139779	0.102495	0.068555	0.107549
VDAC2	-0.22928	-0.20096	-0.30782	-0.13274	-0.04614
VDAC3	0.236698	0.235025	0.173626	0.113312	-0.01024
VDR	0.002125	-0.46303	-0.32004	0.133997	0.031401
VEGF	-0.34849	-0.38522	-0.53279	-0.34255	-0.35983
VEGFC	-0.17064	0.038802	-0.00261	-0.01577	-0.01242
VENTX	-0.02664	0.046553	0.138094	0.207002	0.260188
VEZF1	-0.18767	-0.46994	-0.27744	-0.22355	-0.17253
VEZT	0.421126	0.317794	0.329719	-0.03589	-0.15245
VGCNL1	0.193653	0.074735	0.115702	0.114402	0.169517
VGF	-0.35927	-0.48561	-0.60824	-0.32275	-0.26579
VGLL1	0.039377	0.150058	-0.09392	0.054802	0.181858
VGLL2	-0.60975	0.097868	0.180205	0.242261	-0.0759
VGLL3	-0.03431	-0.14209	-0.17007	0.003191	-0.07312
VGLL4	-0.10859	0.17352	0.025067	-0.05894	0.095657
VHLL	0.537365	1.001474	0.862666	0.666848	0.691401
VIL1	0.278882	0.519533	0.332961	0.35926	0.363196
VIL2	-0.42631	-0.32104	-0.47283	-0.33597	-0.41558
VILL	0.350712	0.638078	0.85152	-0.01164	0.212496
VIM	-0.3792	-0.48823	-0.55095	-0.21445	-0.18121

VIP	-0.26678	-0.20238	-0.36161	-0.20398	-0.54045
VIPR1	-0.01815	0.189298	-0.00551	0.052741	0.070248
VIPR2	0.103235	-0.31658	-0.10825	0.065349	-0.06221
VIT	-0.29739	-0.19573	-0.36002	-0.72277	-0.81613
VKORC1	0.296844	0.234903	0.225449	0.228086	0.220451
VKORC1L1	0.0317	-0.12641	-0.14556	-0.02945	-0.01354
VLDLR	-0.02931	0.359864	-0.01227	-0.01698	0.047727
VMO1	-0.14555	0.371089	0.398385	0.259651	0.270854
VN1R1	0.439915	0.502712	0.40534	0.147938	0.105373
VN1R2	0.646494	0.700632	0.621282	0.508757	0.508055
VN1R4	-0.02764	-0.83489	-0.71086	-0.66484	-0.82573
VN1R5	-0.00465	-0.02063	-0.21204	-0.32329	-0.29228
VNN1	0.3472	-0.51332	-0.84038	0.013634	-0.71545
VNN3	-0.42818	-0.60694	-0.95245	-0.74528	-1.0672
VPREB1	0.239331	0.225765	0.384115	0.022347	0.094752
VPREB3	0.306537	0.445767	0.475212	0.321462	0.472445
VPS11	-0.07915	-0.01439	-0.36677	-0.15321	-0.46561
VPS13A	-0.05175	0.086768	-0.0086	-0.10569	-0.0396
VPS13B	-0.56894	-0.39382	-0.74826	-0.53356	-0.68909
VPS13C	-0.09359	0.05891	0.067785	0.093043	0.030398
VPS13D	0.093874	0.175247	0.305801	0.127854	0.201261
VPS16	0.367803	0.671397	0.602885	0.420308	0.429516
VPS18	0.123622	-0.0417	-0.12823	-0.01405	0.02731
VPS24	0.257657	0.385537	0.234233	0.00779	0.261602
VPS25	-0.1891	-0.17885	-0.42498	-0.33529	-0.3469
VPS26A	0.010607	-0.15462	-0.14305	-0.12784	0.019087
VPS28 NFKBIL2	-0.2889	-0.29659	-0.32695	-0.25785	0.049027
VPS29 RAD9B	0.227834	0.413616	0.21074	-0.2804	0.335978
VPS33A	-0.15668	-0.36973	-0.35163	-0.30321	-0.30971
VPS33B	-0.12119	0.038152	-0.07074	-0.23743	-0.20236
VPS35 ORC6L	-0.49555	-0.40907	-0.59472	-0.30951	-0.55435
VPS36	0.036367	0.117359	0.093502	-0.04796	0.09245
VPS37B	0.39836	0.259346	0.276958	0.358391	0.221735
VPS37C	-0.07428	-0.07944	-0.15004	-0.3061	-0.02624
VPS39	0.294455	-0.09193	0.024933	0.207266	-0.29023
VPS41	-1.18767	-1.11775	-1.50563	-0.91831	-1.19208
VPS45	-0.75324	-0.98336	-1.19218	-0.87549	-0.96991
VPS4A	0.100159	0.144465	0.073699	0.082149	-0.08622
VPS4B	-0.62451	-0.61474	-0.748	-0.5425	-0.41716
VPS52 RPS18	-0.46724	-0.62676	-0.91985	-0.53704	-0.78478
VPS53	-0.11581	0.116729	-0.06457	0.065229	0.144605
VPS54	0.003473	-0.01399	0.045952	-0.03335	0.074283
VPS72	-0.00256	0.097169	-0.98567	0.052932	0.199972
VPS8	-0.10778	-0.19283	-0.19114	-0.20751	-0.2659
VRK1	0.136982	0.340967	0.409937	0.150768	0.097527
VRK2	0.571847	0.440577	0.93769	0.38449	0.575421
VRK3 ZNF473	-0.11439	-0.10272	-0.31855	-0.1419	-0.14619
VSIG1	0.661917	0.658238	0.469706	-0.18048	-0.01243
VSIG2	-0.02558	0.295383	0.378921	0.098205	0.218019
VSIG4	-0.8832	-0.75445	-0.99539	-0.41858	-0.59889

VSNL1	-0.75554	-0.76841	-0.83276	-0.53999	0.094235
VSTM1	0.886129	-0.1216	0.756729	0.645984	0.841964
VSTM2A	-0.48337	-0.60059	-0.67684	-0.56006	-0.63133
VSTM3	0.319053	0.414065	0.466523	-0.0304	0.102916
VSX1	0.127197	0.2604	0.66361	0.480318	0.391893
VSX2	-0.21879	-0.45381	-0.04318	0.028508	-0.11051
VTA1	-0.1729	-0.00627	-0.25145	-0.32558	-0.40071
VTCN1	0.191343	0.136918	0.353803	0.228484	-0.14077
VTI1B	0.18852	0.457491	0.213187	0.170545	0.147015
VTN SARM1	-0.19088	-0.24347	-0.44638	-0.11884	-0.28828
VWA1	0.045117	0.063901	0.17984	0.090288	0.122708
VWA2	0.029002	0.132902	0.597359	0.172839	0.179224
VWA3B	-0.15947	-0.01642	-0.00992	0.207318	0.075011
VWC2	0.020711	-0.05792	0.107586	0.329026	0.226843
VWCE	0.032269	0.171851	0.042675	-0.08779	0.037241
VWF	-0.1886	-0.28878	-0.20412	-0.29938	-0.30512
WAC	-0.17532	-0.1128	-0.23581	-0.07356	-0.14402
WAPAL	-0.27482	-0.11442	-0.15571	-0.21	-0.33812
WARS WDR25	-0.35695	-0.30412	-0.71503	-0.43685	-0.60506
WARS2	-0.17832	-0.22796	-0.35894	-0.32788	-0.47448
WAS	0.367126	0.459682	0.049799	0.195595	0.703238
WASF1 CDC40	-0.48362	-0.12555	-0.40137	-0.23295	-0.31464
WASF2	0.347217	0.147782	0.222652	0.060304	0.267331
WASF3	-0.14988	-0.00143	0.100264	-0.07471	0.037149
WASL	-0.08086	-0.17552	-0.30764	-0.05842	-0.15054
WASPIP	0.117139	0.308604	0.323064	0.168261	0.503539
WBP11 C12orf60	-1.10892	-1.58488	-2.02822	-1.60906	-1.889
WBP2	-0.24625	-0.45645	-0.60592	-0.58415	-0.52273
WBP4	-0.45351	-0.45307	-0.32109	-0.18495	-0.25858
WBP5	-0.20822	-0.5687	-1.2348	-0.64287	-0.79254
WBSCR1	-0.01529	-0.10688	-0.01972	0.106348	0.091141
WBSCR16	0.238259	-0.0293	-0.24143	-0.14157	-0.27612
WBSCR17	0.098934	0.36701	0.55476	0.102318	0.354452
WBSCR18 WBSCR22	-0.37336	-0.1732	-0.43904	-0.23753	-0.40607
WBSCR27	0.09448	0.103746	0.089568	-0.00874	0.158584
WBSCR28	0.137212	0.275219	0.548329	0.096086	0.002475
WDFY1	-0.22475	-0.33226	-0.11306	-0.16989	-0.23577
WDFY2	-0.19754	-0.17333	-0.07951	-0.22163	-0.189
WDFY3 C4orf12. AC108021.3	-0.52848	-0.51809	-0.59492	-0.51572	-0.50332
WDHD1 SOCS4	-0.78371	-0.59427	-0.67295	-0.71118	-0.66804
WDR1	-0.2752	0.214136	0.120993	0.123115	0.091139
WDR11	0.27046	0.269937	0.367825	0.25674	0.117764
WDR12 ALS2CR8	-0.47566	-0.37609	-0.8039	-0.37464	-0.42612
WDR13	0.204524	0.270928	0.402975	0.090246	0.078731
WDR17	0.129094	0.233818	0.101607	-0.09778	-0.07316
WDR18	0.241183	0.159642	0.115608	0.161744	0.162371
WDR19	0.106533	0.175984	0.162426	-0.12483	-0.13398
WDR21A	0.195981	0.503795	0.190885	0.284406	0.052527
WDR21B	0.654943	0.99829	0.919316	0.723695	0.747622
WDR21C	0.577107	0.978057	0.857201	0.846155	0.834665

WDR22	-0.14252	-0.22654	-0.19759	-0.10196	-0.01809
WDR24	0.473422	0.510298	0.363292	0.401349	0.466826
WDR26	-0.30139	-0.18476	-0.24635	-0.22176	-0.22386
WDR27 C6orf120	-0.09114	-0.05349	-0.0459	-0.13415	-0.15494
WDR31	0.248565	0.443582	0.258645	0.256461	0.14183
WDR33	0.20464	0.550669	0.29941	0.321113	-0.04615
WDR34	0.321913	0.366242	0.370689	0.155937	0.4251
WDR35	0.184999	0.358481	0.311483	0.227556	0.390303
WDR36	-0.20179	-0.03068	-0.23849	-0.44444	-0.43208
WDR37	-0.54939	-0.41019	-0.61102	-0.35196	-0.41442
WDR4	-0.02462	0.224228	-0.09479	-0.07589	0.041261
WDR40A	0.005344	0.216237	0.120675	0.086617	0.173982
WDR40B	0.402963	0.461977	0.462019	0.446828	0.449107
WDR40C	0.35378	0.437249	0.237663	0.304095	0.508554
WDR41	-0.15246	-0.00373	-0.04901	-0.23213	-0.23065
WDR42A	-0.3997	-0.37696	-0.49085	-0.51392	-0.42143
WDR42B	0.432521	0.861878	0.134075	0.401267	0.721791
WDR44	0.284601	-0.38806	0.383217	-0.22584	-0.42942
WDR45L	-0.07402	-0.56218	-0.3967	-0.27275	-0.32379
WDR46 PFDN6	-1.09434	-1.33943	-1.8019	-0.85327	-1.71885
WDR47	0.210018	0.190692	0.371508	0.090674	-0.01033
WDR48	-0.14768	0.116536	-0.10957	0.067889	-0.09236
WDR49	-0.87631	-0.89418	-0.83953	-0.72377	-1.00496
WDR51A	-0.2717	-0.03948	-0.33738	-0.20277	0.085037
WDR51B	-0.50824	-0.10419	-0.38713	-0.13847	-0.18607
WDR52	0.618064	0.842771	0.943601	0.624612	0.687536
WDR53 FBXO45	-0.38965	-0.43874	-0.3925	-0.35791	-0.27119
WDR54	0.161785	0.155497	-0.11398	0.025028	0.171263
WDR55	0.081096	-0.0859	-0.10636	-0.01895	-0.08173
WDR57 ZCCHC17	-0.96082	-0.87641	-0.9362	-0.8044	-0.45311
WDR59	0.217537	0.427653	0.357749	0.1623	0.232693
WDR60	0.193849	0.10723	0.16455	0.215145	0.254414
WDR61	-0.07069	-0.08544	-0.33252	-0.16667	-0.23228
WDR64	-0.06016	-0.26928	-0.29457	-0.62309	-0.88017
WDR66	0.370969	0.569988	0.63605	0.430794	0.05295
WDR67	0.224213	0.312433	0.506518	0.231218	0.112003
WDR69	0.129855	0.754056	0.397632	0.209311	0.390979
WDR7	-0.08481	-0.30791	-0.50912	-0.40253	-0.09278
WDR70	-0.03382	-0.05976	-0.35694	-0.10117	-0.33432
WDR72	0.279747	0.129999	0.348609	0.278732	0.254263
WDR73	-0.44305	-0.45558	-0.42261	-0.35871	-0.4119
WDR74	-0.21001	-0.31736	-0.476	-0.16603	0.169437
WDR75	-1.19122	-1.05841	-1.32818	-1.02708	-1.11879
WDR77 ATP5F1	-0.91395	-0.97397	-0.94562	-0.96222	-0.92172
WDR78 MIER1	-0.20228	-0.44003	-0.45636	-0.34191	-0.31813
WDR8 TP73	0.069948	0.265104	0.42924	0.292042	0.349107
WDR82	0.492024	0.439952	0.733083	0.16834	0.200892
WDR85	0.31078	0.523038	0.386335	0.267952	0.352731
WDR86	0.133132	0.33816	0.186204	0.216355	0.38842
WDR87 SIPA1L3	0.067567	0.063903	0.140444	0.04728	0.149888

WDR88	0.472146	0.432158	0.643792	0.239663	0.465419
WDR89	-0.61327	-0.59001	-1.12704	-0.64965	-0.78712
WDR90	0.224348	0.059112	0.070724	0.051934	0.155207
WDR90 RHOT2	0.311696	0.166461	0.284404	0.174665	0.122481
WDR91	0.246945	0.248208	-0.00458	0.208131	0.357597
WDSUB1	0.025147	-0.0762	-0.21329	-0.00741	0.183547
WDTC1	-0.10473	-0.08182	-0.02967	0.013525	-0.03499
WEE1	0.306687	0.179565	0.020156	0.062035	0.095274
WFDC1	0.333037	0.301016	0.281382	0.013135	0.078357
WFDC10B	-0.14222	-1.03289	-0.74051	-0.7168	-1.05678
WFDC10B WFDC13	-0.85291	-0.84189	-0.75622	-0.58302	-0.65937
WFDC11	-0.69075	-1.15365	-1.43539	-1.33191	-1.62479
WFDC12	0.384678	-0.86534	-0.67947	-0.33861	-0.00512
WFDC3 DNTTIP1	0.252861	0.052091	-0.06486	0.170069	-0.10218
WFDC5	0.098755	-0.88041	-0.06496	-0.45911	-0.00129
WFDC8	0.4111	-0.65075	-0.77215	-0.04145	-0.01311
WFDC9	-0.52602	-1.0612	-0.99414	-0.97368	-0.81535
WFIKKN1	0.159278	-0.02933	0.200921	0.076037	0.167393
WFIKKN2	0.488225	0.712771	1.008622	-0.48287	0.755006
WFS1	0.134255	0.233006	0.225833	0.055051	0.049039
WHSC1L1	-0.24126	-0.16355	-0.31314	-0.12192	-0.23459
WHSC2	-0.46417	-0.3933	-0.52209	-0.37619	-0.40532
WIF1	0.275484	0.257443	0.590811	0.337863	0.186806
WIPF2	-0.4686	-0.15834	-0.67662	-0.50718	-0.74351
WIPI1	-0.21881	-0.36738	-0.418	-0.33401	-0.24984
WISP1	0.13809	0.188284	0.414533	0.147574	0.115368
WISP2	0.262462	0.375506	0.343842	0.301574	0.210918
WNK2	0.18716	0.33241	0.354738	0.225655	0.271327
WNK3	0.020704	-0.2289	0.050577	-0.45222	-0.11929
WNK4	-0.02682	0.160585	0.184762	0.396459	0.212062
WNT1	0.203929	0.18133	0.321838	0.276812	0.2616
WNT10A	-0.07855	0.290101	0.201762	0.010877	0.367914
WNT10B	0.237116	-0.22217	-0.26342	-0.09575	-0.2075
WNT11	-0.27442	-0.48726	-0.21482	-0.05934	-0.13599
WNT2	-0.45048	-0.01139	0.295774	0.236022	0.11742
WNT3	0.065028	0.475021	0.579462	0.458443	0.428371
WNT3A	0.208067	0.048355	0.340536	0.435893	0.189743
WNT4	-0.06068	-0.03042	-0.03742	0.001513	-0.07299
WNT5A	-0.81258	-0.38514	-0.80822	-0.40028	0.091219
WNT6	-0.49043	0.12404	0.25206	0.079859	0.229904
WNT7A	0.094276	0.075356	0.098682	0.183722	0.271507
WNT7B	-0.05764	0.122341	0.070343	0.110776	-0.0194
WNT8A	0.042744	0.342764	0.255573	-0.07696	0.038382
WNT8B	-0.32901	-0.85553	-0.57136	-0.42353	-1.26049
WNT9A	-0.05214	-0.16584	-0.19918	-0.11329	0.04447
WNT9B	0.030409	0.122078	0.216695	0.059358	0.089009
WRB	0.259367	0.151553	0.116529	0.021413	0.133132
WRNIP1	-0.07333	-0.18163	-0.17177	-0.11569	-0.10717
WSB1	0.065154	0.366625	0.169454	0.084147	0.262333
WSB2	-1.41858	-1.53222	-1.55309	-1.04619	-0.9071

WSCD1	-0.00363	1.317302	0.068415	0.158867	0.283206
WSCD2	0.115262	0.315174	0.357681	0.318881	0.303142
WT1 WIT1	-0.71313	-0.08933	0.426564	0.38443	0.23319
WTAP	-0.29066	-0.06353	-0.17596	-0.25757	-0.13806
WWC1	-0.02895	0.017948	0.086702	0.034998	0.047401
WWC3	-0.09115	0.05417	0.018245	0.010738	0.086037
WWOX	0.026448	0.130997	0.283453	0.148657	0.171377
WWTR1	-0.14637	0.257834	0.670799	0.422467	0.381333
XAB2	-1.04925	-1.36704	-1.82046	-1.07422	-1.8691
XAF1	0.218533	-0.0207	-0.39612	-0.51784	-0.06534
XAF1 FBXO39	0.014844	-0.25622	0.221594	0.288494	0.310895
XAGE1D	0.54481	0.513255	0.534652	0.438045	0.59849
XAGE2B	0.593303	0.293097	-0.51212	0.280392	0.264513
XAGE3	0.609896	0.54284	0.068525	0.458266	0.564983
XBP1	0.408423	0.189009	0.131418	0.20599	0.141042
XCL1	0.081277	-0.05465	-0.19148	-0.38788	-0.04933
XCL2	0.188292	0.29746	0.068168	-0.45814	-0.15675
XCR1	-0.06986	-0.10958	-0.63435	-0.65739	-0.68937
XDH	0.12689	-0.69794	-0.67992	-0.6155	-0.81542
XG	0.389291	-0.06946	0.042118	-0.20264	0.09598
XIAP	0.16468	-0.31003	0.223787	-0.21301	-0.27745
XIRP2	-0.03713	-0.90224	-0.11339	-0.48115	-0.57512
XK	0.246747	0.19236	0.580535	0.333191	0.294189
XKR3	-0.38095	-0.44271	-0.36174	-0.3776	-0.41824
XKR4	0.913705	-0.13423	0.086759	0.103532	-0.01217
XKR5.	0.189636	0.316394	0.099109	0.189308	0.31656
XKR7	-0.48234	-0.47907	-0.55496	-0.37347	-0.49761
XKR8	0.298175	0.306757	0.579307	0.273084	0.265388
XKRX	-0.05107	0.326505	0.402351	0.237185	0.479016
XKRY	0.208816	0.331005	0.233893	-0.03257	0.143609
XPA	-0.22452	-0.10401	-0.29125	-0.299	-0.27694
XPC LSM3	-0.90987	-0.28082	-0.83885	-0.74856	-0.37287
XPNPEP1	0.208143	0.42758	0.38638	0.315981	-0.0033
XPNPEP2	-0.43235	-0.5035	-1.08536	-0.35313	-0.80024
XPNPEP3	0.276786	0.371138	-0.00598	-0.32713	-0.19295
XPO1	-0.33206	-0.23852	-0.58103	-0.36859	-0.16512
XPO4	-0.37246	-0.21975	-0.38142	-0.50986	-0.58684
XPO5	-0.11804	-0.4159	-0.52332	-0.2976	-0.34097
XPO7	-0.46658	-0.61903	-0.64138	-0.45565	-0.53856
XPOT	0.012571	0.232315	0.301403	0.169792	0.108649
XPR1	-0.36897	-0.4686	-0.56495	-0.53905	-0.35443
XRCC1	0.182008	0.065384	-0.02929	0.069479	0.043637
XRCC2	-0.03107	-0.0958	-0.15483	-0.07608	-0.20895
XRCC3 ZFYVE21	0.077166	0.059171	0.047607	0.148153	0.137329
XRCC5	-0.60548	-0.36337	-0.34579	-0.32874	-0.42162
XRCC6BP1	-0.52601	-0.44494	-0.56579	-0.41759	-0.55313
XRN1	0.159725	0.056325	-0.39584	-0.12951	-0.08754
XRN2	-0.08715	-0.21139	-0.29562	-0.10165	-0.20113
XRRA1 SPCS2	-0.3516	-0.01393	-0.46473	-0.33785	-0.43855
XX-FW88277B6.1	0.133852	0.630366	0.232586	0.254317	0.050551

XYLB	0.151989	0.067563	0.185441	0.024222	0.156703
XYLT1	-0.29174	-0.38613	-0.14064	-0.11129	0.091946
XYLT2	0.001943	0.258483	0.264105	0.114108	0.271005
YAF2	0.03224	0.141472	0.155017	0.089513	0.091893
YAP1	-0.06973	-0.00905	0.049736	0.010878	-0.05972
YARS S100PBP	-0.49741	-0.39005	-0.46078	-0.32923	-0.08264
YARS2	-0.37448	-0.5921	-0.49008	-0.46909	-0.6167
YBX1	-0.40996	-0.29345	-0.36443	-0.26998	-0.1143
YBX2	0.088535	0.149008	0.18036	0.173663	0.23798
YDJC	0.329683	0.349943	0.39465	0.261488	0.313204
YEATS2	-0.06316	0.148018	0.135182	-0.08269	0.135321
YEATS4	0.292078	0.136048	0.453719	0.280033	0.105055
YES1	-0.19141	0.08427	0.057128	-0.13646	0.046984
YIF1A	-0.07927	-0.27174	-0.18184	-0.05388	-0.17452
YIPF1	-0.00891	-0.02857	-0.14826	-0.32781	-0.06861
YIPF2 C19orf52	0.023956	-0.13057	-0.15274	-0.05462	-0.15556
YIPF3 POLR1C	-0.89566	-0.84964	-1.22184	-0.82101	-1.05966
YIPF4	0.237993	0.012213	0.14691	0.144746	0.1343
YIPF5	-0.43831	-0.85453	-0.63319	-0.62862	-0.96082
YIPF6	0.047991	0.085391	-0.08935	-0.02589	-0.12857
YKT6	-0.39244	-0.31185	-0.43796	-0.34247	-0.32001
YME1L1 MASTL	-0.44485	-0.44908	-0.39404	-0.39068	-0.41062
YOD1 PFKFB2	0.104155	0.257508	-0.15814	0.146352	0.015831
YPEL1	-0.14708	-0.11742	-0.13225	-0.12566	-0.06631
YPEL2	0.045249	0.086281	0.15121	0.073035	0.091119
YPEL4	-0.8405	-0.53992	-0.59867	-0.73258	-0.66556
YPEL5	-0.30513	-0.22391	-0.28941	-0.14333	-0.19777
YSK4	0.179946	0.342918	0.482746	0.06866	0.206772
YTHDC1	0.201162	0.290859	0.235593	0.036291	0.131772
YTHDC2	-0.0402	-0.06592	-0.06353	-0.08906	-0.07737
YTHDF1	-0.00063	0.008715	0.027502	0.10687	0.196615
YTHDF2	-0.19749	0.077316	-0.01651	0.014436	0.127005
YTHDF3	-0.26441	-0.24375	-0.22983	-0.19209	-0.2066
YWHAB	-0.11849	-0.02294	-0.30506	-0.20353	-0.17619
YWHAE	-0.06617	0.020056	0.135166	-0.11371	0.152569
YWHAG	-0.06311	-0.15168	-0.25786	-0.15391	-0.27448
YWHAQ	-0.13431	-0.14754	-0.18118	-0.19005	-0.11016
YWHAZ	-0.3816	-0.41073	-0.42635	-0.63653	-0.40984
YY1	-0.31652	-0.33446	-0.32292	-0.35063	-0.23851
YY1AP1 DAP3	-0.43434	-0.71177	-0.82763	-0.57806	-0.63715
Z70227.1 AL034485.16	0.296479	0.216136	0.018303	0.089707	-0.21737
Z82185.1	0.492624	0.54974	0.649795	0.110167	0.255951
Z83854.2	0.347485	0.317149	0.214909	0.318142	0.461628
Z85996.1	0.110041	0.091474	0.066774	0.121535	-0.18887
Z92544.1 WDR24	0.091233	1.103959	0.279902	0.279117	-0.01492
Z93017.6	0.207088	0.574105	0.585991	0.384114	0.605001
Z97195.1	-0.6934	-0.56413	-0.87782	-0.54251	-0.44642
Z97195.1 C1orf105	0.086885	0.541765	-0.10324	0.021419	0.105686
ZACN	0.312257	-0.14371	0.038066	0.301508	0.025992
ZADH1	0.050116	0.146553	-0.89178	-0.48769	-0.40226

ZADH2	-0.2957	-0.30738	-0.2664	-0.12056	-0.17034
ZAR1	0.089795	0.45055	0.472876	0.303311	0.546915
ZBED3	-0.18457	-0.08243	-0.20629	-0.31731	-0.15353
ZBED4	0.042565	0.24859	-0.03014	0.188958	0.038107
ZBED5	-0.42564	-0.32065	-0.39314	-0.3316	-0.1543
ZBP1	0.273391	0.392448	0.555073	0.3792	0.586962
ZBTB10	-0.33546	-0.23086	-0.3456	-0.44383	-0.47644
ZBTB11 AC084198.31	0.115929	-0.34866	-0.0186	0.084928	-0.16474
ZBTB12	0.050475	-0.01559	-0.08677	0.036175	0.084399
ZBTB16	-0.13674	0.084867	-0.0393	0.038724	-0.01974
ZBTB17	0.092306	0.043043	0.112737	0.019626	-0.03906
ZBTB2	-0.49206	-0.20352	-0.41511	-0.40717	-0.37311
ZBTB20	-0.81251	-0.60912	-0.77394	-0.60795	-0.78115
ZBTB22	-0.21113	0.085866	-0.49052	-0.24633	-0.13033
ZBTB24	-0.23449	-0.2389	-0.32262	-0.26565	-0.41034
ZBTB25 ZBTB1	-0.0997	-0.04325	-0.0764	-0.10716	-0.14587
ZBTB26	-0.05951	-0.18835	-0.20406	-0.10186	-0.09562
ZBTB32	0.04476	0.198653	0.568753	-0.28401	0.145359
ZBTB33	0.094327	-0.1487	0.059004	-0.06117	-0.06089
ZBTB39	-0.09369	-0.11135	-0.15558	-0.37564	-0.28335
ZBTB4 POLR2A	-0.41529	-0.17257	-0.62939	-0.38082	-0.3472
ZBTB40	0.200775	0.32499	0.265971	0.21901	0.252947
ZBTB41	-0.47004	-0.82718	-0.86318	-0.62257	-0.60143
ZBTB43	0.133806	0.188091	0.094803	0.033722	0.075618
ZBTB44 AP002986.2	0.000146	0.17003	0.073122	0.100484	0.151047
ZBTB45	-0.30925	-0.32074	-0.35833	-0.3214	-0.45451
ZBTB46	0.337646	0.063245	0.139709	0.279843	0.110734
ZBTB47.	0.154518	0.301202	0.589375	0.193132	0.394529
ZBTB5	-0.47333	-0.5818	-0.42193	-0.32349	-0.41937
ZBTB6	-0.15657	-0.43556	-0.61629	-0.37378	-0.56462
ZBTB7A	-0.29818	-0.36971	-0.46626	-0.36244	-0.35187
ZBTB7B	0.371392	0.144463	0.451234	0.260958	0.13612
ZBTB7C	0.018618	0.255274	0.432872	0.146292	0.344375
ZBTB8OS RBBP4	-0.62954	-0.56558	-0.87964	-0.58718	-0.48345
ZC3H10	-0.62981	-0.87483	-0.86004	-0.56503	-0.73841
ZC3H12A	0.116001	0.314755	-0.10164	0.465017	0.454777
ZC3H12B	0.643814	0.011574	0.505757	0.152201	-0.18426
ZC3H13 CPB2	-0.39605	-0.87357	-0.84975	-0.82637	-0.80376
ZC3H15	-0.35677	-0.26063	-0.28571	-0.12454	0.013092
ZC3H3	0.107678	-0.04335	0.250241	0.237873	0.115873
ZC3H6	-0.01882	-0.25807	-0.53591	-0.45875	-0.14347
ZC3H7A	0.359644	0.552974	0.590157	0.462757	0.631931
ZC3H8	0.11769	0.088288	0.081034	-0.06756	0.039829
ZC3HAV1	0.296994	0.209386	0.326012	0.314406	0.30641
ZC3HAV1L	0.122079	0.173184	0.145116	0.185713	0.212184
ZC3HC1	0.139763	0.419265	0.107657	0.24226	0.019484
ZCCHC10	-0.15051	-0.09437	-0.45282	-0.34185	-0.48744
ZCCHC11	-0.40488	-0.45794	-0.28057	-0.36592	-0.27451
ZCCHC12	0.366176	0.294579	0.339779	0.063934	0.012112
ZCCHC13	0.478987	0.652971	0.81474	0.560314	0.647492

ZCCHC14	0.192932	0.341424	0.146272	0.204741	0.249097
ZCCHC2	0.763303	0.888089	0.857588	0.517495	0.43371
ZCCHC3	0.067041	0.13166	0.154782	0.075665	0.168041
ZCCHC5	-0.58092	-0.6113	-0.78913	-0.46262	-0.63833
ZCCHC6	0.049445	0.129193	0.03322	-0.05954	0.071841
ZCCHC7	-0.4403	-0.28025	-0.37263	-0.64353	-0.39036
ZCCHC8	0.170919	0.151187	0.233747	0.115177	0.067088
ZCCHC9	-0.43055	-0.51622	-0.95176	-0.41503	-0.73094
ZCRB1 PPHLN1	-0.34122	-0.40209	-0.55776	-0.56754	-0.57665
ZCSL2 OXNAD1	-0.33071	-0.11642	-0.43656	-0.28995	-0.05334
ZCWPW1 MEPCE	-0.30786	-0.20863	-0.15623	-0.3325	-0.1412
ZDHHC1	0.436293	0.309009	0.338092	0.215435	0.145035
ZDHHC11	0.492746	0.20573	0.257472	0.505249	-0.06559
ZDHHC12	-0.04053	-0.39691	-0.23949	-0.31571	-0.34064
ZDHHC13	0.006168	0.073416	0.142251	0.027813	0.201888
ZDHHC14	0.01088	-0.00094	0.227792	0.003952	0.18765
ZDHHC15	-0.21175	-0.55319	-0.01333	-0.39957	-0.28165
ZDHHC17	0.042496	0.144734	-0.0206	0.06366	0.21778
ZDHHC18	0.107644	0.13152	0.213484	-0.07043	0.29631
ZDHHC19	0.092752	0.121805	0.198056	0.245401	0.166572
ZDHHC2	-0.13062	-0.14916	-0.03378	-0.10906	-0.17265
ZDHHC20	0.191434	0.454504	0.219614	0.245737	0.225678
ZDHHC23	0.127996	0.12837	-0.05507	0.039588	-0.00543
ZDHHC24 ACTN3.	-0.29189	0.007761	0.262782	0.175881	0.074579
ZDHHC3 EXOSC7	-0.32538	-0.01094	-0.28385	-0.30781	-0.05257
ZDHHC4	0.250449	0.194129	0.194721	-0.13467	0.174662
ZDHHC5	-0.31209	-0.23824	-0.28738	-0.34274	0.022336
ZDHHC6 VTI1A	-0.40278	-0.51326	-0.76612	-0.57158	-0.67599
ZDHHC7	-0.09057	0.334293	-0.16321	0.219875	0.072424
ZDHHC8	0.035405	0.289678	0.192709	0.148342	0.093693
ZDHHC9	0.251949	-0.10543	0.222813	0.02762	-0.08577
ZEB1	-0.2726	-0.26282	-0.22235	-0.27723	-0.32662
ZER1	0.088482	0.087151	0.177013	0.205357	0.023597
ZFAND1	-0.07861	0.051279	0.042064	-0.02041	-0.11947
ZFAND2A	0.043307	-0.16284	-0.22521	-0.02735	0.036454
ZFAND2B	-0.14787	-0.33324	-0.43047	-0.22196	-0.31907
ZFAND3	-0.22965	-0.25522	-0.22596	-0.16061	-0.23178
ZFAND5	-0.15603	-0.14606	-0.18865	-0.11578	-0.13286
ZFAND6	-0.18871	-0.10287	-0.13493	-0.15953	-0.1729
ZFHX1B	-0.89185	-1.13561	-1.23715	-0.6143	-0.76481
ZFHX2	0.055226	0.308285	0.121143	0.195693	0.097097
ZFHX4	0.665424	0.629572	0.834196	0.252591	0.471103
ZFP1	0.040407	0.091636	0.111445	0.03849	-0.02578
ZFP106	-0.10296	-0.23902	0.229415	-0.02856	-0.20844
ZFP14	-0.2402	-0.19941	-0.43672	-0.24943	-0.29308
ZFP161	0.466936	0.757802	0.616047	0.044887	0.085305
ZFP2	0.127672	0.230922	0.272326	0.121414	0.117246
ZFP28	-0.24498	-0.19928	-0.26929	-0.21887	-0.14604
ZFP3	0.383764	0.627397	0.400184	0.560322	0.483491
ZFP30	-0.31585	-0.44759	-0.2076	-0.15202	-0.14003

ZFP36	0.002876	-0.12281	0.160595	0.060115	0.074085
ZFP36L1	-1.12374	-1.18015	0.748907	-0.92106	-0.57083
ZFP36L2	-0.22497	-0.05151	-0.1333	0.039595	-0.11504
ZFP37	0.054641	-0.20695	0.117595	0.28783	-0.31659
ZFP42	0.213101	0.310211	0.542788	0.433991	0.361455
ZFP64	-0.02225	-0.15026	-0.067	-0.04883	-0.06093
ZFP90	0.029861	0.07094	-0.00277	0.016249	0.050679
ZFPM1	0.034772	0.092249	-0.00774	0.03985	0.150315
ZFPM2	-0.42917	-0.47462	-0.21253	-0.52867	-0.48946
ZFR	0.045839	0.107754	-0.03278	-0.27549	0.107789
ZFX	-0.01728	0.032499	0.046801	0.040656	-0.00102
ZFY	-0.06171	0.224037	0.338602	0.008596	0.234479
ZFYVE16	0.075346	0.050085	0.037653	-0.15415	0.1583
ZFYVE20	-0.21642	0.179972	-0.05121	-0.26751	-0.10584
ZFYVE26	-0.01793	0.134368	0.134229	0.184348	-0.21485
ZFYVE27	0.309236	0.364179	0.38198	0.099519	0.305858
ZFYVE28 AL645924.13	-0.15456	-0.03698	-0.20812	0.051969	-0.11598
ZFYVE9	0.17497	0.126295	0.097522	0.039922	0.194262
ZGPAT LIME1	0.277426	0.373841	0.444734	0.254754	0.403662
ZHX1	-0.02289	-0.11688	-0.09262	-0.18363	-0.14491
ZHX2	-0.75072	-0.69684	-0.72801	-0.6298	-0.66156
ZHX3	0.268708	0.478596	0.399279	-0.05904	0.325845
ZIC1	-0.32561	-0.18288	-0.19946	0.257936	0.388981
ZIC2	-0.00462	0.053479	0.141172	0.052547	0.02293
ZIC3	0.221586	0.17974	0.338174	0.035485	0.354263
ZIC4	-0.56693	-0.18757	1.031155	0.366833	-0.22696
ZIC5	-0.02781	0.31939	0.626702	0.053273	0.609989
ZIK1	0.091273	0.234441	0.434198	0.436189	0.259424
ZIM3	0.339313	-0.62451	-0.15483	-0.09102	-0.11307
ZKSCAN1	0.017367	0.050631	-0.11458	-0.21982	-0.15119
ZKSCAN2	-0.1668	-0.16348	-0.11229	-0.05859	-0.17375
ZKSCAN4	-0.45249	-0.33638	-0.43107	-0.39615	-0.34078
ZKSCAN5	-0.27577	-0.29865	-0.45876	-0.24246	-0.40175
ZMAT2	0.346	0.165421	0.147511	0.306718	0.110468
ZMAT3	-0.04901	-0.10764	-0.12373	0.020941	0.018588
ZMAT4	0.011738	-0.08298	-0.27503	-0.49743	-0.61522
ZMAT5	-0.32236	-0.33169	-0.36673	-0.22682	-0.48621
ZMPSTE24	-0.68528	-0.76271	-0.9564	-0.51629	-0.31953
ZMYM1	0.071034	0.119796	-0.52984	-0.31429	0.068392
ZMYM2	-0.00947	-0.15521	-0.14482	-0.13555	-0.21059
ZMYM3	-0.16596	-0.469	-0.72346	-0.37486	-0.44926
ZMYM4	-0.72094	-0.42011	-0.70972	-0.48993	-0.20665
ZMYM5	-0.05915	-0.17799	-0.20172	-0.22726	-0.11056
ZMYM6	-0.07284	0.132477	-0.33547	-0.36776	-0.19553
ZMYND10	0.248919	0.321264	0.623289	0.248851	0.248718
ZMYND11	-0.4489	-0.22878	-0.21411	-0.20177	-0.28922
ZMYND12 PPCS	0.26298	0.145832	-0.03197	0.228872	0.424726
ZMYND17	-0.77594	-0.63946	-0.84205	-0.82573	-0.81414
ZMYND19	0.101586	0.015654	0.159903	0.152929	0.118149
ZMYND8	-0.61065	-0.9302	-0.65167	-0.34845	-0.75112

ZNF10	-0.13788	0.593006	-0.23044	-0.04256	-0.18997
ZNF100	-0.313	0.030302	-0.3284	-0.22666	-0.28709
ZNF101	0.080918	-0.04733	-0.18505	0.074327	0.046388
ZNF114	0.137114	-0.60844	-0.25721	0.268988	-0.38755
ZNF12	0.14937	0.073678	-0.1491	-0.11279	-0.01235
ZNF121	-0.11965	0.129885	-0.11925	-0.05005	-0.13951
ZNF124	0.161212	0.253312	0.184659	0.069309	0.180396
ZNF131	-0.19618	-0.07796	-0.20525	-0.27109	-0.15703
ZNF133	-0.30509	-0.41804	-0.6686	-0.3846	-0.68312
ZNF135	0.397566	0.539044	0.788952	0.432478	0.481908
ZNF136	0.104903	0.095833	-0.32375	-0.08762	-0.06059
ZNF137.	0.198068	0.238754	0.177444	-0.08427	-0.2268
ZNF138	-0.00239	0.175361	0.044218	0.069298	0.079
ZNF14	0.06027	0.319249	0.048482	0.123588	0.06616
ZNF140	-0.64707	-0.35983	-0.34777	-0.28668	-0.47174
ZNF141	0.0314	-0.03214	-0.10153	0.032721	0.038205
ZNF143	-0.4578	-0.38699	-0.52019	-0.41343	-0.23501
ZNF146	0.214585	0.170182	0.246603	-0.13531	0.054738
ZNF154	0.388952	0.584671	0.70398	0.515556	0.608709
ZNF155	-0.27289	-0.15694	-0.34582	-0.35906	-0.23635
ZNF157	0.049811	0.37636	-0.07889	0.075213	0.334449
ZNF16	0.23362	0.43565	0.611009	0.136823	0.368998
ZNF165	0.040513	-0.24235	0.050389	-0.36692	-0.36202
ZNF167	-0.33509	0.274591	-0.35027	-0.24637	0.106421
ZNF17	-0.09389	-0.56301	-0.62912	-0.12698	-0.80677
ZNF175	-0.80631	-0.79661	-0.85874	-0.79638	-0.90887
ZNF177	0.321645	0.522057	0.2729	0.23547	0.123941
ZNF179	0.287372	0.073544	-0.17721	-0.1438	0.027519
ZNF18	0.07805	-0.08106	-0.13951	0.093294	-0.12704
ZNF180	-0.05843	0.231226	0.312927	0.177499	0.140223
ZNF181	-0.07591	-0.1124	-0.14417	-0.17118	-0.1355
ZNF184	-0.30619	-0.00767	-0.20393	0.014695	-0.05145
ZNF185.	0.29486	0.421864	0.147511	0.348447	0.460023
ZNF187	-0.61493	-0.45187	-0.76376	-0.70163	-0.69155
ZNF19	0.501948	0.652754	0.650559	0.436147	0.471591
ZNF192	-0.58939	-0.37307	-0.66209	-0.48535	-0.50716
ZNF193	-0.56824	-0.29274	-0.59566	-0.63385	-1.02306
ZNF195	-0.05762	-0.00305	-0.14692	-0.19164	-0.05141
ZNF197	-0.18093	-0.19597	-0.34258	-0.32059	-0.08693
ZNF197 ZNF35	-0.30103	-0.23473	-0.33347	-0.3559	-0.15506
ZNF2	-0.5028	-0.37841	-0.66143	-0.47404	-0.21202
ZNF20	0.032464	-0.17738	-0.36005	-0.23476	-0.29234
ZNF200	0.290979	0.323773	0.369624	0.28958	0.406706
ZNF202	-0.48757	0.042004	-0.37334	-0.23141	-0.24596
ZNF205	-0.39456	-0.51983	-0.76702	-0.70587	-0.74998
ZNF211	0.091311	-0.00063	0.103927	0.100727	0.086329
ZNF212	0.1337	-0.03117	-0.11939	-0.03828	0.045915
ZNF213	-0.39228	-0.23936	-0.28179	-0.25203	-0.29151
ZNF214 NLRP14	-0.11705	-0.04347	-0.3864	0.498143	0.47372
ZNF215	-0.84387	-1.46854	-1.21742	0.136819	-0.69676

ZNF217	-0.20433	-0.39337	-0.2334	-0.4171	-0.66883
ZNF219	-0.27408	-0.28654	-0.49237	-0.42235	-0.5138
ZNF221	-0.23702	0.131042	-0.35702	-0.26285	-0.31288
ZNF222	0.015458	0.391407	-0.17889	-0.19765	-0.08914
ZNF224	-0.74551	-0.56874	-1.10237	-0.66604	-1.10777
ZNF225	-0.71596	-0.40768	-0.82696	-0.68952	-0.80237
ZNF226	-0.98364	-0.94505	-1.11853	-0.98252	-1.16261
ZNF227	-0.10696	-0.06683	-0.09699	-0.06778	-0.14613
ZNF228	-0.09854	0.090407	-0.41791	0.402775	-0.34832
ZNF23	-0.03153	-0.04412	-0.07107	-0.10253	-0.18488
ZNF230	-0.41974	-0.44183	-0.53125	-0.3642	-0.60945
ZNF232 USP6	0.180856	0.214961	0.406983	0.236657	0.255706
ZNF233	-0.16529	0.672401	-0.36833	0.476502	-0.4606
ZNF235	0.351721	0.5931	0.605763	0.223816	0.373607
ZNF236	0.378052	0.511208	0.879998	0.549949	0.649728
ZNF238	-0.22962	-0.06731	-0.52638	-0.87203	-0.54651
ZNF239	0.417115	0.478724	0.68131	0.129322	-0.03506
ZNF24	-0.21178	0.035917	-0.3352	-0.18903	0.202468
ZNF248	-0.22872	-0.05337	0.01467	-0.22129	-0.08747
ZNF25	0.347527	0.238047	0.15995	0.209899	-0.06463
ZNF250	-0.22713	-0.18913	-0.11223	-0.3404	0.031024
ZNF254	-0.14961	0.170385	-0.07602	-0.06863	0.044474
ZNF256	0.364287	0.326782	0.482853	0.28828	0.321587
ZNF257.	-0.23685	0.005438	-0.56341	0.019538	0.004378
ZNF259	-0.37568	-0.55475	-1.15117	-0.69246	-1.01191
ZNF26	0.065663	-0.1407	-0.02812	-0.15884	0.051659
ZNF260	-0.72862	-0.33384	-0.80764	-0.72859	-0.74918
ZNF263	-0.1744	-0.26697	-0.21293	-0.1332	-0.10273
ZNF264	0.329177	0.290544	0.392921	0.261272	0.308159
ZNF266	-0.434	-0.19342	-0.37607	-0.27904	-0.49169
ZNF267	0.044471	0.139959	0.07627	-0.08299	0.167454
ZNF268.	0.379297	0.574723	0.760774	0.299189	0.31551
ZNF273	-0.14521	-0.1928	-0.31754	-0.02701	-0.14052
ZNF274	-0.26953	-0.20195	-0.24279	0.144767	-0.52889
ZNF277P	-0.48862	-0.37145	-0.89993	-0.41617	-0.56149
ZNF28	0.015987	0.28141	-0.14593	-0.04585	-0.02698
ZNF280A	0.543987	0.505095	0.800742	0.39979	0.500057
ZNF280B	0.390377	0.385301	0.365414	-0.04123	0.302055
ZNF280C	0.460286	0.374479	0.216913	0.18184	0.336825
ZNF281	-0.46196	-0.50658	-1.06684	-0.31882	-0.65321
ZNF282	0.243592	0.134236	0.041434	-0.04763	0.131687
ZNF283	0.351642	0.757338	0.664203	0.331714	0.268292
ZNF284	0.367704	0.11541	0.069972	0.090112	-0.00367
ZNF287	0.158593	0.207711	0.072854	0.147621	0.338538
ZNF294	-0.33699	-0.40389	-0.67554	-0.60966	-0.57565
ZNF295	-0.06389	0.03563	0.182894	0.131315	0.021734
ZNF300	-0.23599	0.741665	-0.55573	-0.89503	-0.75823
ZNF304	-0.16496	-0.13236	-0.58185	-0.01841	-0.29112
ZNF311	0.548075	-0.05983	0.4983	0.141384	0.500553
ZNF313	0.078606	-0.12423	0.041409	0.096608	-0.0366

ZNF317	-0.27126	-0.63744	-1.00584	-0.68598	-0.8021
ZNF318	0.117262	0.30041	0.215422	0.210119	0.138768
ZNF319 C16orf57	-0.08086	0.005373	-0.13889	0.032956	0.022598
ZNF32	0.228377	0.362014	0.268735	0.221429	0.365155
ZNF320	-0.32889	-0.05829	-0.36618	-0.34491	-0.20885
ZNF320 ZNF816A	-0.11831	0.224852	-0.07528	-0.09786	-0.07128
ZNF322A	-0.19809	-0.23237	-0.03004	-0.11439	-0.16042
ZNF322B	0.678529	0.493869	0.661991	0.330514	0.335392
ZNF323 ZKSCAN3	0.273864	0.281879	0.353099	0.251689	0.297553
ZNF324	0.014259	-0.24266	-0.03989	0.033908	-0.04663
ZNF326	-0.00559	-0.04659	0.045983	-0.10026	0.159701
ZNF330	0.426212	0.462132	0.566071	0.220155	0.239283
ZNF331	0.075345	0.277514	0.133692	0.144568	0.087323
ZNF333	-0.21029	-0.09925	-0.41951	-0.30428	-0.35993
ZNF334	0.284859	0.297902	0.384294	0.168669	0.241294
ZNF335	0.17469	0.191062	0.223821	0.163641	0.089598
ZNF337	-0.16956	-0.05859	-0.15175	-0.12653	-0.21677
ZNF33B	-0.11818	-0.07507	-0.14674	0.032529	0.098744
ZNF341	0.231896	0.084834	0.432765	0.154998	0.290532
ZNF342	0.279365	0.085163	0.407224	0.241061	-0.1462
ZNF343	-0.05627	-0.354	-0.27991	0.038302	-0.14728
ZNF345	-0.61487	-0.46163	-0.70879	-0.55256	-0.65526
ZNF346	0.170914	0.297343	0.087062	0.088682	0.075948
ZNF347	-0.17212	0.351826	-0.49256	-0.38302	-0.22978
ZNF350	0.149519	0.836534	0.53743	0.312638	0.320754
ZNF354A	0.195001	0.556467	0.433317	0.169743	0.383944
ZNF354B	-0.0174	-0.07347	-0.21028	-0.17127	-0.11681
ZNF354C	0.239138	0.419933	0.220077	0.393047	0.200109
ZNF358	-0.10144	-0.21355	-0.0497	-0.15651	-0.25543
ZNF366	0.526087	0.663415	0.654524	0.466488	0.54359
ZNF367	-0.11822	-0.07635	0.064926	-0.0962	-0.0598
ZNF37A	0.143946	0.191658	-0.06793	0.172268	-0.01511
ZNF382	-0.32145	-0.36927	-0.5017	-0.32798	-0.31152
ZNF383	0.122246	0.429797	0.612022	0.203106	0.313889
ZNF384	-0.50971	-0.60769	-0.71615	-0.70711	-0.7974
ZNF385A	0.148454	-0.21066	0.041208	-0.09991	0.074761
ZNF385C	0.307894	0.433846	0.560367	0.388617	0.478759
ZNF391	-0.14963	-0.09512	-0.19366	0.172537	-0.09506
ZNF394	-0.03183	0.166284	-0.12079	-0.17692	-0.06765
ZNF395	-0.20698	-0.22639	-0.49808	0.011409	-0.25085
ZNF396	0.311084	0.213726	0.272402	0.342378	0.346822
ZNF397	-0.49102	-0.13674	-0.44038	-0.43814	-0.34526
ZNF397OS ZNF271	-0.13556	-0.4613	-0.65874	-0.45478	-0.07356
ZNF398	0.205194	0.052452	0.024003	0.16528	0.224546
ZNF403	-0.12021	-0.20341	-0.25692	-0.09832	-0.19258
ZNF407	0.58859	0.630888	0.685187	0.287778	0.59357
ZNF41 CXorf24	0.204004	-0.20386	0.324685	-0.14457	-0.0866
ZNF410	0.086289	0.028971	0.109151	0.109152	0.016694
ZNF414	0.052201	-0.00175	-0.26539	-0.02361	-0.10824
ZNF415	0.820843	0.1543	-0.51349	-0.45577	-0.07456

ZNF416	-0.08758	-0.03137	-0.05013	-0.16212	0.043345
ZNF417	-0.3604	-0.18776	-0.41959	-0.25672	-0.32572
ZNF418	0.334743	0.781865	0.933667	0.622312	0.2832
ZNF419	-0.392	-0.12926	-0.54491	-0.44493	-0.42671
ZNF420	-0.07117	-0.03002	-0.04411	-0.29757	-0.34556
ZNF423	0.44908	0.664834	0.670227	0.451833	0.854787
ZNF425	-0.27417	-0.28361	-0.44719	-0.43069	-0.22225
ZNF426	-0.07946	-0.03691	-0.41278	-0.18327	-0.50259
ZNF428	0.289525	0.076214	0.158593	-0.01057	0.290084
ZNF429	-0.32513	-0.32123	-0.58153	-0.43829	-0.36796
ZNF43	-0.0891	0.069539	-0.25071	-0.02991	-0.01686
ZNF430	-0.06126	0.03767	-0.62165	0.071365	-0.01587
ZNF431	0.160006	0.15394	-0.19205	0.029502	0.271634
ZNF433	-0.13116	-0.04767	0.01423	-0.24129	-0.0264
ZNF434 ZNF174	-0.0385	-0.09763	-0.17856	-0.13653	-0.1586
ZNF435	-0.03734	-0.72254	-0.82652	-0.16836	-0.92805
ZNF436	-0.34983	-0.36783	-0.32913	-0.12984	-0.0953
ZNF438	0.016642	0.129048	0.041928	0.034339	0.078097
ZNF439	0.319112	0.148692	0.098417	0.095967	0.174854
ZNF44	0.070032	0.119938	0.074449	0.017423	-0.14107
ZNF440	0.102169	0.315377	0.170935	0.096583	0.160112
ZNF441	-0.11388	-0.05748	-0.02712	-0.09318	-0.11229
ZNF442	-0.26133	-0.3344	-0.34879	-0.26135	-0.27209
ZNF443	0.072527	-0.25115	-0.40962	-0.1991	-0.50763
ZNF444	-0.29454	-0.28979	-0.72667	-0.38017	-0.70515
ZNF446	-0.02938	-0.00285	-0.06845	-0.0104	0.018688
ZNF45	0.336875	0.329927	0.308296	0.017987	0.164134
ZNF451	-0.36087	-0.43723	-0.63973	-0.52232	-0.47705
ZNF452	0.256231	-0.00867	-0.48028	-0.39593	-0.59236
ZNF454	0.076865	0.471328	0.495966	0.445811	0.202115
ZNF460	-0.01376	-0.01871	-0.08769	-0.10005	-0.00554
ZNF461	-0.25618	-0.32468	-0.50019	-0.34952	-0.60936
ZNF462	-0.54663	-0.67421	-0.66484	-0.51409	-0.52623
ZNF467	-0.41028	-0.5204	-0.67771	-0.37652	-0.33046
ZNF471	-0.08086	0.347874	-0.51934	-0.00669	-0.43455
ZNF474	-0.40577	-0.3115	-0.42087	-0.43419	-0.6719
ZNF480	-0.13582	-0.36773	-0.28828	-0.2812	-0.15491
ZNF483	0.261486	0.235346	0.341958	0.282003	0.319865
ZNF484	0.406038	0.53225	0.599355	0.297821	0.407077
ZNF488	-0.0597	-0.04303	-0.06258	-0.01483	-0.04209
ZNF490 ZNF791	-0.25677	-0.38888	-0.8163	-0.4104	-0.54065
ZNF491	-0.07394	-0.07336	-0.12757	-0.10015	-0.21112
ZNF493	-0.06627	0.025397	-0.15572	-0.11708	-0.07424
ZNF496	0.083295	-0.00277	0.047522	0.092175	0.171813
ZNF497	0.159284	0.100699	0.072885	0.153677	0.246239
ZNF498	-0.05423	-0.00825	-0.12291	0.120522	-0.21851
ZNF500	0.223633	0.105422	0.228944	0.230116	0.227047
ZNF501	0.252364	0.397941	0.57197	0.309976	0.133322
ZNF502	-0.19329	0.676704	-0.46986	-0.31107	-0.47306
ZNF503	-0.48999	-0.26116	-0.43026	-0.35048	-0.35419

ZNF507	0.205665	0.480971	0.589332	0.288	0.364208
ZNF510	-0.54588	-0.24305	-1.06825	-0.78267	-0.78156
ZNF512	0.136573	0.240791	0.140303	0.163768	0.339152
ZNF512B PRR17	0.04711	0.00202	0.111748	0.097131	-0.21124
ZNF513 PPM1G	0.224353	0.214157	0.248075	0.233957	0.061544
ZNF514	0.097519	-0.08858	-0.02199	-0.07803	-0.03141
ZNF516 AC018413.10	0.033612	0.166726	-0.17911	-0.20174	0.057783
ZNF517	0.284715	0.268931	0.213755	0.12486	0.198694
ZNF518A	0.327571	0.427602	0.433481	0.396576	0.31025
ZNF518B	0.25407	0.56763	0.509506	0.274451	0.550226
ZNF519	-0.23764	-0.08104	-0.34726	-0.35696	-0.36871
ZNF521	-0.1796	-0.0133	-0.02604	-0.15219	-0.09887
ZNF529	-0.47463	-0.61978	-0.88457	-0.43616	-0.55956
ZNF530	0.288996	0.456454	0.505039	0.185109	0.260271
ZNF532	0.466194	0.711799	0.803788	0.184304	0.167839
ZNF534	-0.19074	0.520712	-0.11532	-0.46418	-0.3772
ZNF536	0.369241	0.280417	0.408877	0.118064	0.294015
ZNF541.	0.27819	0.328639	0.462708	0.282437	0.402642
ZNF543	-0.11689	-0.28208	-0.16578	-0.03967	-0.14356
ZNF544	0.042076	0.013363	0.33246	0.305468	0.254399
ZNF545	0.209876	0.17132	0.075924	0.008162	0.063361
ZNF546	0.072998	-0.10748	-0.41998	-0.22707	-0.15943
ZNF547	0.112179	-0.05574	-0.78911	0.018513	-0.05334
ZNF548	0.374471	0.502976	0.505344	0.217607	0.378191
ZNF549	-0.14617	-0.01029	-0.1839	-0.1092	-0.06391
ZNF551	0.039437	0.275881	0.286527	0.143067	0.092349
ZNF552	0.032446	-0.16094	-0.13044	-0.04623	-0.17325
ZNF554	0.200981	0.253183	0.38975	0.188929	0.326914
ZNF555	0.118531	0.048491	-0.01818	0.036124	-0.05525
ZNF556	0.304724	-0.21655	-0.34652	0.208298	-0.20553
ZNF557	-0.29558	-0.34716	-0.86334	-0.57456	-0.61067
ZNF558	0.424232	0.805249	0.518801	0.385086	0.618498
ZNF559	0.31139	0.203357	5.18E-05	0.169096	0.063754
ZNF560	0.0605	0.602094	0.126529	0.610527	0.308297
ZNF562	-0.00531	0.050548	-0.48252	-0.11886	-0.34387
ZNF563	-0.0299	-0.0876	-0.06878	-0.03517	0.016454
ZNF564	-0.11563	-0.15958	-0.29555	-0.22364	-0.32879
ZNF565 ZNF146	-0.51036	-0.48354	-0.68606	-0.39693	-0.55958
ZNF566	-0.57311	-0.35711	-0.58088	-0.38245	-0.55959
ZNF567	0.290314	0.515206	0.482809	0.263493	0.251255
ZNF569 ZNF570	0.138125	0.002776	-0.12396	0.018415	0.146481
ZNF571 ZNF540	-0.05525	-0.38257	-0.67564	-0.27434	-0.55767
ZNF572	0.402819	0.366011	-0.23628	0.163747	-0.35014
ZNF573	-0.32891	-0.22368	-0.59507	-0.39063	-0.44813
ZNF574	-0.19103	0.056173	-0.29062	0.006912	0.188433
ZNF575	-0.32757	-0.37268	-0.47792	-0.00113	-0.52554
ZNF577	-0.60035	0.737375	0.619231	0.501614	-0.95896
ZNF579	0.144015	-0.27993	0.0035	-0.08285	0.047142
ZNF580 ZNF581	-0.47795	-0.66389	-0.2409	-0.32954	-0.67059
ZNF582	0.591349	0.866174	0.830108	0.433939	0.511549

ZNF583	-0.02006	0.097035	0.034578	0.04103	-0.29903
ZNF585A	-0.09758	0.224215	-0.68266	-0.50783	-0.56066
ZNF585B	0.488235	0.674039	-0.50871	0.143724	-0.30806
ZNF586	0.273552	0.751369	0.656536	0.485592	0.403652
ZNF587	-0.53167	-0.18053	-0.64509	-0.44723	-0.56272
ZNF588.	-0.11908	-0.05128	-0.30475	-0.07697	-0.01339
ZNF589	0.157933	0.570408	0.251959	0.239816	0.313834
ZNF595	0.407311	-0.27824	-0.41111	0.450773	0.393967
ZNF597 AC004224.1	0.129951	-0.0781	-0.04725	0.140818	0.055683
ZNF598	0.138275	0.122948	-0.07974	-0.02644	0.002995
ZNF599	-0.24107	-0.09079	-0.44799	-0.48936	-0.20099
ZNF600	-0.01494	0.230828	-0.24699	-0.08272	0.27025
ZNF605	0.082976	0.208368	-0.04049	-0.01557	-0.06511
ZNF606 AC008969.6	0.153122	0.38978	0.667429	0.254671	0.389503
ZNF607	0.060313	0.243651	0.052844	0.063875	0.143891
ZNF608	-0.69534	-0.85049	-1.07096	-0.49234	-0.76048
ZNF609	0.268436	0.332741	0.265793	0.032875	0.106035
ZNF610	0.595188	0.601559	0.811466	0.322336	0.371493
ZNF613	-0.8486	-0.25928	-0.49435	-0.26171	-0.19969
ZNF615	0.389692	0.604558	0.72757	0.359106	0.062256
ZNF616	-0.21039	0.028504	-0.28805	-0.3761	-0.64989
ZNF619	0.562821	0.307161	0.612683	0.408526	0.154807
ZNF620	0.129004	0.409561	0.424377	0.128455	-0.03919
ZNF621	0.001712	-0.20425	-0.38298	-0.1984	-0.10212
ZNF622	-0.13264	-0.28684	-0.29288	-0.18543	-0.25079
ZNF623	0.367679	0.350768	0.542091	0.255032	0.416444
ZNF624	0.111393	0.247551	-0.45452	-0.28764	-0.07806
ZNF625	0.135274	-0.04236	-0.08248	-0.11091	0.021162
ZNF626	0.068235	0.306362	-0.05339	0.256738	0.094635
ZNF627	0.211187	-0.14858	0.090354	0.079872	-0.14353
ZNF628	0.269326	0.269122	0.211036	0.016734	0.243171
ZNF638	-0.23214	-0.11829	-0.16124	-0.1966	-0.26934
ZNF639	-0.15044	-0.07204	0.024015	0.004671	-0.17555
ZNF641	-0.3614	-0.28762	-0.34556	-0.25471	-0.17886
ZNF642	0.102799	0.170298	0.076983	0.085485	0.260031
ZNF643	0.130276	0.289262	-0.04285	-0.09479	0.095022
ZNF644	0.108202	-0.00455	0.156211	-0.30902	0.174476
ZNF645	0.422478	0.42111	0.582029	0.345667	0.354173
ZNF648	-0.22208	-0.82858	-0.37426	-0.68598	-0.66549
ZNF649	-0.57384	0.193081	-0.3283	-0.37362	-0.58629
ZNF652	-0.00217	0.256444	0.358564	0.041671	0.176853
ZNF653 ECSIT	0.218802	0.203194	0.366227	0.248663	0.207295
ZNF654	0.610033	0.948959	0.810597	0.495089	0.754743
ZNF655	-0.18056	-0.33337	-0.72331	-0.30146	-0.26275
ZNF658	0.347858	0.136414	-0.23072	-0.31319	-0.31584
ZNF658B	-0.83409	-0.65589	-0.87572	-0.58102	-0.72921
ZNF659	-0.06649	0.510737	-0.7151	-0.65683	-0.64967
ZNF660	-0.51943	-0.35057	-0.657	-0.6057	-0.38902
ZNF662	0.371709	0.457793	0.539038	0.254859	0.366348
ZNF665	-0.02387	0.274835	-0.11162	-0.18485	-0.40846

ZNF667	-0.25314	0.178571	-0.31591	-0.131	0.011829
ZNF668 ZNF646	-0.41992	-0.42102	-0.51425	-0.43606	-0.67631
ZNF669	0.20309	0.276752	0.149513	0.094923	0.216976
ZNF670	0.130717	-0.01152	0.350141	0.101829	0.021038
ZNF671	0.382039	0.752611	0.905691	0.616476	-0.14255
ZNF672	-0.13214	-0.21279	-0.27948	-0.1914	-0.15277
ZNF673	0.019341	0.068679	-0.1915	-0.06776	-0.23117
ZNF674	0.14984	0.114831	-0.0638	0.012988	0.013476
ZNF675	-0.18497	-0.33802	-0.6014	-0.27602	-0.21931
ZNF677	0.198077	0.834	0.596967	0.196447	0.086028
ZNF678	0.203386	0.14693	-0.0995	-0.04011	-0.0307
ZNF680	-0.25864	-0.50263	-0.71992	-0.27961	-0.36348
ZNF681	0.201866	0.315561	0.077268	0.202622	0.137298
ZNF682	0.296313	0.242223	0.52073	0.094826	0.205728
ZNF683	0.240699	-0.26544	0.205906	-0.64554	0.050423
ZNF684	-0.35239	-0.18979	-0.89776	-0.54138	-0.57858
ZNF687	-0.34561	-0.33395	-0.53782	-0.46711	-0.21827
ZNF689	0.045398	0.178877	0.054707	0.010673	-0.01866
ZNF69	-0.02255	0.333573	-0.0308	0.04085	0.029933
ZNF690 TUBGCP4	-0.13874	-0.01525	-0.22595	-0.20704	-0.12762
ZNF691	-0.34811	-0.81096	-0.92904	-0.77361	-0.56927
ZNF692	0.09458	0.055264	-0.04784	-0.337	0.09316
ZNF695	-0.02121	0.142196	0.024098	-0.07542	0.038691
ZNF696	0.382121	0.306111	0.336824	0.245506	0.247351
ZNF70	-0.01059	0.114659	0.249209	0.098103	0.142021
ZNF700	-0.03176	0.307655	-0.11522	-0.05805	-0.06889
ZNF701	-0.20146	0.2256	-0.45681	-0.24756	-0.26862
ZNF702. ZNF160	-0.30977	-0.06189	-0.50132	-0.34148	-0.28725
ZNF703	-0.45392	-0.35607	-0.42026	-0.34308	-0.32416
ZNF704	0.459754	0.30978	0.291386	0.175449	0.335215
ZNF705A	0.121449	-0.81434	-0.3627	-0.76548	-0.96416
ZNF706	-0.17819	-0.05775	-0.00619	-0.17137	-0.13978
ZNF707	0.115732	0.266067	0.224941	0.159602	-0.01078
ZNF708	-0.14475	0.058145	-0.00598	0.083346	0.174419
ZNF709	-0.30478	-0.05111	-0.24803	-0.06114	-0.26287
ZNF71	-0.76152	-0.84655	-0.94512	-0.95908	-0.97099
ZNF710	-0.26453	-0.057	0.072868	-0.08056	-0.04993
ZNF711	0.107029	0.003441	0.300388	-0.07372	0.07861
ZNF713	0.070059	0.497413	0.267669	0.13594	0.07485
ZNF714	0.053684	-0.00019	-0.14812	-0.02249	-0.02523
ZNF718.	-0.02196	0.1469	0.145997	0.350819	-0.08664
ZNF720.	-0.45188	-0.50014	-0.68366	-0.29632	-0.52659
ZNF721 PIGG	0.200839	0.164901	-0.12682	0.256278	0.138129
ZNF746	0.00982	0.030353	0.156438	-0.0344	0.05876
ZNF749	0.258221	0.524511	0.233668	-0.15595	0.409737
ZNF75 ZNF449	-0.03373	-0.44925	-0.04362	-0.3726	-0.44689
ZNF76	-0.18155	-0.0323	-0.17169	-0.04843	-0.1425
ZNF763	-0.24058	-0.21432	-0.54785	-0.17683	-0.32769
ZNF765	-0.38225	0.052515	-0.6499	-0.16345	-0.3892
ZNF767	0.101118	0.180448	0.279605	0.127921	0.108748

ZNF770	-0.42833	-0.61026	-0.49003	-0.24405	-0.5957
ZNF772 ZNF419	0.144132	0.076895	0.088058	0.185321	0.203367
ZNF773	-0.16779	-0.12871	0.01898	-0.21059	-0.06949
ZNF774	0.210059	0.098991	-0.02006	0.128891	0.180706
ZNF775	-0.35803	-0.14019	-0.12039	-0.06424	-0.1302
ZNF776	0.156691	-0.04239	-0.43949	-0.28957	-0.12703
ZNF777	0.440437	0.773945	0.672632	0.503637	0.652826
ZNF780A	0.220279	0.391663	0.06375	-0.12418	0.198806
ZNF781	0.397146	0.540081	0.096239	-0.03345	0.148812
ZNF783	0.115122	0.265652	-0.00446	0.0424	0.203147
ZNF784	0.040968	-0.14771	-0.58502	-0.43425	-0.28512
ZNF787	0.018853	0.199491	-0.10596	0.024545	0.131981
ZNF789	-0.12267	-0.06979	-0.17701	-0.12329	-0.23003
ZNF79	-1.06613	-0.66896	-1.10552	-0.75878	-1.00449
ZNF790	-0.43085	-0.1685	-0.48093	0.105849	-0.19115
ZNF792	0.189151	0.459192	0.40825	0.187362	0.520019
ZNF8	-0.16823	-0.17214	-0.03733	-0.20249	-0.16644
ZNF800	-0.26263	-0.0674	-0.23718	-0.15426	-0.13003
ZNF806	0.491302	0.366218	0.181192	0.125131	-0.03217
ZNF808	0.608153	0.543744	0.684181	0.363683	0.294472
ZNF81	-0.0709	-0.34844	-0.75947	-0.30734	-0.35965
ZNF812	-0.54905	-0.63821	-0.47777	-0.50786	-0.59572
ZNF818.	-0.14165	-0.04212	-0.2307	-0.48682	-0.15411
ZNF821	-0.22558	-0.36664	-0.31931	-0.13967	-0.3809
ZNF826	0.188763	0.260822	0.163648	0.235878	0.226618
ZNF827	-1.04842	-1.21668	-0.79631	-0.95458	-0.92179
ZNF829 ZNF568	-0.35935	0.481828	-0.77063	-0.31617	-0.31581
ZNF83	-0.23757	0.116687	-0.4673	-0.14828	-0.23939
ZNF84	-0.06829	-0.05395	-0.21434	0.065059	-0.07526
ZNF85	-0.07728	-0.05429	-0.1803	0.146182	-0.0332
ZNF91	-0.43096	-0.18864	-0.54513	-0.16858	-0.35477
ZNF92	-0.06317	-0.11135	-0.36095	-0.02692	-0.13698
ZNF93	0.05893	0.011355	-0.16821	-0.07534	-0.30511
ZNFX1 C20orf199.	-0.94591	-0.91845	-0.92697	-0.83815	-0.75147
ZNHIT2	0.285267	0.318656	0.42536	0.264346	0.341714
ZNHIT3	-0.26128	0.013144	-0.07211	-0.20442	-0.14045
ZNRF1	0.177533	0.033235	0.179472	0.1941	0.007704
ZNRF2	0.197549	0.121696	0.16777	0.092401	0.288939
ZNRF4	0.3769	0.507063	0.407998	0.388697	0.437614
ZP1	0.351483	0.270834	0.283947	0.025195	0.222264
ZP2	0.107857	0.337321	-0.01332	-0.52261	-0.68465
ZP4	0.27238	0.713405	0.611919	0.347099	-0.05906
ZPBP	0.347731	0.596381	0.813102	0.459101	0.508251
ZPBP2	0.458076	0.782353	0.660531	0.440955	0.575286
ZPLD1	-0.41967	-0.15649	-0.68184	-0.38332	-0.81142
ZRANB1	0.649313	0.628989	0.851371	0.304324	0.548902
ZRANB2	-0.16672	-0.38139	-0.26291	-0.29648	-0.22537
ZRANB3 R3HDM1	-0.80271	-0.613	-0.89345	-0.74291	-0.6192
ZRSR2	-0.12562	0.072622	-0.24346	-0.0219	-0.02178
ZSCAN1	0.194349	0.311074	0.202396	0.258901	0.356468

ZSCAN10	0.366556	-0.13	0.579386	0.194477	0.202125
ZSCAN12	-0.14866	-0.3445	-0.66305	-0.33043	-0.50788
ZSCAN18	-0.59194	0.068837	-0.87525	-0.41364	-0.65374
ZSCAN2	-0.21722	-0.33217	-0.33383	-0.32888	-0.29587
ZSCAN20	-0.14773	-0.28179	-0.41726	-0.42984	-0.09901
ZSCAN21	0.275387	0.421509	0.48577	0.278687	0.434138
ZSCAN22	-0.35966	-0.51856	-0.6696	-0.45742	-0.5423
ZSCAN4	0.247568	-0.36028	0.18397	-0.01379	-0.07652
ZSCAN5	0.580021	0.326371	0.749662	0.389315	0.419878
ZSCAN5 ZNF542.	0.171833	0.649194	0.219427	0.02569	0.166041
ZSWIM1	0.019854	0.15806	-0.02942	0.073596	0.175175
ZSWIM2	-0.24288	-0.30422	-0.20451	0.004523	-0.08403
ZSWIM4	-0.18845	-0.20435	-0.211	-0.03467	-0.10955
ZSWIM7 TTC19	0.045434	-0.00361	0.069558	-0.03675	0.05976
ZUFSP	0.384997	0.515832	0.575442	0.368912	0.428974
ZW10	-0.42117	-0.22111	-0.54506	-0.287	-0.4759
ZWINT	-0.18026	-0.24736	-0.46005	-0.29929	-0.29698
ZXDA	0.223147	0.29858	0.00024	0.111957	-0.17574
ZXDB	0.350096	0.205642	0.173355	0.274784	0.096205
ZXDC	0.159435	0.311573	0.46095	0.209814	0.281561
ZYG11A	0.080349	0.279322	0.265669	0.287726	0.395281
ZYG11B	-0.10567	-0.14335	-0.24311	-0.34052	-0.38057
ZYX	-0.247	-0.10298	-0.12845	-0.03885	-0.1744
ZZEF1 CYB5D2	-0.31555	0.03765	-0.07429	-0.12887	0.014347
ZZZ3	-0.85028	-0.71392	-0.72201	-0.74785	-0.43142
