# Supplementary material for: Genome‐wide DNA methylation analysis identifies MEGF10 as a novel epigenetically repressed candidate tumor suppressor gene in neuroblastoma
Source: Mol Carcinog. 2016 Nov 29;56(4):1290–301. doi: 10.1002/mc.22591 (PMC5396313; doi:10.1002/mc.22591)
Supplement: Supplementary file 5 — supplementary Table S4 [file MC-56-1290-s005.pdf]

Table S4: Oligonucleotide primers used in the study

Application	Gene / oligonucleotides F = forward, R = Reverse		5' - 3' sequence
Genomic DNA QPCR	*SNRPN*	F	TACATCAGGGTGATTGCAGTTCC
		R	TACCGATCACTTCACGTACCTTCG
	TBP	F	GCCCGAAACGCCGAATAT
		R	CCGTGGTTCGTGGCTCTCT
	WISP3	F	TGTACGTGAGGGTGAAGCTG
		R	ACCTGTTCCTGGAGAGCAGA
RT-PCR (endpoint and QPCR)	*ALOX15*	F	GCCCCTCTAGTCATGCTGAA
		R	AGGCAAGAAAAGGGGAGGT
	FAS	F	AACTTGGAAGGCCTGCATC
		R	CAGTCTGGTTCATCCCCATT
	FOXG1	F	GCGCAACTTGAAGCAACTTT
		R	TCTGGTCGCTGTACGTGTTG
	GAPDH	F	GTTCGACAGTCAGCCGCATC
		R	GGAATTTGCCATGGGTGGA
	HOXA11	F	CAGCCCCGAGTCGTCTTC
		R	CAGCTCTCGGATCTGGTACTT
	HOXD3	F	GGATGAAAGAGTCTCGACAGAAC
		R	GTACCCGCTTGGATGCTG
	KLHL14	F	GACGCCATGAACTACCACCT
		R	CAGCCCTCCAACCAATAACA
	MEGF10	F	ACTGGATTGGGACAGCATCA
		R	GGATGTGGGTATGACTCTTGC
	OTX2	F	AGAGGAGGTGGCACTGAAAA
		R	GCTGTTGTTGCTGTTGTTGG
	TBP	F	GCCCGAAACGCCGAATAT
		R	CCGTGGTTCGTGGCTCTCT
	TBX4	F	TCCAGAAGCTGAAGCTGACA
		R	ATCAGCCTTAACGATGTGGAG
	TGFB2	F	TTCAGACACTCAGCACAGCA
		R	TTGGGTGTTTTGCCAATGTA
	ULBP	F	TGGAGAATTTAATACCCATTGAGC
		R	TGTCCATTGAAGAGGAACTGC

ChIP	*GAPDH*	F	TACTAGCGGTTTTACGGGCG
		R	TCGAACAGGAGGAGCAGAGAGCGA
	MEGF10 prom	F	GAACACCTCCCAGGGACTCT
		R	GGTCGCGAAGTTAGCAAATG
	MEGF10 EX1	F	GTTCCTCTTTCCCGCTTCTC
		R	AGCCAGCCAAGACACAAAAT
