# Supplementary material for: Genome‐wide DNA methylation analysis identifies MEGF10 as a novel epigenetically repressed candidate tumor suppressor gene in neuroblastoma
Source: Mol Carcinog. 2016 Nov 29;56(4):1290–301. doi: 10.1002/mc.22591 (PMC5396313; doi:10.1002/mc.22591)
Supplement: Supplementary file 6 — supplementary Table S5 [file MC-56-1290-s006.pdf]

**Table S5: Gene ontology of methylated genes**

Gene ontology of the genes identified by MCIP in neural crest (hNCC) or neuroblastoma cell lines (NB) were assessed using the functional classification in PANTHER (http://www.pantherdb.org/).

| hNCC not shared | Category name (Accession) | # genes | Percent of gene hit against total # genes | Percent of gene hit against total # Process hits |
|---|---|---|---|---|
| 1 | cellular component organization or biogenesis (GO:0071840) | 6 | 6.30% | 3.60% |
| 2 | cellular process (GO:0009987) | 41 | 42.70% | 24.30% |
| 3 | localization (GO:0051179) | 11 | 11.50% | 6.50% |
| 4 | biological regulation (GO:0065007) | 21 | 21.90% | 12.40% |
| 5 | response to stimulus (GO:0050896) | 24 | 25.00% | 14.20% |
| 6 | developmental process (GO:0032502) | 8 | 8.30% | 4.70% |
| 7 | multicellular organismal process (GO:0032501) | 19 | 19.80% | 11.20% |
| 8 | biological adhesion (GO:0022610) | 5 | 5.20% | 3.00% |
| 9 | locomotion (GO:0040011) | 1 | 1.00% | 0.60% |
| 10 | metabolic process (GO:0008152) | 24 | 25.00% | 14.20% |
| 11 | immune system process (GO:0002376) | 9 | 9.40% | 5.30% |

| hNCC shared | Category name (Accession) | # genes | Percent of gene hit against total # genes | Percent of gene hit against total # Process hits |
|---|---|---|---|---|
| 1 | cellular component organization or biogenesis (GO:0071840) | 42 | 8.00% | 6.10% |
| 2 | cellular process (GO:0009987) | 192 | 36.60% | 27.70% |
| 3 | localization (GO:0051179) | 51 | 9.70% | 7.30% |
| 4 | reproduction (GO:0000003) | 14 | 2.70% | 2.00% |
| 5 | biological regulation (GO:0065007) | 53 | 10.10% | 7.60% |
| 6 | response to stimulus (GO:0050896) | 53 | 10.10% | 7.60% |
| 7 | developmental process (GO:0032502) | 54 | 10.30% | 7.80% |
| 8 | multicellular organismal process (GO:0032501) | 39 | 7.40% | 5.60% |
| 9 | locomotion (GO:0040011) | 4 | 0.80% | 0.60% |
| 10 | biological adhesion (GO:0022610) | 13 | 2.50% | 1.90% |
| 11 | metabolic process (GO:0008152) | 147 | 28.10% | 21.20% |
| 12 | immune system process (GO:0002376) | 31 | 5.90% | 4.50% |
| 13 | cell killing (GO:0001906) | 1 | 0.20% | 0.10% |

| All NB not shared with hNCC | Category name (Accession) | # genes | Percent of gene hit against total # genes | Percent of gene hit against total # Process hits |
|---|---|---|---|---|
| 1 | cellular component organization or biogenesis (GO:0071840) | 125 | 7.90% | 5.20% |
| 2 | cellular process (GO:0009987) | 639 | 40.60% | 26.70% |
| 3 | localization (GO:0051179) | 171 | 10.90% | 7.20% |
| 4 | reproduction (GO:0000003) | 38 | 2.40% | 1.60% |
| 5 | biological regulation (GO:0065007) | 171 | 10.90% | 7.20% |
| 6 | response to stimulus (GO:0050896) | 182 | 11.60% | 7.60% |
| 7 | developmental process (GO:0032502) | 198 | 12.60% | 8.30% |
| 8 | rhythmic process (GO:0048511) | 2 | 0.10% | 0.10% |
| 9 | multicellular organismal process (GO:0032501) | 165 | 10.50% | 6.90% |
| 10 | locomotion (GO:0040011) | 16 | 1.00% | 0.70% |
| 11 | biological adhesion (GO:0022610) | 54 | 3.40% | 2.30% |
| 12 | metabolic process (GO:0008152) | 519 | 33.00% | 21.70% |
| 13 | growth (GO:0040007) | 2 | 0.10% | 0.10% |
| 14 | immune system process (GO:0002376) | 107 | 6.80% | 4.50% |
| 15 | cell killing (GO:0001906) | 1 | 0.10% | 0.00% |
