# Supplementary material for: Genome‐wide DNA methylation analysis identifies MEGF10 as a novel epigenetically repressed candidate tumor suppressor gene in neuroblastoma
Source: Mol Carcinog. 2016 Nov 29;56(4):1290–301. doi: 10.1002/mc.22591 (PMC5396313; doi:10.1002/mc.22591)
Supplement: Supplementary file 7 — supplementary Table S6 [file MC-56-1290-s007.pdf]

Table S6: Summary of properties of hypermethylated and hypomethylated genes

List of genes identified as differentially methylated between neural crest cells (hNCC) and neuroblastoma cell lines using Chipmonk software. Gene methylation levels were derived from the mean probe ratios of the differentially methylated probes within 700bp of the transcriptional start site (log_2). PRC marked indicates those genes marked by PRC2 in ES cells (either Suz12 and/or, Eed and/or H3K27me3 marked; Lee, T. I., R. G. Jenner, et al. (2006) "Control of developmental regulators by Polycomb in human embryonic stem cells." Cell 125(2): 301-313). LCF, ICF and HCF indicate low-, intermediate- or high-CpG promoters, as defined in: Weber, M., I. Hellmann, et al. (2007) "Distribution, silencing potential and evolutionary impact of promoter DNA methylation in the human genome." Nat Genet. 39(4): 457-466.

		Cell line								
	Gene	**hNCC**	**SK-N-AS**	**SHSY-5Y**	**IMR32**	**BE(2)-C**	**PRC marked**	**LCP**	**ICP**	**HCP**
**Hypermethylated**	*ADRA1A*	-0.094	0.486	0.527	0.483	0.67	Y			Y
	ALOX15	-0.51	0.129	0.676	0.451	0.411	Y		Y	
	CHAT	-0.573	0.597	0.463	0.381	0.504			Y	
	CYP26B1	-0.059	0.519	0.253	0.012	0.241	Y			Y
	FAS	-0.473	0.557	0.588	0.507	0.624			Y	
	FOXG1	-0.654	0.279	0.406	0.309	0.407				Y
	GPRC5C	-0.454	0.708	0.779	0.669	0.603				Y
	HISPPD2A	-1.016	0.162	-0.045	0.016	-0.164			Y	
	HOXA11	-0.686	0.336	0.526	0.58	0.288			Y	
	HOXD3	0.084	0.773	0.689	0.556	0.772	Y		Y	
	KCNJ3	-0.417	0.675	0.712	0.46	0.498	Y			Y
	KLHL14	-1.329	0.564	0.619	0.221	0.366	Y		Y	
	LBX1	-0.127	0.323	0.439	0.306	0.36	Y			Y
	MEGF10	-0.743	0.678	0.606	0.518	0.711				
	OSR1	-0.534	0.045	0.177	0.127	0.337	Y			Y
	OTX2	-0.623	0.452	1.367	0.796	0.788	Y		Y	
	PCDHA9	-0.261	0.252	0.934	0.232	0.469		Y		
	RAB38	-0.589	0.428	0.281	0.43	0.532				Y
	RNF220	-1.765	-0.021	-0.617	-0.332	0.218				
	SIX6	-0.525	0.257	-0.45	0.713	0.024	Y			Y
	SLC17A6	-0.599	0.923	0.882	0.741	0.685	Y		Y	
	TBX4	-0.083	0.363	0.738	0.466	0.591				Y
	TFAP2A	-0.835	0.226	0.44	-0.16	0.266				Y
	TGFB2	-0.069	0.751	0.906	0.678	0.676	Y			Y
	ULBP1	-1.22	0.513	0.084	0.281	0.202	Y			Y
	WIT1	-0.899	0.105	0.614	0.499	0.29			Y	
**Hypomethylated**	*ABCA7*	-0.042	-1.232	-1.243	-0.956	-0.949			Y	
	AR	0.37	-0.571	-0.849	-0.509	-0.453			Y	
	ATP13A5	0.221	-1.361	-1.21	-0.416	-1.344		Y		
	CCL3	0.237	-1.143	-1.77	-1.184	-1.009		Y		
	CD69	0.349	-1.259	-0.931	-0.77	-0.825		Y		
	CDYL	0.368	-1.052	-0.743	-0.882	-0.672				
	CLEC4D	0.277	-1.293	-1.217	-0.862	-0.774		Y		
	CTSG	0.288	-0.961	-1.005	-0.997	-0.811		Y		
	CYP2A7	-0.69	-2.392	-2.105	-1.889	-1.989	Y	Y		
	DARC	0.148	-2.054	-1.467	-1.328	-0.793		Y		
	DDR2	0.288	-1.591	-1.604	-1.009	-1.282		Y		
	DEFA1	-0.148	-1.607	-1.926	-1.415	-1.128		Y		
	DEFB104B	0.271	-1.821	-1.792	-0.868	-1.491		Y		
	DEFB128	0.07	-1.459	-1.231	-1.269	-1.119		Y		
	DLC1	0.456	-0.65	-0.404	-0.32	-0.597	Y	Y		
	DPCR1	-0.657	-3.584	-3.675	-3.113	-3.079		Y		
	EYA1	0.339	-0.725	-0.742	-0.629	-0.747			Y	
	FAM103A1	0.281	-0.499	-0.8	-0.414	-0.51				Y
	FCGR2B	0.076	-1.86	-1.618	-1.348	-0.971		Y		
	FRMD4A	0.46	-0.443	-0.624	-0.411	-0.713		Y		
	GATA3	-0.09	-1.868	-1.772	-1.343	-1.554	Y			Y
	GRIK2	0.29	-1.047	-0.983	-0.334	-1.002				Y
	GZMA	0.347	-0.787	-0.879	-0.5	-1.054		Y		
	HBG1	0.32	-1.561	-1.379	-1.054	-0.832		Y		
	HBG2	0.302	-1.304	-0.97	-0.893	-0.666				
	HLA-DPA1	0.376	-1.575	-1.548	-0.977	-1.066		Y		
	HTN1	0.594	-1.019	-0.789	-0.604	-0.659		Y		
	HTN3	0.53	-0.93	-0.899	-0.57	-0.96			Y	
	IGFL2	0.176	-1.943	-0.951	-1.434	-1.069		Y		
	IGFL4	0.155	-1.577	-2.044	-1.363	-1.743		Y		
	KIR3DL3	0.393	-0.952	-1.388	-0.423	-0.789		Y		
	KRTAP4-5	0.489	-0.07	-1.334	-0.94	-1.291		Y		
	LCE1A	-0.166	-1.38	-1.456	-1.338	-1.194		Y		
	LILRA3	0.054	-1.185	-1.12	-0.79	-0.868		Y		
	LILRB2	0.315	-1.953	-1.749	-0.975	-1.431		Y		
	MED12L	0.358	-0.521	-0.503	-0.286	-0.422				
	MGP	0.577	-0.925	-1.173	-0.085	-0.754		Y		
	MYH13	0.562	-0.733	-0.68	-0.652	-0.462		Y		
	MYT1	0.553	-0.626	-1.562	-0.994	-1.357				
	NBEA	0.485	-1.123	-1.278	-1.128	-1.256			Y	
	NLRP11	0.679	-1.287	-1.022	-0.605	0.104		Y		
	OR10G2	0.353	-1.662	-1.943	-1.361	-1.371		Y		
	OR10K2	-0.424	-2.153	-1.617	-1.72	-1.492		Y		
	OR14I1	0.113	-1.299	-0.835	-0.799	-0.884		Y		
	OR1B1	0.27	-2.663	-1.953	-1.363	-1.681		Y		
	OR1D4	0.218	-1.298	-1.565	-1.223	-1.262		Y		
	OR1G1	-0.393	-1.243	-1.747	-1.475	-1.119		Y		
	OR1N1	0.24	-0.834	-0.545	-0.388	-0.959		Y		
	OR2C3	-0.108	-1.468	-1.254	-0.952	-0.911		Y		
	OR2D2	0.364	-1.722	-1.595	-1.393	-1.181		Y		
	OR2T29	0.078	-1.536	-1.098	-1.238	-0.923		Y		
	OR2T3	-0.047	-1.74	-1.244	-1.143	-0.987		Y		
	OR2T34	-0.036	-1.772	-1.146	-1.241	-0.935		Y		
	OR2T5	0.254	-1.721	-1.36	-1.294	-1.125		Y		
	OR3A4	0.683	-0.9	-1.411	-0.599	-0.324		Y		
	OR4D1	0.009	-1.083	-0.948	-1.127	-0.758		Y		
	OR6C70	0.095	-0.799	-0.688	-0.657	-0.664		Y		
	OR6K6	0.387	-1.394	-0.987	-0.991	-0.554		Y		
	OR8D1	0.034	-0.744	-1.229	-0.718	-1.04		Y		
	PATE1	-0.035	-1.204	-1.801	-1.032	-1.75		Y		
	PRB1	-0.251	-1.516	-1.372	-0.869	-1.228		Y		
	RLN2	0.161	-0.401	-0.865	-0.451	-0.665			Y	
	SPINLW1	-0.314	-1.52	-1.401	-1.084	-1.251		Y		
	SPRYD5	-0.053	-1.155	-1.11	-0.876	-1.086		Y		
	TP53TG3	0.708	-0.338	-0.625	-0.012	-0.497			Y	
	UGT2B11	-0.19	-2.051	-1.675	-1.562	-1.684		Y		
	UGT2B28	-0.058	-1.916	-1.492	-1.352	-1.643		Y		
