# Supplementary material for: Genome‐wide DNA methylation analysis identifies MEGF10 as a novel epigenetically repressed candidate tumor suppressor gene in neuroblastoma
Source: Mol Carcinog. 2016 Nov 29;56(4):1290–301. doi: 10.1002/mc.22591 (PMC5396313; doi:10.1002/mc.22591)
Supplement: Supplementary file 8 — supplementary Table S7 [file MC-56-1290-s008.pdf]

Table S7: Gene ontology of differentially methylated genes

Gene ontology of the genes identified by Chipmonk software as being differentially methylated between neural crest and neuroblastoma cell lines - see Table S6 for full list. Gene ontology was assessed using the statistical overrepresentation test in PANTHER (http://www.pantherdb.org/). Only results with P<0.05 are shown.

Hypermethylated genes

PANTHER GO-Slim Biological Process	2006-11-02_HG18_CpG_Promo gene list.txt (REF) #	Client Text Box Input (Hierarchy) #	expected	Fold Enrichment	+/-	P value
Unclassified	6156	3	9.38	0.32	-	0.00E+00
transcription from RNA polymerase II promoter	1082	8	1.65	4.85	+	3.48E-02
developmental process	1807	10	2.75	3.63	+	4.54E-02

Hypomethylated genes

PANTHER GO-Slim Biological Process	2006-11-02_HG18_CpG_Promo gene list.txt (REF) #	Client Text Box Input (Hierarchy) #	expected	Fold Enrichment	+/-	P value
Unclassified	6156	27	27.77	0.97	-	0.00E+00
sensory perception of chemical stimulus	375	17	1.69	10.05	+	2.18E-10
sensory perception	603	18	2.72	6.62	+	3.90E-08
G-protein coupled receptor signaling pathway	500	16	2.26	7.09	+	1.77E-07
sensory perception of smell	255	11	1.15	9.56	+	5.37E-06
response to stimulus	2277	29	10.27	2.82	+	1.85E-05
system process	1264	20	5.7	3.51	+	1.38E-04
neurological system process	1051	18	4.74	3.8	+	1.88E-04
single-multicellular organism process	1641	21	7.4	2.84	+	1.99E-03
multicellular organismal process	1654	21	7.46	2.81	+	2.25E-03
cell surface receptor signaling pathway	1141	17	5.15	3.3	+	2.58E-03
