# Supplementary material for: Genome‐wide DNA methylation analysis identifies MEGF10 as a novel epigenetically repressed candidate tumor suppressor gene in neuroblastoma
Source: Mol Carcinog. 2016 Nov 29;56(4):1290–301. doi: 10.1002/mc.22591 (PMC5396313; doi:10.1002/mc.22591)
Supplement: Supplementary file 9 — supplementary Table S8 [file MC-56-1290-s009.pdf]

Table S8: Hypermethylated genes - survival and expression data

Analysed in R2 (http://r2.amc.nl) using neural crest- Etchevers - GSE14340, neuroblastoma - Versteeg - GSE16476, neuroblastoma cells lines - Versteeg - GSE28019 for expression analysis and Versteeg GSE16476 for survival analysis .

"Y" in NB survival indicates poorer relapse-free survivla patients with low gene expression and p<0.05.

"Y" in NB expression indicates reduced expression in both NB cell lines and NB tumours compared to hNCC with p<0.05.

			R2 neuroblastoma relapse free survival	R2 neuroblastoma expression				
			low expression and poor survival	UP compared to hNCC				
			high expression and poor survival	DOWN compared to hNCC				
			GSE16476	GSE14340	GSE28019	GSE16476		
**Hypermethylated shared hits**	**NB survival**	**NB expression**	**P**	**hNCC median**	**NB cell line median**	**NB tumour median**	**Anova P**	Low expression in NB and NB lines + low = poor survival
ADRA1A			0.048	3.19	2.91	3.01	0.73	
ALOX15			0.076	0.77	1.04	1.20	0.15	
CHAT			0.152	1.54	2.32	1.32	0.12	
CYP26B1			0.001	1.68	0.81	4.14	0.00000047	
FAS		Y	0.018	7.85	4.64	6.34	0.00000000014	
FOXG1		Y	0.265	3.55	0.14	0.49	0.00070	
GPRC5C		Y	0.104	5.83	3.23	4.57	0.020	
HISPPD2A	Y		0.005	4.69	5.88	5.89	0.0015	
HOXA11			0.001	1.00	1.41	1.32	0.050	
HOXD3			0.000	4.25	6.77	6.70	0.000000078	
KCNJ3	Y		0.009	3.28	3.47	4.73	0.0015	
KLHL14	Y		0.031	2.14	2.80	5.05	0.0000000082	
LBX1			0.045	0.00	0.00	0.00	0.62	
MEGF10	Y	Y	0.036	4.80	2.14	3.91	0.0000050	
OSR1		Y	0.015	6.16	0.00	0.58	0.0000011	
OTX2			0.056	3.38	3.04	3.01	0.92	
PCDHA9			0.252	4.39	5.53	7.10	0.00000000042	
RAB38	Y		0.029	3.57	3.22	3.13	0.41	
RNF220	Y		0.047	7.75	7.82	7.70	0.90	
SIX6			0.013	0.38	3.04	2.92	0.06	
SLC17A6			0.004	2.23	2.77	3.63	0.01	
TBX4			0.180	0.49	0.96	1.38	0.06	
TFAP2A	Y		0.034	4.52	2.70	3.95	0.00086	
TGFB2		Y	0.050	10.75	5.18	5.20	################	
ULBP1			0.140	3.32	3.13	3.77	0.0000037	
WIT1		Y	0.080	3.52	3.42	3.33	0.72	
